# Site-Selective Dehydroxy-Chlorination of Secondary Alcohols in Unprotected Glycosides

**DOI:** 10.1021/acs.orglett.2c01992

**Published:** 2022-07-17

**Authors:** Ji Zhang, Niels R. M. Reintjens, Jayaraman Dhineshkumar, Martin D. Witte, Adriaan J. Minnaard

**Affiliations:** Stratingh Institute for Chemistry, University of Groningen, Groningen 9747 AG, The Netherlands

## Abstract

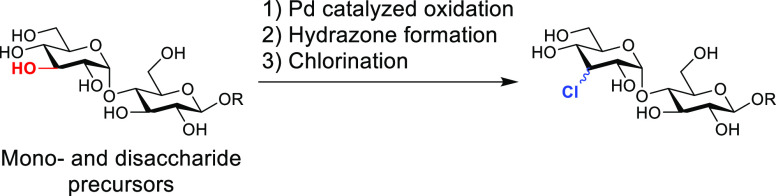

To circumvent protecting groups, the site-selective modification of unprotected glycosides is intensively studied. We show that site-selective oxidation, followed by treatment of the corresponding trityl hydrazone with *tert-*butyl hypochlorite and a H atom donor provides an effective way to introduce a chloride substituent in a variety of mono- and disaccharides. The stereoselectivity can be steered, and a new geminal dichlorination reaction is described as well. This strategy challenges existing methods that lead to overchlorination.

Site-selective modification of carbohydrates is challenging because of the presence of several or multiple hydroxyl groups with similar reactivity. Nonetheless, prior research has shown that it is possible to perform site-selective transformations such as esterification, sulfonylation, silylation, arylation, and alkylation on partially protected, or even unprotected, carbohydrates.^1^ The substitution of a secondary hydroxy group with a halogen in an unprotected carbohydrate is difficult, but it is used in the development of enzyme inhibitors^2,3^ and to prepare starting materials for further substitution^4,5^ and deoxygenation^6,7^ reactions. This transformation is currently achieved by reacting unprotected carbohydrates with sulfuryl chloride or a combination of mesyl chloride and DMF, but this leads to simultaneous chlorination of the primary hydroxy group, producing 3,6-dichlorinated products.^4,5,7−11^ The combination of triphenylphosphine and tetrachloromethane has also been used, but also here the primary hydroxy group is converted into the chloride.^12−14^ Ring-opening of epoxides^15−17^ have been explored; however, their synthesis requires the use of protecting groups, and Payne rearrangement can result in mixtures of epoxides.^15^ Therefore, all-but-one protection of the hydroxy groups, followed by conversion of the remaining hydroxy group into a good leaving group and nucleophilic substitution, is the common path.^18−21^ This, however, becomes especially problematic when a halogen has to be introduced into a disaccharide or oligosaccharide. Furthermore, the removal of protecting groups has to be compatible with the halogen, which means that hydrogenolysis, for example, is hardly an option.

One approach that allows straightforward modification of unprotected glycosides is site-selective oxidation.^22^ Our group and the group of Waymouth have shown that the C3 position in pyranosides can be selectively oxidized with catalytic [(neocuproine)PdOAc]_2_OTf_2_.^23−25^ This method has successfully been applied to 1,4-linked glucans to introduce the keto functionality on the terminal residue.^24^ The keto group can serve as a handle for further modifications, for example in indium mediated allylations or to introduce epoxides.^26^ Moreover, it can be used to form oximes and hydrazones, which can be reduced to the corresponding amine to obtain aminoglycosides.^26,27^ However, it appeared challenging to convert the keto group into a chloride without involving a hydroxy group as an intermediate, which obviously would nullify the site selectivity.

In our search for such a strategy, we realized that hydrazones, readily available from ketones, can react both as electrophiles and nucleophiles,^28,29^ and can be used to functionalize unprotected carbohydrates.^30^ Baldwin et al. demonstrated that lithiated trityl hydrazones act as 1,2-diazaallyl anions and add to aldehydes. The resulting unstable azo-intermediate decomposes at room temperature in a carbon-centered radical that undergoes hydrogen atom transfer in the presence of a thiol (Figure 1A).^31,32^ In 2016, Reyes and Rawal extended this work using *tert*-butyl hypochlorite (*t*BuOCl) as an effective electrophile (Figure 1B).^33^ This leads overall to a rare “dehydroxy-chlorination” procedure.

**Figure 1:**
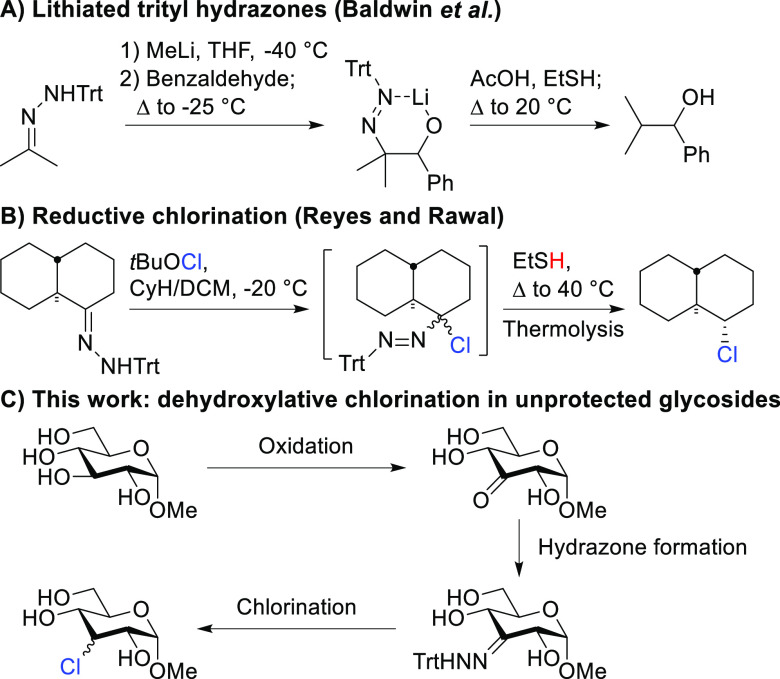
Trityl hydrazones as carbon nucleophiles.

With the regioselective oxidation and subsequent hydrazone formation in place, we realized that this strategy could meet the challenge to selectively introduce a chloride substituent in glycosides as this procedure does not have a hydroxy group as an intermediate (Figure 1C).

To investigate this strategy, previously reported keto-GlcNAc **1a** and keto-glucoside **1b**(34,35) were converted into the corresponding *E*/*Z* trityl hydrazones **2a** and **2b** (Figure 2). Subsequently, GlcNAc derivative **2a** was studied first in the dehydroxy-chlorination reaction and was reacted with freshly prepared *t*BuOCl at −20 °C. The formed unstable azo-intermediate **2a′** was subjected to thermolysis using the conditions reported by Reyes and Rawal^33^ (40 °C, 80 equiv of ethanethiol (EtSH)), which afforded **3a** in an excellent 80% yield (Figure 2A). This showed our hypothesis to be correct. NMR analysis established the structure of **3a** as a mixture of epimers (eq/ax: 1.4/1) with a slight preference for the equatorial chloride. The configuration of **3a**-ax was confirmed by its X-ray structure.

**Figure 2:**
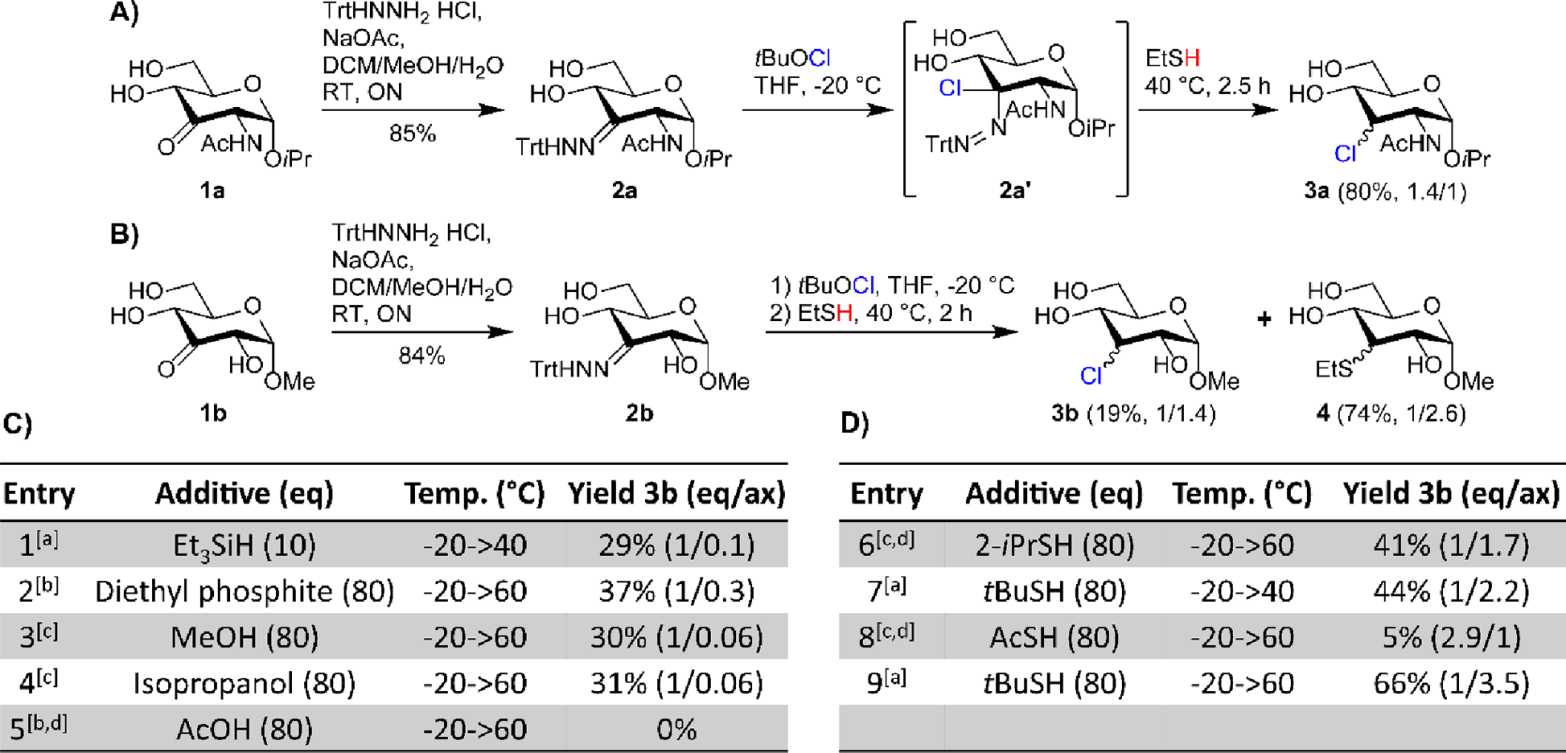
Dehydroxy-chlorination of GlcNAc (A) and Glc (B). Optimization of H donor (C) and the thermolysis conditions (D). The eq/ax ratio was determined by NMR analysis. The additive was added at −20 °C and thermolysis was performed at the indicated temperature for ^[a]^1 h, ^[b]^1.5 h, or ^[c]^2 h. ^[d]^Substitution products were also isolated (Supporting Information).

Excited about this result, we applied the same reaction conditions to glucoside **2b**, which, to our surprise gave only 19% of the expected epimeric mixture of 3-chloro glycoside **3b**. Instead, thioether **4** was isolated as the major product (74%) (Figure 2B). To circumvent thioether formation, we attempted the thermolysis reaction in the presence of triethylsilane, diethyl phosphite, methanol, 2-propanol, or acetic acid (Figure 2C). Four of these gave the desired chloride in low yield, but with good to excellent selectivity for the equatorial product. Dissatisfied by the yield, we attempted to avoid thioether formation by increasing the steric bulk of the thiol (Figure 2D). The yield of desired chloride **3b** increased to 41% when thermolysis was performed with 2-propanethiol (*i*PrSH) (entry 6). However, considerable amounts of thioether product were still isolated from the reaction mixture. The use of *tert*-butyl thiol (*t*BuSH) (entry 7) as the H atom donor completely circumvented thioether formation. Increasing the steric bulk of the thiol also had an effect on the stereochemical outcome of the reaction. The product ratio shifted slightly in favor of the axial chloride (eq/ax: 1/1.4 (EtSH), 1/1.7 (*i*PrSH), 1/2.2 (*t*BuSH)). Thermolysis with thioacetic acid (AcSH) was also tried. However, this gave a minimal amount of **3b** and, as expected, formation of the thioacetyl product (entry 8).

We also investigated the influence of the effect of the temperature on the thermolysis and found that a temperature of 60 °C led to a considerably higher isolated yield (66%) and to a higher selectivity for the axial chloride (from 1/2.2 to 1/3.5; entries 7 and 9).

With these results in hand, we studied the substrate scope of the reaction (Figure 3). We prepared keto-sugars **1c**–**1f** via [(neocuproine)PdOAc]_2_OTf_2_ oxidation, with the exception of the 4-keto sugar **1c** which was prepared with Oc_2_SnCl_2_ and Br_2_.^35,36^ The keto-sugars were converted into the corresponding trityl hydrazones **2c**–**2f** and reacted with *t*BuOCl. Thermolysis with the optimized conditions gave chlorides **3c**–**3f** after column purification in good yields (65–74%). The major product invariably had the chloride substituent in the axial position, and in a number of cases the epimers could be separated by column chromatography. The isolated yields may be increased by omitting the column purification step, as was exemplified for 3-deoxy-3-chloro glycoside **3b**. On the 1 mmol scale, the *t*BuSH and trityl residues were readily removed by extraction, giving the desired pure **3b** in an excellent 92% isolated yield. Especially the synthesis of disaccharides **3e** and **3f** in high yields shows the strength of this selective dehydroxy-chlorination approach, compared to the known chlorination methods in the literature.

**Figure 3:**
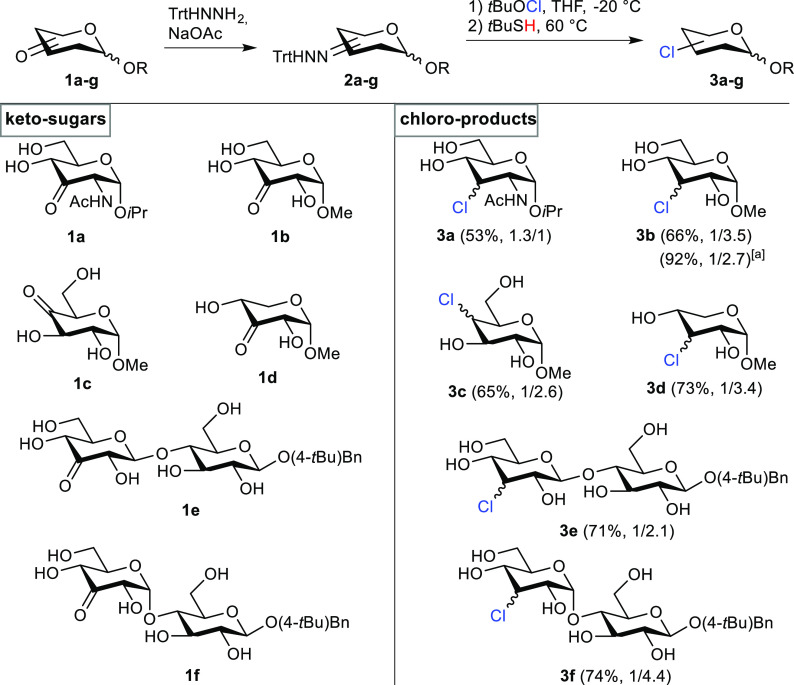
Substrate scope. In brackets the yield for the chlorination step and the equatorial/axial ratio are given. The reaction time was 1 h for **3b**–**3f** and 2.5 h for **3a**. The eq/ax ratio was determined by NMR analysis. ^[a]^92% yield was obtained via extraction.

Although *t*BuSH gave the desired chloro-glycosides in good yields, a major disadvantage is its obnoxious smell. Therefore, we performed the reaction with **2b** using adamantanethiol and *tert*-nonyl mercaptan (*tert*-nonylSH) instead of *t*BuSH. These H atom donors gave chloro-glycoside **3b** in 72% and 58% yield with again a higher selectivity for the axial chloride (entries 1 and 2, Table S1) and could be used as less odorous alternatives for *t*BuSH.

As an alternative approach, we performed thermolysis with lower amounts of thiol. Because lowering the thiol concentration might suppress the formation of substitution products, we also used AcSH and EtSH. When we subjected the in situ formed azo-intermediate of **2b** to 4 equiv of thiol (entries 3–5, Table S1), the desired product **3b** was isolated in moderate to good yield, without noticeable amounts of substitution products. Interestingly, the chloride substitution product **3b** was obtained in 67% yield with an equatorial/axial ratio of 1/1.1 on a 2.5 mmol scale with AcSH as the H atom donor. This shows that when the equatorial chloride is desired, 4 equiv of AcSH can be used instead of *t*BuSH (e.g., the eq/ax ratio shifts from 1/3.5 to 1/1.1).

Finally, we investigated whether the reaction with **2b** could be performed in MeOH (entries 6 and 7, Table S1), because minimally protected oligosaccharides have limited solubility in THF. Chloride **3b** was not formed, but surprisingly, α,α-dichloro compound **5** was isolated in 22% yield (Figure 4). The yield of **5** increased to 34% when 2.2 equiv of *t*BuOCl was used. Although the yield is not yet up to standards, this finding, which is currently under study, provides potentially an efficient entry into this motif in carbohydrate chemistry. *gem*-Dihalo glycosides are rare but are used in nucleoside chemistry as viral reverse transcriptase inhibitors.^37^

**Figure 4:**
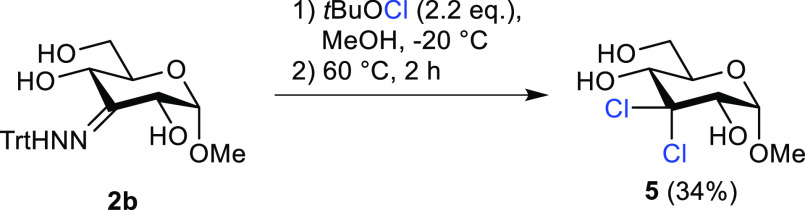
Chlorination of **2b** in methanol.

We demonstrated already in earlier work that nucleophilic attack on the keto group in 3-ketoglucoside **2b** takes place preferentially from the top face.^26^ Similarly, epoxidation of the corresponding exocyclic alkylidene with dimethyldioxirane takes place from the top face.^38,39^ In both cases, attack via the bottom face is less favorable because of a 1,3-diaxial interaction with the methoxy substituent on the anomeric center. We hypothesized that the stereoselectivity of both steps in the dehydroxy-chlorination reaction, and so chloride introduction and hydrogen atom-transfer, is also determined by 1,3-diaxial interactions and that the second step will determine the final stereochemical outcome (vide infra). To underpin this hypothesis, the 2,4-dinitrophenyl hydrazones of methyl α- and β-glycosides **6a** and **6b** were prepared. Treatment of these hydrazones with *t*BuOCl gave stable azo-intermediates (Figure 5A/B). NMR analysis revealed that **6a** cleanly converted into a single azo-product **7a**, whereas β-glycoside **6b** gave a mixture of the two epimeric products, **7b**. This result confirmed that 1,3-diaxial interactions indeed determine the stereochemical outcome of the reaction with *t*BuOCl. On the basis of these results, we argue that treatment of the trityl hydrazone of α-glucoside **2b** with *t*BuOCl will also predominantly form a single azo-intermediate (**I**), as is shown in Figure 5C.^40^

**Figure 5:**
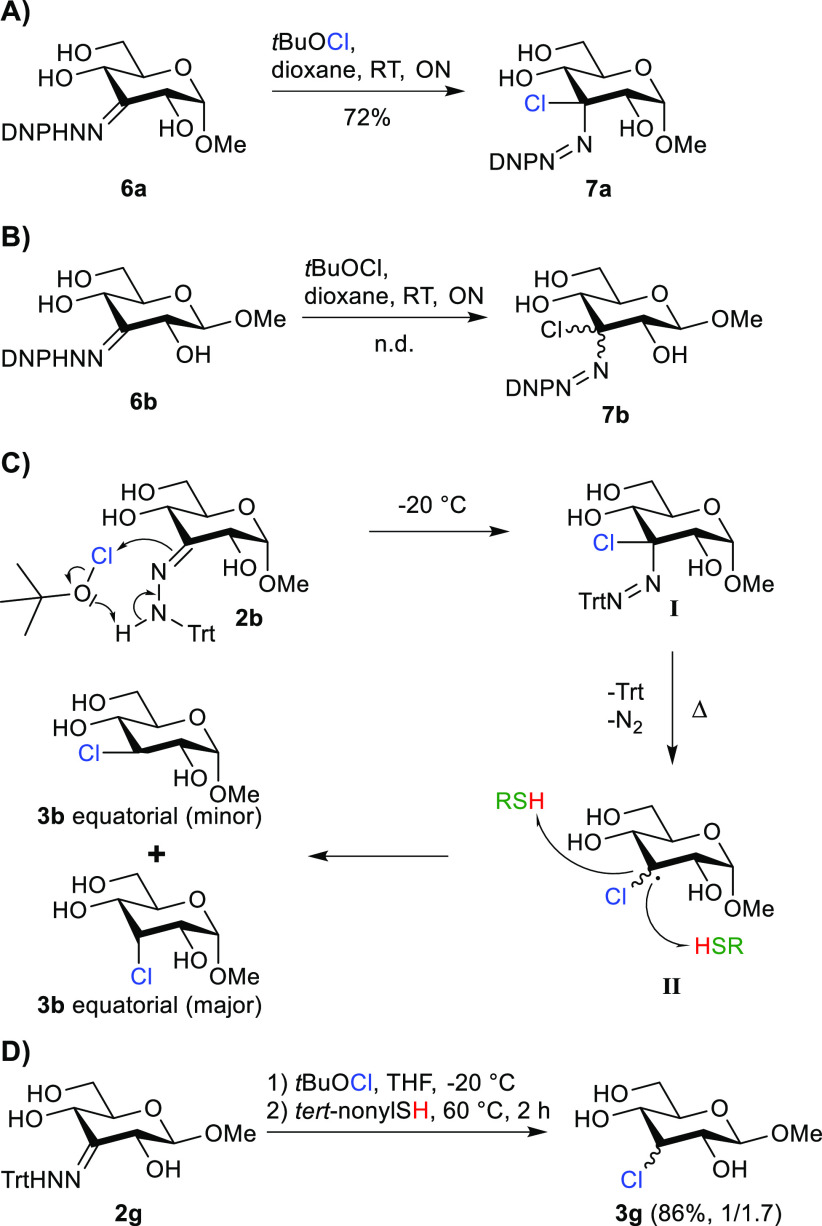
(A/B) Synthesis of the azo-intermediates **7a** and **7b**. The yield of **7b** could not be determined because it could not be isolated from **6b**. (C) Proposed mechanism for the dehydroxy-chlorination reaction. (D) Synthesis of **3g**. The eq/ax ratio was determined by NMR analysis.

Because **3b** is obtained as a mixture of equatorial and axial chloride after the thermolysis step, we conclude that, after extrusion of dinitrogen and the trityl radical, the final stereochemical outcome is determined in the hydrogen atom-transfer step. This reasoning is supported by the observation that increasing the steric bulk of the thiol H atom donor affects the stereochemical outcome of the reaction (vide supra). The H atom donor can approach the carbon-centered radical at C3 either via the top or the bottom face of **II** (Figure 5). The face selectivity is determined by the axial substituent on C1 and the steric bulk of the H atom donor. Approach from the least hindered side (the top side) is more favorable for α-glucosides, especially in combination with bulky thiol H atom donors and this leads to preferential formation of the axial chlorides as we observed in the experiments.

In β-glucosides, there is less hindrance from the bottom face, and these substrates should lead to the formation of more equatorial chloride. Therefore, we also chlorinated β-glucoside **2g** (Figure 5D). Chloride **3g** was isolated in 84% yield with a lower stereoselectivity (1/1.7) compared to the reaction with α-glucoside **2b** 1/3.9 (entry 2, Table S1), further confirming that the anomeric configuration influences the stereochemical outcome.

In conclusion, we have developed a novel approach for the site-selective dehydroxy-chlorination of glycosides. Our methodology involves site-selective oxidation, followed by conversion of the ketone to the corresponding trityl hydrazone. Chlorination of this trityl hydrazone with *t*BuOCl and subsequent thermolysis of the intermediate with a suitable H atom donor give the desired chloro-sugars as mixtures of the equatorial and the axial chloride. The choice of H atom donor is crucial to obtain a high yield in the chlorination reaction and steers the stereoselectivity. A bulky thiol such as *tert*-butyl thiol and *tert*-nonyl mercaptan leads to a preference for the axial chloride substituent, whereas the use of 4 equiv of AcSH provides a near one to one mixture of epimers. The dehydroxy-chlorination of the disaccharides cellobiose and maltose, in high yield, shows that this method can be extended beyond monosaccharides for the selective introduction of a chloride in glycosides, which is a considerable advantage over existing methods. Apart from the (known) utility of chlorosugars per se, we are currently studying the reactivity of the chloro substituent, in particular in nucleophilic substitution reactions. The serendipitously observed geminal dichlorination of substrate **2b** will elicit further study on this reaction as well.

## Supporting Information Available

Detailed experimental procedures, synthesis diagrams, X-ray structures of compounds **1d**, **2d**, **3a**, and **3b**, and copies of NMR and HRMS spectra (PDF)

## Accession Codes

CCDC 2150508, 2150537, and 2150550–2150551 contain the supplementary crystallographic data for this paper. These data can be obtained free of charge via www.ccdc.cam.ac.uk/data_request/cif, or by emailing data_request@ccdc.cam.ac.uk, or by contacting The Cambridge Crystallographic Data Centre, 12 Union Road, Cambridge CB2 1EZ, UK; fax: +44 1223 336033.

## References

[ref1] Dimakos V.; Taylor M. S. Site-Selective Functionalization of Hydroxyl Groups in Carbohydrate Derivatives. Chem. Rev. 2018, 118 (23), 11457–11517. 10.1021/acs.chemrev.8b00442. 30507165

[ref2] Woodward G. E.; Cramer F. B.; Hudson M. T. Carbohydrate Analogs as Antagonists of Glucose in Carbohydrate Metabolism of Yeast. J. Franklin Inst. 1953, 256 (6), 577–587. 10.1016/0016-0032(53)91167-3.

[ref3] Auzanneau F.-I.; Bundle D. R. Synthesis of Chlorodeoxy Trisaccharides Related to the Shigella Flexneri Y Polysaccharide. Carbohydr. Res. 1993, 247, 195–209. 10.1016/0008-6215(93)84252-2. 7693346

[ref4] Khan R.; Bosco M.; Konowicz P. A.; Stucchi L.; Rizzo R. Synthesis of 6-Deoxy-6-Halolaminarans and Conversion of 6-Chloro-6-Deoxylaminaran into the 6-Amino-6-Deoxy Derivative. Carbohydr. Res. 1996, 292, 39–46. 10.1016/S0008-6215(96)91021-6.

[ref5] Gao C.; Liu S.; Edgar K. J. Regioselective Chlorination of Cellulose Esters by Methanesulfonyl Chloride. Carbohydr. Polym. 2018, 193, 108–118. 10.1016/j.carbpol.2018.03.093. 29773362

[ref6] Wheatley D. E.; Fontenelle C. Q.; Kuppala R.; Szpera R.; Briggs E. L.; Vendeville J.-B.; Wells N. J.; Light M. E.; Linclau B. Synthesis and Structural Characteristics of All Mono- and Difluorinated 4,6-Dideoxy-d-*Xylo* -Hexopyranoses. J. Org. Chem. 2021, 86 (11), 7725–7756. 10.1021/acs.joc.1c00796. 34029099

[ref7] van Summeren R. P.; Feringa B. L.; Minnaard A. J. New Approaches towards the Synthesis of the Side-Chain of Mycolactones A and B. Org. Biomol. Chem. 2005, 3 (14), 2524–2533. 10.1039/b505980a. 15999184

[ref8] Bragg P. D.; Jones J. K. N.; Turner J. C. The reaction of sulphuryl chloride with glycosides and sugar alcohols. Part I. Can. J. Chem. 1959, 37 (9), 1412–1416. 10.1139/v59-207.

[ref9] Street I. P.; Armstrong C. R.; Withers S. G. Hydrogen Bonding and Specificity. Fluorodeoxy Sugars as Probes of Hydrogen Bonding in the Glycogen Phosphorylase-Glucose Complex. Biochemistry 1986, 25 (20), 6021–6027. 10.1021/bi00368a028. 3790503

[ref10] Velvadapu V.; Andrade R. B. Concise Syntheses of D-Desosamine, 2-Thiopyrimidinyl Desosamine Donors, and Methyl Desosaminide Analogues from d-Glucose. Carbohydr. Res. 2008, 343 (1), 145–150. 10.1016/j.carres.2007.10.004. 17977522

[ref11] Edwards R. G.; Hough L.; Richardson A. C.; Tarelli E. The Stereoselective Replacement of Hydroxyl Groups by Chlorine, Using the Mesyl Chloride-N,N-Dimethylformamide Reagent. Carbohydr. Res. 1974, 35 (1), 111–129. 10.1016/S0008-6215(00)84839-9.

[ref12] Khan R.; Patel G. Halogenation Reactions of Derivatives Of d-Glucose and Sucrose. Carbohydr. Res. 1990, 205, 211–223. 10.1016/0008-6215(90)80141-O. 2276136

[ref13] Limousin C.; Olesker A.; Cléophax J.; Petit A.; Loupy A.; Lukacs G. Halogenation of Carbohydrates by Triphenylphosphine Complex Reagents in Highly Concentrated Solution under Microwave Activation or Conventional Heating. Carbohydr. Res. 1998, 312 (1–2), 23–31. 10.1016/S0008-6215(98)00224-9.

[ref14] Anizon F.; Moreau P.; Sancelme M.; Laine W.; Bailly C.; Prudhomme M. Rebeccamycin Analogues Bearing Amine Substituents or Other Groups on the Sugar Moiety. Bioorg. Med. Chem. 2003, 11 (17), 3709–3722. 10.1016/S0968-0896(03)00343-2. 12901916

[ref15] Buchanan J. G. 511. The Behaviour of Derivatives of 3:4-Anhydrogalactose towards Acidic Reagents. Part II. J. Chem. Soc. 1958, 2511–2516. 10.1039/jr9580002511.

[ref16] Afza N.; Malik A.; Voelter W. A Regioselective Synthesis of 3-Chloro-3-Deoxy Sugars by Dichlorobis(Benzonitrile)Palladium(II). J. Chem. Soc. Perkin Trans. 1 1983, 1349–1351. 10.1039/p19830001349.

[ref17] Umemura E.; Tsuchiya T.; Kobayashi Y.; Tanaka K. A Synthetic Study of Methyl 3-Deoxy-3-Fluoro-α-d-Glucopyranosides from Methyl 2,3-Anhydro-α-d-Allopyranosides, and Synthesis of 3′-Deoxy-3′-Fluorokanamycin A and 3′-Chloro-3′-Deoxykanamycin A. Carbohydr. Res. 1992, 224, 141–163. 10.1016/0008-6215(92)84101-W. 1591758

[ref18] Binkley R. W.; Ambrose M. G.; Hehemann D. G. Synthesis of Deoxyhalogeno Sugars. Displacement of the (Trifluoromethanesulfonyl)Oxy (Triflyl) Group by Halide Ion. J. Org. Chem. 1980, 45 (22), 4387–4391. 10.1021/jo01310a025.

[ref19] Chowdhary M. S.; Hough L.; Richardson A. C. The Use of Pivalic Esters of Sucrose for the Synthesis of Chloro, Azido, and Anhydro Derivatives. Carbohydr. Res. 1986, 147 (1), 49–58. 10.1016/0008-6215(86)85006-6.

[ref20] Krejzová J.; Kalachova L.; Šimon P.; Pelantová H.; Slámová K.; Křen V. Inhibition of Microbial β-N-Acetylhexosaminidases by 4-Deoxy- and Galacto-Analogues of NAG-Thiazoline. Bioorg. Med. Chem. Lett. 2014, 24 (22), 5321–5323. 10.1016/j.bmcl.2014.09.066. 25442323

[ref21] Azad C. S.; Saxena A. K. One Pot Conversion of Carbohydrates Alcohol into Chloride via Benzotriazole Sulfonate. Tetrahedron 2013, 69 (12), 2608–2612. 10.1016/j.tet.2013.01.044.

[ref22] Gorelik D. J.; Dimakos V.; Adrianov T.; Taylor M. S. Photocatalytic, Site-Selective Oxidations of Carbohydrates. Chem. Commun. 2021, 57, 12135–12138. 10.1039/D1CC05124E. 34723300

[ref23] Jäger M.; Hartmann M.; de Vries J. G.; Minnaard A. J. Catalytic Regioselective Oxidation of Glycosides. Angew. Chemie Int. Ed. 2013, 52 (30), 7809–7812. 10.1002/anie.201301662. 23780519

[ref24] Eisink N. N. H. M.; Lohse J.; Witte M. D.; Minnaard A. J. Regioselective Oxidation of Unprotected 1,4 Linked Glucans. Org. Biomol. Chem. 2016, 14 (21), 4859–4864. 10.1039/C6OB00608F. 27159790

[ref25] Chung K.; Waymouth R. M. Selective Catalytic Oxidation of Unprotected Carbohydrates. ACS Catal. 2016, 6 (7), 4653–4659. 10.1021/acscatal.6b01501.

[ref26] Marinus N.; Tahiri N.; Duca M.; Mouthaan L. M. C. M.; Bianca S.; van den Noort M.; Poolman B.; Witte M. D.; Minnaard A. J. Stereoselective Protection-Free Modification of 3-Keto-Saccharides. Org. Lett. 2020, 22 (14), 5622–5626. 10.1021/acs.orglett.0c01986. 32635733 PMC7372562

[ref27] Zhang J.; Eisink N. N. H. M.; Witte M. D.; Minnaard A. J. Regioselective Manipulation of GlcNAc Provides Allosamine, Lividosamine, and Related Compounds. J. Org. Chem. 2019, 84 (2), 516–525. 10.1021/acs.joc.8b01949. 30569712 PMC6343366

[ref28] de Gracia Retamosa M.; Matador E.; Monge D.; Lassaletta J. M.; Fernández R. Hydrazones as Singular Reagents in Asymmetric Organocatalysis. Chem. - A Eur. J. 2016, 22 (38), 13430–13445. 10.1002/chem.201602430. 27552942

[ref29] Li J.; Huang C.; Li C. Deoxygenative Functionalizations of Aldehydes, Ketones and Carboxylic Acids. Angew. Chem. Int. Ed. 2022, 61 (10), e202112770 10.1002/anie.202112770. 34780098

[ref30] Kan J.; Chen Z.; Qiu Z.; Lv L.; Li C.; Li C.-J. Umpolung Carbonyls Enable Direct Allylation and Olefination of Carbohydrates. Sci. Adv. 2022, 8 (10), eabm6840 10.1126/sciadv.abm6840. 35263121 PMC8906572

[ref31] Baldwin J. E.; Bottaro J. C.; Kolhe J. N.; Adlington R. M. Azo Anions in Synthesis. Use of Trityl- and Diphenyl-4-Pyridylmethyl-Hydrazones for Reductive C–C Bond Formation from Aldehydes and Ketones. J. Chem. Soc., Chem. Commun. 1984, (1), 22–23. 10.1039/C39840000022.

[ref32] Baldwin J. E.; Adlington R. M.; Bottaro J. C.; Jain A. U.; Kolhe J. N.; Perry M. W. D.; Newington I. M. Michael Additions of Hydrazones for Carbon–Carbon Bond Formation. J. Chem. Soc., Chem. Commun. 1984, (16), 1095–1096. 10.1039/C39840001095.

[ref33] Reyes J. R.; Rawal V. H. Reductive Chlorination and Bromination of Ketones via Trityl Hydrazones. Angew. Chemie Int. Ed. 2016, 55 (9), 3077–3080. 10.1002/anie.201510909. PMC9078849 26823122

[ref34] Zhang J.; Eisink N. N. H. M.; Witte M. D.; Minnaard A. J. Regioselective Manipulation of GlcNAc Provides Allosamine, Lividosamine, and Related Compounds. J. Org. Chem. 2019, 84 (2), 516–525. 10.1021/acs.joc.8b01949. 30569712 PMC6343366

[ref35] Eisink N. N. H. M.; Witte M. D.; Minnaard A. J. Regioselective Carbohydrate Oxidations: A Nuclear Magnetic Resonance (NMR) Study on Selectivity, Rate, and Side-Product Formation. ACS Catal. 2017, 7 (2), 1438–1445. 10.1021/acscatal.6b03459. 28367353 PMC5370080

[ref36] Muramatsu W. Catalytic and Regioselective Oxidation of Carbohydrates To Synthesize Keto-Sugars under Mild Conditions. Org. Lett. 2014, 16 (18), 4846–4849. 10.1021/ol502344h. 25198882

[ref37] Li Y.; Li P.; Li Y.; Zhang R.; Yu P.; Ma Z.; Kainov D. E.; de Man R. A.; Peppelenbosch M. P.; Pan Q. Drug Screening Identified Gemcitabine Inhibiting Hepatitis E Virus by Inducing Interferon-like Response via Activation of STAT1 Phosphorylation. Antiviral Res. 2020, 184, 104967 10.1016/j.antiviral.2020.104967. 33137361

[ref38] Yoshimura J.; Sato K.; Kobayashi K.; Shin C. Branched-Chain Sugars. II. On the Configuration of Branched-Chain Sugars from Methyl 2- O -Benzoyl-4,6- O -Benzylidene-α-D-Ribo-Hexopyranosid-3-Ulose. Bull. Chem. Soc. Jpn. 1973, 46 (5), 1515–1519. 10.1246/bcsj.46.1515.

[ref39] Rosenthal A.; Khong-Seng O. Synthesis of Branched-Chain Nitro and Amino Sugars via the Nitromethane Synthesis. Tetrahedron Lett. 1969, 10 (45), 3981–3983. 10.1016/S0040-4039(01)88592-0.

[ref40] We were unable to isolate and analyze this intermediate because of its instability.

