# Supplementary material for: Site-Selective Dehydroxy-Chlorination of Secondary Alcohols in Unprotected Glycosides
Source: Org Lett. 2022 Jul 17;24(29):5339–44. doi: 10.1021/acs.orglett.2c01992 (PMC9490796; doi:10.1021/acs.orglett.2c01992)

Supporting information

Site-selective dehydroxy-chlorination of secondary alcohols in unprotected glycosides

Ji Zhang[ǂ], Niels R.M. Reintjens[ǂ], Jayaraman Dhineshkumar, Martin D. Witte*, Adriaan J. Minnaard*

Stratingh Institute for Chemistry, University of Groningen, Nijenborgh 7, 9747 AG Groningen, the Netherlands

[ǂ]Authors have contributed equally.

*Correspondence: m.d.witte@rug.nl and a.j.minnaard@rug.nl

Experimental procedures **S-5**

Synthesis of GlcNAc derivatives S-5

Synthesis of α-Glc derivatives S-6

Synthesis of Gal derivatives S-9

Synthesis of Xyl derivatives S-11

Synthesis of cellobiose derivatives S-12

Synthesis of maltose derivatives S-15

Synthesis of β-Glc derivatives S-17

Scale-up synthesis **S-19**

Crystal structures **S-20**

Supplemental references **S-24**

NMR spectraNMR spectra of GlcNAc derivatives S-25

NMR spectra of α-Glc derivatives S-32

NMR spectra of Gal derivatives S-57

NMR spectra of Xyl derivatives S-63

NMR spectra of cellobiose derivatives S-74

NMR spectra of β-Glc derivatives S-107

HRMS spectra **S-119**

HRMS spectra of GlcNAc derivatives S-119

HRMS spectra of α-Glc derivatives S-122

HRMS spectra of Gal derivatives S-132

HRMS spectra of Xyl derivatives S-134

HRMS spectra of cellobiose derivatives S-138

HRMS spectra of maltose derivatives S-145

HRMS spectra of β-Glc derivatives S-152

Supplementary schemes

Table S1. Site-selective dehydroxy-chlorination of secondary alcohols in unprotected glycosides

Entry	Substrate	H-donor (eq.)	Temp. (°C)	Yield 3-Cl (Eq/Ax)	Yield 3-X (Eq/Ax)
1	**2b** α-Glc	Adamantanethiol (20)	-20->60	72% (1/4.4)	-
2	**2b** α-Glc	Tert-nonyl mercaptan (80)	-20->60	58% (1/3.9)	-
3	**2b** α-Glc	tBuSH (4)	-20->60	44% (1/2.5)	-
4	**2b** α-Glc	EtSH (4)	-20->40	51% (1/1.3)	-
5	**2b** α-Glc	AcSH (4)	-20->60	67% (1/1)	-
6[a]	**2b** α-Glc	*t*BuSH (80)	-20->60	-	22% (**5**)
7[b]	**2b** α-Glc	-	-20 -> 40	-	34% (**5**)

The eq/ax ratios were determined by NMR analysis.

[a]MeOH was used as solvent instead of THF.

[b]MeOH was used as solvent instead of THF and 2.2 eq. were used instead of 1.1 eq. *t*BuOCl. 27% of **1b** was also isolated.

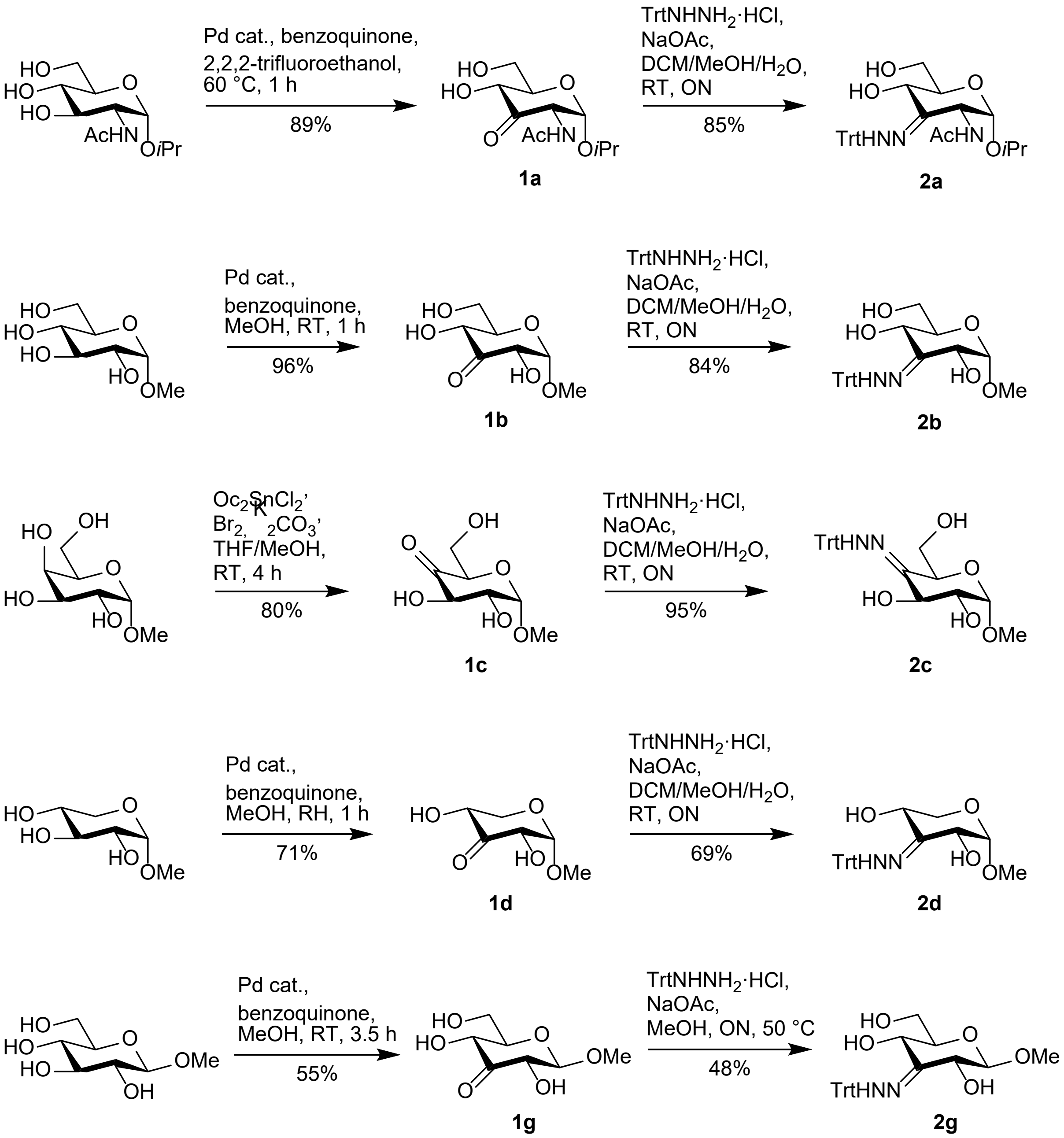

Scheme S1. Synthesis of hydrazones **2a**-**2d** and **2g**. The synthesis of **1a** was reported earlier.[1]

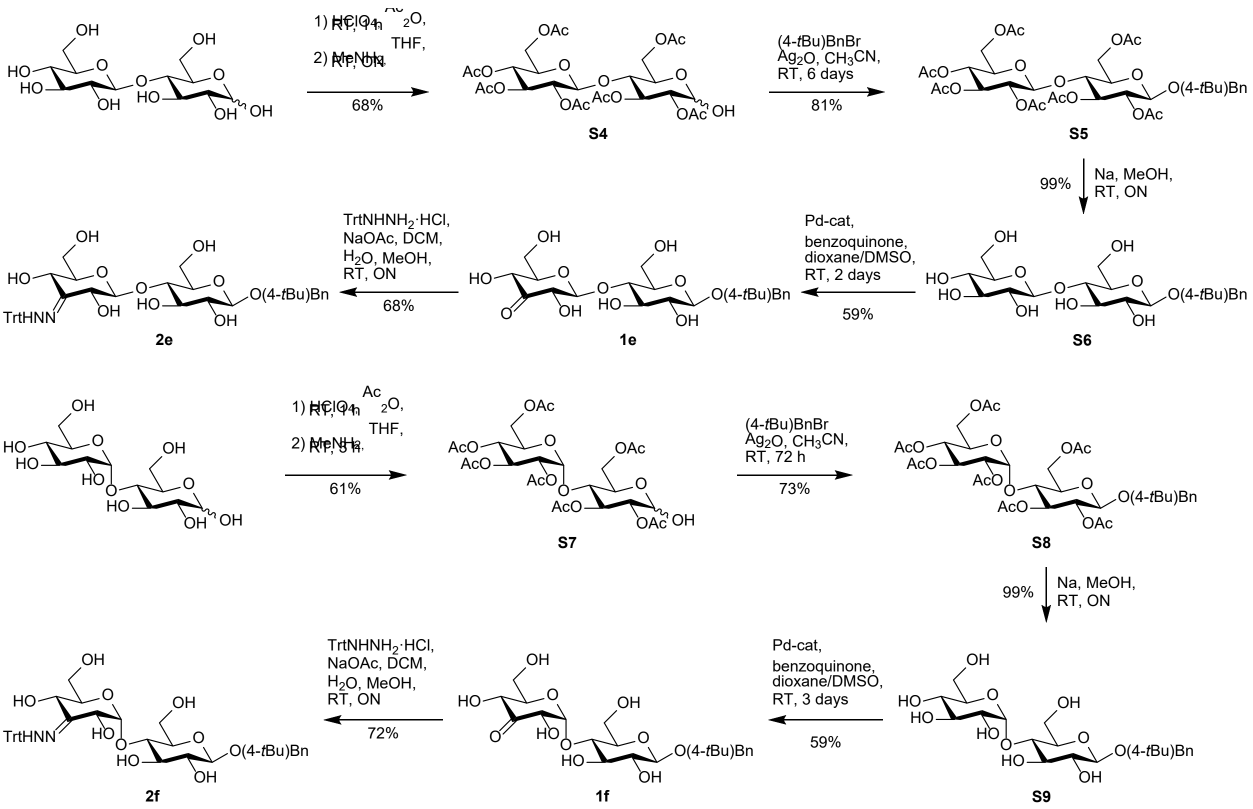

Scheme S2. Synthesis of hydrazones **2e** and **2f**.

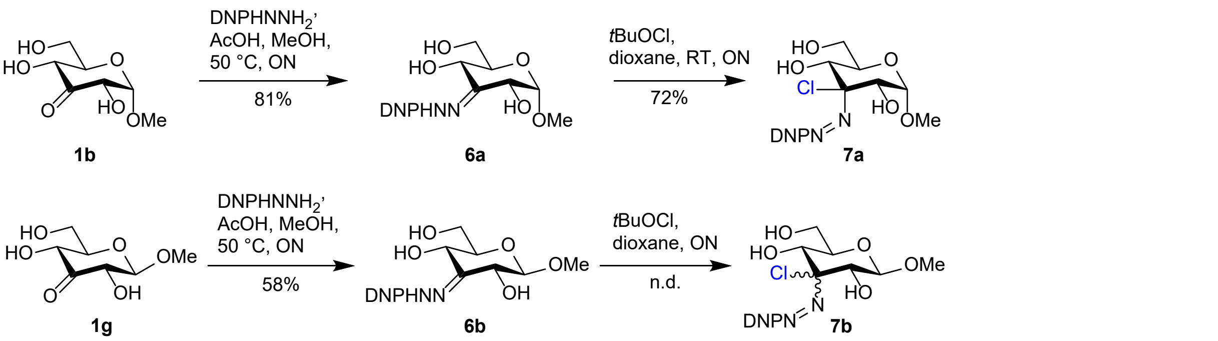

Scheme S3. Synthesis of the azo-compounds **7a** and **7b**. The yield of **7b** could not be determined since an inseparable mixture of **6b** and **7b** was obtained.

Experimental procedures

General Information

All solvents used for reaction, extraction, filtration, and chromatography were of commercial grade and used without further purification. [(neocuproine)Pd(μ-OAc)]$_2$(OTf)$_2$ was prepared according to the literature procedure.[2] Automated flash chromatography was performed on a Reveleris® X2 Flash Chromatography, using Grace® Reveleris Silica flash cartridges (4 grams, 12 grams, 15 grams, 24 grams, 40 grams, 80 grams, and 120 grams). ^{1}H-, ^{13}C-, APT-, HSQC-, and COSY-NMR were recorded on a Varian AMX400 spectrometer (400, 101 MHz, respectively) using DMSO-*d*$_6$, chloroform-*d*, methanol-*d*$_4$, or acetonitrile-*d*$_3$ as solvent. Chemical shifts are given in ppm (δ) relative to the solvent residual peak. Data are reported as follows: chemical shifts (δ), multiplicity (s = singlet, d = doublet, dd = double doublet, ddd = double double doublet, t = triplet, q =quartet, m = multiplet), coupling constants J (Hz), and integration. High Resolution Mass measurements were performed using a ThermoScientific LTQ OribitrapXL spectrometer, negative modes were measures in the presence of guanidinium chloride. For the analysis of the crystal structures, the following software was used: Bruker, (2016); *APEX3* (v2016.1-0), *SAINT* (Version 8.37A) and *SADABS* (Version 2014/5); Bruker AXS Inc., Madison, Wisconsin, USA

Preparation and titration of *tert*-butyl hypochlorite

10-15% aqueous NaOCl (100 mL) was diluted with water (150 mL). The solution was cooled to 0 °C and placed in the dark. AcOH (10 mL) and *t*BuOH (15.5 mL) were added in one single portion. The solution was stirred for 15 min. The formed *tert*-butyl hypochlorite formed an immiscible layer on top of the water. The reaction mixture was poured into a separatory funnel and the aqueous layer was discarded. The neat *tert*-butyl hypochlorite layer was washed with sat. aq. $NaHCO_3$ (1× 100 mL), water (1× 50 mL) and filtered over a pipet filled with $CaCl_2$. Neat yellow *tert*-butyl hypochlorite was obtained, which was stored over $CaCl_2$ under N_2 atmosphere in a fridge for a maximum period of one month. When used in a reaction, it was dissolved in the solvent mentioned.

Synthesis of GlcNAc derivatives

Isopropyl-2-acetamido-2-deoxy-3-(trityl)hydrazone-α-D-glucopyranoside (2a)

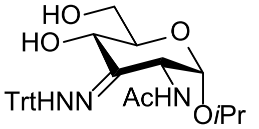

TrtNHNH$_2$·HCl (6.21 g, 20.0 mmol, 2.0 eq.), NaOAc·3H$_2$O (1.6 g, 20 mmol, 2.0 eq.), and isopropyl 2-acetamido-2-deoxy-3-keto-α-D-glucopyranoside[1] (1.16 g, 4.44 mmol, 1.0 eq.) were dissolved in dichloromethane (DCM) (77 mL), water (6.2 mL) and methanol (25 mL). The flask was subsequently evacuated and backfilled with nitrogen three times. Under N_2 flow, shielded from light, the reaction was stirred at ambient temperature overnight. Subsequently, the solvent was evaporated under reduced pressure. Purification by automated flash chromatography on an 80 g silica cartridge with heptane/EtOAc (0 to 100% EtOAc in heptane) provided the product (4.4 g, 8.5 mmol, 85%) as a yellow solid. HRMS (ESI pos) m/z calcd for $C_{30}H_{36}N_3O_5$ $[M+H]^+$: 518.2650, found: 518.2677. ^{1}H NMR (400 MHz, methanol-*d*$_4$) δ = 7.31 – 7.19 (m, 15H), 6.47 (d, *J*=6.8, 1H), 5.09 (d, *J*=3.6, 1H), 4.45 (dd, *J*=9.8, 0.8, 1H), 4.21 – 4.16 (m, 1H), 3.90 – 3.80 (m, 2H), 3.79 – 3.68 (m, 2H), 1.61 (s, 3H), 1.22 (d, *J*=6.2, 3H), 1.04 (d, *J*=6.1, 3H). ^{13}C NMR (101 MHz, methanol-*d*$_4$) δ = 172.2, 147.6, 136.7, 130.4, 128.6, 127.6, 97.0, 75.4, 74.1, 72.3, 71.3, 62.3, 55.2, 23.8, 22.6, 21.9.

Isopropyl 2-acetamido-3-chloro-2,3-dideoxy-α-D-allo/glucopyranoside (3a)

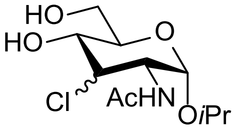

A 100 mL flask equipped with a magnetic stir bar was charged with trityl hydrazone **2a** (0.21 g, 0.39 mmol, 1.0 eq.) and anhydrous THF (3.9 mL). The resulting solution was evacuated and backfilled with nitrogen three times and then cooled to -20 °C (external temperature). *tert*-Butyl hypochlorite (1.24 M in anhydrous DCM, 0.35 mL, 1.1 eq.) was added dropwise to the cooled solution of hydrazone and stirred for 15 min. The resulting light-yellow solution was frozen in a liquid N_2 bath and degassed by two freeze-pump-thaw cycles, each time thawing in a -20 °C bath. After backfilling with N_2, the reaction was maintained at an external temperature ≤ -15 °C for 20 min. During this

time EtSH was degassed in a separated flask by a single freeze-pump-thaw cycle. Excess EtSH (2.3 mL, 0.31 mol, 80 eq.) was added to the cooled reaction. The reaction flask was subsequently transferred to a pre-heated 40 °C heating mantle and covered in aluminum foil. After 2.5 h, the reaction was cooled to ambient temperature and concentrated *in vacuo*. The product was purified by automated flash chromatography on a 15 g silica cartridge with DCM/MeOH (0 to 10% MeOH in DCM), which gave the title compound (87 mg, 0.31 mmol, 80%). The NMR analysis shows an equatorial to axial ratio of 1.4/1. **3a-equatorial:** HRMS (ESI pos) m/z calcd for $C_{11}H_{21}ClNO_5Na$ $[M+Na]^+$: 304.0922 and 306.0893, found: 304.0919 and 306.0888. ^1H NMR (400 MHz, methanol-d_4) δ = 4.89 (d, *J*=3.4, 1H), 4.09 (dd, *J*=11.6, 3.4, 1H), 4.02 (dd, *J*=11.7, 8.8, 1H), 3.91 (p, *J*=6.2, 1H), 3.83 – 3.66 (m, 3H), 3.54 (t, *J*=9.1, 1H), 1.99 (s, 3H), 1.23 (d, *J*=6.2, 3H), 1.14 (d, *J*=6.1, 3H). ^{13}C NMR (101 MHz, methanol-d_4) δ = 173.2, 96.6, 74.5, 72.5, 71.3, 64.3, 62.6, 56.2, 23.5, 22.3, 21.6. **3a-axial:** HRMS (ESI pos) m/z calcd for $C_{11}H_{21}ClNO_5Na$ $[M+Na]^+$: 304.0922, found: 304.0921. ^1H NMR (400 MHz, methanol-d_4) δ = 4.86 (overlaps with the H_2O peak in CD_3OD, 1H), 4.47 (t, *J*=3.8, 1H), 4.42 (t, *J*=4.2, 1H), 4.00 – 3.96 (m, 1H), 3.94 – 3.88 (m, 1H), 3.88 – 3.79 (m, 2H), 3.74 (dd, *J*=11.9, 5.2, 1H), 2.04 (s, 3H), 1.24 (d, *J*=6.3, 3H), 1.15 (d, *J*=6.1, 3H). ^{13}C NMR (101 MHz, methanol-d_4) δ = 173.0, 95.9, 71.2, 68.9, 67.8, 64.4, 62.4, 51.0, 23.8, 22.4, 21.5. Isopropyl 2-acetamido-3-chloro-2,3-dideoxy-α-D-allo/glucopyranoside (**3a-axial**) was isolated after column chromatography as a colorless syrup, which crystallized upon standing.

Synthesis of α-Glc derivatives

Methyl 3-keto-α-D-glucopyranoside (1b)

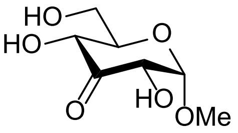

Methyl α-D-glucopyranoside (1.0 g, 5.2 mmol, 1.0 eq.) and benzoquinone (0.70 g, 6.4 mmol, 1.2 eq.) were dissolved in MeOH (12.9 ml). The catalyst $[(neocuproine)Pd(\mu\text{-}OAc)]_2(OTf)_2$ (27 mg, 0.5 mol%) was added and the mixture was stirred at room temperature for 1 h. The reaction mixture was concentrated *in vacuo* and subsequent purification by automated flash chromatography on a 24 g silica cartridge using pentane/EtOAc (0 to 100% EtOAc in pentane) afforded **1b** as a white solid (0.97 g, 5.0 mmol, 96%). ^1H NMR (400 MHz, methanol-d_4) δ = 5.05 (d, *J*=4.2, 1H), 4.40 (dd, *J*=4.3, 1.5, 1H), 4.23 (dd, *J*=9.7, 1.5, 1H), 3.88 (dd, *J*=12.1, 2.2, 1H), 3.80 (dd, *J*=12.1, 4.6, 1H), 3.65 (dddd, *J*=9.7, 4.7, 2.2, 0.6, 1H), 3.40 (s, 3H). ^{13}C NMR (101 MHz, methanol-d_4) δ = 207.0, 103.8, 76.7, 76.0, 73.3, 62.5, 55.7. Analysis was in agreement with literature[3].

Methyl-3-(trityl)hydrazone-α-D-glucopyranoside (2b)

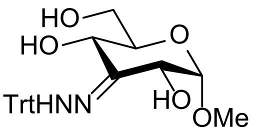

In a 100 mL flask, $TrtHNH_2\cdot HCl$ (2.4 g, 7.6 mmol, 1.5 eq.), $NaOAc\cdot 3H_2O$ (1.0 g, 7.6 mmol, 1.5 eq.), and **1b** (0.97 g 5.0 mmol, 1.0 eq.) were dissolved in DCM (39 mL), water (3.1 mL) and methanol (12.5 mL). The flask was subsequently evacuated and backfilled with nitrogen three times. Under N_2 flow, shielded from light, the reaction was stirred overnight, after which the reaction mixture was concentrated *in vacuo*. Purification by automated flash chromatography on an 80.0 g silica cartridge using pentane/EtOAc (0% to 90% EtOAc in pentane) gave the hydrazone as a mixture of *E/Z* isomers as yellow semi solid (1.9 g, 4.2 mmol, 84%). HRMS (ESI pos) m/z calcd for $C_{26}H_{29}N_2O_5$ $[M+H]^+$: 449.2071, found: 449.2072. Reported NMR data is for the major isomer. ^1H NMR (400 MHz, methanol-d_4) δ = 7.34 – 7.16 (m, 15H), 4.71 (d, *J*=3.7, 1H), 4.51 (d, *J*=3.7, 1H), 3.72 – 3.67 (m, 2H), 3.60 (dd, *J*=11.9, 5.5, 1H), 3.39 (s, 3H), 3.20 – 3.15 (m, 1H). ^{13}C NMR (101 MHz, methanol-d_4) δ = 147.1, 141.4, 130.3, 128.6, 127.6, 102.3, 76.7, 75.0, 71.1, 68.5, 62.7, 55.4.

Methyl 3-chloro-3-deoxy-α-D-allo/glucopyranoside (3b)

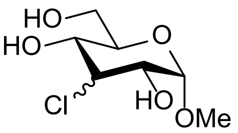

A 100 mL flask equipped with a magnetic stir bar was charged with trityl hydrazone **2b** (0.22 g, 0.50 mmol, 1.0 eq.) and anhydrous THF (5 mL). The resulting solution was evacuated and backfilled with nitrogen three times and then cooled to -20 °C (external temperature). *tert*-Butyl hypochlorite (1.24 M in anhydrous THF, 0.44 mL, 1.1 eq.) was added dropwise to the cooled solution of hydrazone and stirred for 15 min. The resulting light-yellow solution

was then frozen in a liquid N_2 bath and degassed by two freeze-pump-thaw cycles, each time thawing in a -20 °C bath. After backfilling with N_2, the reaction was maintained at an external temperature ≤ -15 °C for 20 min. During this time tBuSH was degassed in a separated flask by a single freeze-pump-thaw cycle. Excess *tert*-butylthiol (4.5 mL, 40 mmol, 80 eq.) was added to the cooled reaction. The reaction flask was subsequently transferred to a pre-heated 60 °C heating mantle and covered in aluminum foil. After 1 h, the reaction was allowed to cool to ambient temperature. Then the reaction mixture was concentrated *in vacuo* and the product was purified by automated flash chromatography on a 15 g silica cartridge using pentane/EtOAc (0 to 100% EtOAc in pentane). The title compound (71 mg, 0.33 mmol, 66%) was obtained as a colorless oil. The NMR analysis shows an equatorial to axial ratio of 1/3.5. **3b-equatorial:** HRMS (ESI neg) m/z calcd for $C_7H_{12}ClO_5$ $[M-H]^-$: 211.0379 and 211.0349, found: 211.0380 and 213.0350. ^{1}H NMR (400 MHz, methanol-*d*$_4$) δ = 4.70 (d, *J*=3.6, 1H), 3.94 (dd, *J*=10.4, 9.2, 1H), 3.81 (dd, *J*=11.9, 2.3, 1H), 3.71 (dd, *J*=11.8, 4.9, 1H), 3.59 – 3.54 (m, 1H), 3.54 – 3.51 (m, 1H), 3.50 – 3.45 (m, 1H). ^{13}C NMR (101 MHz, methanol-*d*$_4$) δ = 101.0, 74.1, 73.8, 72.1, 67.3, 62.5, 55.6. **3b-axial:** HRMS (ESI neg) m/z calcd for $C_7H_{12}ClO_5$ $[M-H]^-$: 211.0379 and 213.0349, found: 211.0370 and 213.0340. ^{1}H NMR (400 MHz, methanol-*d*$_4$) δ = 4.67 (d, *J*=4.2, 1H), 4.50 (t, *J*=3.5, 1H), 3.91 (t, *J*=4.1, 1H), 3.86 – 3.77 (m, 3H), 3.72 (dd, *J*=12.4, 5.4, 1H), 3.40 (s, 3H). ^{13}C NMR (101 MHz, methanol-*d*$_4$) δ = 100.7, 69.0, 68.9, 67.6, 66.7, 62.3, 56.0. Methyl 3-chloro-3-deoxy-α-D-allo/glucopyranoside (**3b-axial**) was crystallized from methanol.

Methyl 3-S-ethyl-3-deoxy-α-D-allo/glucopyranoside (4)

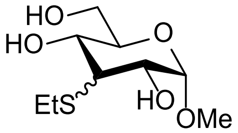

A 100 mL flask equipped with a magnetic stir bar was charged with trityl hydrazone **2b** (0.19 g, 0.40 mmol, 1.0 eq.) and anhydrous THF (4 mL). The resulting solution was evacuated and backfilled with nitrogen three times and then cooled to -20 °C (external temperature). *tert*-Butyl hypochlorite (1.24 M in anhydrous DCM, 0.35 mL, 1.1 eq.) was added dropwise to the cooled solution of hydrazone and stirred for 15 min. The resulting light-yellow solution was then frozen in a liquid N_2 bath and degassed by two freeze-pump-thaw cycles, each time thawing in a -20 °C bath. After backfilling with N_2, the reaction was maintained at an external temperature ≤ -15 °C for 20 min. During this time EtSH was degassed in a separated flask by a single freeze-pump-thaw cycle. Excess EtSH (2.3 mL, 32 mmol, 80 eq.) was added to the cooled reaction. The reaction flask was subsequently transferred to a pre-heated 40 °C heating mantle and the flask was covered in aluminum foil. After 2 h, the reaction was allowed to cool to ambient temperature. Then the reaction mixture was concentrated *in vacuo* and purification by automated flash chromatography on a 15 g silica cartridge using DCM/MeOH (0 to 10% MeOH in DCM) gave the title compound (71 mg, 0.30 mmol, 74%). The NMR analysis shows an equatorial to axial ratio of 1/2.6. Compound **4** (16 mg, 7.5 µmol, 19%) was also obtained with an equatorial and axial ratio of 1:1.4. HRMS (ESI neg) m/z calcd for $C_9H_{17}O_5S$ $[M-H]^-$: 237.0802, found: 237.0806. Reported NMR data is for the major compound (axial). ^{1}H NMR (400 MHz, methanol-*d*$_4$) δ = 4.57 (d, *J*=3.5, 1H), 3.92 – 3.86 (m, 1H), 3.86 – 3.70 (m, 2H), 3.69 – 3.60 (m, 1H), 3.38 (s, 3H), 3.35 – 3.29 (m, 1H), 2.71 – 2.58 (m, 1H), 2.60 – 2.47 (m, 1H), 1.26 (t, *J*=7.4, 3H). ^{13}C NMR (101 MHz, methanol-*d*$_4$) δ = 100.5, 70.3, 68.8, 68.3, 62.5, 57.2, 55.3, 30.7, 15.2.

Methyl 3-O-acetyl-α-D-glucopyranoside (S1)

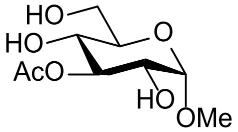

A 100 mL flask equipped with a magnetic stir bar was charged with trityl hydrazone **2b** (0.19 g, 0.40 mmol, 1.0 eq.) and anhydrous THF (4 mL). The resulting solution was evacuated and backfilled with nitrogen three times and then cooled to -20 °C (external temperature). *tert*-Butyl hypochlorite (1.24 M in anhydrous DCM, 0.35 mL, 1.1 eq.) was added dropwise to the cooled solution of hydrazone and stirred for 15 min. The resulting light-yellow solution was then frozen in a liquid N_2 bath and degassed by two freeze-pump-thaw cycles, each time thawing in a -20 °C bath. After backfilling with N_2, the reaction was maintained at an external temperature ≤ -15 °C for 20 min. During this time AcOH was degassed in a separated flask by a single freeze-pump-thaw cycle. Excess AcOH (1.8 mL, 32 mmol, 80 eq.) was added to the cooled reaction. The reaction flask was subsequently transferred to a pre-heated 60 °C heating mantle and the flask was covered in aluminum foil. After 1.5 h, the reaction was allowed to cool to ambient temperature. Then the reaction mixture was concentrated *in vacuo* and purification by automated flash chromatography on a 15 g silica cartridge using DCM/MeOH (0 to 10% MeOH in DCM) gave the title compound

S1 (30 mg, 0.13 mmol, 32%). HRMS (ESI neg) m/z calcd for $C_9H_{16}O_7$ $[M-H]^-$: 235.0823, found: 235.0820. ^1H NMR (400 MHz, methanol-d_4) δ = 5.15 (t, *J*=9.6, 1H), 4.71 (d, *J*=3.7, 1H), 3.81 (dd, *J*=11.9, 2.3, 1H), 3.70 (dd, *J*=11.9, 5.1, 1H), 3.63 – 3.57 (m, 1H), 3.56 – 3.51 (m, 1H), 3.48 – 3.45 (m, 1H), 3.43 (s, 3H), 2.10 (s, 3H). ^{13}C NMR (101 MHz, methanol-d_4) δ = 172.9, 101.1, 77.1, 73.4, 71.8, 69.7, 62.3, 55.6, 21.1.

Methyl 3-S-isopropyl-3-deoxy-α-D-allo/glucopyranoside (S2)

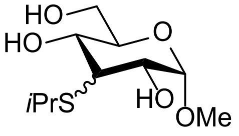

A 100 mL flask equipped with a magnetic stir bar was charged with trityl hydrazone **2b** (0.19 g, 0.40 mmol, 1.0 eq.) and anhydrous THF (4 mL). The resulting solution was evacuated and backfilled with nitrogen three times and then cooled to -20 °C (external temperature). *tert*-Butyl hypochlorite (1.24 M in anhydrous DCM, 0.35 mL, 1.1 eq.) was added dropwise to the cooled solution of hydrazone and stirred for 15 min. The resulting light-yellow solution was then frozen in a liquid N_2 bath and degassed by two freeze-pump-thaw cycles, each time thawing in a -20 °C bath. After backfilling with N_2, the reaction was maintained at an external temperature ≤ -15 °C for 20 min. During this time isopropyl mercaptan was degassed in a separated flask by a single freeze-pump-thaw cycle. Excess isopropyl mercaptan (3.0 mL, 32 mmol, 80 eq.) was added to the cooled reaction. The reaction flask was subsequently transferred to a pre-heated 60 °C heating mantle and the flask was covered in aluminum foil. After 2 h, the reaction was allowed to cool to ambient temperature. Then the reaction mixture was concentrated *in vacuo* and purification by automated flash chromatography on a 15 g silica cartridge using DCM/MeOH (0 to 10% MeOH in DCM) gave the title compound **S2** (35 mg, 0.14 mmol, 34%). The NMR analysis shows an equatorial to axial ratio of 1/2.2. Compound **3b** (35 mg, 0.17 mmol, 41%) was also obtained with an equatorial and axial ratio of 1:1.7. HRMS (ESI pos) m/z calcd for $C_{10}H_{20}O_5SNa$ $[M+Na]^+$: 275.0924, found: 275.0915. Reported NMR data is for the major compound (axial). ^1H NMR (400 MHz, methanol-d_4) δ = 4.53 (d, *J*=3.6, 1H), 3.86 (dd, *J*=5.3, 3.6, 1H), 3.80 (dd, *J*=6.1, 2.5, 1H), 3.76 – 3.67 (m, 2H), 3.59 – 3.55 (m, 1H), 3.38 – 3.33 (m, 4H), 2.91 – 2.77 (m, 1H), 1.31 (d, *J*=8.0, 3H), 1.28 (d, *J*=8.0, 3H). ^{13}C NMR (101 MHz, methanol-d_4) δ = 100.5, 70.4, 68.6, 67.9, 62.5, 55.6, 55.3, 39.0, 24.3, 23.6.

Methyl 3-S-acetyl-3-deoxy-α-D-allo/glucopyranoside (S3)

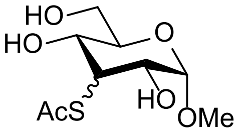

A 100 mL flask equipped with a magnetic stir bar was charged with trityl hydrazone **2b** (0.19 g, 0.40 mmol, 1.0 eq.) and anhydrous THF (4 mL). The resulting solution was evacuated and backfilled with nitrogen three times and then cooled to -20 °C (external temperature). *tert*-Butyl hypochlorite (1.24 M in anhydrous DCM, 0.35 mL, 1.1 eq.) was added dropwise to the cooled solution of hydrazone and stirred for 15 min. The resulting light-yellow solution was then frozen in a liquid N_2 bath and degassed by two freeze-pump-thaw cycles, each time thawing in a -20 °C bath. After backfilling with N_2, the reaction was maintained at an external temperature ≤ -15 °C for 20 min. During this time AcSH was degassed in a separated flask by a single freeze-pump-thaw cycle. Excess AcSH (2.3 mL, 32 mmol, 80 eq.) was added to the cooled reaction. The reaction flask was subsequently transferred to a pre-heated 60 °C heating mantle and the flask was covered in aluminum foil. After 2 h, the reaction was allowed to cool to ambient temperature. Then the reaction mixture was concentrated *in vacuo* and purification by automated flash chromatography on a 15 g silica cartridge using DCM/MeOH (0 to 10% MeOH in DCM) gave the title compound **S3** (17 mg, 6.7 µmol, 17%). The NMR analysis shows an equatorial to axial ratio of 9/1. Compound **3b** (4 mg, 1.9 µmol, 5%) was also obtained with an equatorial and axial ratio of 2.9:1. HRMS (ESI neg) m/z calcd for $C_9H_{15}O_6S$ $[M-H]^-$: 251.0595, found: 251.0597. Reported NMR data is for the major compound (equatorial). ^1H NMR (400 MHz, methanol-d_4) δ = 4.73 (d, *J*=3.4, 1H), 3.90 – 3.79 (m, 1H), 3.79 – 3.67 (m, 1H), 3.66 – 3.61 (m, 1H), 3.60 – 3.53 (m, 1H), 3.49 (s, 3H), 2.38 (s, 3H). ^{13}C NMR (101 MHz, methanol-d_4) δ = 197.2, 100.6, 74.5, 70.9, 69.1, 62.7, 55.5, 52.3, 30.7.

Methyl 3,3-dichloro-3-deoxy-α-D-glucopyranoside (5)

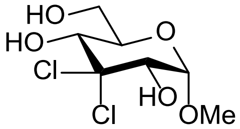

A 100 mL flask equipped with a magnetic stir bar was charged with trityl hydrazone **2b** (0.19 g, 0.40 mmol, 1.0 eq.) and dry MeOH (4 mL). The resulting solution was evacuated and backfilled with nitrogen three times and then cooled to -20 °C (external temperature). *tert*-Butyl hypochlorite (1.24 M in anhydrous DCM, 0.7 mL, 2.2 eq.) was added dropwise to the cooled solution of hydrazone and stirred for 15 min. The resulting light-yellow solution was then frozen in a liquid N_2 bath and degassed by two freeze-pump-thaw cycles, each time thawing in a -20 °C bath. After backfilling with N_2, the reaction was maintained at an external temperature ≤ -15 °C for 20 min. The reaction flask was subsequently transferred to a pre-heated 40 °C heating mantle and the flask was covered in aluminum foil. After 2 h, the reaction was allowed to cool to ambient temperature. Then the reaction mixture was concentrated *in vacuo* and purification by automated flash chromatography on a 15 g silica cartridge using DCM/MeOH (0 to 10% MeOH in DCM) gave the title compound **5** (34 mg, 0.14 mmol, 34%). Compound **1b** was also isolated in 27% yield (21 mg, 0.11 mmol). HRMS (ESI neg) m/z calcd for $C_7H_{11}Cl_2O_5$ $[M-H]^-$: 244.9989 and 246.9960, found: 244.9990 and 246.9960. ^{1}H NMR (400 MHz, methanol-d_4) δ = 4.73 (d, *J*=4.4, 1H), 4.02 (d, *J*=4.4, 1H), 3.89 (d, *J*=9.3, 1H), 3.86 – 3.70 (m, 4H), 3.39 (s, 3H). ^{13}C NMR (101 MHz, methanol-d_4) δ = 101.0, 96.9, 76.9, 75.7, 71.8, 62.2, 56.1.

Methyl-3-(2,4-dinitrophenyl)hydrazone-α-D-glucopyranoside (6a)

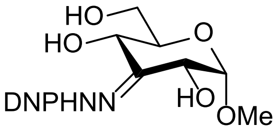

1b (1.0 g 5.2 mmol, 1.0 eq.) and dinitrophenyl hydrazine (contains 30% H_2O, 2.0 g, 6.7 mmol, 1.3 eq.) were dissolved in MeOH (13 mL). AcOH (0.06 mL, 1.0 mmol, 0.2 eq.) was added and the reaction mixture was stirred overnight at 50 °C while kept in the dark. TLC analysis showed full conversion and the reaction mixture was concentrated *in vacuo*. Purification by flash chromatography using heptane/EtOAc (0% to 100% EtOAc in heptane) gave the hydrazone as a mixture of *E/Z* isomers as an orange powder (1.56 g, 4.20 mmol, 81%). HRMS (ESI pos) m/z calcd for $C_{13}H_{17}N_4O_9$ $[M+H]^+$: 373.0990, found: 373.0997. Reported NMR data is for the major isomer. ^{1}H NMR (400 MHz, methanol-d_4) δ = 9.03 (dd, *J*=2.6, 0.8, 1H), 8.31 (dd, *J*=9.7, 2.6, 1H), 8.15 (d, *J*=9.7, 1H), 4.86 (1H, overlap with the peak of water), 4.74 (d, *J*=3.6, 1H), 4.18 (d, *J*=9.7, 1H), 3.90 (dd, *J*=12.0, 2.4, 1H), 3.86 – 3.81 (m, 2H), 3.60 (ddd, *J*=9.7, 4.7, 2.3, 1H), 3.44 (s, 3H). ^{13}C NMR (101 MHz, methanol-d_4) δ = 153.0, 146.4, 138.5, 130.4, 124.1, 116.8, 76.9, 75.6, 69.7, 62.5, 55.6.

Methyl-3-chloro-3-(2,4-dinitrophenyl)diazene-α-D-glucopyranoside (7a)

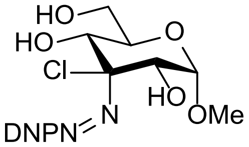

To hydrazone **S4** (0.19 g, 0.50 mmol, 1.0 eq.) was added dry dioxane (3 mL). The suspension was evacuated and backfilled with nitrogen three times. *tert*-Butyl hypochlorite (1.24 M in dry dioxane, 0.6 mL, 1.5 eq.) was added dropwise to suspension. After stirring overnight, the resulting solution was lyophilized to afford the title compound (0.19 g, 0.36 mmol, 72%). HRMS (ESI neg) m/z calcd for $C_{13}H_{14}ClN_4O_9$ $[M-H]^-$: 405.0455 and 407.0425, found: 405.0443 and 407.0411. ^{1}H NMR (400 MHz, acetonitrile-d_3) δ = 8.79 (d, *J*=2.3, 1H), 8.56 (dd, *J*=8.8, 2.4, 1H), 7.63 (d, *J*=8.8, 1H), 4.92 (dd, *J*=4.1, 0.6, 1H), 4.40 – 4.32 (m, 1H), 4.26 (d, *J*=4.1, 1H), 4.17 (d, *J*=9.8, 1H), 3.88 – 3.73 (m, 2H), 3.47 (s, 3H). ^{13}C NMR (101 MHz, acetonitrile-d_3) δ = 148.0, 129.7, 122.6, 121.0, 100.9, 100.0, 77.4, 76.1, 72.4, 62.4, 56.2.

Synthesis of Gal derivatives

Methyl a-D-xylohexopyranosid-4-ulose (1c)

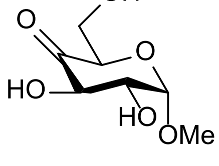

Methyl α-D-galactopyranoside (0.58 g, 3.0 mmol, 1.0 eq.) and dichlorodioctyltin[4] (25 mg, 60 µmol, 2 mol %) were dissolved in THF/MeOH (30 mL, 4/1 v/v). After 10 min, anhydrous K_2CO_3 (0.62 g, 4.5 mmol, 1.5 eq.) and Br_2 (0.23 mL, 4.5 mmol, 1.5 eq.) were added to the solution at room temperature. After stirring vigorously for 4 h, the reaction mixture was directly poured onto a column of silica gel. The column was flushed thoroughly with DCM to remove tin compounds and the excess of bromine until the red color disappeared, then the product was eluted with $CHCl_3$/MeOH (85/15 v/v) to give **1c** (0.47 g, 2.4 mmol, 80%). ^{1}H NMR (400 MHz, DMSO-d_6) δ = 5.40 (d, *J*=6.6,

1H, OH), 5.34 (d, *J*=5.6, 1H, OH), 4.78 (d, *J*=3.5, 1H), 4.67 (t, *J*=5.9, 1H, OH), 4.14 (dd, *J*=10.1, 5.7, 1H), 4.06 (dd, *J*=6.2, 3.6, 1H), 3.72 (ddd, *J*=11.8, 5.9, 3.6, 1H), 3.57 – 3.50 (m, 2H), 3.39 (s, 3H). ^{13}C NMR (101 MHz, DMSO-d_6) δ = 204.5, 99.5, 75.7, 74.4, 73.3, 59.0, 55.2, 39.5. Analysis was in agreement with literature.[4]

Methyl 4-(trityl)hydrazone-a-D-xylohexopyranosid-4-ulose (2c)

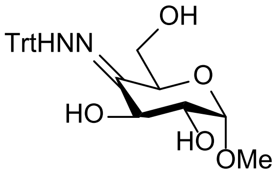

A 100 mL flask equipped with magnetic stir bar was charged with $TrtNHNH_2·HCl$ (2.7 g, 8.7 mmol, 2.7 eq.), $NaOAc·3H_2O$ (0.70 g, 8.5 mmol, 2.6 eq.), **1c** (0.63 g, 3.3 mmol, 1.0 eq.). DCM (24 mL), water (0.5 mL) and methanol (14 ml) were added. The flask was subsequently evacuated and backfilled with nitrogen three times. Under N_2 flow, shielded from light, the reaction was stirred at ambient temperature overnight. Then the solvent was evaporated and purification by automated flash chromatography on an 80 g silica cartridge using DCM/MeOH (0% to 4% MeOH in DCM) provided the product as a yellow semi-solid (1.4 g, 3.1 mmol, 95%). HRMS (ESI pos) m/z calcd for $C_{26}H_{28}N_2O_5Na$ $[M+Na]^+$: 471.1890, found: 471.1892. ^1H NMR (400 MHz, methanol-d_4) δ = 7.31 – 7.18 (m, 15H), 4.71 (d, *J*=3.5, 1H), 4.59 (d, *J*=9.2, 1H), 4.04 (t, *J*=5.2, 1H), 3.58 (dd, *J*=9.2, 3.5, 1H), 3.46 (dd, *J*=11.7, 4.6, 1H), 3.40 (s, 3H), 3.36 (dd, *J*=11.7, 5.7, 1H). ^{13}C NMR (101 MHz, methanol-d_4) δ = 147.3, 143.3, 130.2, 128.7, 127.7, 101.1, 75.1, 73.9, 73.7, 72.1, 64.4, 55.9.

Methyl 4-chloro-4-deoxy-α-D-galacto/glucopyranoside (3c)

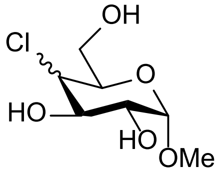

In a 100 mL flask equipped with a magnetic stir bar, **2c** (0.31 g, 0.69 mmol, 1.0 eq.) was dissolved in anhydrous THF (6.9 mL) and the resulting light-yellow solution was evacuated and backfilled with nitrogen gas three times. After cooling to -20 °C (external temperature), *tert*-butyl hypochlorite (1.24 M in anhydrous THF, 0.61 mL, 1.1 eq.) was added dropwise to the cooled solution of hydrazone and stirred for 15 min. The solution was then frozen in a liquid N_2 bath and degassed by two freeze-pump-thaw cycles, each time thawing in a -20 °C bath. After backfilling with N_2, the reaction was maintained at an external temperature ≤ -15 °C for 20 min. During this time *tert*-butylthiol was degassed once by freeze-pump-thaw, followed by the addition of *tert*-butylthiol (6.2 mL, 55.2 mmol, 80 eq.) to the cooled reaction. The reaction flask was subsequently transferred to a pre-heated 60°C heating mantle and the flask was covered in aluminum foil. After 1 h, the reaction was allowed to cool to ambient temperature and concentrated *in vacuo*. Purification by automated flash chromatography on a 25 g silica cartridge using DCM/MeOH (0% to 6% MeOH in DCM) provided the product as a colorless oil (95 mg, yield: 65%). The equatorial and axial product could not be separated and NMR analysis shows an equatorial to axial ratio of 1/2.6. HRMS (ESI pos) m/z calcd for $C_7H_{13}ClO_5Na$ $[M+Na]^+$: 235.0344 and 237.0314, found: 235.0345 and 237.0312. Reported NMR data is for the major isomer (axial). ^1H NMR (400 MHz, methanol-d_4) δ = 4.72 (d, *J*=3.8, 1H), 4.39 (dd, *J*=3.7, 1.3, 1H), 4.03 – 3.96 (m, 2H), 3.83 – 3.77 (m, 1H), 3.68 (dd, *J*=6.3, 3.0, 2H), 3.42 (s, 3H). ^{13}C NMR (101 MHz, methanol-d_4) δ = 101.5, 71.1, 70.3, 70.0, 64.9, 63.0, 55.8.

Synthesis of Xyl derivatives

Methyl 3-keto-α-D-xylopyranoside (1d)

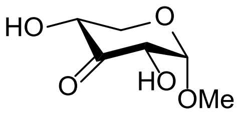

Methyl α-D-xylopyranoside (0.74 g, 4.5 mmol, 1.0 eq.) and benzoquinone (0.54 g, 5.0 mmol, 1.1 eq.) were dissolved in MeOH (11 mL). The catalyst $[(neocuproine)Pd(\mu\text{-}OAc)]_2(OTf)_2$ (24 mg, 0.5 mol %) was added and the mixture was stirred at room temperature for 1 h after which TLC analysis showed complete conversion of the starting material whereupon the solvent was evaporated. Subsequent purification by automated flash chromatography on a 40 g silica cartridge with pentane/EtOAc (0 to 100% EtOAc) afforded the title compound as a colorless oil (0.52 g, 3.2 mmol, 71%). HRMS (ESI neg) m/z calcd for $C_6H_9O_5$ $[M-H]^-$: 161.0456, found: 161.0458. ^1H NMR (400 MHz, methanol-d_4) δ = 5.01 (d, *J* = 4.4 Hz, 1H), 4.42 – 4.32 (m, 2H), 4.01 (dd, *J* = 10.3, 7.9 Hz, 1H), 3.58 (dd, *J* = 10.7, 10.1 Hz, 1H), 3.39 (s, 3H).^{13}C NMR (101 MHz, methanol-d_4) δ = 206.6, 104.4, 76.3, 73.1, 65.2, 55.7. Methyl 3-keto-α-D-xylopyranoside (**1d**) was isolated after column chromatography as a colorless syrup, which crystallized upon standing.

Methyl 3-(trityl)hydrazone-α-D-xylopyranoside (2d)

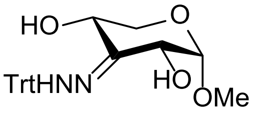

A 100 mL flask equipped with magnetic stir bar was charged with TrtNHNH$_2$·HCl (1.5 g, 4.7 mmol, 1.5 eq.), NaOAc·3H$_2$O (0.65 g, 4.7 mmol, 1.5 eq.), **1d** (0.51 g, 3.2 mmol, 1.0 eq.). DCM (24 mL), water (1.9 mL) and methanol (7.8 mL) were added. The flask was subsequently evacuated and backfilled with nitrogen three times. Under N$_2$ flow, shielded from light, the reaction was stirred at room temperature overnight. Then, the solvent was evaporated and purification by automated flash chromatography on a 40 g silica cartridge with pentane/EtOAc (0% to 50% EtOAc) provided **2d** as a mixture of *E/Z* isomers (0.93 g, 2.2 mmol, 69%) as a yellow oil. HRMS (ESI pos) m/z calcd for $C_{25}H_{26}N_2O_4Na$ [M+Na]$^+$: 441.1785, found: 441.1777. ***Z*** isomer: ^{1}H NMR (400 MHz, methanol-*d*$_4$) δ = 7.34 – 7.18 (m, 15H), 4.64 (d, *J* = 3.7 Hz, 1H), 4.49 (d, *J* = 3.7 Hz, 1H), 3.88 (dd, *J* = 10.2, 6.0 Hz, 1H), 3.62 (dd, *J* = 10.1, 5.9 Hz, 1H), 3.37 (s, 3H), 3.11 (t, *J* = 10.1 Hz, 1H). ^{13}C NMR (101 MHz, methanol-*d*$_4$) δ 147.1, 142.0, 130.3, 128.6, 127.6, 102.7, 74.8, 74.1, 68.5, 65.8, 55.5. ***E*** isomer: ^{1}H NMR (400 MHz, methanol-*d*$_4$) δ = 7.33 – 7.18 (m, 15H), 4.58 (d, *J* = 3.5 Hz, 1H), 4.52 (t, *J* = 8.1 Hz, 1H), 3.89 (d, *J* = 3.5 Hz, 1H), 3.59 (d, *J* = 8.1 Hz, 2H), 3.31 (s, 3H).^{13}C NMR (101 MHz, methanol-*d*$_4$) δ = 147.1, 142.0, 130.3, 128.6, 127.6, 101.6, 74.0, 71.2, 70.9, 65.1, 55.4. Methyl 3-(trityl)hydrazone-α-D-xylopyranoside (**2d**) was isolated after column chromatography as a colorless syrup, the E isomer formed crystal immediately after evaporation.

Methyl 3-chloro-3-deoxy-α-D-ribose/xylopyranoside (3d)

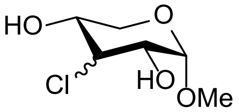

A 100 mL flask equipped with a magnetic stir bar was charged with trityl hydrazone **2d** (0.18 g, 0.40 mmol, 1.0 eq.) and anhydrous THF (4 mL). The resulting yellow solution was evacuated and backfilled with nitrogen three times and then cooled to -20 °C (external temperature), *tert*-Butyl hypochlorite (1.24 M in anhydrous DCM, 0.35 mL, 1.1 eq.) was added dropwise to the cooled solution of hydrazone and stirred for 15 min. The resulting light-yellow solution was then frozen in a liquid N$_2$ bath and degassed by two freeze-pump-thaw cycles, each time thawing in a -20 °C bath. After backfilling with N$_2$, the reaction was maintained at an external temperature ≤ -15 °C for 30 min. During this time *tert*-butylthiol was degassed in a separated flask by purging with N$_2$ for 5 min, after which 3.6 mL (32 mmol, 80 eq.) was added to the cooled reaction. The reaction flask was subsequently transferred to a pre-heated 60 °C heating mantle and the flask was covered in aluminum foil. After 2 h, the reaction mixture was allowed to cool to room temperature and concentrated *in vacuo*. Purification by automated flash chromatography on a 15 g silica cartridge using heptane/EtOAc (0 to 100% EtOAc) gave the products as a yellow oil (53 mg, 0.29 mmol, 73%). The equatorial- and axial-products were isolated separately with a ratio of 1/3.4. **3d-equatorial**: HRMS (ESI neg) m/z calcd for $C_6H_{11}Cl_2O_4$ [M+Cl]$^-$: 217.0029 and 218.9999, found: 217.0043 and 219.0020. ^{1}H NMR (400 MHz, methanol-*d*$_4$) δ = 4.63 (d, *J*=3.6, 1H), 3.86 (dd, *J*=10.3, 8.9, 1H), 3.67 – 3.58 (m, 2H), 3.54 (dd, *J*=10.3, 3.6, 1H), 3.49 – 3.43 (m, 1H), 3.40 (s, 3H). ^{13}C NMR (101 MHz, methanol-*d*$_4$) δ = 101.1, 73.8, 72.1, 67.0, 63.4, 55.6. **3d-axial**: HRMS (ESI neg) m/z calcd for $C_6H_{10}ClO_4$ [M-H]$^-$: 181.0273 and 183.0244, found: 181.0277 and 183.0245. ^{1}H NMR (400 MHz, methanol-*d*$_4$) δ = 4.44 (d, *J*=2.4, 1H), 4.33 (t, *J*=3.2, 1H), 3.96 – 3.89 (m, 1H), 3.89 – 3.83 (m, 2H), 3.53 – 3.47 (m, 1H), 3.46 (s, 3H). ^{13}C NMR (101 MHz, methanol-*d*$_4$) δ = 102.0, 71.3, 69.1, 64.7, 62.9, 56.7.

Synthesis of cellobiose derivatives

2,3,6,2',3',4',6'-Hepta-*O*-acetyl-cellobiose (S4)

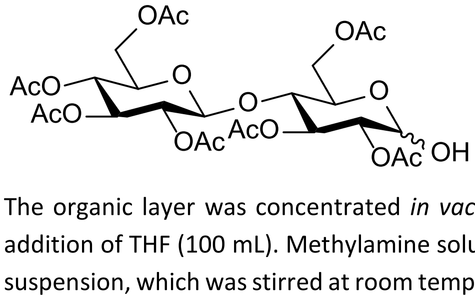

To a stirred suspension of D-(+)-cellobiose (5.0 g, 14.6 mmol) in acetic anhydride (40 mL) at 0°C was added HClO$_4$ (3 drops, 70% aq.) dropwise, after which the reaction was stirred at room temperature for 1 h. The reaction mixture was diluted with EtOAc and washed subsequently with sat. aq. NaHCO$_3$ (3×) and brine (1×). The organic layer was concentrated *in vacuo* and the crude was co-evaporated with toluene (1×) before the addition of THF (100 mL). Methylamine solution (40% in water, 2.6 mL, 29 mmol, 2 eq.) was added to the white suspension, which was stirred at room temperature overnight. TLC analysis showed still starting material present.

Therefore, another 2.6 mL of methylamine solution (40% in water) was added and after 1 h TLC analysis showed complete conversion. The reaction mixture was concentrated *in vacuo* and purified by flash column chromatography using pentane/EtOAc (0 to 60% EtOAc in pentane) affording the product as an alpha-beta mixture as a white solid (6.3 g, 9.9 mmol, 68%). HRMS (ESI pos) m/z calcd for $C_{26}H_{36}O_{18}Na$ [M+Na]$^+$: 659.1794, found: 659.1768. Reported NMR data is for the major isomer. ^{1}H NMR (400 MHz, chloroform-*d*) δ = 5.49 (t, *J*=10.2, 9.3, 1H), 5.35 (d, *J*=3.6, 1H), 5.14 – 5.11 (m, 1H), 5.09 – 5.05 (m, 1H), 4.92 (dd, *J*=9.1, 8.0, 1H), 4.82 (dd, *J*=10.2, 3.6, 1H), 4.54 – 4.49 (m, 2H), 4.38 – 4.33 (m, 1H), 4.18 – 4.14 (m, 1H), 4.10 (dd, *J*=11.9, 4.5, 1H), 4.07 – 4.01 (m, 1H), 3.75 (m, 1H), 3.68 – 3.64 (m, 1H), 2.12 (s, 3H), 2.08 (s, 3H), 2.06 (s, 3H), 2.02 (s, 6H), 2.00 (s, 3H), 1.97 (s, 3H). ^{13}C NMR (101 MHz, chloroform-*d*) δ = 171.0, 170.7, 170.6, 170.6, 170.6, 170.4, 170.4, 169.8, 169.8, 169.4, 169.2, 169.2, 100.8, 95.4, 90.2, 76.6, 73.1, 72.0, 71.8, 71.4, 69.4, 68.4, 68.0, 61.9, 61.7, 21.0, 20.9, 20.8, 20.8, 20.7, 20.7. Analysis was in agreement with literature[5].

4-*tert*-butylbenzyl-β-D-heptaacetyl-cellobioside (S5)

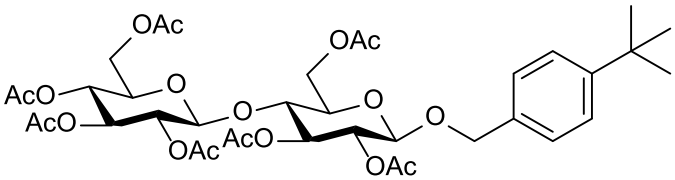

To an anomeric mixture of **S4** (5.4 g, 8.6 mmol, 1.0 eq.) suspended in acetonitrile (38 mL) in the dark, was added silver oxide (6.1 g, 27 mmol, 3.0 eq.) while stirring. After 15 min, 4-*tert*-butylbenzyl bromide (4.9 mL, 26 mmol. 3.0 eq.) was added and the mixture left to stir for six days. The reaction mixture was diluted with dichloromethane, filtered through a plug of celite and concentrated *in vacuo.* After two purifications by automated flash chromatography on a 25 g silica cartridge using DCM/EtOAc (0% to 50% (1st column) and 0% to 20% (2nd column) EtOAc in DCM) the title compound was obtained as a white solid (5.5 g, 7.0 mmol, 81%) HRMS (ESI pos) m/z calcd for $C_{37}H_{50}O_{18}Na$ [M+Na]$^+$: 805.2889, found: 805.2889. ^{1}H NMR (400 MHz, chloroform-*d*) δ = 7.35 (d, *J*=8.3, 2H), 7.19 (d, *J*=8.3, 2H), 5.13 (m, 2H), 5.05 (t, *J*=9.6, 1H), 4.98 – 4.89 (m, 2H), 4.82 (d, *J*=12.1, 1H), 4.58 – 4.52 (m, 2H), 4.50 (m, 2H), 4.36 (dd, *J*=12.5, 4.4, 1H), 4.10 (dd, *J*=12.0, 5.0, 1H), 4.03 (dd, *J*=12.4, 2.3, 1H), 3.78 (t, *J*=9.5, 1H), 3.65 (ddd, *J*=9.9, 4.5, 2.3, 1H), 3.56 (ddd, *J*=9.9, 4.9, 2.1, 1H), 2.14 (s, 3H), 2.07 (s, 3H), 2.03 (s, 3H), 2.00 (s, 6H), 1.99 (s, 3H), 1.97 (s, 3H), 1.31 (s, 9H). ^{13}C NMR (101 MHz, chloroform-*d*) δ = 170.6, 170.5, 170.3, 170.0, 169.7, 169.4, 169.2, 151.2, 133.6, 127.8, 125.5, 100.9, 99.0, 76.6, 73.1, 72.8, 72.7, 72.1, 71.8, 71.7, 70.6, 67.9, 62.0, 61.7, 34.7, 31.5, 21.0, 20.8, 20.8, 20.7, 20.7.

4-*tert*-butylbenzyl-β-D-cellobioside (S6)

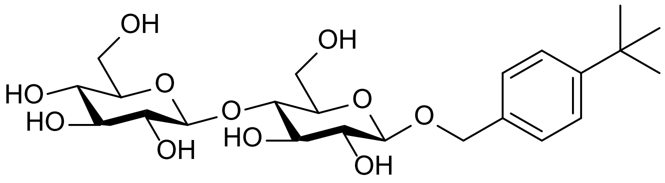

To a suspension of **S5** (5.0 g, 6.3 mmol.) in MeOH (64 mL) was added sodium (19 mg) and the resulting mixture was allowed to stir overnight. Amberlite 120 H$^+$ was added to quench the reaction and the mixture was filtered. The product was obtained as a white solid (3.1 g, 6.3 mmol, 99%). HRMS (ESI pos) m/z calcd for $C_{23}H_{36}O_{11}Na$ [M+Na]$^+$: 511.2150, found: 511.2143. ^{1}H NMR (400 MHz, methanol-*d*$_4$) δ = 7.41 – 7.40 (m, 2H), 7.37 – 7.35 (m, 2H), 4.91 (d, *J*=11.7, 1H), 4.67 (d, *J*=11.6, 1H), 4.45 (d, *J*=7.9, 1H), 4.41 (d, *J*=7.8, 1H), 3.99 – 3.88 (m, 3H), 3.69 (dd, *J*=11.9, 5.3, 1H), 3.65 – 3.59 (m, 1H), 3.54 (t, *J*=8.9, 1H), 3.45 – 3.31 (m, 6H), 3.26 (dd, *J*=8.9, 7.9, 1H), 1.34 (s, 9H). ^{13}C NMR (101 MHz, methanol-*d*$_4$) δ = 151.8, 135.9, 129.1, 126.1, 104.6, 102.9, 80.7, 78.1, 77.8, 76.5, 76.4, 74.9, 74.9, 71.6, 71.4, 62.4, 61.9, 35.4, 31.8.

4-tert-butylbenzyl-β-3-ketocellobioside (1e)

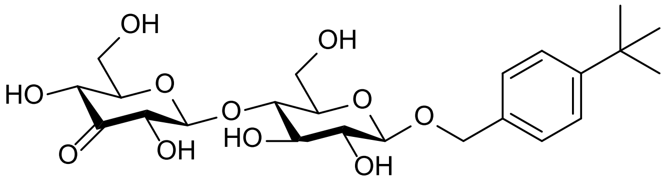

S6 (1.9 g, 3.9 mmol, 1.0 eq.) was dissolved in a dioxane/DMSO mixture (4/1 v/v, 13 mL). Benzoquinone (0.52 g, 4.8 mmol, 1.1 eq.) and [(neocuproine)Pd(μ-OAc)]$_2$(OTf)$_2$ (61 mg, 1.5 mol%) were added and the reaction mixture was stirred overnight, after which an additional 61 mg of [(neocuproine)Pd(μ-OAc)]$_2$(OTf)$_2$ was added and the mixture

stirred overnight. Water (150 mL) was added and the mixture was lyophilized to afford the crude product. Subsequent purification by automated flash chromatography on a 40 g silica cartridge using DCM/MeOH (0% to 7% MeOH in DCM) afforded the product as a brown oil (1.1 g, 2.3 mmol, 59%). HRMS (ESI pos) m/z calcd for $C_{23}H_{34}O_{11}Na$ [M+Na]$^+$: 509.1993, found: 509.1985. ^{1}H NMR (400 MHz, methanol-*d*$_4$) δ = 7.38 (d, *J*=8.5, 2H), 7.34 (d, *J*=8.4, 2H), 4.89 – 4.87 (overlap with HDO peak, 1H), 4.64 (d, *J*=11.7, 1H), 4.56 (d, *J*=7.9, 1H), 4.39 (d, *J*=7.9, 1H), 4.25 (dd, *J*=10.2, 1.7, 1H), 4.19 (dd, *J*=8.0, 1.7, 1H), 3.97 – 3.84 (m, 3H), 3.82 – 3.75 (m, 1H), 3.68 (t, *J*=9.3, 1H), 3.56 (t, *J*=9.0, 1H), 3.42 – 3.36 (m, 2H), 3.32-3.31 (m, 1H), 1.31 (s, 9H). ^{13}C NMR (101 MHz, methanol-*d*$_4$) δ = 206.7, 151.8, 135.9, 129.1, 126.2, 105.7, 103.0, 80.3, 78.2, 78.2, 76.5, 76.4, 74.9, 73.4, 71.6, 62.3, 61.5, 35.4, 31.8.

4-*tert*-butylbenzyl-3-(trityl)hydrazone-β-D-cellobioside (2e)

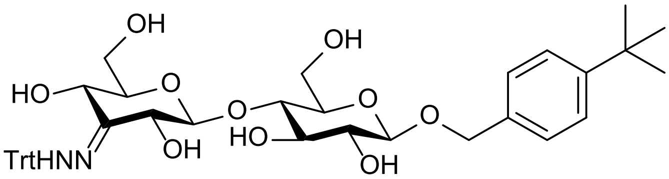

In a 100 mL flask TrtNHNH$_2$·HCl (0.76 g, 2.4 mmol, 1.1 eq.), NaOAc·3H$_2$O (0.33 g, 2.4 mmol, 1.1 eq.), and **1e** (1.1 g 2.2 mmol, 1.0 eq.) were dissolved in a mixture of MeOH (5.7 mL), DCM (16.8 mL), and water (1 mL). The flask was subsequently evacuated and backfilled with nitrogen three times. Under N$_2$ flow, shielded from light, the reaction was stirred at ambient temperature overnight, after which the reaction mixture was concentrated *in vacuo*. Purification by automated flash chromatography on a 40 g silica cartridge using pentane/EtOAc (0% to 100% EtOAc in pentane) and DCM/MeOH (0% to 4% MeOH in DCM) gave the title compound as a yellow semi-solid (1.1 g, 1.5 mmol, 68%). The product was obtained as a mixture of *E/Z* isomers. HRMS (ESI pos) m/z calcd for $C_{42}H_{51}N_2O_{10}$ [M+H]$^+$: 743.3538, found: 743.3554. ***Z*-isomer:** ^{1}H NMR (400 MHz, methanol-*d*$_4$) δ = 7.37 – 7.15 (m, 19H), 4.85 (d, *J*=12.0, 1H), 4.60 (d, *J*=11.6, 1H), 4.32 (d, *J*=7.8, 1H), 4.27 (d, *J*=7.7, 1H), 4.23 (d, *J*=7.7, 1H), 3.87 (dd, *J*=12.1, 2.4, 1H), 3.79 (dd, *J*=12.1, 4.4, 1H), 3.73 – 3.66 (m, 2H), 3.57 – 3.50 (m, 2H), 3.47 – 3.42 (m, 1H), 3.36 – 3.31 (m, 1H), 3.28 (1H, overlap with the peak of CD_3OD), 2.79 – 2.75 (m, 1H), 1.28 (s, 9H). ^{13}C NMR (101 MHz, methanol-*d*$_4$) δ = 151.8, 146.8, 142.2, 135.9, 130.1, 129.2, 128.8, 127.7, 126.2, 105.1, 102.8, 80.8, 79.9, 76.5, 76.3, 75.9, 74.8, 74.2, 71.5, 68.6, 62.5, 61.9, 35.4, 31.8. ***E*-isomer:** ^{1}H NMR (400 MHz, methanol-*d*$_4$) δ = 7.38 – 7.16 (m, 19H), 4.84 (d, *J*=11.4, 1H), 4.60 (d, *J*=11.7, 1H), 4.44 (d, *J*=9.8, 1H), 4.32 (d, *J*=7.8, 1H), 3.97 (d, *J*=7.0, 1H), 3.82 (dd, *J*=12.1, 2.3, 1H), 3.74 (dd, *J*=11.9, 2.3, 1H), 3.70 – 3.59 (m, 3H), 3.45 – 3.40 (m, 2H), 3.28 – 3.22 (m, 3H), 1.28 (s, 9H). ^{13}C NMR (101 MHz, methanol-*d*$_4$) δ = 151.8, 146.9, 142.4, 135.9, 130.1, 129.2, 128.8, 127.7, 126.2, 106.1, 102.9, 80.8, 79.4, 76.6, 76.3, 74.8, 74.2, 72.9, 71.5, 70.7, 61.9, 61.7, 35.4, 31.8.

4-*tert*-butylbenzyl-3-chloro-3-deoxy-β-D-cellobioside (3e)

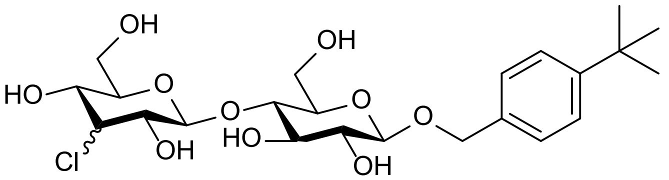

In a 100 mL flask equipped with a magnetic stir bar, **2e** (0.37 g, 0.50 mmol, 1.0 eq.) was dissolved in anhydrous THF (5.0 mL) and the resulting light-yellow solution was evacuated and backfilled with nitrogen three times. After cooling to -20 °C (external temperature), *tert*-butyl hypochlorite (1.24 M in anhydrous THF, 0.44 mL, 1.1 eq.) was added dropwise to the cooled solution of hydrazone and stirred for 15 min. The solution was then frozen in a liquid N$_2$ bath and degassed by two freeze-pump-thaw cycles, each time thawing in a -20 °C bath. After backfilling with N$_2$, the reaction was maintained at an external temperature ≤ -15 °C for 20 min. During this time *tert*-butylthiol was degassed once by freeze-pump-thaw, followed by the addition of *tert*-butylthiol (4.5 mL, 40 mmol, 80 eq.) to the cooled reaction. The reaction flask was subsequently transferred to a pre-heated 60°C heating mantle and the flask was covered in aluminum foil. After 1 h, the reaction was allowed to cool to ambient temperature and concentrated *in vacuo*. Purification by automated flash chromatography on a 25 g silica cartridge using DCM/MeOH (0% to 5% MeOH in DCM) provided the product as a colorless oil (0.18 g, 0.36 mmol, 71%). The ratio between the equatorial and axial product is 1/2.1. **3e-equatorial:** HRMS (ESI pos) m/z calcd for $C_{23}H_{35}ClO_{10}Na$ [M+Na]$^+$: 529.1811 and 531.1782, found: 529.1803 and 531.1771. ^{1}H NMR (400 MHz, methanol-*d*$_4$) δ = 7.35 (d, *J*=8.4, 2H), 7.31 (d, *J*=8.4, 2H), 4.85 (d, *J*=11.4, 1H), 4.61 (d, *J*=11.6, 1H), 4.43 (d, *J*=7.7, 1H), 4.34 (d, *J*=7.8, 1H), 3.94 – 3.82 (m, 3H), 3.70 – 3.63 (m,

2H), 3.58 (t, *J*=9.2, 1H), 3.50 – 3.43 (m, 2H), 3.38 – 3.31 (m, 3H), 3.28 (1H, overlap with the peak of CD_3OD), 1.28 (s, 9H). ^{13}C NMR (101 MHz, methanol-*d*$_4$) δ = 151.8, 135.9, 129.1, 126.2, 104.7, 102.9, 80.5, 79.1, 76.5, 76.4, 75.4, 74.9, 71.8, 71.6, 68.7, 62.3, 61.7, 35.4, 31.8. **3e-axial:** HRMS (ESI pos) m/z calcd for $C_{23}H_{35}ClO_{10}Na$ $[M+Na]^+$: 529.1811 and 531.1782, found: 529.1801 and 531.1768. ^{1}H NMR (400 MHz, methanol-*d*$_4$) δ = 7.35 (d, *J*=8.4, 2H), 7.31 (d, *J*=8.5, 2H), 4.84 (s, 1H), 4.79 (d, *J*=7.8, 1H), 4.61 (d, *J*=11.6, 1H), 4.58 – 4.56 (m, 1H), 4.34 (d, *J*=7.8, 1H), 3.92 – 3.80 (m, 5H), 3.68 – 3.62 (m, 2H), 3.56 (t, *J*=9.1, 1H), 3.47 (t, *J*=8.9, 1H), 3.37 – 3.33 (m, 1H), 3.28 (1H, overlap with the peak of CD_3OD), 1.28 (s, 9H). ^{13}C NMR (101 MHz, methanol-*d*$_4$) δ = 151.8, 135.9, 129.2, 126.2, 102.9, 101.9, 80.5, 76.5, 76.4, 75.9, 74.8, 71.5, 70.8, 69.0, 67.6, 62.2, 61.8, 35.4, 31.8.

Synthesis of maltose derivatives

2,3,6,2',3',4',6'-Hepta-*O*-acetyl-maltose (S7)

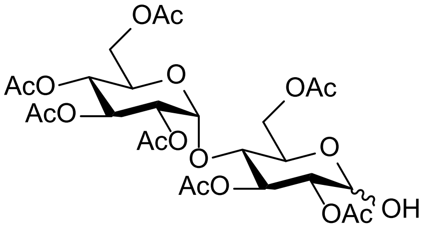

To a stirred suspension of D-maltose (20 g, 58 mmol) in acetic anhydride (0.16 L) at 0 °C was added $HClO_4$ (12 drops, 70% aq.) dropwise. The mixture was stirred at room temperature for 1 h, after which the solution was diluted with EtOAc. The solution was washed with sat. aq. $NaHCO_3$ (3×) and brine (1×). The organic layer was dried over Na_2SO_4 and concentrated *in vacuo* to afford the crude per-acetylated product, which was dissolved in THF (0.4 L) and treated with an aqueous methylamine solution (40% in water, 10.5 mL, 0.12 mol, 2 eq.). After stirring at room temperature for 3 h, TLC showed full conversion. The reaction mixture was concentrated *in vacuo* and purification by flash column chromatography using pentane/EtOAc (20% to 70% EtOAc in pentane) gave the product as an alpha-beta mixture as a white solid (23 g, 36 mmol, 61%). HRMS (ESI pos) m/z calcd for $C_{26}H_{36}O_{18}Na$ $[M+Na]^+$: 659.1794, found: 659.1773. Reported NMR data is for the major isomer. ^{1}H NMR (400 MHz, chloroform-*d*) δ = 5.57 (dd, *J*=10.1, 8.9, 1H), 5.43 (d, *J*=4.0, 1H), 5.36 – 5.33 (m, 2H), 5.06 (t, *J*=9.9, 1H), 4.85 (dd, *J*=10.5, 4.0, 1H), 4.76 (dd, *J*=10.1, 3.5, 1H), 4.52 – 4.46 (m, 1H), 4.27 – 4.19 (m, 4H), 4.04 (dd, *J*=12.5, 2.4, 1H), 3.99 – 3.94 (m, 2H), 2.14 (s, 3H), 2.09 (s, 3H), 2.05 (s, 3H), 2.04 (s, 3H), 2.01 (s, 3H), 2.00 (s, 3H), 1.99 (s, 3H). ^{13}C NMR (101 MHz, chloroform-*d*) δ = 170.82, 170.78, 170.7, 170.4, 170.1, 170.1, 169.6, 95.6, 90.1, 72.7, 72.4, 71.7, 70.1, 69.5, 68.5, 68.1, 67.9, 62.9, 61.5, 21.1, 21.0, 20.82, 20.81, 20.74, 20.72. Analysis was in agreement with literature[5].

4-*tert*-butylbenzyl-β-D-heptaacetyl-maltoside (S8)

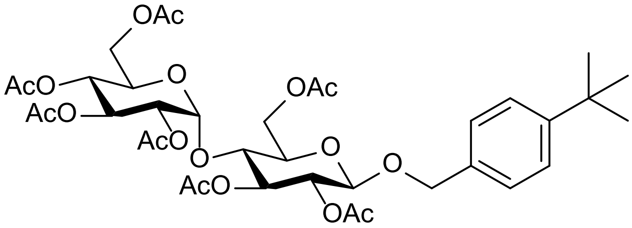

To an anomeric mixture of **S7** (4.5 g, 7.1 mmol, 1.0 eq.) suspended in acetonitrile (31 mL) in the dark, was added silver oxide (4.8 g, 22 mmol, 3.0 eq.) while stirring. After 15 min, 4-*tert* butylbenzyl bromide (3.9 mL, 22 mmol, 3.0 eq.) was added and the mixture was left to stir for 72 h. The reaction mixture was diluted with DCM, filtered through a plug of celite, and concentrated *in vacuo*. Purification by automated flash chromatography on a 120 g silica cartridge using pentane/EtOAc (0% to 40% EtOAc in pentane) yielded the title compound as a white fluffy solid (4.1 g, 5.2 mmol, 73%). HRMS (ESI pos) m/z calcd for $C_{37}H_{50}O_{18}Na$ $[M+Na]^+$: 805.2889, found: 805.2887. ^{1}H NMR (400 MHz, chloroform-*d*) δ = 7.37 – 7.33 (m, 2H), 7.21 – 7.17 (m, 2H), 5.39 (d, *J*=4.0, 1H), 5.33 (dd, *J*=10.5, 9.5, 1H), 5.20 (t, *J*=9.1, 1H), 5.03 (t, *J*=9.8, 1H), 4.90 – 4.79 (m, 3H), 4.60 – 4.53 (m, 2H), 4.49 (dd, *J*=12.1, 2.8, 1H), 4.27 – 4.21 (m, 2H), 4.05 – 3.98 (m, 2H), 3.97 – 3.93 (m, 1H), 3.66 – 3.62 (m, 1H), 2.15 (s, 3H), 2.09 (s, 3H), 2.01 (s, 6H), 1.98 (s, 6H), 1.96 (s, 3H), 1.31 (s, 9H). ^{13}C NMR (101 MHz, chloroform-*d*) δ = 170.6, 170.6, 170.6, 170.4, 170.0, 169.7, 169.5, 151.2, 133.6, 127.8, 125.5, 98.6, 95.6, 75.6, 72.8, 72.2, 72.2, 70.6, 70.1, 69.4, 68.6, 68.1, 63.0, 61.6, 34.7, 31.4, 21.0, 21.0, 20.8, 20.73, 20.70, 20.68, 20.65.

4-*tert*-butylbenzyl-β-D-maltoside (S9)

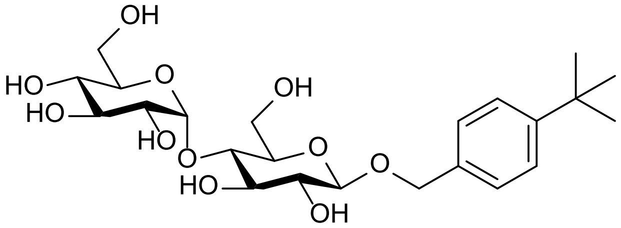

To a suspension of **S8** (4.7 g, 6.0 mmol.) in MeOH (60 mL) was added sodium methoxide (0.39 g, 1.8 mmol, 0.3 eq.) and the resulting mixture was allowed to stir overnight. Amberlite 120 H^+ was added to quench the reaction and the mixture was filtered. The product was obtained as a white solid (2.9 g, 5.9 mmol, 99%). HRMS (ESI pos) m/z calcd for $C_{23}H_{36}O_{11}Na$ $[M+Na]^+$: 511.2150 found: 511.2140. ^1H NMR (400 MHz, methanol-d_4) δ = 7.40 (d, *J*=8.4, 2H), 7.36 (d, *J*=8.5, 2H), 5.19 (d, *J*=3.8, 1H), 4.90 (d, *J*=11.6, 1H), 4.66 (d, *J*=11.6, 1H), 4.39 (d, *J*=7.8, 1H), 3.95 (dd, *J*=12.2, 2.1, 1H), 3.88 – 3.82 (m, 2H), 3.74 – 3.55 (m, 5H), 3.47 (dd, *J*=9.7, 3.7, 1H), 3.41 – 3.38 (m, 1H), 3.35 – 3.34 (m, 1H), 3.33 – 3.29 (m, 1H), 1.33 (s, 9H). ^{13}C NMR (101 MHz, methanol-d_4) δ = 151.8, 135.9, 129.1, 126.2, 103.1, 102.9, 81.3, 77.8, 76.6, 75.1, 74.8, 74.7, 74.2, 71.6, 71.5, 62.7, 62.2, 35.4, 31.8.

4-*tert*-butylbenzyl-β-3-ketomaltoside (1f)

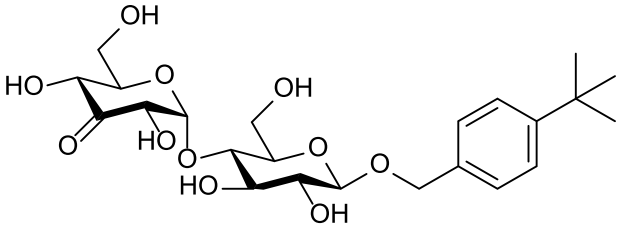

S9 (2.1 g, 4.2 mmol, 1.0 eq.) was dissolved in a dioxane/DMSO mixture (4/1 v/v, 14 mL). Benzoquinone (0.50 g, 4.6 mmol, 1.1 eq.) and $[(neocuproine)Pd(\mu\text{-}OAc)]_2(OTf)_2$ (22 mg, 0.5 mol%) were added and the reaction mixture was stirred overnight. An additional 22 mg of $[(neocuproine)Pd(\mu\text{-}OAc)]_2(OTf)_2$ was added and after 24 h again an additional 5.5 mg of $[(neocuproine)Pd(\mu\text{-}OAc)]_2(OTf)_2$. After overnight stirring, TLC analysis showed full conversion and water (120 mL) was added. The mixture was lyophilized to afford the crude product. Subsequent purification by automated flash chromatography on a 40 g silica cartridge using DCM/MeOH (0% to 7% MeOH in DCM) afforded the product as a brown oil (1.2 g, 2.5 mmol, 59%). HRMS (ESI pos) m/z calcd for $C_{23}H_{34}O_{11}Na$ $[M+Na]^+$: 509.1993, found: 509.1982. ^1H NMR (400 MHz, methanol-d_4) δ = 7.42 – 7.38 (m, 2H), 7.35 (d, *J*=8.4, 2H), 5.67 (d, *J*=4.5, 1H), 4.89 (d, *J*=11.8, 1H), 4.65 (d, *J*=11.6, 1H), 4.48 (dd, *J*=4.5, 1.5, 1H), 4.37 (d, *J*=7.8, 1H), 4.29 (dd, *J*=9.5, 1.6, 1H), 3.96 – 3.76 (m, 5H), 3.65 (t, *J*=9.0, 1H), 3.60 (t, *J*=8.9, 1H), 3.36 – 3.32 (m, 1H), 3.32 – 3.28 (m, 1H), 1.34 (s, 9H). ^{13}C NMR (101 MHz, methanol-d_4) δ = 207.0, 151.8, 135.9, 129.1, 126.1, 104.7, 103.0, 80.4, 77.9, 77.6, 76.5, 76.3, 74.8, 73.3, 71.6, 62.5, 62.1, 35.3, 31.8.

4-*tert*-butylbenzyl-β-3-(trityl)hydrazone maltoside (2f)

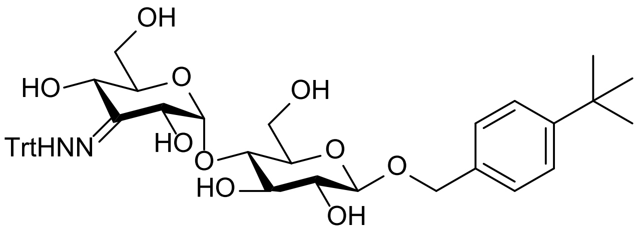

$TrtNHNH_2{\cdot}HCl$ (0.96 g, 3.1 mmol, 1.5 eq.), NaOAc (0.25 g, 3.1 mmol, 1.5 eq.), and **1f** (1.0 g 2.1 mmol, 1.0 eq.) were dissolved in a mixture of MeOH (5.7 mL), DCM (16.8 mL), and water (1 mL). The flask was subsequently evacuated and backfilled with nitrogen three times. Under N_2 flow, shielded from light, the reaction was stirred at ambient temperature overnight, after which the reaction mixture was concentrated *in vacuo*. Purification by automated flash chromatography on a 40 g silica cartridge using heptane/EtOAc (0% to 100% EtOAc in pentane) gave the title compound (1.1 g, 1.5 mmol, 72%). The product was obtained as a mixture of *E/Z* isomers. HRMS (ESI pos) m/z calcd for $C_{42}H_{51}N_2O_{10}$ $[M+H]^+$: 743.3538, found: 743.3559. Reported NMR data is for the major isomer. ^1H NMR (400 MHz, methanol-d_4) δ = 7.44 – 7.13 (m, 19H), 5.15 – 5.12 (m, 1H), 4.88 (d, *J*=11.7, 1H), 4.68 (d, *J*=11.6, 1H), 4.58 (d, *J*=3.6, 1H), 4.28 (d, *J*=7.6, 1H), 3.86 – 3.81 (m, 2H), 3.78 – 3.74 (m, 1H), 3.74 – 3.65 (m, 2H), 3.62 (dd, *J*=11.9, 5.7, 1H), 3.47 (t, *J*=9.7, 8.6, 1H), 3.33 – 3.29 (1H, overlap with the peak of CD_3OD), 3.28 – 3.17 (m, 3H), 1.32 (s, 9H). ^{13}C NMR (101 MHz, methanol-d_4) δ = 152.0, 146.9, 140.9, 135.7, 130.2, 129.4, 128.7, 127.6, 126.2, 104.1, 102.3, 81.8, 77.9, 77.7, 76.3, 75.5, 74.4, 74.0, 71.3, 68.4, 62.7, 62.2, 35.4, 31.8.

4-*tert*-butylbenzyl-3-chloro-3-deoxy-β-D-maltoside (3f)

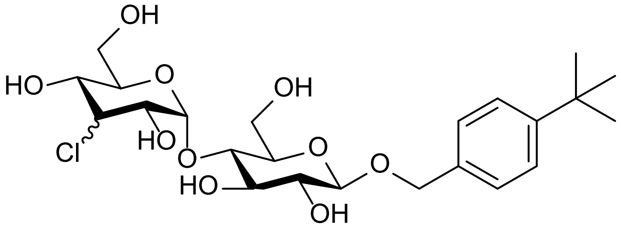

In a 100 mL flask equipped with a magnetic stir bar, **2f** (0.37 g, 0.50 mmol, 1.0 eq.) was dissolved in anhydrous THF (5.0 mL) and the resulting light-yellow solution was evacuated and backfilled with nitrogen three times. After cooling to -20 °C (external temperature), *tert*-butyl hypochlorite (1.24 M in anhydrous THF, 0.44 mL, 1.1 eq.) was added dropwise to the cooled solution of hydrazone and stirred for 15 min. The solution was then frozen in a liquid N_2 bath and degassed by two freeze-pump-thaw cycles, each time thawing in a -20 °C bath. After backfilling with N_2, the reaction was maintained at an external temperature ≤ -15 °C for 20 min. During this time *tert*-butylthiol was degassed once by freeze-pump-thaw, followed by the addition of *tert*-butylthiol (4.5 mL, 40 mmol, 80 eq.) to the cooled reaction. The reaction flask was subsequently transferred to a pre-heated 60°C heating mantle and the flask was covered in aluminum foil. After 1 hour, the reaction was allowed to cool to ambient temperature and concentrated *in vacuo*. Purification by automated flash chromatography on a 25 g silica cartridge using DCM/MeOH (0% to 5% MeOH in DCM) provided the product as a colorless oil (0.19 g, 0.37 mmol, 74%). The ratio between the equatorial and axial product is 1/4.4. **3f-equatorial:** HRMS (ESI pos) m/z calcd for $C_{23}H_{35}ClO_{10}Na$ $[M+Na]^+$: 529.1811 and 531.1782, found: 529.1801 and 531.1769. ^1H NMR (400 MHz, methanol-d_4) δ = 7.40 (d, *J*=8.5, 2H), 7.36 (d, *J*=8.4, 2H), 5.25 (d, *J*=3.7, 1H), 4.90 (d, *J*=11.7, 1H), 4.66 (d, *J*=11.6, 1H), 4.39 (d, *J*=7.8, 1H), 3.98 – 3.90 (m, 2H), 3.88 – 3.81 (m, 2H), 3.76 – 3.69 (m, 2H), 3.67 – 3.58 (m, 3H), 3.49 (t, *J*=9.3, 1H), 3.42 – 3.38 (m, 1H), 3.32 – 3.28 (m, 1H), 1.33 (s, 9H). ^{13}C NMR (101 MHz, methanol-d_4) δ = 151.9, 135.9, 129.2, 126.2, 103.0, 102.3, 81.1, 77.8, 76.5, 75.3, 74.8, 74.5, 71.8, 71.6, 67.4, 62.5, 62.2, 35.4, 31.8. **3f-axial:** HRMS (ESI pos) m/z calcd for $C_{23}H_{35}ClO_{10}Na$ $[M+Na]^+$: 529.1811 and 531.1782, found: 529.1797 and 531.1767. ^1H NMR (400 MHz, methanol-d_4) δ = 7.40 (d, *J*=8.4, 2H), 7.36 (d, *J*=8.4, 2H), 5.28 (d, *J*=4.2, 1H), 4.91 (d, *J*=11.6, 1H), 4.66 (d, *J*=11.6, 1H), 4.52 (t, *J*=3.7, 1H), 4.38 (d, *J*=7.8, 1H), 4.00 – 3.93 (m, 3H), 3.92 – 3.85 (m, 2H), 3.81 (dd, *J*=9.7, 3.4, 1H), 3.74 (dd, *J*=11.9, 5.6, 1H), 3.69 – 3.59 (m, 2H), 3.40 – 3.36 (1H, overlap with the peak of CD_3OD), 3.30 (t, *J*=8.3, 1H), 1.34 (s, 9H). ^{13}C NMR (101 MHz, methanol-d_4) δ = 151.8, 135.9, 129.2, 126.1, 103.1, 101.0, 79.3, 77.9, 76.8, 75.2, 71.6, 70.0, 69.3, 67.4, 66.4, 62.4, 62.2, 35.4, 31.8.

Synthesis of β-Glc derivatives

Methyl 3-keto-β-D-glucopyranoside (1g)

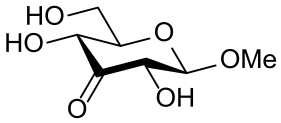

Methyl β-D-glucopyranoside (2.0 g, 10 mmol, 1.0 eq.) and benzoquinone (1.2 g, 11 mmol, 1.1 eq.) were dissolved in MeOH (41 mL). The catalyst [(neocuproine)Pd(μ-OAc)]$_2$(OTf)$_2$ (0.22 g, 0.02 mol%) was added and the mixture was stirred at room temperature for 3.5 h. The reaction mixture was concentrated *in vacuo* and subsequent purification by automated flash chromatography on an 80 g silica cartridge using heptane/EtOAc (0 to 100% EtOAc in heptane) afforded **1g** as a white solid (1.1 g, 5.6 mmol, 55%). HRMS (ESI neg) m/z calcd for $C_7H_{11}O_6$ $[M-H]^-$: 191.0561, found: 191.0563. ^1H NMR (400 MHz, methanol-d_4) δ = 4.32 (d, *J*=7.9, 1H), 4.26 (dd, *J*=10.2, 1.8, 1H), 4.15 (dd, *J*=7.9, 1.8, 1H), 3.98 (dd, *J*=12.1, 2.2, 1H), 3.83 (dd, *J*=12.1, 4.9, 1H), 3.62 (s, 3H), 3.36 – 3.33 (m, 1H). ^{13}C NMR (101 MHz, methanol-d_4) δ = 207.0, 106.7, 78.2, 78.2, 73.6, 62.5, 57.5.

Methyl-3-(trityl)hydrazone-β-D-glucopyranoside (2g)

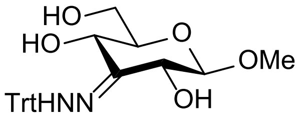

In a 100 mL flask TrtNHNH$_2$·HCl (1.5 g, 4.7 mmol, 1.5 eq.), NaOAc·3H$_2$O (0.38 g, 4.7 mmol, 1.5 eq.), and **1g** (0.6 g 3.1 mmol, 1.0 eq.) were dissolved in CH_3OH (16 mL). The flask was subsequently evacuated and backfilled with nitrogen three times. Under N_2 flow, shielded from light, the reaction was stirred at 50 °C overnight, after which the reaction mixture was concentrated *in vacuo*. Purification by automated flash chromatography on a 24 g silica cartridge using heptane/EtOAc (0% to 100% EtOAc in heptane) gave the hydrazone as a mixture of

E/Z isomers as yellow semi solid (0.73 g, 1.5 mmol, 48%). HRMS (ESI pos) m/z calcd for $C_{26}H_{26}N_2O_5Na$ $[M+Na]^+$: 471.1890, found: 471.1987. Reported NMR data is for the major isomer. ^{1}H NMR (400 MHz, methanol-*d*$_4$) δ = 7.37 – 7.16 (m, 15H), 4.21 (d, *J*=7.4, 1H), 4.02 (d, *J*=7.5, 1H), 3.75 – 3.54 (m, 4H), 3.49 (s, 3H), 2.81 (ddd, *J*=9.1, 5.8, 2.5, 1H). ^{13}C NMR (101 MHz, methanol-*d*$_4$) δ = 146.9, 130.1, 128.7, 127.7, 106.4, 80.8, 75.9, 68.9, 62.9, 57.3.

Methyl 3-chloro-3-deoxy-β-D-allo/glucopyranoside (3g)

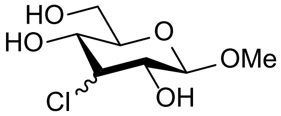

A 100 mL flask equipped with a magnetic stir bar was charged with trityl hydrazone **2g** (0.19 g, 0.40 mmol, 1.0 eq.) and anhydrous THF (4 mL). The resulting solution was evacuated and backfilled with nitrogen three times and then cooled to -20 °C (external temperature). *Tert*-Butyl hypochlorite (1.24 M in anhydrous DCM, 0.35 mL, 1.1 eq.) was added dropwise to the cooled solution of hydrazone and stirred for 15 min. The resulting light-yellow solution was then frozen in a liquid N_2 bath and degassed by two freeze-pump-thaw cycles, each time thawing in a -20 °C bath. After backfilling with N_2, the reaction was maintained at an external temperature ≤ -15 °C for 20 min. During this time *tert*-nonyl mercaptan was degassed in a separated flask by a single freeze-pump-thaw cycle. Excess *tert*-nonyl mercaptan (5.9 mL, 32 mmol, 80 eq.) was added to the cooled reaction. The reaction flask was subsequently transferred to a pre-heated 60 °C heating mantle and the flask was covered in aluminum foil. After 2 h, the reaction was allowed to cool to ambient temperature. Then the reaction mixture was concentrated *in vacuo* and purification by automated flash chromatography on a 15 g silica cartridge using DCM/MeOH (0 to 10% MeOH in DCM) gave the title compound (72 mg, 0.34 mmol, 86%). The NMR analysis shows an equatorial to axial ratio of 1/1.7. HRMS (ESI neg) m/z calcd for $C_7H_{12}ClO_5Na$ $[M-H]^-$: 211.0379 and 213.0349, found: 211.0380 and 213.0350. Reported NMR data is for the major compound (axial). ^{1}H NMR (400 MHz, methanol-*d*$_4$) δ = 4.61 – 4.56 (m, 2H), 3.91 – 3.80 (m, 2H), 3.77 (dd, *J*=5.1, 2.2, 1H), 3.75 – 3.64 (m, 1H), 3.58 (dd, *J*=7.6, 3.4, 1H), 3.52 (s, 3H). ^{13}C NMR (101 MHz, methanol-*d*$_4$) δ = 102.5, 75.7, 71.1, 69.0, 67.9, 62.5, 57.2.

Methyl-3-(2,4-dinitrophenyl)hydrazone-β-D-glucopyranoside (6b)

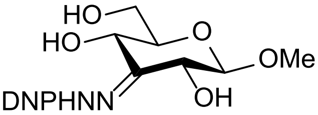

1g (0.19 g 1.0 mmol, 1.0 eq.) and dinitrophenyl hydrazine (contains 30% H_2O, 0.4 g, 1.3 mmol, 1.3 eq.) were dissolved in MeOH (2.5 mL). AcOH (11 µL, 0.20 mmol, 0.2 eq.) was added and the reaction mixture was stirred overnight at 50 °C while kept in the dark. TLC analysis showed full conversion and the reaction mixture was concentrated *in vacuo*. Purification by flash chromatography using pentane/EtOAc (0% to 100% EtOAc in pentane) gave the hydrazone as a mixture of *E/Z* isomers as an orange powder (0.21 g, 0.58 mmol, 58%). HRMS (ESI pos) m/z calcd for $C_{13}H_{17}N_4O_9$ $[M+H]^+$: 373.0990, found: 373.0999. Reported NMR data is for the major isomer. ^{1}H NMR (400 MHz, acetonitrile-*d*$_3$) δ = 8.96 (d, *J*=2.7, 1H), 8.28 (dd, *J*=9.7, 2.6, 1H), 8.07 (d, *J*=9.5, 1H), 4.45 (m, 2H), 4.11 – 4.06 (m, 1H), 3.88 – 3.83 (m, 1H), 3.76 – 3.69 (m, 1H), 3.56 (s, 3H), 3.34 – 3.29 (m, 1H). ^{13}C NMR (101 MHz, acetonitrile-*d*$_3$) δ = 151.7, 146.0, 130.6, 124.2, 116.6, 105.1, 79.6, 76.3, 69.6, 62.6, 57.5.

Methyl-3-chloro-3-(2,4-dinitrophenyl)diazene-β-D-glucopyranoside (7b)

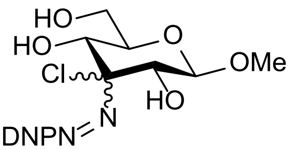

To hydrazone **6b** (53 mg, 0.14 mmol, 1.0 eq.) was added dry dioxane (2 mL). The suspension was evacuated and backfilled with nitrogen three times. *tert*-Butyl hypochlorite (1.24 M in dry dioxane, 0.17 mL, 1.5 eq.) was added dropwise to the suspension. After stirring overnight, the resulting solution was lyophilized to afford a mixture of unreacted hydrazone **6b** and the title compound. The yield could not be determined. HRMS (ESI neg) m/z calcd for $C_{13}H_{14}ClN_4O_9$ $[M-H]^-$: 405.0455 and 407.0425, found: 405.0446 and 407.0593. The product was not isolated. The NMR spectrum of the crude can be found at the NMR spectra.

Scale-up synthesis

Methyl 3-chloro-3-deoxy-α-D-allo/glucopyranoside (3b)

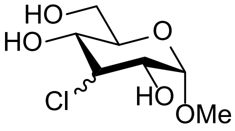

A 100 mL flask equipped with a magnetic stir bar was charged with trityl hydrazone **2b** (0.48 g, 1.0 mmol, 1.0 eq.) and anhydrous THF (10 mL). The resulting solution was evacuated and backfilled with nitrogen three times and then cooled to -20 °C (external temperature). *tert*-Butyl hypochlorite (1.24 M in anhydrous DCM, 0.89 mL, 1.1 eq.) was added dropwise to the cooled solution of the hydrazone and stirred was continued for 15 min. The resulting light-yellow solution was then frozen in a liquid N_2 bath and degassed by two freeze-pump-thaw cycles, each time thawing in a -20 °C bath. After backfilling with N_2, the reaction was maintained at an external temperature ≤ -15 °C for 35 min. During this time, *t*BuSH was purged for 5 min with nitrogen. Excess *t*BuSH (9 mL, 80 mmol, 80 eq.) was added to the cooled reaction. The reaction flask was subsequently transferred to a pre-heated 60 °C heating mantle and covered in aluminum foil. After 2 h, the reaction was allowed to cool to ambient temperature. Then the reaction mixture was concentrated *in vacuo* and diluted with pentane/Et_2O (1/1 v/v). The organic layer was extracted with water (2x). The combined aqueous layers were concentrated *in vacuo* to give the title compound (197 mg, 0.92 mmol, 92%). The NMR analysis shows an equatorial to axial ratio of 1/2.7. The analysis is in agreement with the experiment described earlier.

Methyl 3-chloro-3-deoxy-α-D-allo/glucopyranoside (3b)

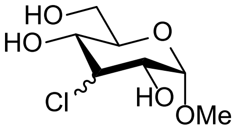

A 100 mL flask equipped with a magnetic stir bar was charged with trityl hydrazone **2b** (1.2 g, 2.50 mmol, 1.0 eq.) and anhydrous THF (25 mL). The resulting solution was evacuated and backfilled with nitrogen three times and then cooled to -20 °C (external temperature). *tert*-Butyl hypochlorite (1.24 M in anhydrous DCM, 2.2 mL, 1.1 eq.) was added dropwise to the cooled solution of the hydrazone and stirring was continued for 15 min. The resulting light-yellow solution was then frozen in a liquid N_2 bath and degassed by two freeze-pump-thaw cycles, each time thawing in a -20 °C bath. After backfilling with N_2, the reaction was maintained at an external temperature ≤ -15 °C for 25 min. During this time AcSH was degassed in a separated flask by a single freeze-pump-thaw cycle. Excess AcSH (0.7 mL, 10 mmol, 4 eq.) was added to the cooled reaction. The reaction flask was subsequently transferred to a pre-heated 60 °C heating mantle and covered in aluminum foil. After 2.5 h, the reaction was allowed to cool to ambient temperature. Then the reaction mixture was concentrated *in vacuo* and the product was purified by automated flash chromatography on a 15 g silica cartridge using DCM/MeOH (0 to 20% MeOH in DCM), which gave the title compound (0.36 g, 1.7 mmol, 67%). The NMR analysis shows an equatorial to axial ratio of 1/1. The analysis is in agreement with the experiment described earlier.

Crystal structures

Methyl 3-keto-α-D-xylopyranoside (1d)

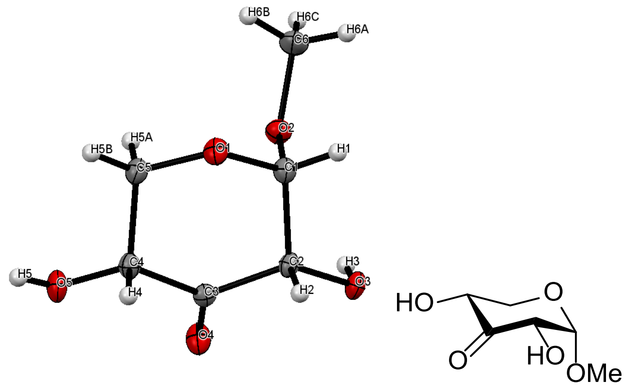

Figure S1. Molecular structure of compound **1d**, showing 50% probability ellipsoids. CCDC 2150537

Methyl 3-keto-α-D-xylopyranoside (**1d**) was isolated after column chromatography as a colorless syrup, which crystallized upon standing. A single crystal of compound **1d** was mounted on top of a cryoloop and transferred into the cold nitrogen stream (100 K) of a Bruker-AXS D8 Venture diffractometer. Data collection and reduction was done using the Bruker software suite APEX3. The final unit cell was obtained from the xyz centroids of 5440 reflections after integration. A multiscan absorption correction was applied, based on the intensities of symmetry-related reflections measured at different angular settings (*SADABS*). The structures were solved by direct methods using *SHELXT*[6] and refinement of the structure was performed using *SHELXL*.[7] The hydrogen atoms were generated by geometrical considerations, constrained to idealized geometries and allowed to ride on their carrier atoms with an isotropic displacement parameter related to the equivalent displacement parameter of their carrier atoms. The absolute structure was chosen based on the known configuration of the starting material. Crystal data and details on data collection and refinement are presented in Table S2.

Table S2. Crystallographic data for **1d**

chem formula	C6 H10 O5		temp (K)	100(2)
M_r	162.14		θ range (deg)	3.491 - 27.893
cryst syst	orthorhombic		data collected (h,k,l)	-7:6, -11:11, -20:20
color, habit	colorless, needle		no. of rflns collected	8208
size (mm)	0.40 x 0.19 x 0.07		no. of indpndt reflns	1738
space group	$P2_12_12_1$		observed reflns	1657($F_o \geq 2\ \sigma(F_o)$)
a (Å)	5.3384(3)		R(F) (%)	3.24
b (Å)	8.9055(5)		wR(F^2) (%)	8.07
c (Å)	15.4503(8)		GooF	1.101
V (Å^3)	734.52(7)		Weighting a,b	0.0351, 0.3135
Z	4		params refined	105
ρ_{calc}, g.cm^{-3}	1.466		restraints	0
μ(Mo K$\bar{\alpha}$), cm^{-1}	0.129		min, max resid dens	-0.212, 0.307
F(000)	344			

Methyl 3-(trityl)hydrazone-α-D-xylopyranoside (2d)

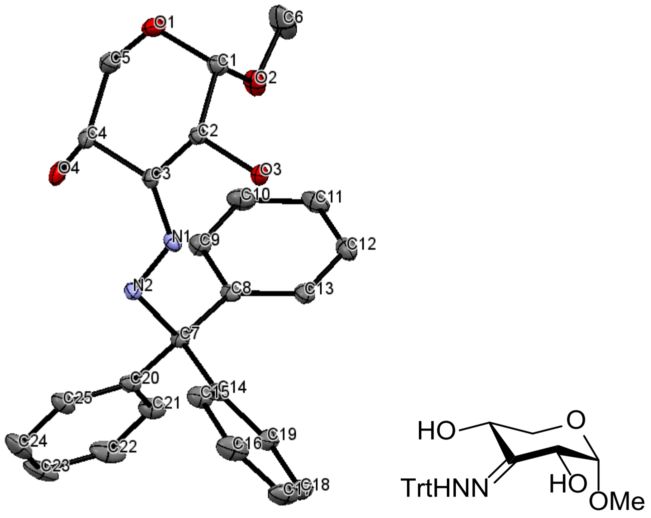

Figure S2. Molecular structure of compound **2d** (*E*-isomer), showing 50% probability ellipsoids. CCDC 2150551

Methyl 3-(trityl)hydrazone-α-D-xylopyranoside (**2d**) was isolated after column chromatography as a colorless syrup, the E isomer formed crystals immediately after evaporation. A single crystal of compound **2d** (*E*-isomer) was mounted on top of a cryoloop and transferred into the cold nitrogen stream (100 K) of a Bruker-AXS D8 Venture diffractometer. Data collection and reduction was done using the Bruker software suite APEX3. The final unit cell was obtained from the xyz centroids of 9856 reflections after integration. A multiscan absorption correction was applied, based on the intensities of symmetry-related reflections measured at different angular settings (*SADABS*). The structures were solved by direct methods using *SHELXT*[6] and refinement of the structure was performed using *SHELXL*.[7] The hydrogen atoms were generated by geometrical considerations, constrained to idealized geometries and allowed to ride on their carrier atoms with an isotropic displacement parameter related to the equivalent displacement parameter of their carrier atoms. The absolute structure was chosen based on the known configuration of the starting material. Crystal data and details on data collection and refinement are presented in Table S3.

Table S3. Crystallographic data for **2d** (*E*-isomer)

chem formula	C25 H26 N2 O4		temp (K)	100(2)
M_r	418.48		θ range (deg)	3.007 - 28.763
cryst syst	orthorhombic		data collected (h,k,l)	-11:11, -16:16, -27:27
color, habit	colorless, block		no. of rflns collected	49183
size (mm)	0.57 x 0.30 x 0.15		no. of indpndt reflns	5743
space group	$P2_12_12_1$		observed reflns	5471($F_o \geq 2\sigma(F_o)$)
a (Å)	8.8434(6)		R(F) (%)	3.19
b (Å)	12.3334(7)		wR(F^2) (%)	7.98
c (Å)	20.3036(13)		GooF	1.050
V (Å^3)	2214.5(2)		Weighting a,b	0.0445, 0.4440
Z	4		params refined	288
ρ_{calc}, g.cm^{-3}	1.255		restraints	0
μ(Mo K$\bar{\alpha}$), cm^{-1}	0.085		min, max resid dens	-0.215, 0.263
F(000)	888			

Isopropyl 2-acetmido-3-chloro-2,3-dideoxy-α-D-allopyranoside (3a)

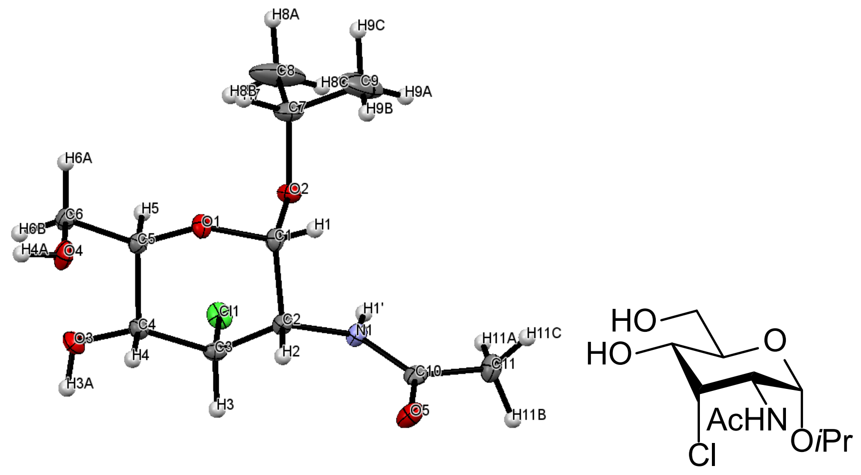

Figure S3. Molecular structure of compound **3a** (3-axial), showing 50% probability ellipsoids. CCDC 2150550

Isopropyl 2-acetamido-3-chloro-2,3-dideoxy-α-D-allo/glucopyranoside (**3a-axial**) was isolated after column chromatography as a colorless syrup, which crystallized upon standing. A single crystal of compound **3a** (3-axial) was mounted on top of a cryoloop and transferred into the cold nitrogen stream (100 K) of a Bruker-AXS D8 Venture diffractometer. Data collection and reduction was done using the Bruker software suite APEX3. The final unit cell was obtained from the xyz centroids of 9903 reflections after integration. A multiscan absorption correction was applied, based on the intensities of symmetry-related reflections measured at different angular settings (*SADABS*). The structures were solved by direct methods using *SHELXT*[6] and refinement of the structure was performed using *SHELXL*.[7] The hydrogen atoms were generated by geometrical considerations, constrained to idealized geometries and allowed to ride on their carrier atoms with an isotropic displacement parameter related to the equivalent displacement parameter of their carrier atoms. The absolute structure was chosen based on a refinement of Flack's parameter (x = 0.002(12)). Crystal data and details on data collection and refinement are presented in Table S4.

Table S4. Crystallographic data for **3a** (3-axial)

chem formula	C11 H20 Cl N O5		temp (K)	100(2)
M_r	281.73		θ range (deg)	2.937 - 27.149
cryst syst	orthorhombic		data collected (h,k,l)	-8:8, -10:10, -31:30
color, habit	colorless, block		no. of rflns collected	16872
size (mm)	0.36 x 0.32 x 0.08		no. of indpndt reflns	3081
space group	$P2_12_12_1$		observed reflns	2987($F_o \geq 2\ \sigma(F_o)$)
a (Å)	6.8245(2)		R(F) (%)	2.55
b (Å)	8.4581(3)		wR(F^2) (%)	6.23
c (Å)	24.2453(8)		GooF	1.123
V (Å^3)	1399.49(8)		Weighting a,b	0.0276, 0.4412
Z	4		params refined	170
ρ_{calc}, g.cm^{-3}	1.337		restraints	0
μ(Mo K$\bar{\alpha}$), cm^{-1}	0.286		min, max resid dens	-0.238, 0.218
F(000)	600			

Methyl 3-chloro-3-deoxy-α-D-allopyranoside (3b)

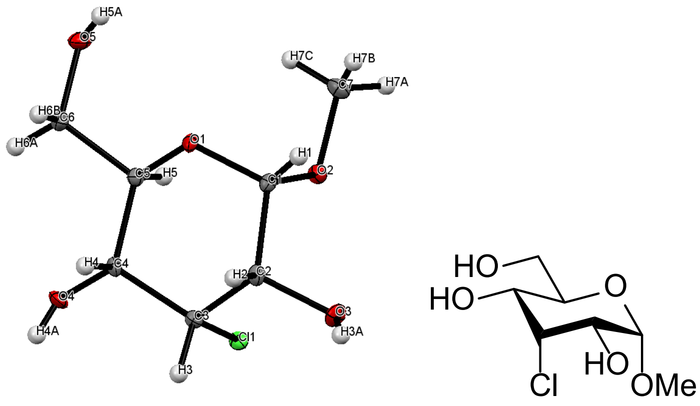

Figure S4. Molecular structure of compound **3b (3-axial)**, showing 50% probability ellipsoids. CCDC 2150508

Methyl 3-chloro-3-deoxy-α-D-allo/glucopyranoside (**3b-axial**) was crystallized from methanol. A single crystal of compound **3b (3-axial)** was mounted on top of a cryoloop and transferred into the cold nitrogen stream (100 K) of a Bruker-AXS D8 Venture diffractometer. Data collection and reduction was done using the Bruker software suite APEX3. The final unit cell was obtained from the xyz centroids of 7756 reflections after integration. A multiscan absorption correction was applied, based on the intensities of symmetry-related reflections measured at different angular settings (*SADABS*). The structures were solved by direct methods using *SHELXT*[6] and refinement of the structure was performed using *SHELXL*.[7] Refinement was initially poor, with several atoms having non-positive definite displacement parameters when allowed to refine anisotropically. The *PLATON*/*TWINROTMAT* routine was applied to check for possible twinning. Twinning was identified and subsequent refinement with the appropriate twin law proceeded smoothly resulting in a BASF of 0.34. The hydrogen atoms were generated by geometrical considerations, constrained to idealized geometries and allowed to ride on their carrier atoms with an isotropic displacement parameter related to the equivalent displacement parameter of their carrier atoms. The absolute structure was chosen based on the known configuration of the starting material. Crystal data and details on data collection and refinement are presented in Table S5.

Table S5. Crystallographic data for **3b (3-axial)**

chem formula	C7 H13 Cl O5		temp (K)	100(2)
M_r	212.62		θ range (deg)	3.090 - 27.870
cryst syst	tetragonal		data collected (h,k,l)	-17:17, -17:17, -6:6
color, habit	colorless, needle		no. of rflns collected	14795
size (mm)	0.64 x 0.08 x 0.08		no. of indpndt reflns	2145
space group	$P4_1$		observed reflns	2127($F_o \geq 2\ \sigma(F_o)$)
a (Å)	13.1870(7)		R(F) (%)	1.81
b (Å)	13.1870(7)		wR(F^2) (%)	4.26
c (Å)	5.1745(2)		GooF	1.075
V (Å^3)	899.83(10)		Weighting a,b	0.0261, 0.0713
Z	4		params refined	126
ρ_{calc}, g.cm^{-3}	1.569		restraints	1
μ(Mo K$\overline{\alpha}$), cm^{-1}	0.413		min, max resid dens	-0.208, 0.194
F(000)	448			

Supplemental references

(1) Zhang, J.; Eisink, N. N. H. M.; Witte, M. D.; Minnaard, A. J. Regioselective Manipulation of GlcNAc Provides Allosamine, Lividosamine, and Related Compounds. *J. Org. Chem.* **2019**, *84* (2), 516–525.

(2) Ho, W. C.; Chung, K.; Ingram, A. J.; Waymouth, R. M. Pd-Catalyzed Aerobic Oxidation Reactions: Strategies to Increase Catalyst Lifetimes. *J. Am. Chem. Soc.* **2018**, *140* (2), 748–757.

(3) Marinus, N.; Tahiri, N.; Duca, M.; Mouthaan, L. M. C. M.; Bianca, S.; van den Noort, M.; Poolman, B.; Witte, M. D.; Minnaard, A. J. Stereoselective Protection-Free Modification of 3-Keto-Saccharides. *Org. Lett.* **2020**, *22* (14), 5622–5626.

(4) Muramatsu, W. Catalytic and Regioselective Oxidation of Carbohydrates To Synthesize Keto-Sugars under Mild Conditions. *Org. Lett.* **2014**, *16* (18), 4846–4849.

(5) Wang, R.; Chen, J.-Z.; Zheng, X.-A.; Kong, R.; Gong, S.-S.; Sun, Q. Hafnium(IV) Triflate as a Potent Catalyst for Selective 1-O-Deacetylation of Peracetylated Saccharides. *Carbohydr. Res.* **2018**, *455*, 114–118.

(6) Sheldrick, G. M. SHELXT – Integrated Space-Group and Crystal-Structure Determination. *Acta Crystallogr. Sect. A Found. Adv.* **2015**, *71* (1), 3–8.

(7) Sheldrick, G. M. A Short History of SHELX. *Acta Crystallogr. Sect. A Found. Crystallogr.* **2008**, *64* (1), 112–122.

NMR spectraNMR spectra of GlcNAc derivatives

Isopropyl-2-acetamido-2-deoxy-3-(trityl)hydrazone-α-D-glucopyranoside (2a)

^{1}H NMR, 400 MHz, CD$_3$OD of compound **2a**

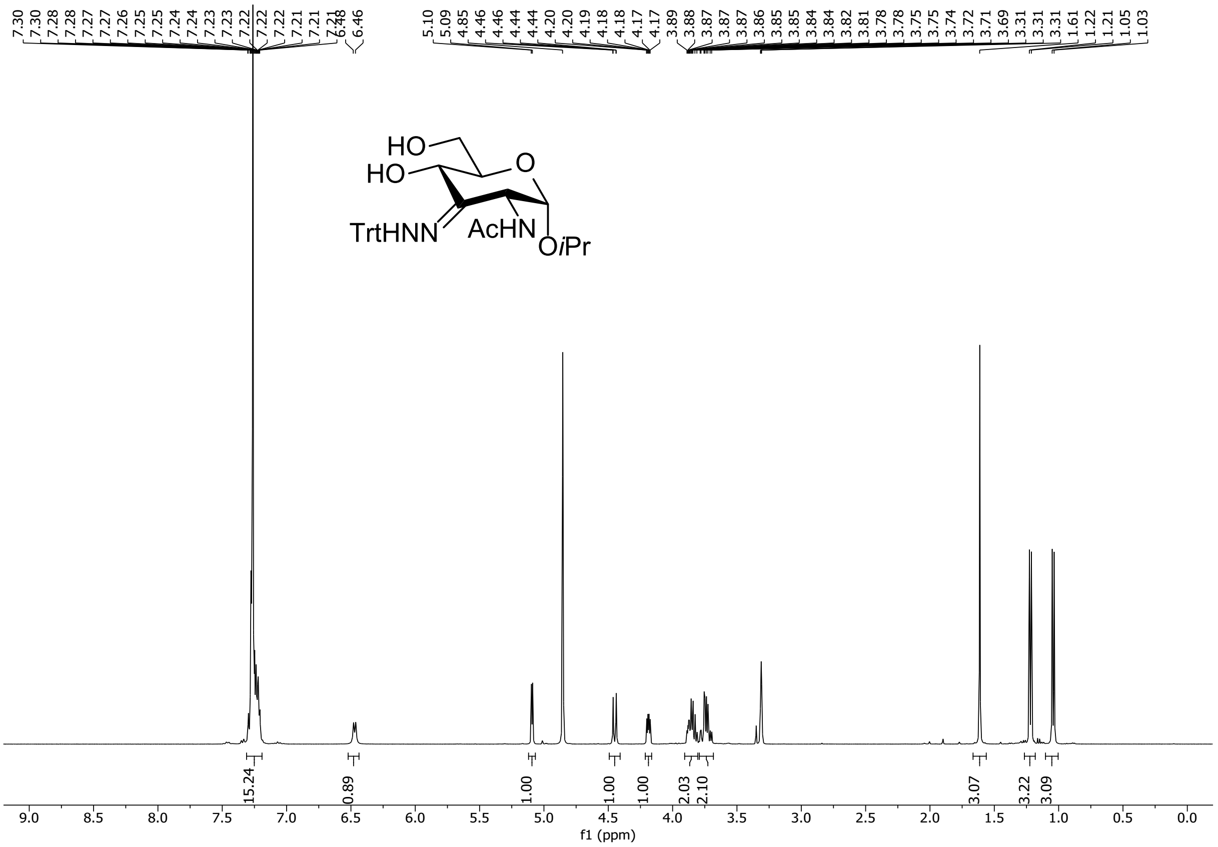

^{13}C NMR, 400 MHz, CD$_3$OD of compound **2a**

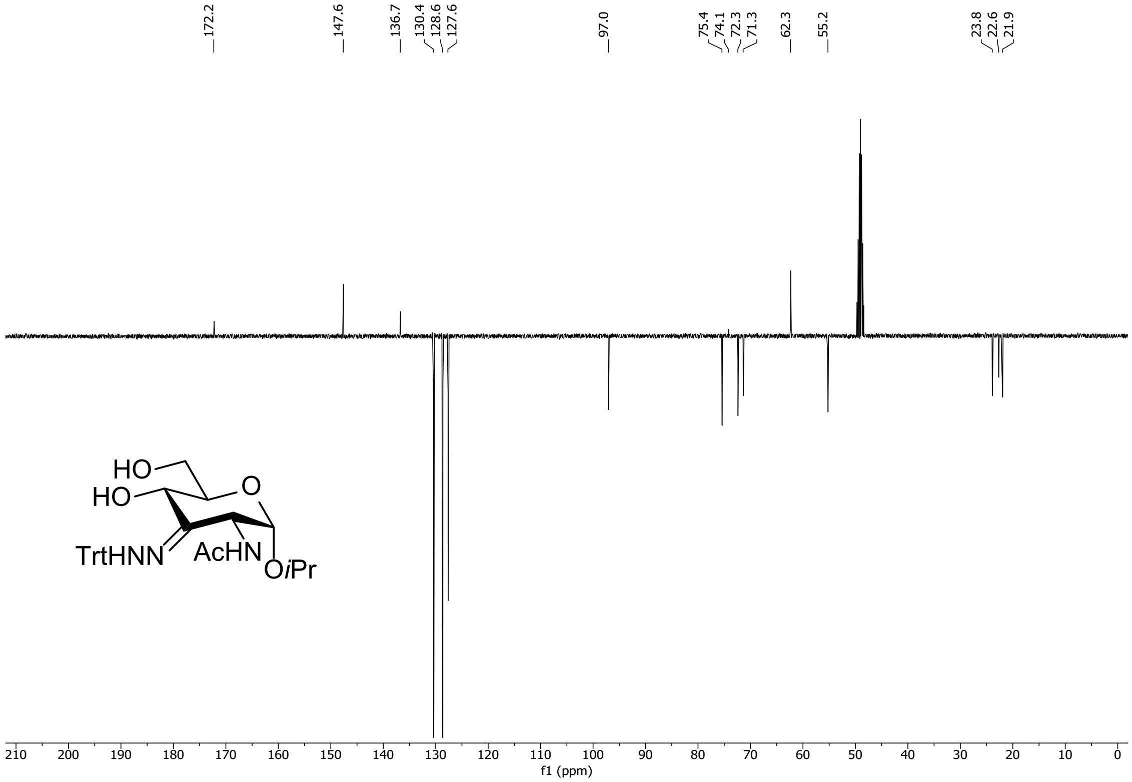

^{1}H-^{1}H COSY of compound **2a**

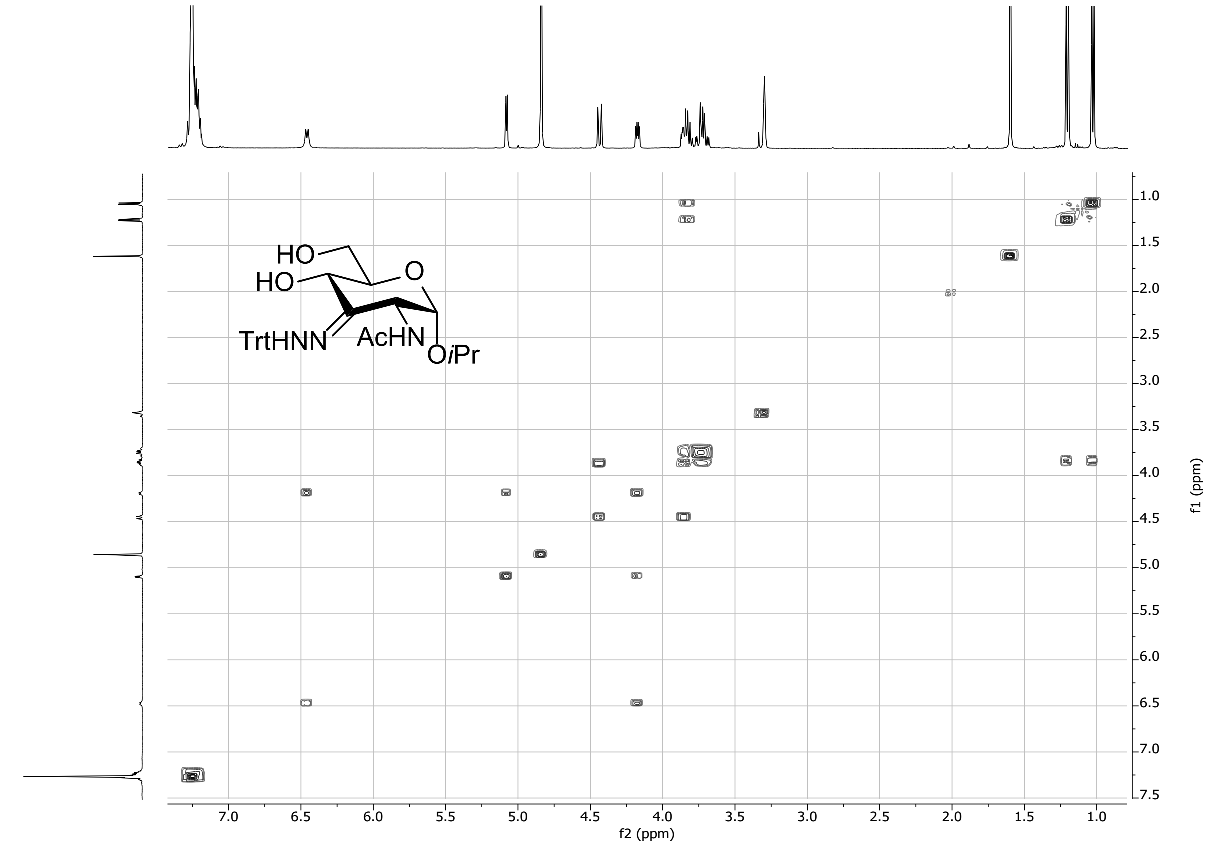

^{1}H-^{13}C HSQC of compound **2a**

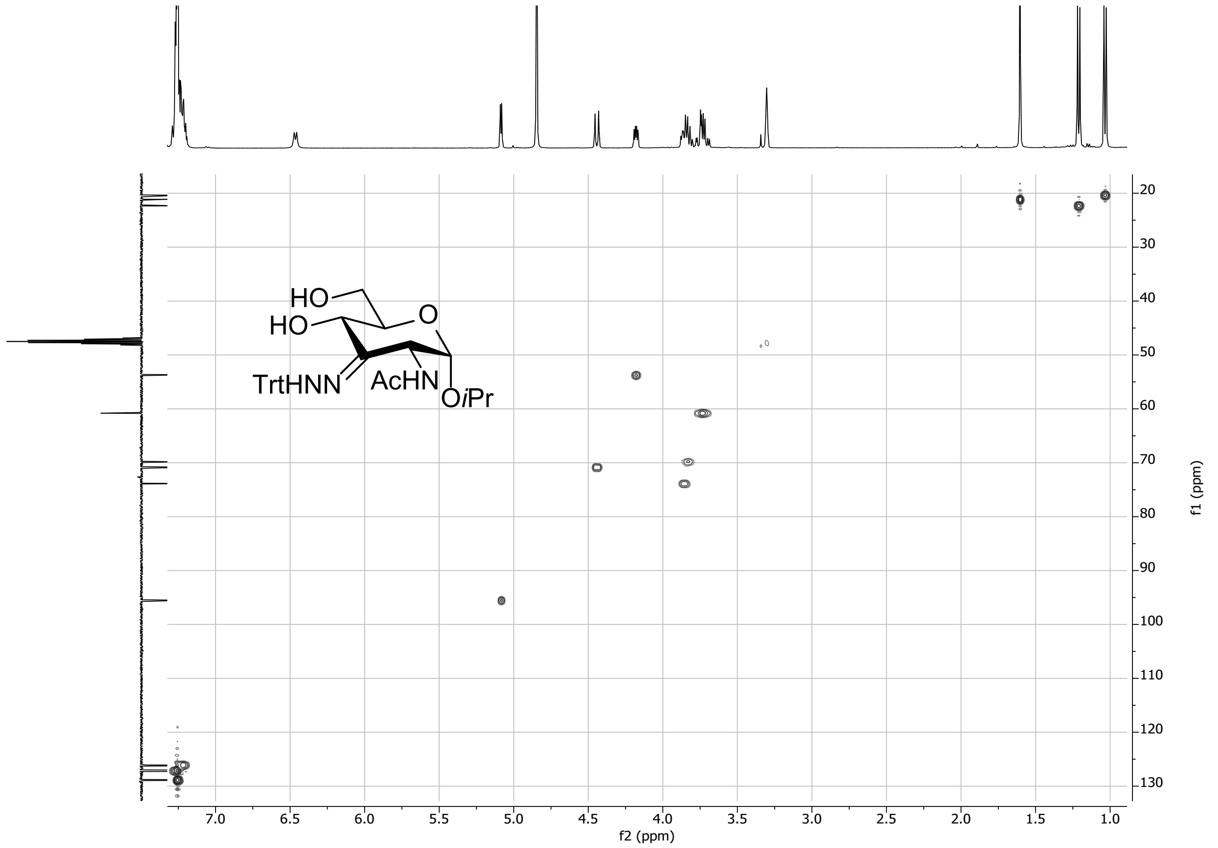

Isopropyl 2-acetmido-3-chloro-2,3-dideoxy-α-D-allo/glucopyranoside (3a)

Mixture of equatorial and axial, 3-equtorial : 3-axial ≈ 1.4 : 1

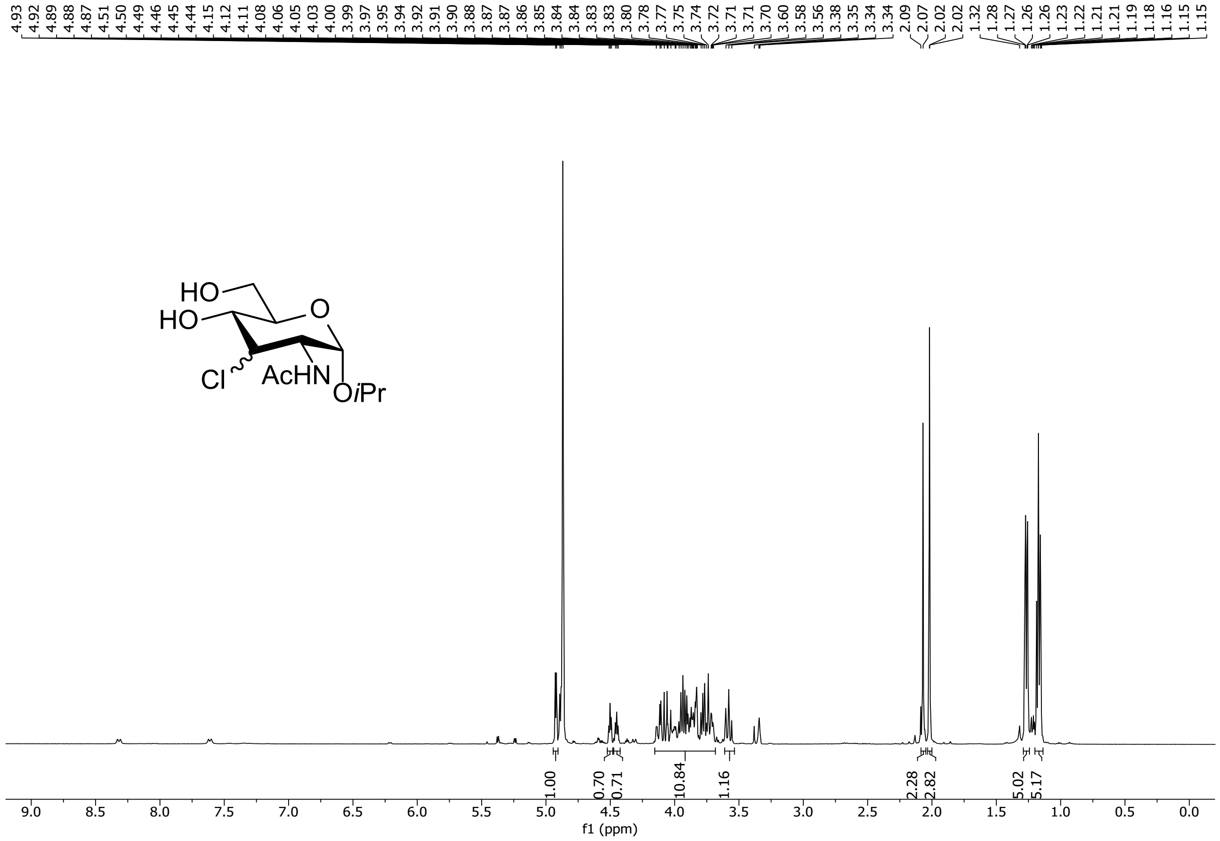

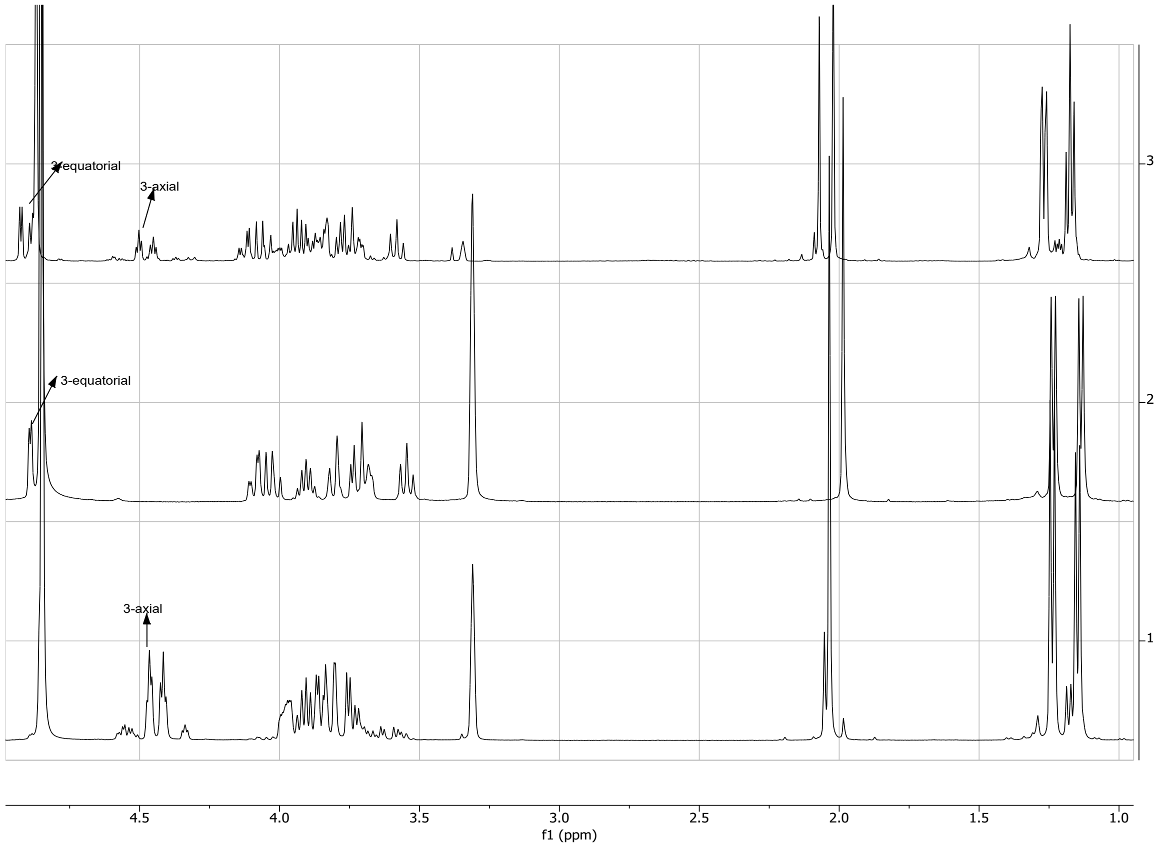

^{1}H NMR, 400 MHz, CD_3OD of compound **3a-equatorial**

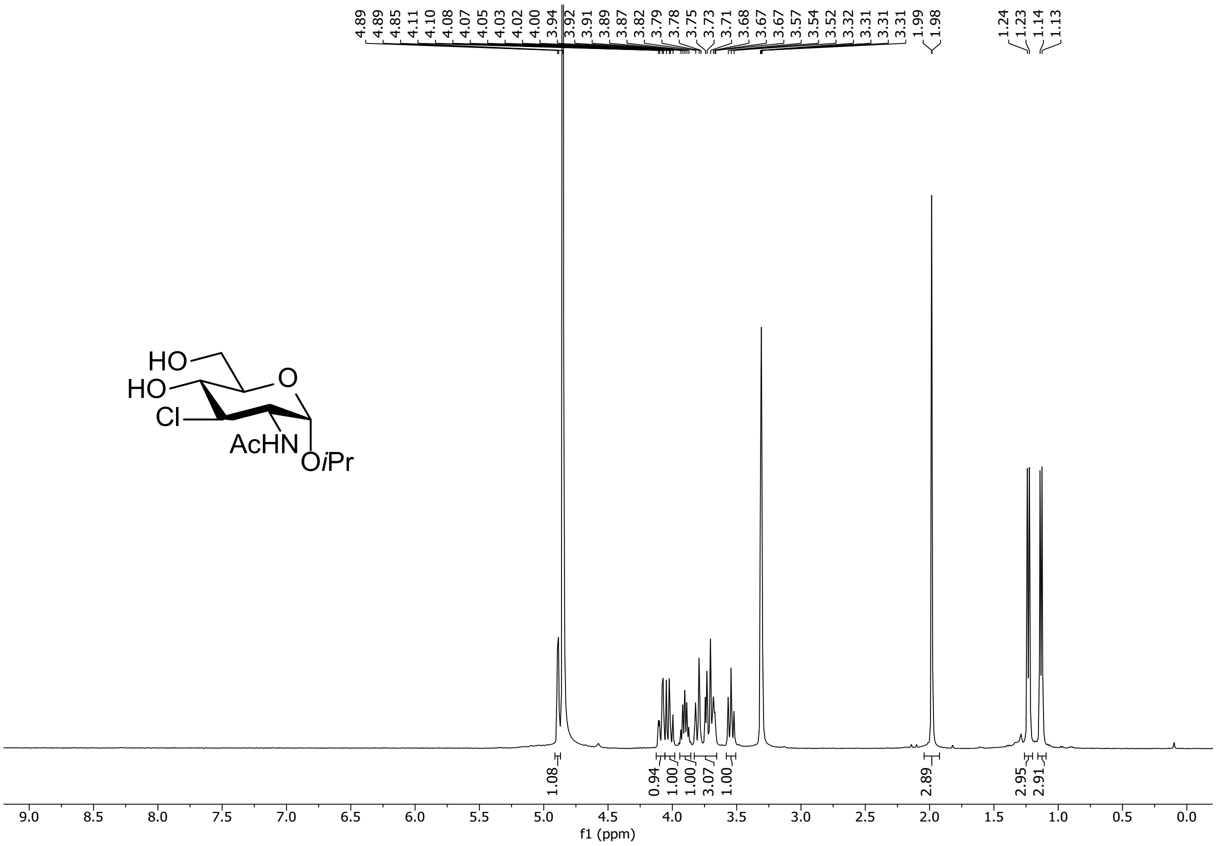

^{13}C NMR, 400 MHz, CD_3OD of compound **3a-equatorial**

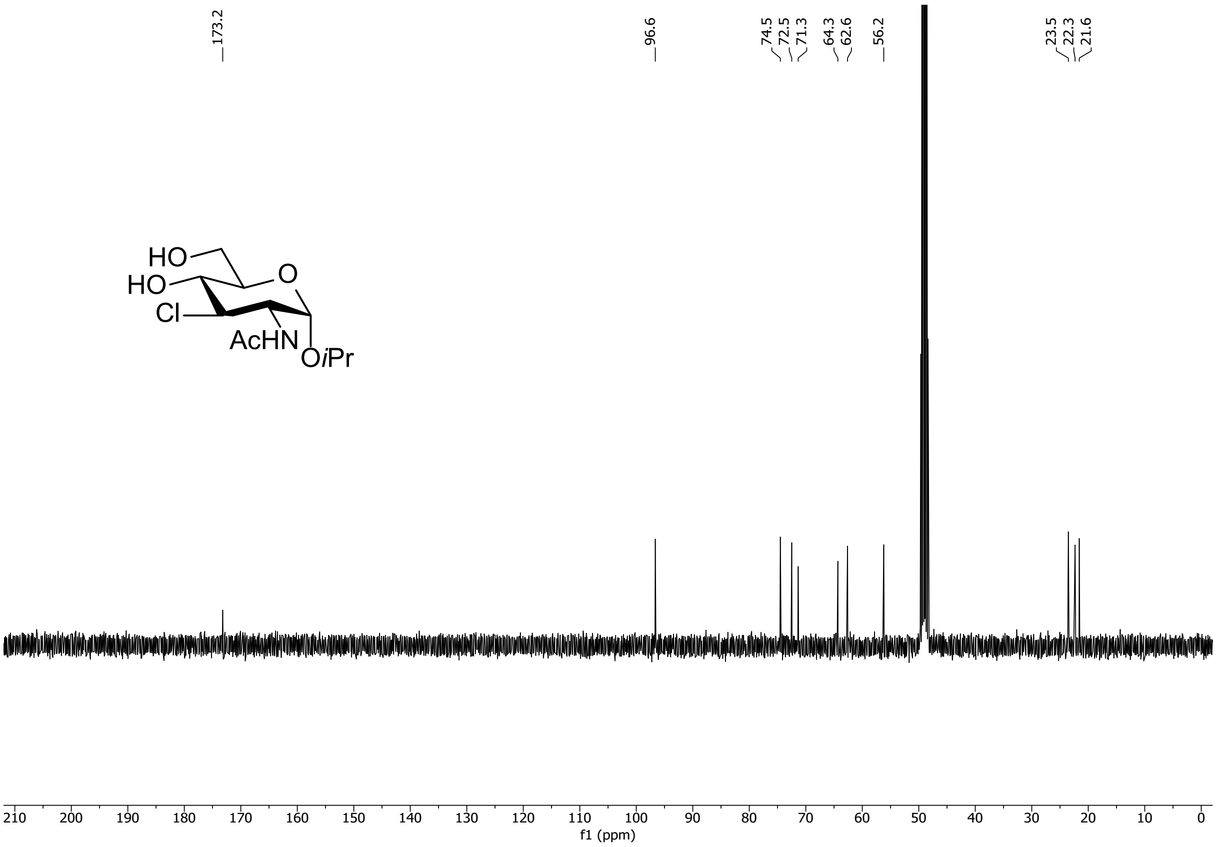

^{1}H-^{1}H COSY of compound **3a-equatorial**

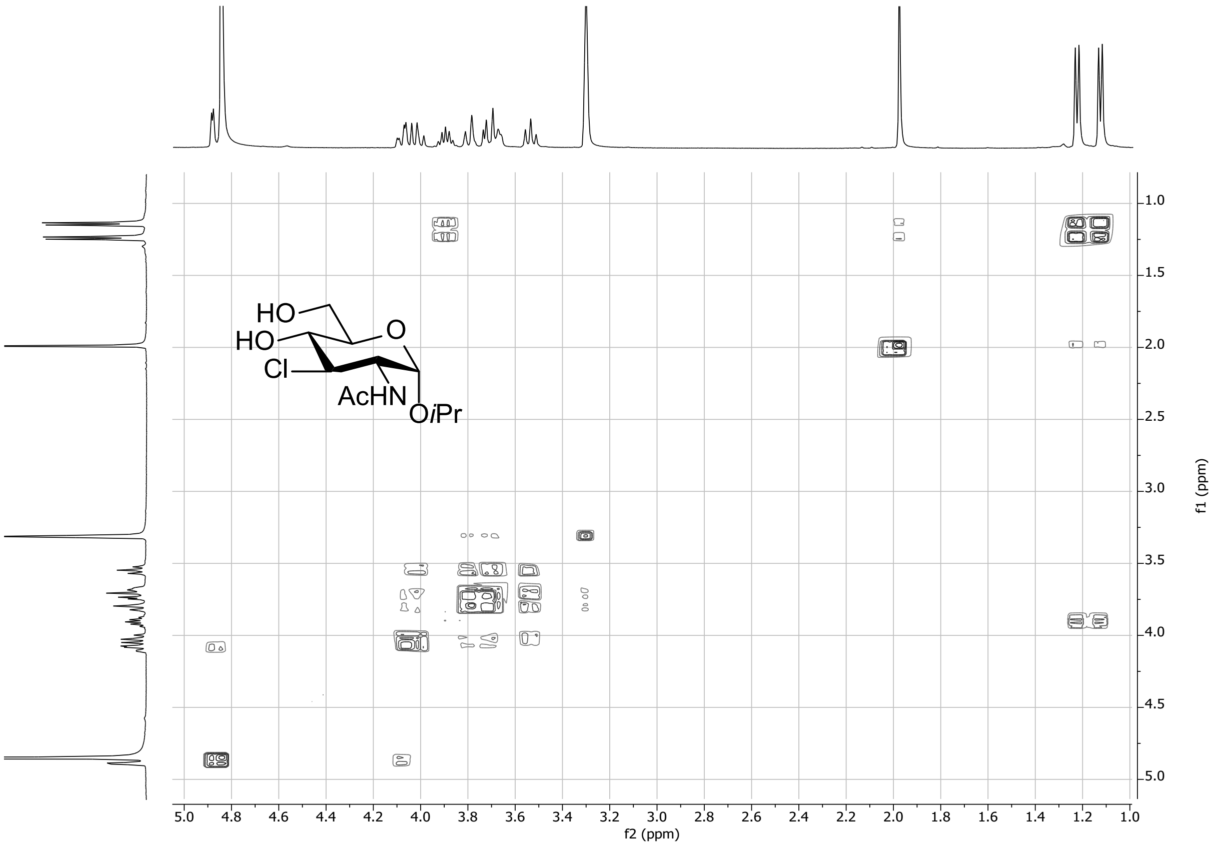

^{1}H-^{13}C HSQC of compound **3a-equatorial**

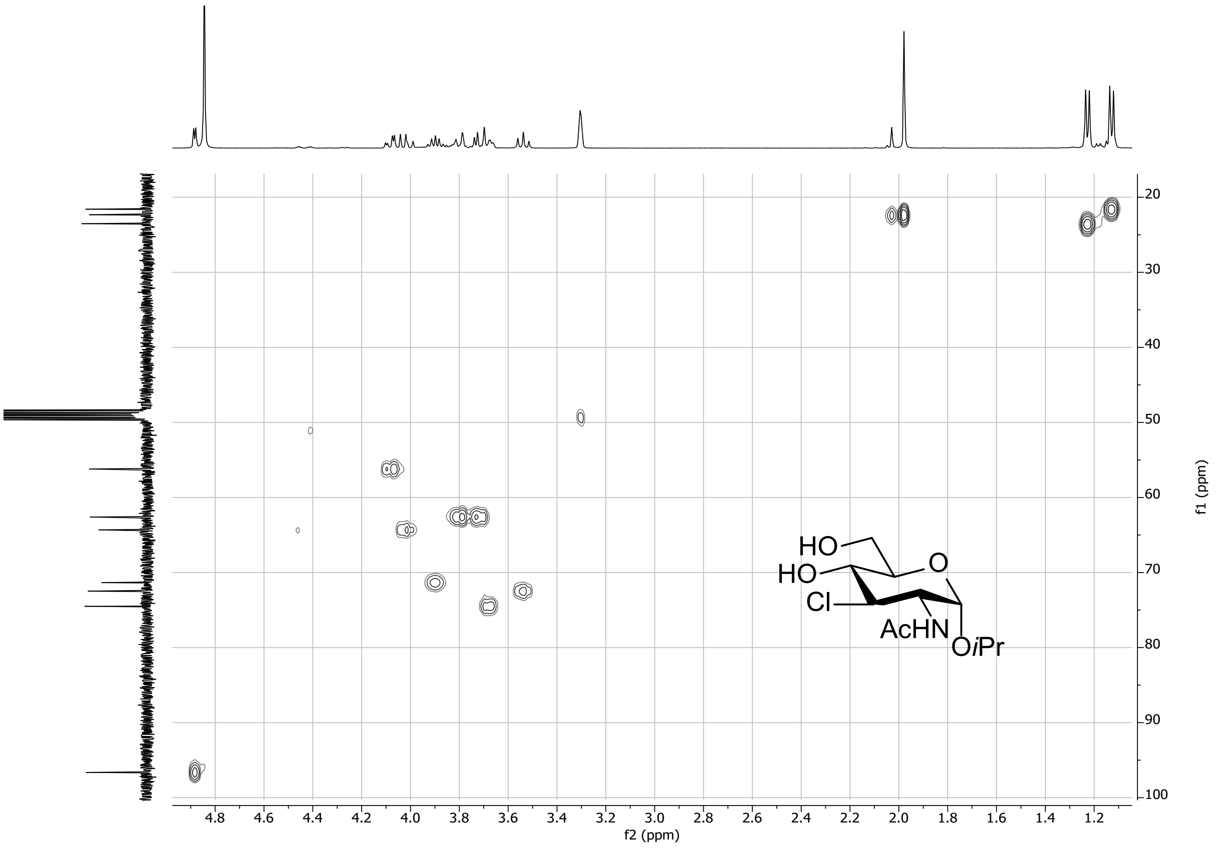

^{1}H NMR, 400 MHz, CD_3OD of compound **3a-axial**

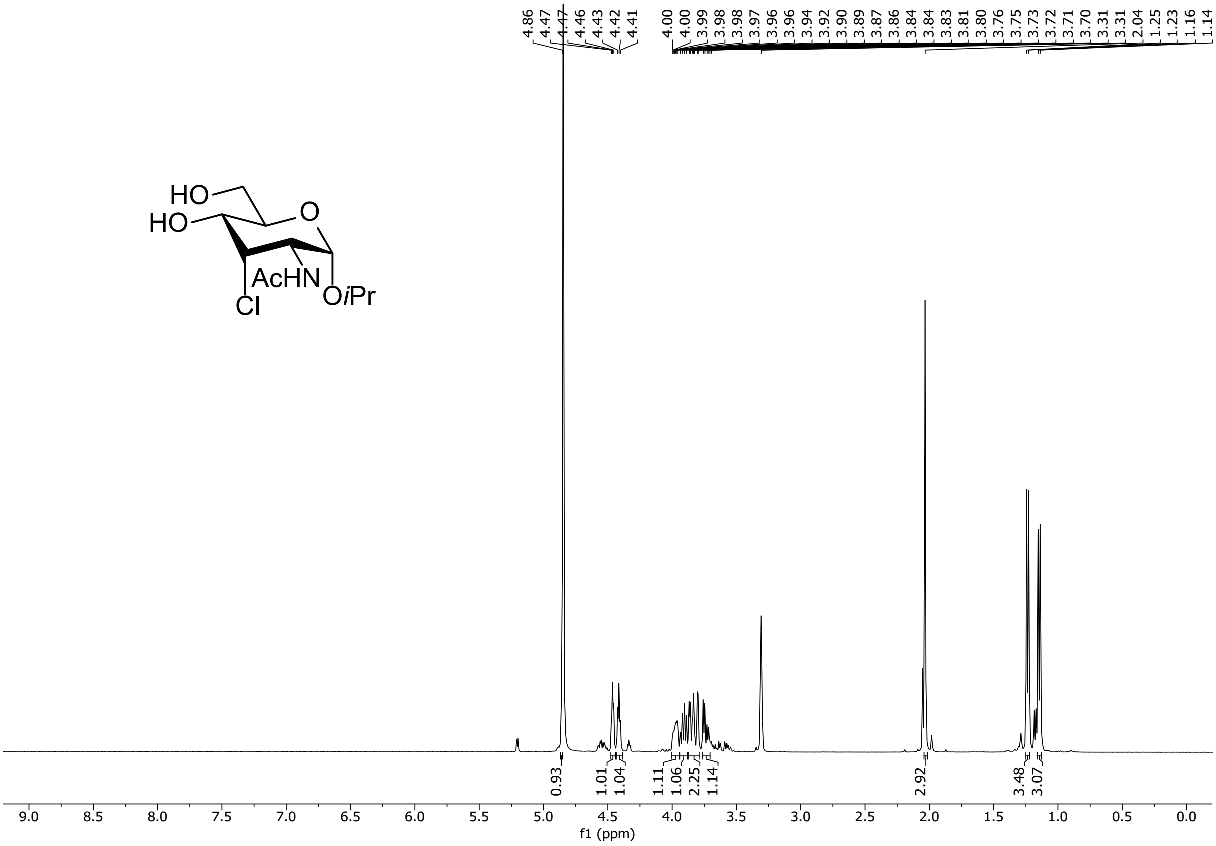

^{13}C NMR, 400 MHz, CD_3OD of compound **3a-axial**

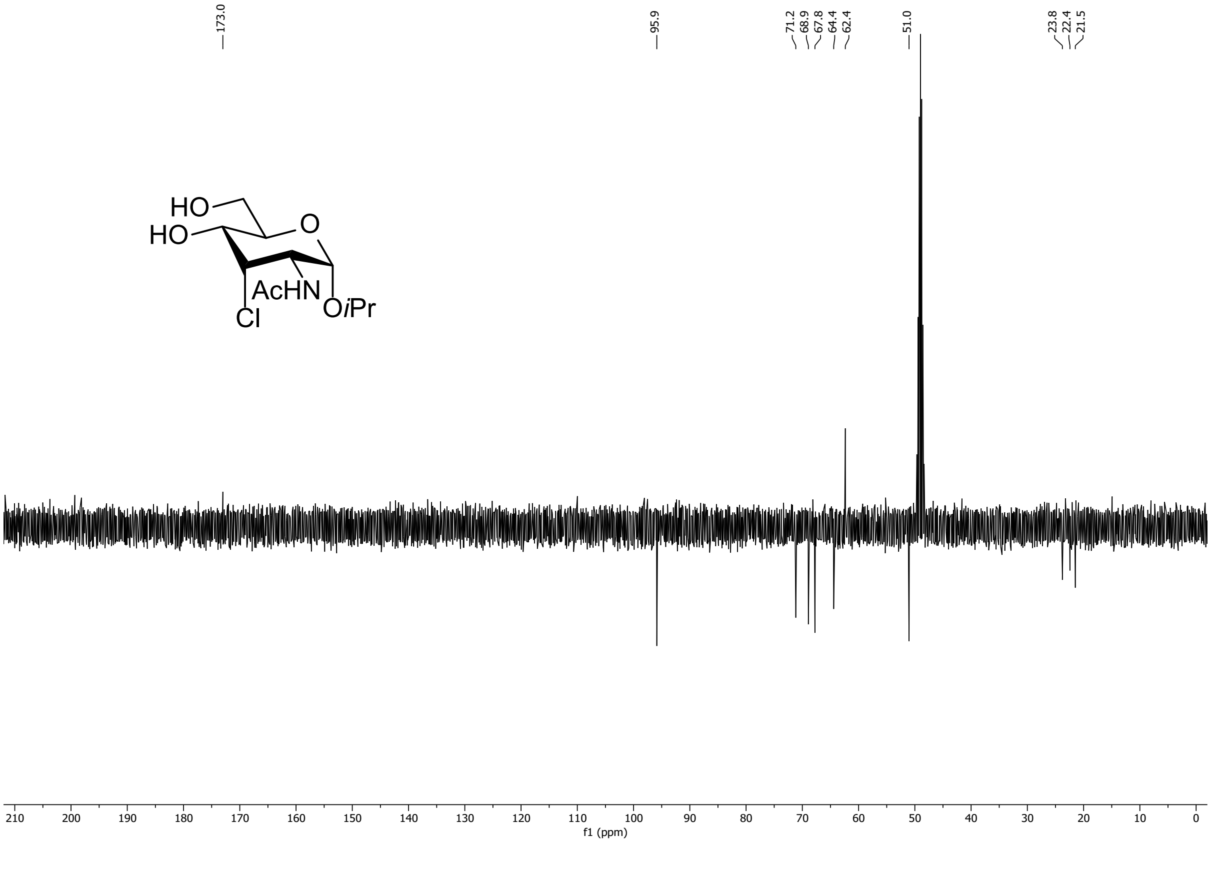

^{1}H-^{1}H COSY of compound **3a-axial**

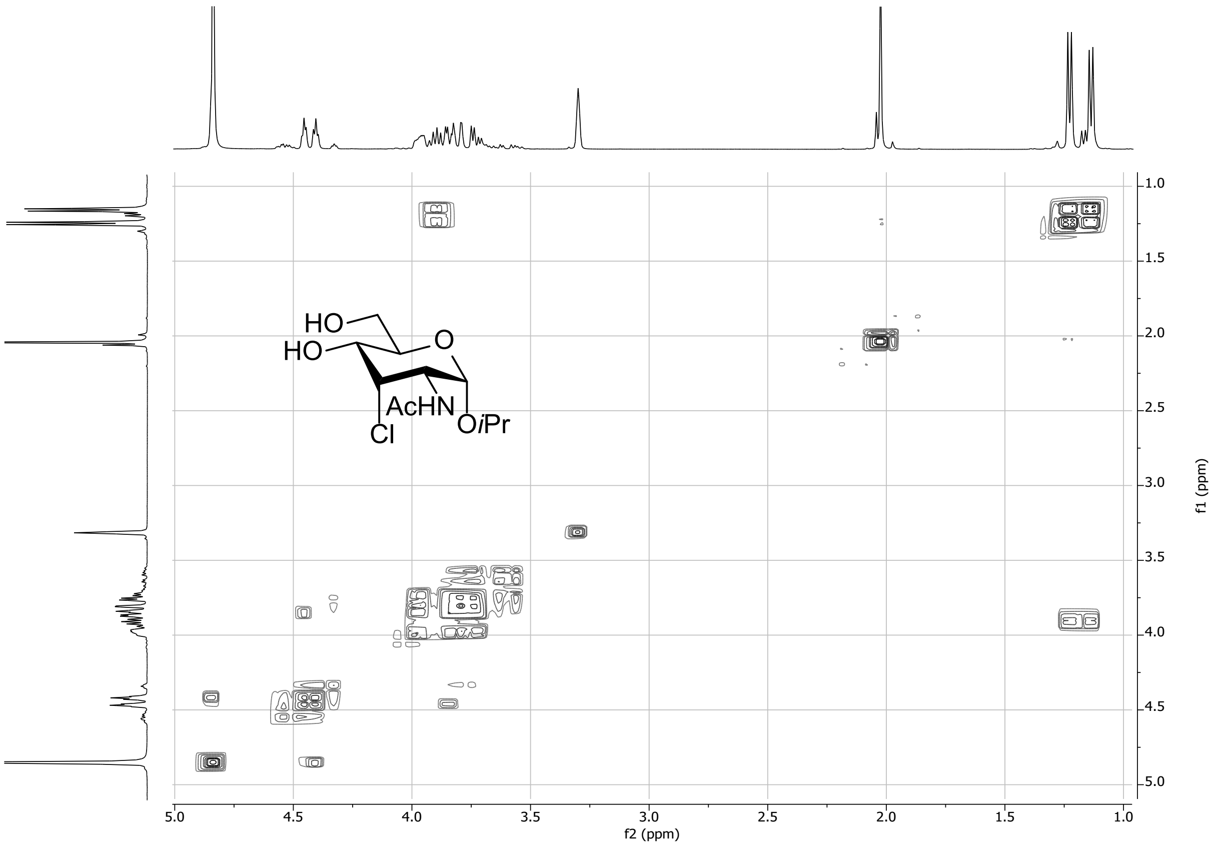

^{1}H-^{13}C HSQC of compound **3a-axial**

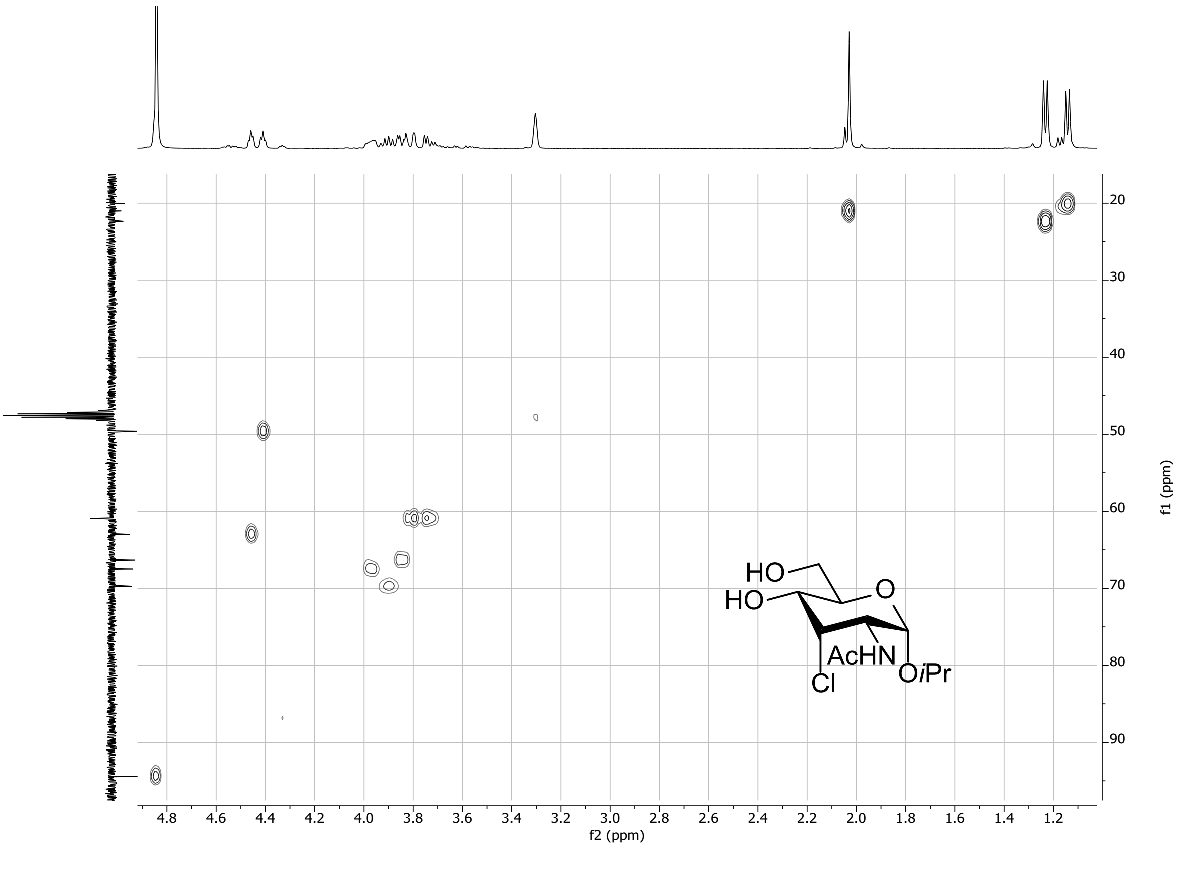

NMR spectra of α-Glc derivatives

Methyl 3-keto-α-D-glucopyranoside (1b)

^{1}H NMR, 400 MHz, CD_3OD of compound **1b**

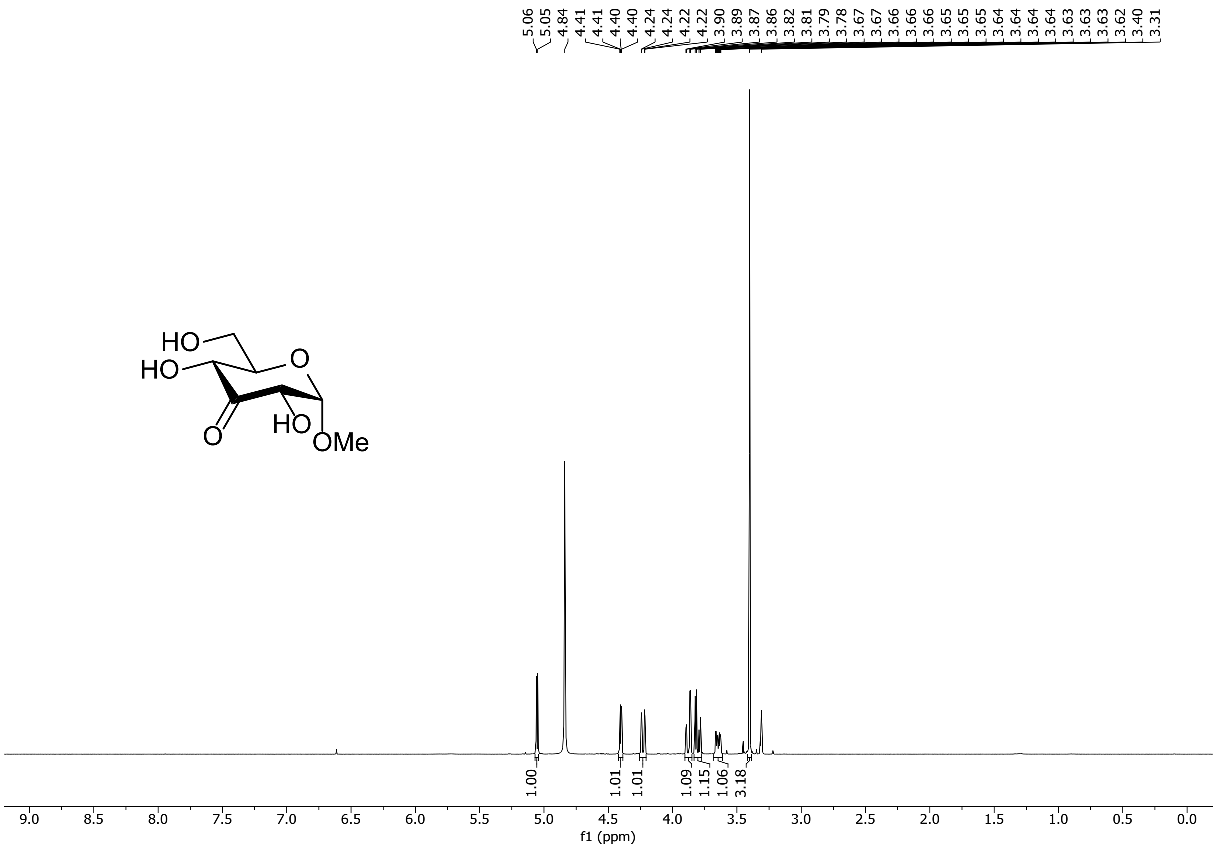

^{13}C NMR, 400 MHz, CD_3OD of compound **1b**

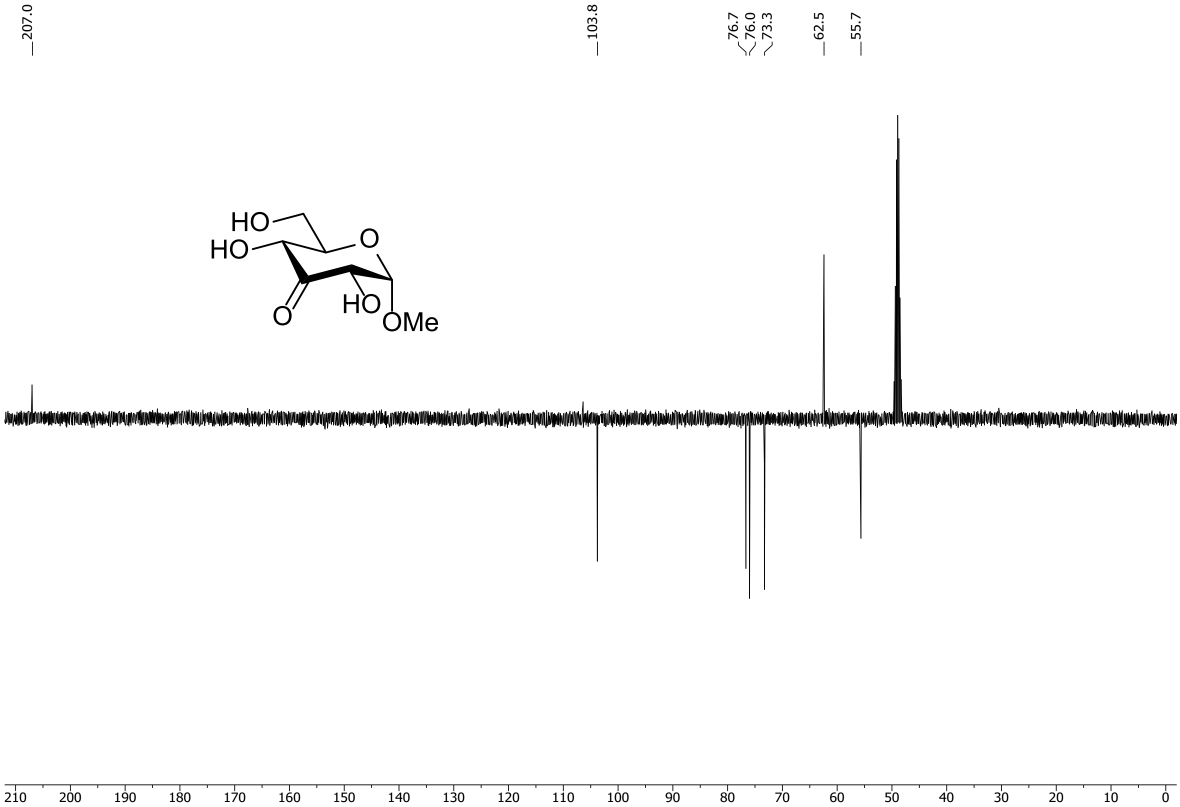

^{1}H-^{1}H COSY of compound **1b**

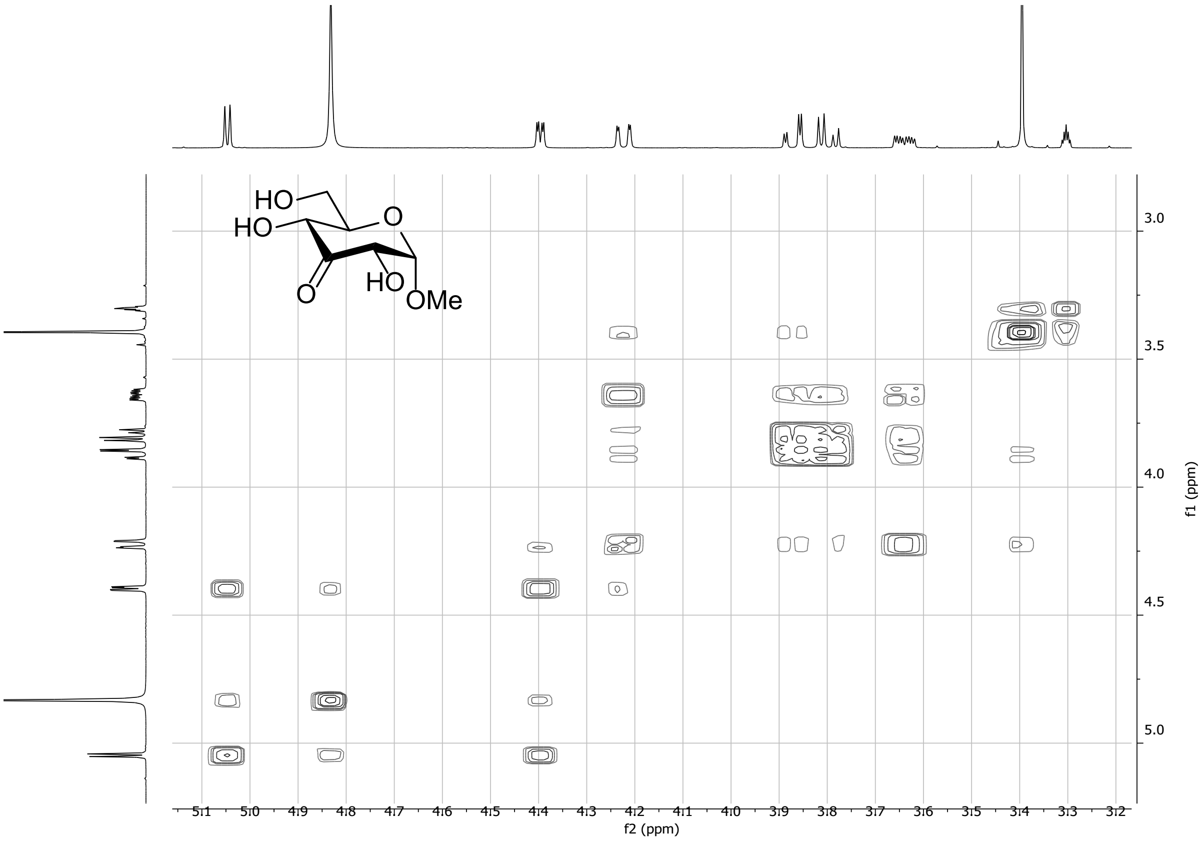

H-^{13}C HSQC of compound **1b**

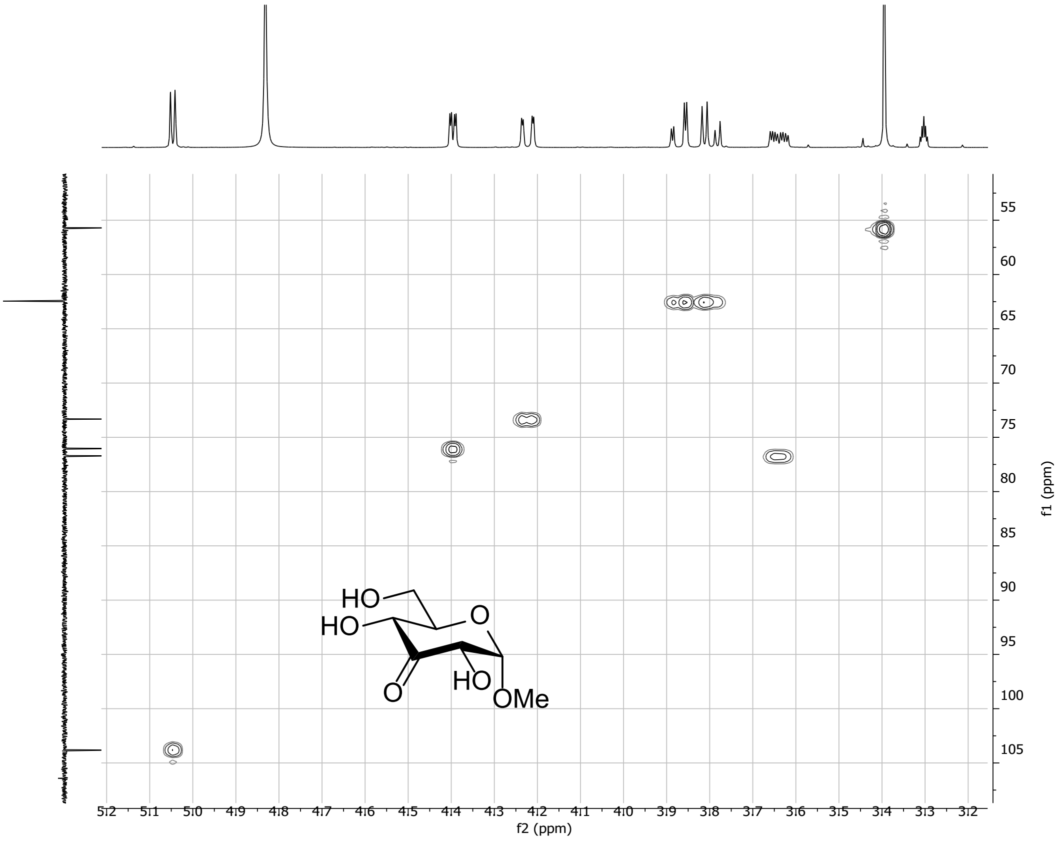

Methyl-3-(trityl)hydrazone-α-D-glucopyranoside (2b)

Mixture of *E* and *Z*: ratio ≈ 0.33:1

^{1}H NMR, 400 MHz, CD$_3$OD of compound **2b**

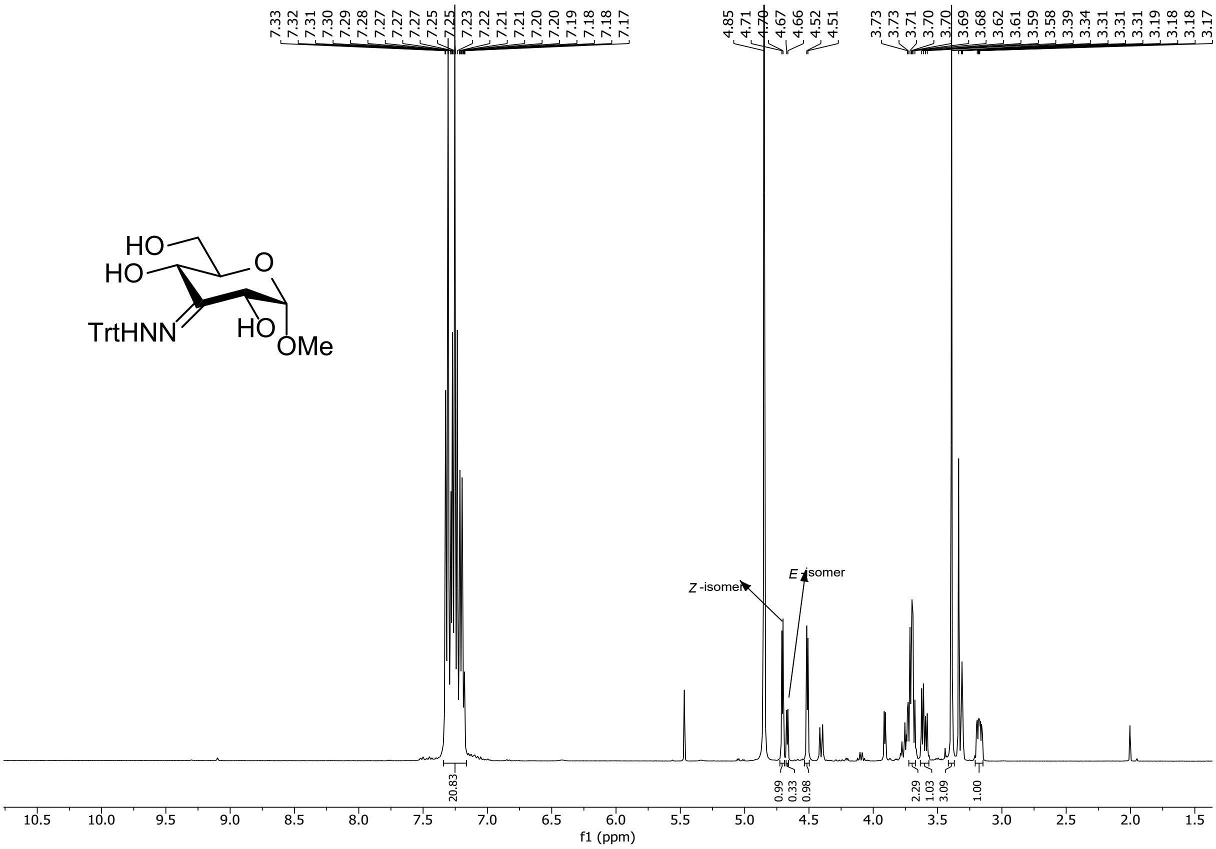

^{13}C NMR, 400 MHz, CD$_3$OD of compound **2b**

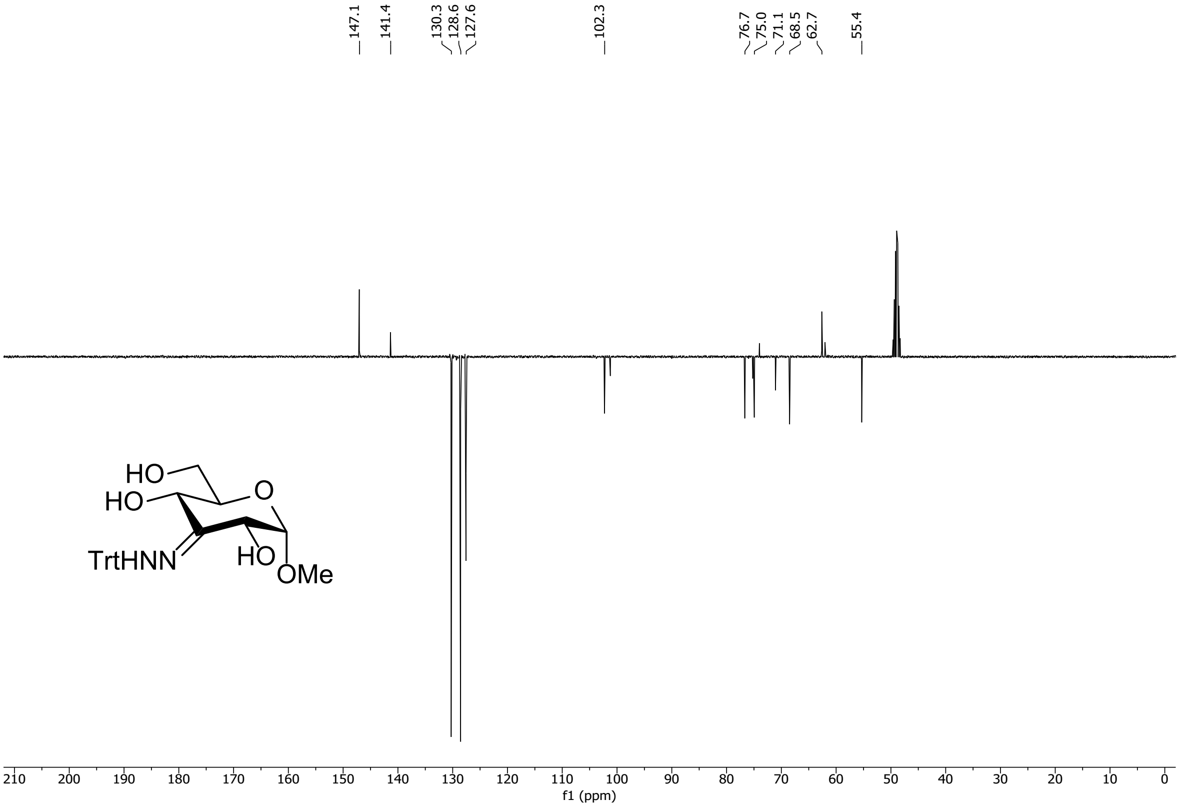

^{1}H-^{1}H COSY of compound **2b**

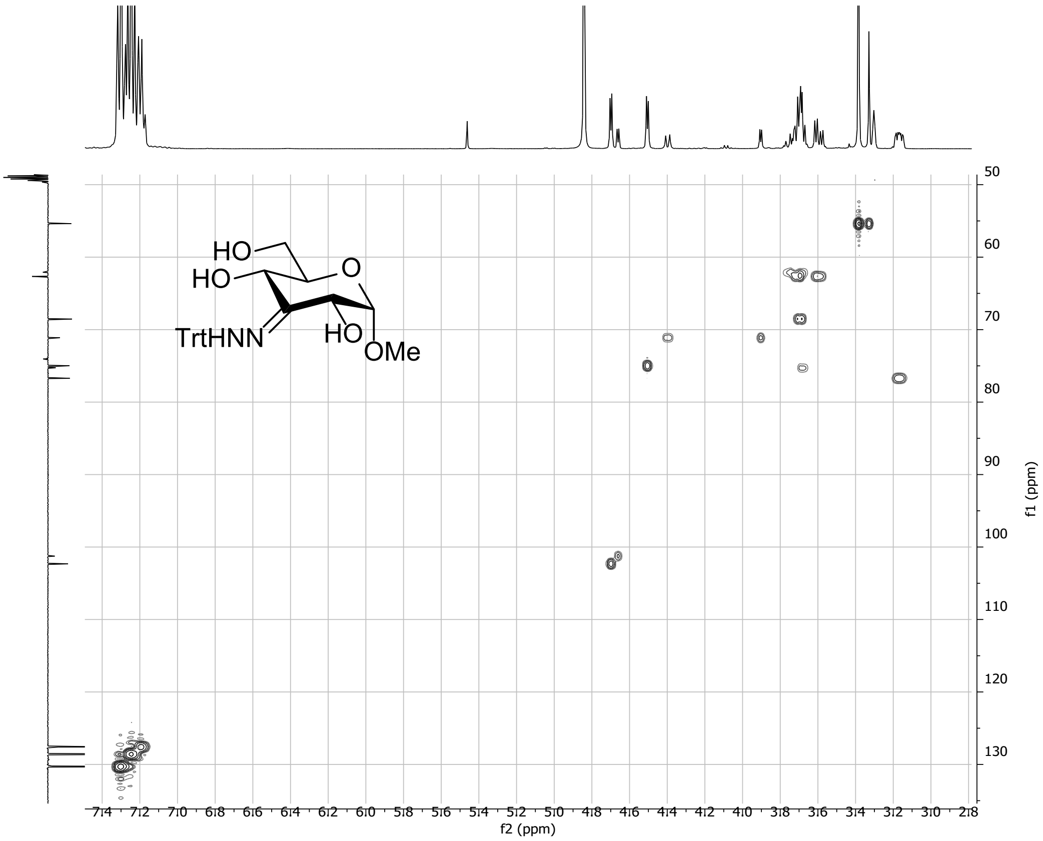

H-^{13}C HSQC of compound **2b**

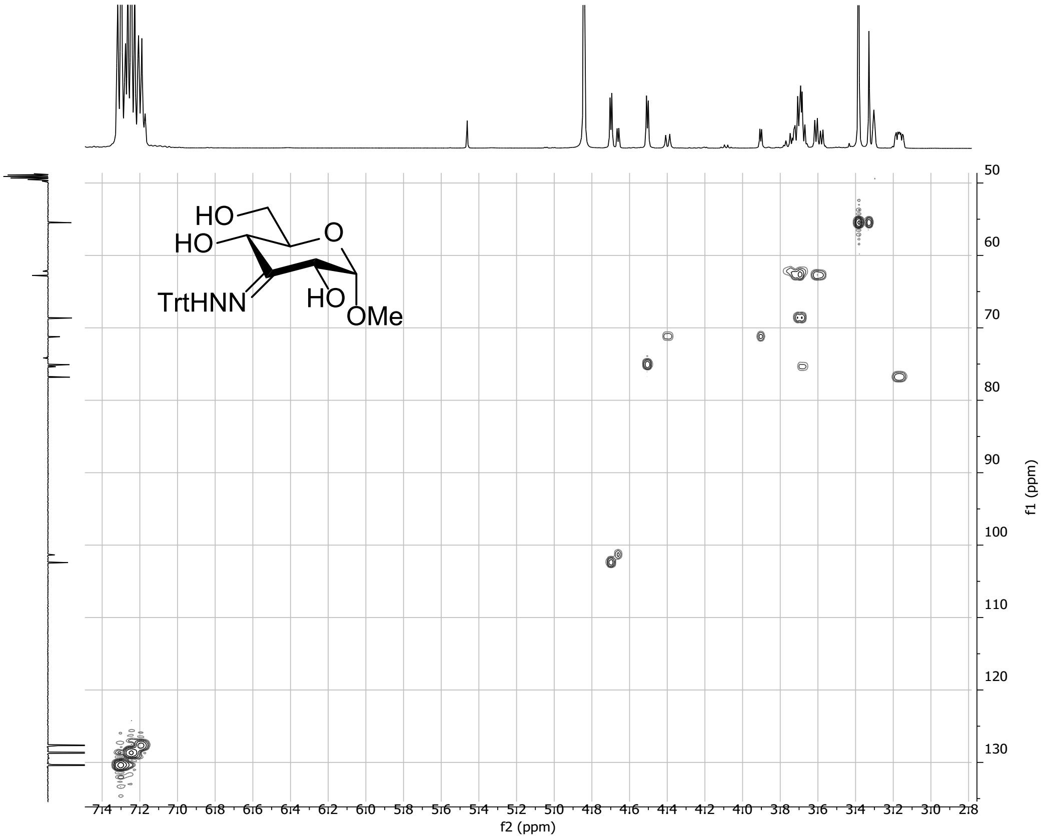

Methyl 3-chloro-3-deoxy-α-D-allo/glucopyranoside (3b)

Mixture of equatorial and axial, the chlorination is performed with t-BuSH at 60℃, 3-equatorial : 3-axial ≈ 1:3.5

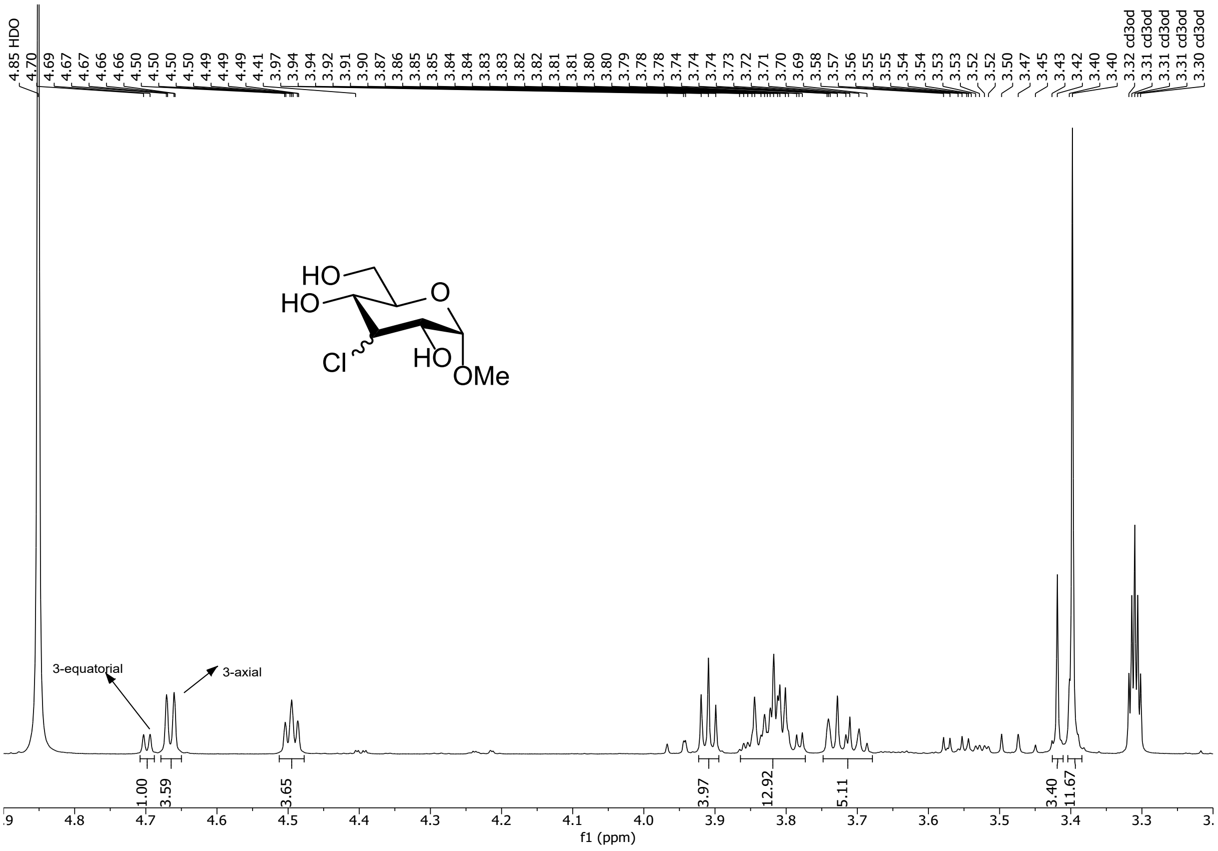

The chlorination is performed with t-BuSH at 50℃, 3-equatorial : 3-axial ≈ 1:3.3

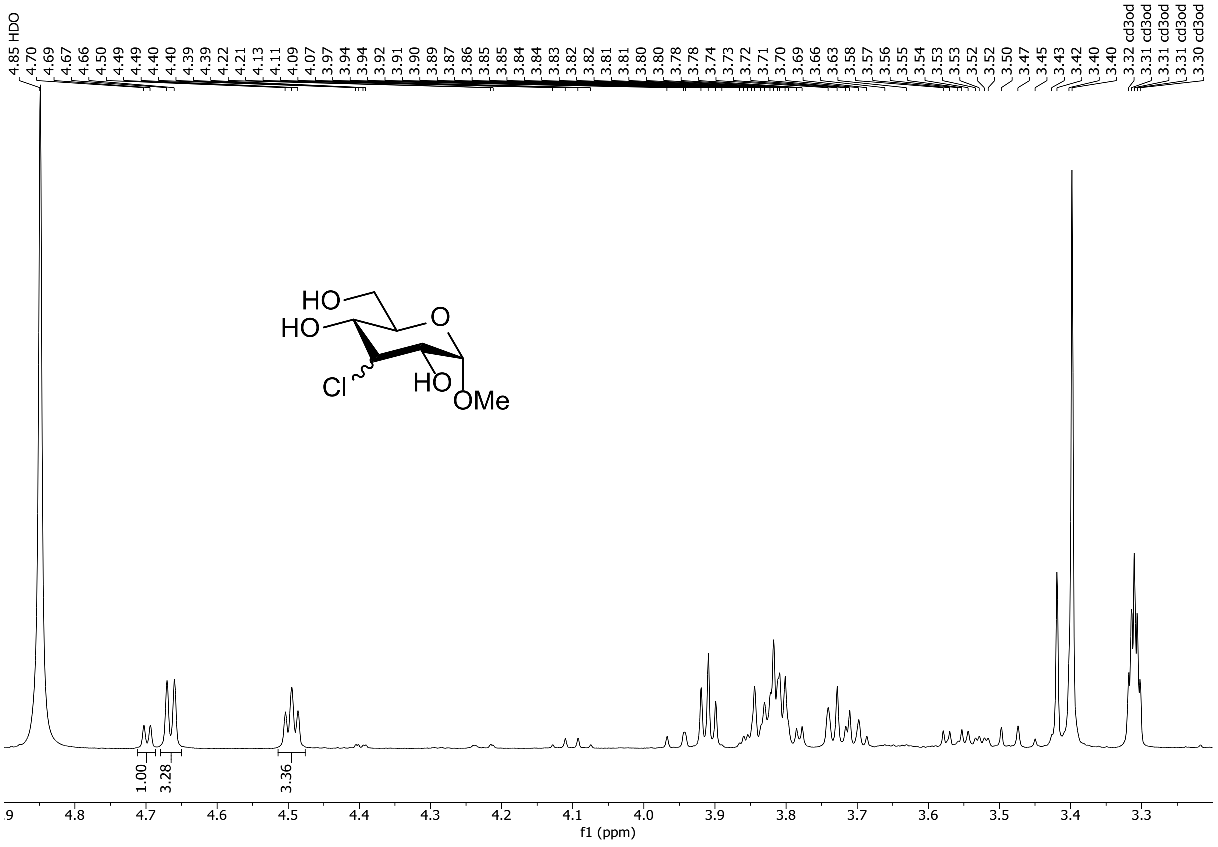

The chlorination is performed with t-BuSH at 40℃, 3-equatorial : 3-axial ≈ 1:2.2

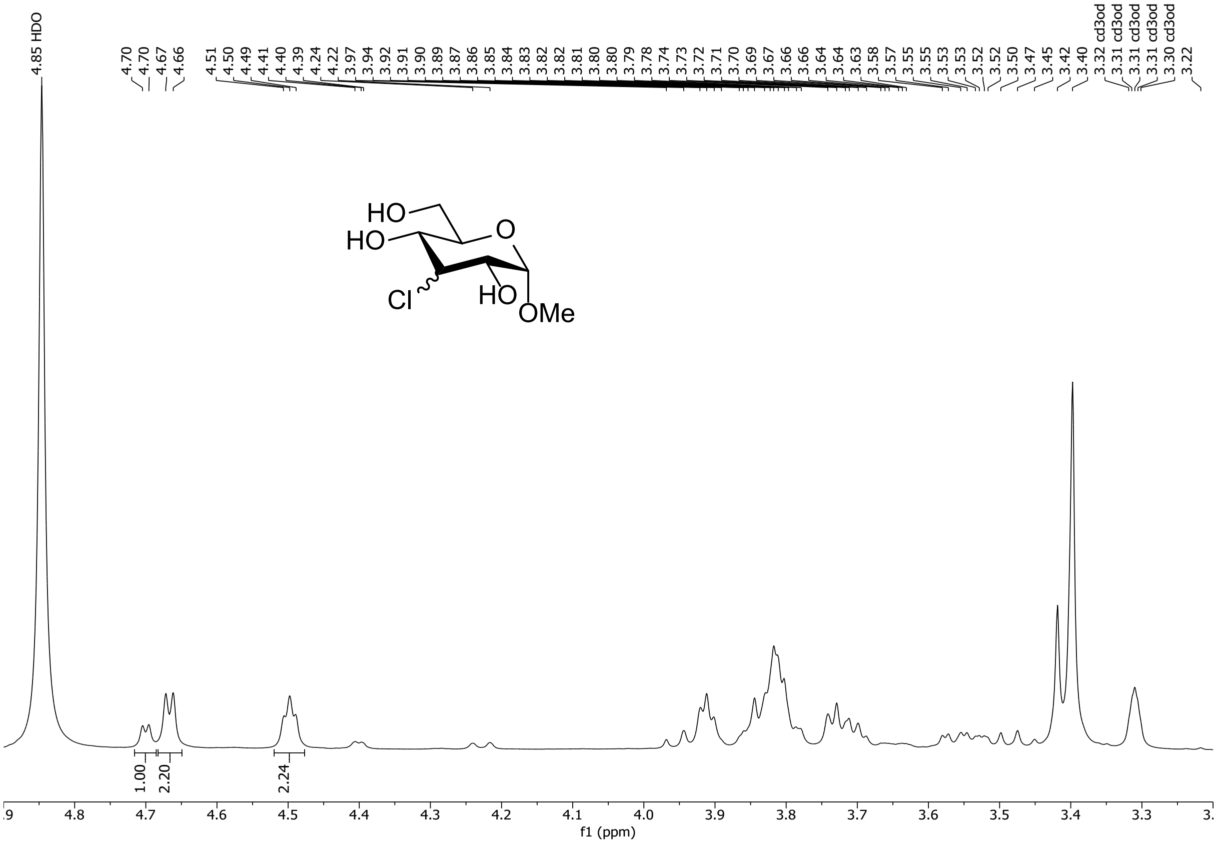

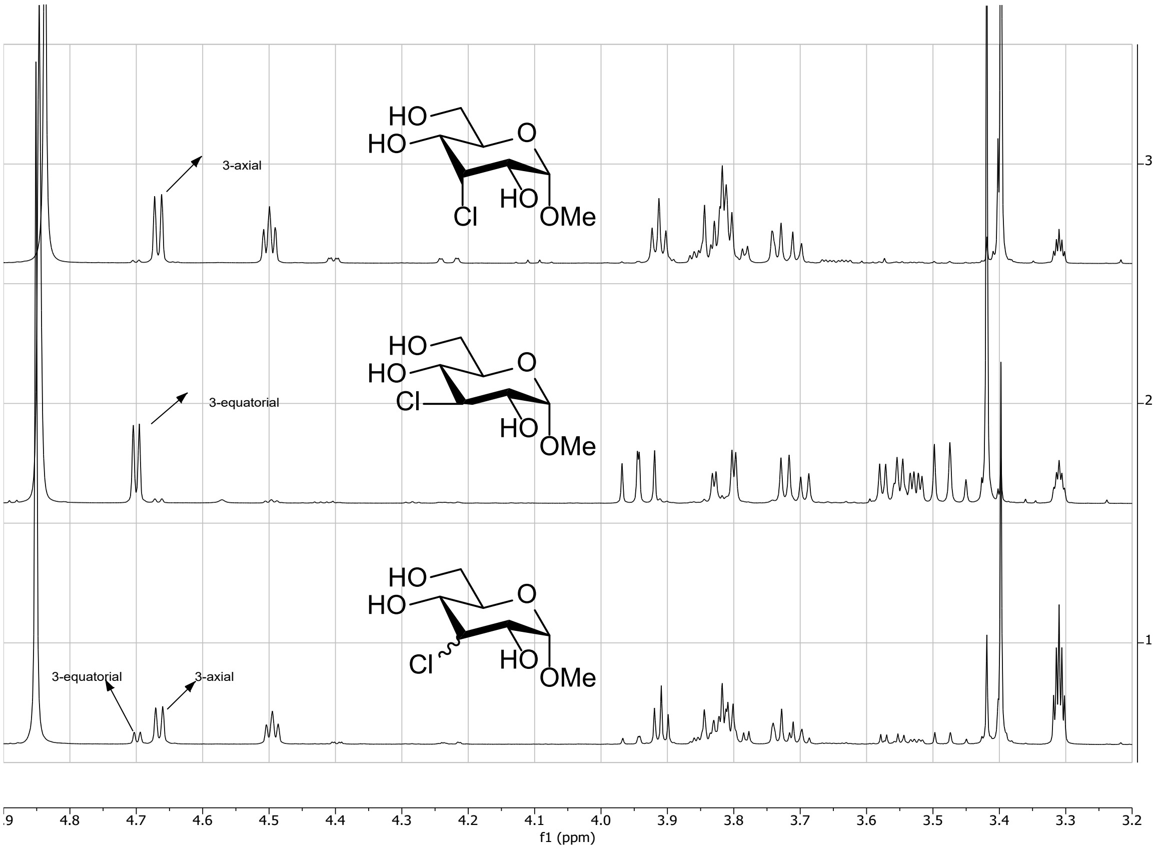

^{1}H NMR, 400 MHz, CD_3OD of compound **3b:** comparison between column purification (above) and without column purification (below).

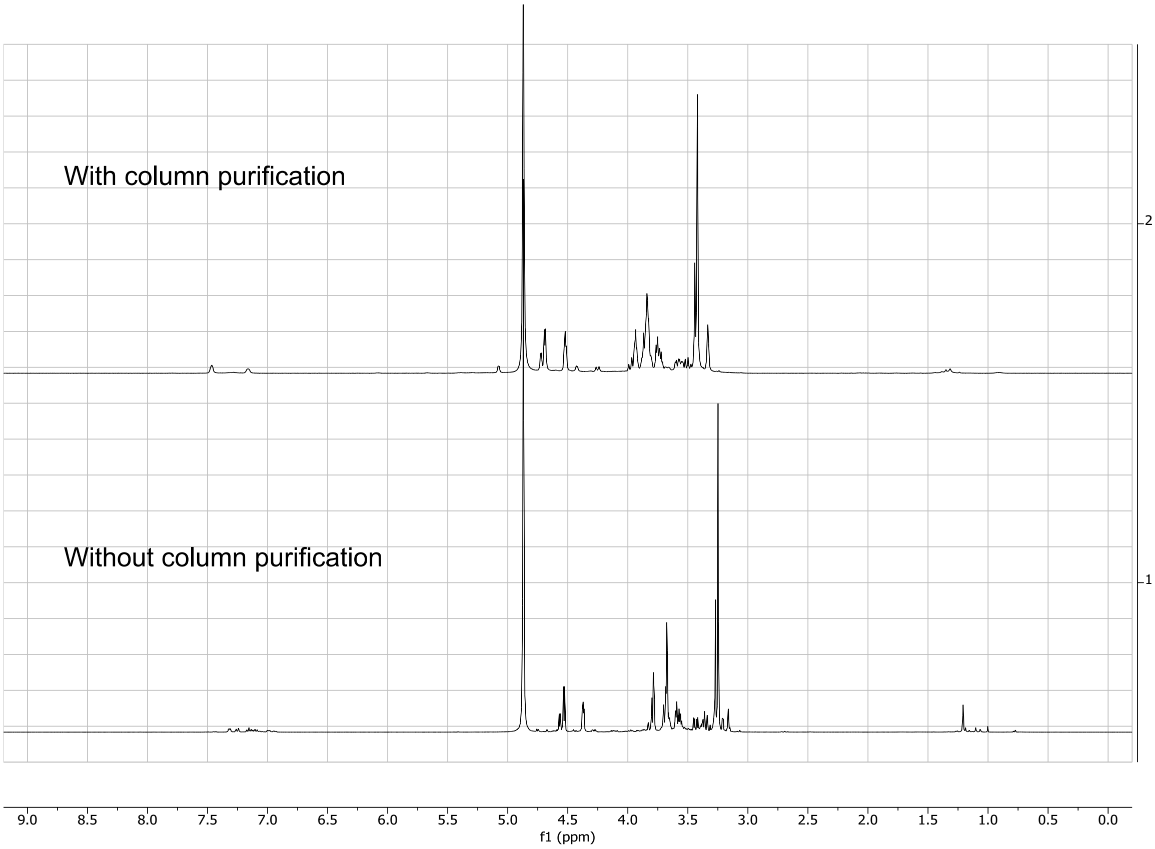

^{1}H NMR, 400 MHz, CD_3OD of compound **3b: equatorial**

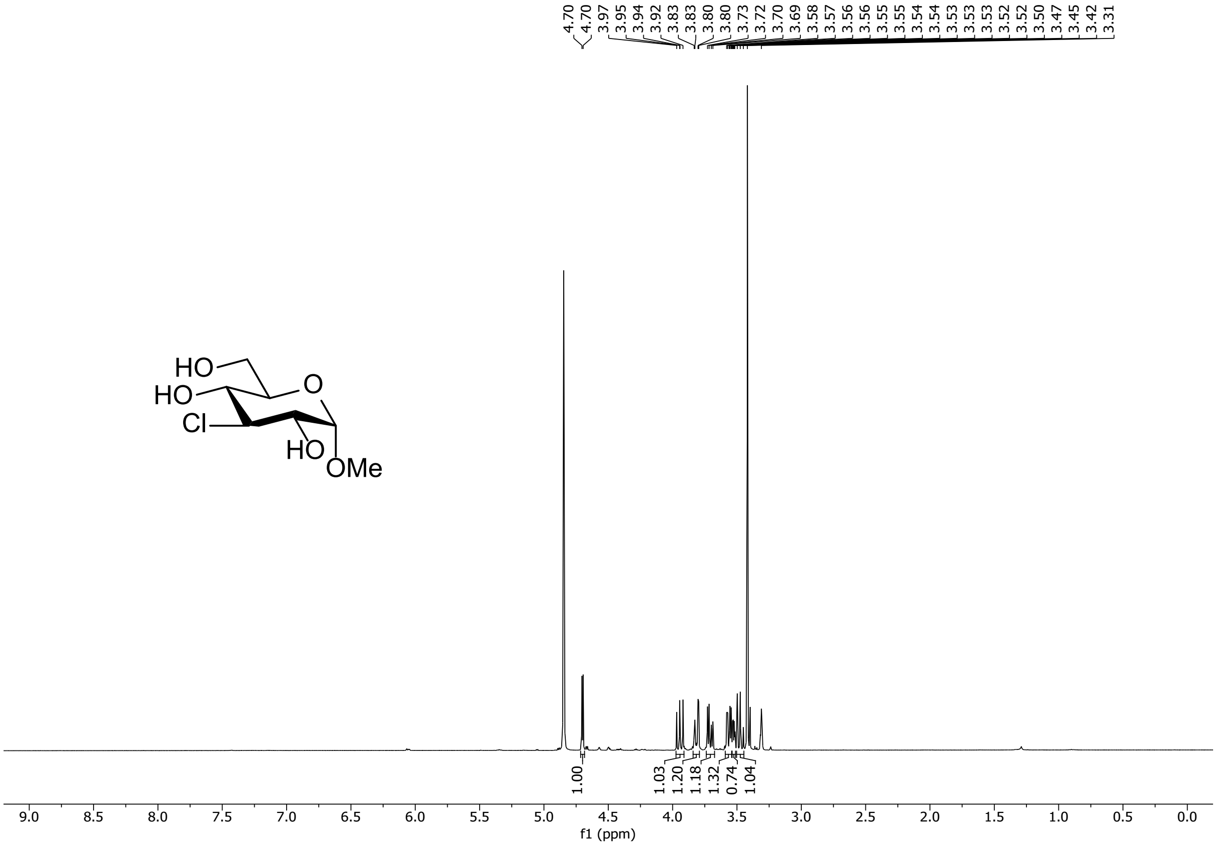

^{13}C NMR, 400 MHz, CD_3OD of compound **3b: equatorial**

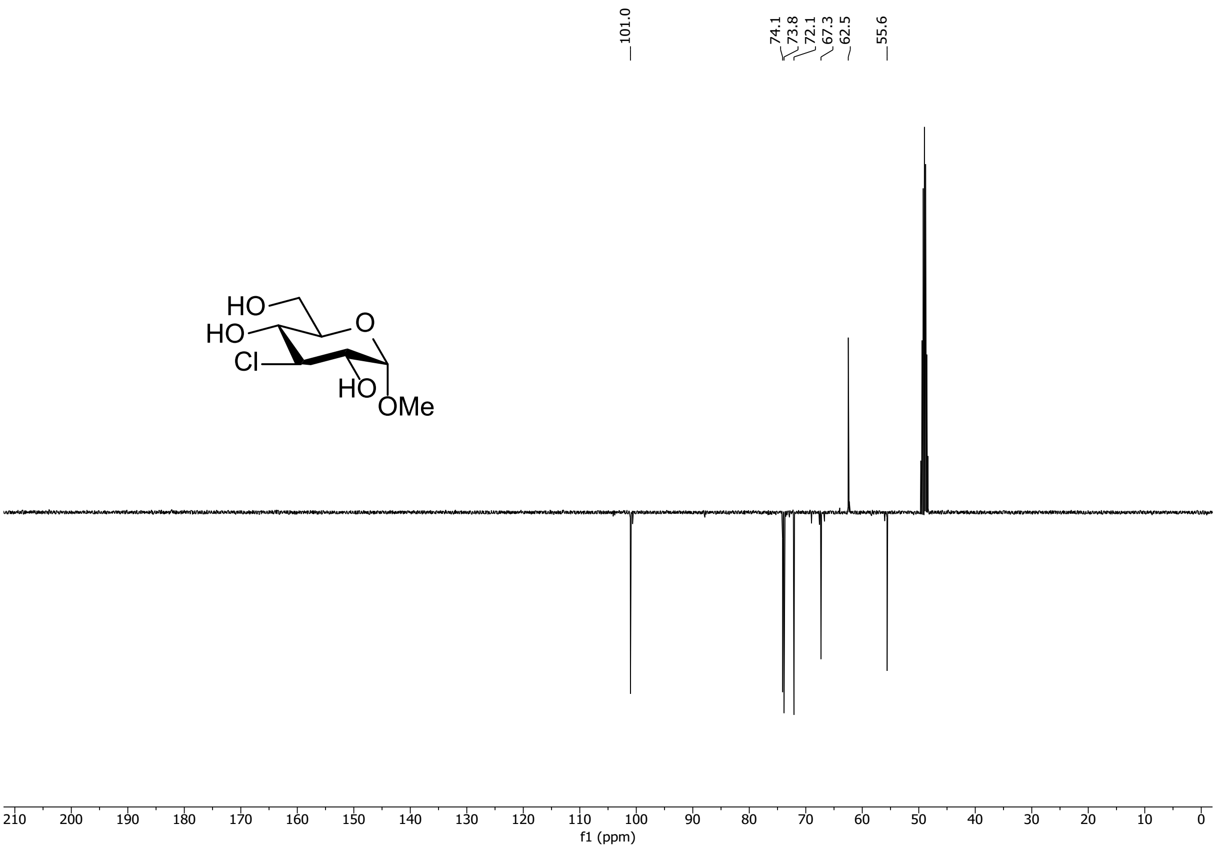

^{1}H-^{1}H COSY of compound **3b: equatorial**

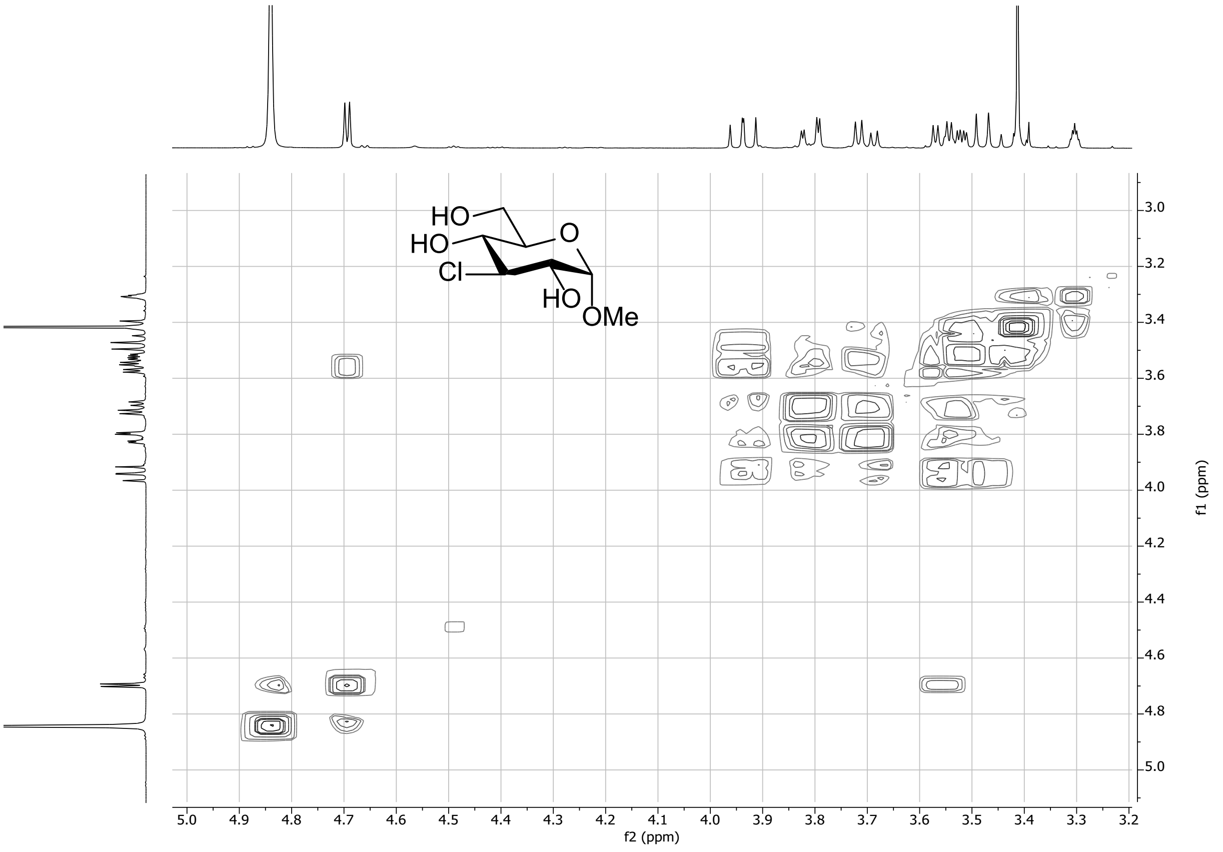

H-^{13}C HSQC of compound **3b: equatorial**

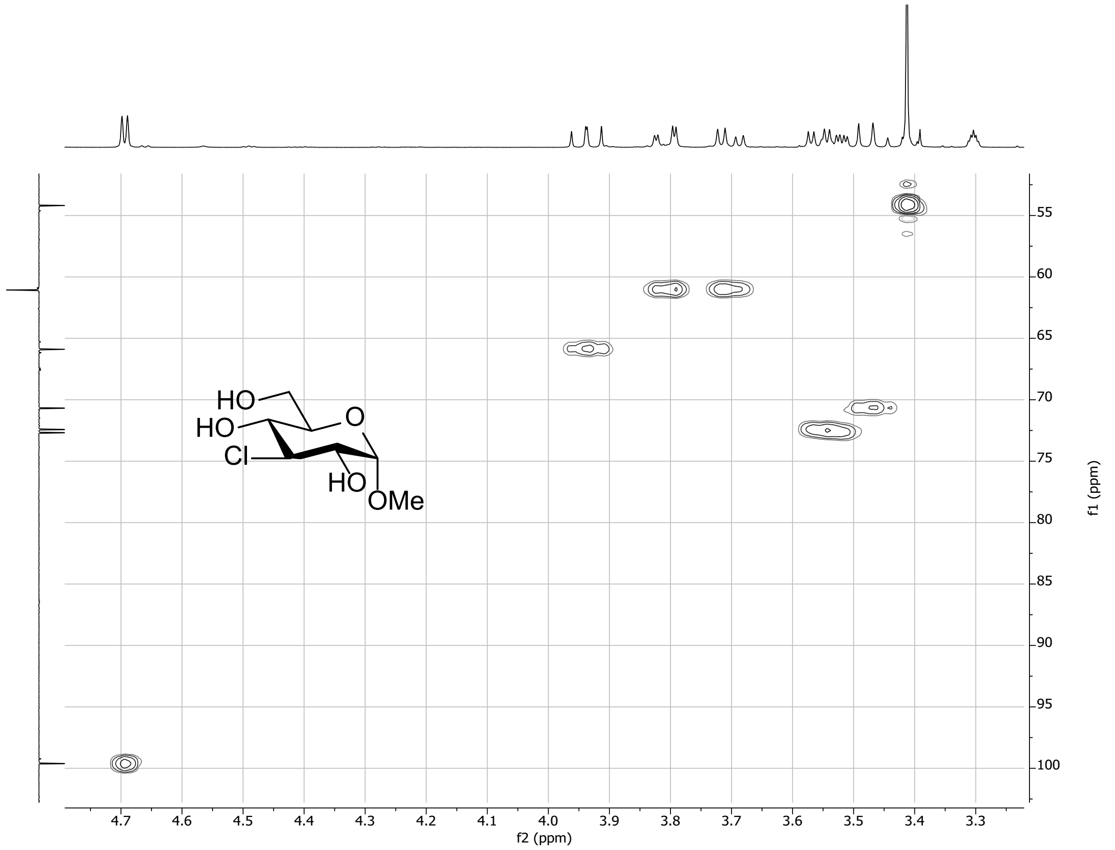

^{1}H NMR, 400 MHz, CD_3OD of compound **3b: axial**

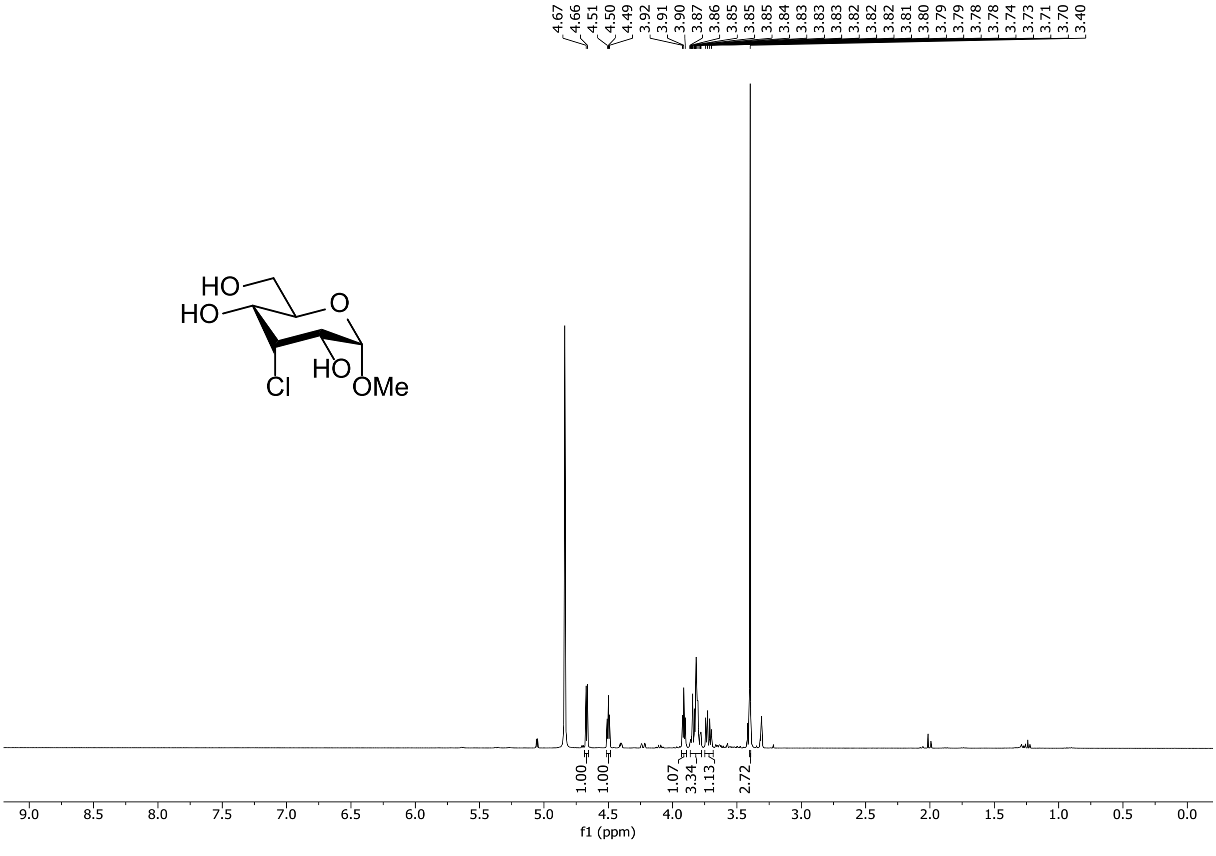

^{13}C NMR, 400 MHz, CD$_3$OD of compound **3b: axial**

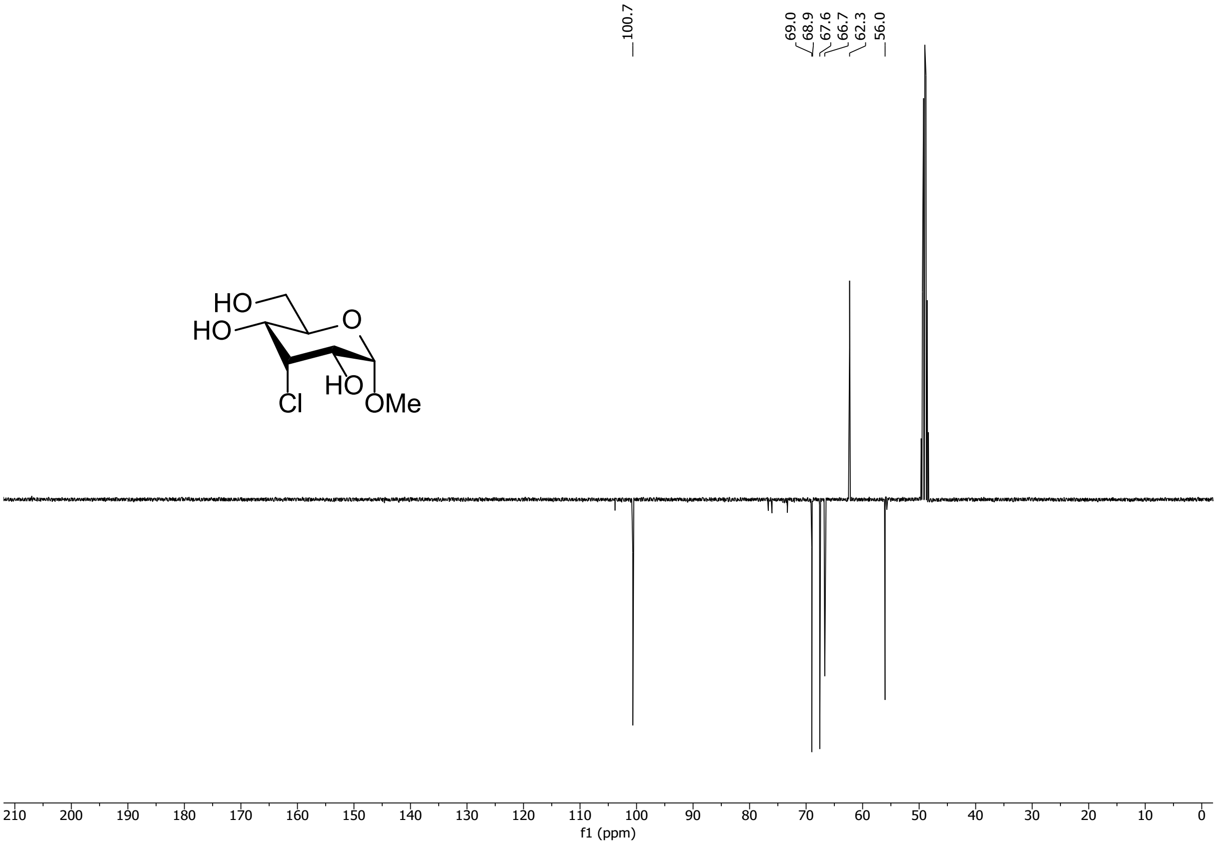

^{1}H-^{1}H COSY of compound **3b: axial**

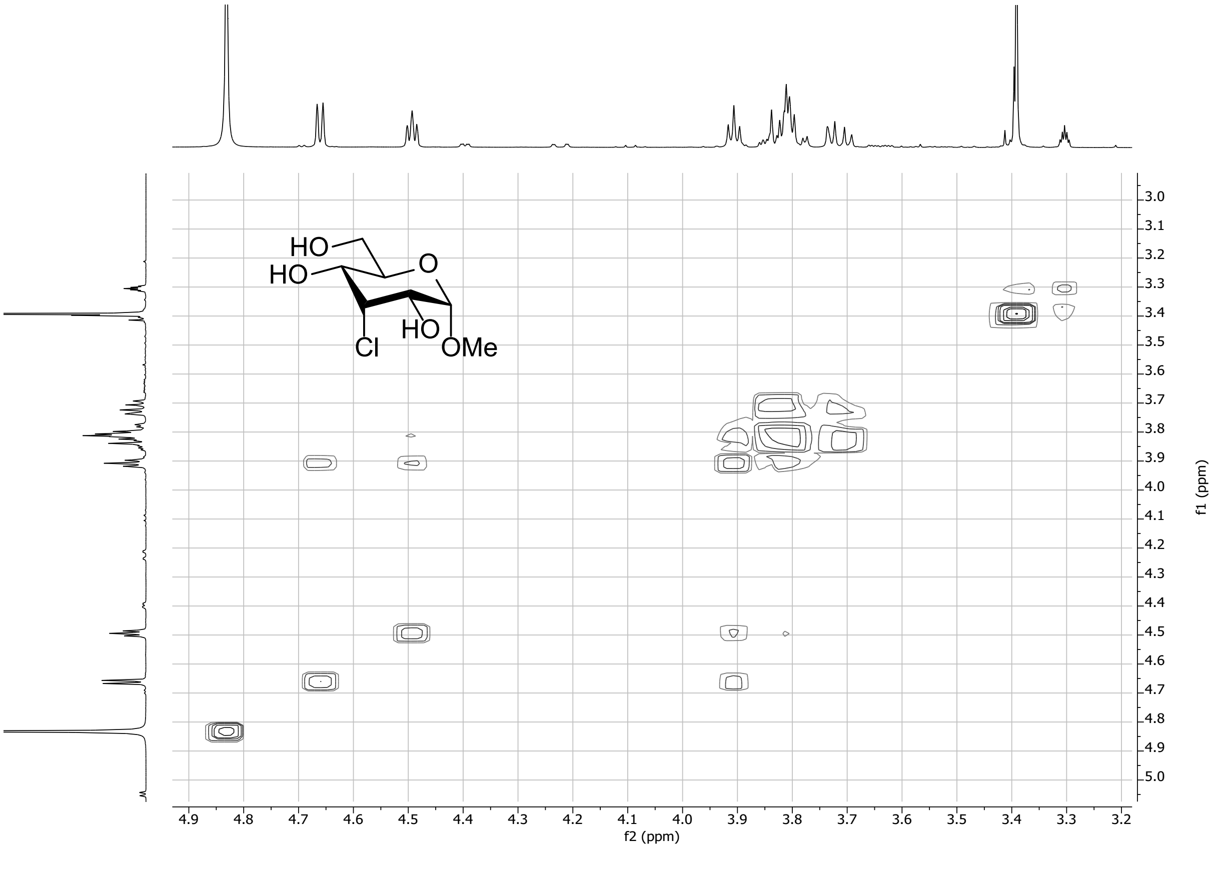

H-^{13}C HSQC of compound **3b: axial**

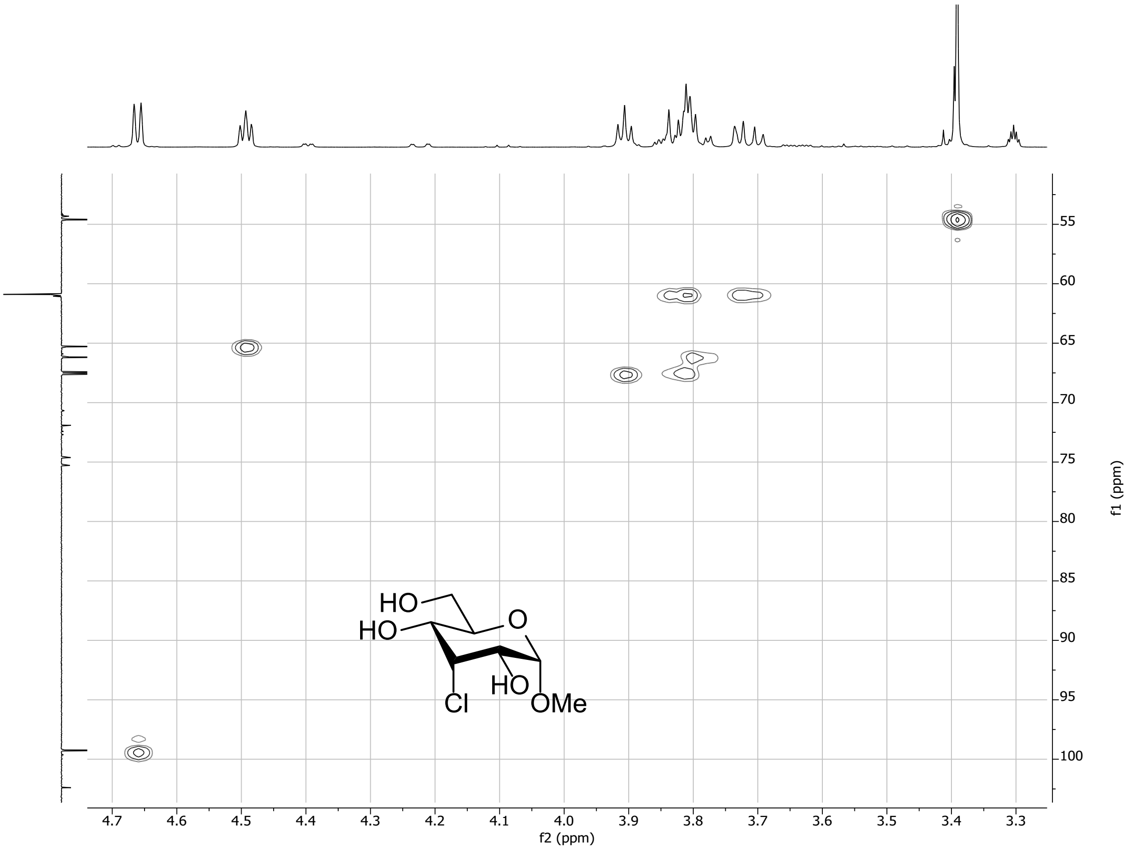

Methyl 3-S-ethyl-3-deoxy-α-D-allo/glucopyranoside (4)

^{1}H NMR, 400 MHz, CD_3OD of compound **4**

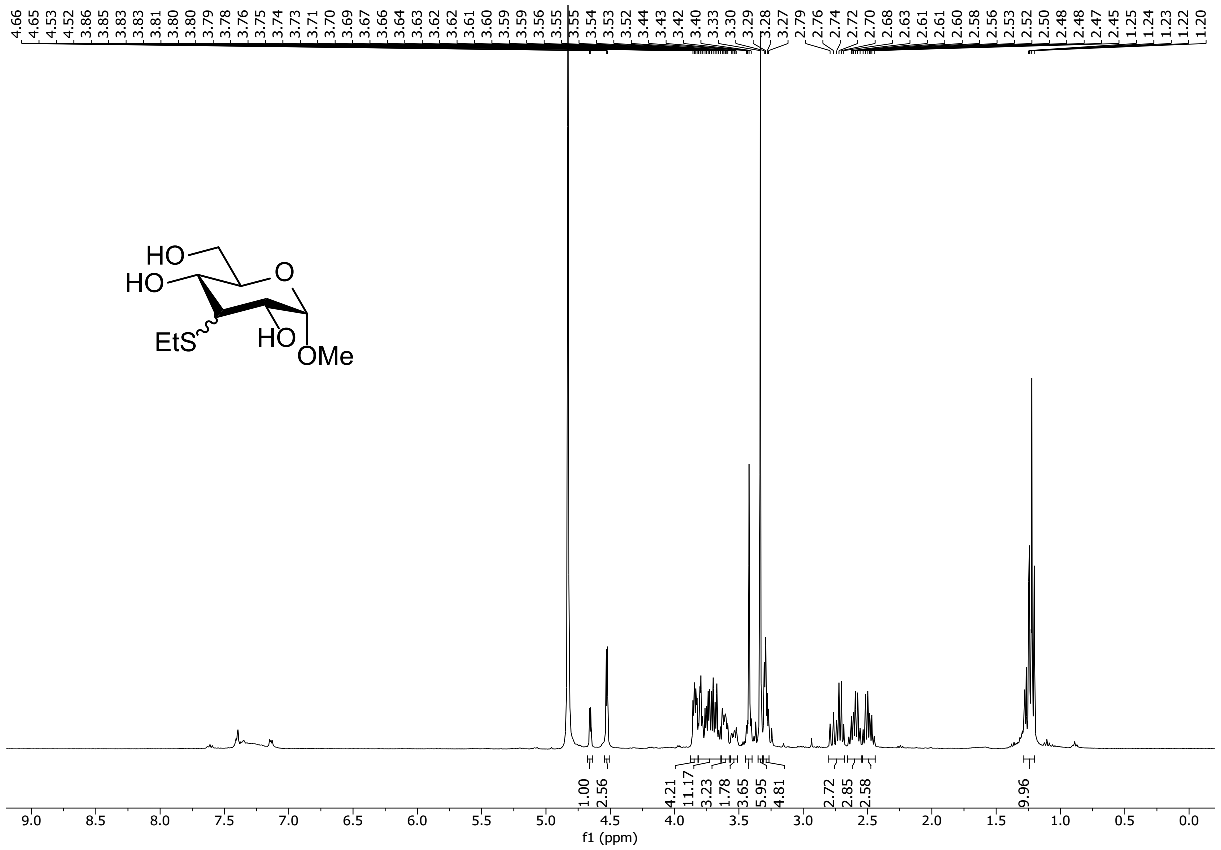

^{13}C NMR, 400 MHz, CD_3OD of compound **4**

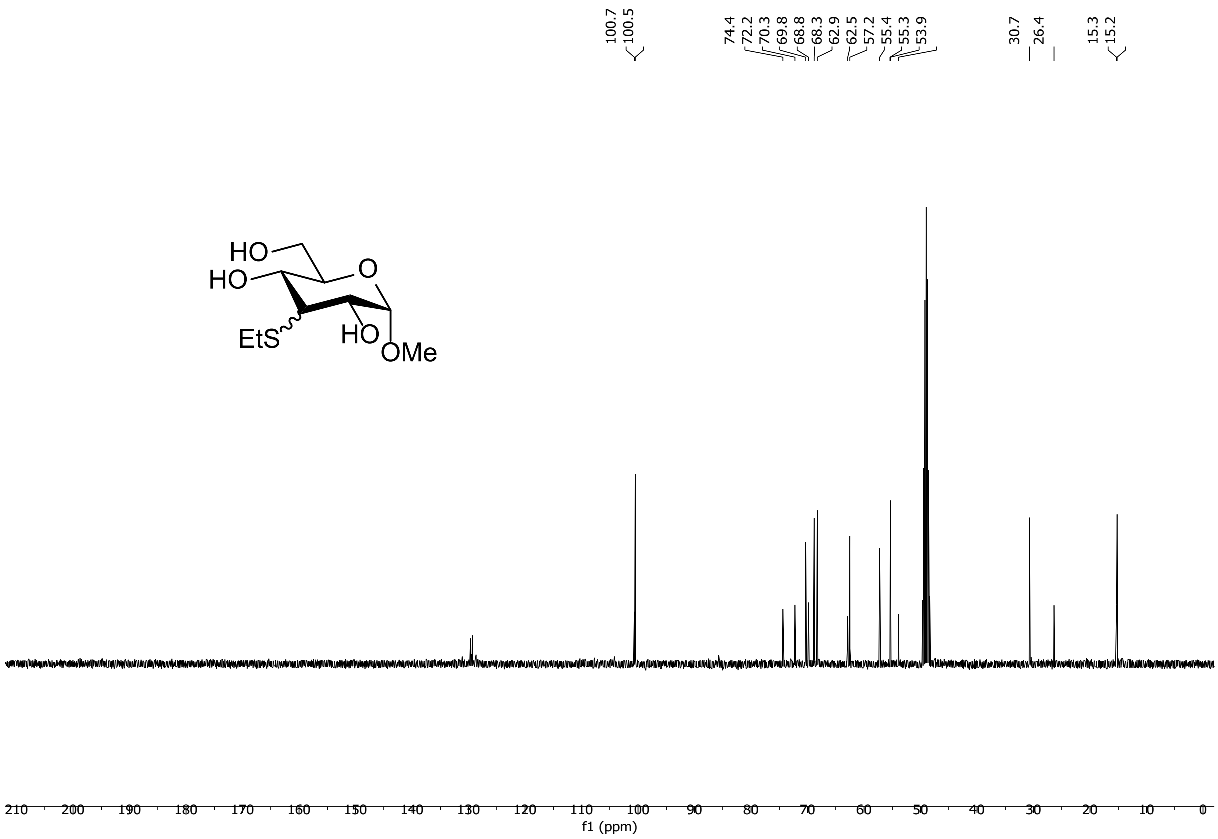

^{1}H-^{1}H COSY of compound **4**

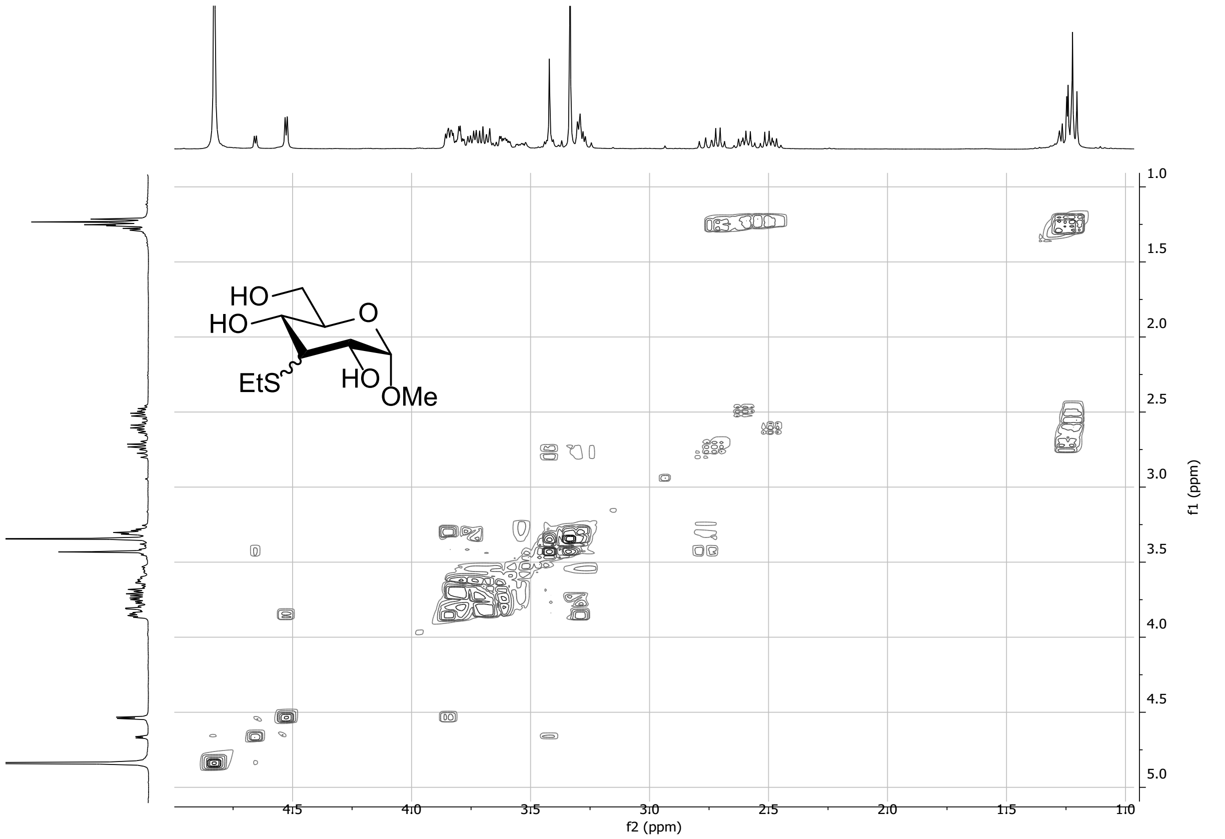

H-^{13}C HSQC of compound **4**

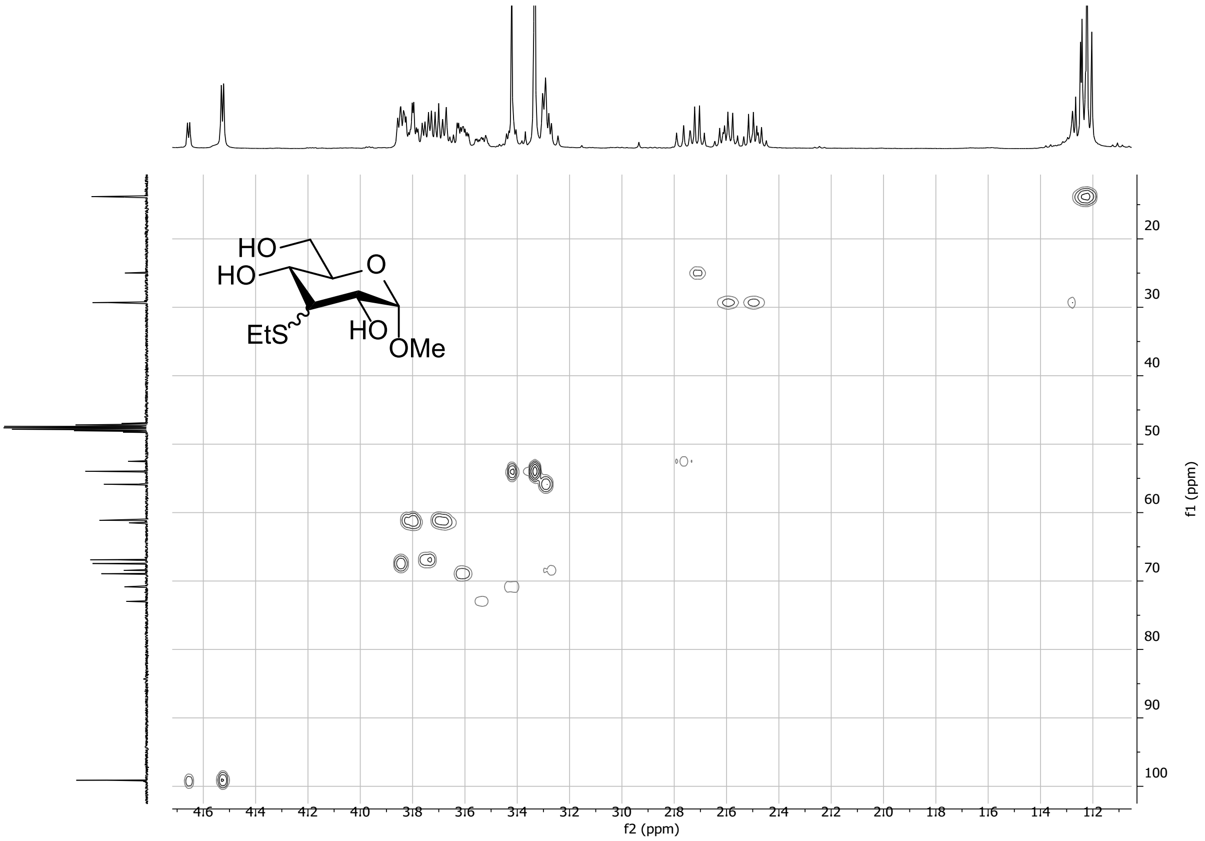

Methyl 3-O-acetyl-α-D-allo/glucopyranoside (S1)

^{1}H NMR, 400 MHz, CD$_3$OD of compound **S1**

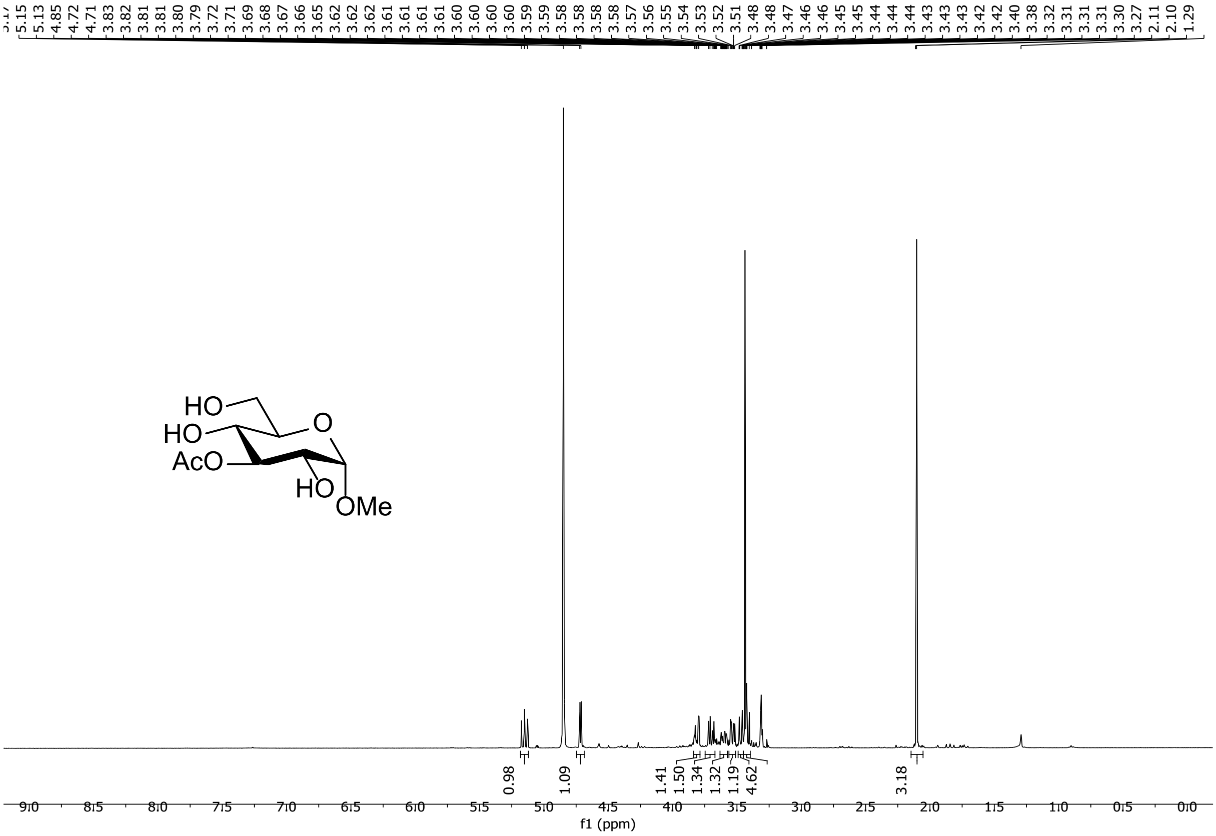

^{13}C NMR, 400 MHz, CD_3OD of compound **S1**

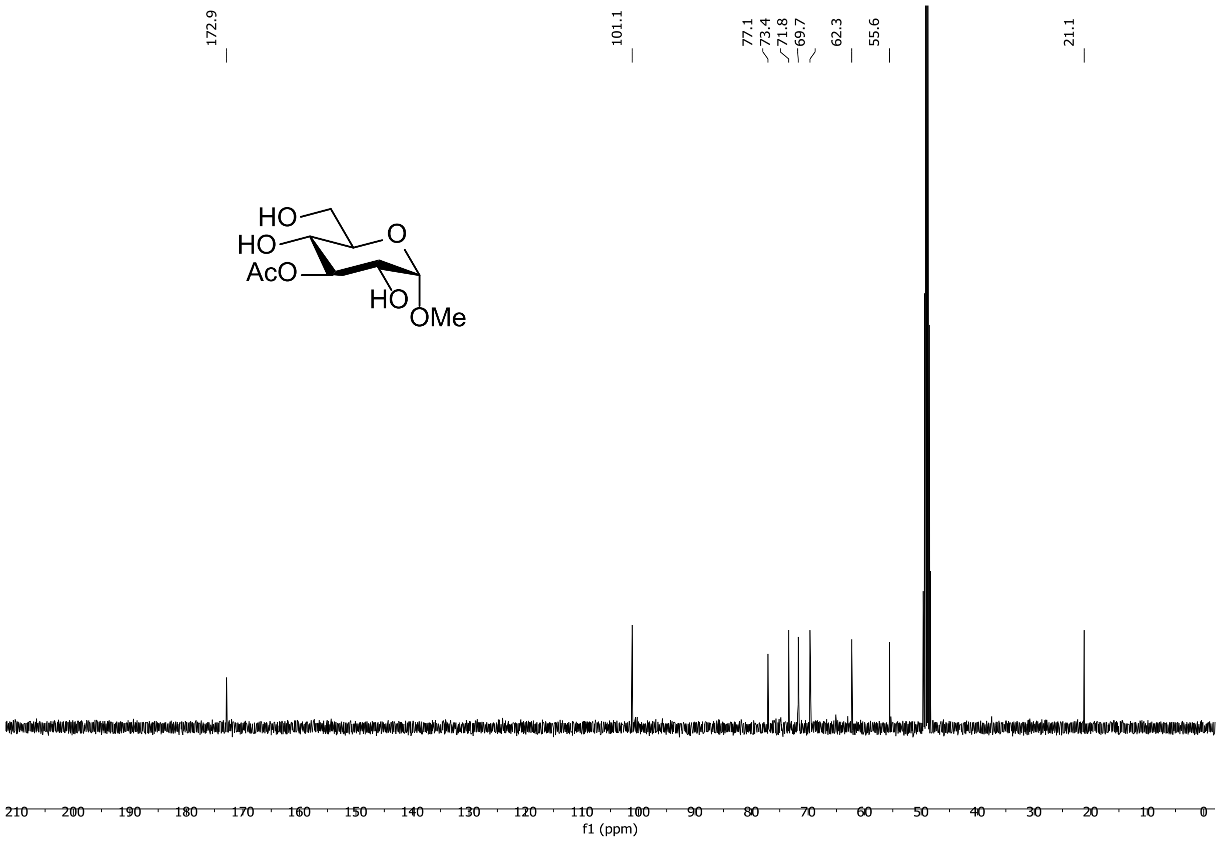

^{1}H-^{1}H COSY of compound **S1**

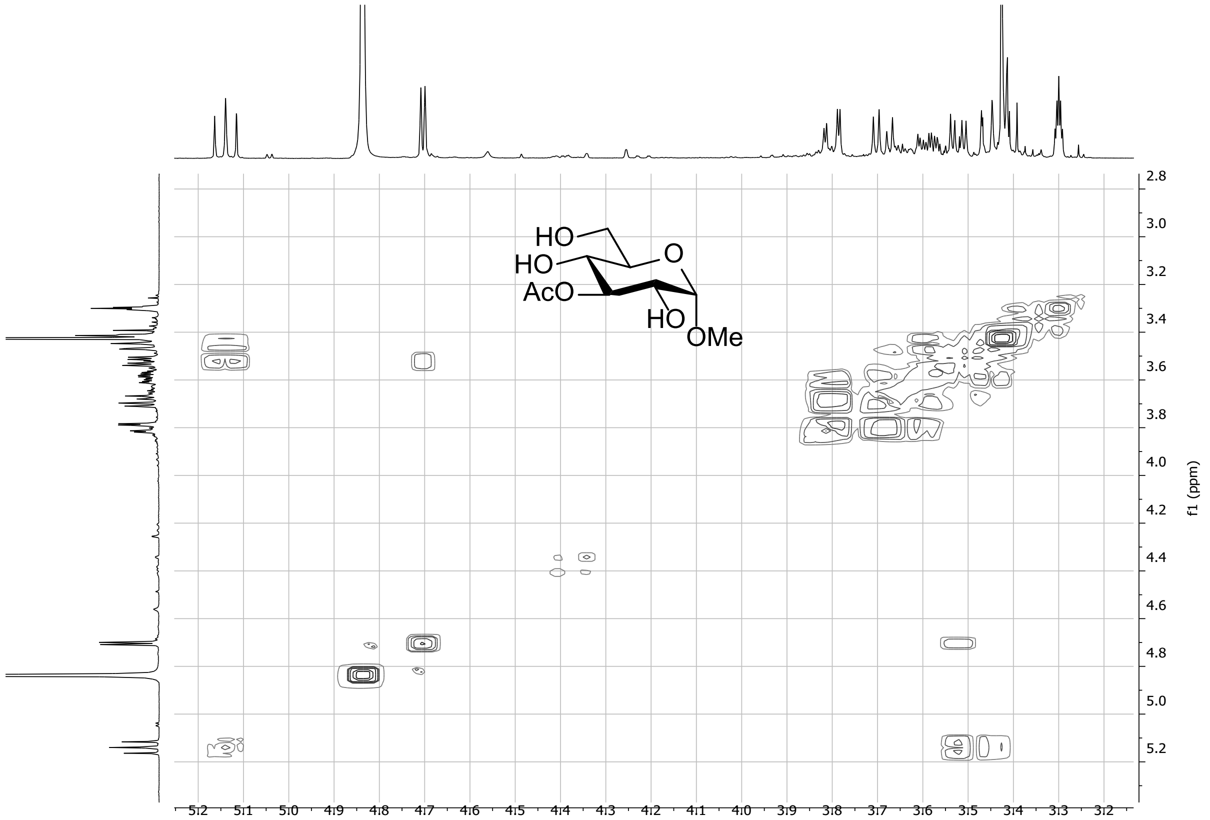

H-^{13}C HSQC of compound **S1**

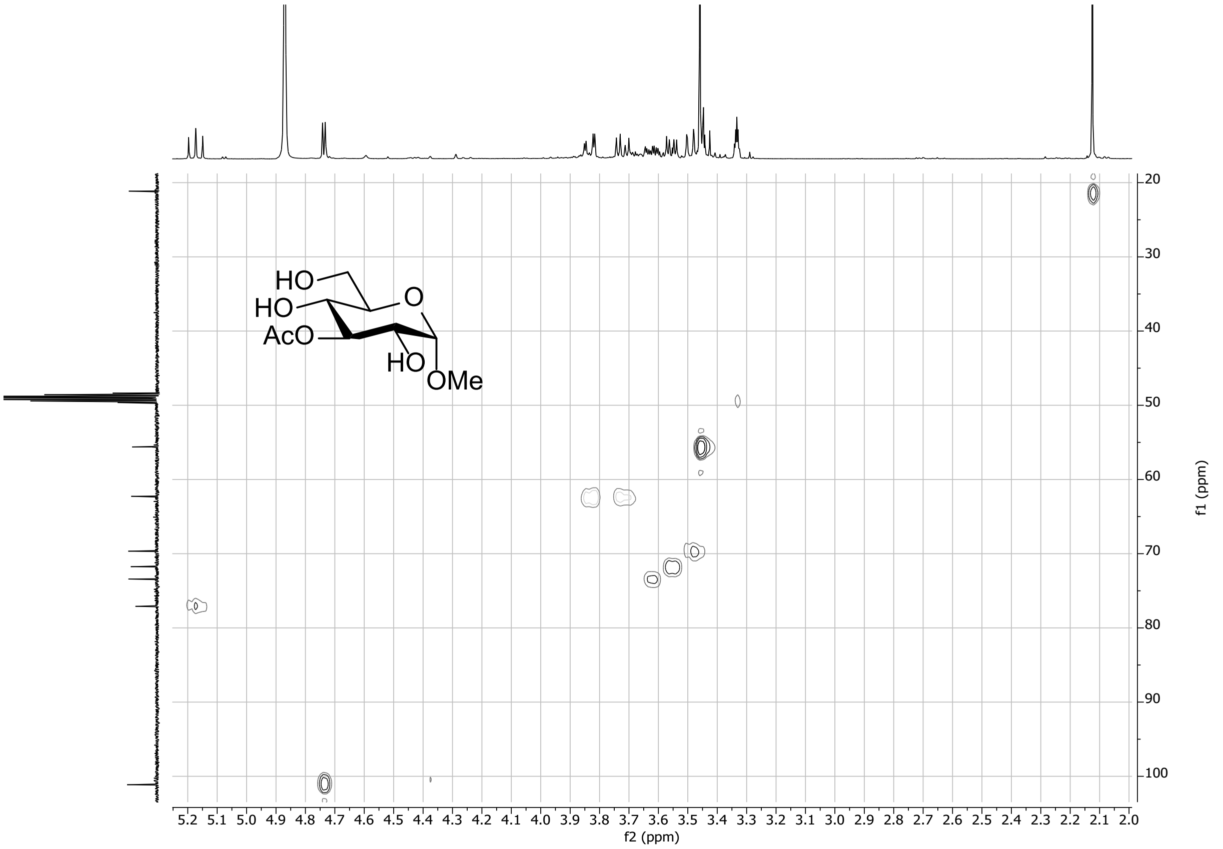

Methyl 3-S-isopropyl-3-deoxy-α-D-allo/glucopyranoside (S2)

^{1}H NMR, 400 MHz, CD_3OD of compound **S2**

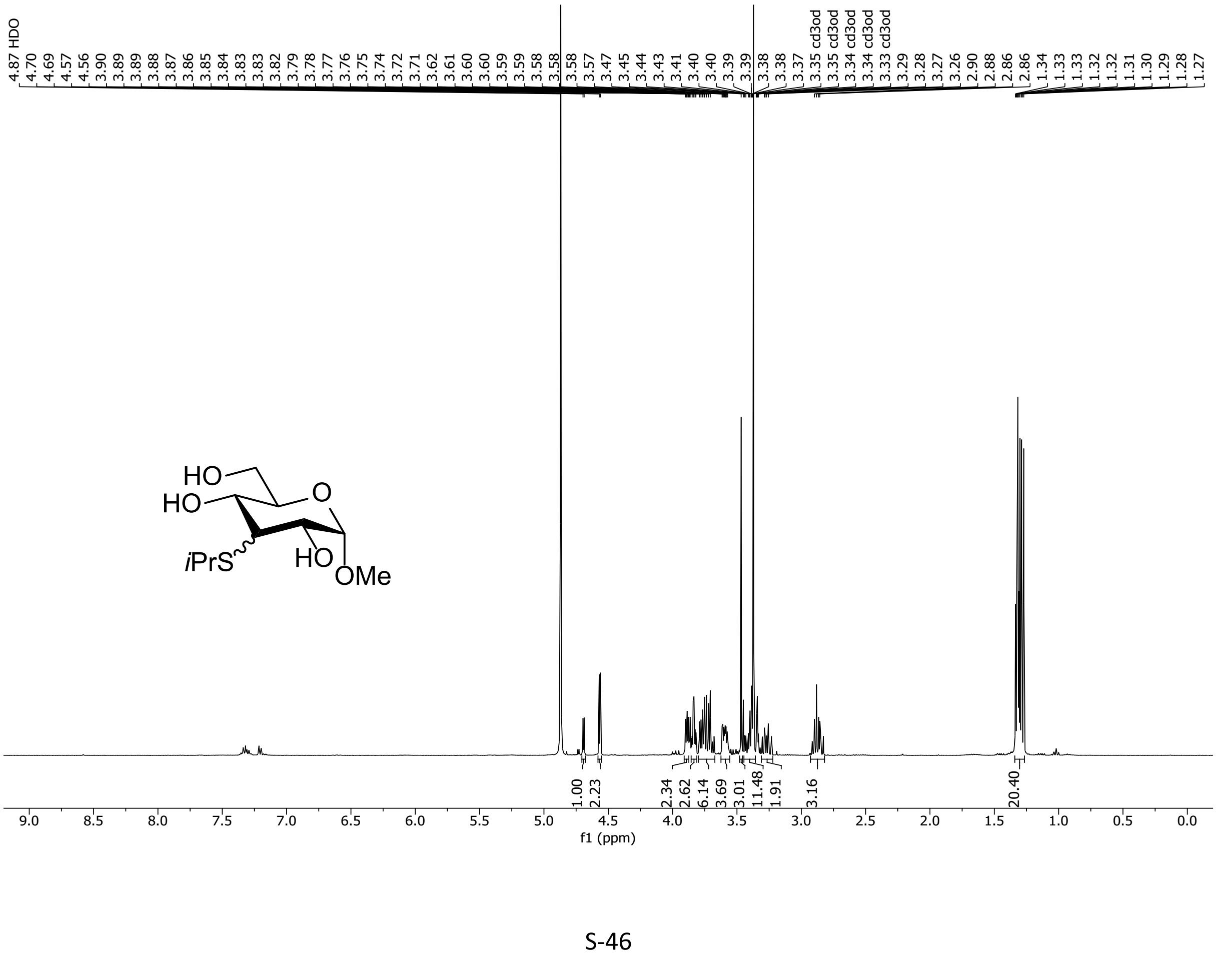

^{13}C NMR, 400 MHz, CD3OD of compound **S2**

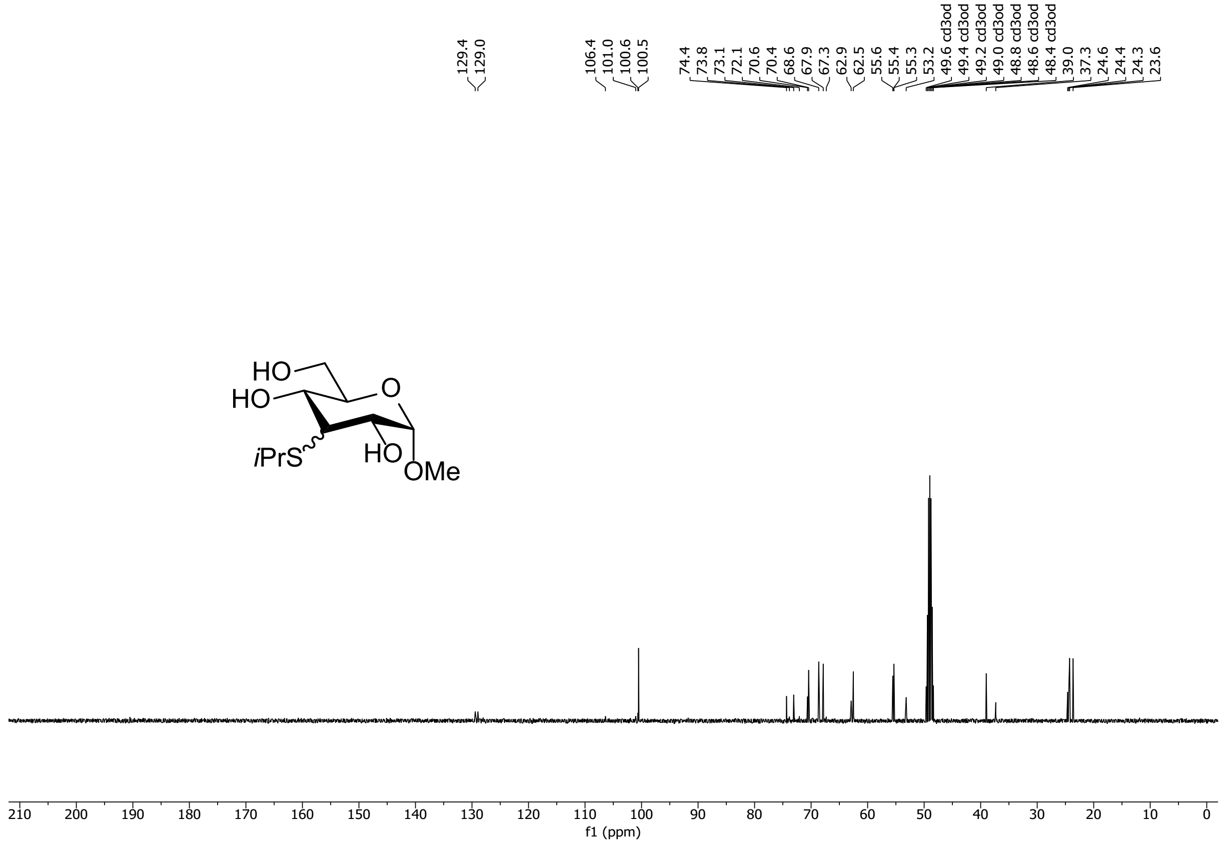

^{1}H-^{1}H COSY of compound **S2**

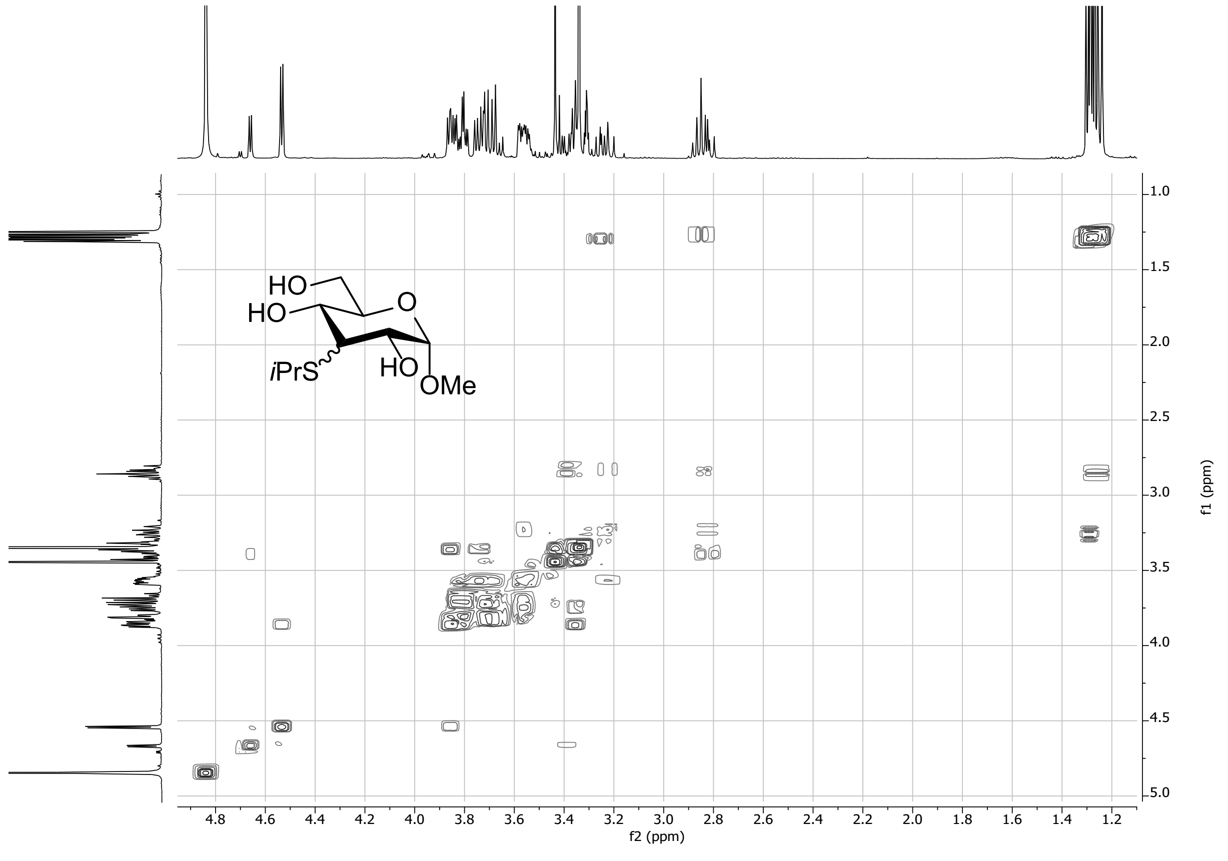

H-^{13}C HSQC of compound **S2**

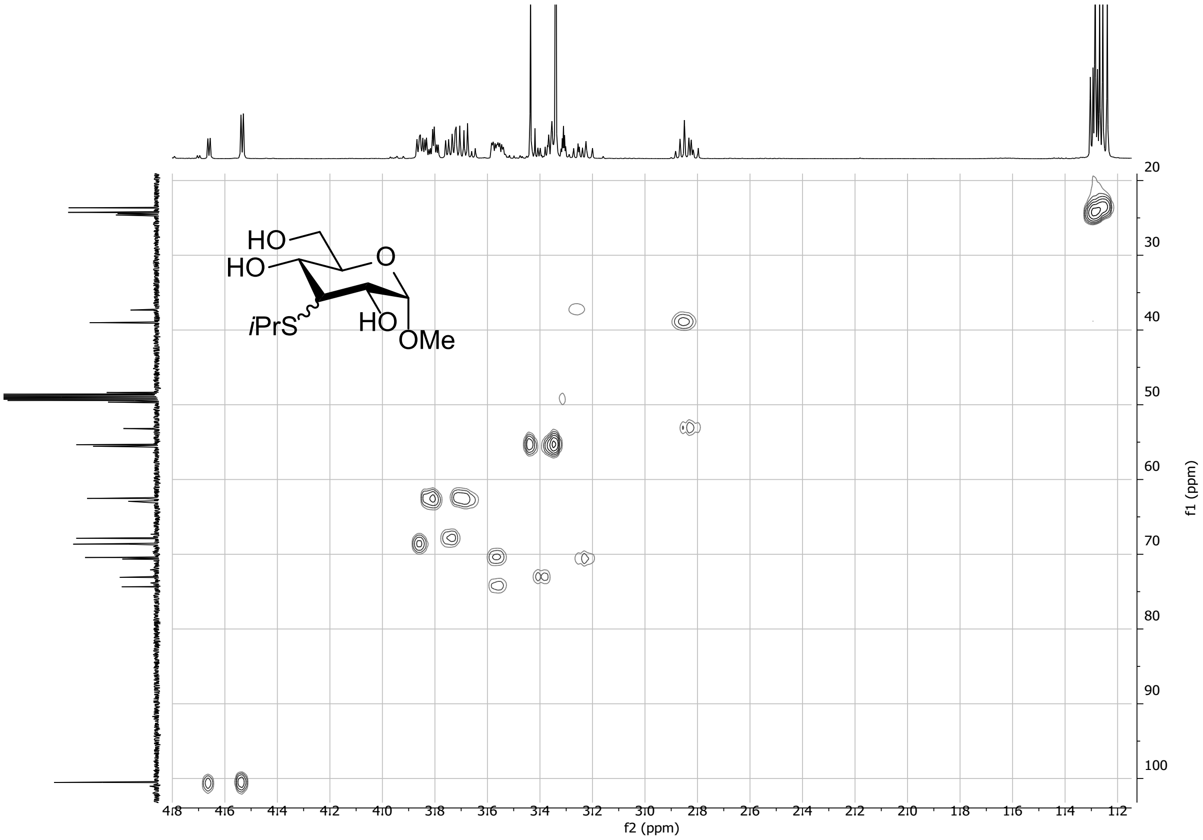

Methyl 3-S-acetyl-3-deoxy-α-D-allo/glucopyranoside (S3)

^{1}H NMR, 400 MHz, CD_3OD of compound **S3**. Small amounts of **3b** also visible.

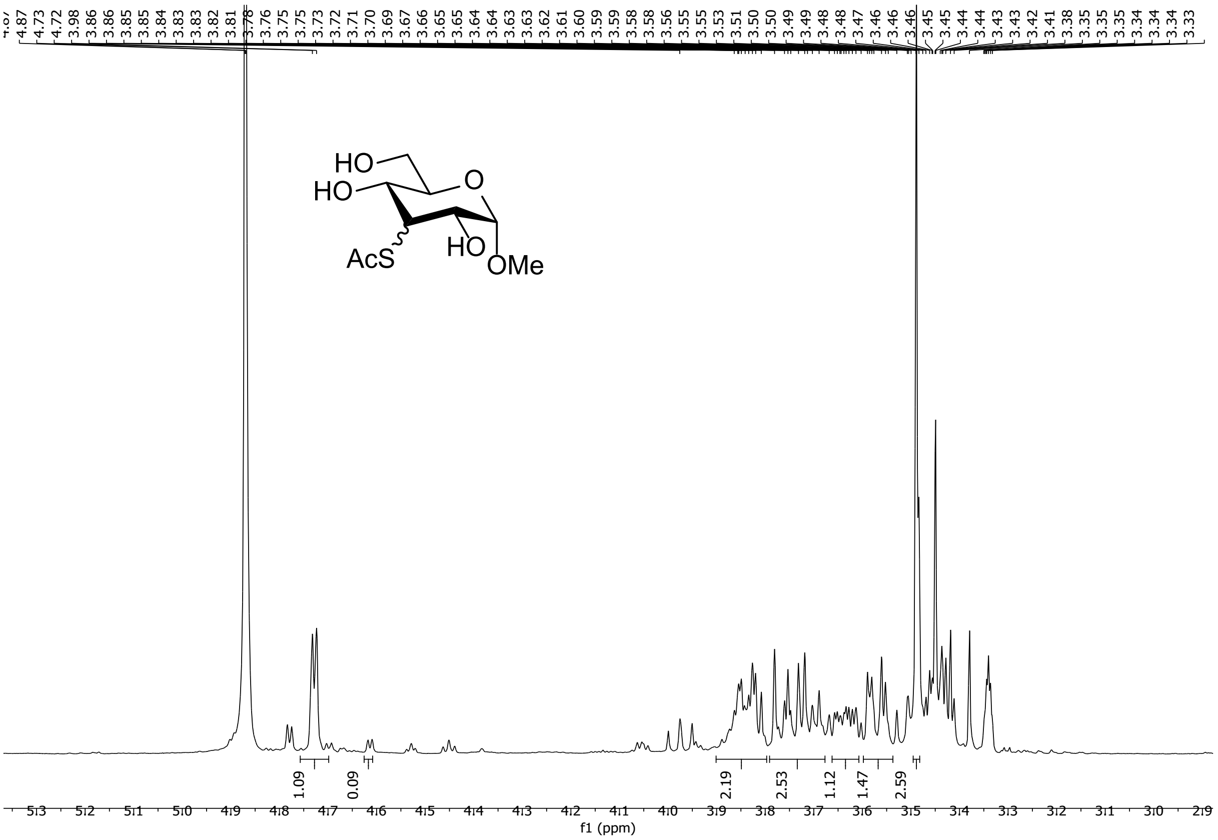

^{1}H NMR, 400 MHz, CD_3OD of compound **S3**: Analytical sample taken with ratio of equatorial/axial: 1/0.8. Over time, acyl migration occurs.

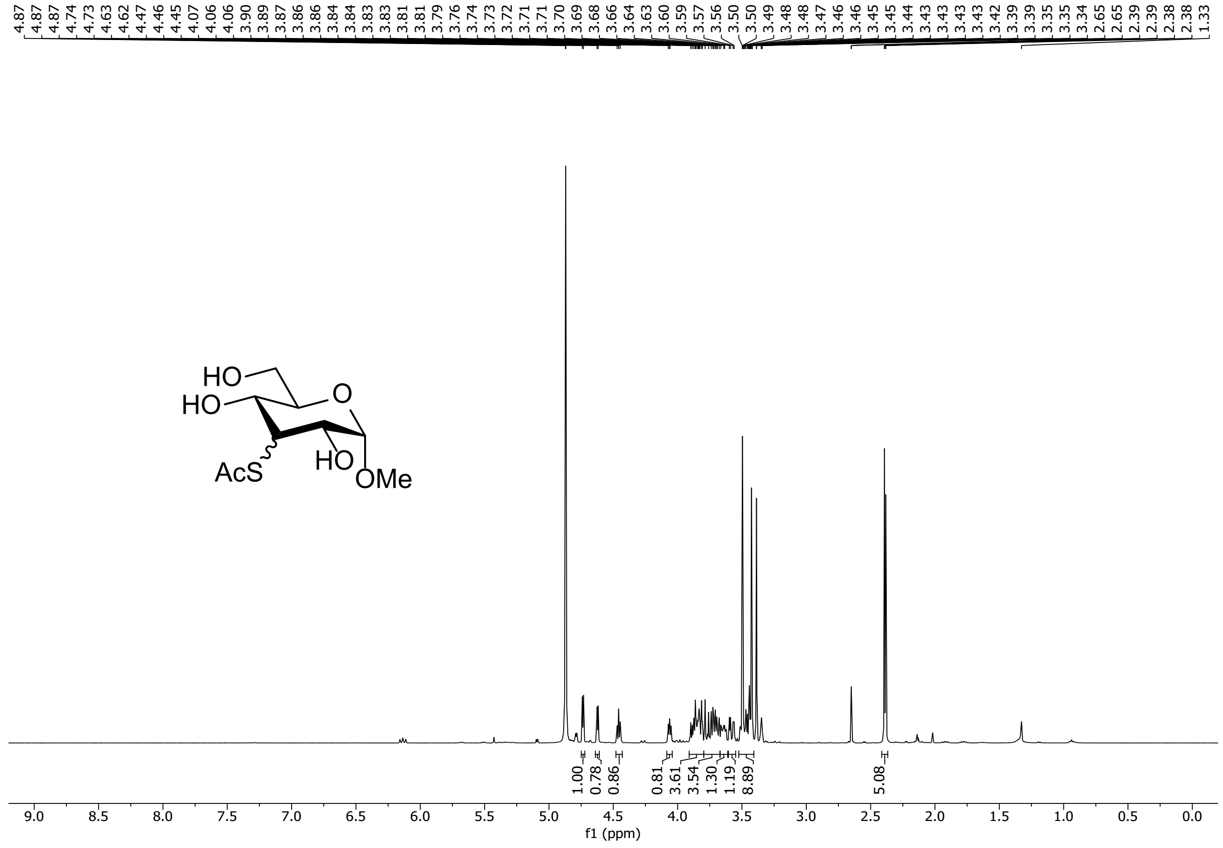

^{13}C NMR, 400 MHz, CD_3OD of compound **S3**

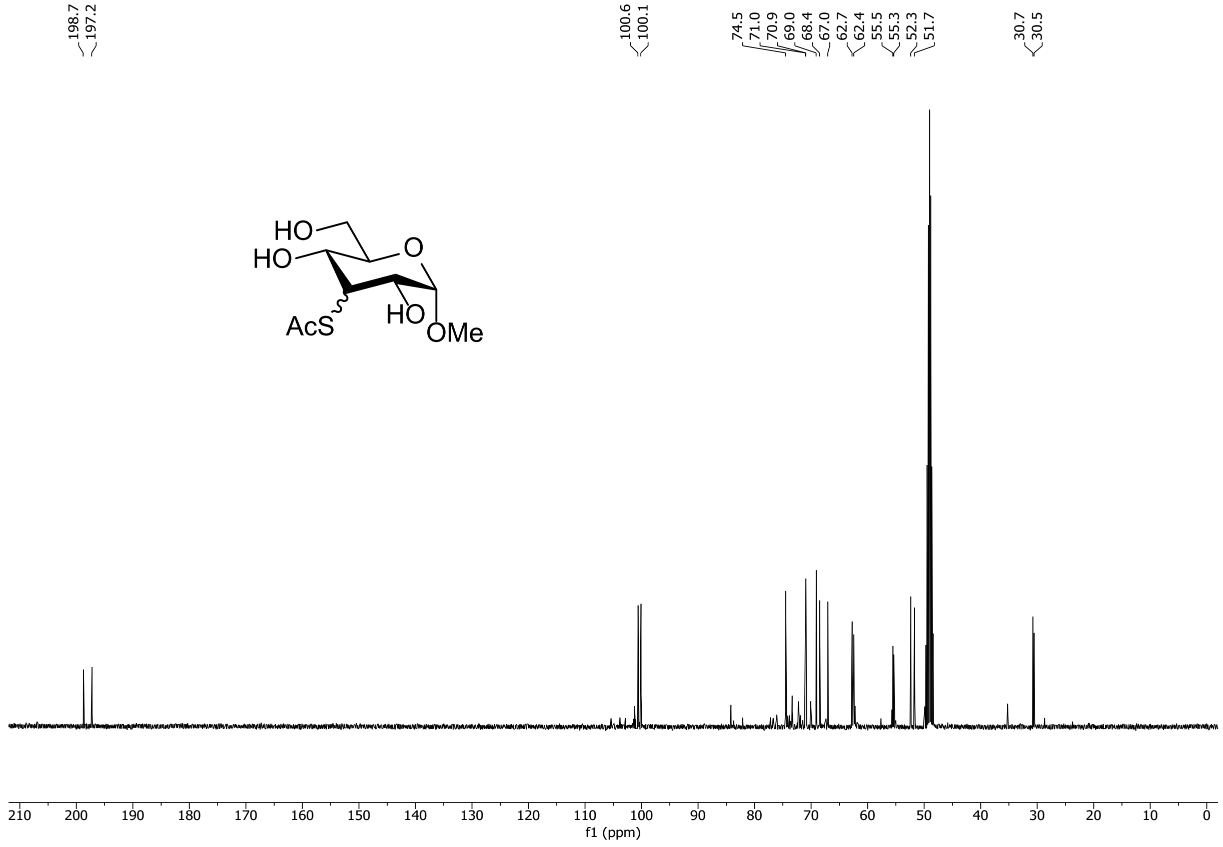

^1H-^1H COSY of compound **S3**

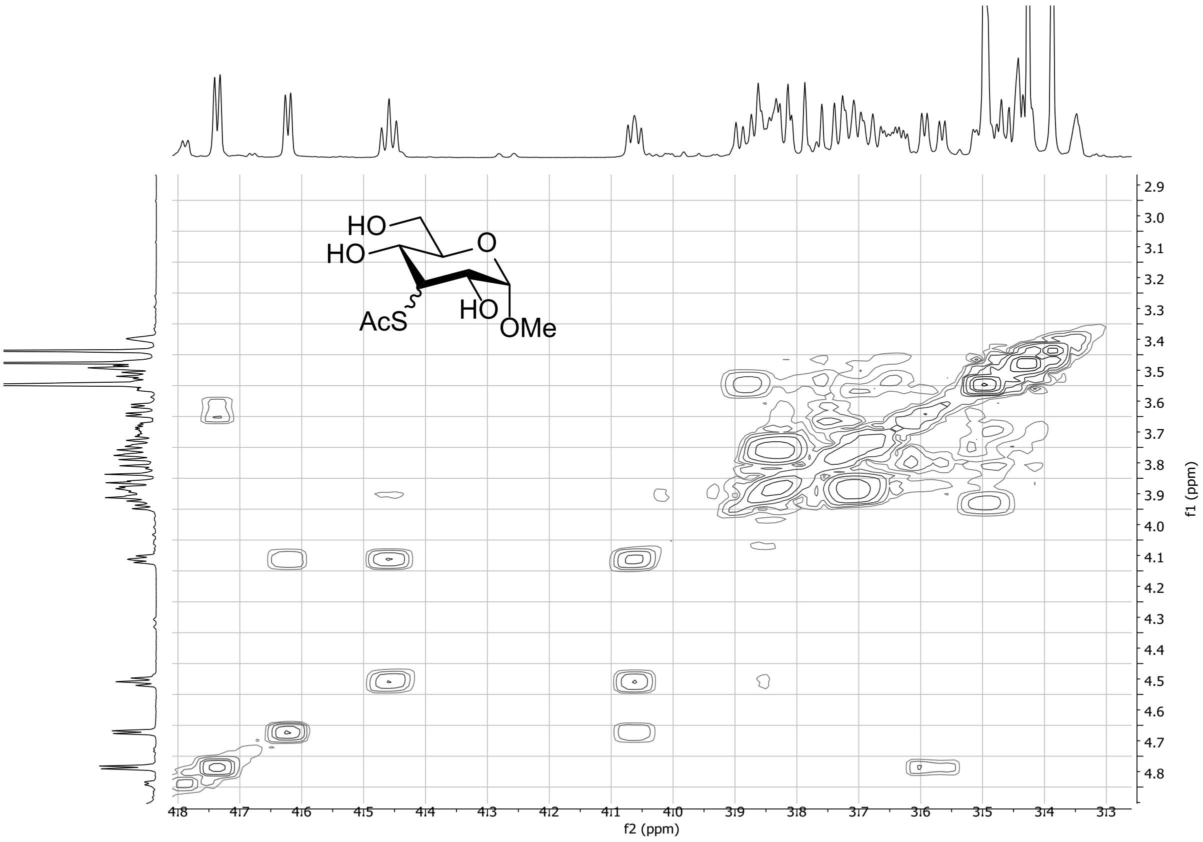

H-^{13}C HSQC of compound **S3**

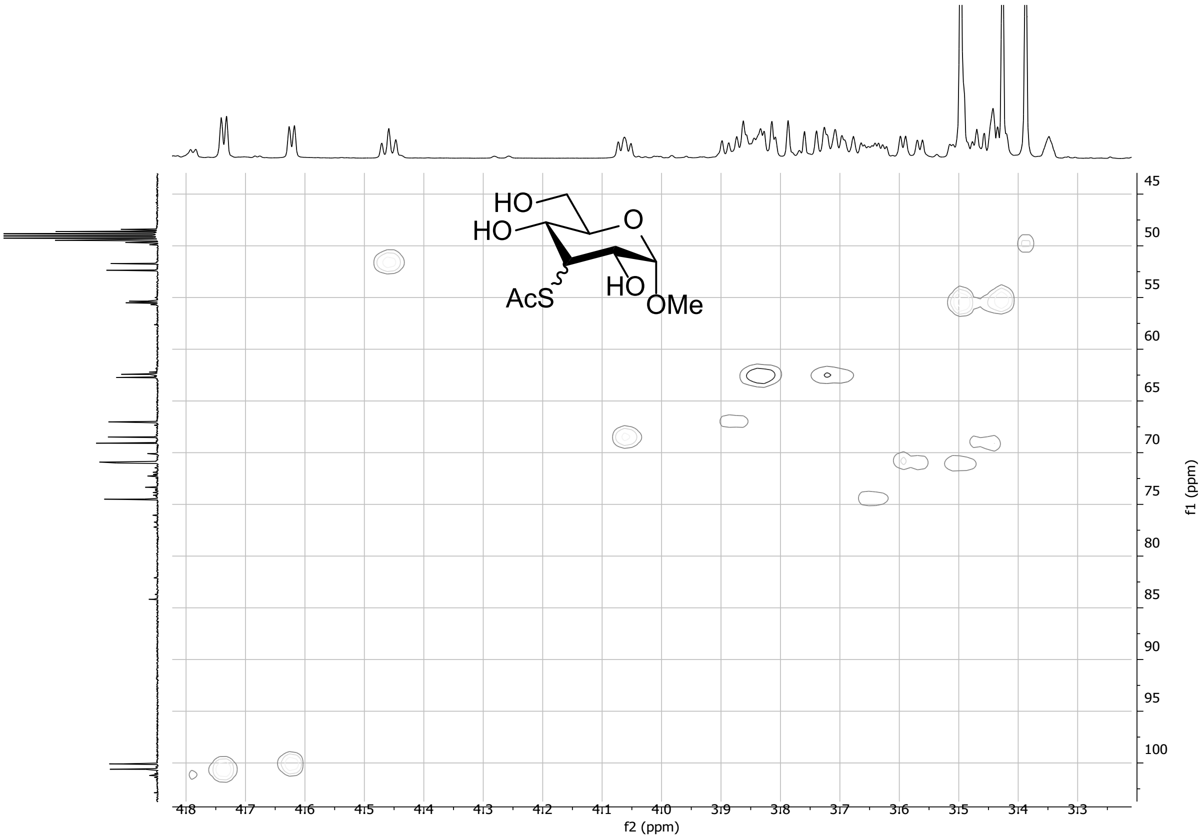

Methyl 3,3-dichloro-3-deoxy-α-D-glucopyranoside (5)

^{1}H NMR, 400 MHz, CD_3OD of compound **5**

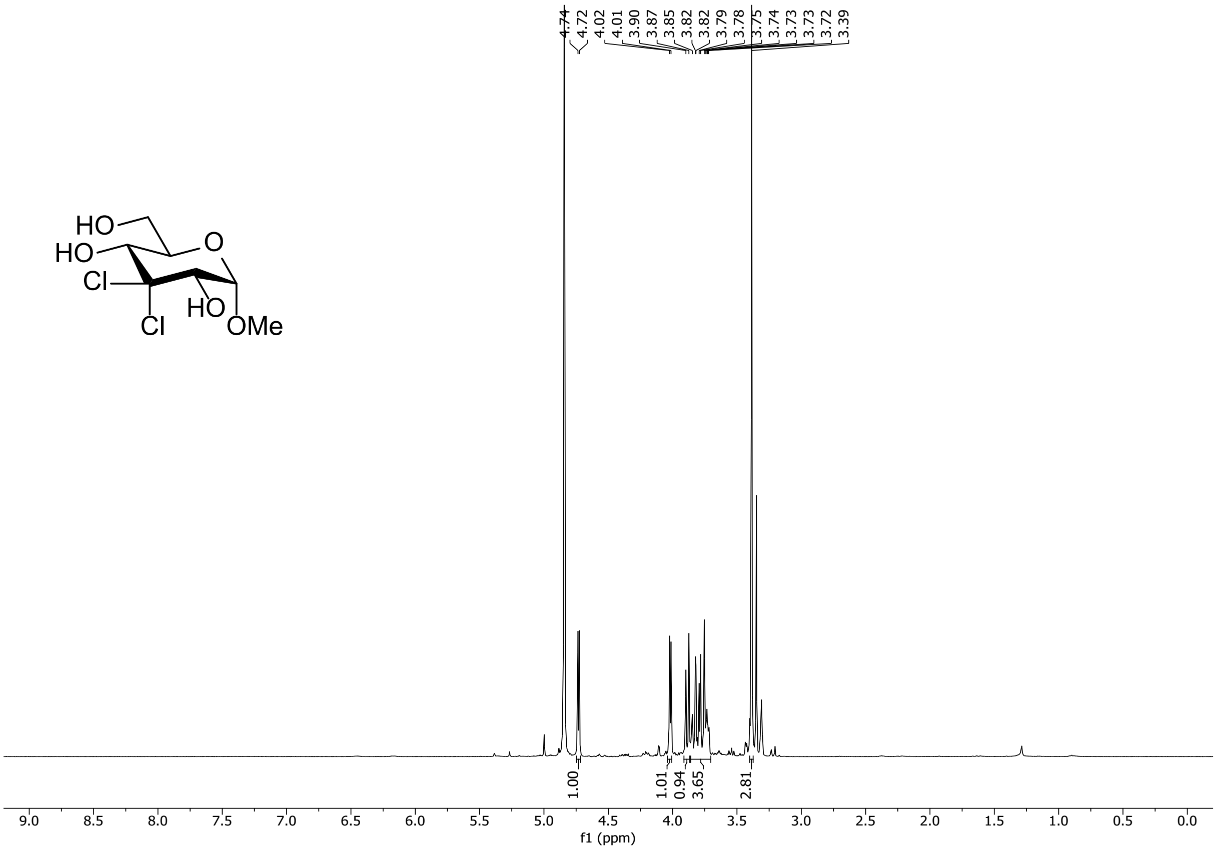

^{13}C NMR, 400 MHz, CD_3OD of compound **5**

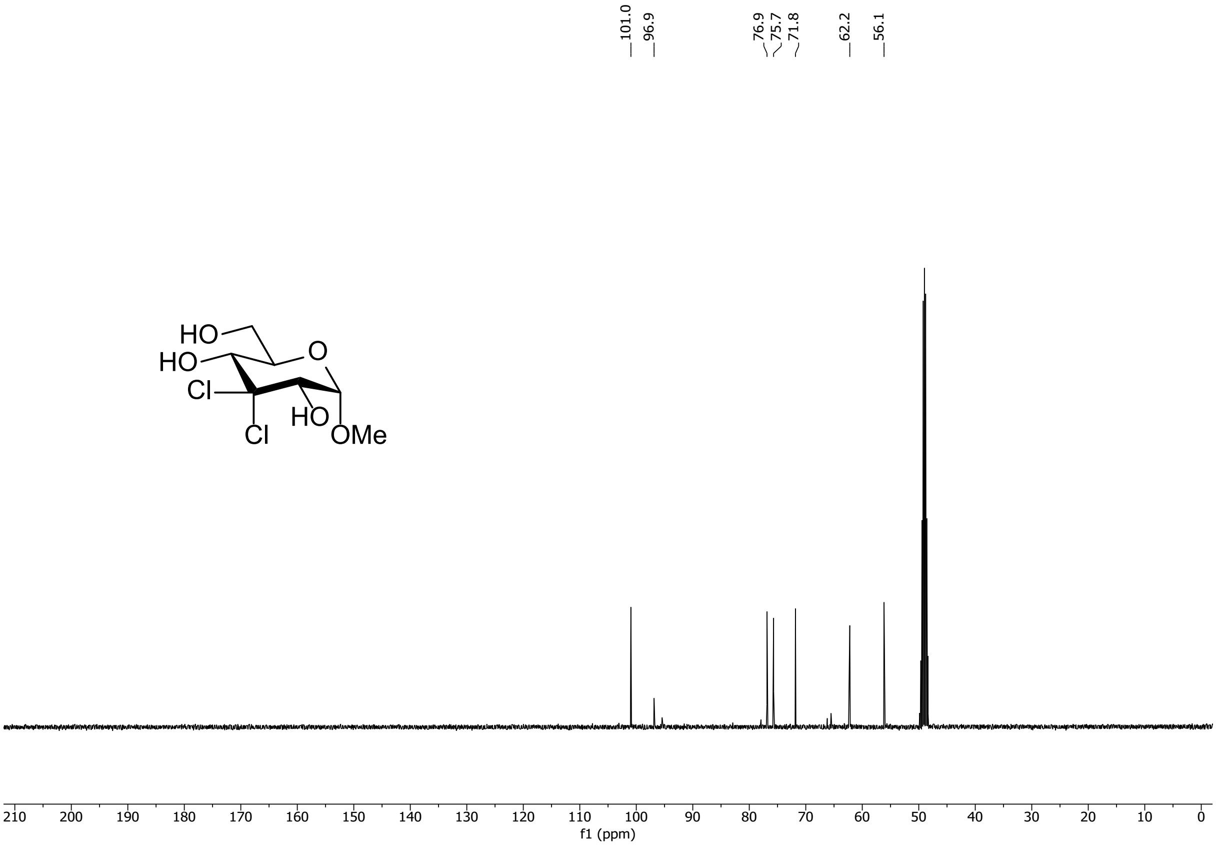

¹H-¹H COSY of compound **5**

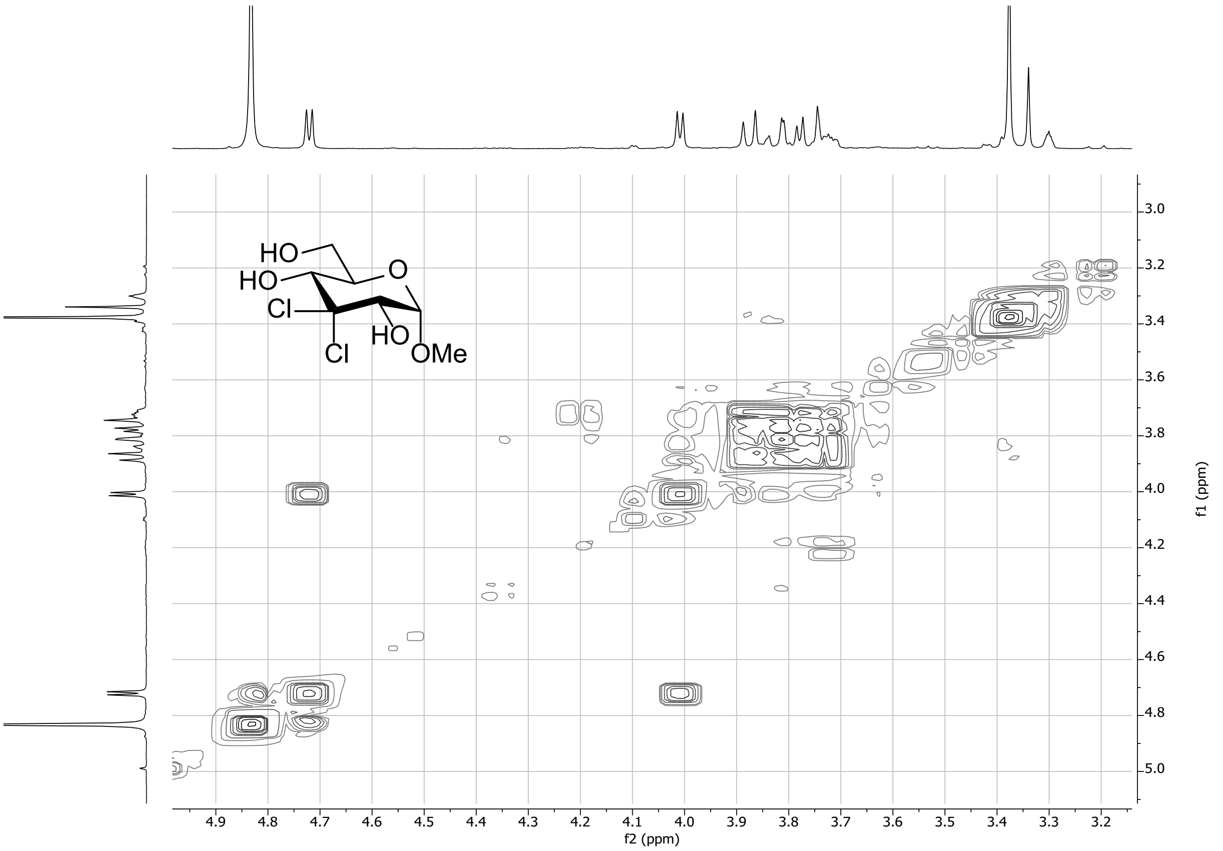

H-¹³C HSQC of compound **5**

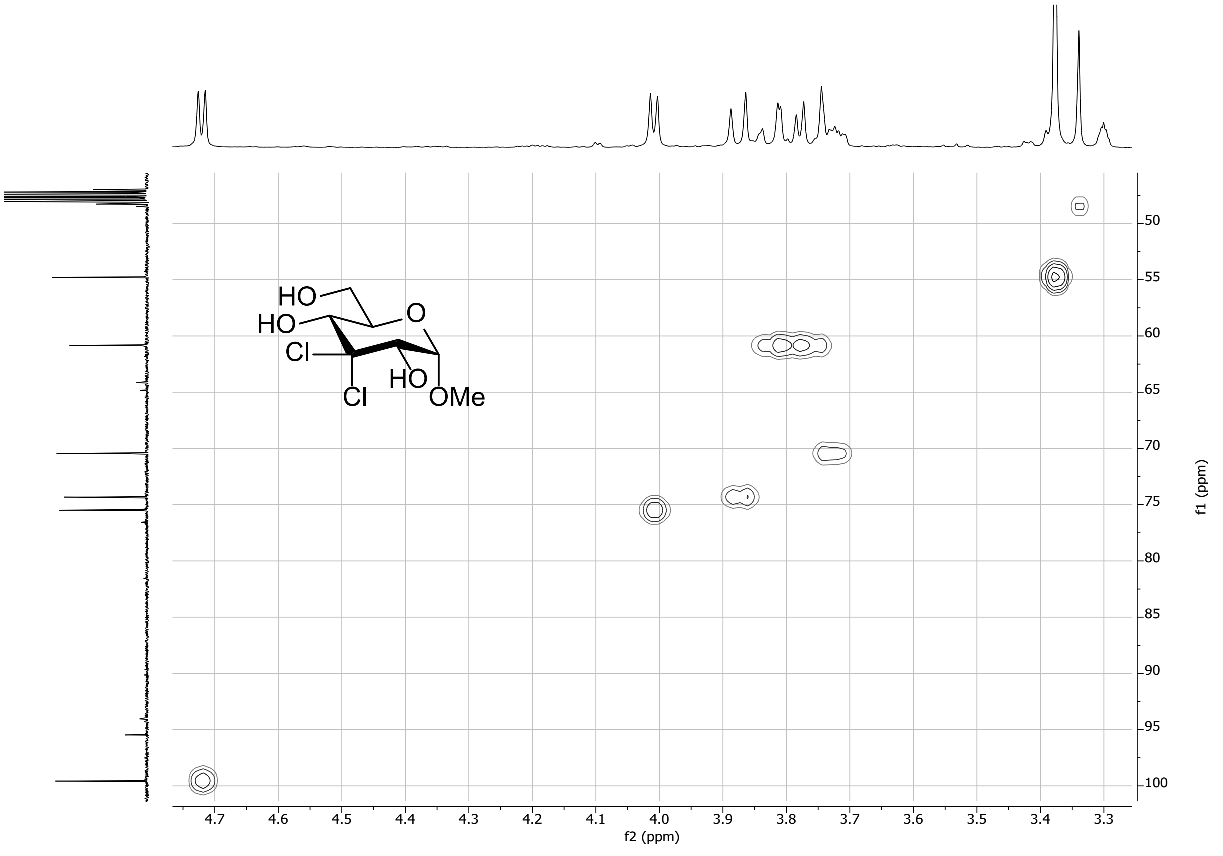

Methyl-3-(2,4-dinitrophenyl)hydrazone-α-D-glucopyranoside (6a)

^{1}H NMR, 400 MHz, CD_3OD of compound **6a**

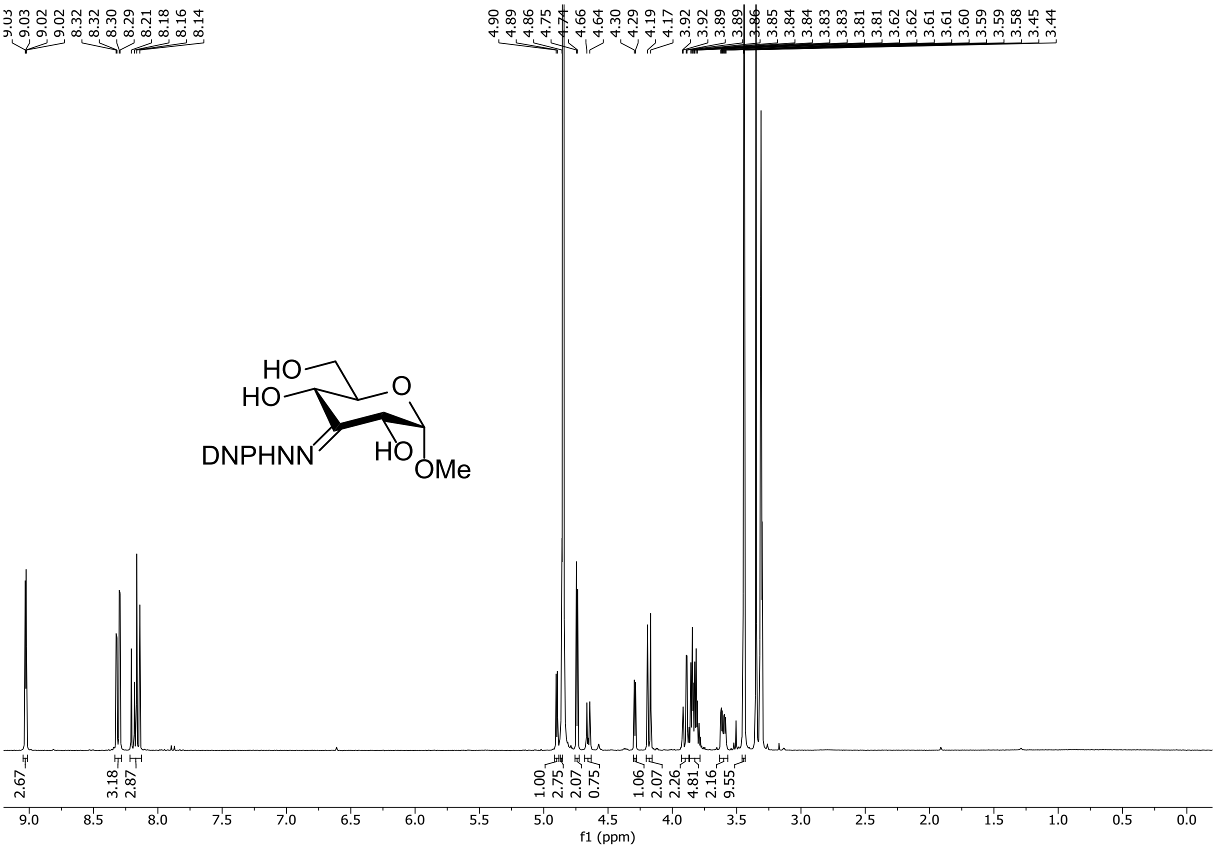

^{13}C NMR, 400 MHz, CD_3OD of compound **6a**

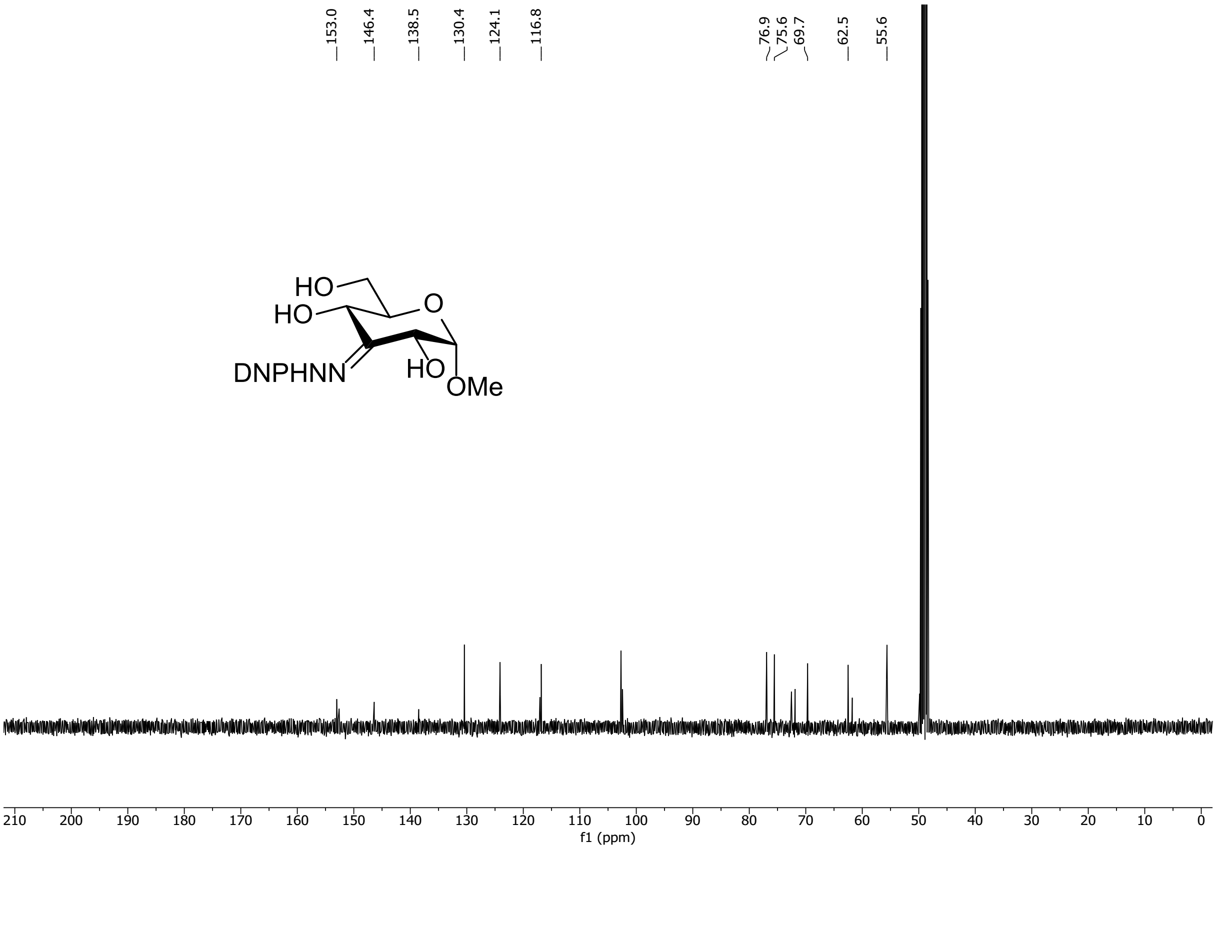

^1H-^1H COSY of compound **6a**

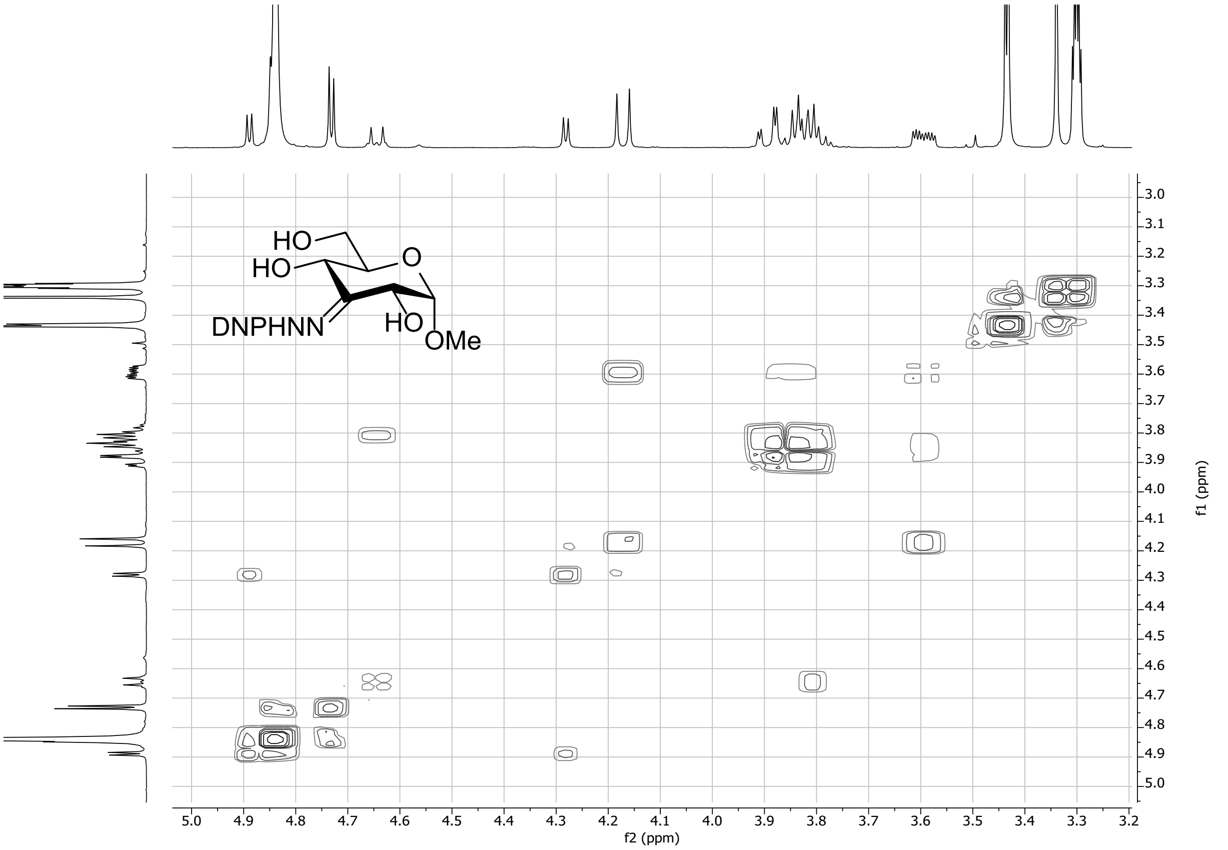

H-^{13}C HSQC of compound **6a**

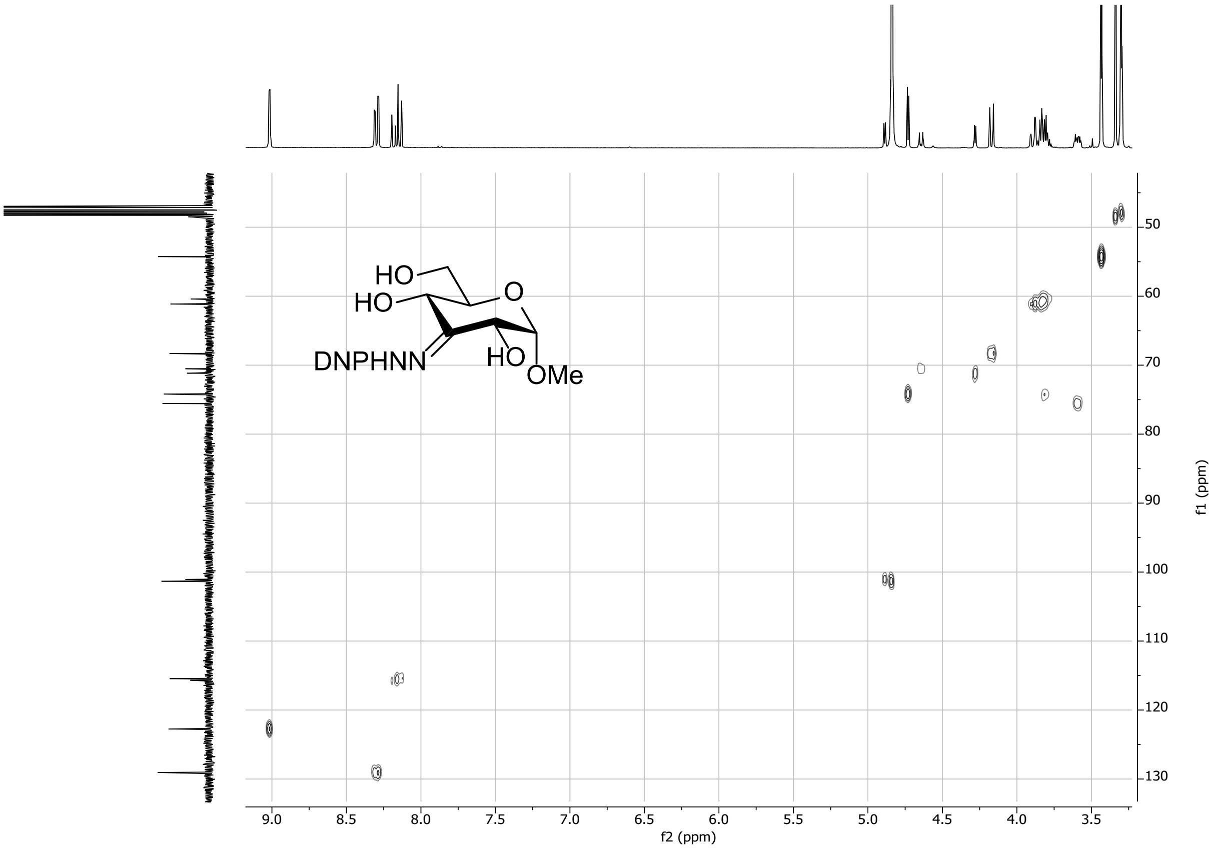

Methyl-3-chloro-3-(2,4-dinitrophenyl)diazene-α-D-glucopyranoside (7a)

^{1}H NMR, 400 MHz, CD_3CN of compound **7a**

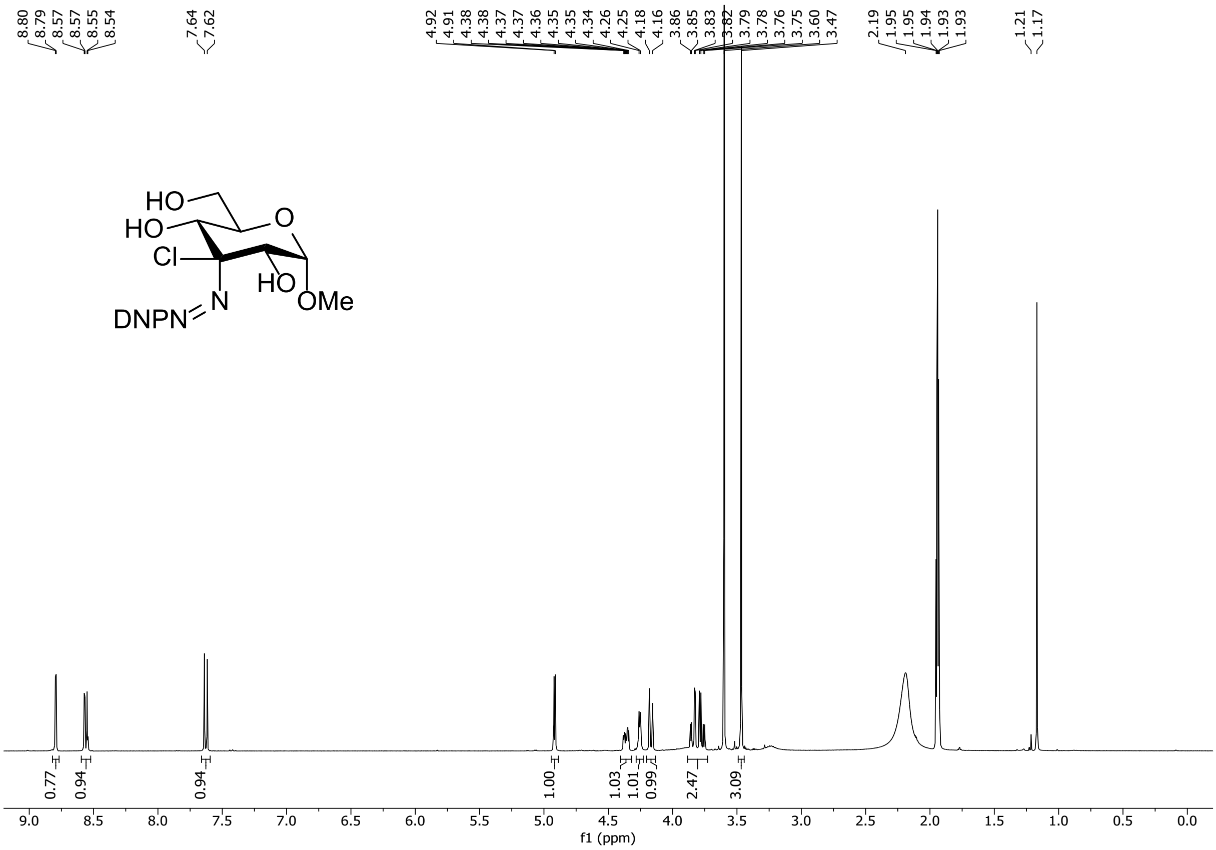

^{13}C NMR, 400 MHz, CD_3CN of compound **7a**

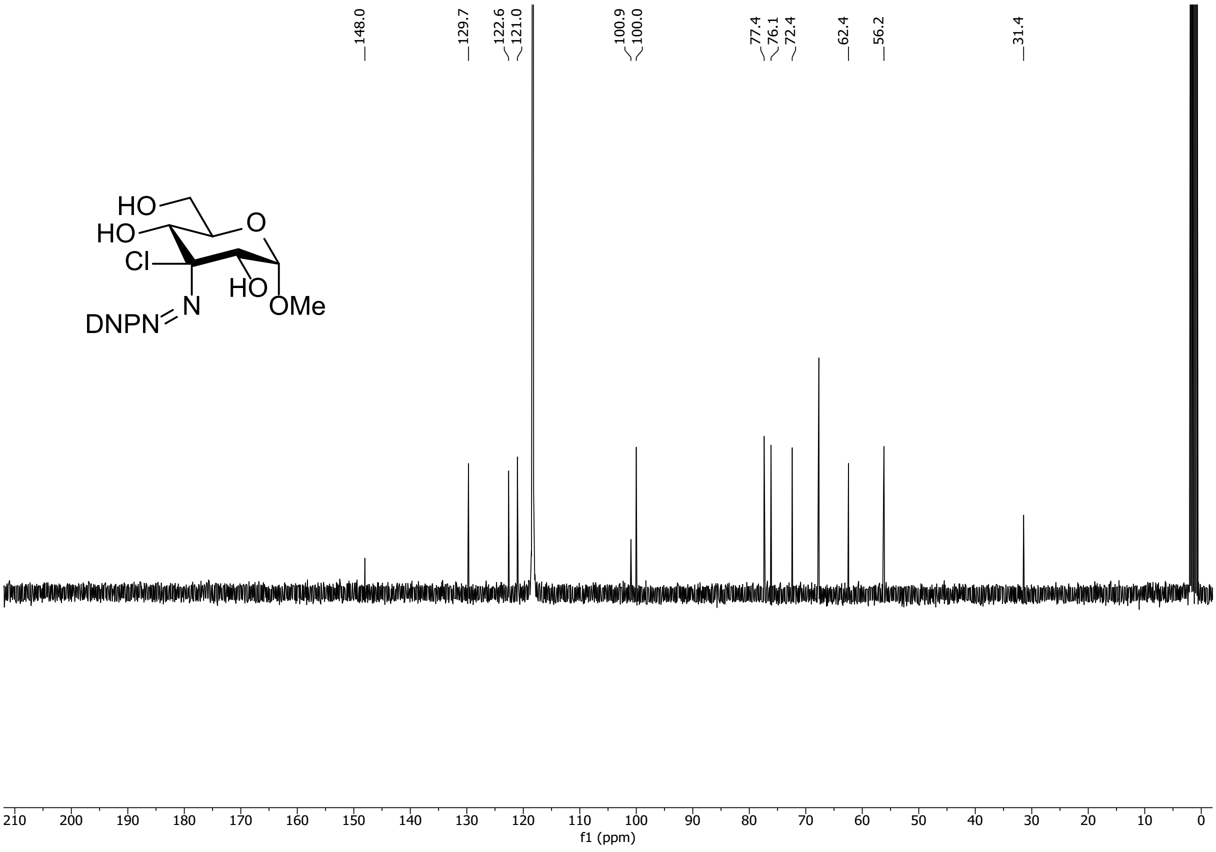

^{1}H-^{1}H COSY of compound **7a**

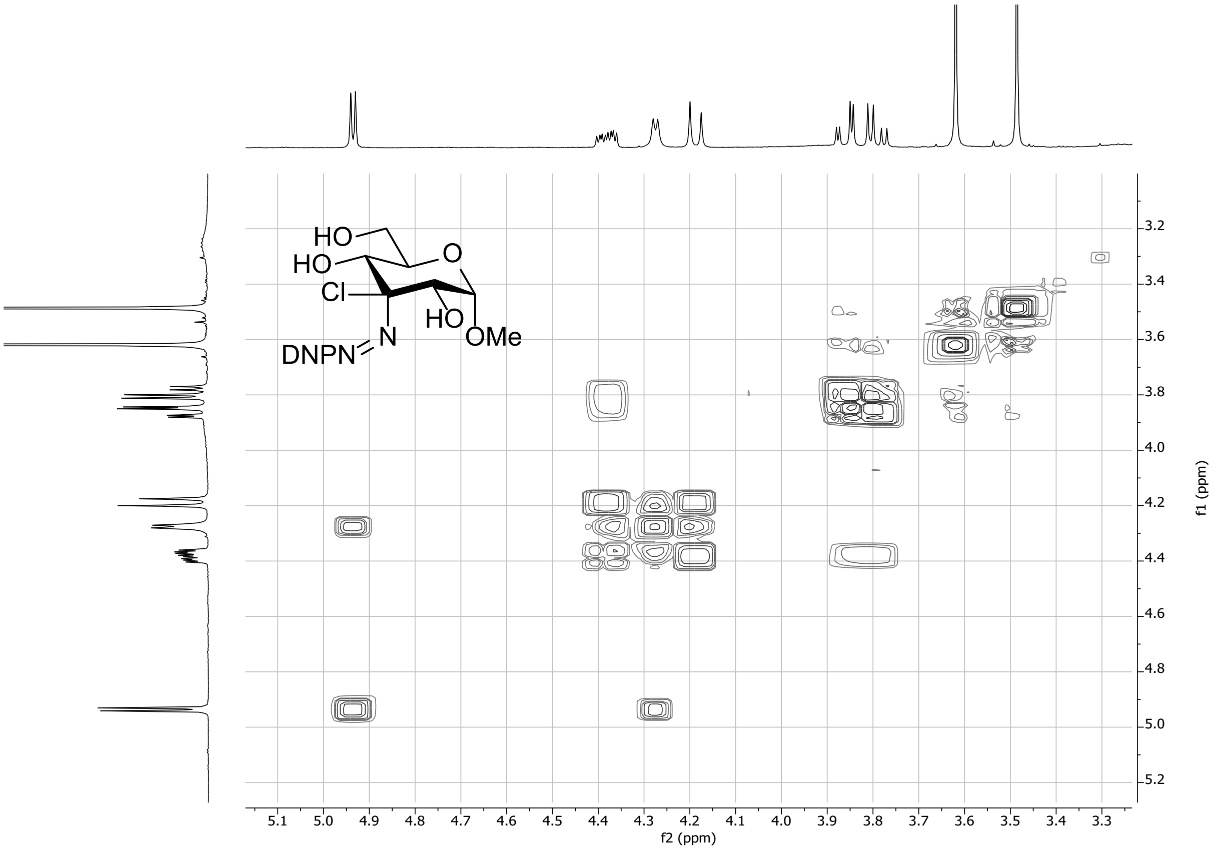

H-^{13}C HSQC of compound **7a**

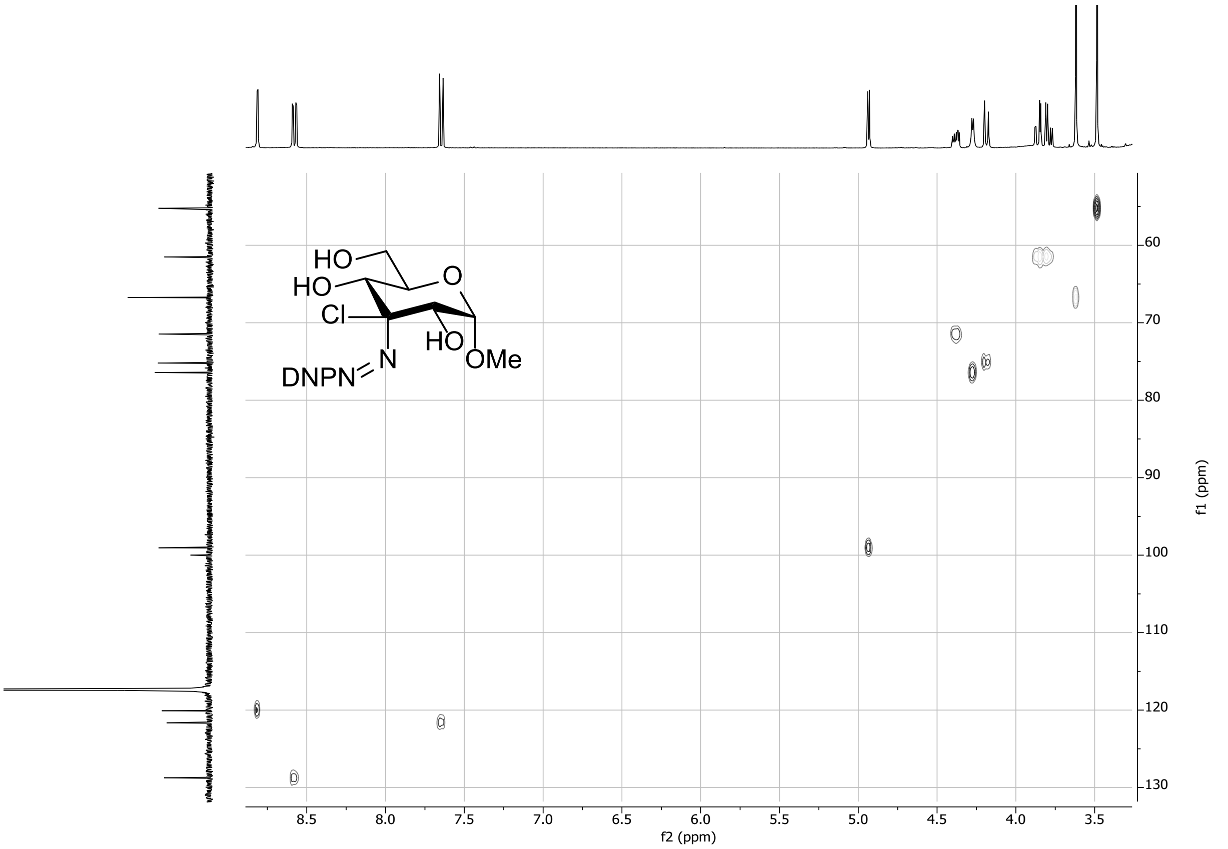

NMR spectra of Gal derivatives

Methyl a-D-xylohexopyranosid-4-ulose (1c)

^{1}H NMR, 400 MHz, DMSO-d*6* of compound **1c**

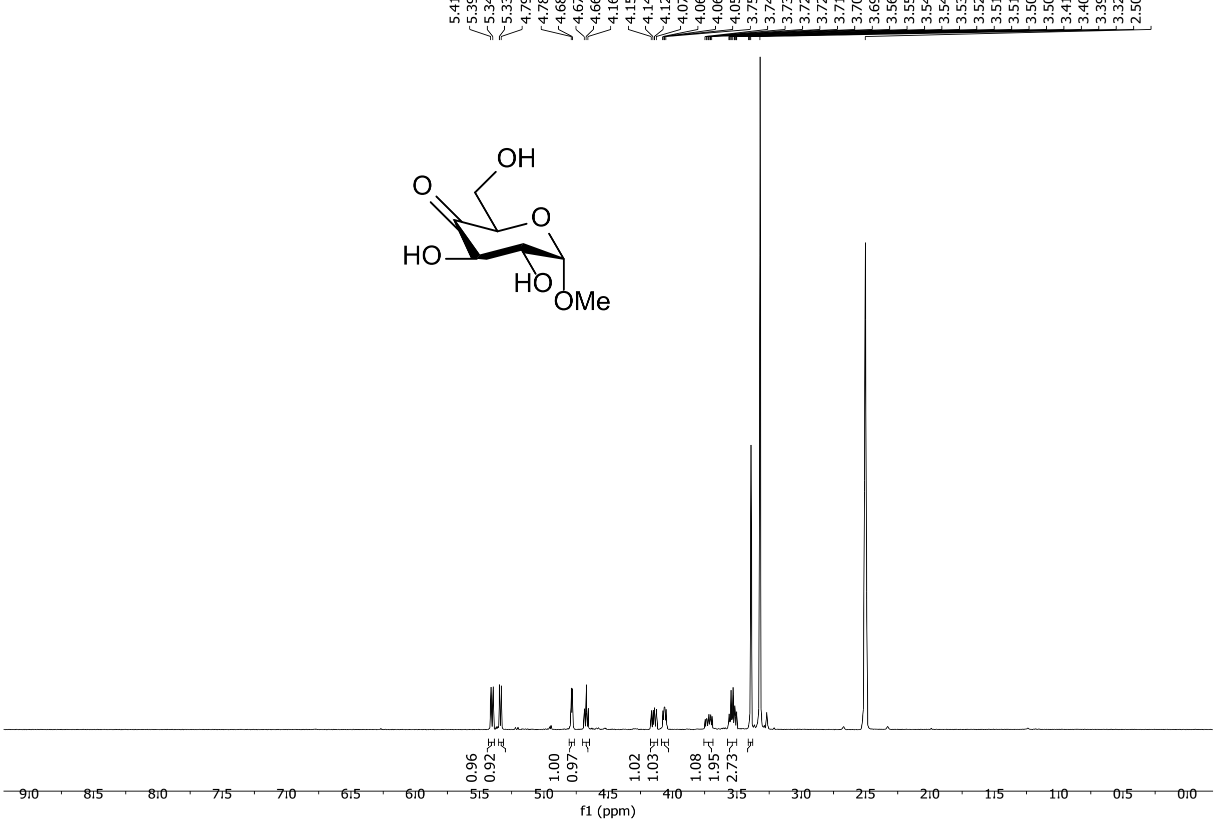

^{13}C NMR, 101 MHz, DMSO-d*6* of compound **1c**

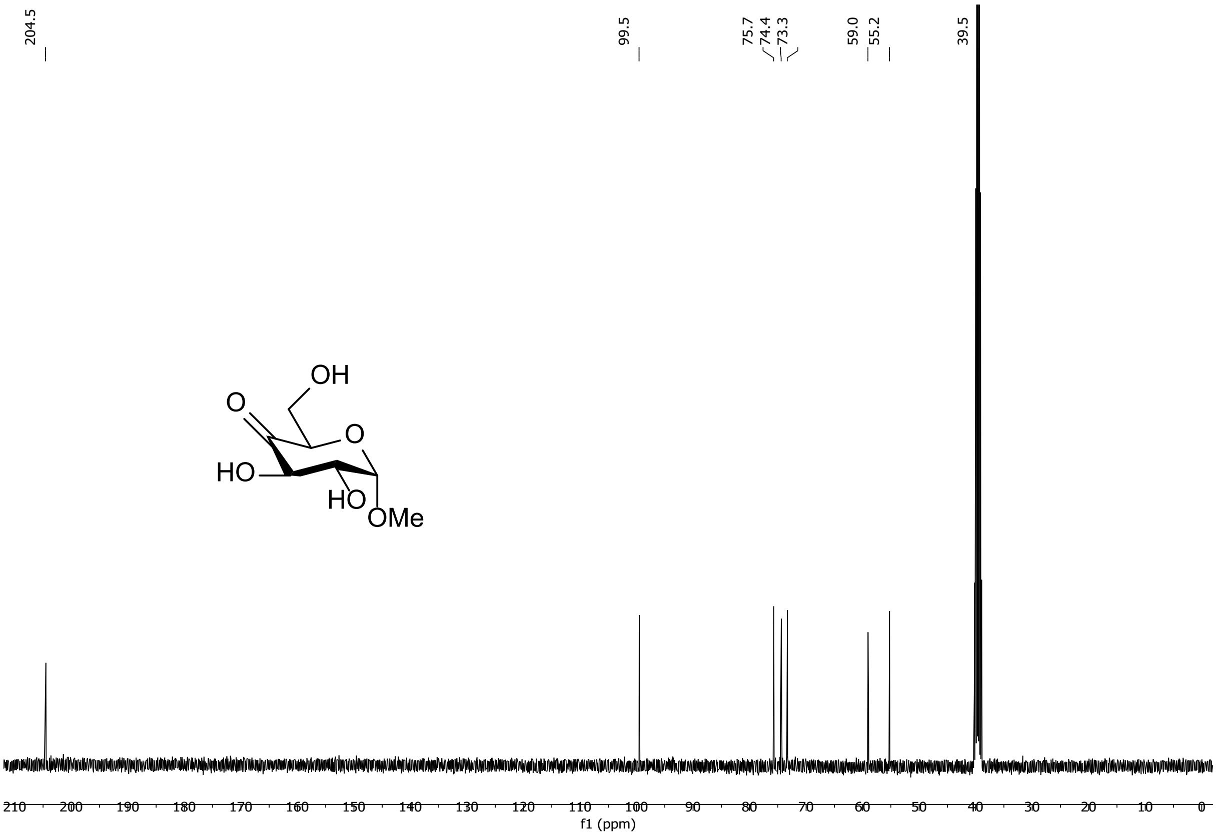

^{1}H-^{1}H COSY of compound **1c**

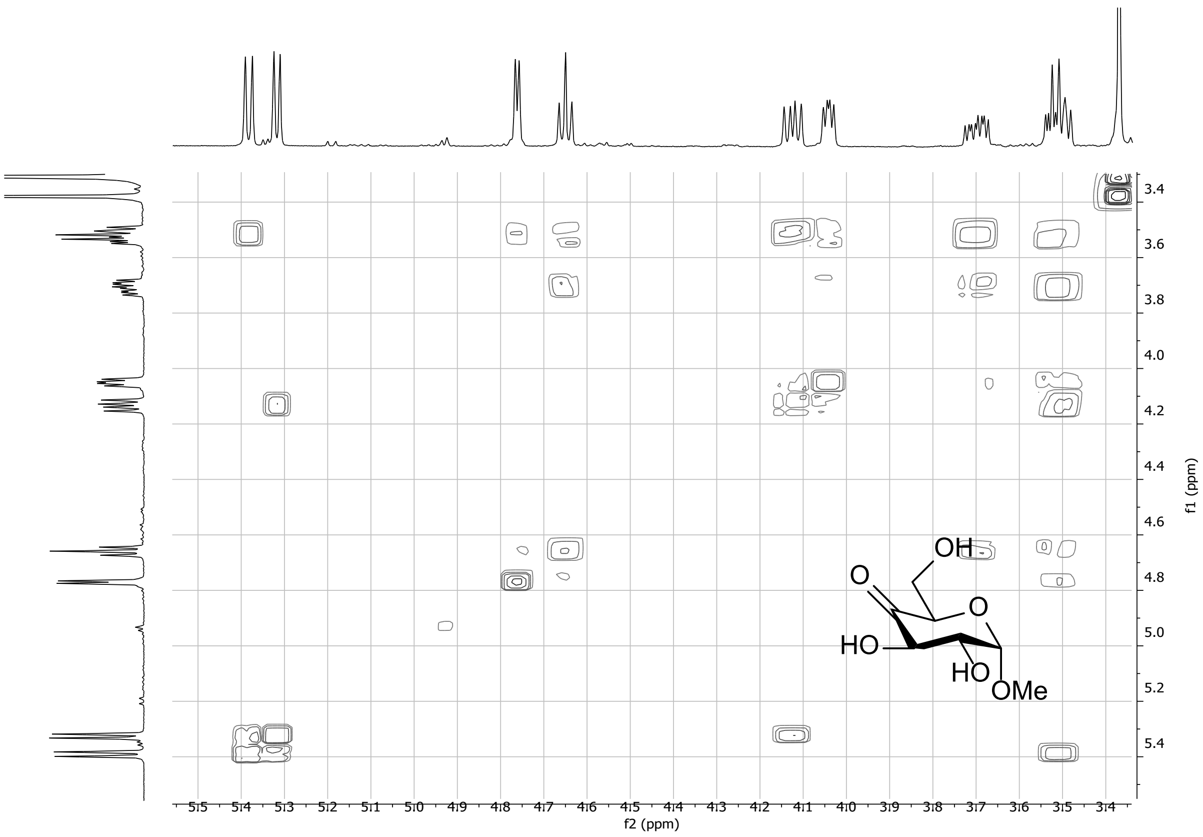

^{1}H-^{13}C HSQC of compound **1c**

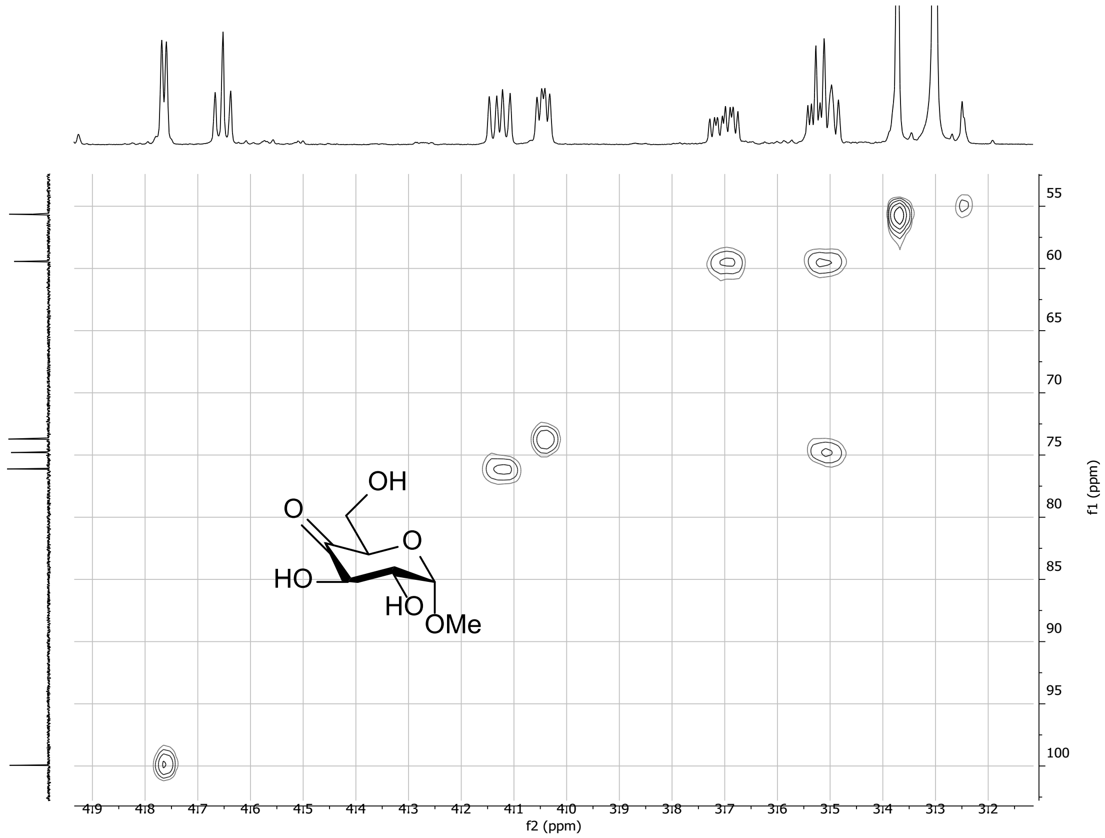

Methyl 4-(trityl)hydrazone-a-D-xylohexopyranosid-4-ulose (2c)

^{1}H NMR, 400 MHz, CD_3OD of compound **2c**

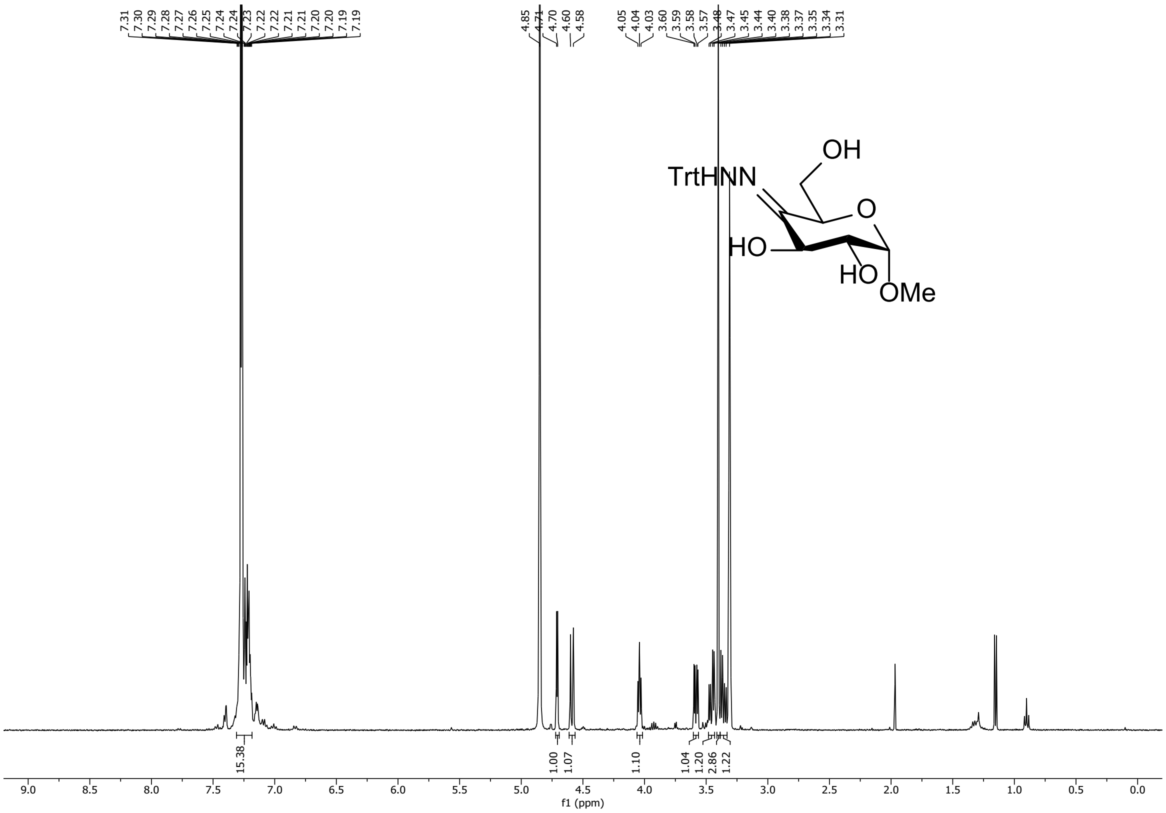

^{13}C-APT NMR, 101 MHz, CD_3OD of compound **2c**

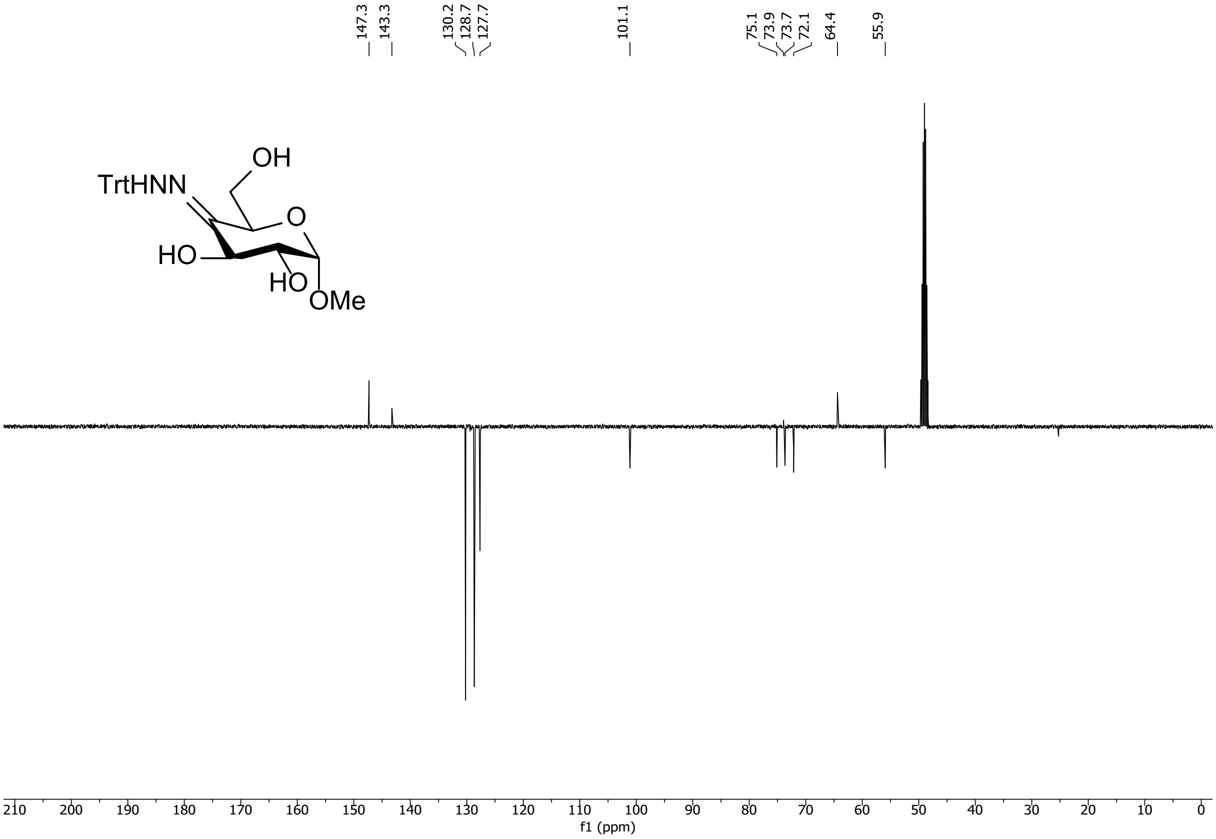

^{1}H-^{1}H COSY of compound **2c**

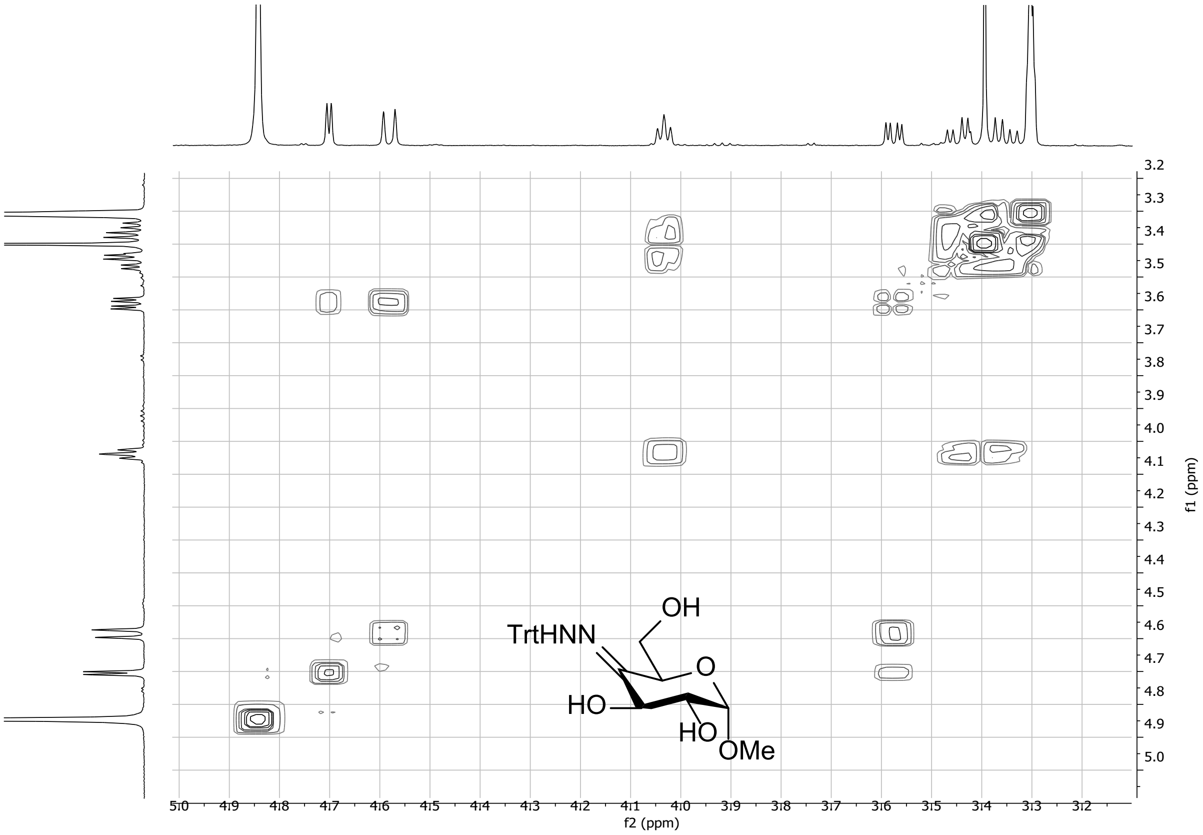

^{1}H-^{13}C HSQC of compound **2c**

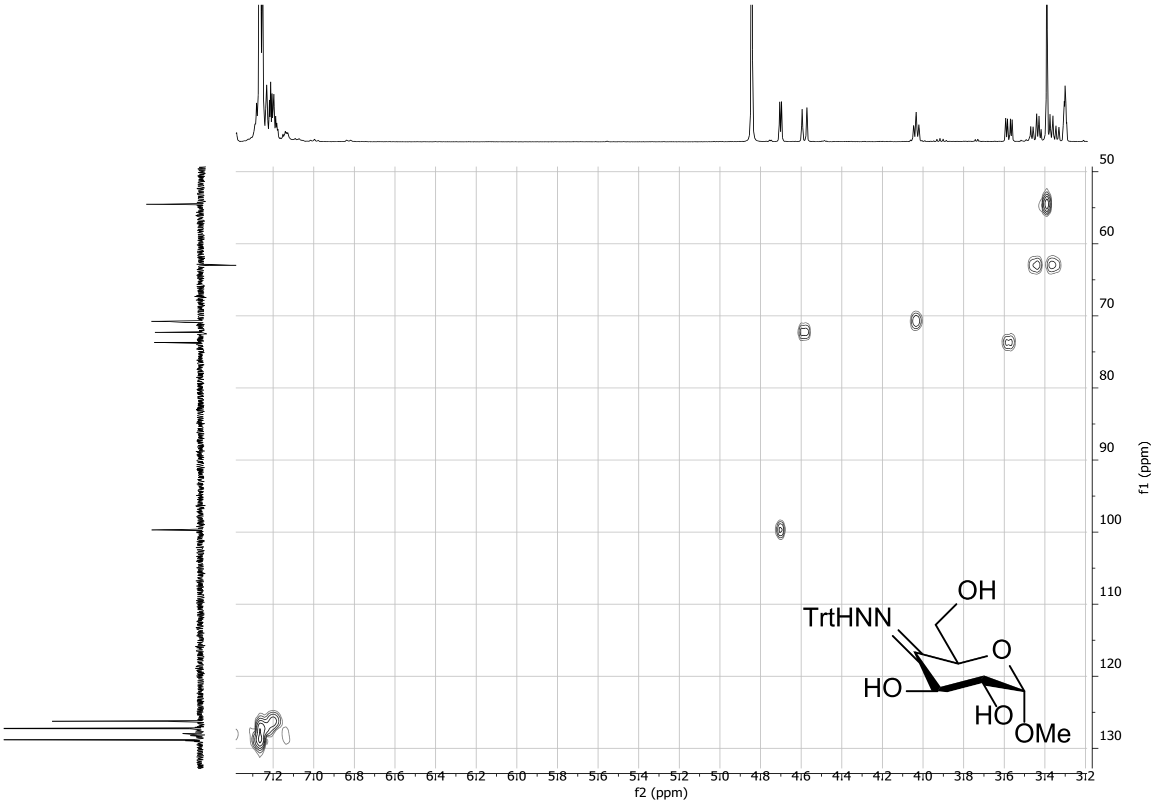

Methyl 4-chloro-4-deoxy-α-D-galacto/glucopyranoside (3c)

4-equtorial : 4-axial ≈ 1 : 2.6

^{1}H NMR, 400 MHz, CD_3OD of compound **3c**

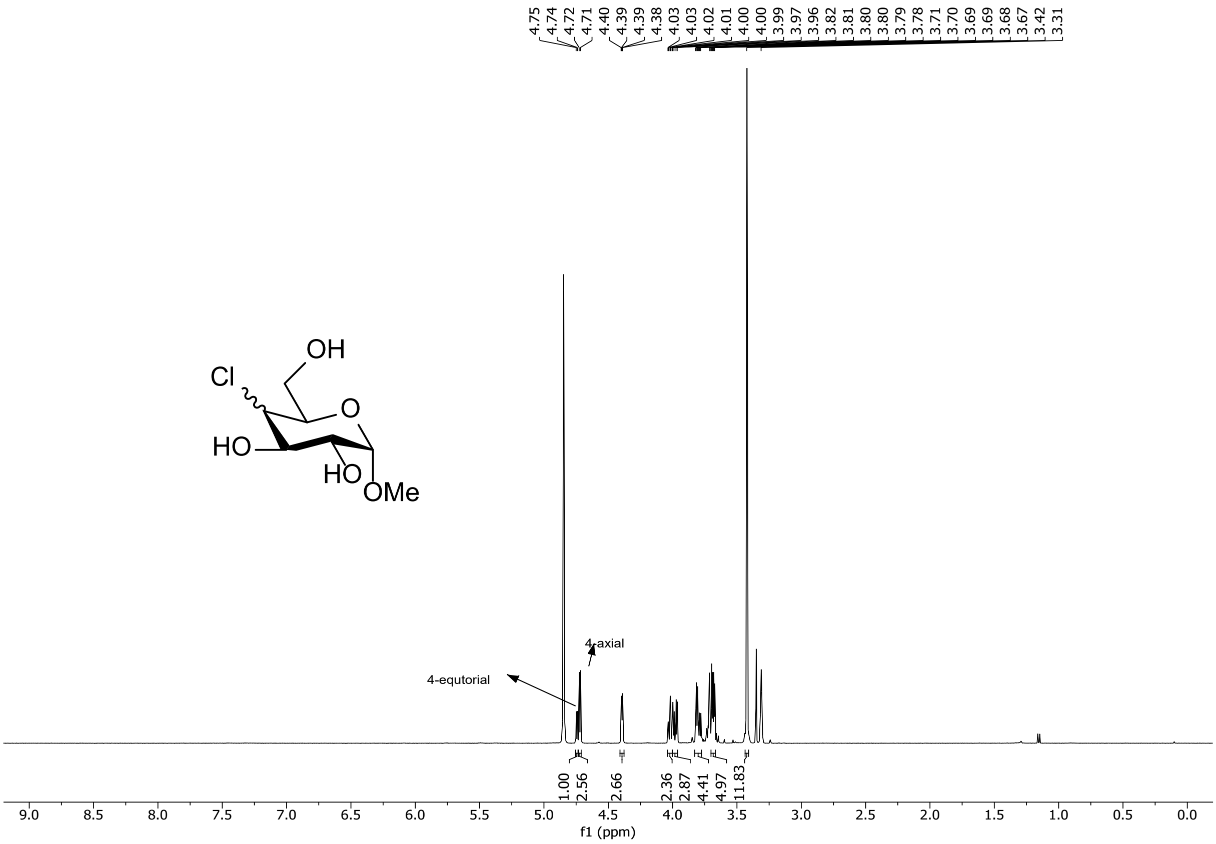

^{13}C NMR, 101 MHz, CD_3OD of compound **3c**

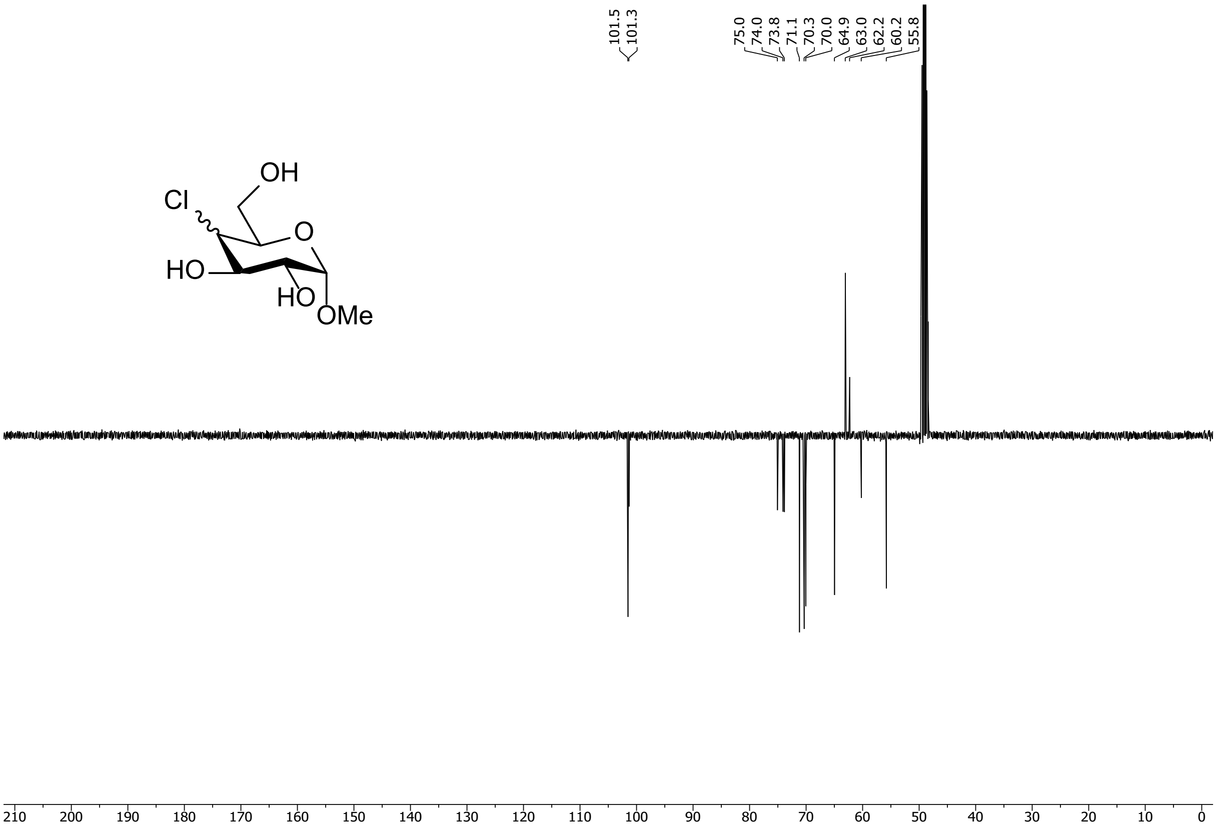

^{1}H-^{1}H COSY of compound **3c**

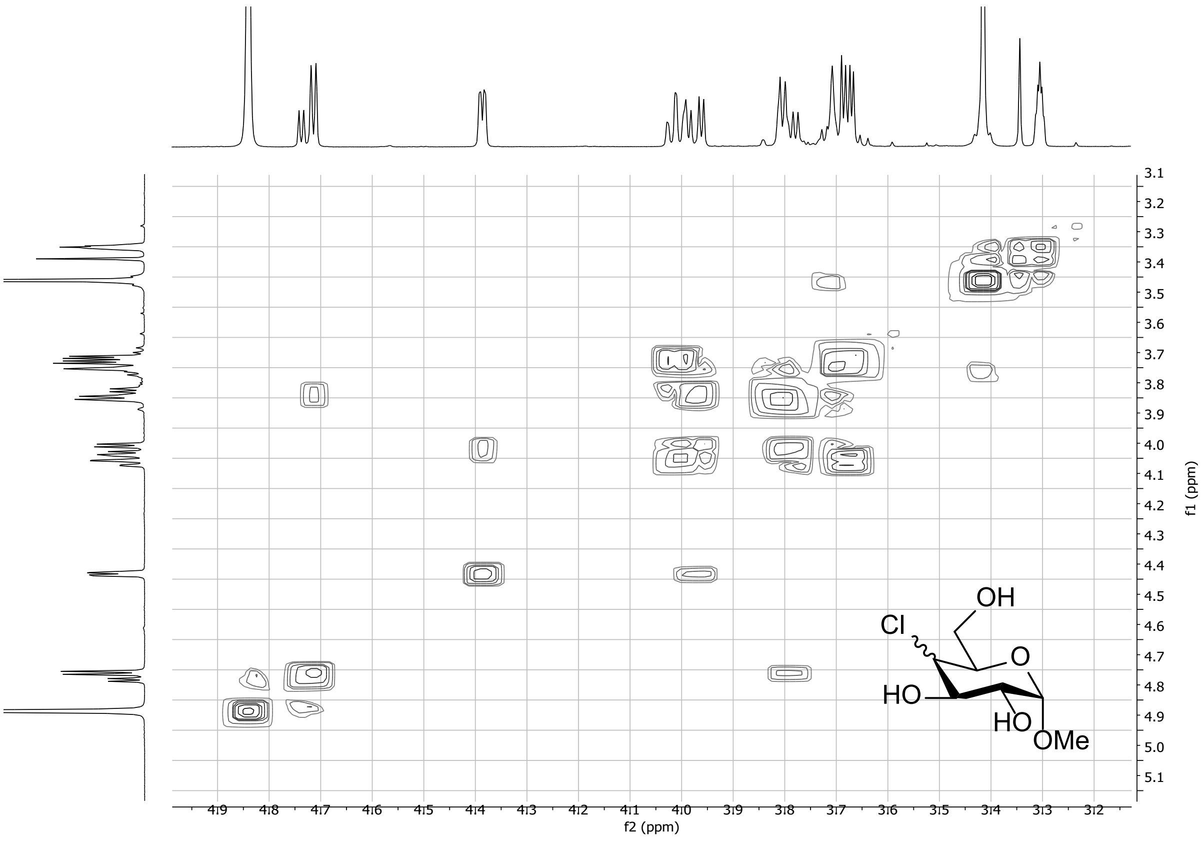

^{1}H-^{13}C HSQC of compound **3c**

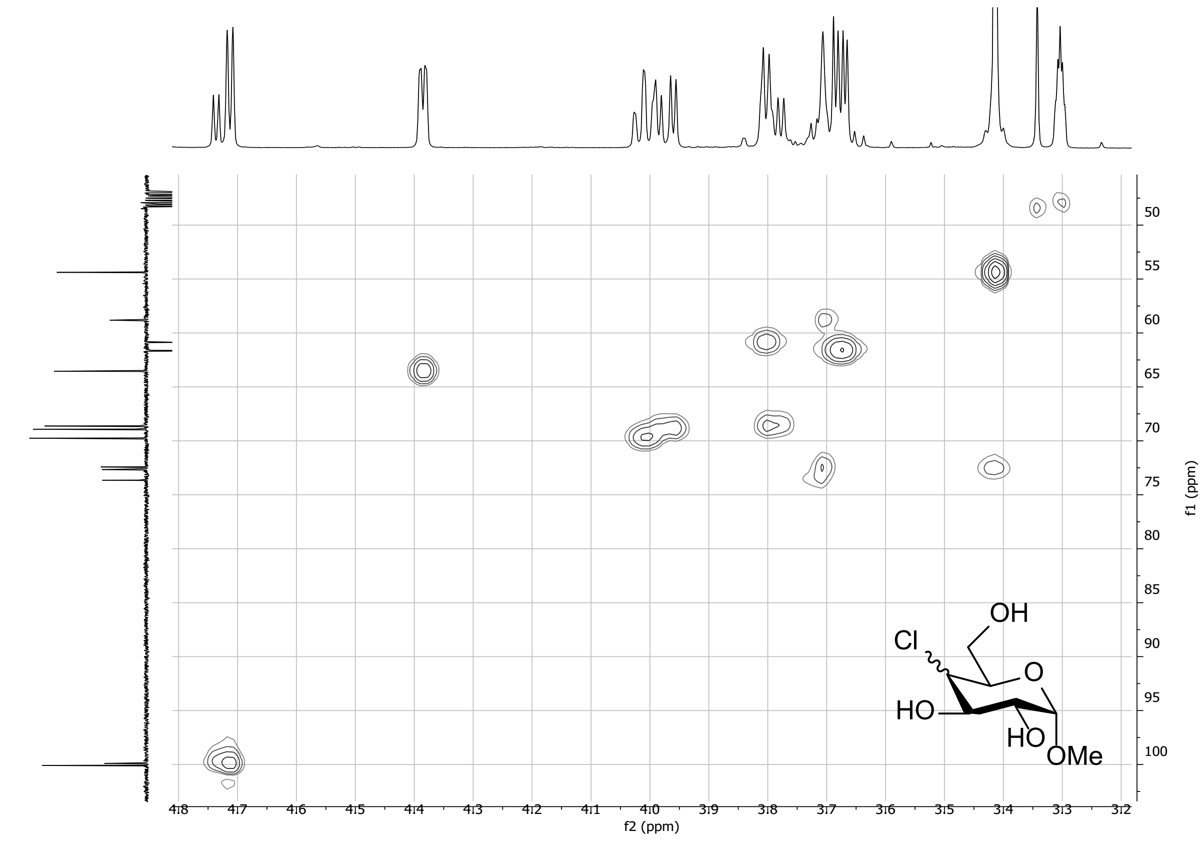

NMR spectra of Xyl derivatives

Methyl 3-keto-α-D-xylopyranoside (1d)

^{1}H NMR, 400 MHz, CD_3OD of compound **1d**

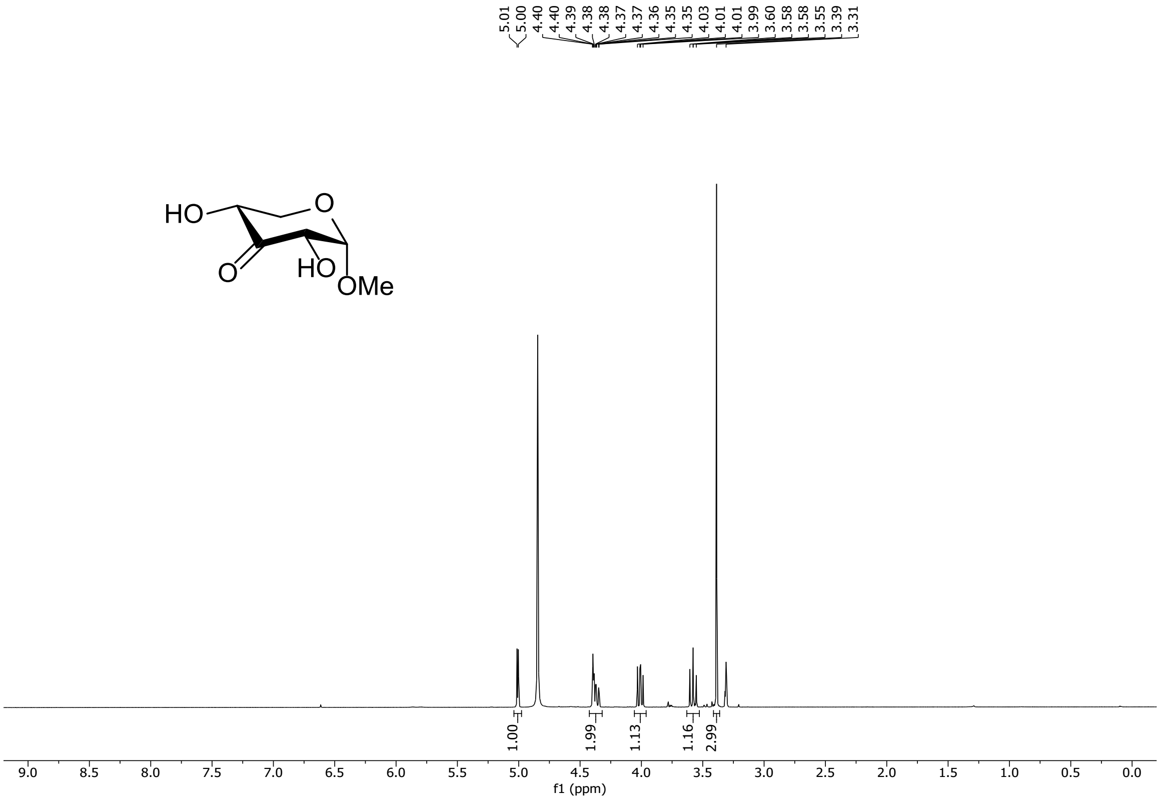

^{13}C-APT NMR, 101 MHz, CD_3OD of compound **1d**

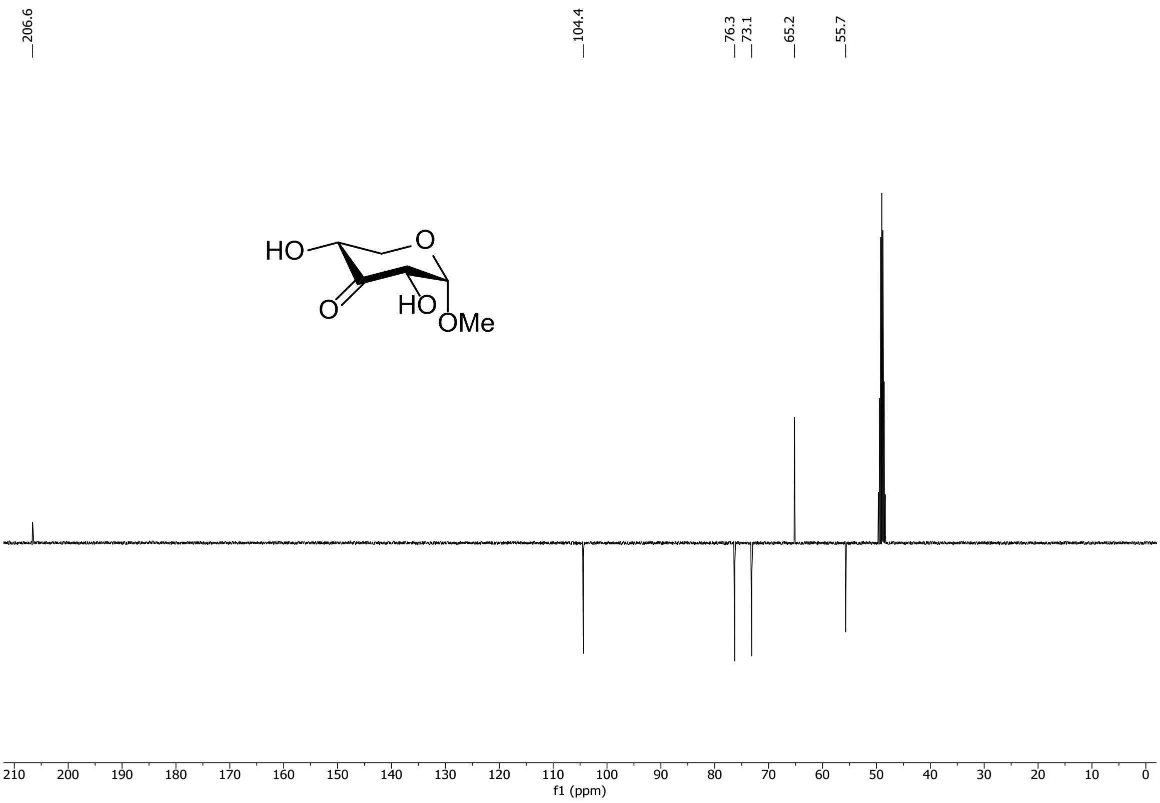

^1H-^1H COSY of compound **1d**

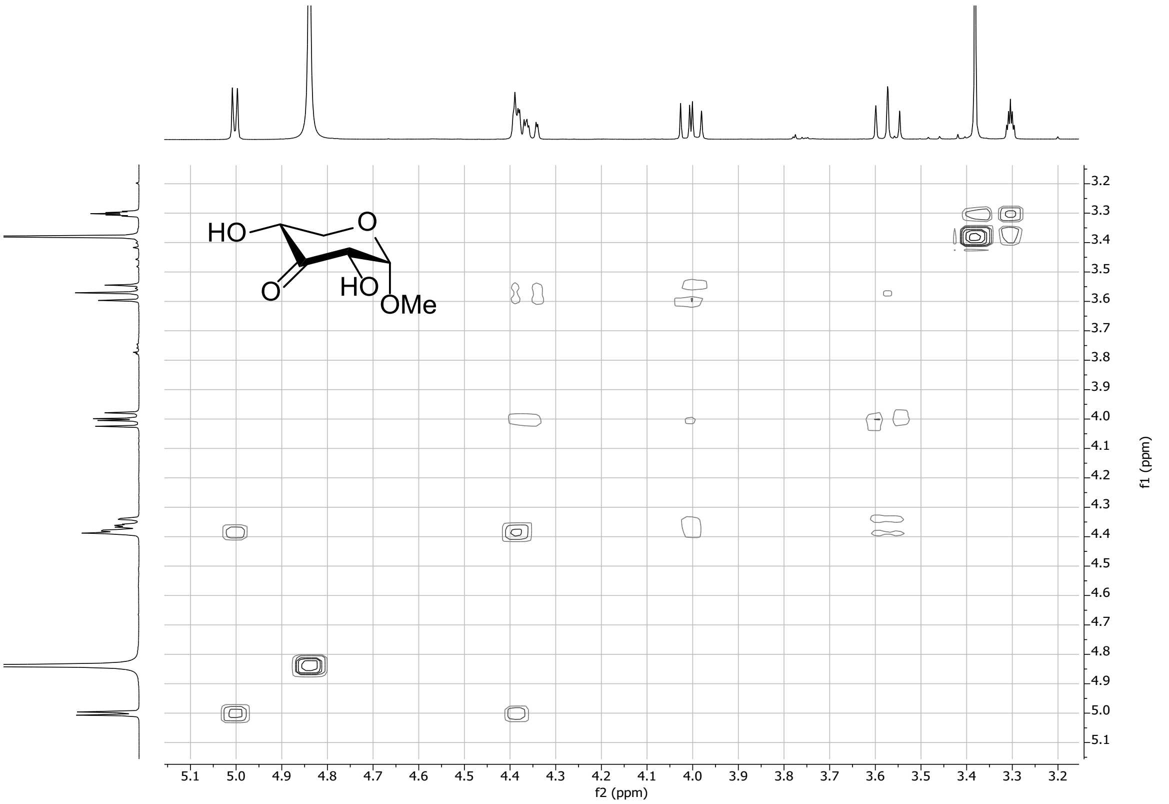

^1H-^{13}C HSQC of compound **1d**

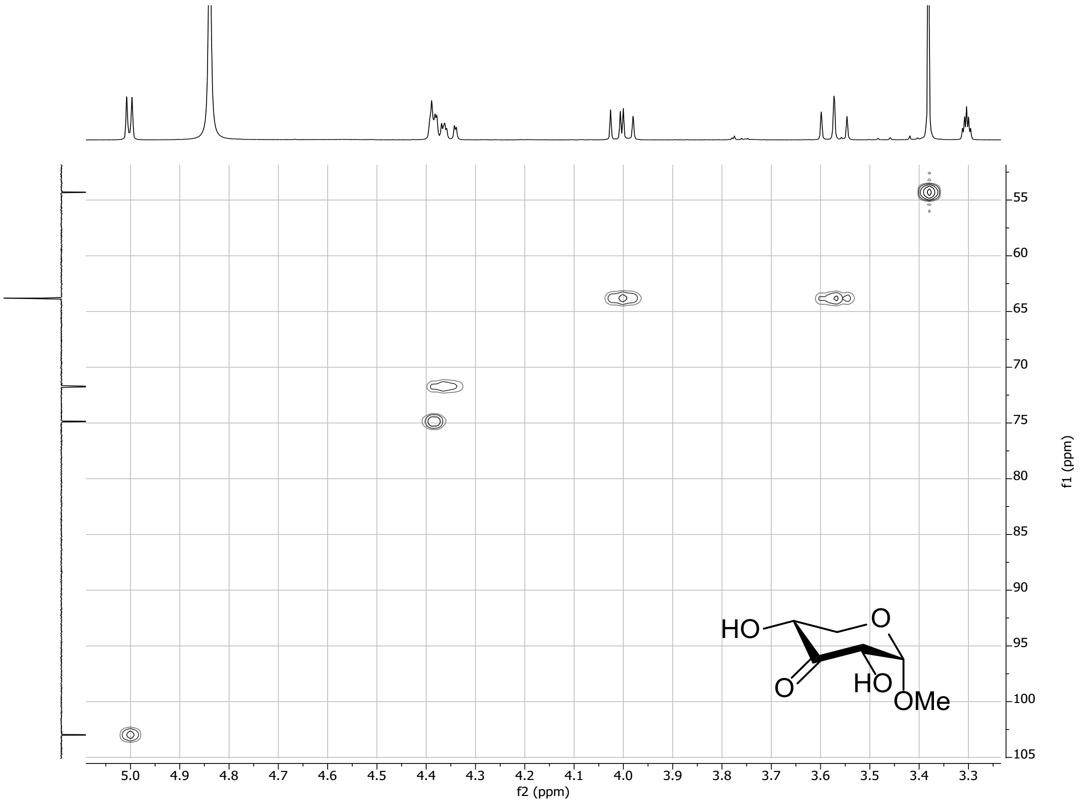

Methyl 3-(trityl)hydrazone-α-D-xylopyranoside (2d)

Mixture of *E* and *Z*: ratio ≈ 0.6:1

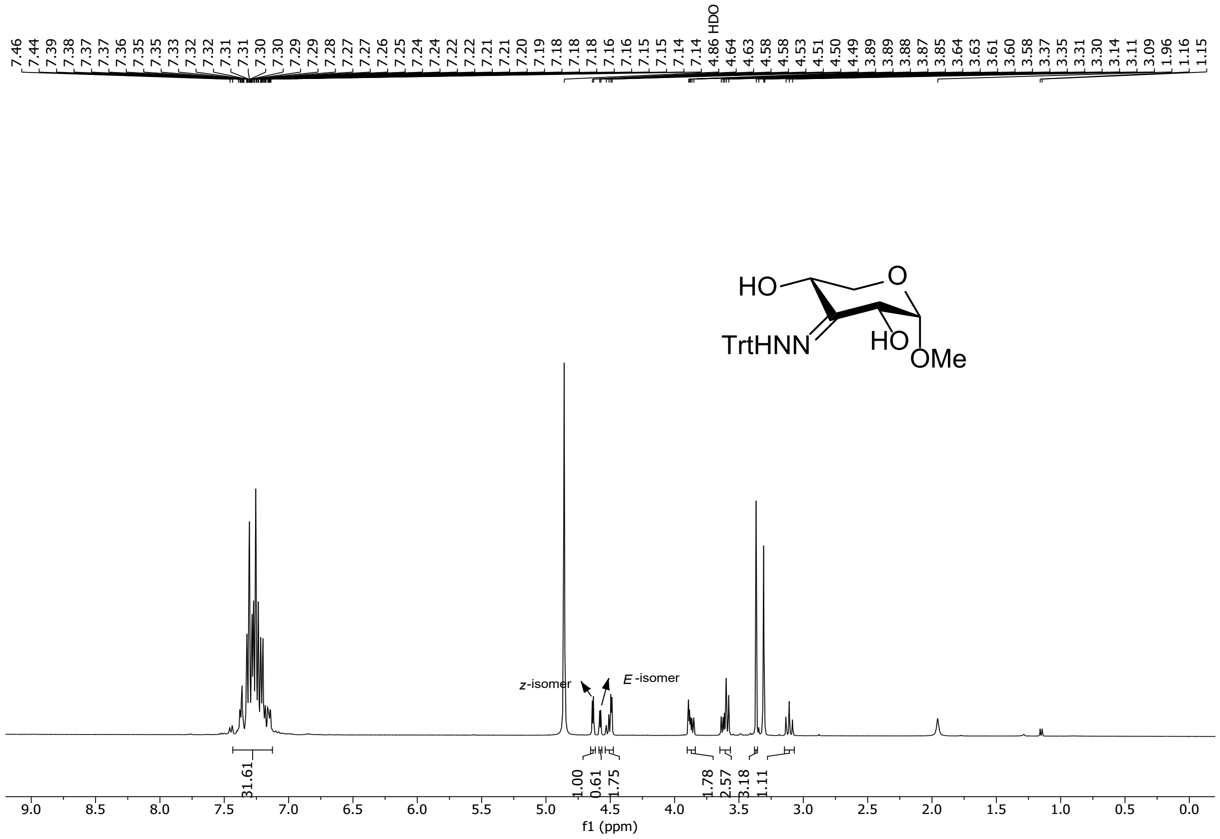

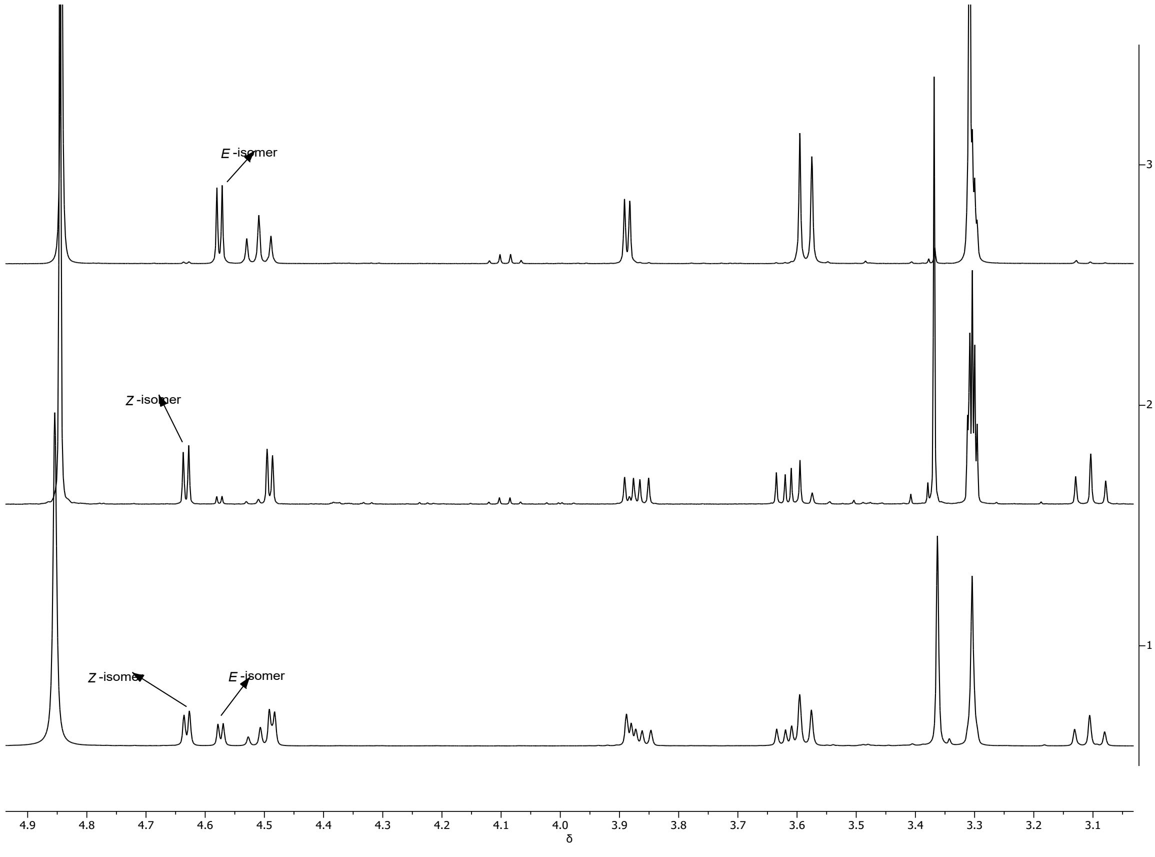

^{1}H NMR, 400 MHz, CD_3OD of compound **2d**: Z-isomer

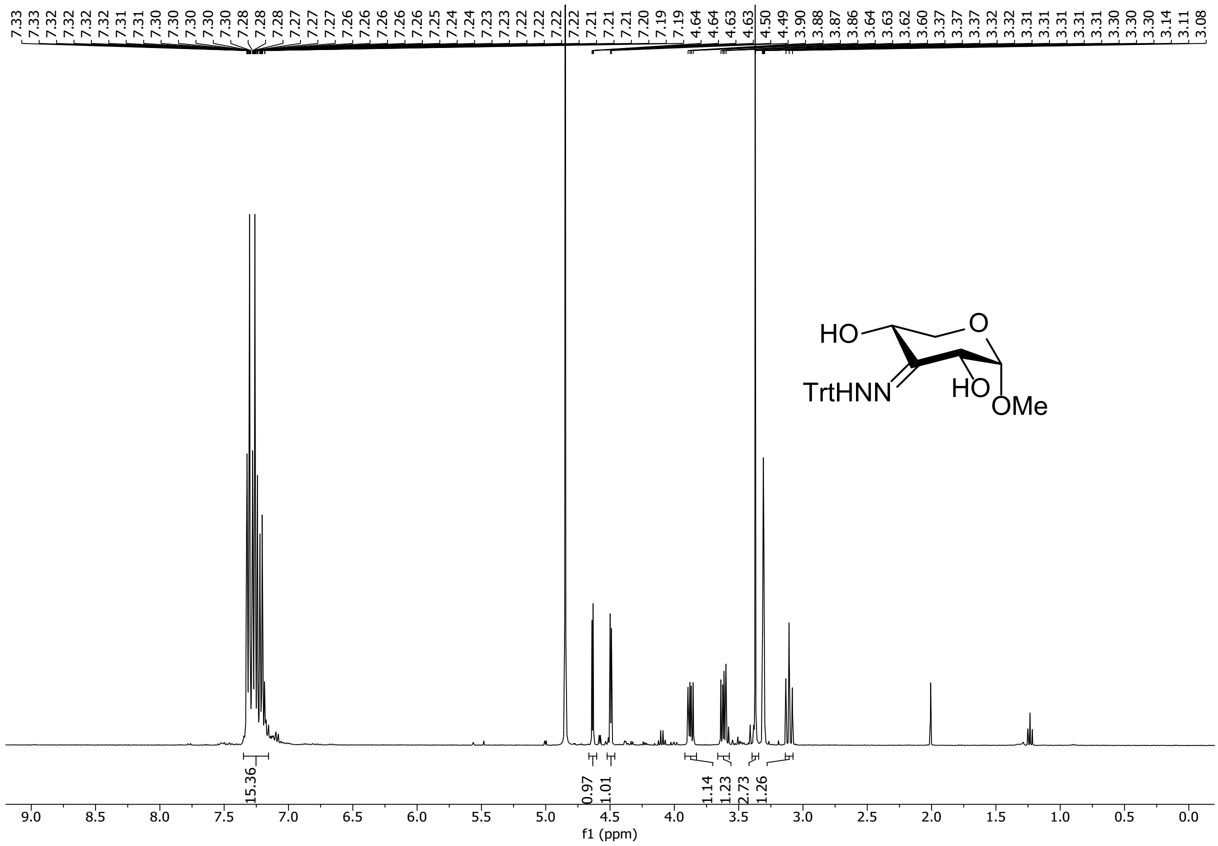

^{13}C-APT NMR, 101 MHz, CD_3OD of compound **2d**: Z-isomer

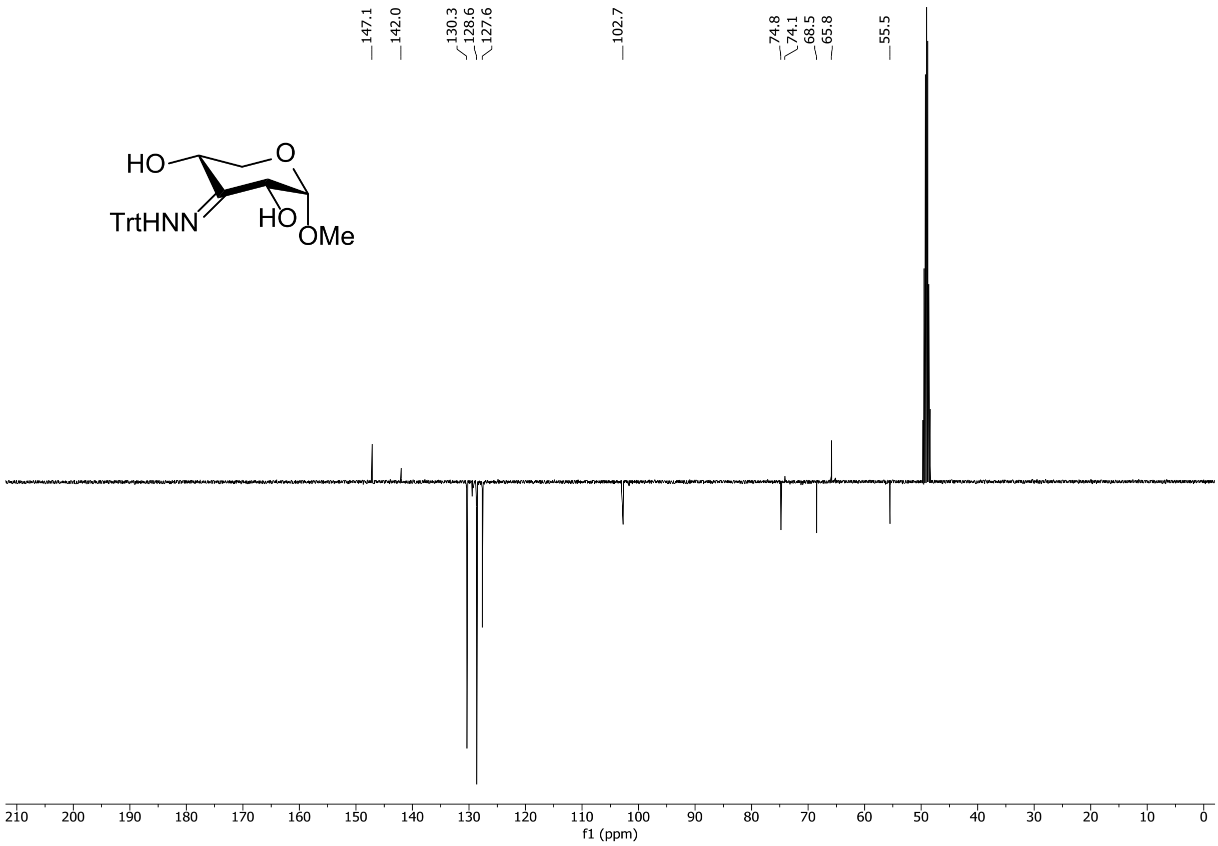

^1H-^1H COSY of compound **2d**: Z-isomer

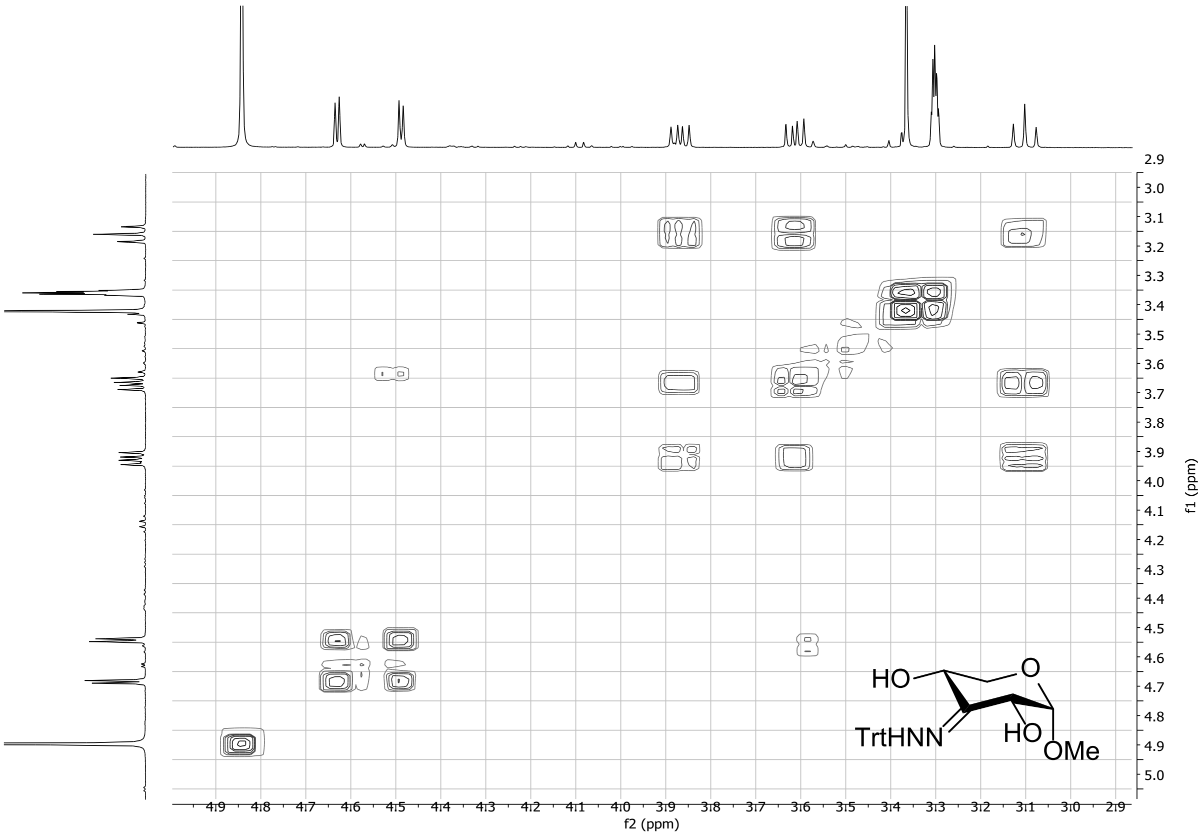

^1H-^{13}C HSQC of compound **2d**: Z-isomer

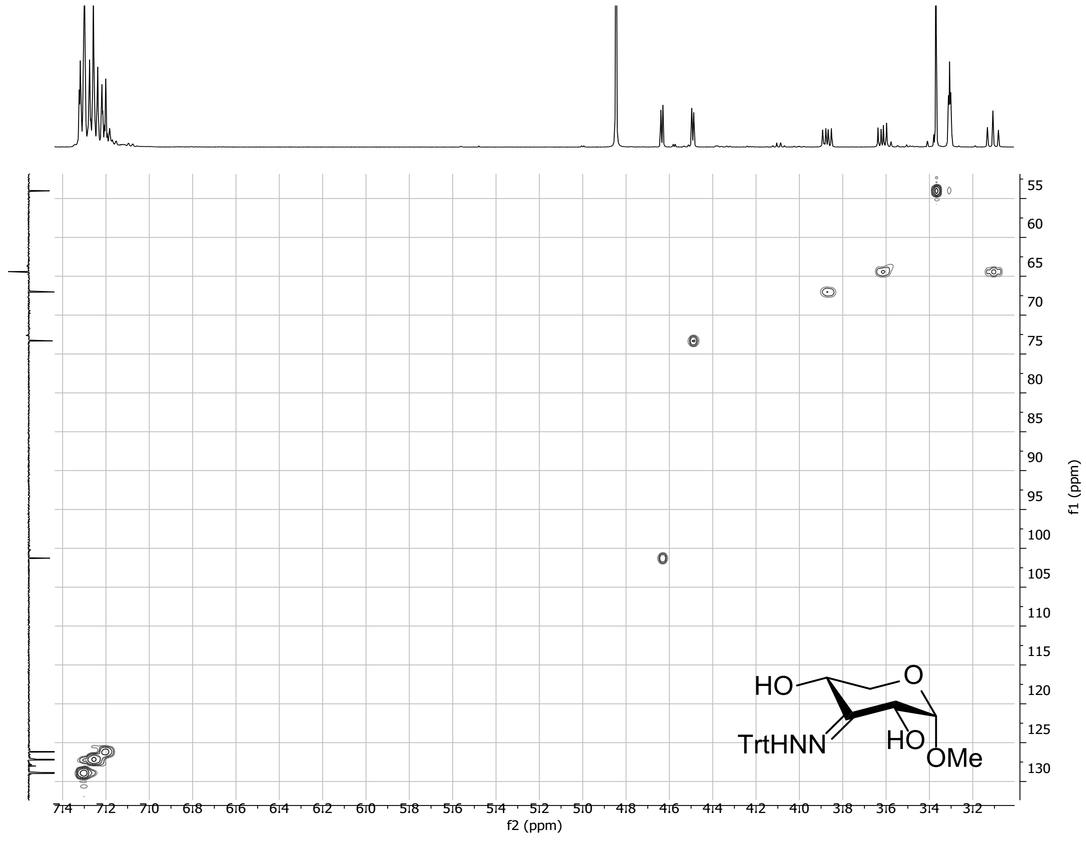

^{1}H NMR, 400 MHz, CD_3OD of compound **2d**: Z-isomer

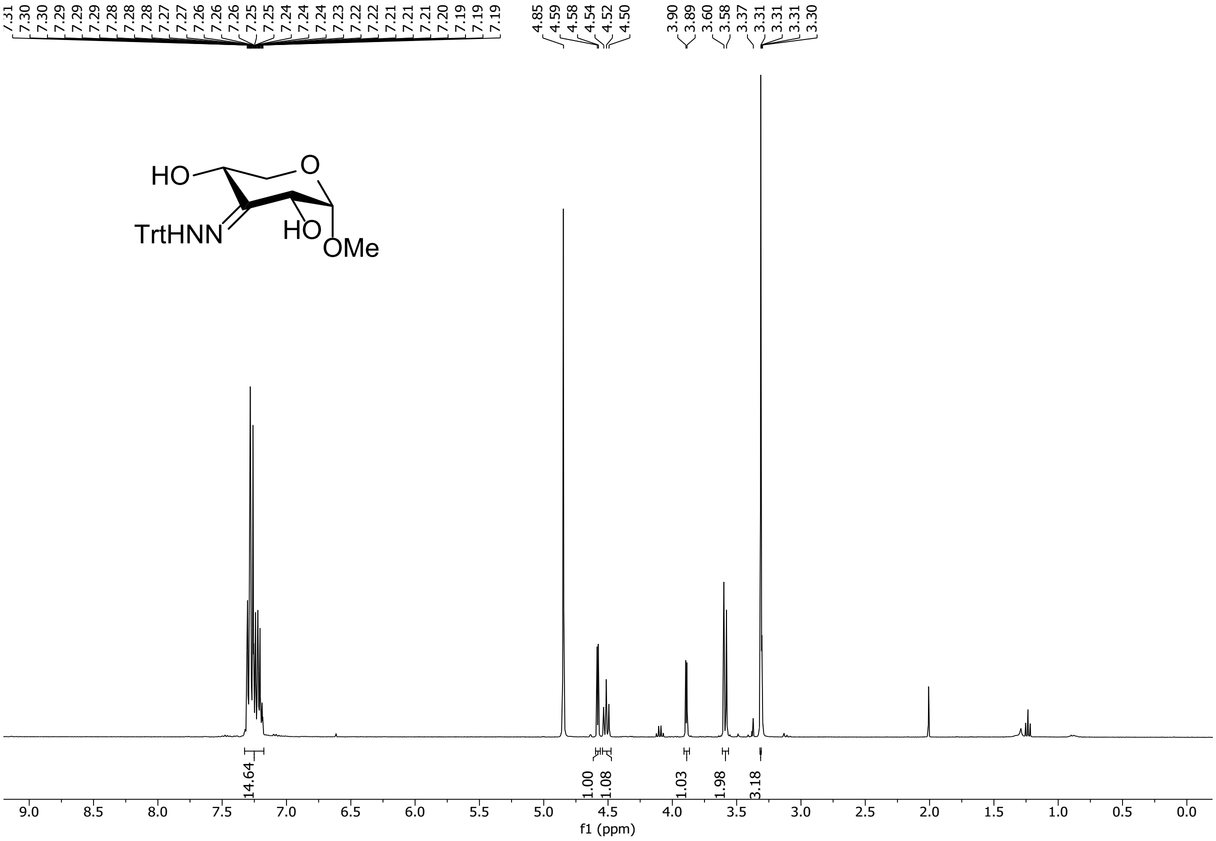

^{13}C-APT NMR, 101 MHz, CD_3OD of compound **2d**: Z-isomer

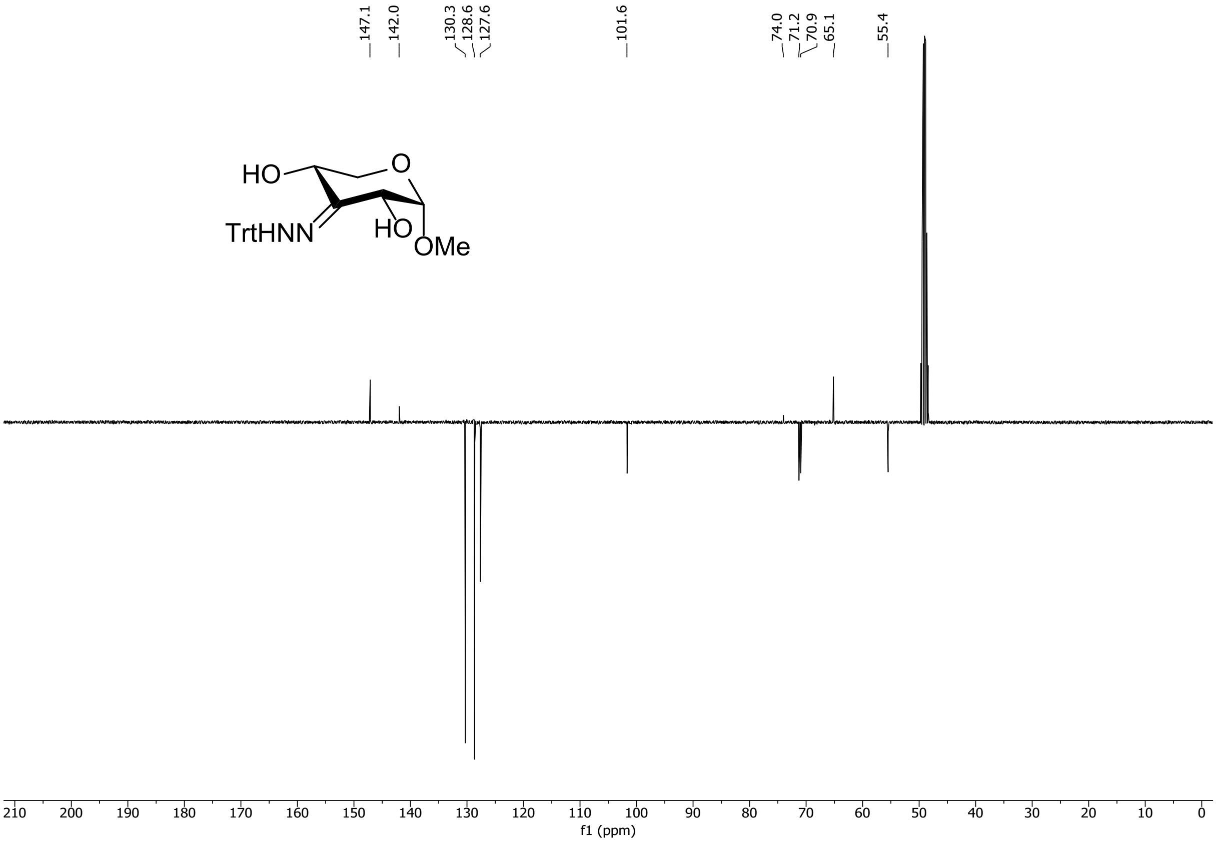

^{1}H-^{1}H COSY of compound **2d**: Z-isomer

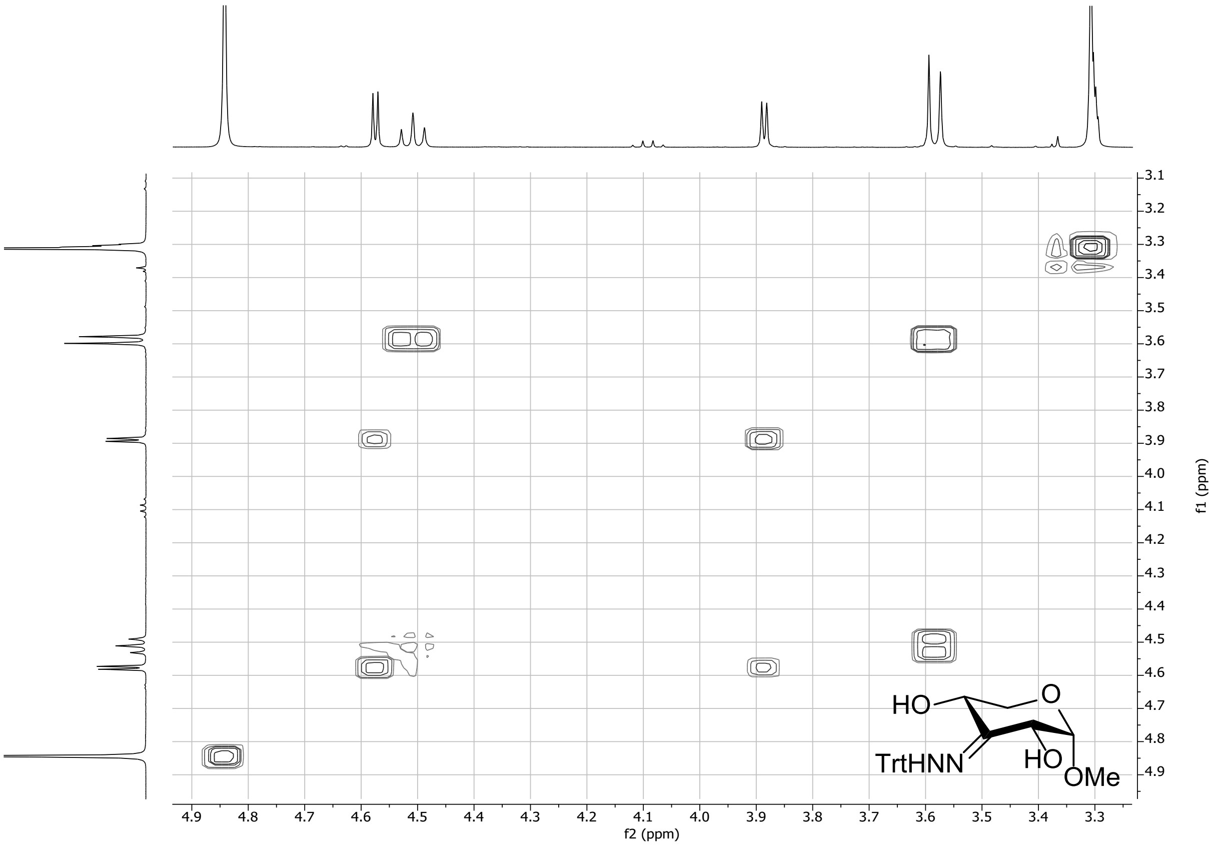

^{1}H-^{13}C HSQC of compound **2d**: Z-isomer

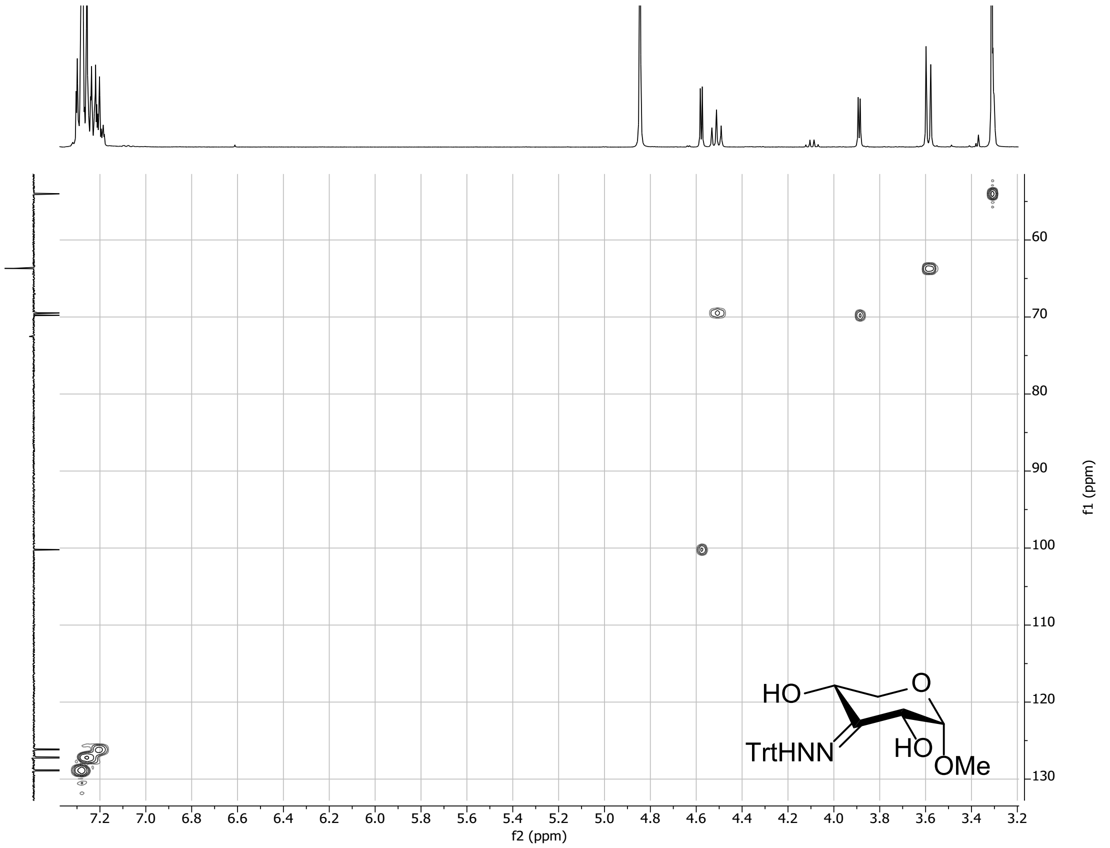

Methyl 3-chloro-3-deoxy-α-D-ribose/xylopyranoside (3d)

^{1}H NMR, 400 MHz, CD$_3$OD of compound **3d**: 3-equatorial

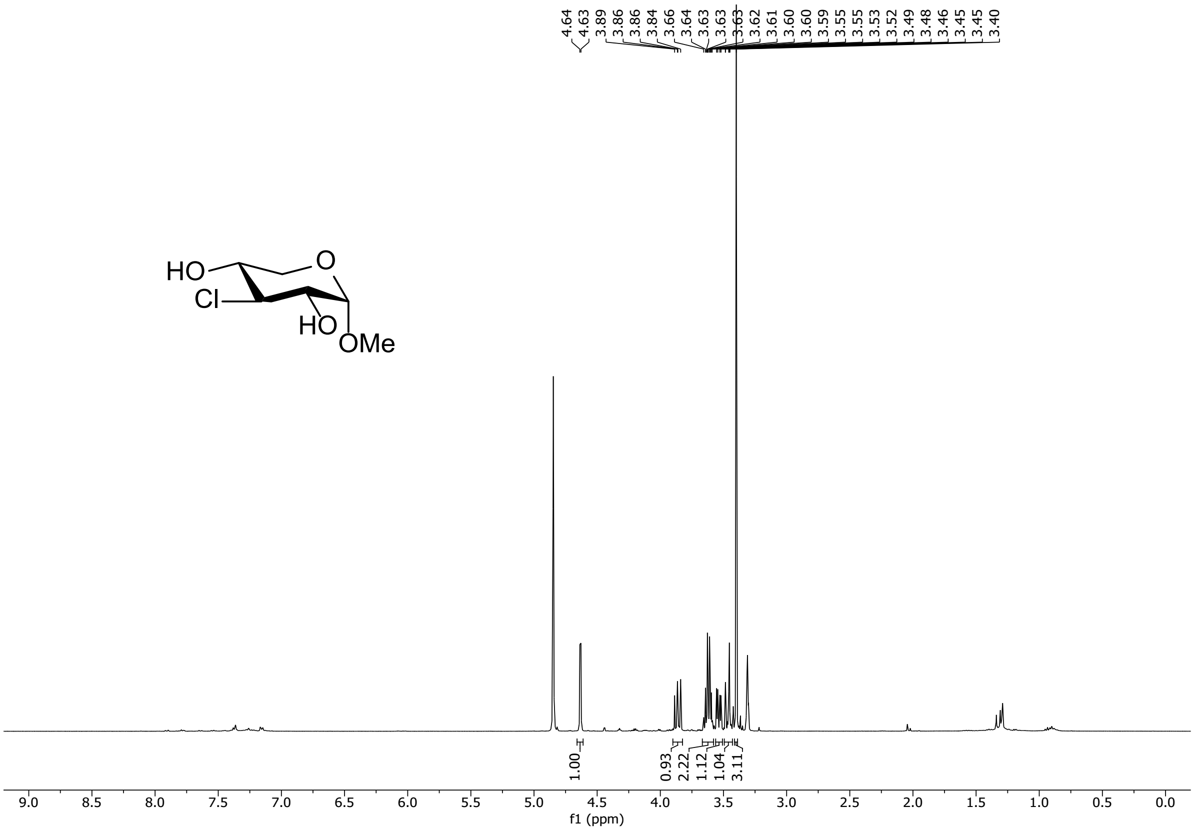

^{13}C NMR, 101 MHz, CD$_3$OD of compound **3d**: 3-equatorial

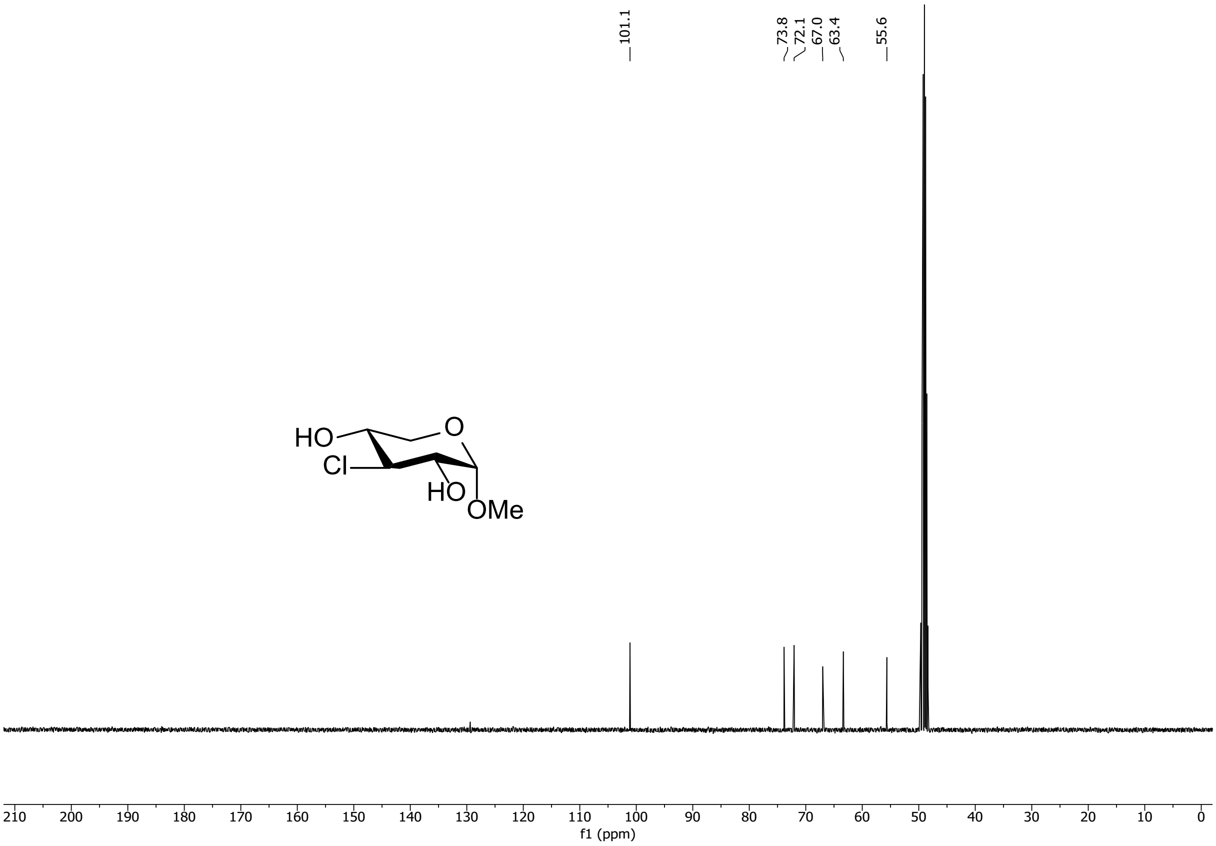

^{1}H-^{1}H COSY of compound **3d**: 3-equatorial

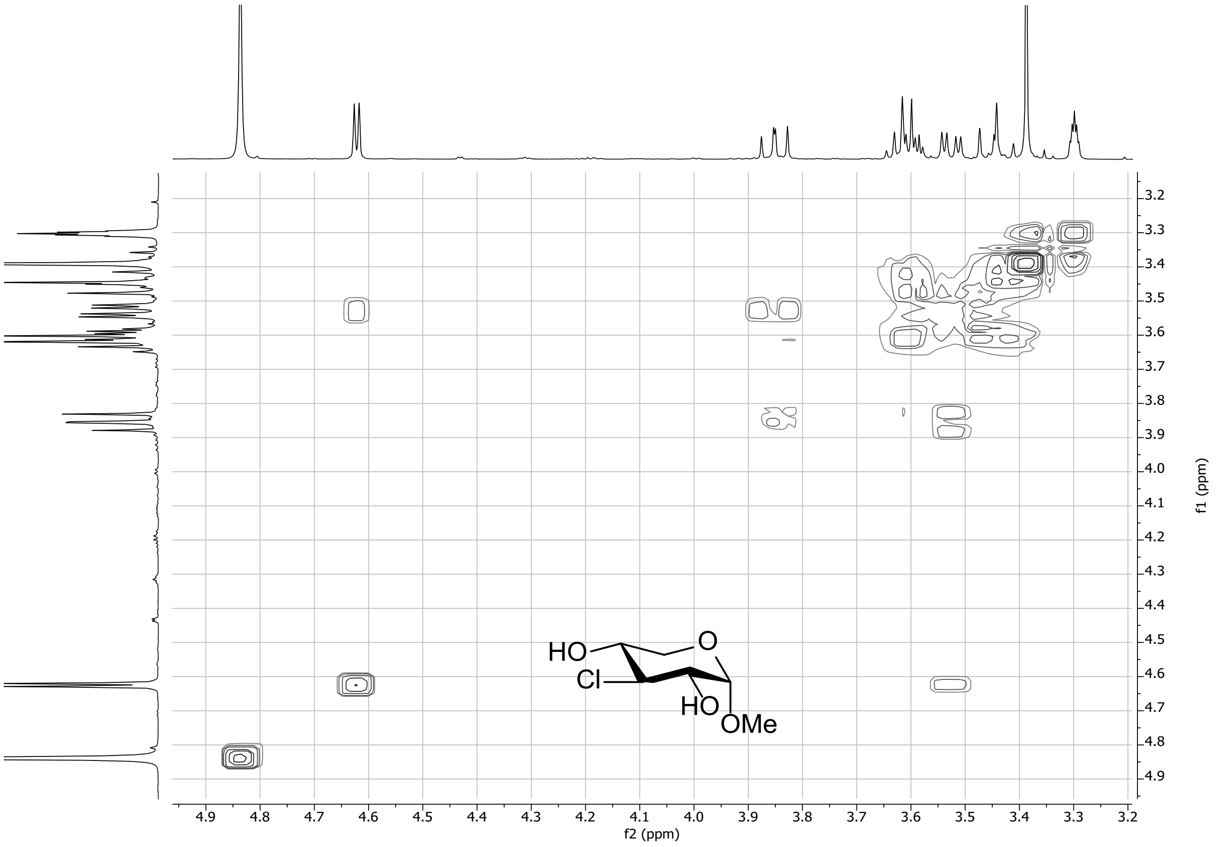

^{1}H-^{13}C HSQC of compound **3d**: 3-equatorial

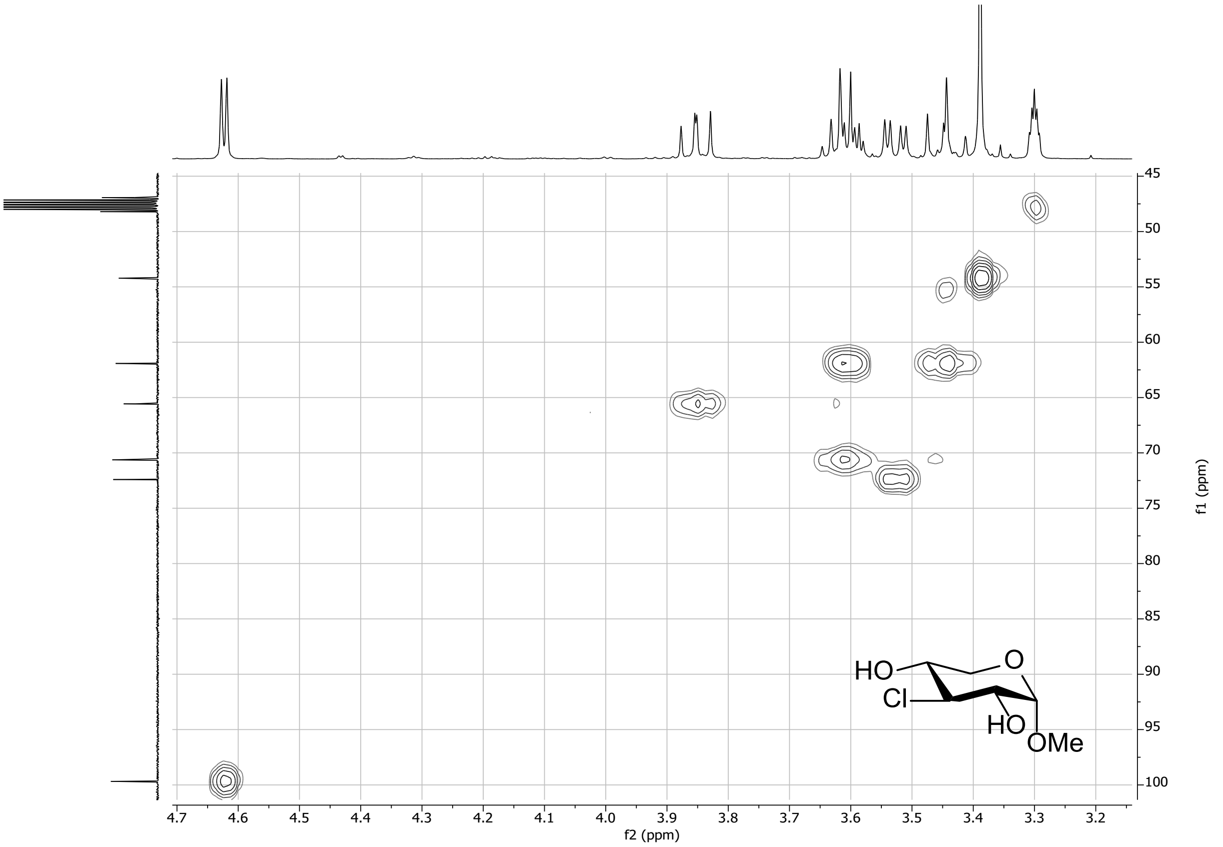

^{1}H NMR, 400 MHz, CD_3OD of compound **3d**: 3-axial

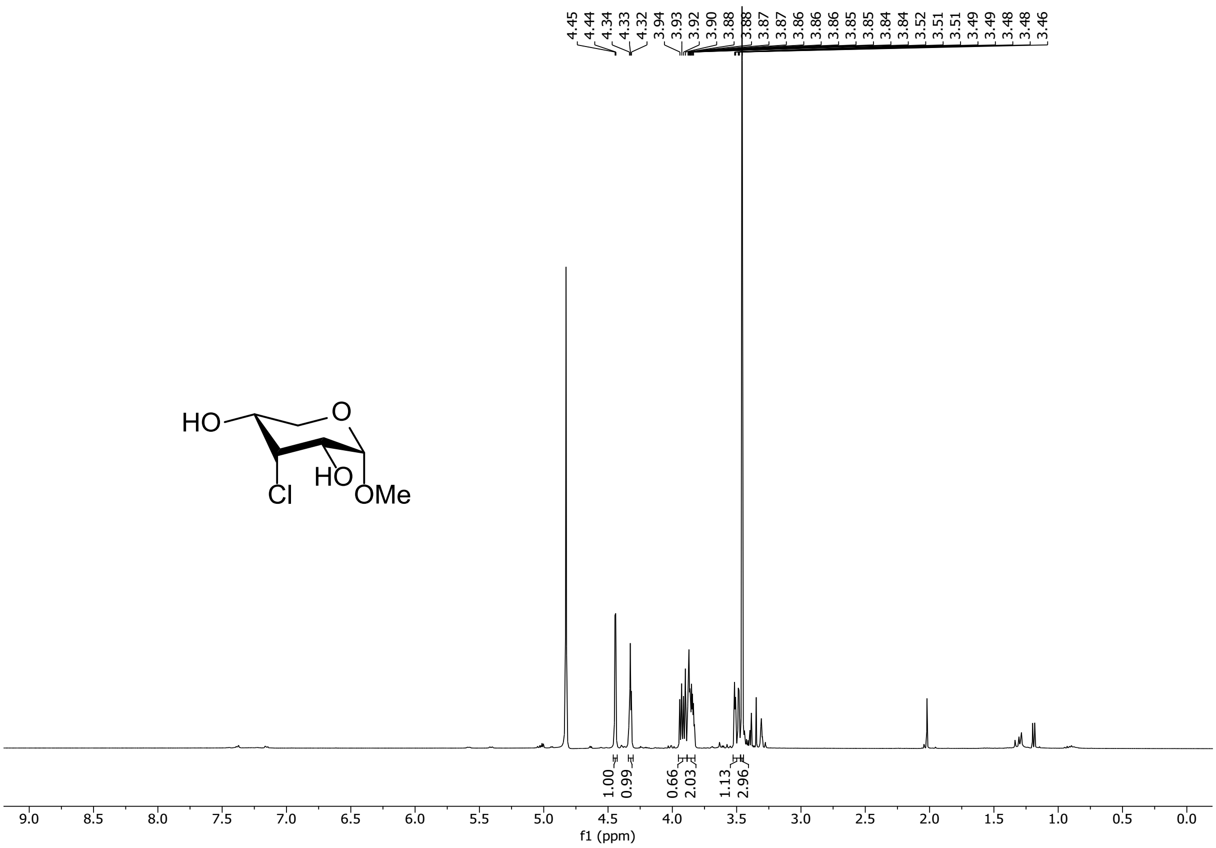

^{13}C NMR, 400 MHz, CD_3OD of compound **3d**: 3-axial

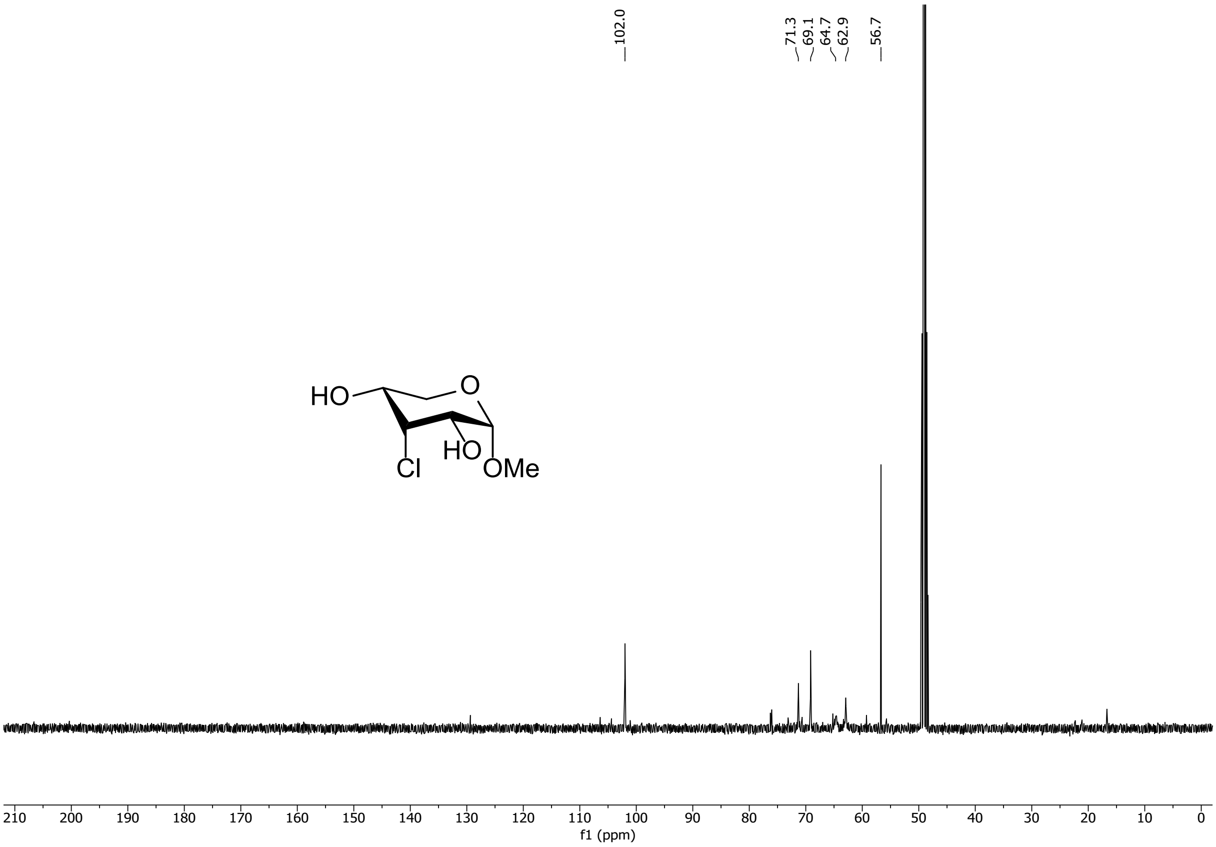

^{1}H-^{1}H COSY of compound **3d**: 3-axial

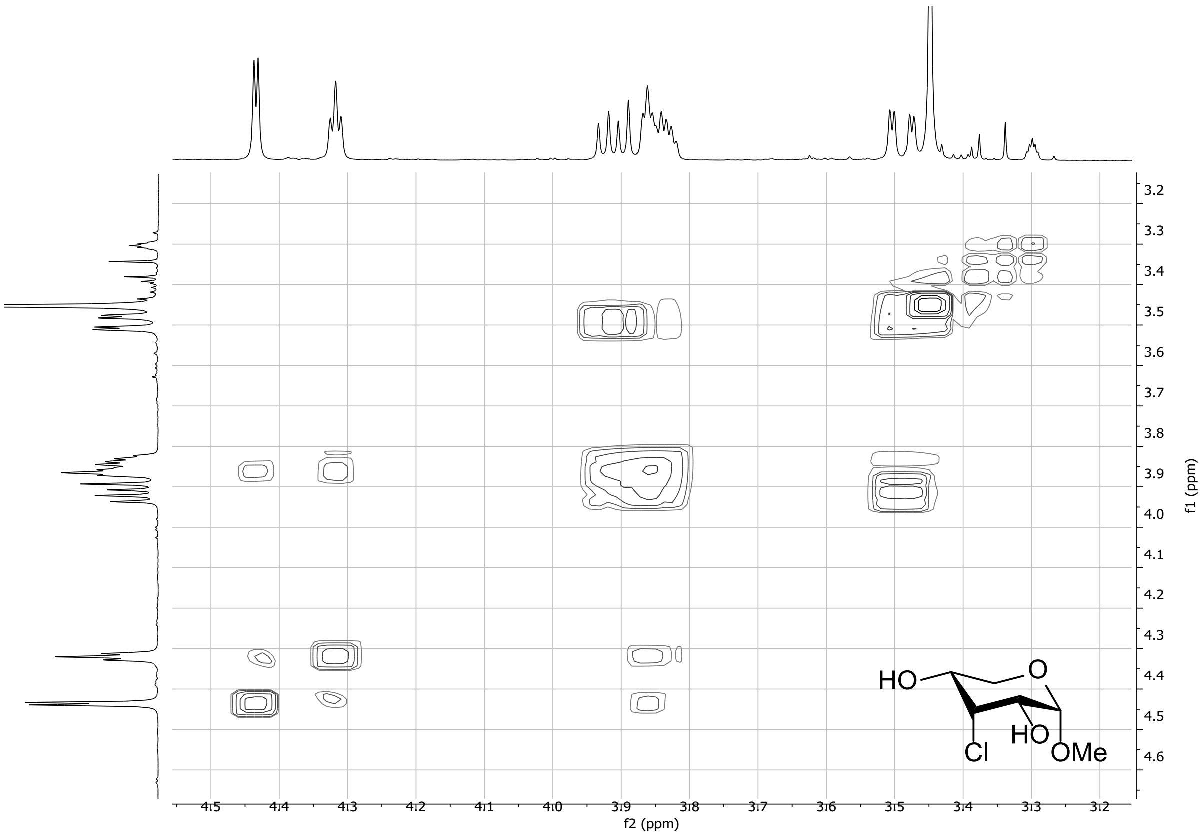

^{1}H-^{13}C HSQC of compound **3d**: 3-axial

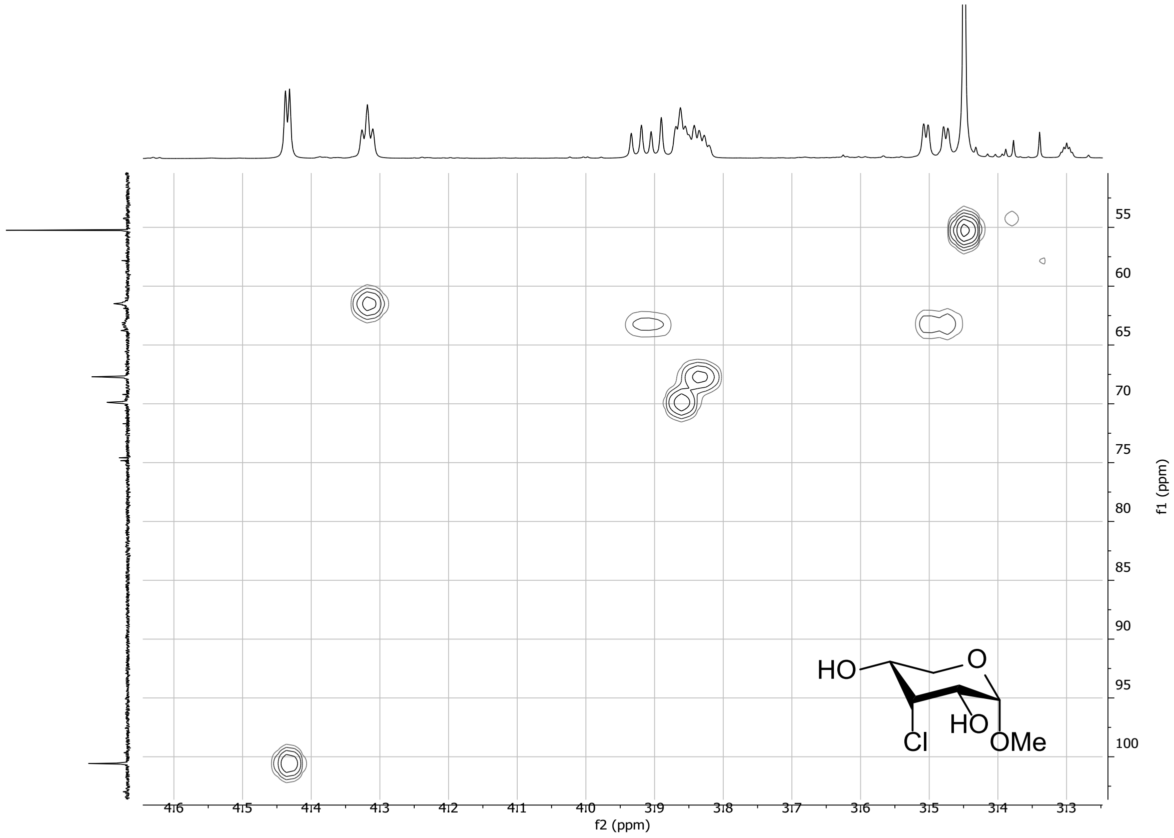

NMR spectra of cellobiose derivatives

2,3,6,2',3',4',6'-Hepta-*O*-acetyl-cellobiose (S4)

^{1}H NMR, 400 MHz, $CDCl_3$ of compound **S4**

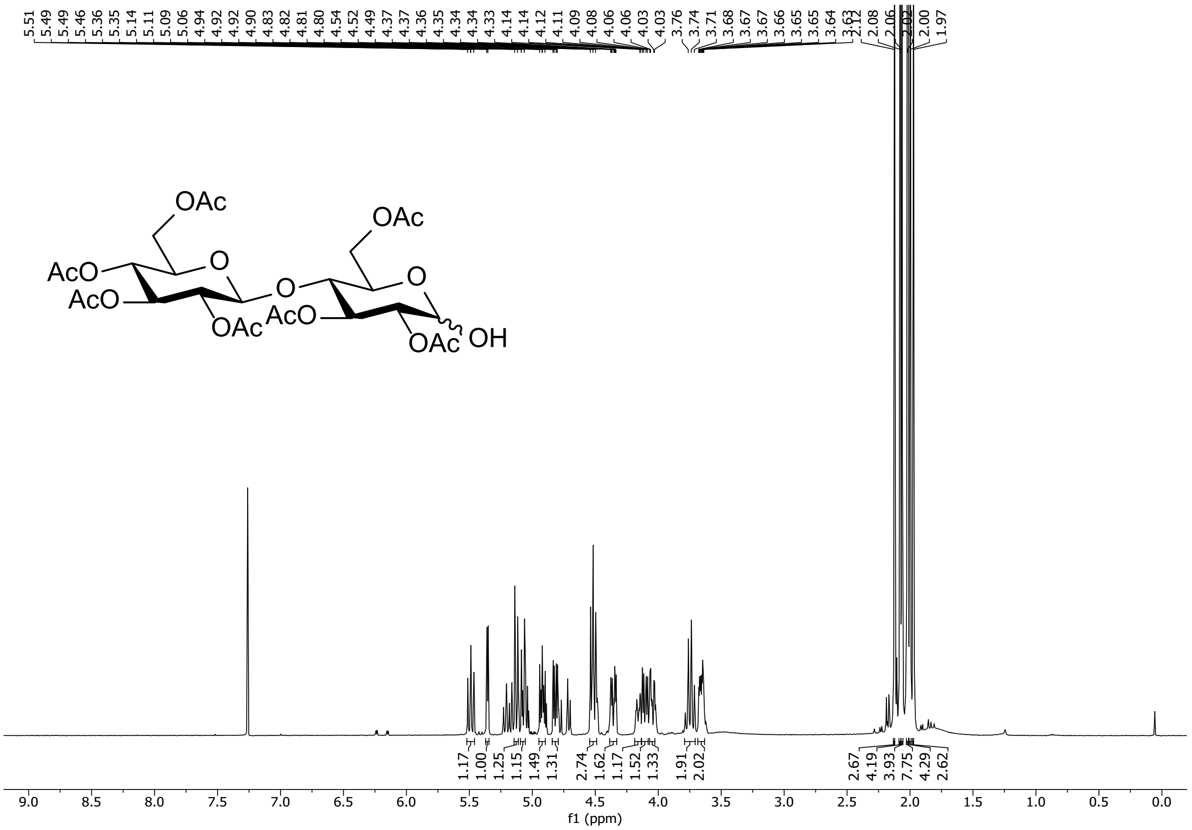

^{13}C NMR, 400 MHz, $CDCl_3$ of compound **S4**

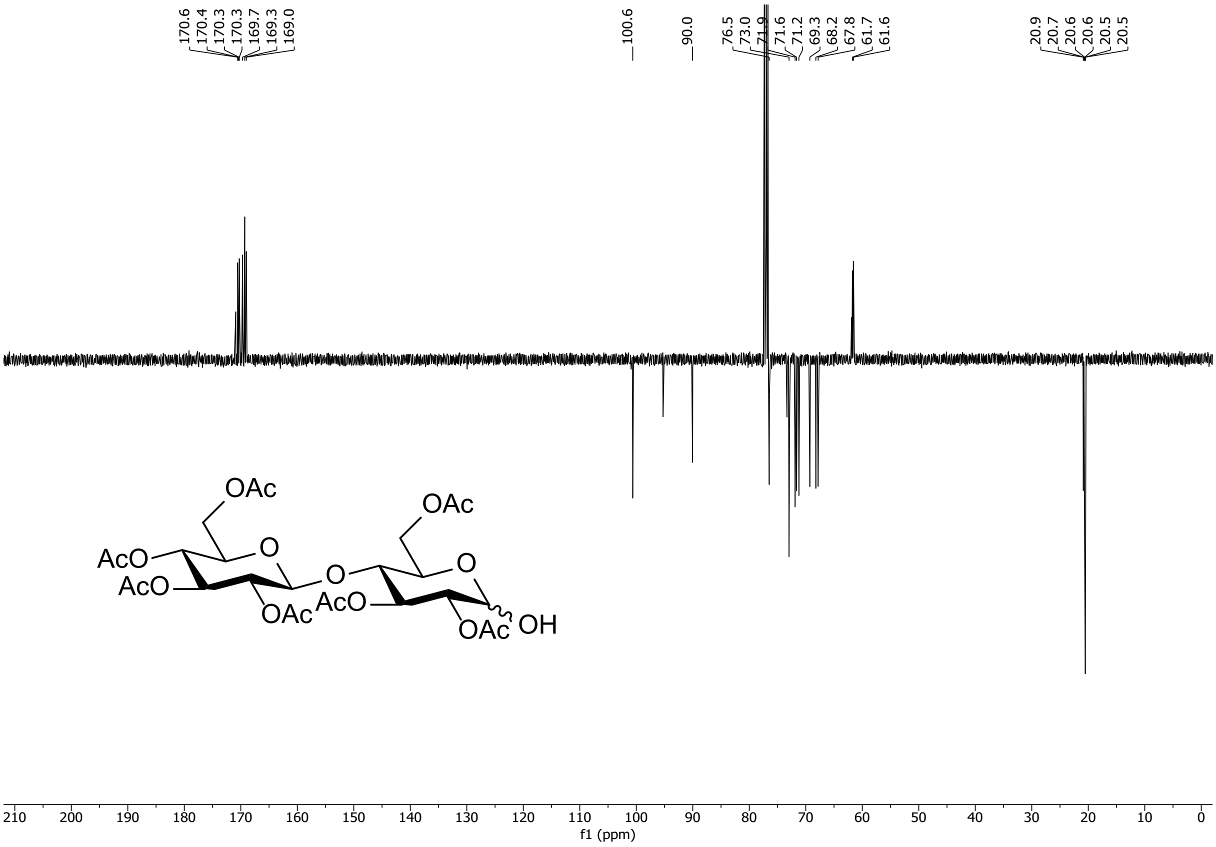

^1H-^1H COSY of compound **S4**

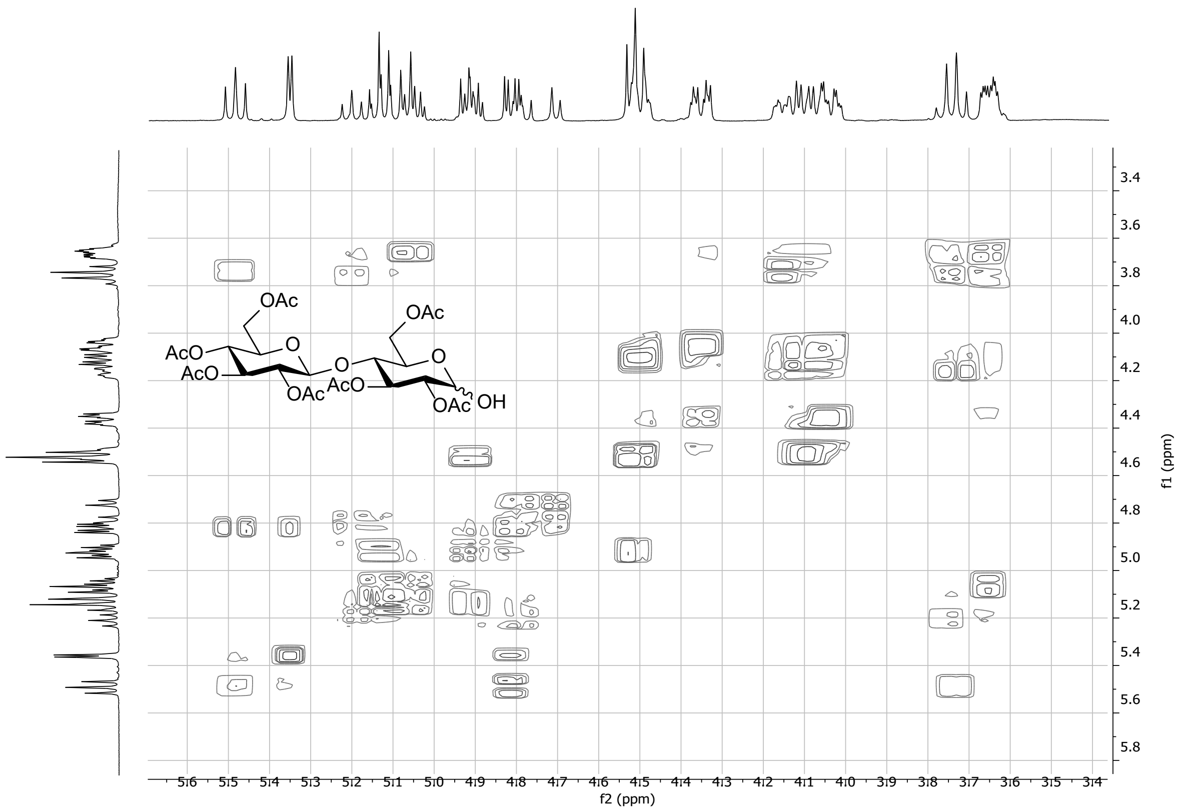

^1H-^{13}C HSQC of compound **S4**

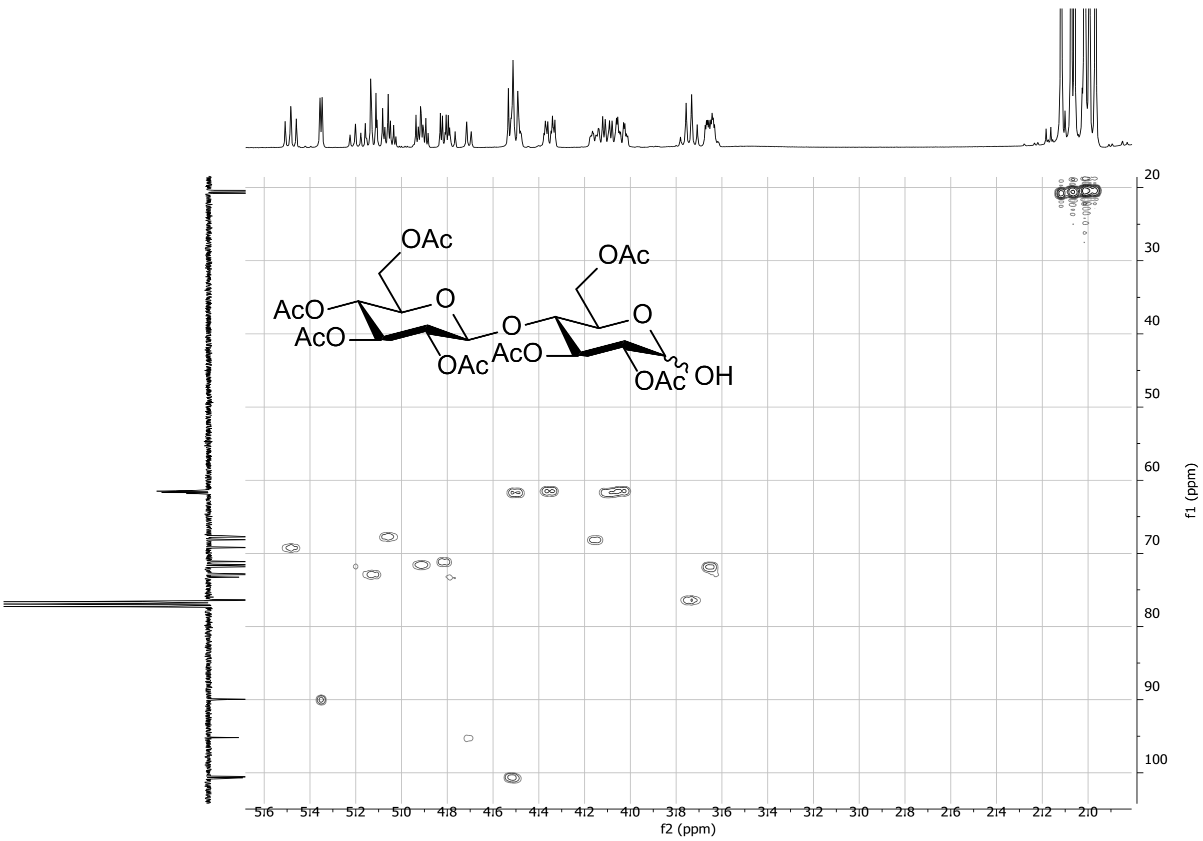

4-tert-butylbenzyl-β-D-heptaacetyl-cellobioside (S5)

^{1}H NMR, 400 MHz, $CDCl_3$ of compound **S5**

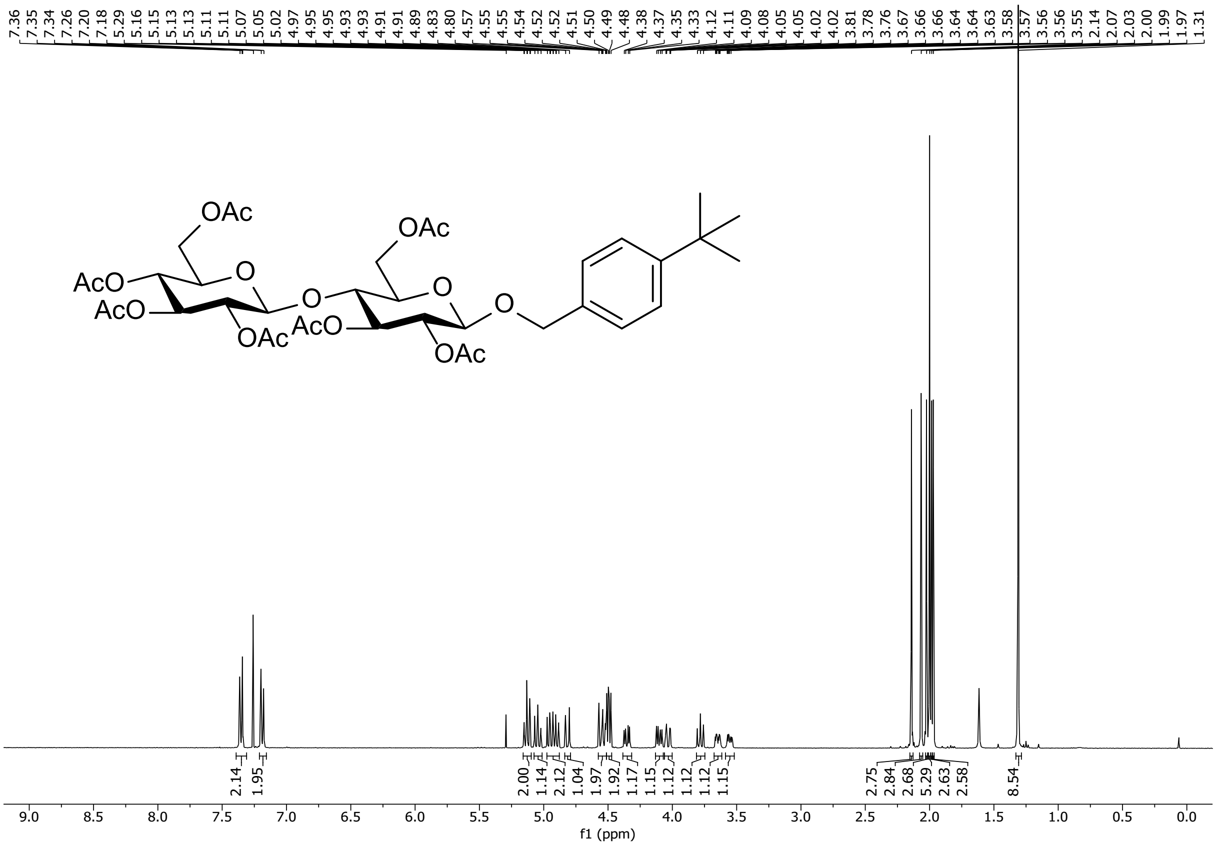

^{13}C NMR, 400 MHz, $CDCl_3$ of compound **S5**

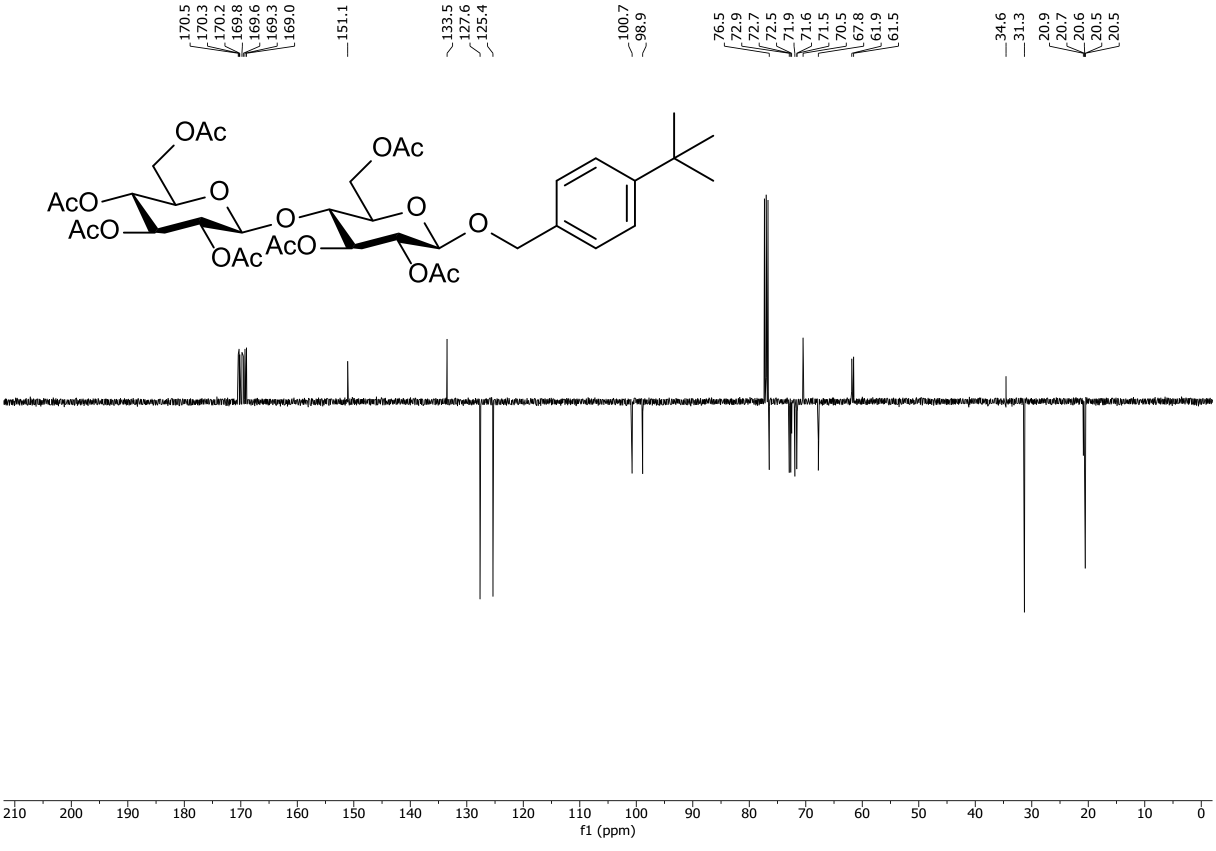

^1H-^1H COSY of compound **S5**

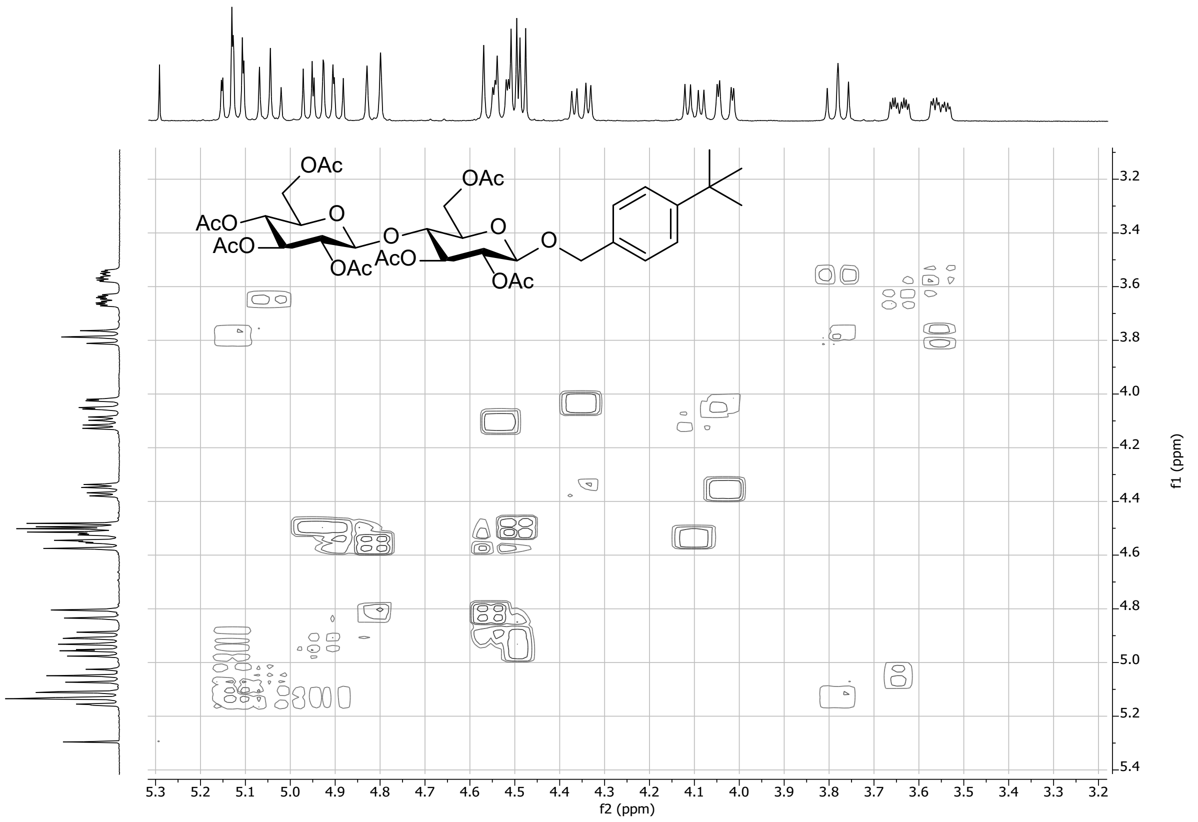

^1H-^{13}C HSQC of compound **S5**

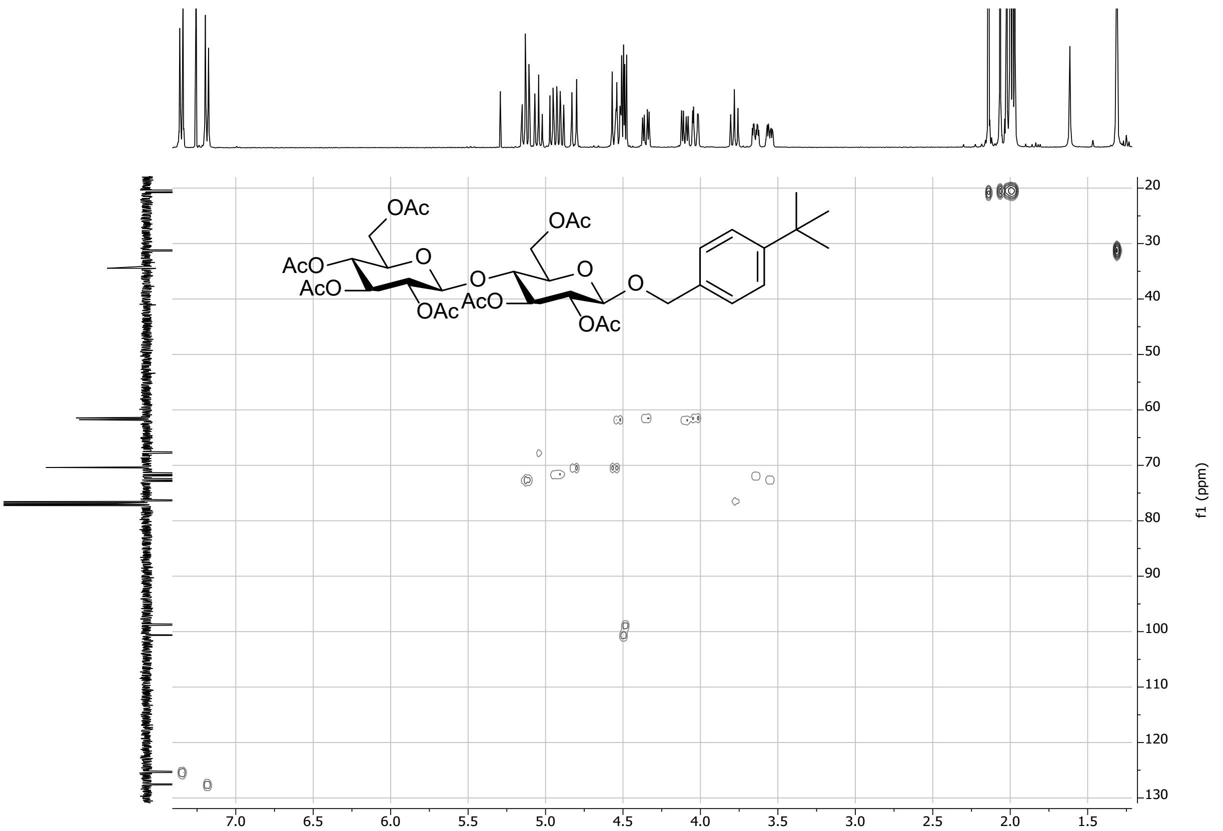

4-tert-butylbenzyl-β-D-cellobioside (S6)

^{1}H NMR, 400 MHz, CD_3OD of compound **S6**

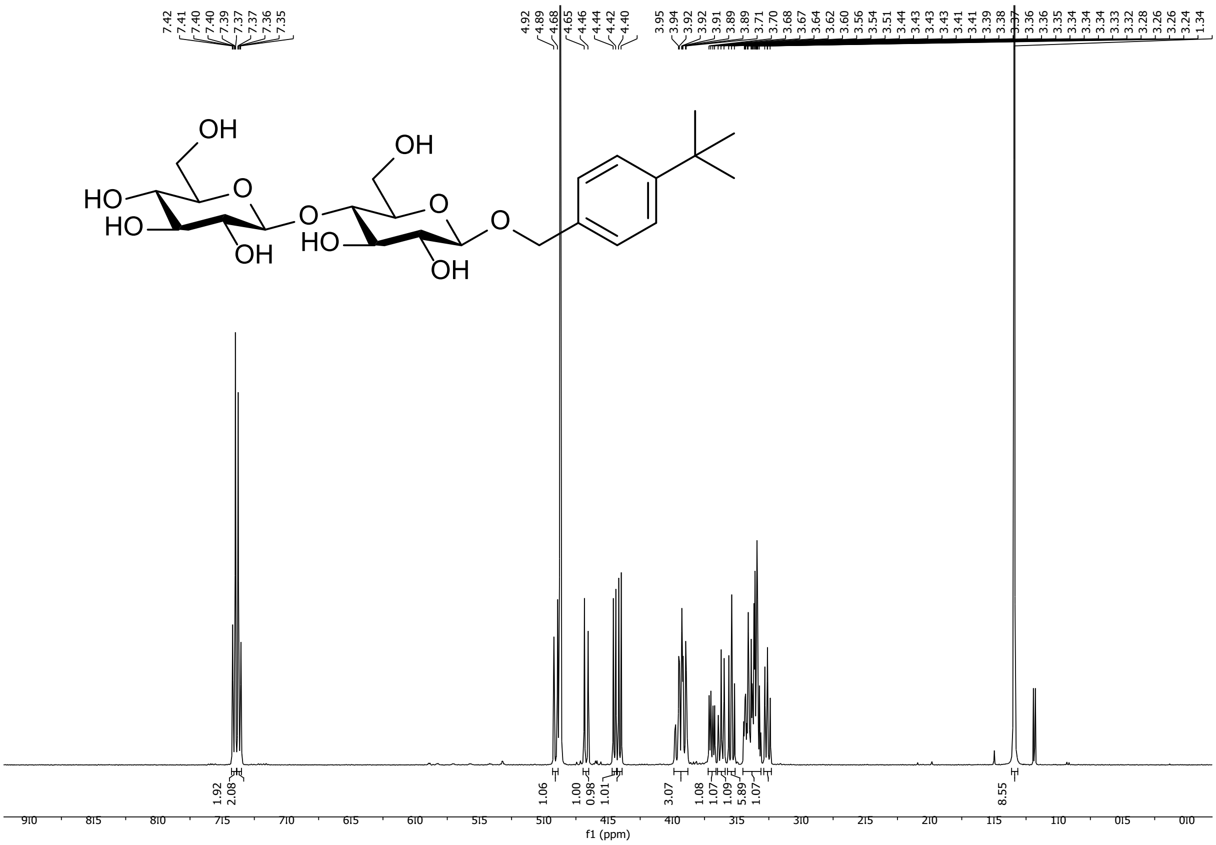

^{13}C NMR, 400 MHz, CD_3OD of compound **S6**

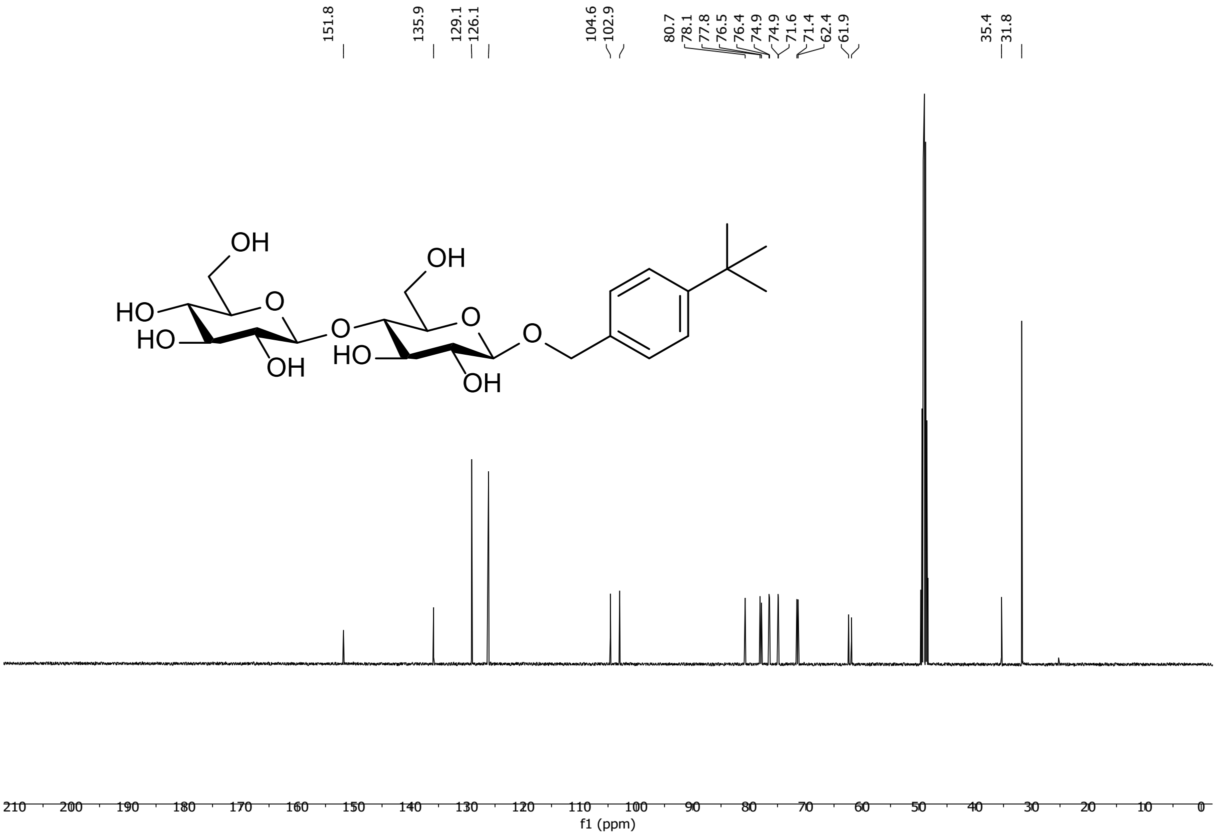

^{1}H-^{1}H COSY of compound **S6**

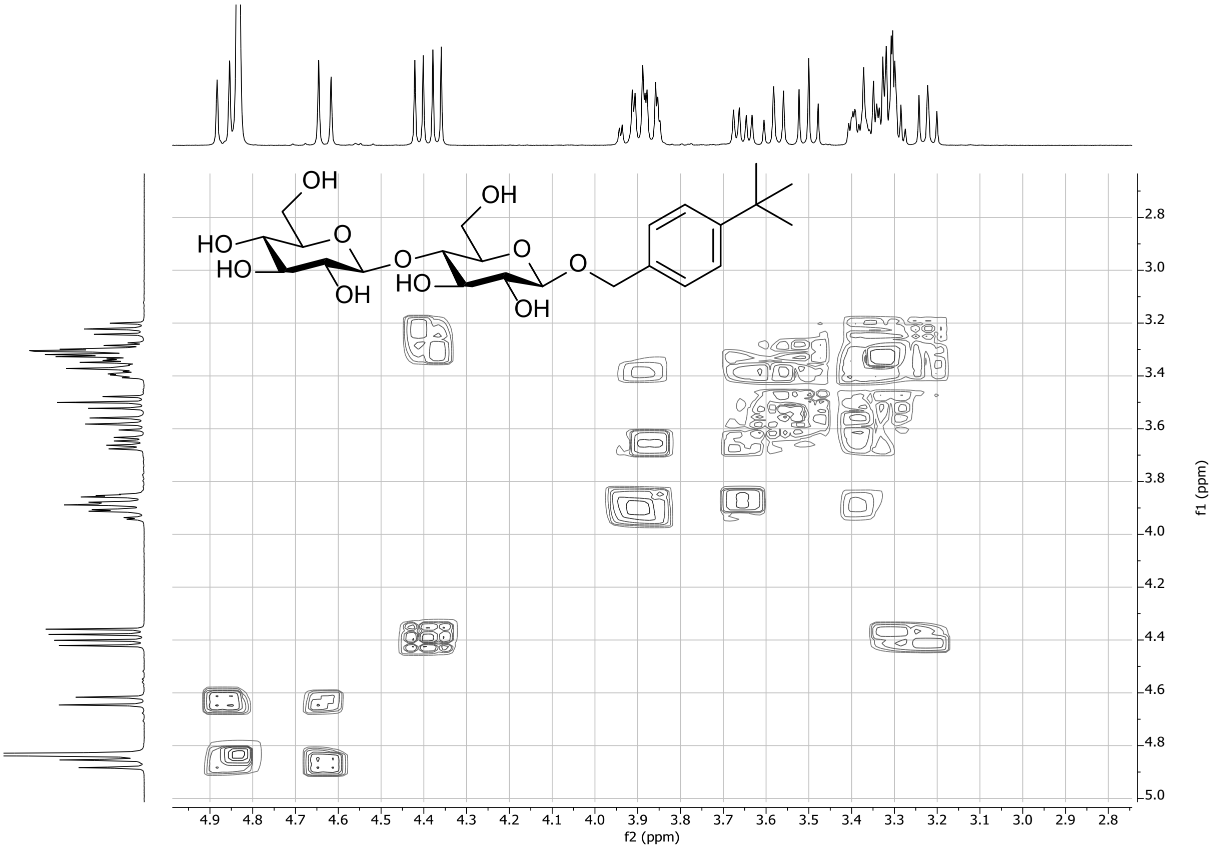

^{1}H-^{13}C HSQC of compound **S6**

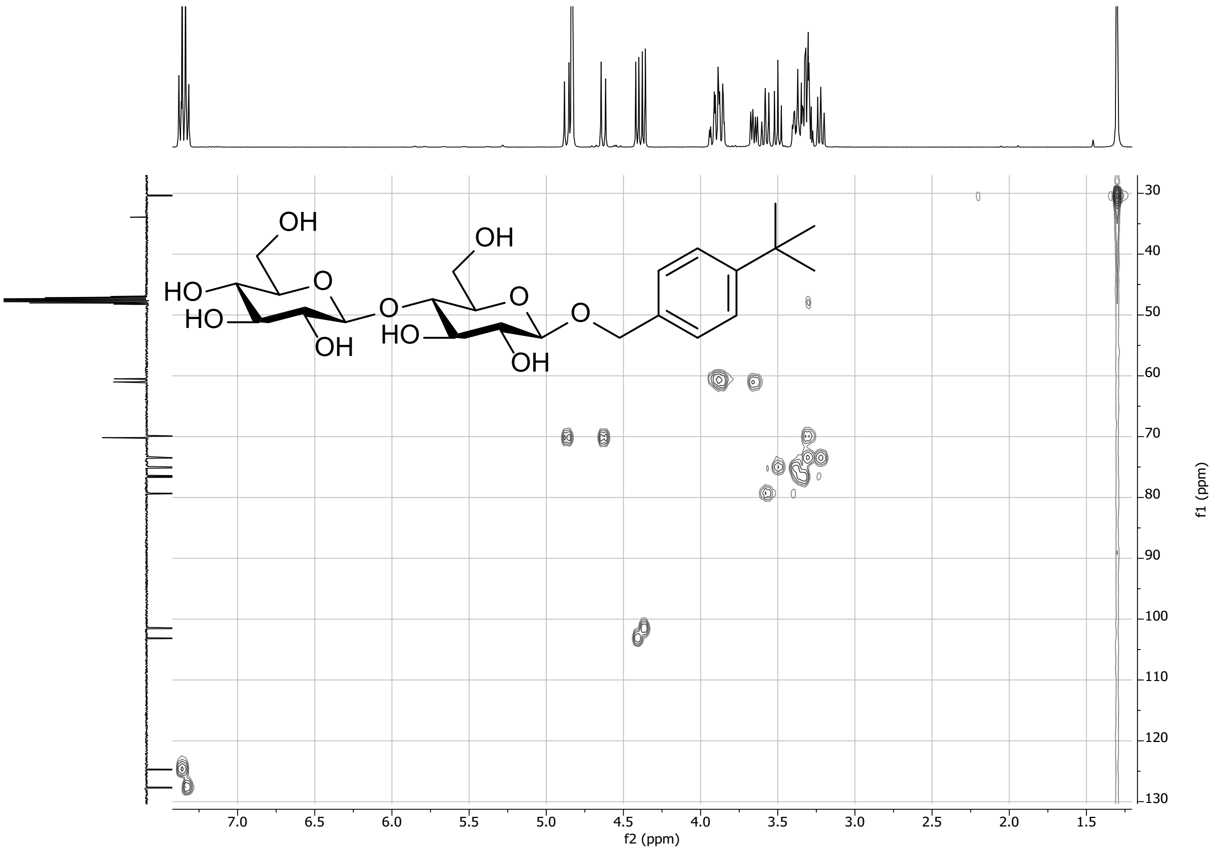

4-tert-butylbenzyl-β-3-ketocellobioside (1e)

^{1}H NMR, 400 MHz, CD_3OD of compound **1e**

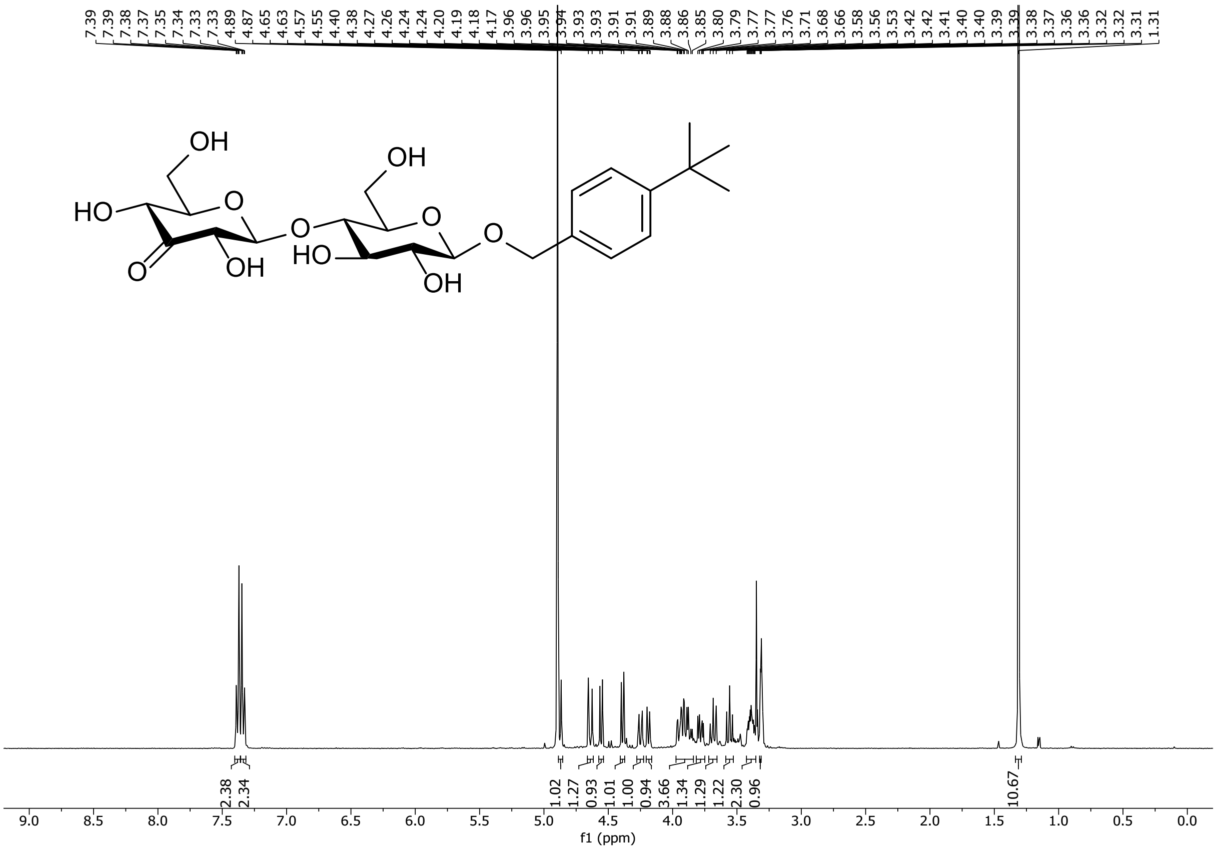

^{13}C NMR, 400 MHz, CD_3OD of compound **1e**

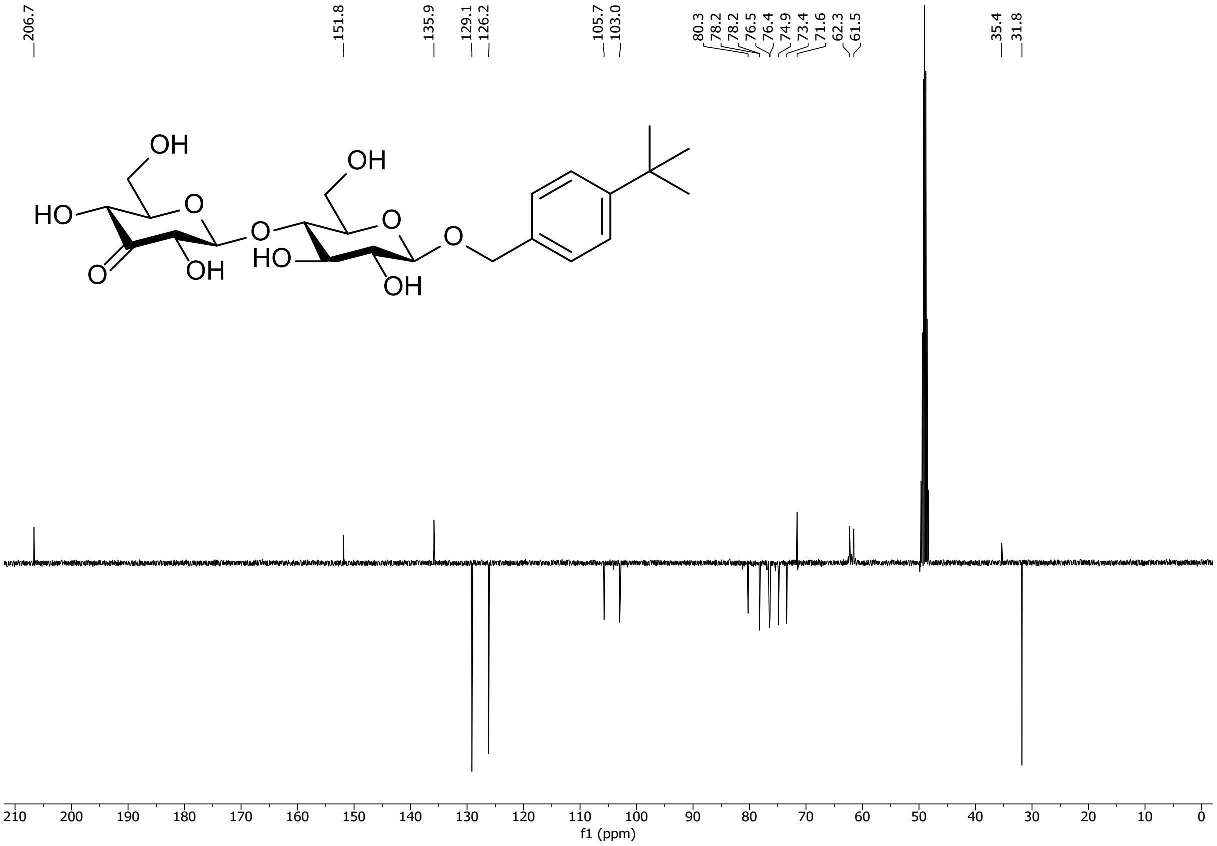

^1H-^1H COSY of compound **1e**

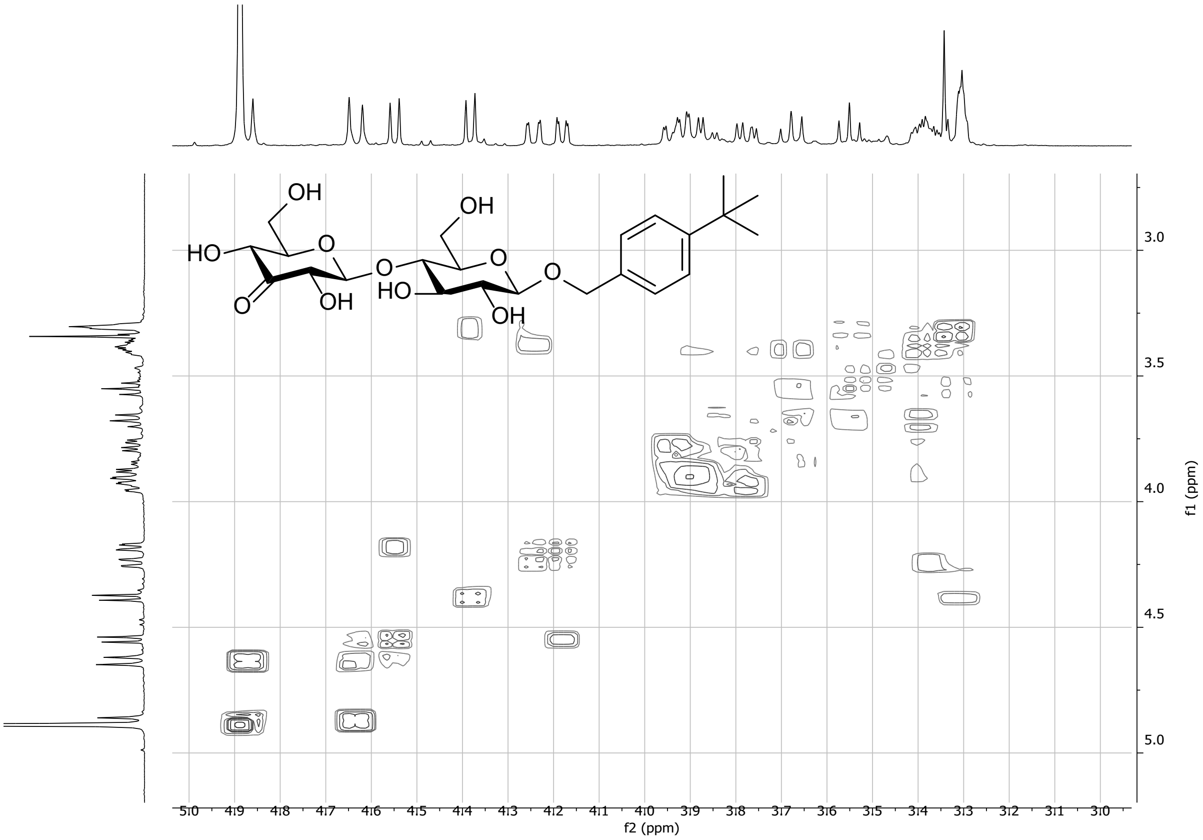

^1H-^{13}C HSQC of compound **1e**

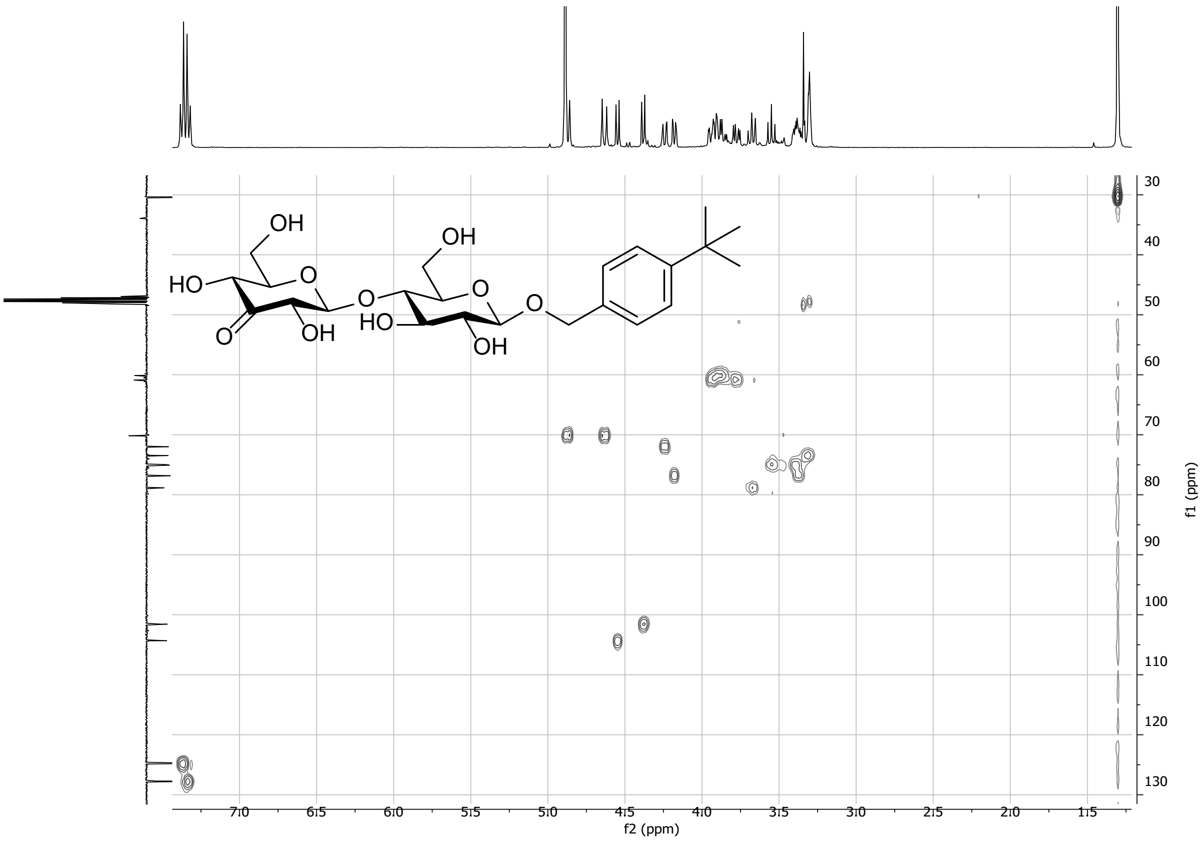

4-tert-butylbenzyl-3-(trityl)hydrazone-β-D-cellobioside (2e)

Mixture of *E* and *Z*: ratio ≈ 1:1.5

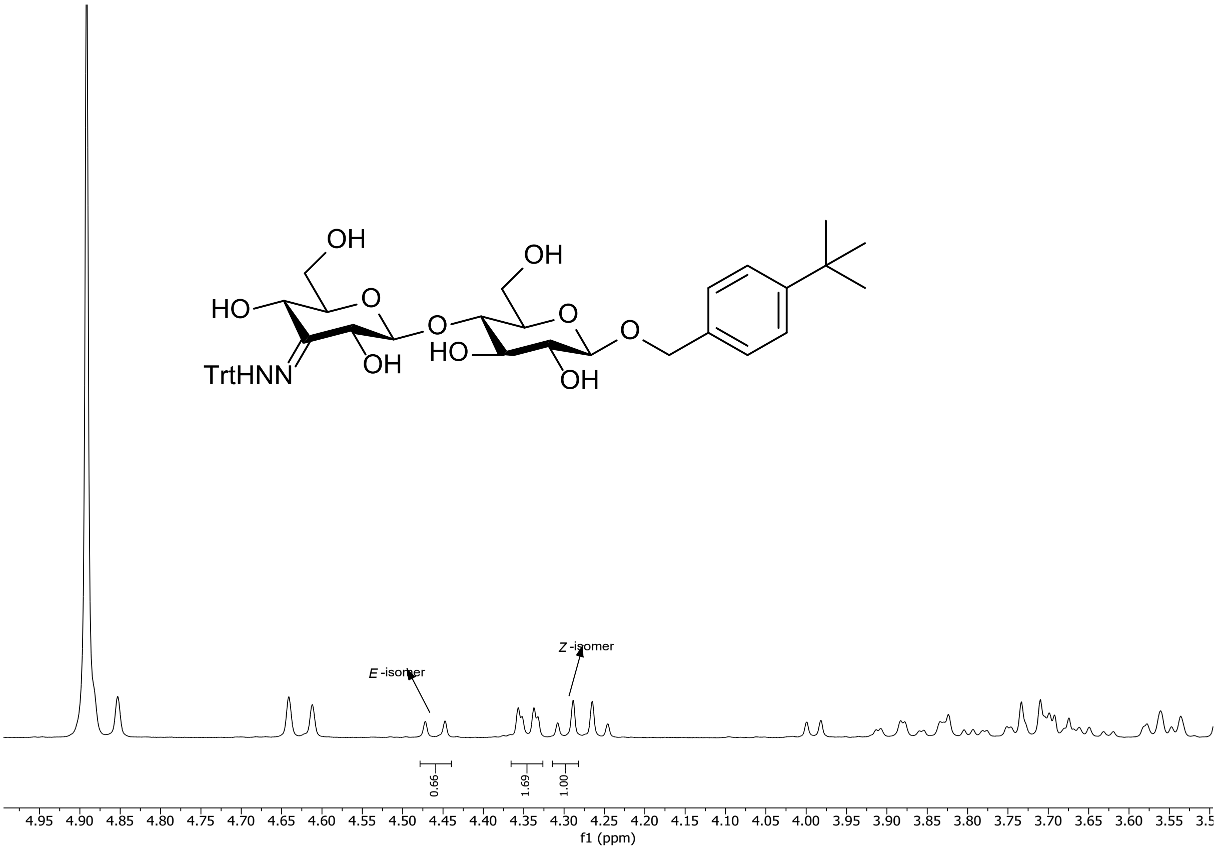

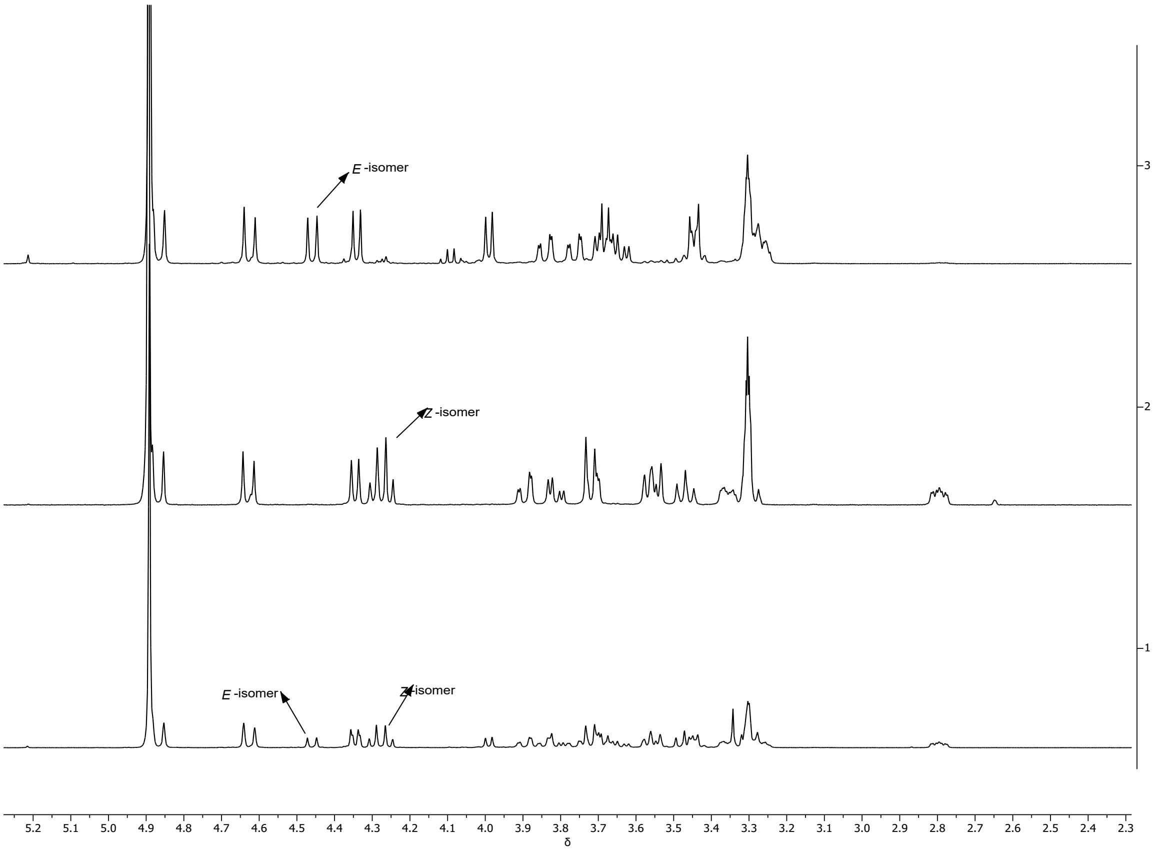

^{1}H NMR, 400 MHz, CD3OD of compound **2e: *Z*-isomer**

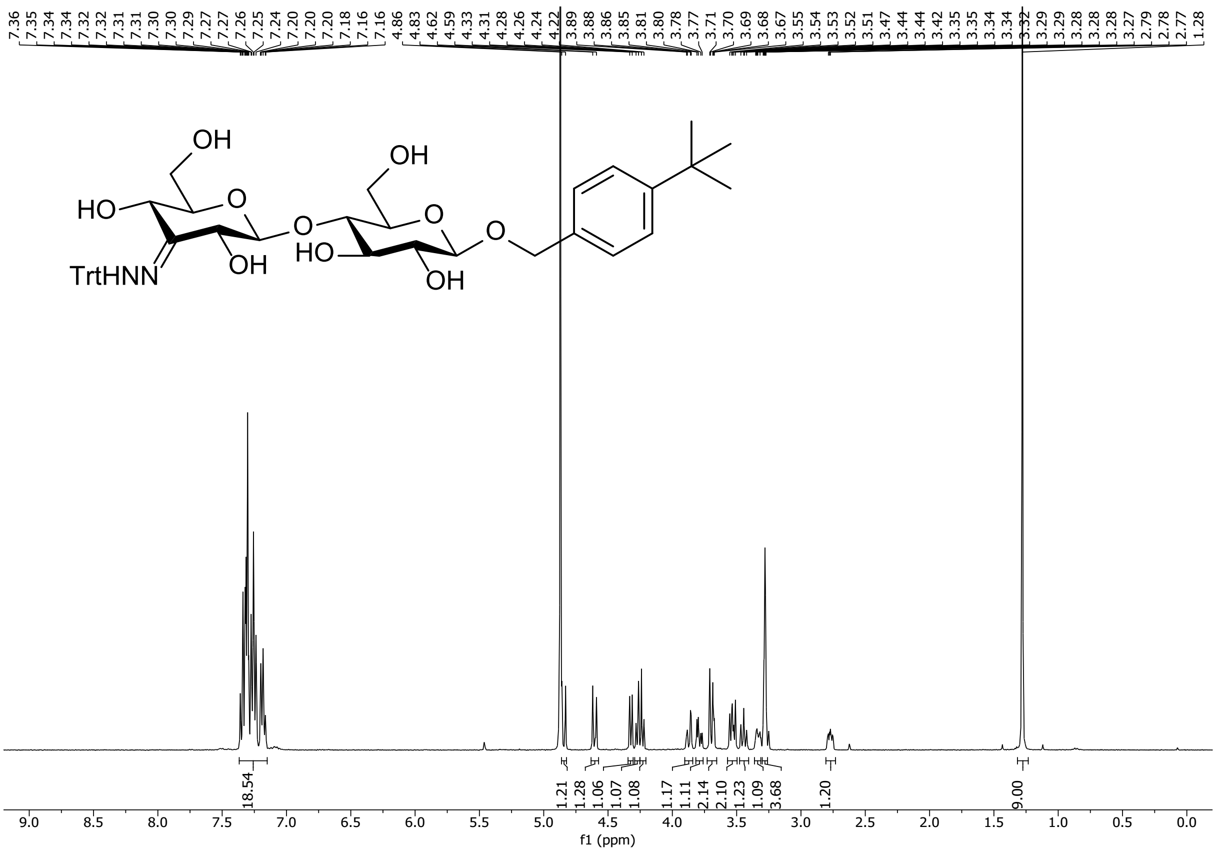

^{13}C NMR, 400 MHz, CD3OD of compound **2e: *Z*-isomer**

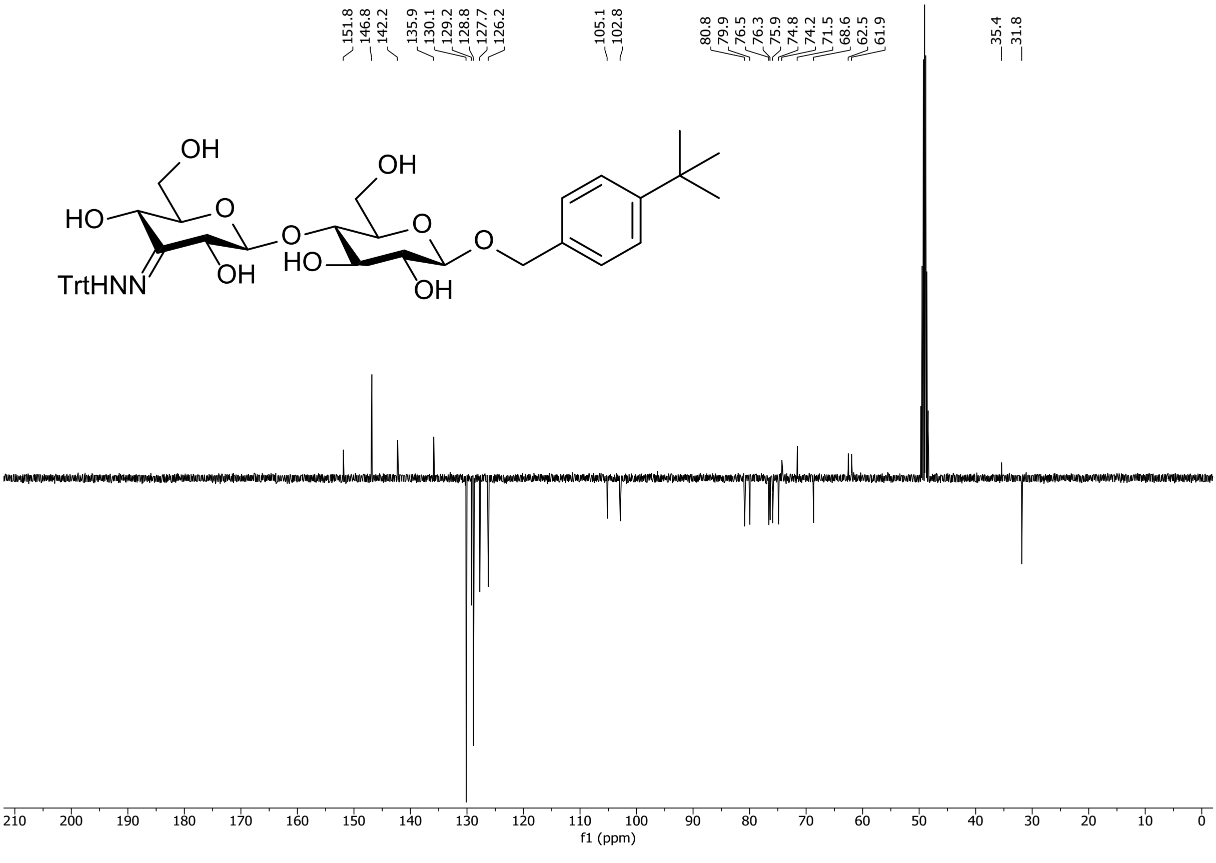

^{1}H-^{1}H COSY of compound **2e: *Z*-isomer**

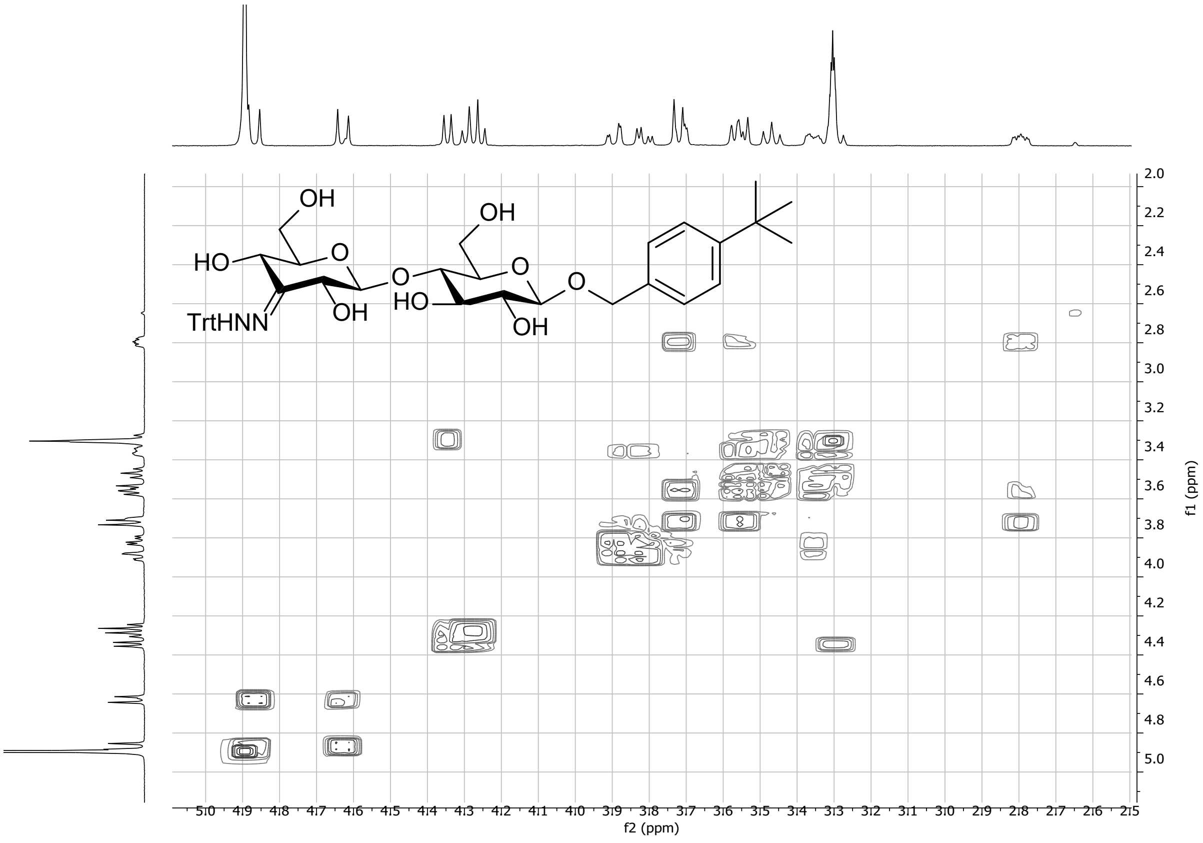

^{1}H-^{13}C HSQC of compound **2e: *Z*-isomer**

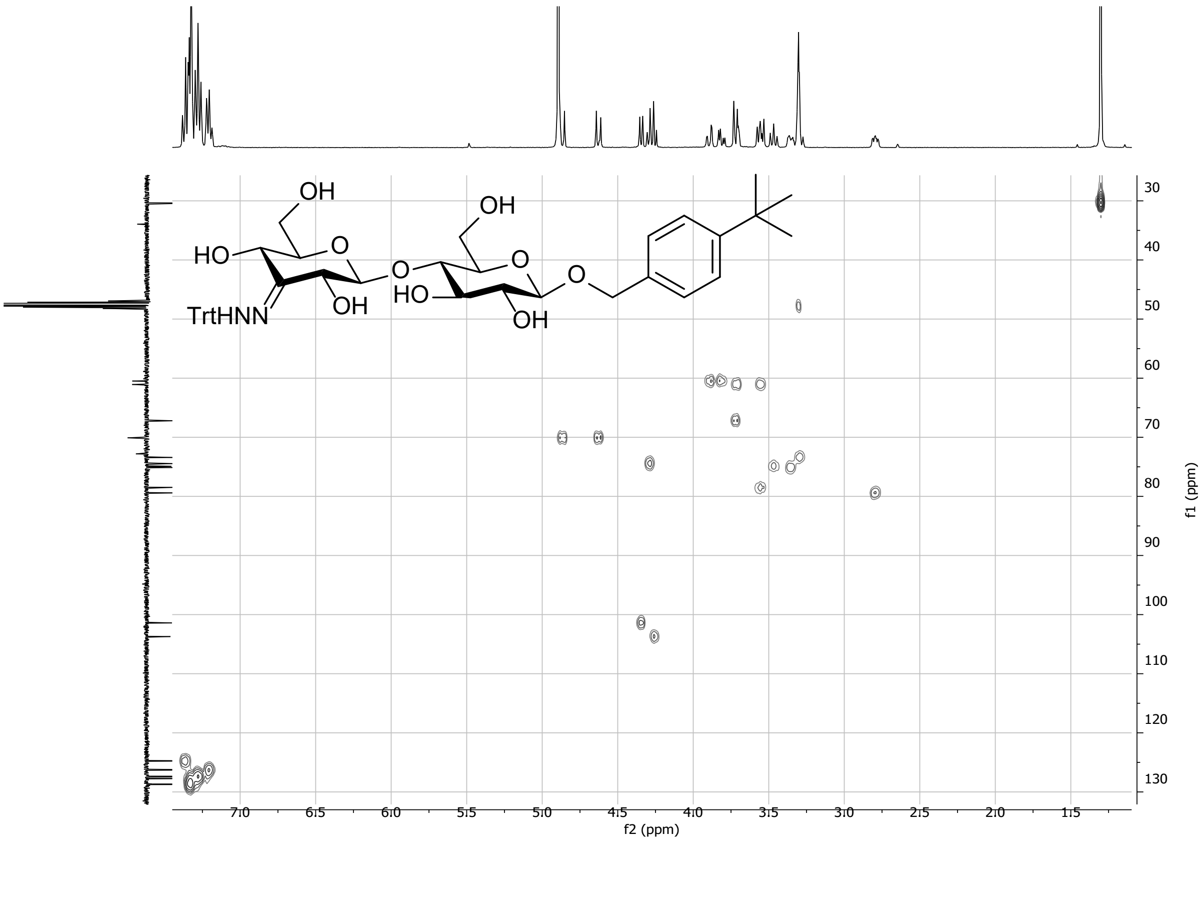

^{1}H NMR, 400 MHz, CD_3OD of compound **2e:** ***E*-isomer**

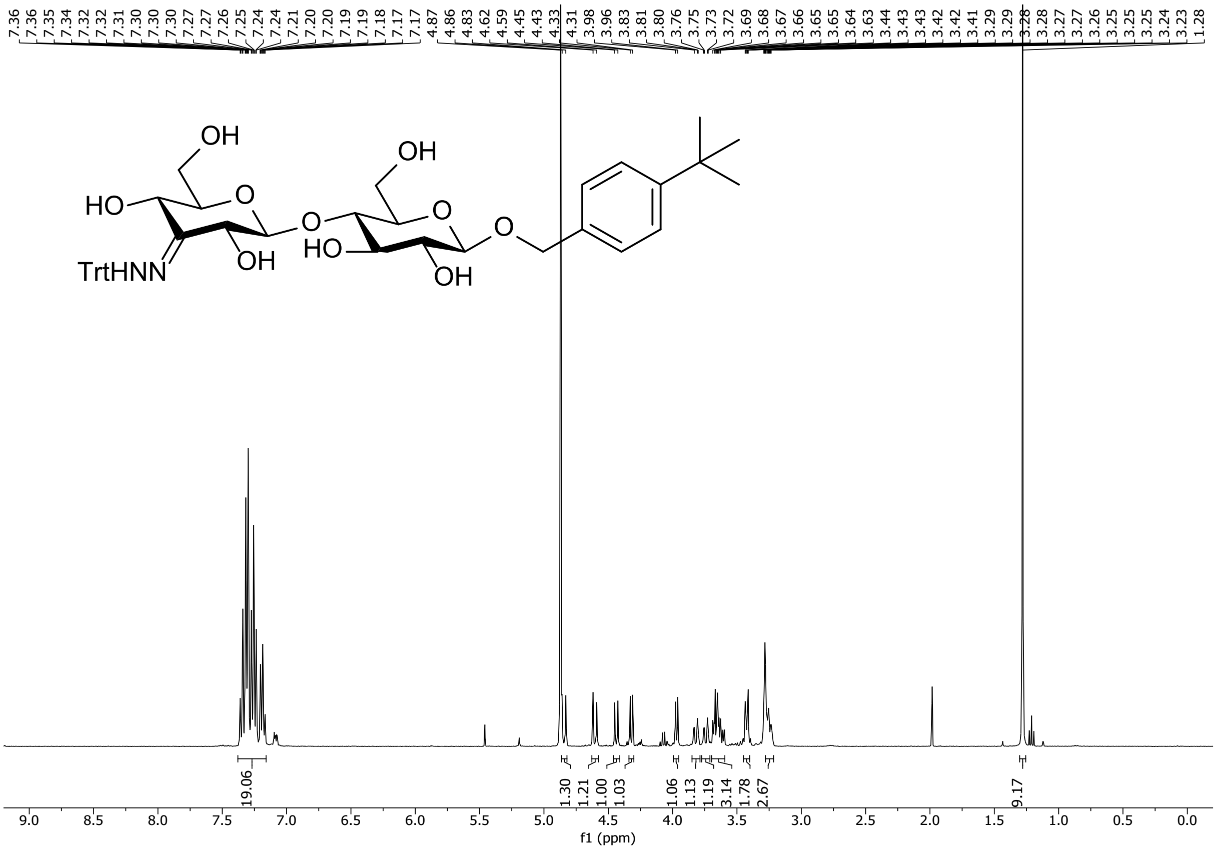

^{13}C NMR, 400 MHz, CD_3OD of compound **2e:** ***E*-isomer**

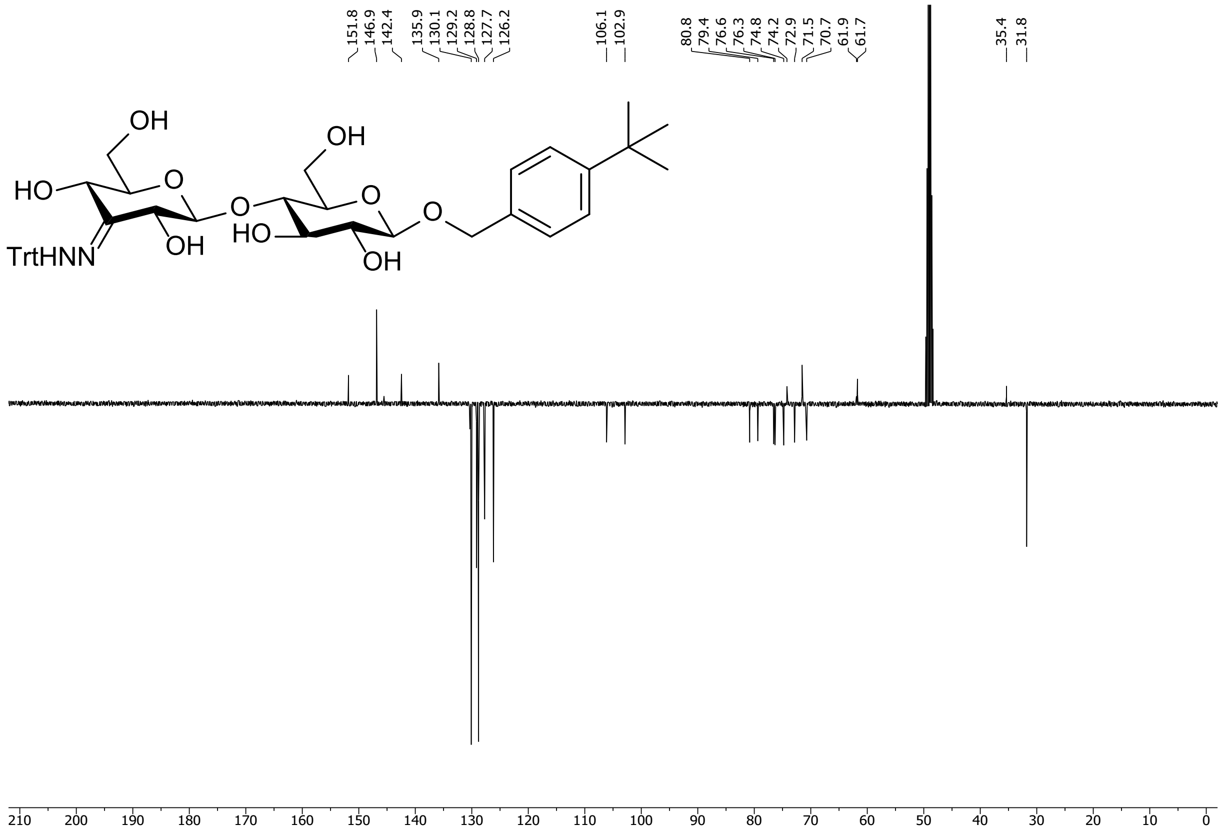

^1H-^1H COSY of compound **2e: *E*-isomer**

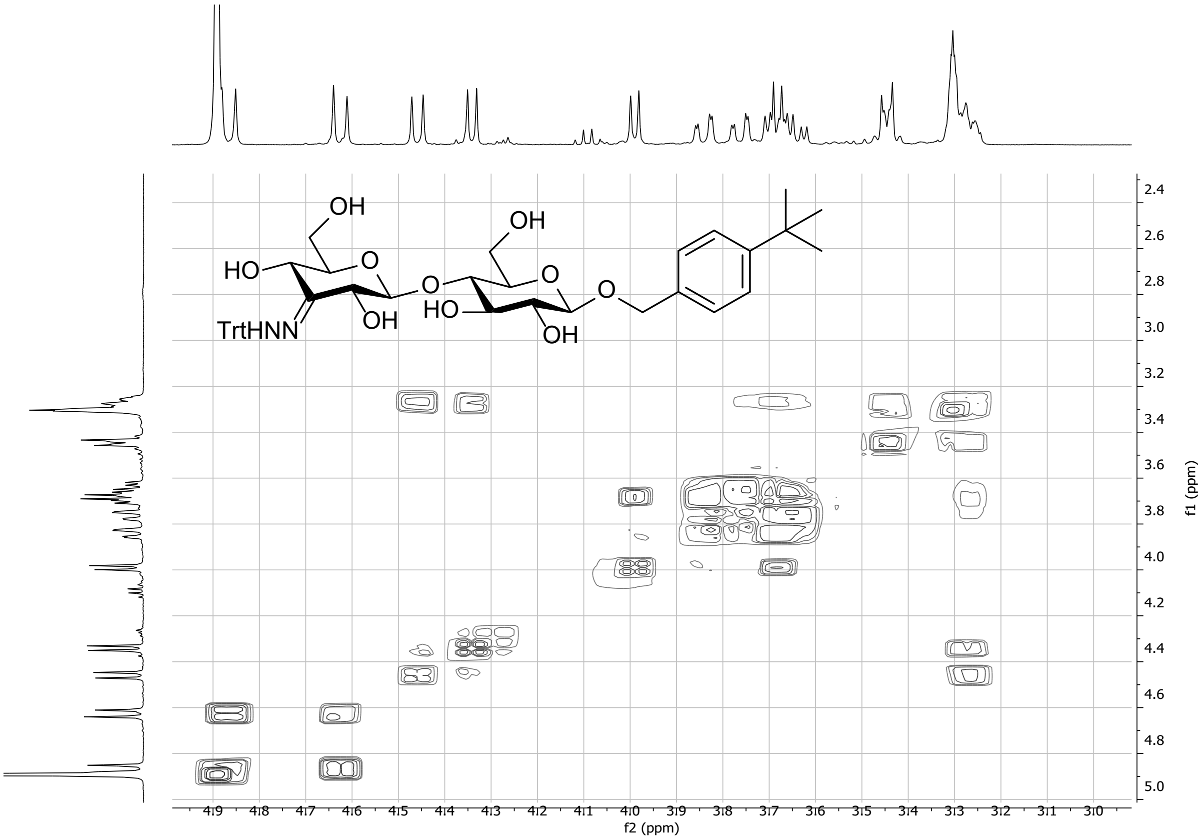

^1H-^{13}C HSQC of compound **2e: *E*-isomer**

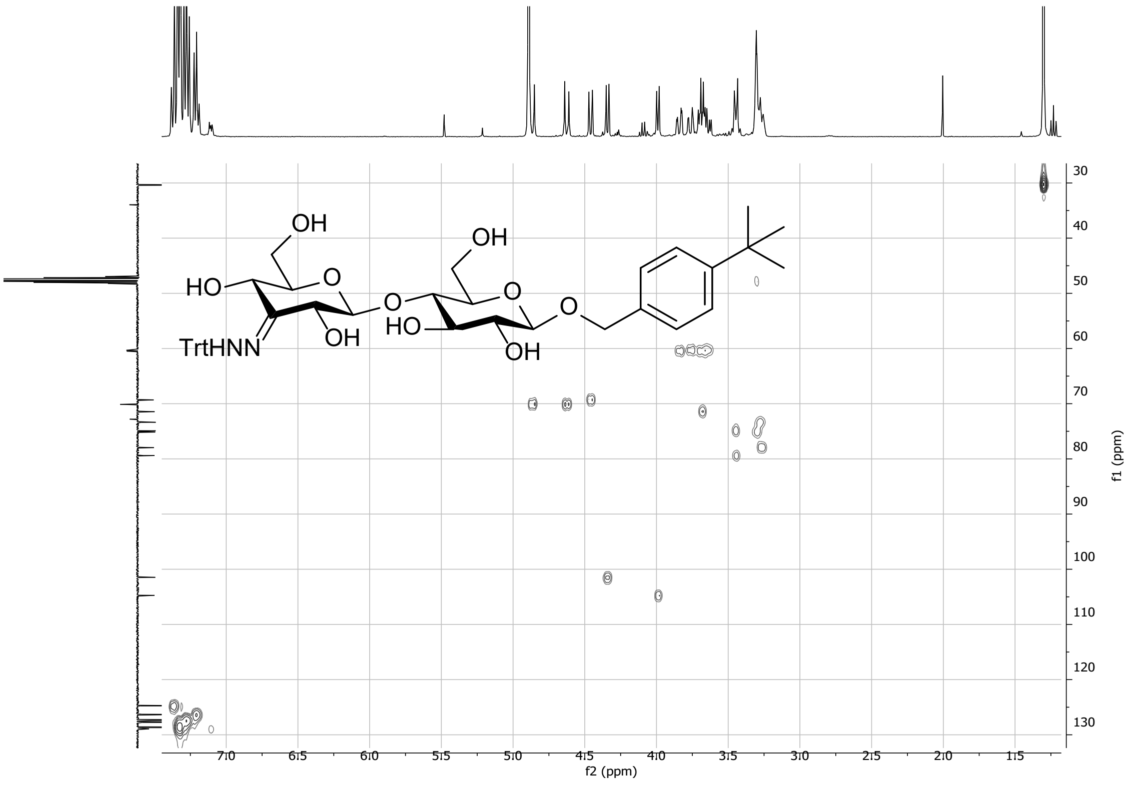

4-tert-butylbenzyl-3-chloro-3-deoxy-β-D-cellobioside (3e)

Mixtue of equatorial and axial, 3-equtorial : 3-axial ≈ 1:2.1

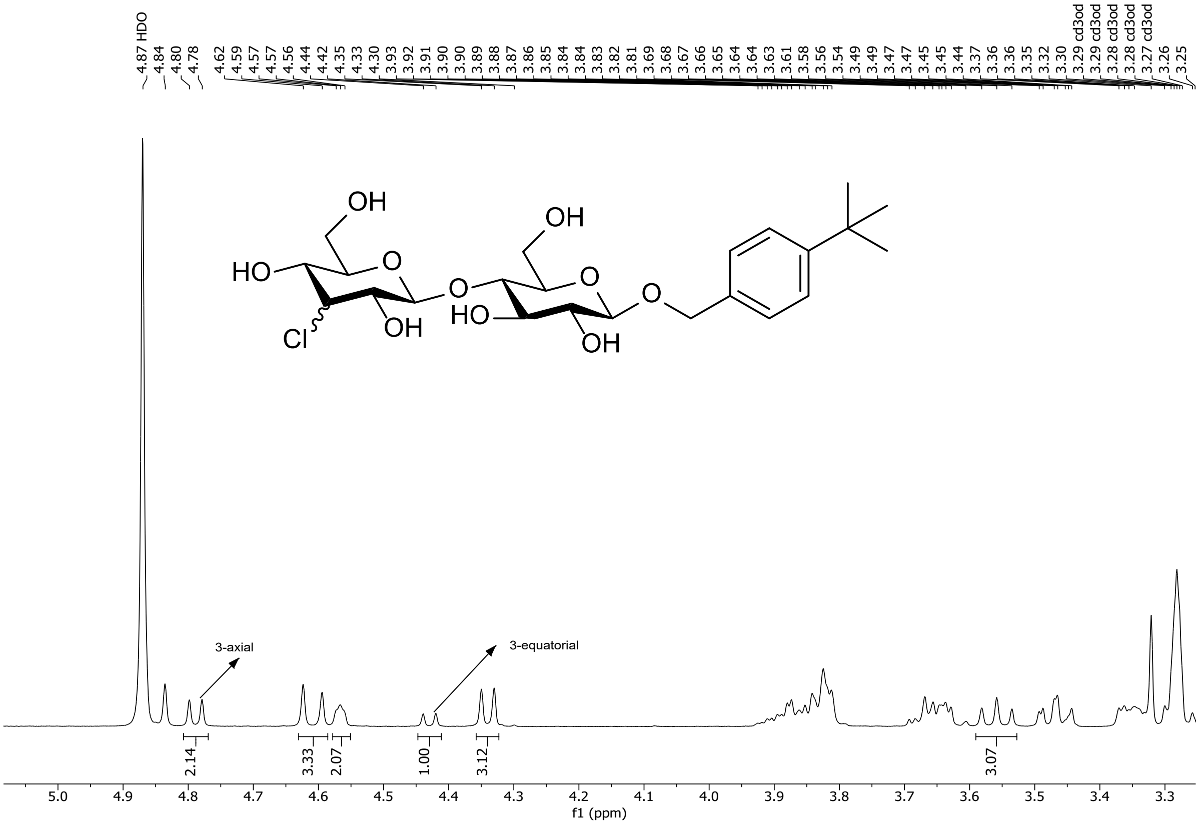

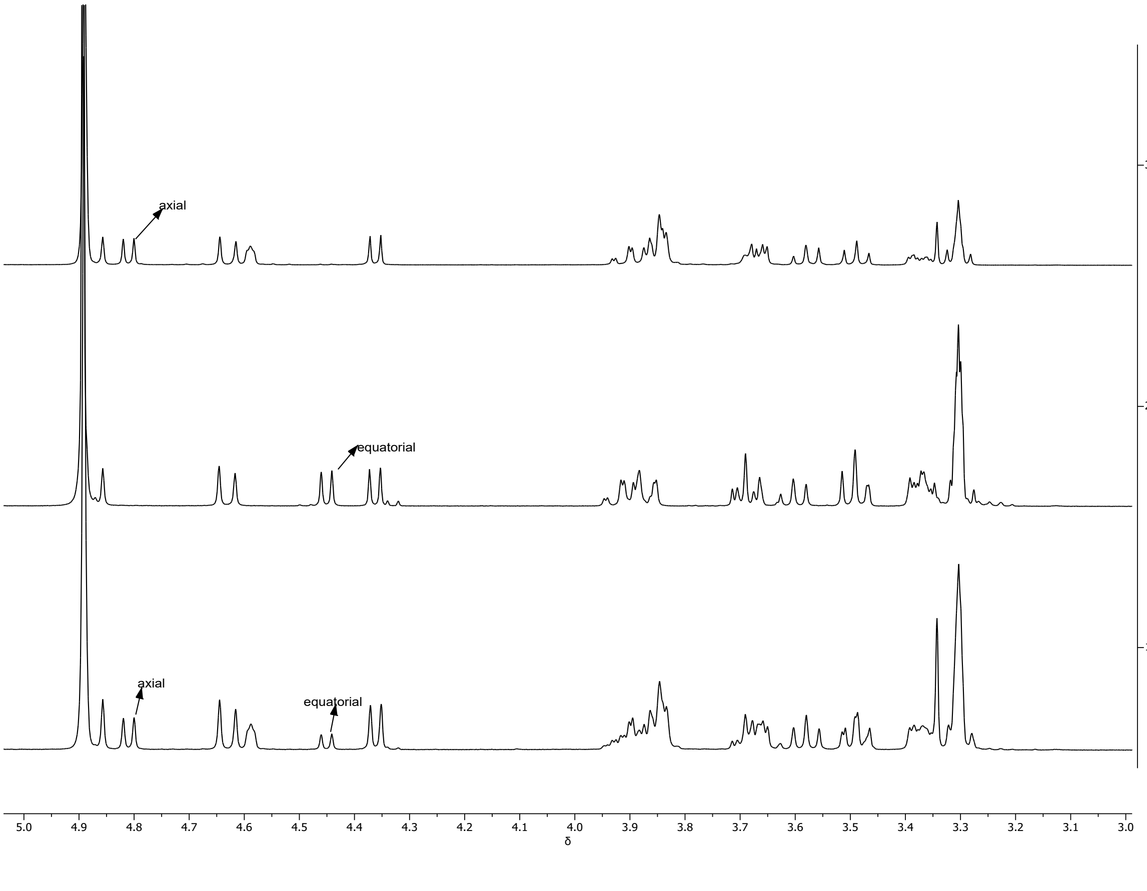

^{1}H NMR, 400 MHz, CD_3OD of compound **3e: equatorial**

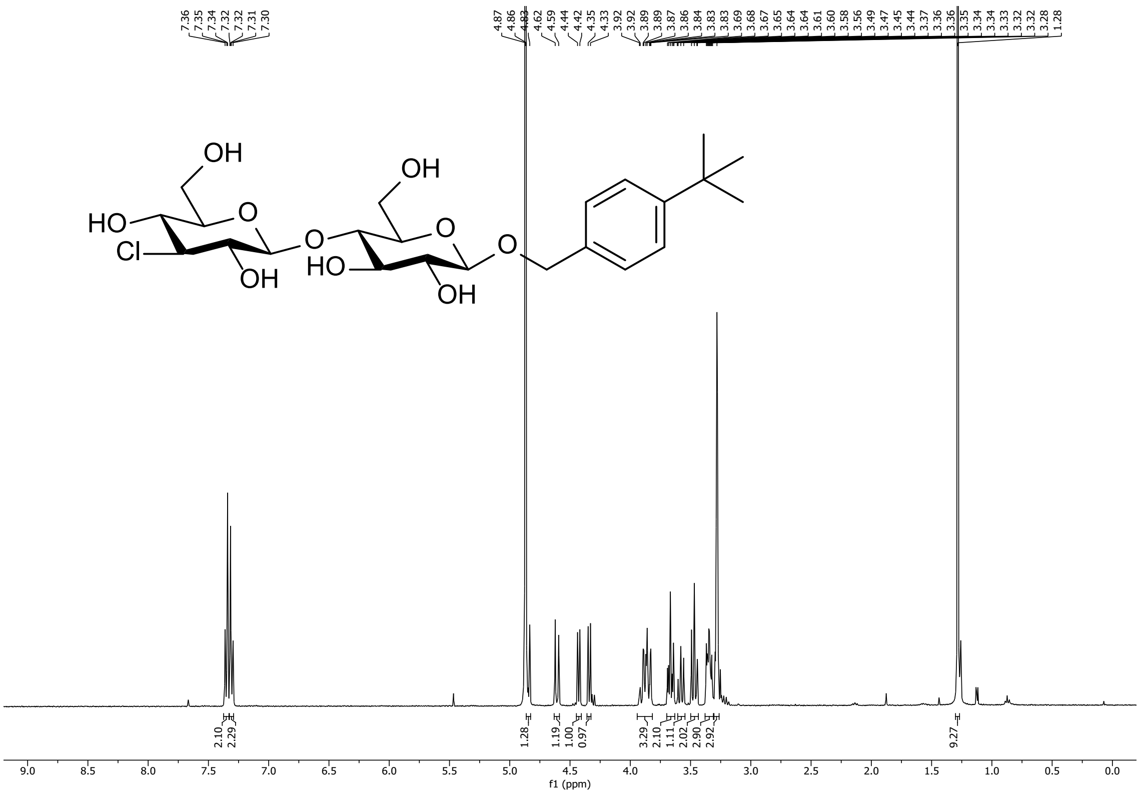

^{13}C NMR, 400 MHz, CD_3OD of compound **3e: equatorial**

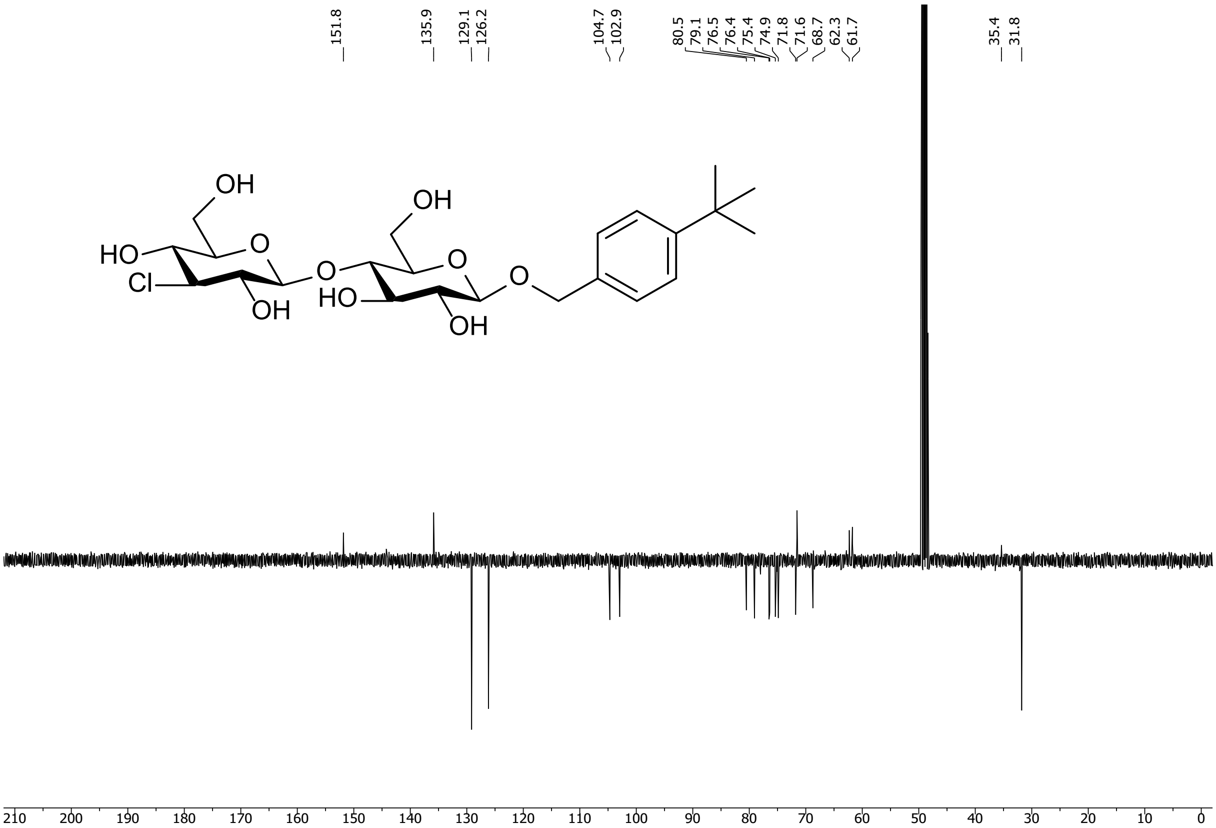

^{1}H-^{1}H COSY of compound **3e: equatorial**

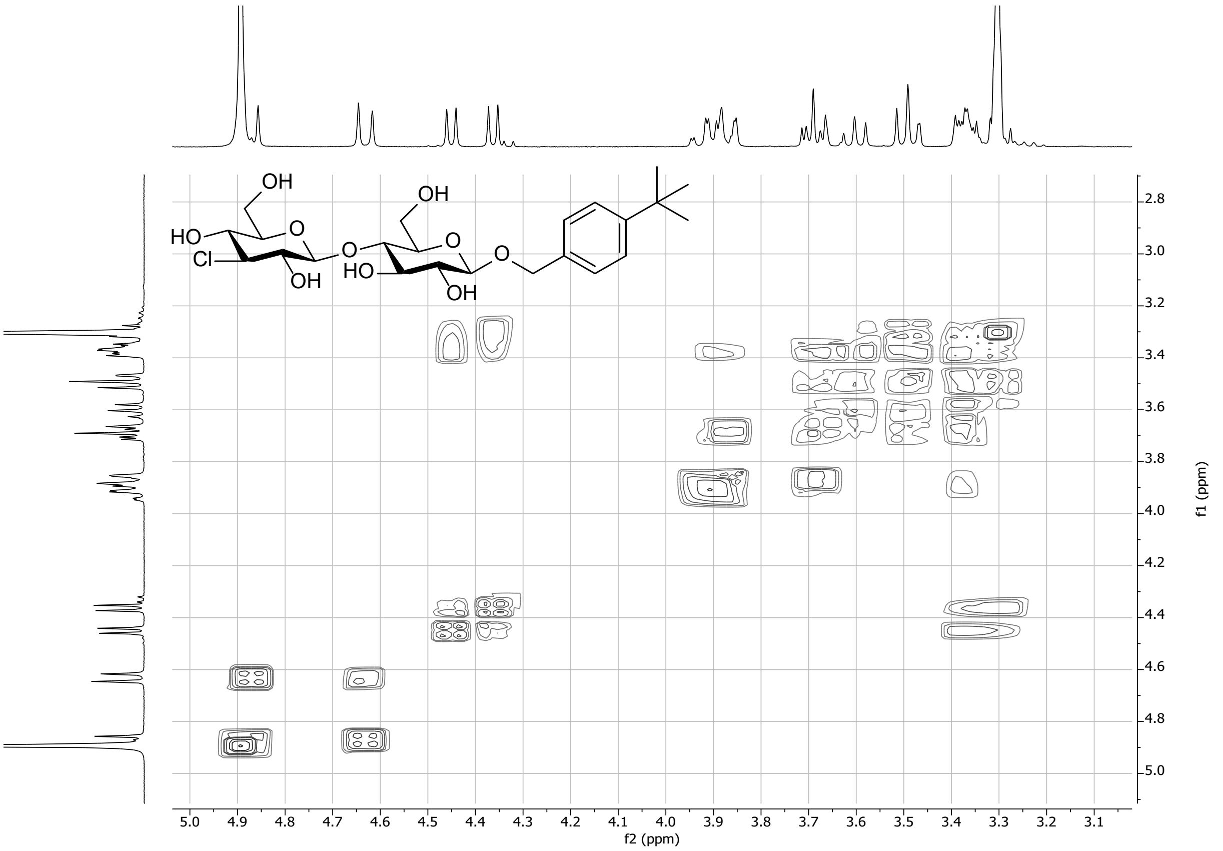

^{1}H-^{13}C HSQC of compound **3e: equatorial**

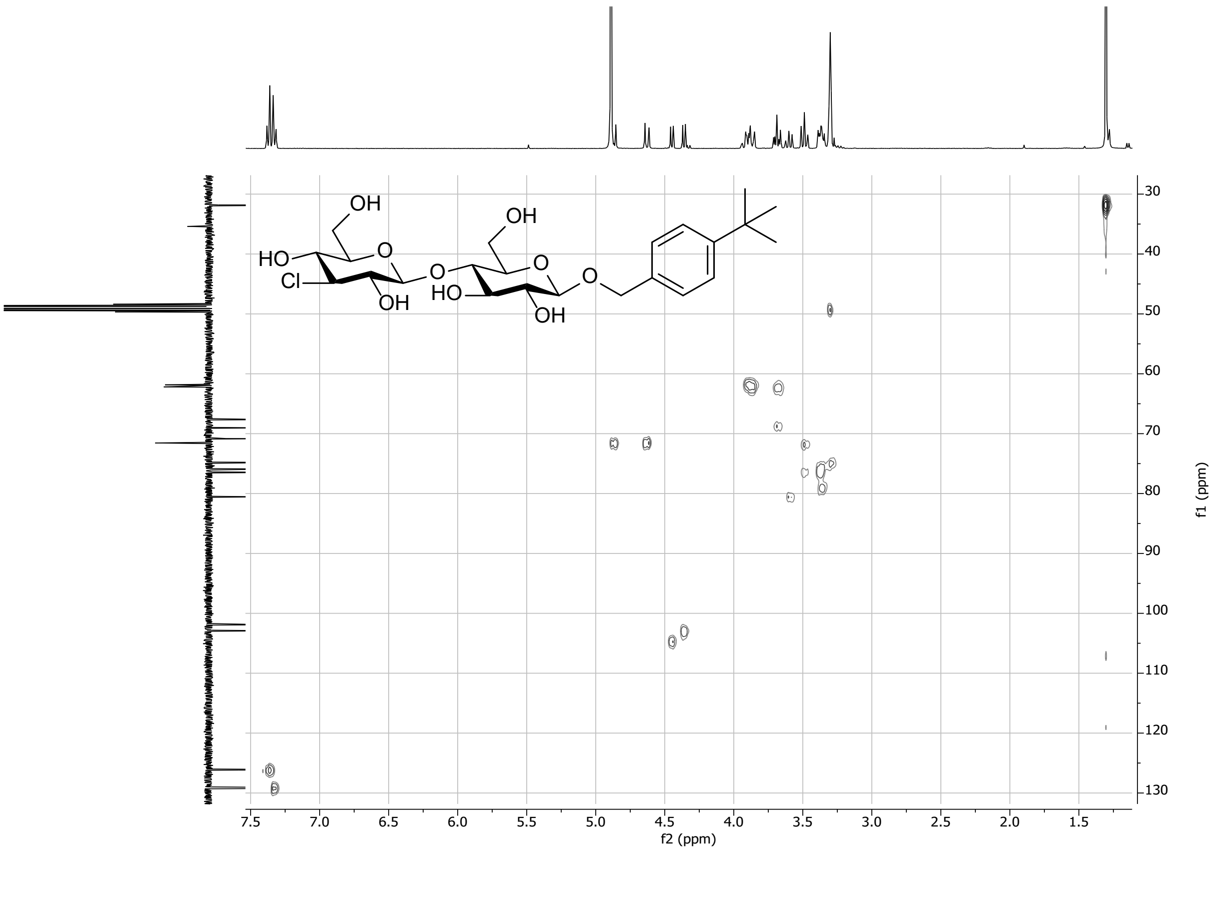

^{1}H NMR, 400 MHz, CD_3OD of compound **3e: axial**

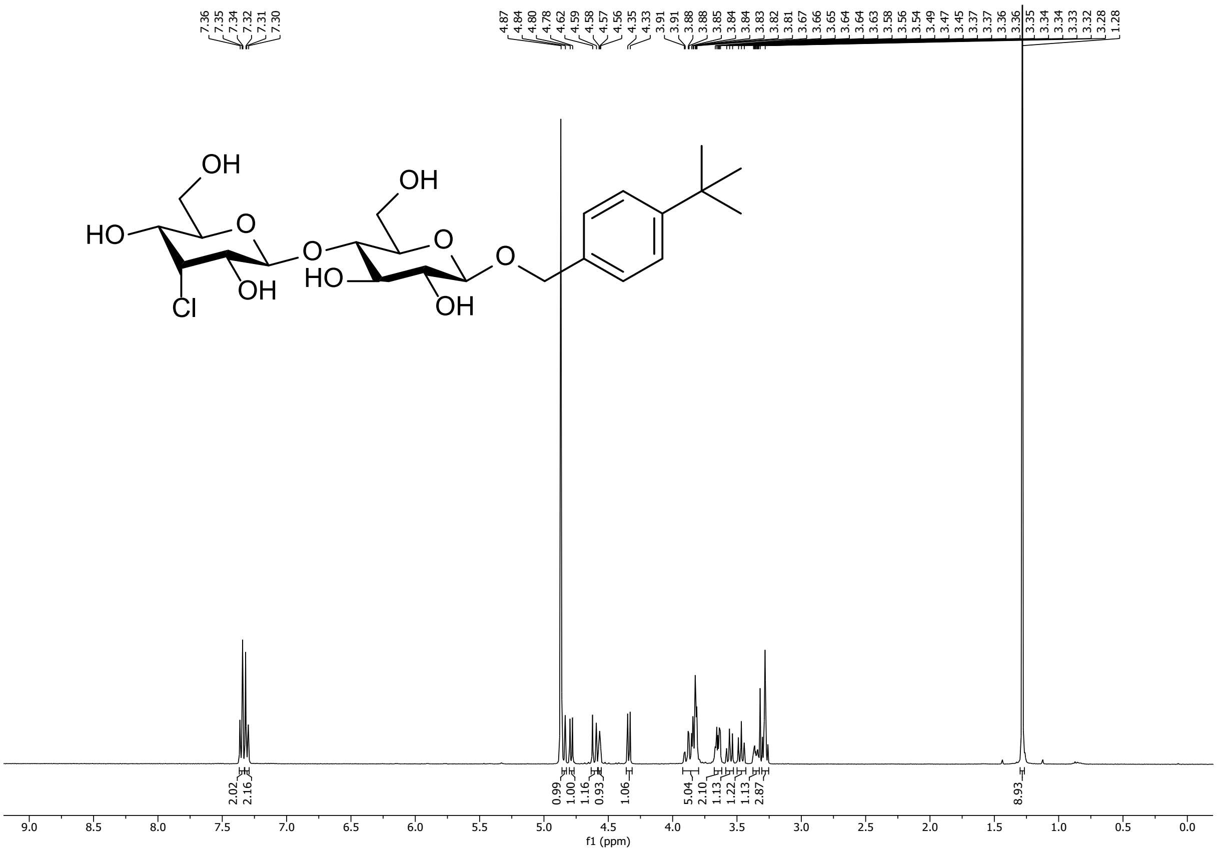

^{13}C NMR, 400 MHz, CD_3OD of compound **3e: axial**

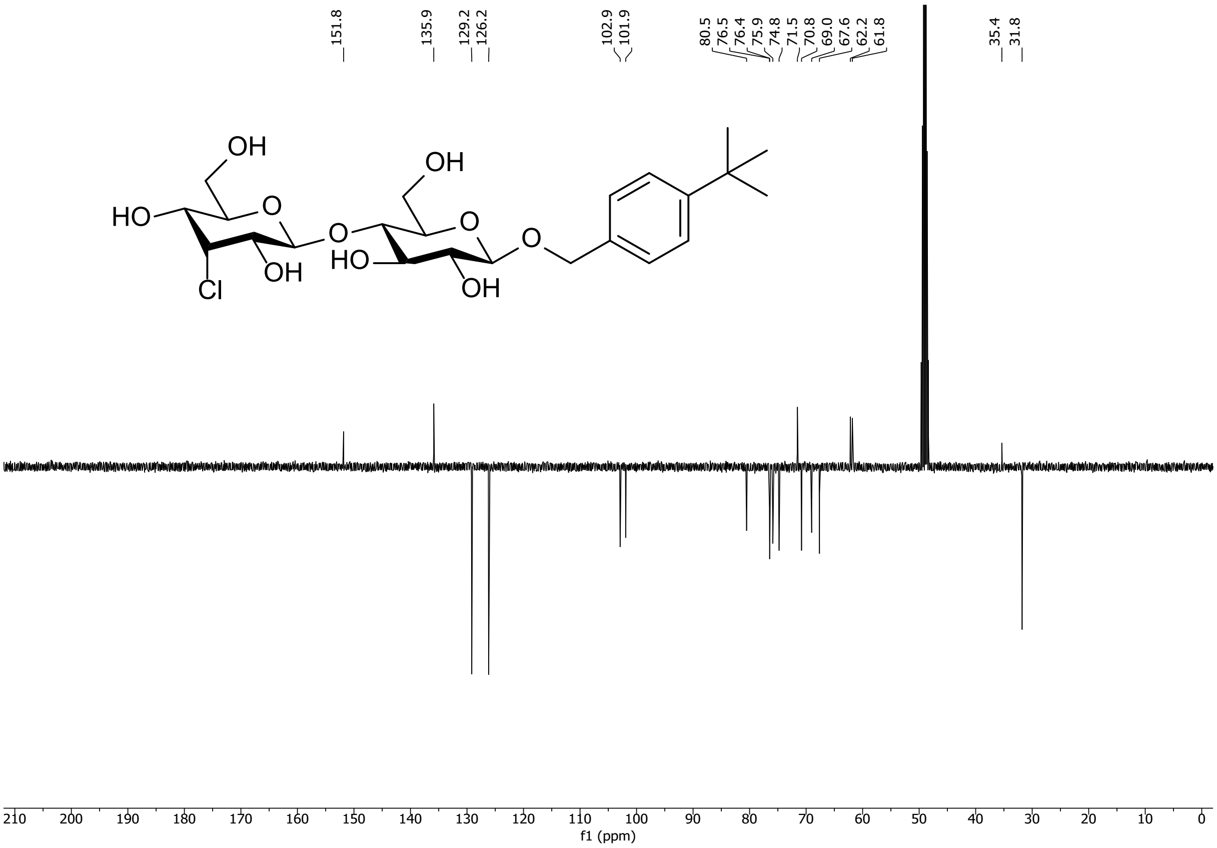

^{1}H-^{1}H COSY of compound **3e: axial**

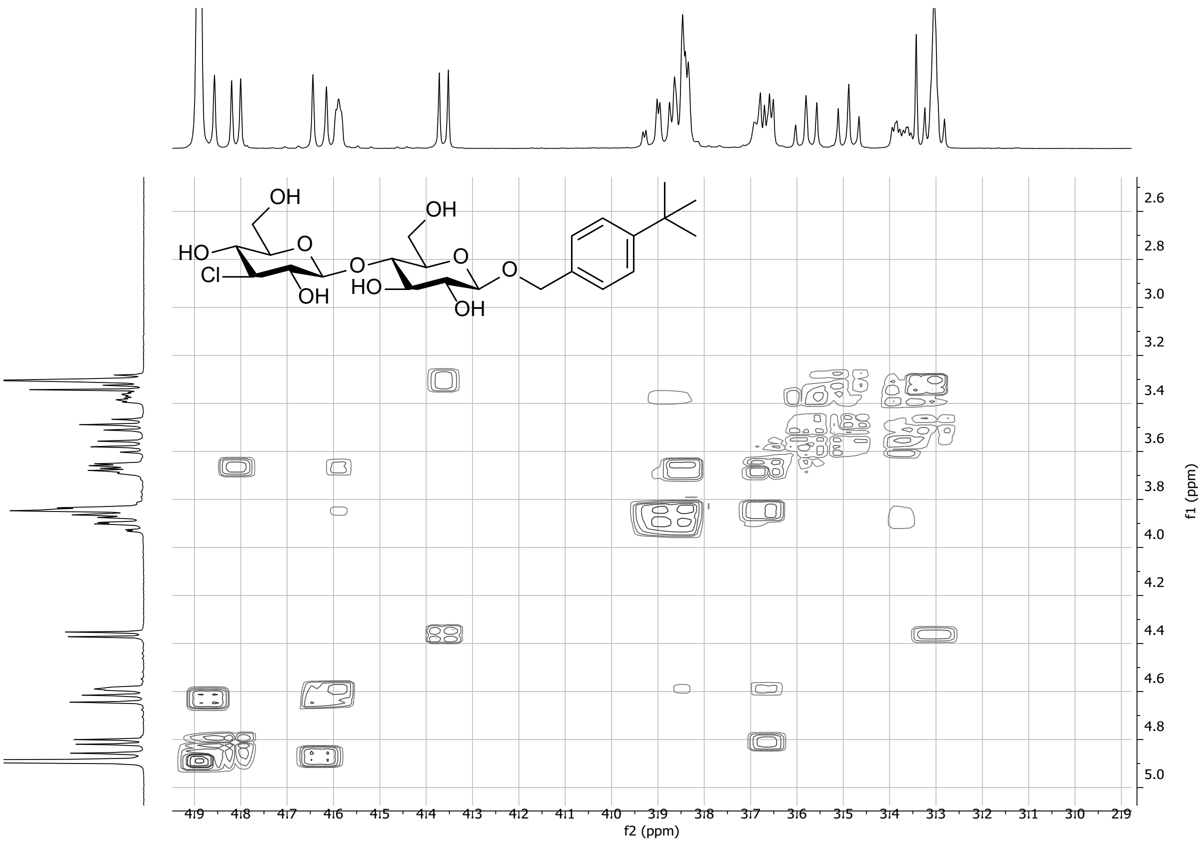

^{1}H-^{13}C HSQC of compound **3e: axial**

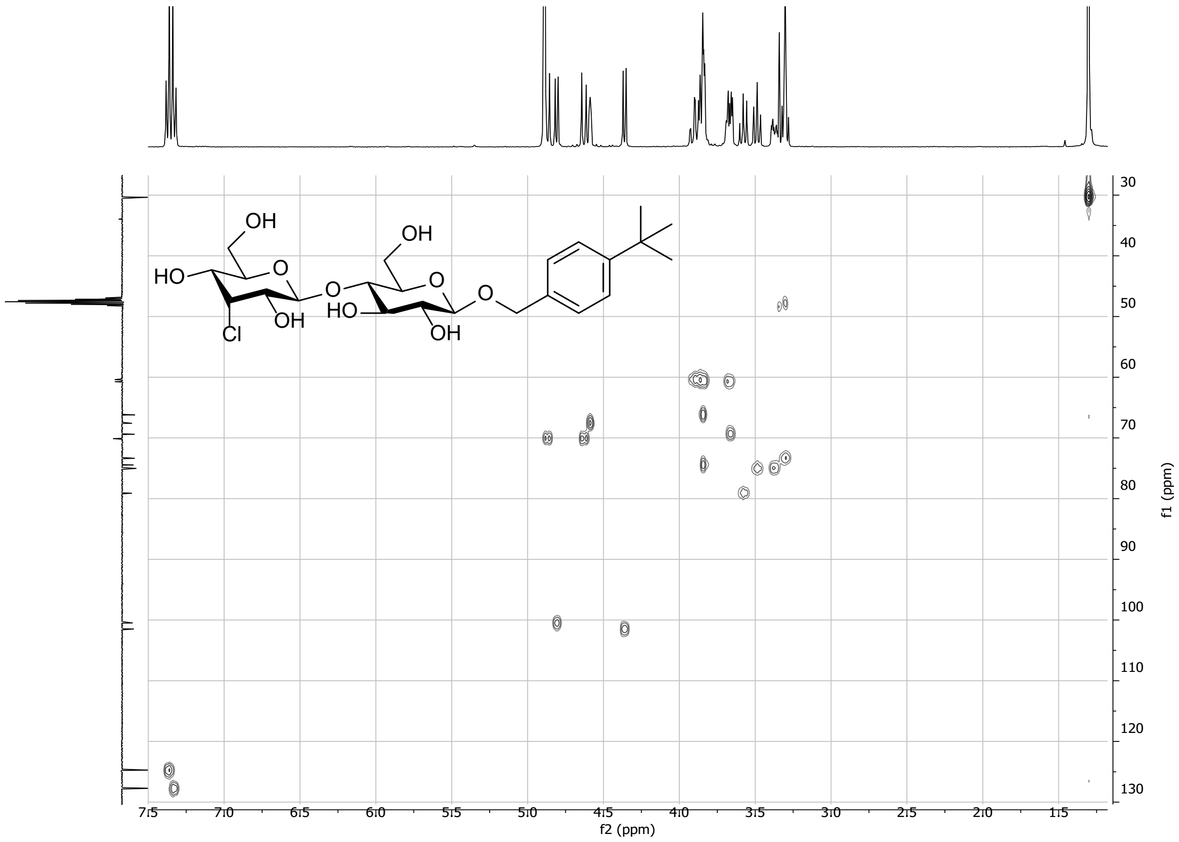

NMR spectra of maltose derivatives**2,3,6,2',3',4',6'-Hepta-*O*-acetyl-maltose (S7)**

^{1}H NMR, 400 MHz, $CDCl_3$ of compound **S7**

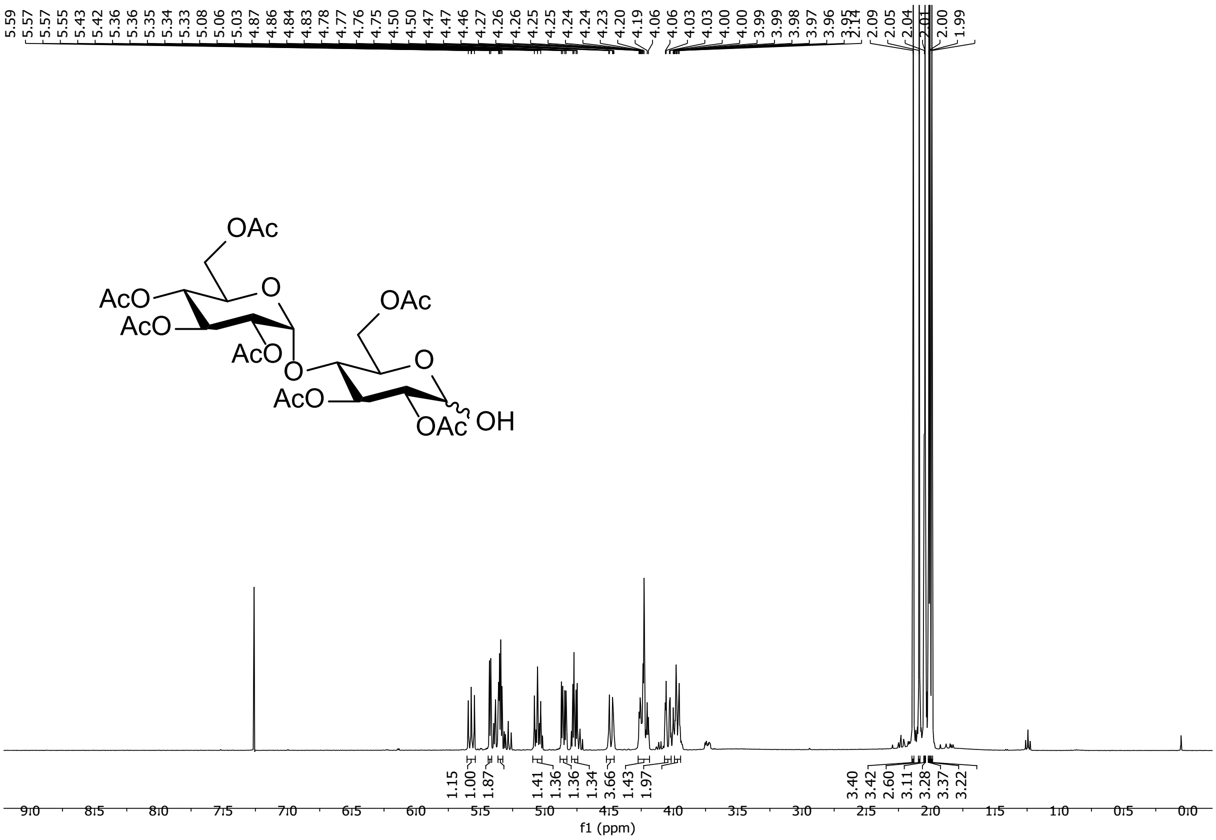

^{13}C NMR, 400 MHz, $CDCl_3$ of compound **S7**

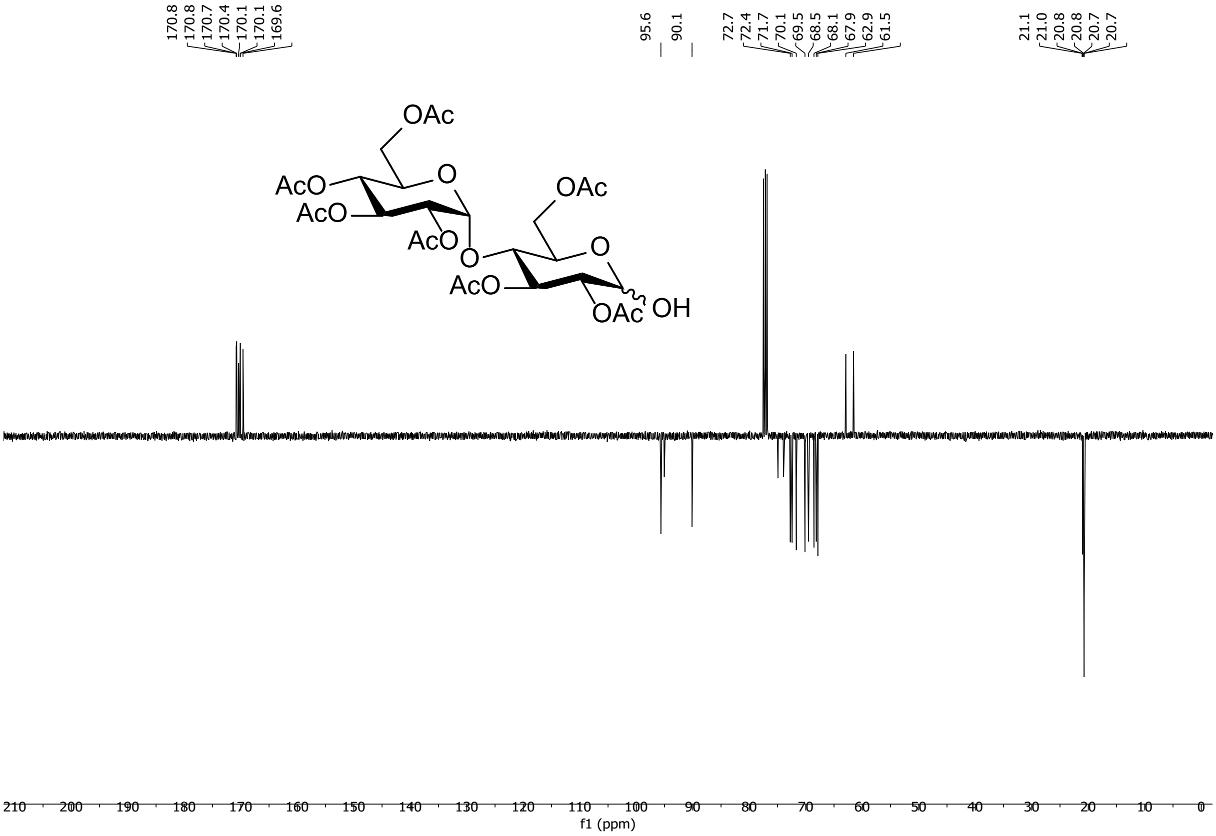

^{1}H-^{1}H COSY of compound **S7**

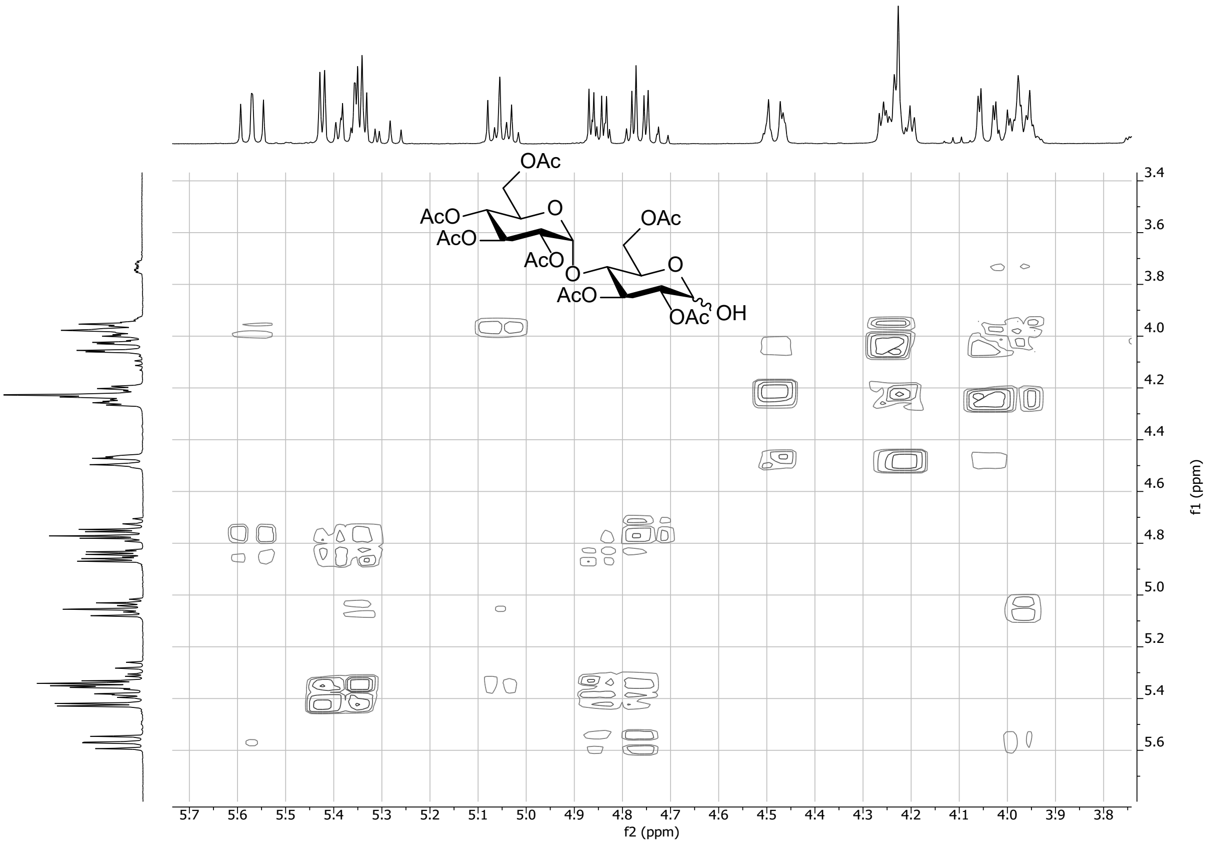

^{1}H-^{13}C HSQC of compound **S7**

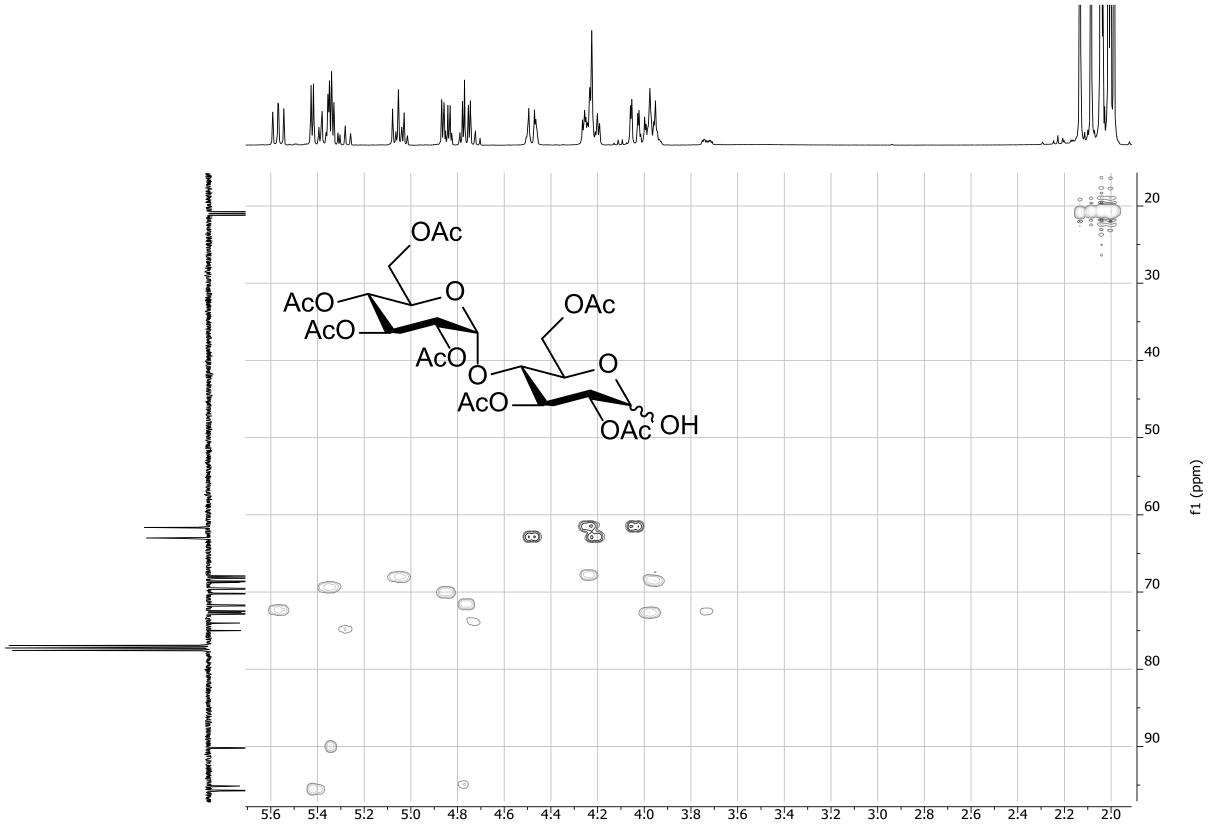

4-tert-butylbenzyl-β-D-heptaacetyl-maltoside (S8)

^{1}H NMR, 400 MHz, $CDCl_3$ of compound **S8**

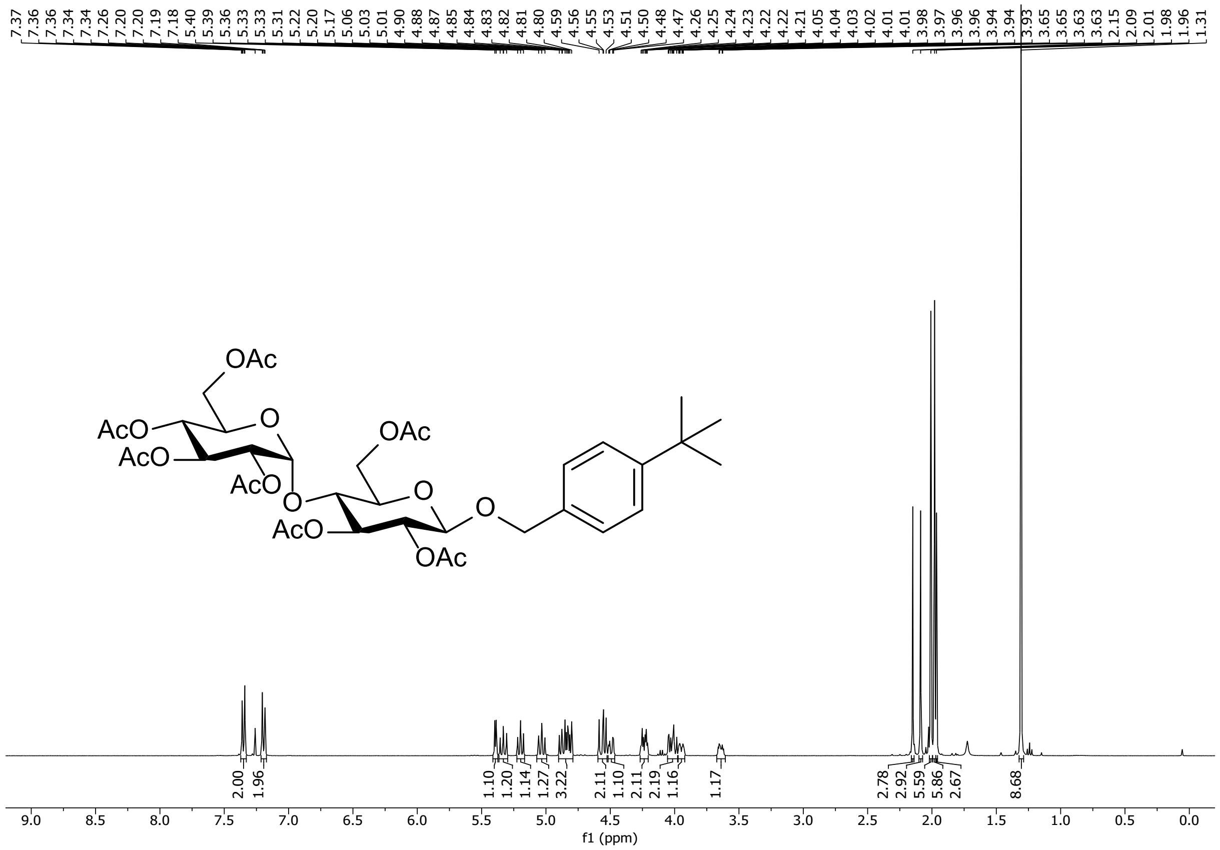

^{13}C NMR, 400 MHz, $CDCl_3$ of compound **S8**

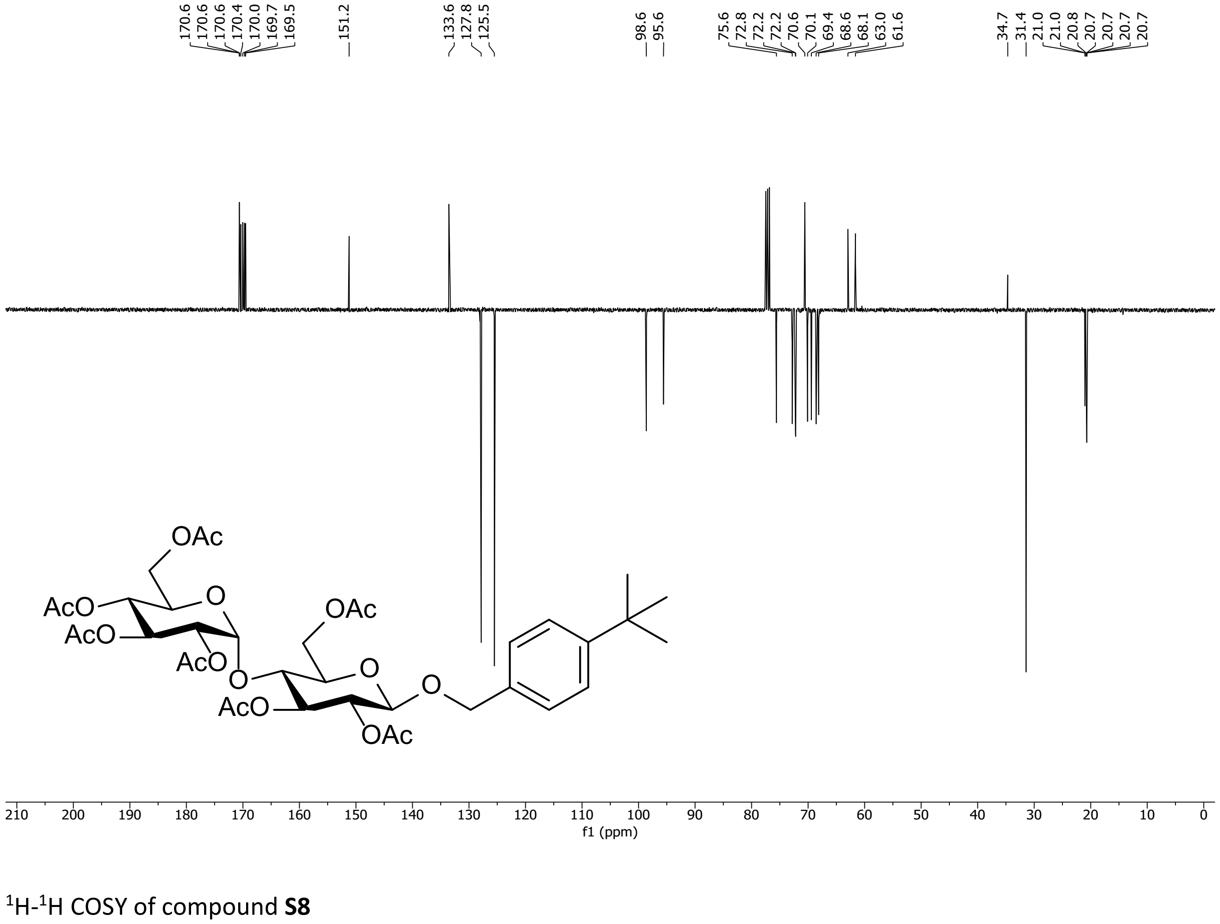

^{1}H-^{1}H COSY of compound **S8**

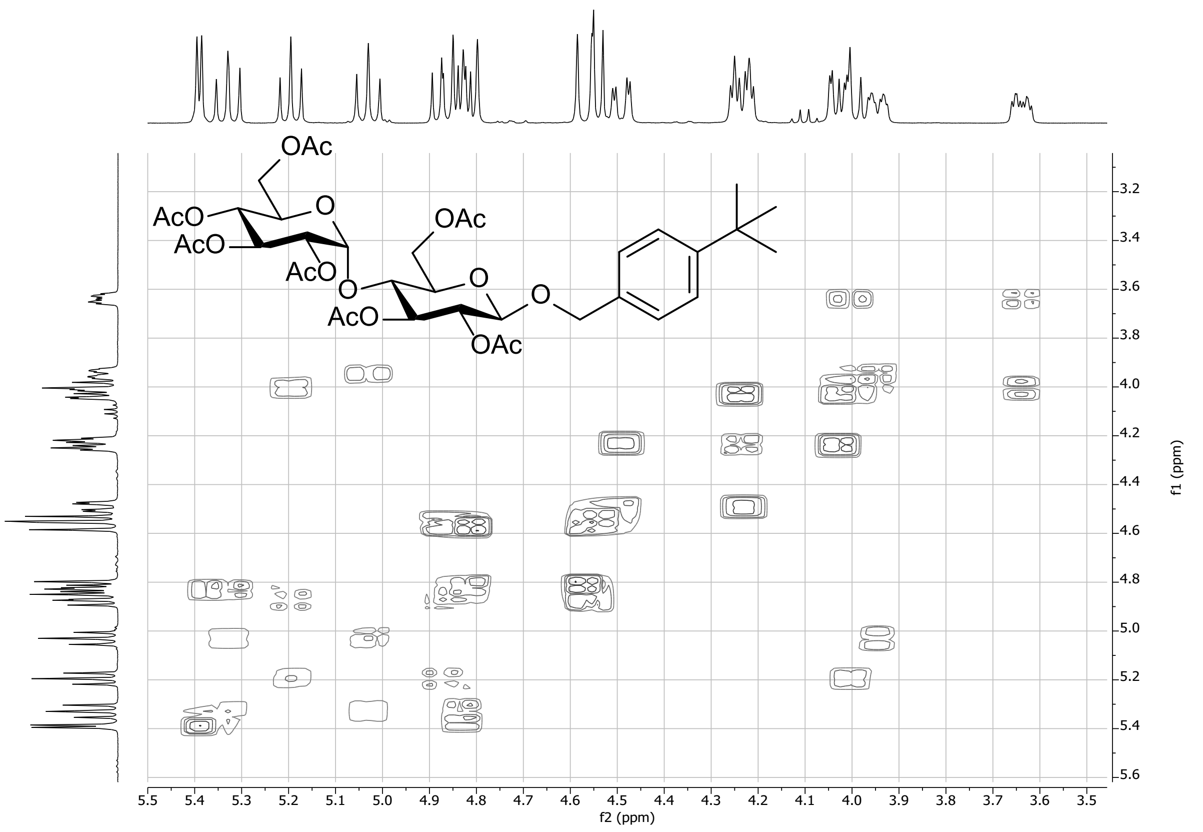

^1H-^{13}C HSQC of compound **S8**

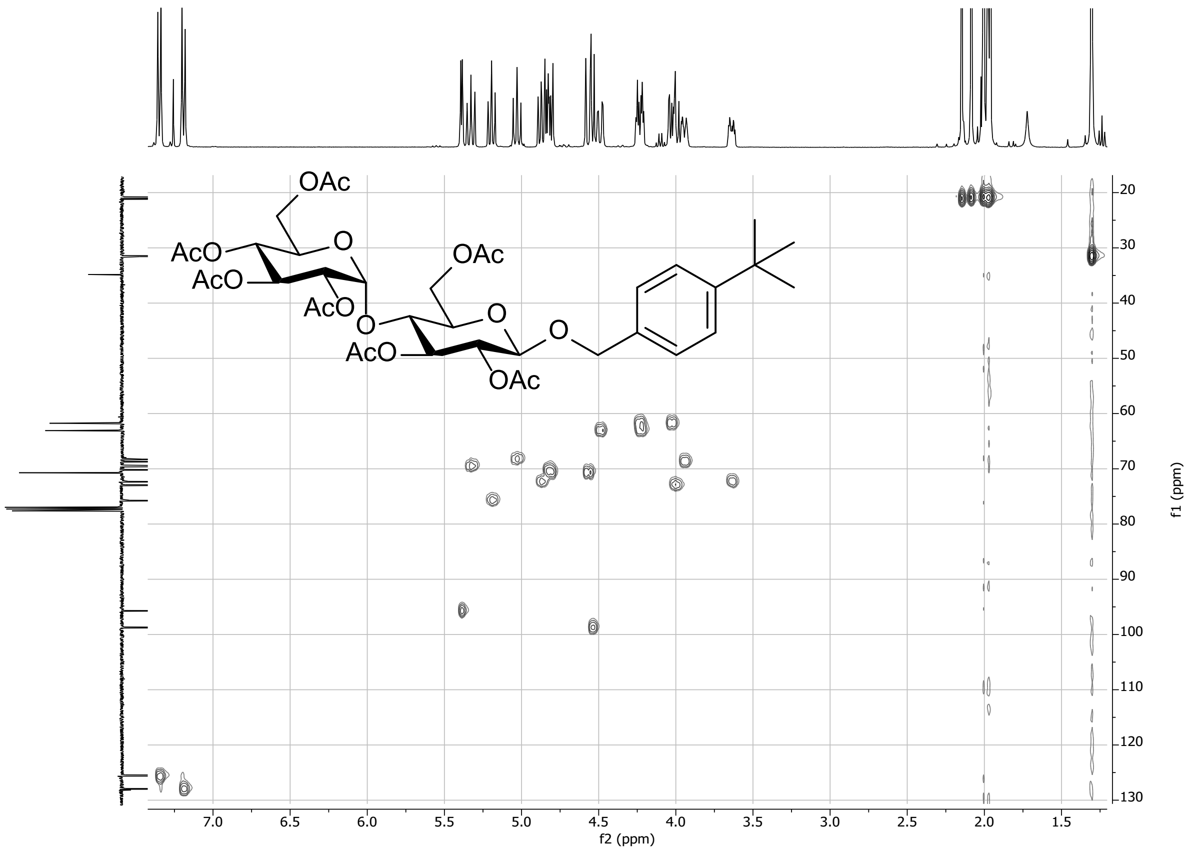

4-tert-butylbenzyl-β-D-maltoside (S9)

^{1}H NMR, 400 MHz, CD_3OD of compound **S9**

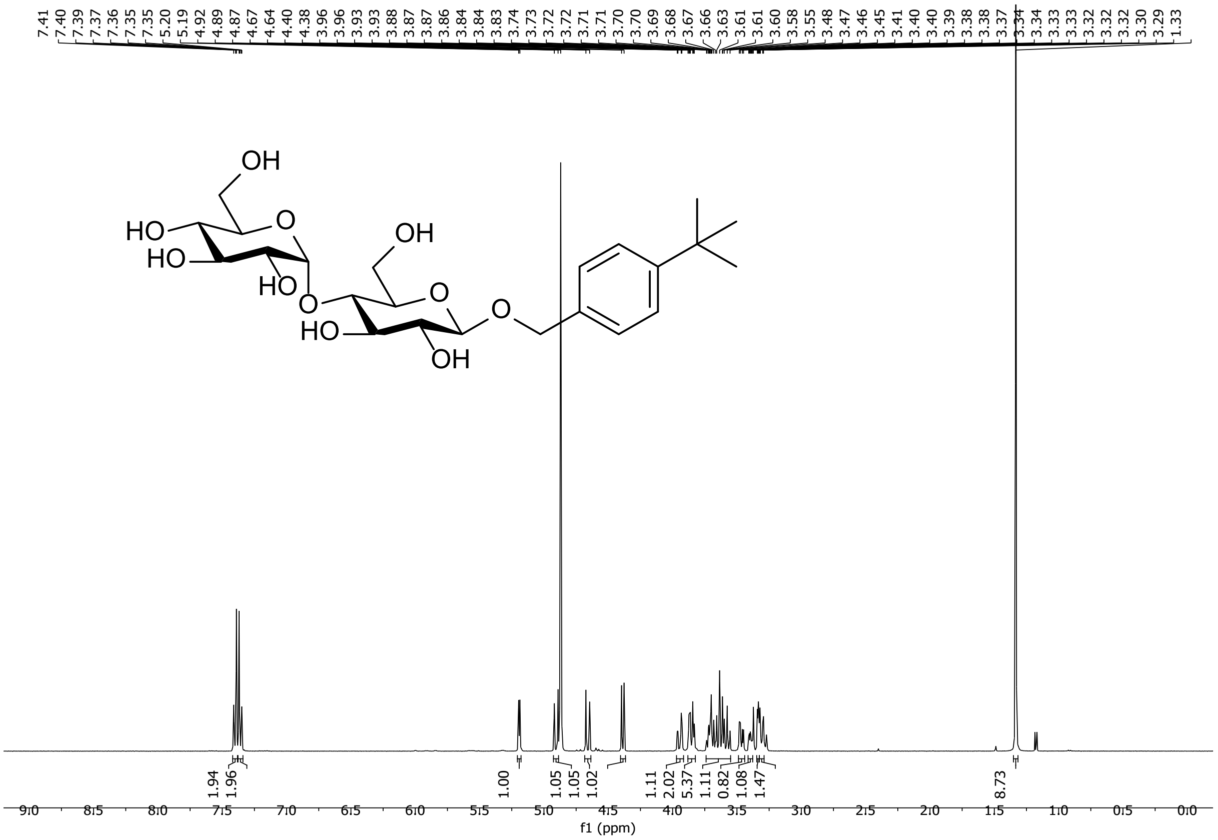

^{13}C NMR, 400 MHz, CD_3OD of compound **S9**

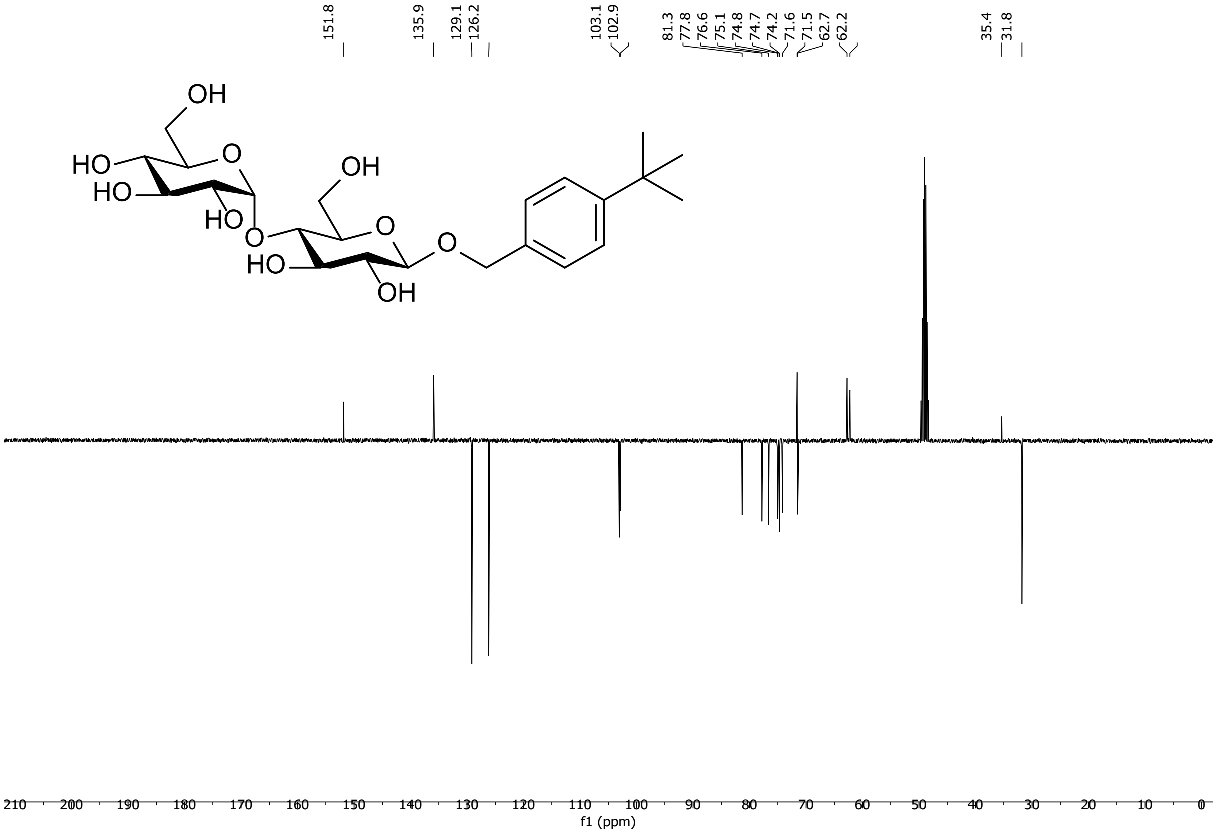

^{1}H-^{1}H COSY of compound **S9**

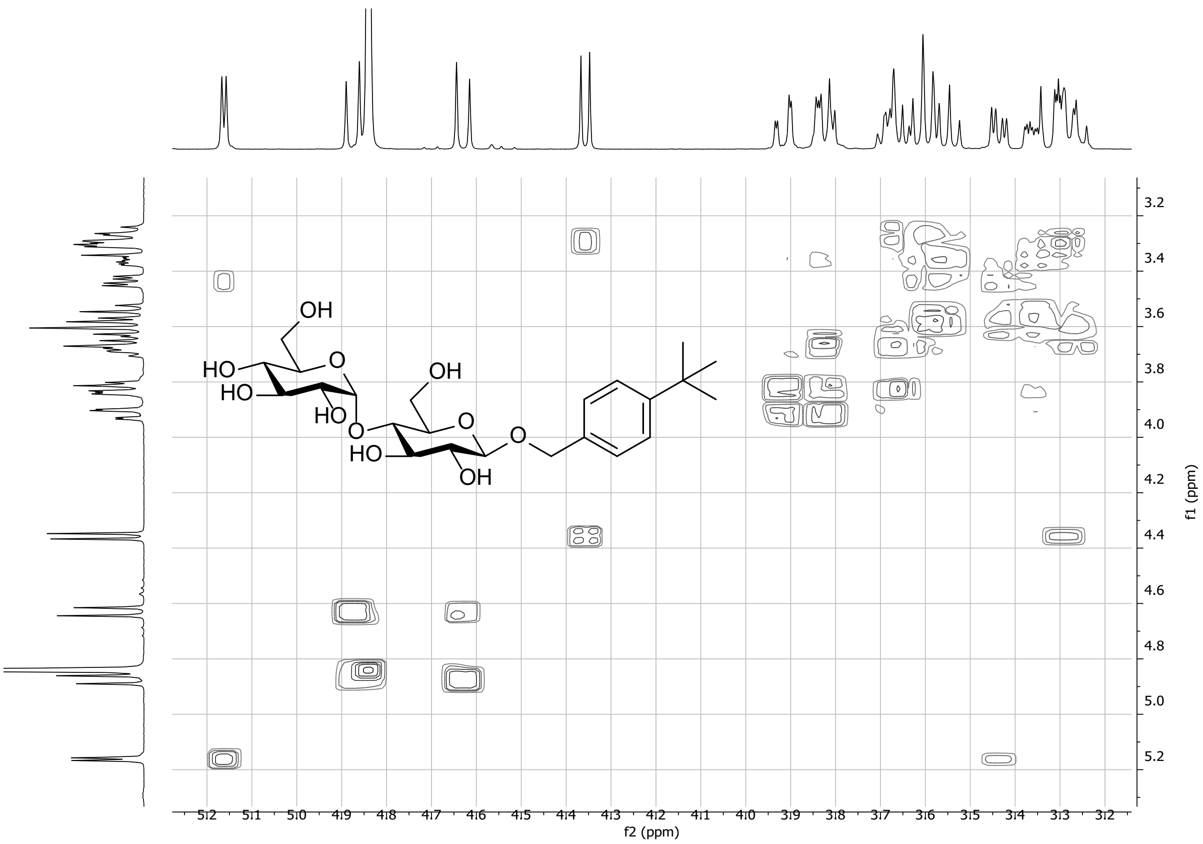

^{1}H-^{13}C HSQC of compound **S9**

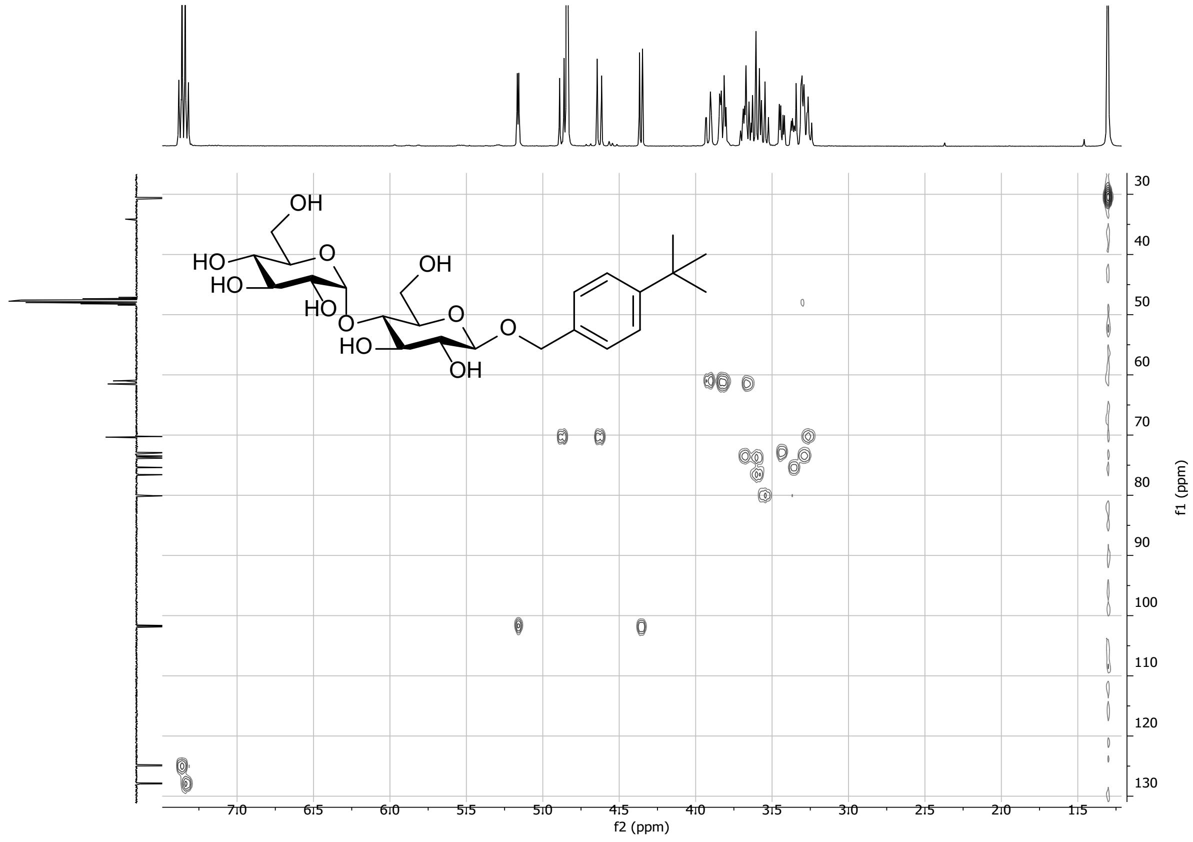

4-tert-butylbenzyl-β-3-ketomaltoside (1f)

^{1}H NMR, 400 MHz, CD_3OD of compound **1f**

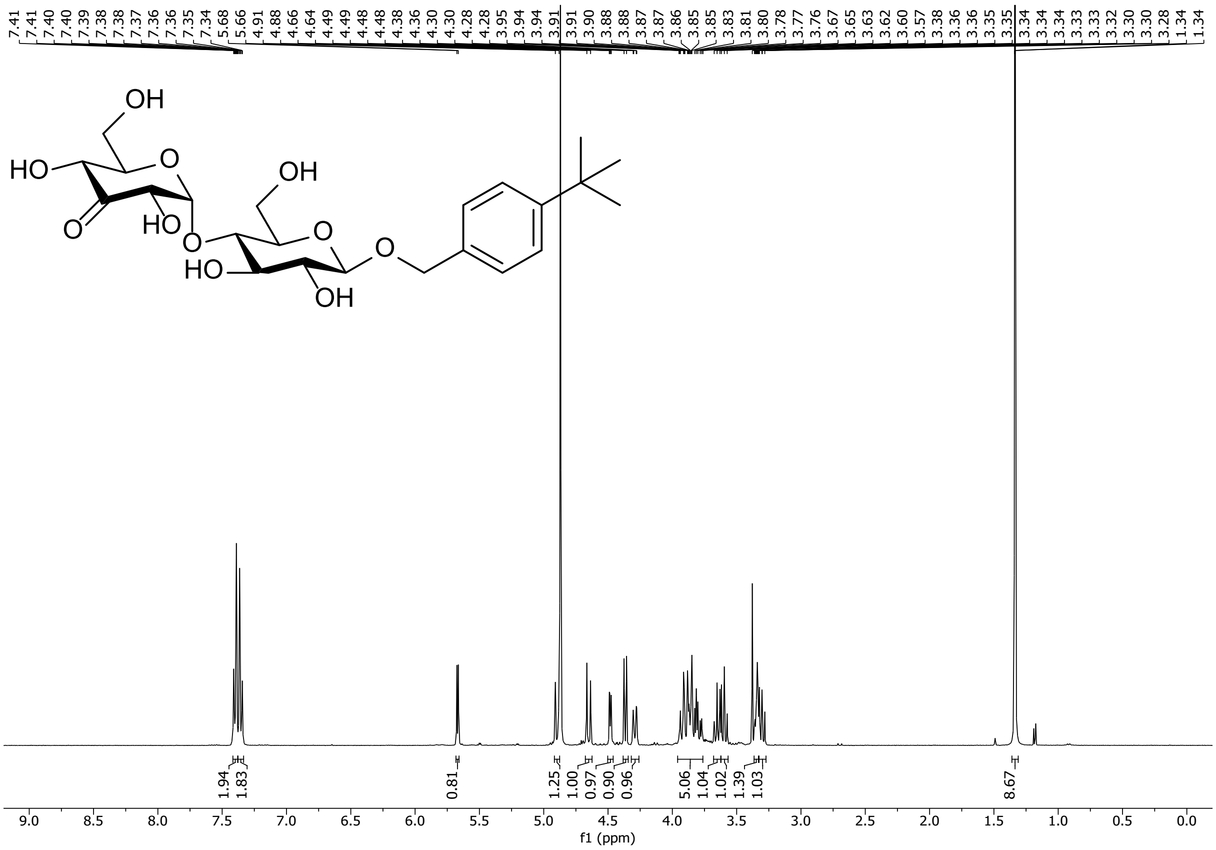

^{13}C NMR, 400 MHz, CD_3OD of compound **1f**

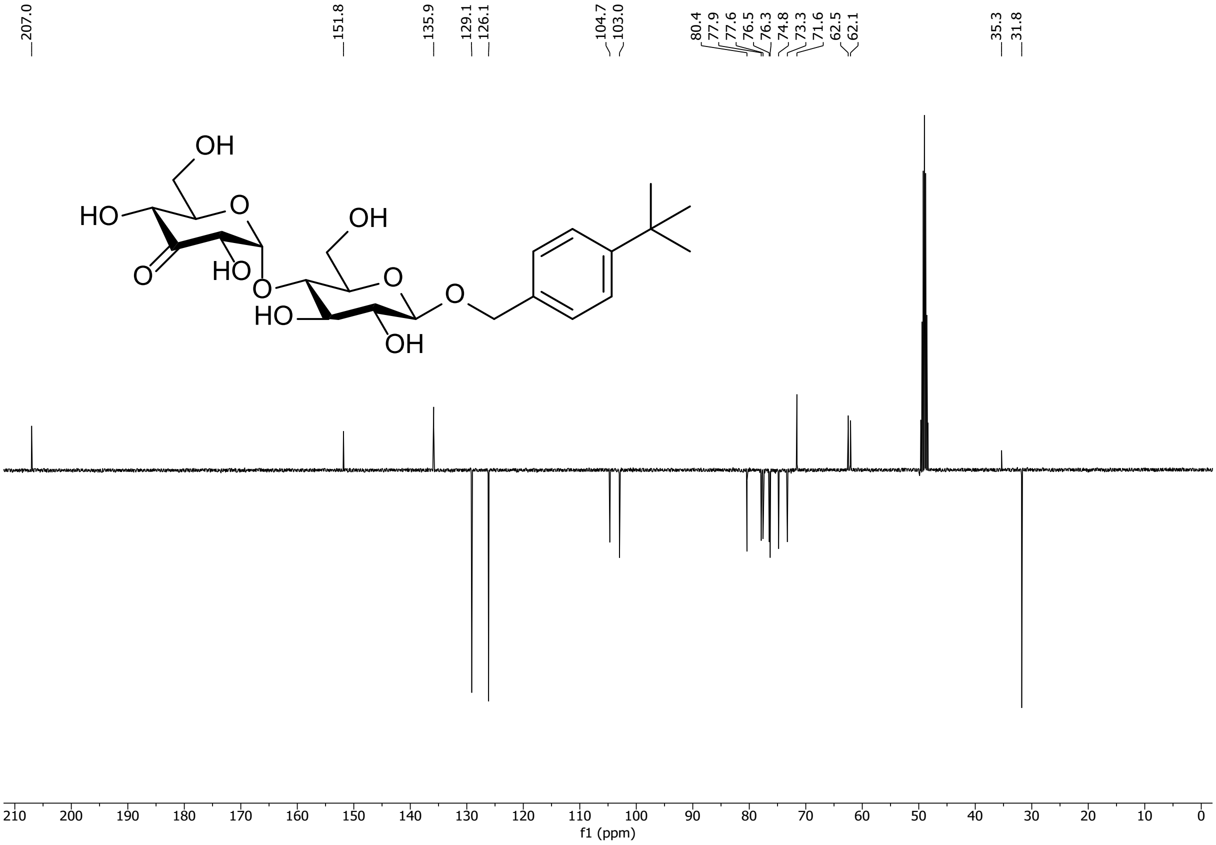

^1H-^1H COSY of compound **1f**

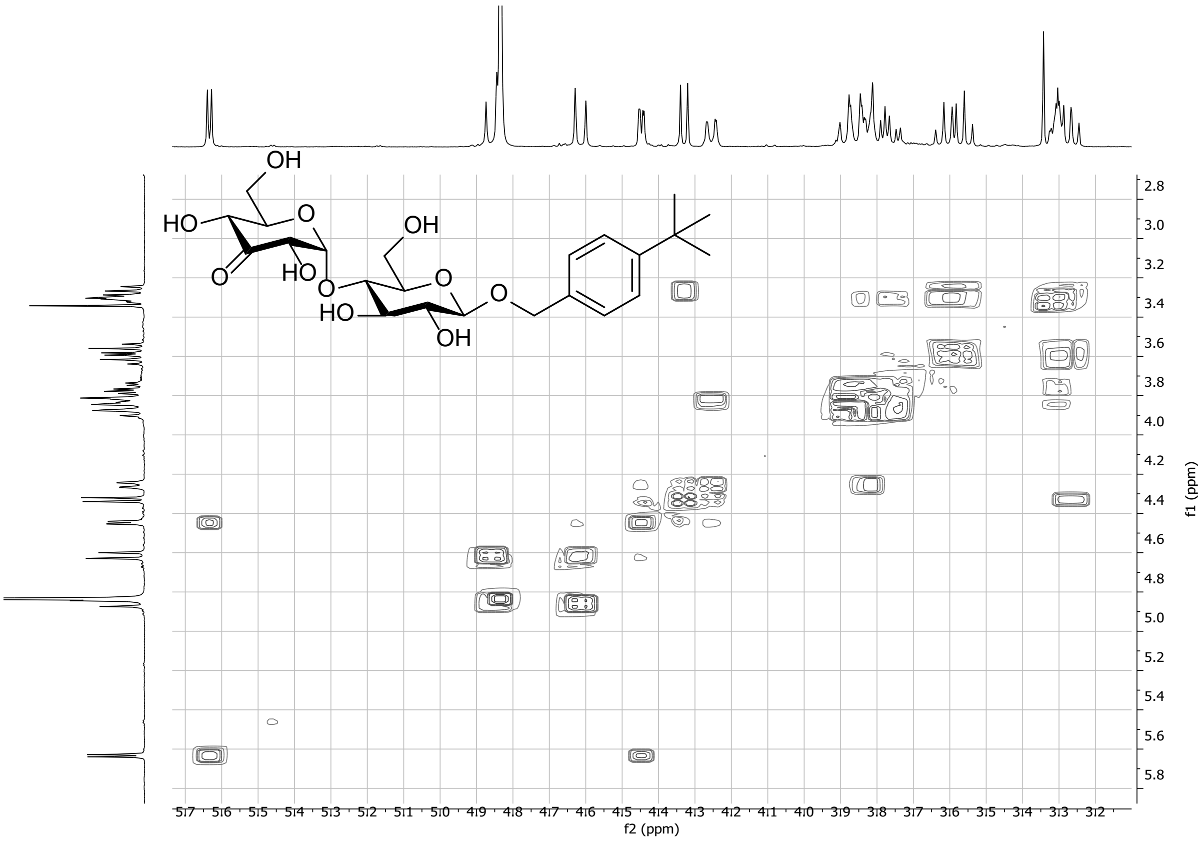

^1H-^{13}C HSQC of compound **1f**

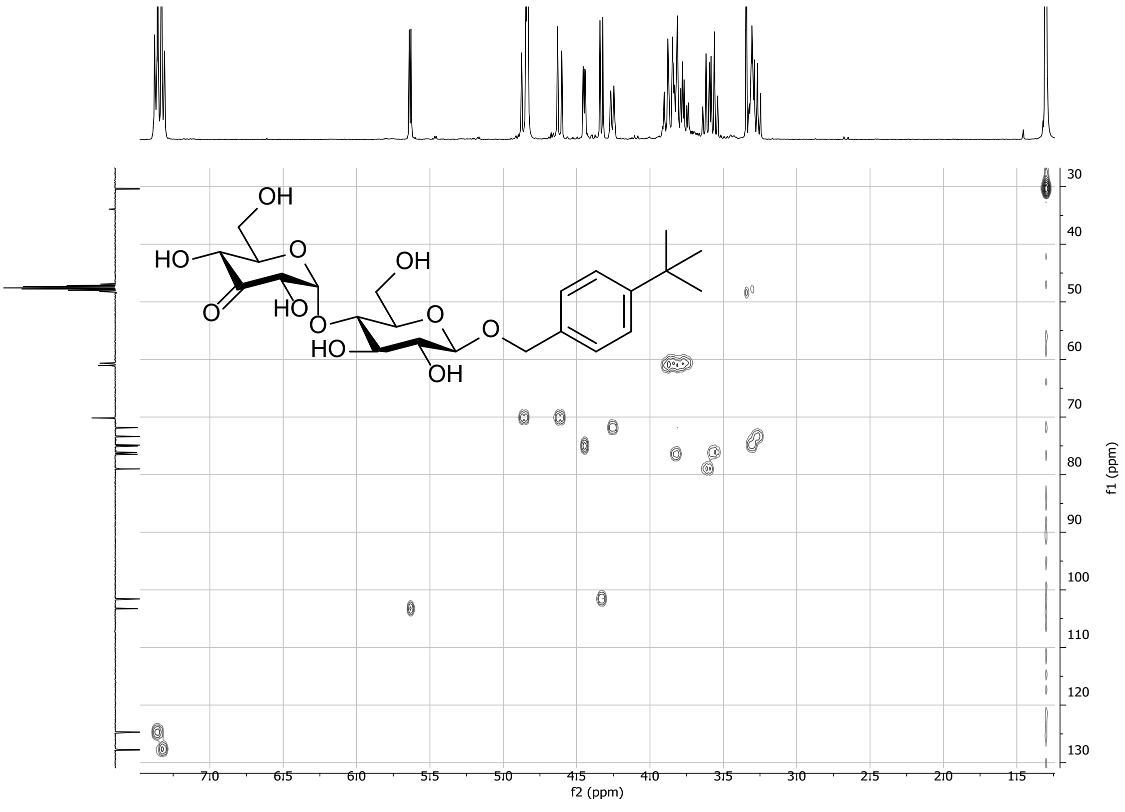

4-tert-butylbenzyl-β-3-(trityl)hydrazone maltoside (2f)

^{1}H NMR, 400 MHz, CD_3OD of compound **2f**

Mixture of *E* and *Z*: ratio ≈ 1:2

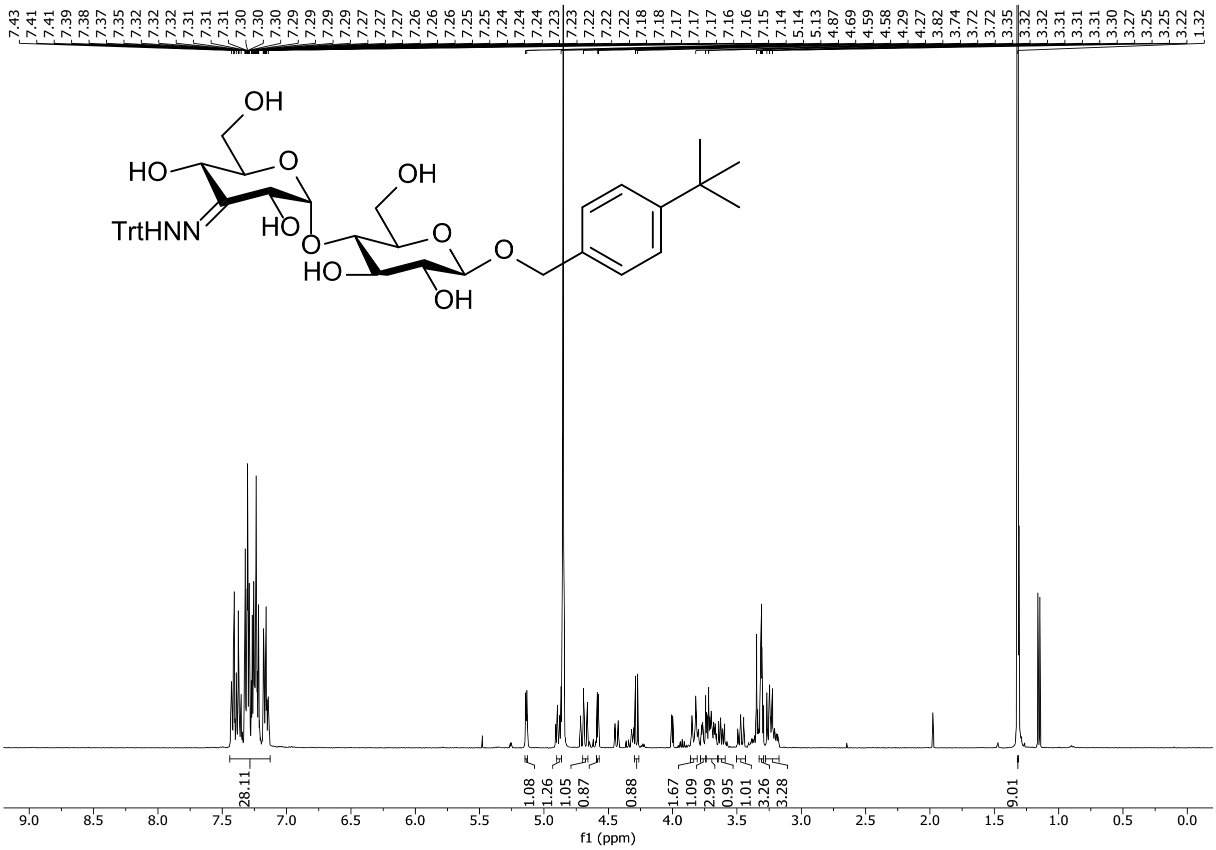

^{13}C NMR, 400 MHz, CD_3OD of compound **2f**

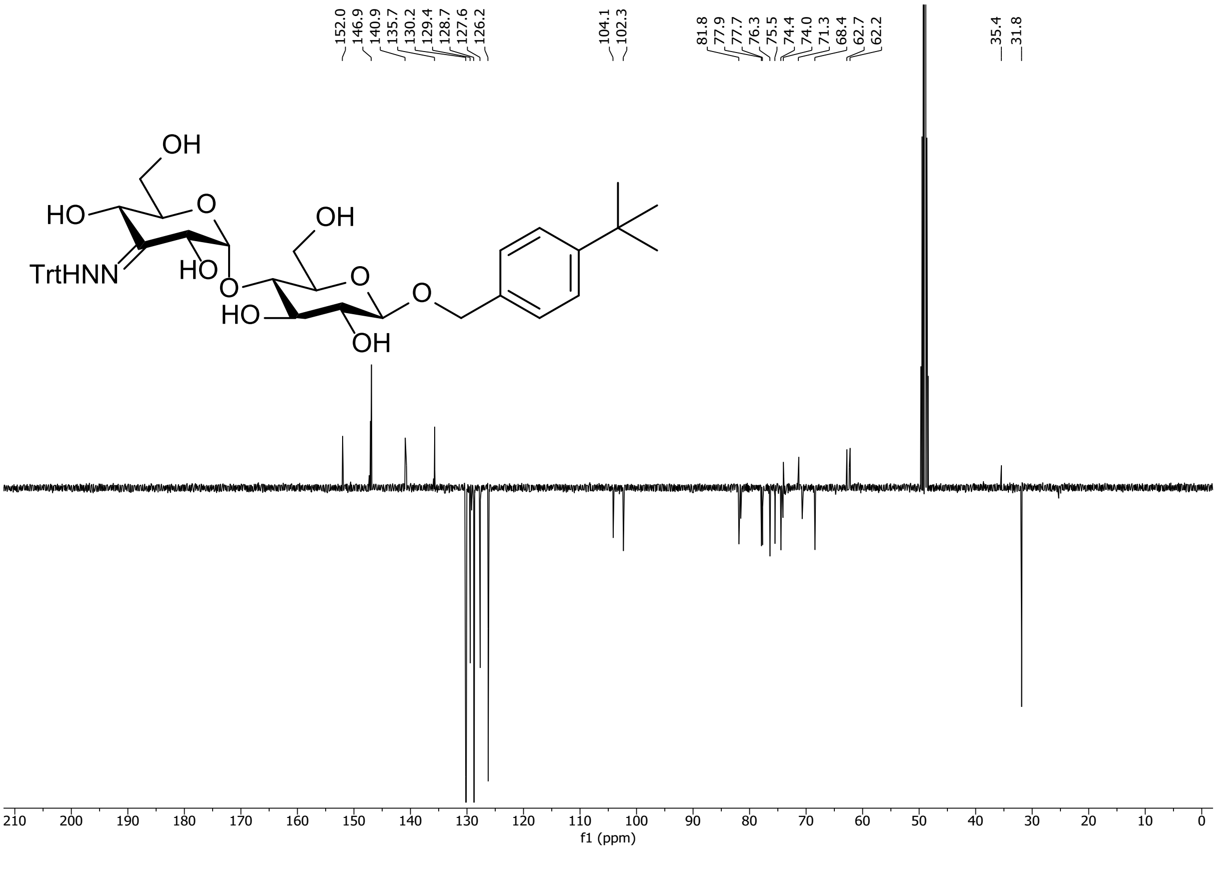

^1H-^1H COSY of compound **2f**

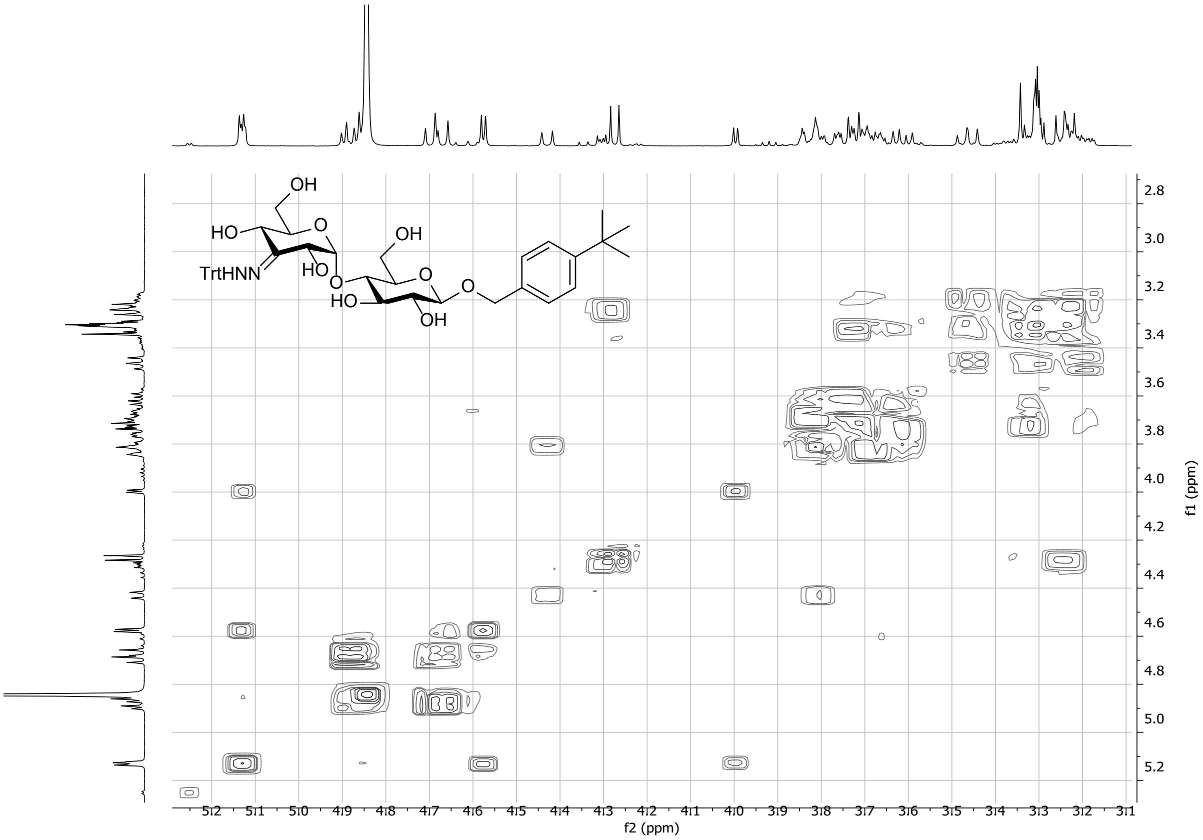

^1H-^{13}C HSQC of compound **2f**

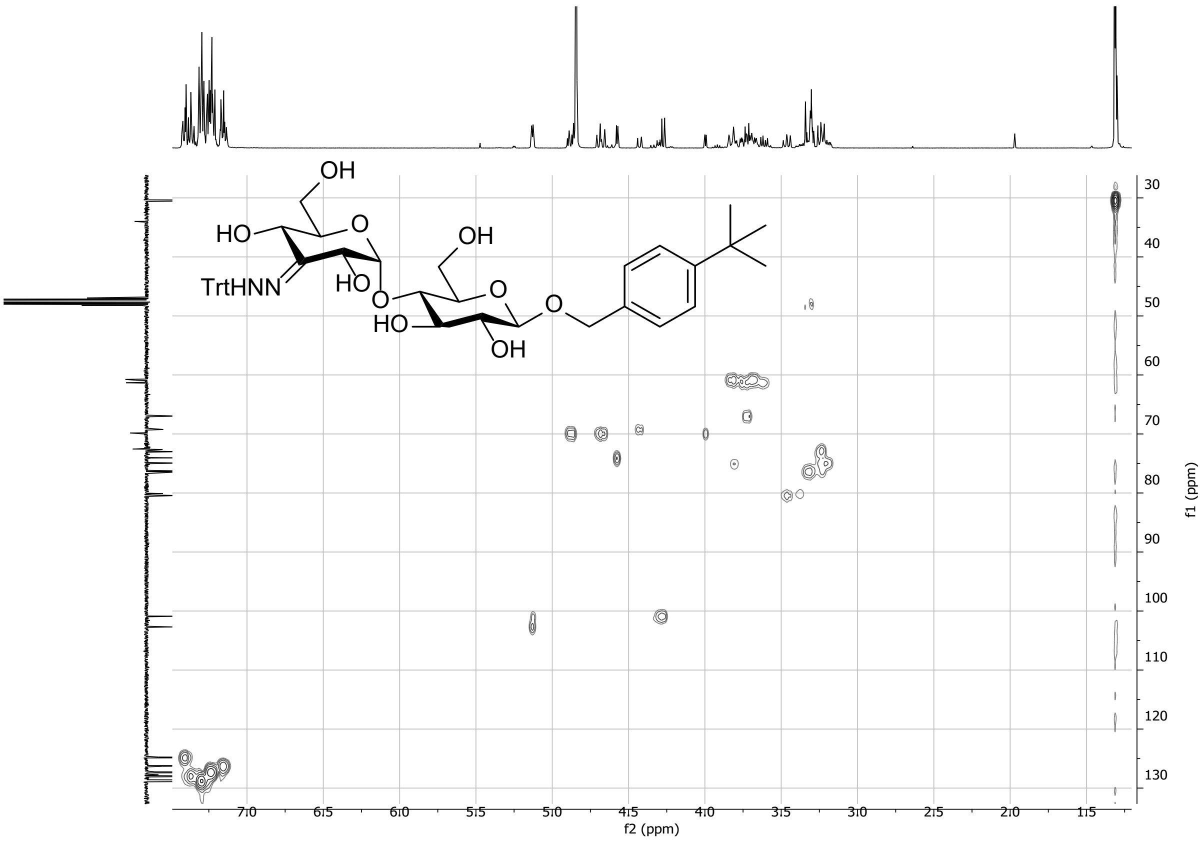

4-tert-butylbenzyl-3-chloro-3-deoxy-β-D-maltoside (3f)

Mixture of equatorial and axial, 3-equtorial : 3-axial ≈ 1:4.4

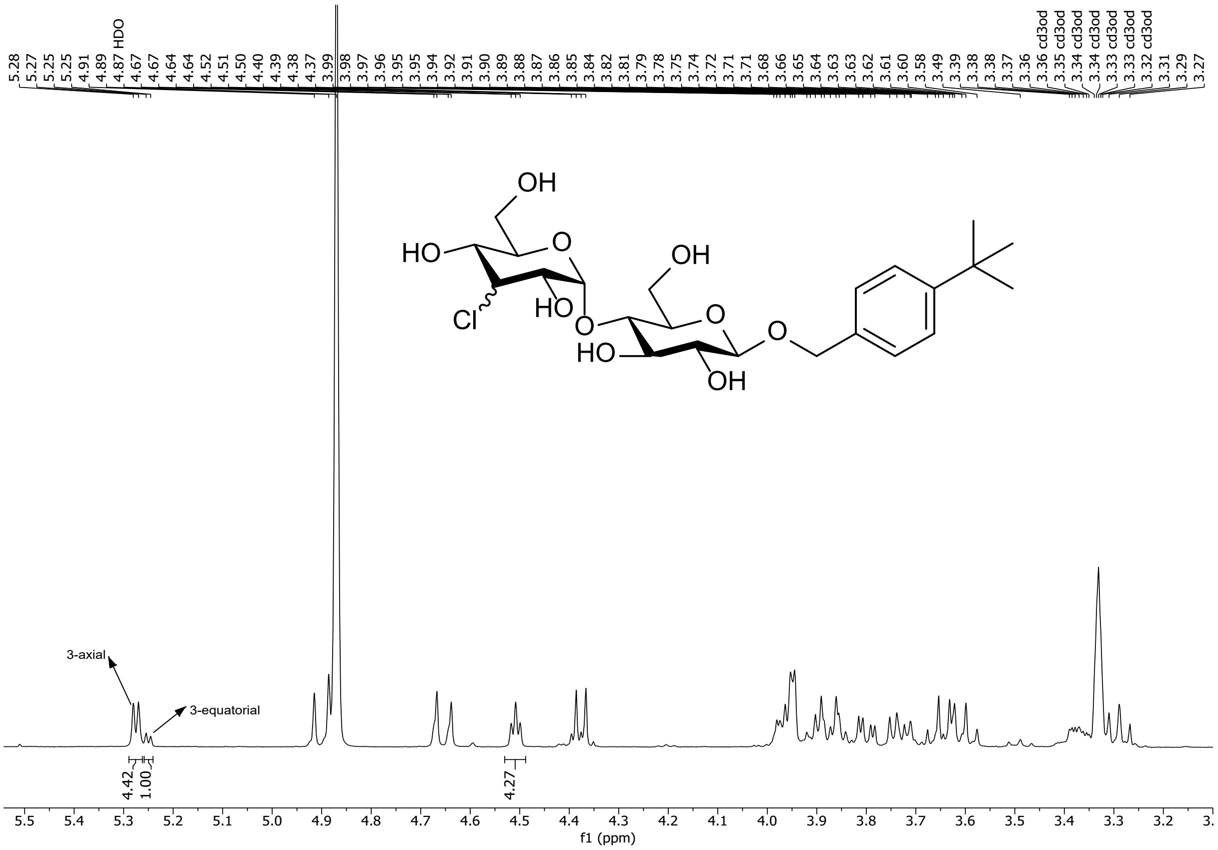

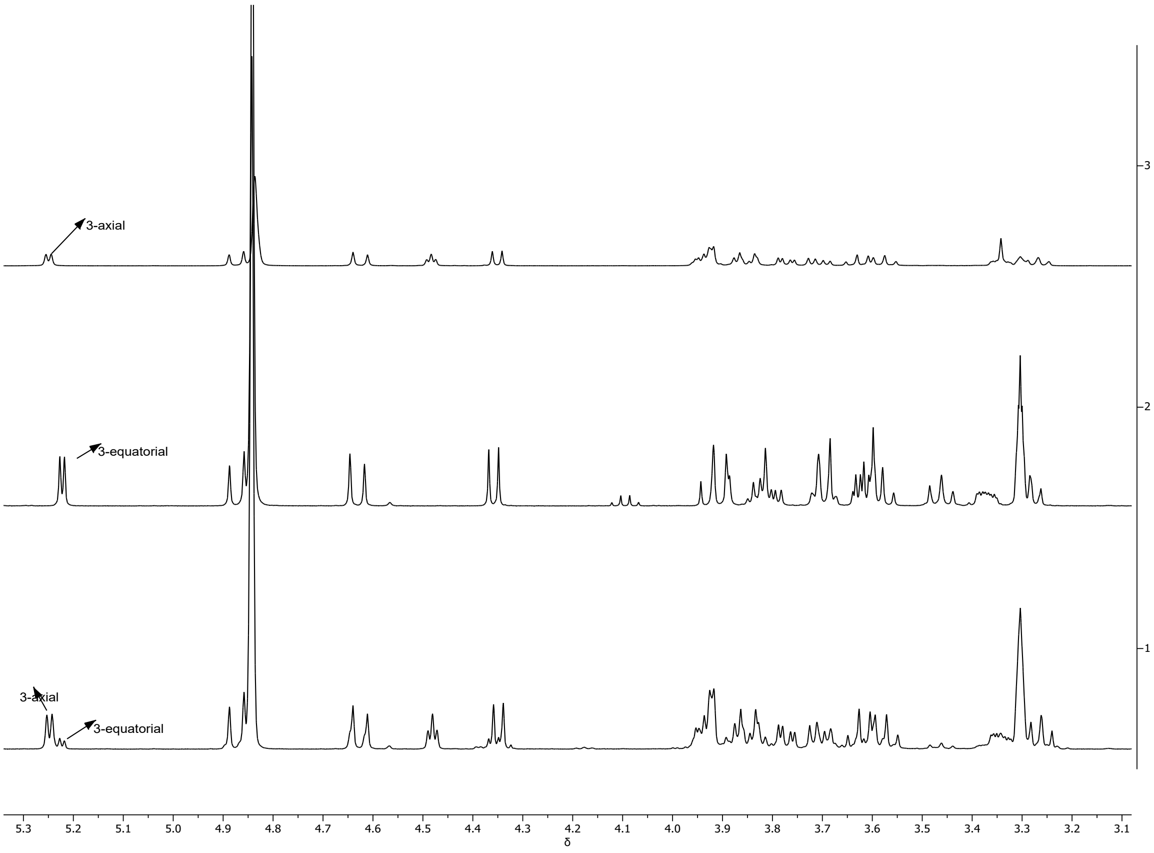

^{1}H NMR, 400 MHz, CD_3OD of compound **3f: equatorial**

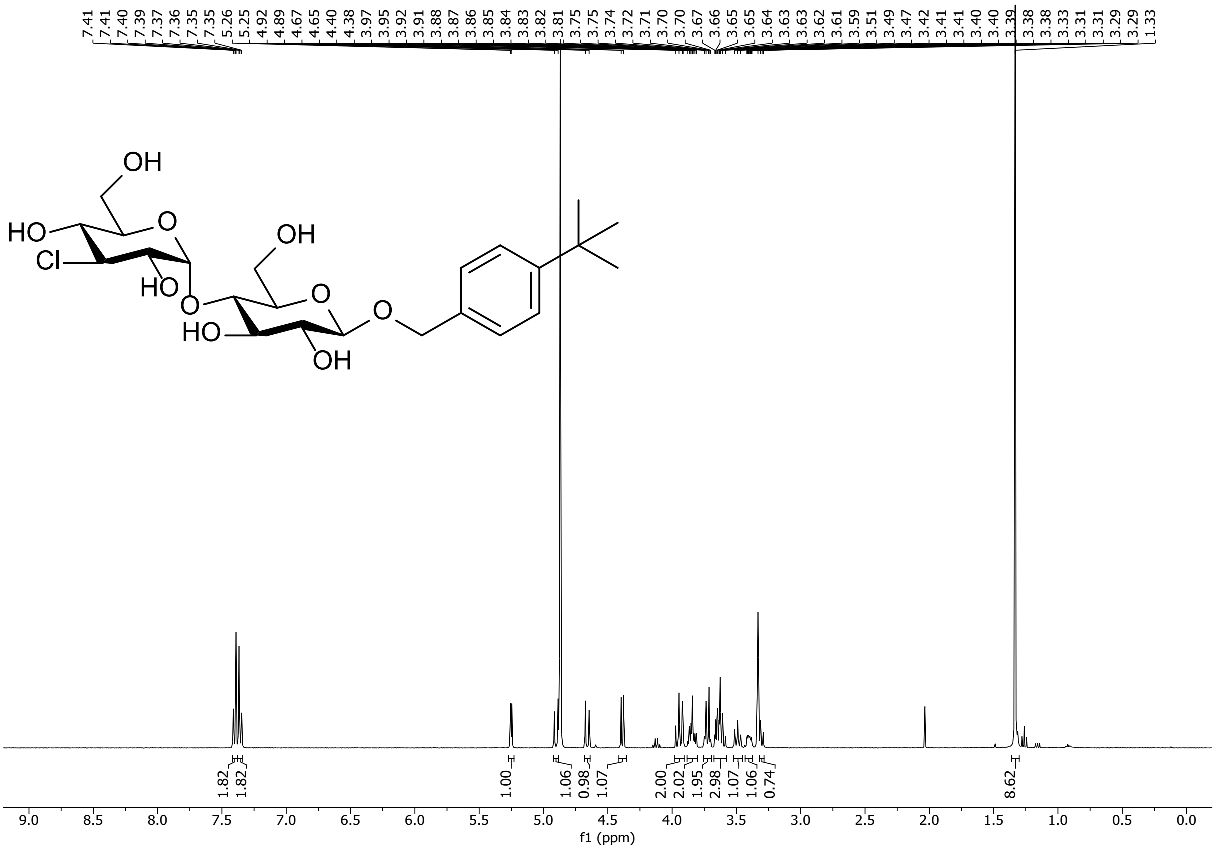

^{13}C NMR, 400 MHz, CD_3OD of compound **3f: equatorial**

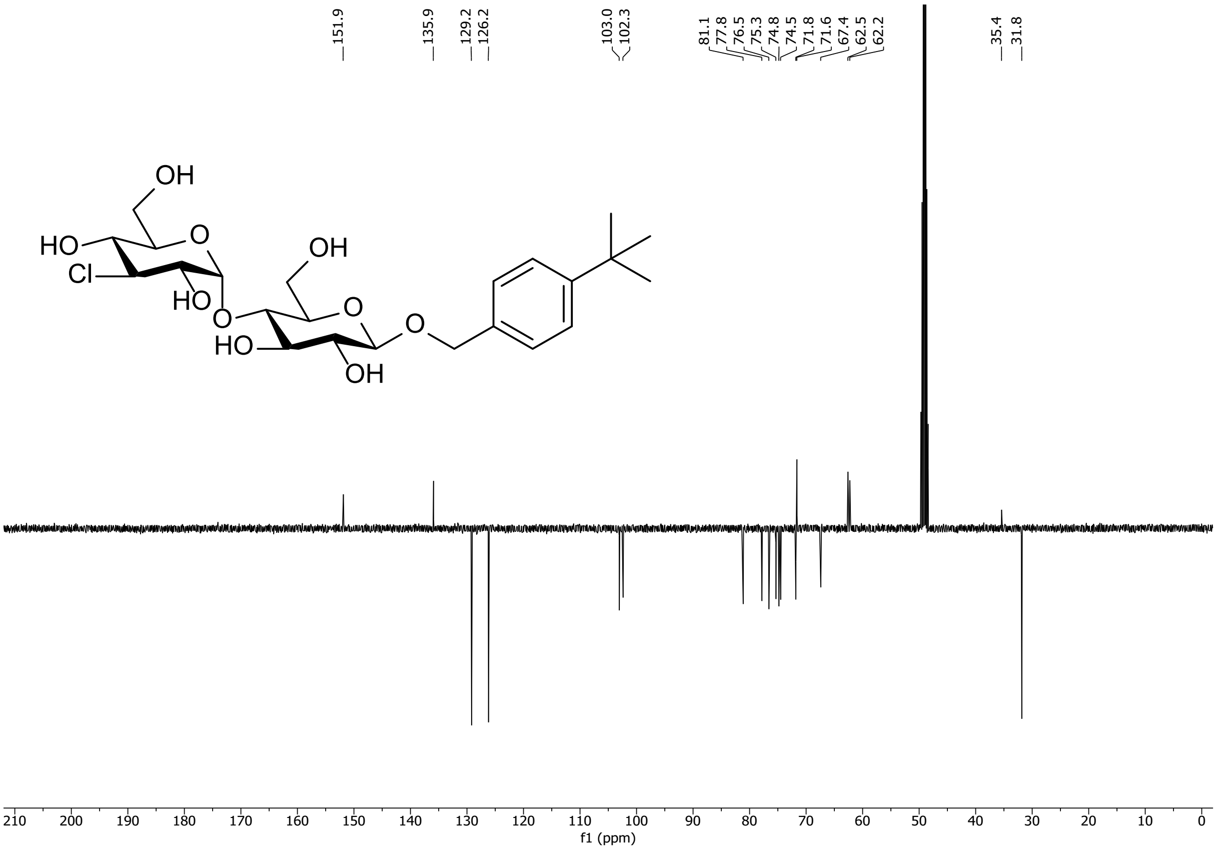

^{1}H-^{1}H COSY of compound **3f: equatorial**

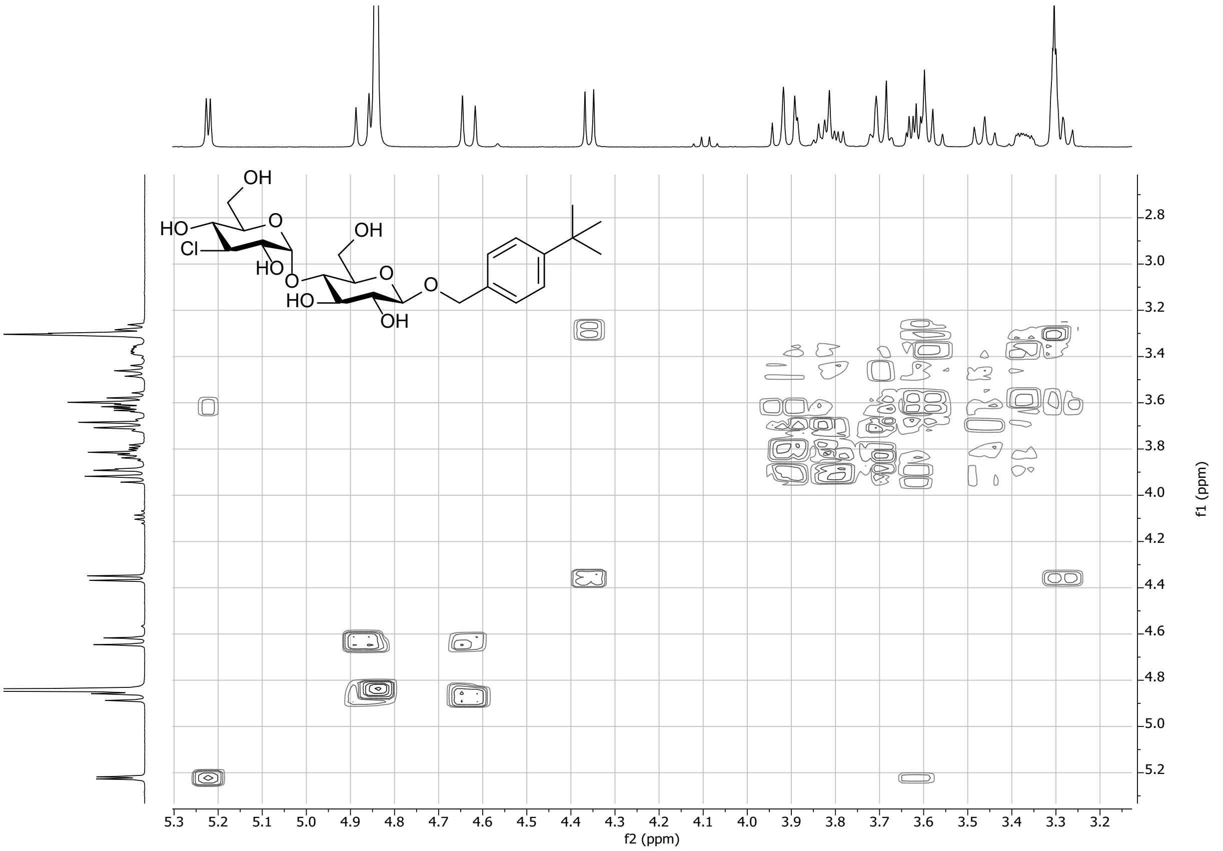

^{1}H-^{13}C HSQC of compound **3f: equatorial**

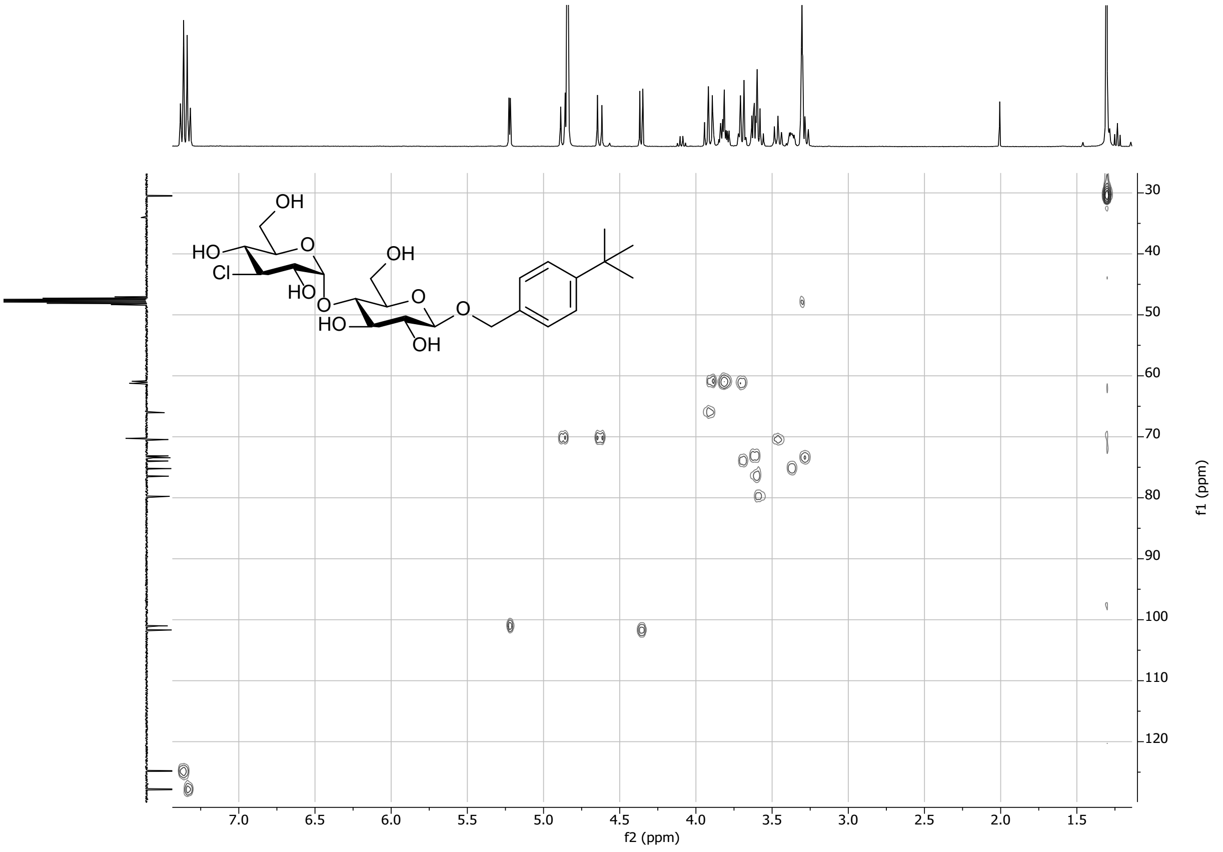

^{1}H NMR, 400 MHz, CD_3OD of compound **3f: axial**

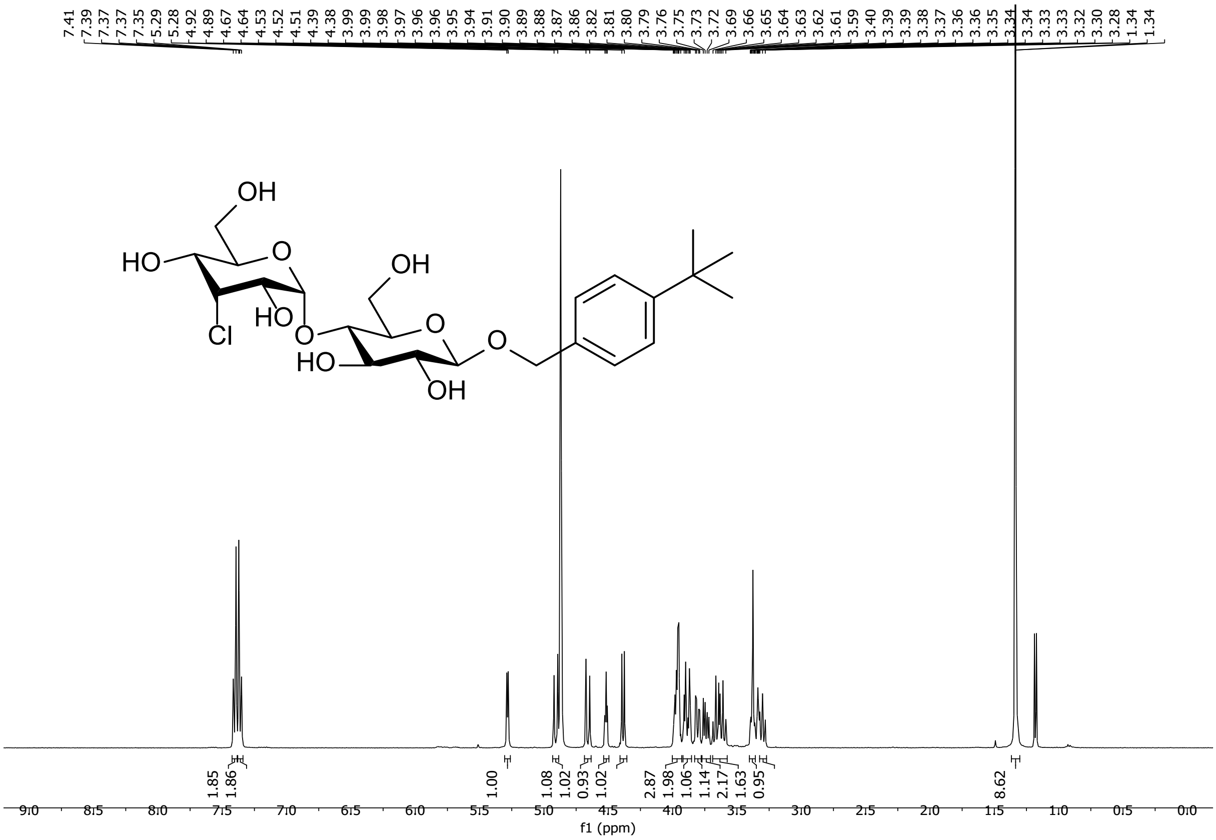

^{13}C NMR, 400 MHz, CD_3OD of compound **3f: axial**

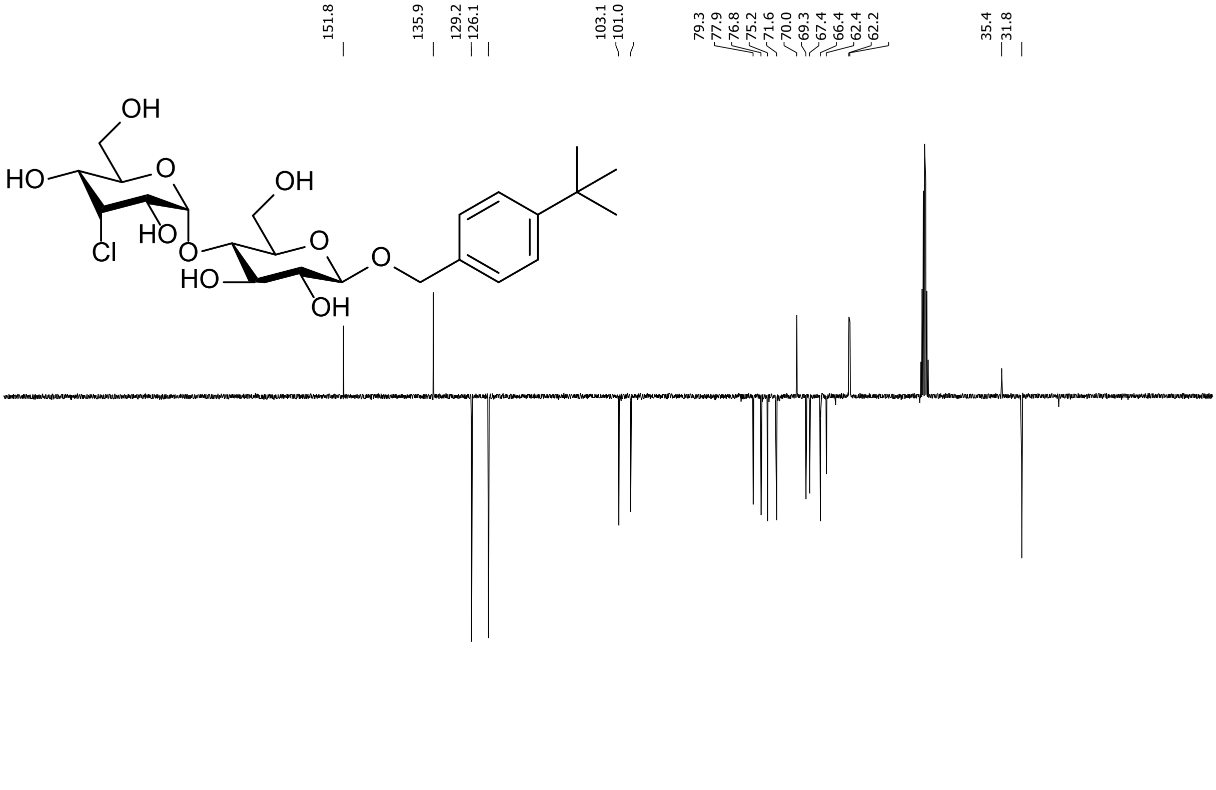

^{1}H-^{1}H COSY of compound **3f: axial**

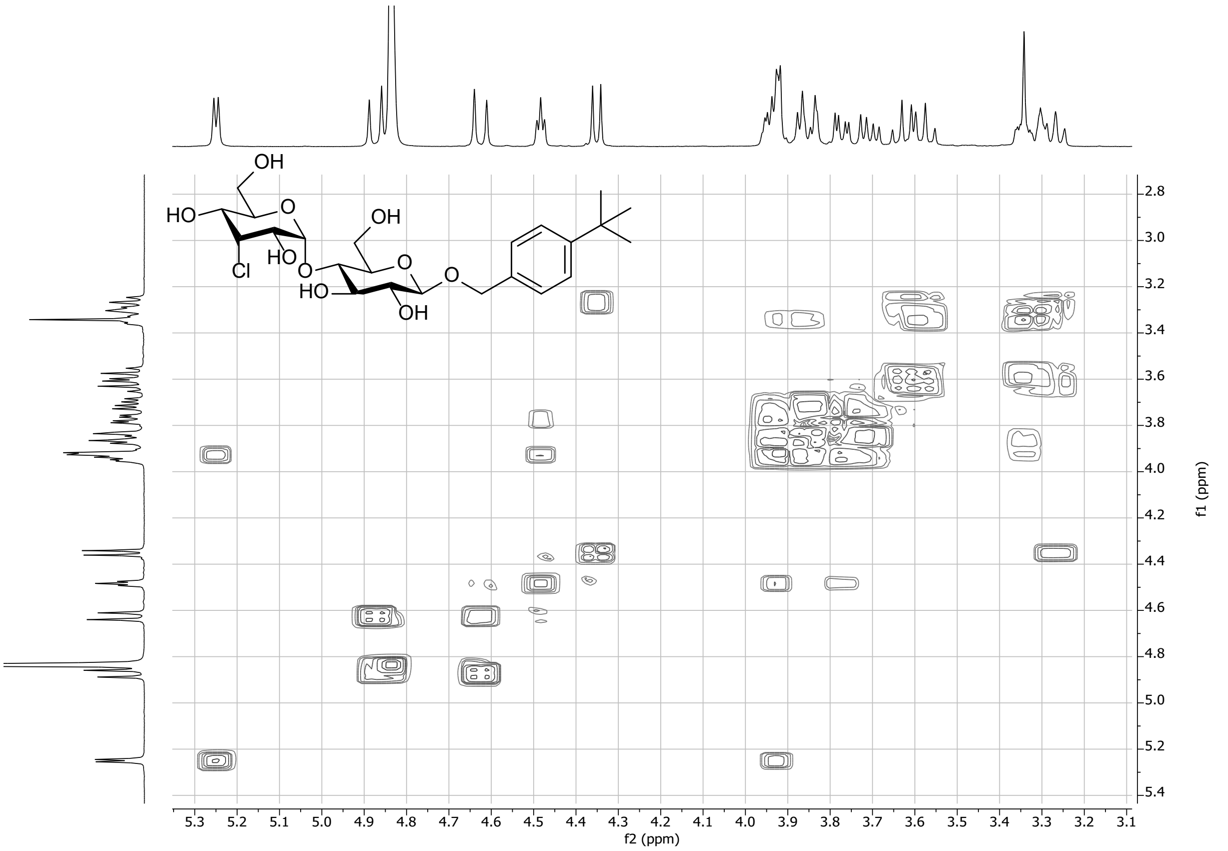

^{1}H-^{13}C HSQC of compound **3f: axial**

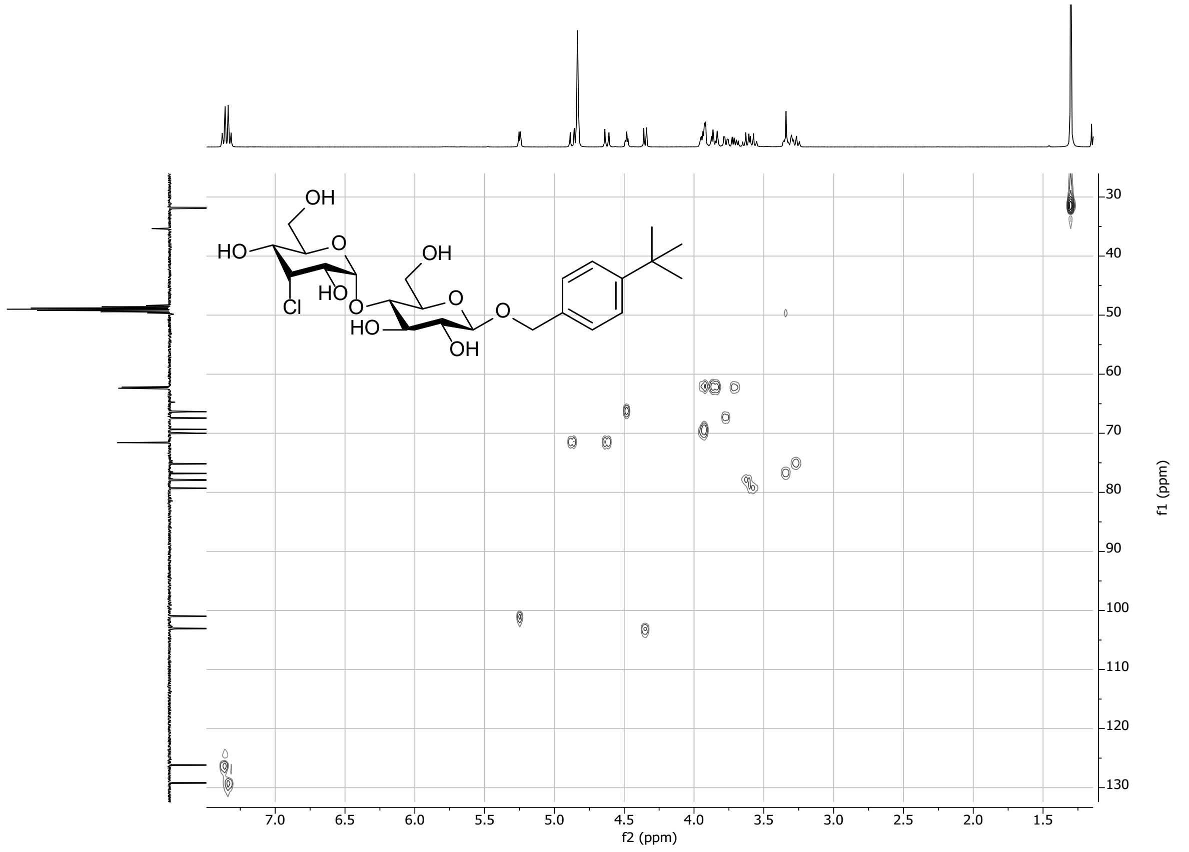

NMR spectra of β-Glc derivatives

Methyl 3-keto-β-D-glucopyranoside (1g)

^{1}H NMR, 400 MHz, CD_3OD of compound **1g**

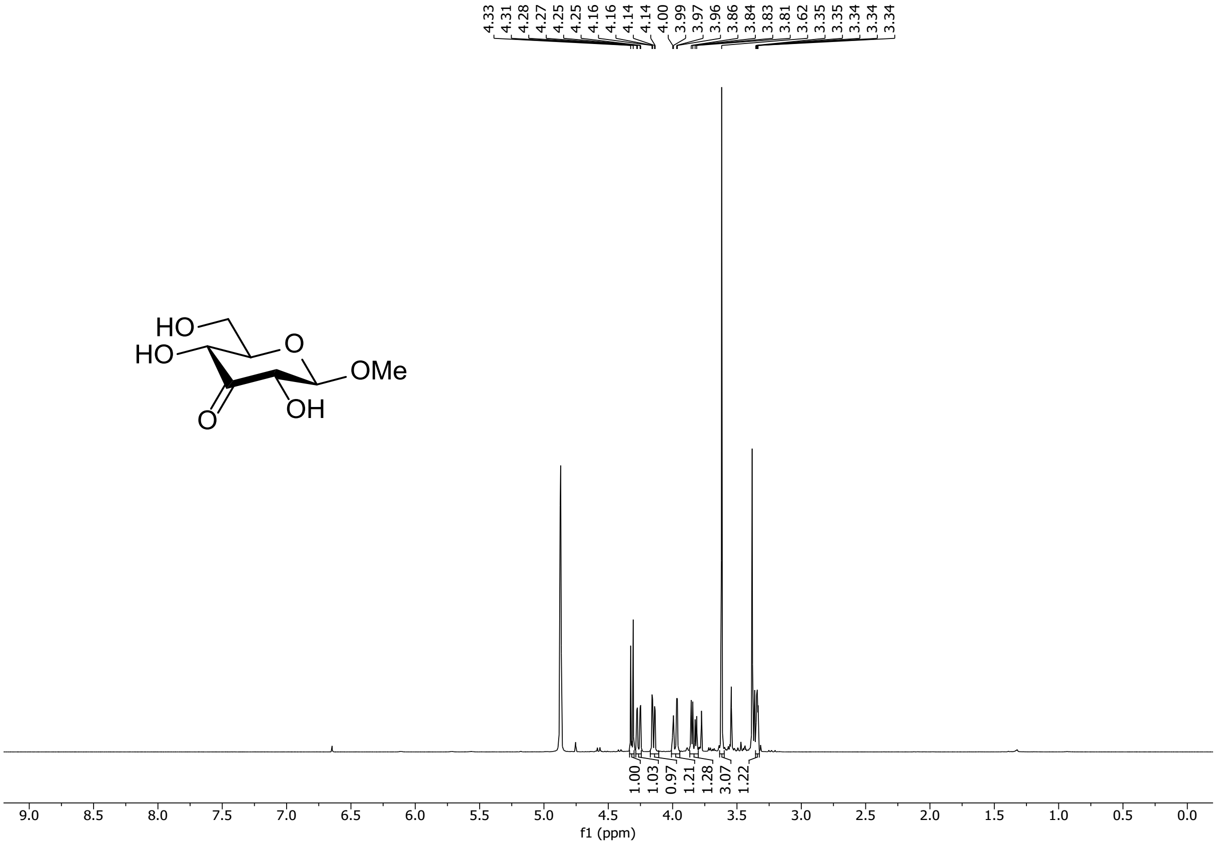

^{13}C NMR, 400 MHz, CD_3OD of compound **1g**

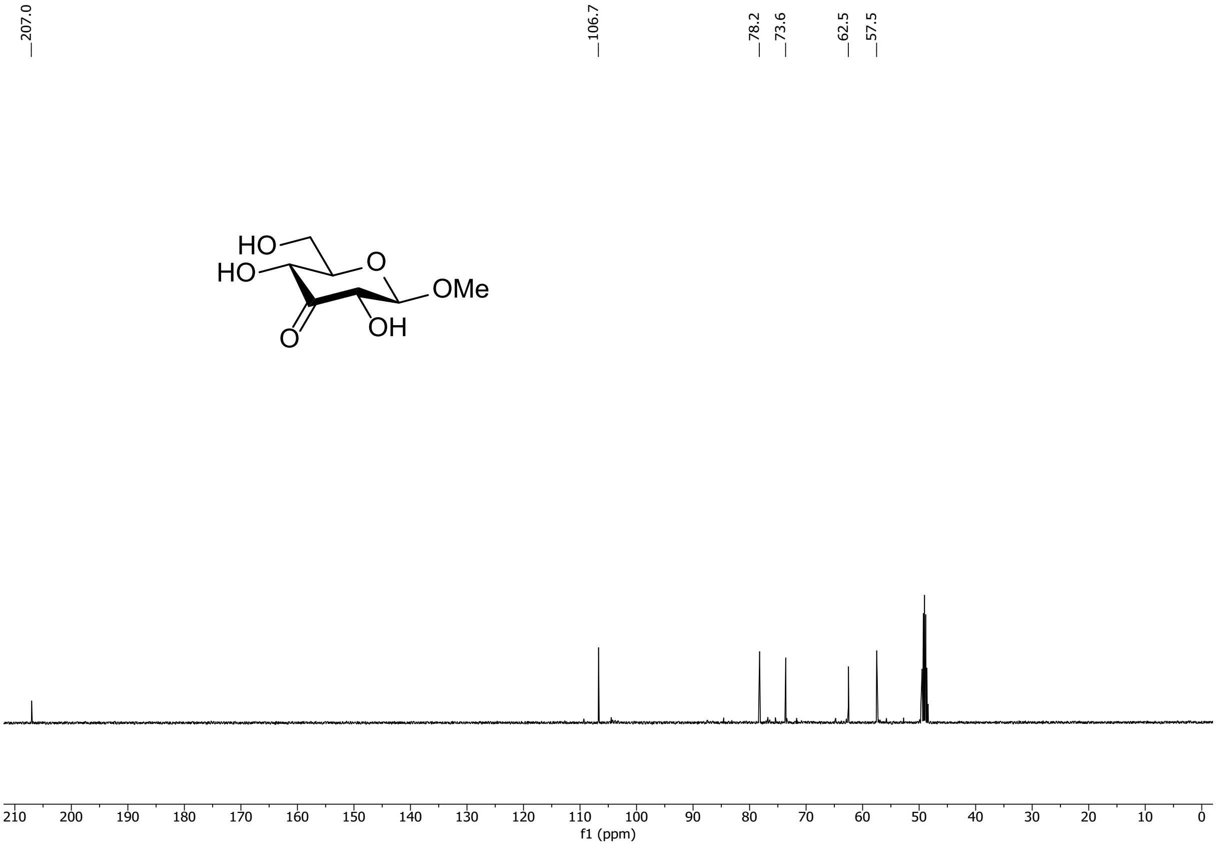

^1H-^1H COSY of compound **1g**

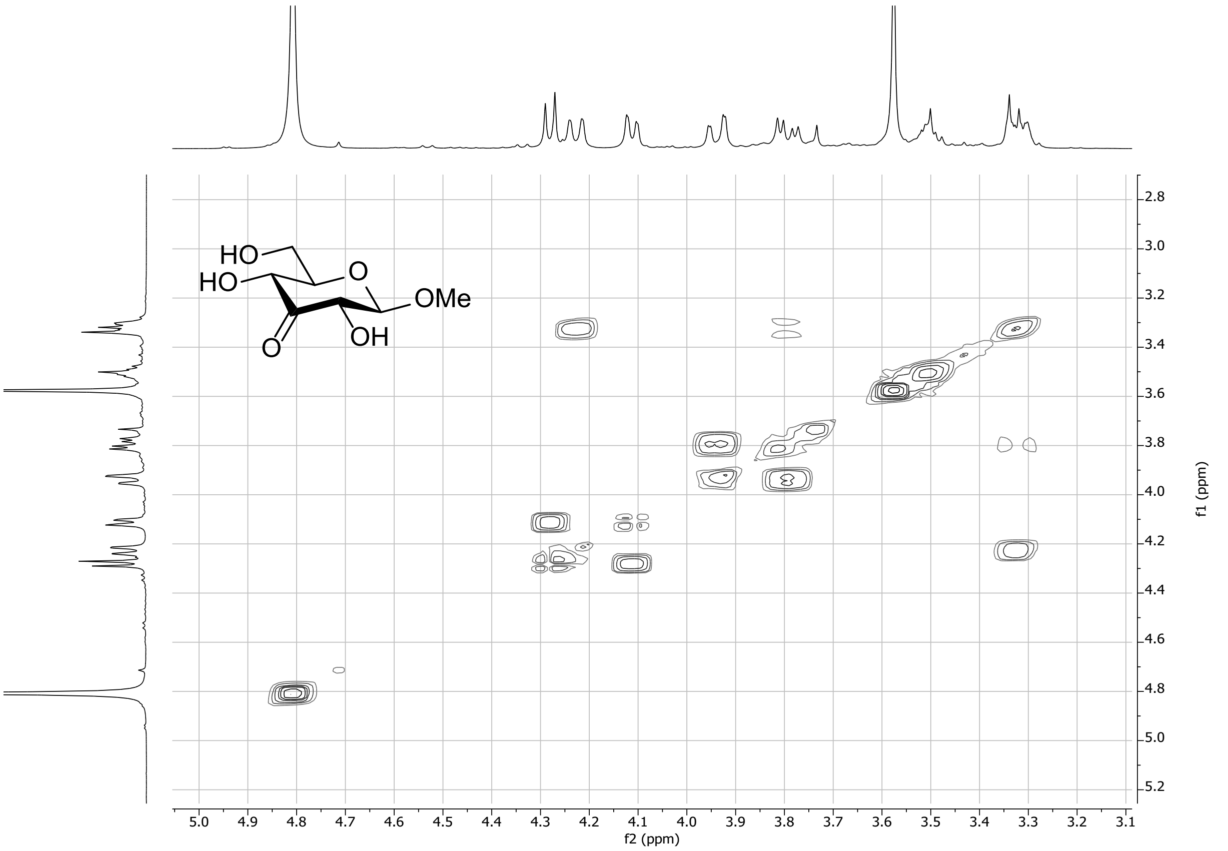

H-^{13}C HSQC of compound **1g**

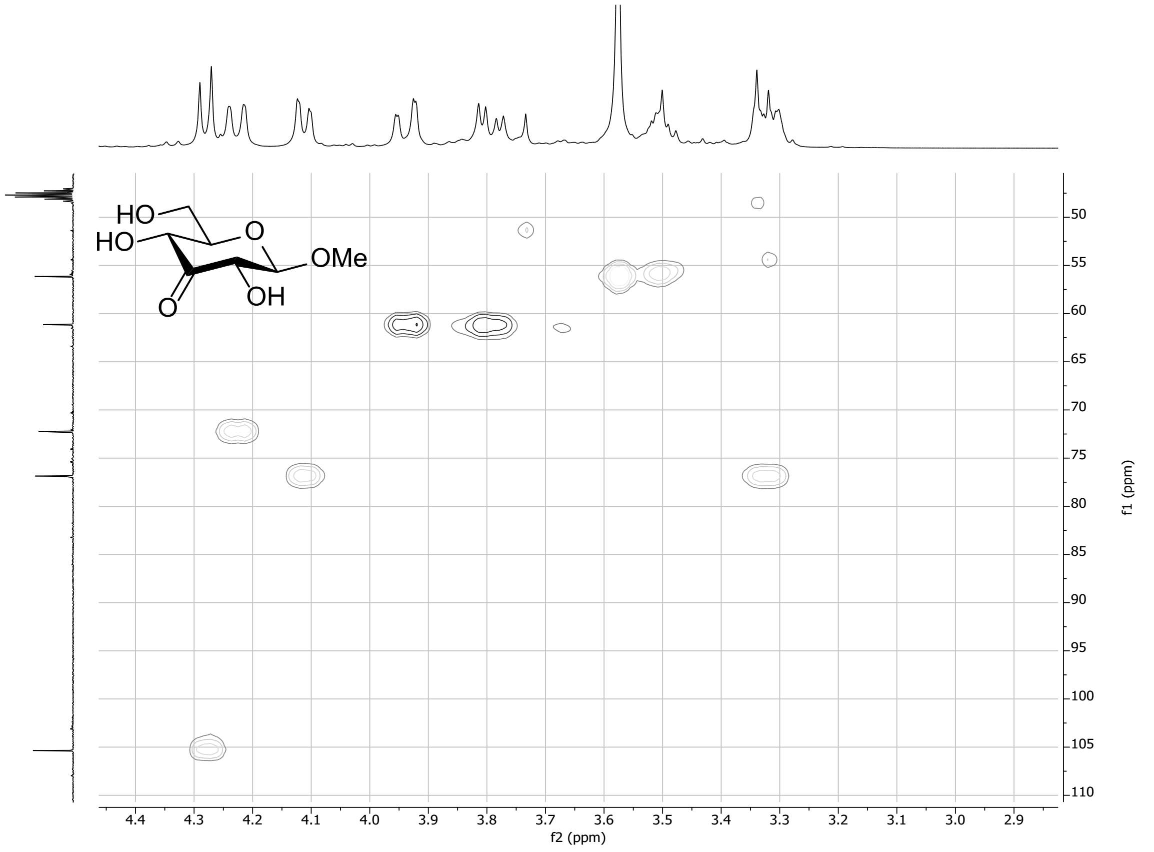

Methyl-3-(trityl)hydrazone-β-D-glucopyranoside (2g)

Mixture of *E* and *Z*: ratio ≈ 1:1.3

^{1}H NMR, 400 MHz, CD_3OD of compound **2g**

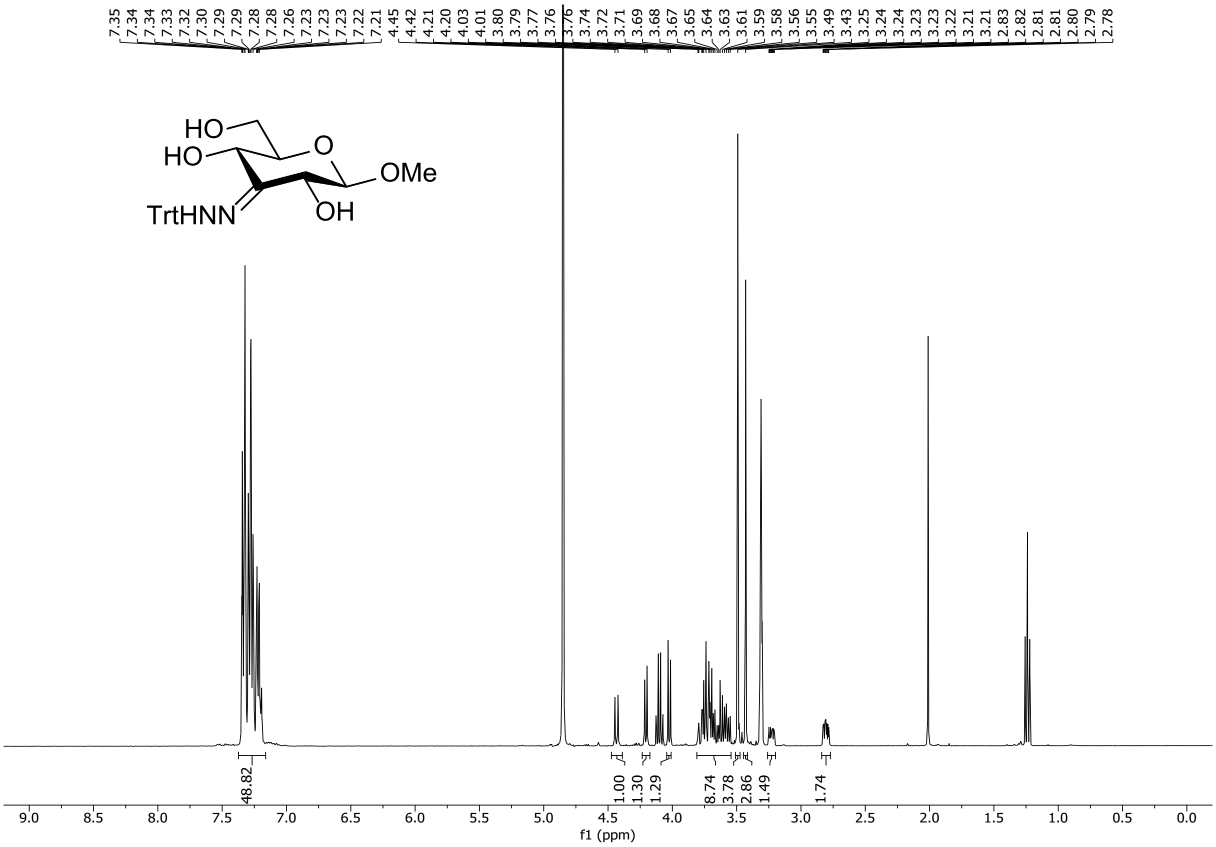

^{13}C NMR, 400 MHz, CD_3OD of compound **2g**

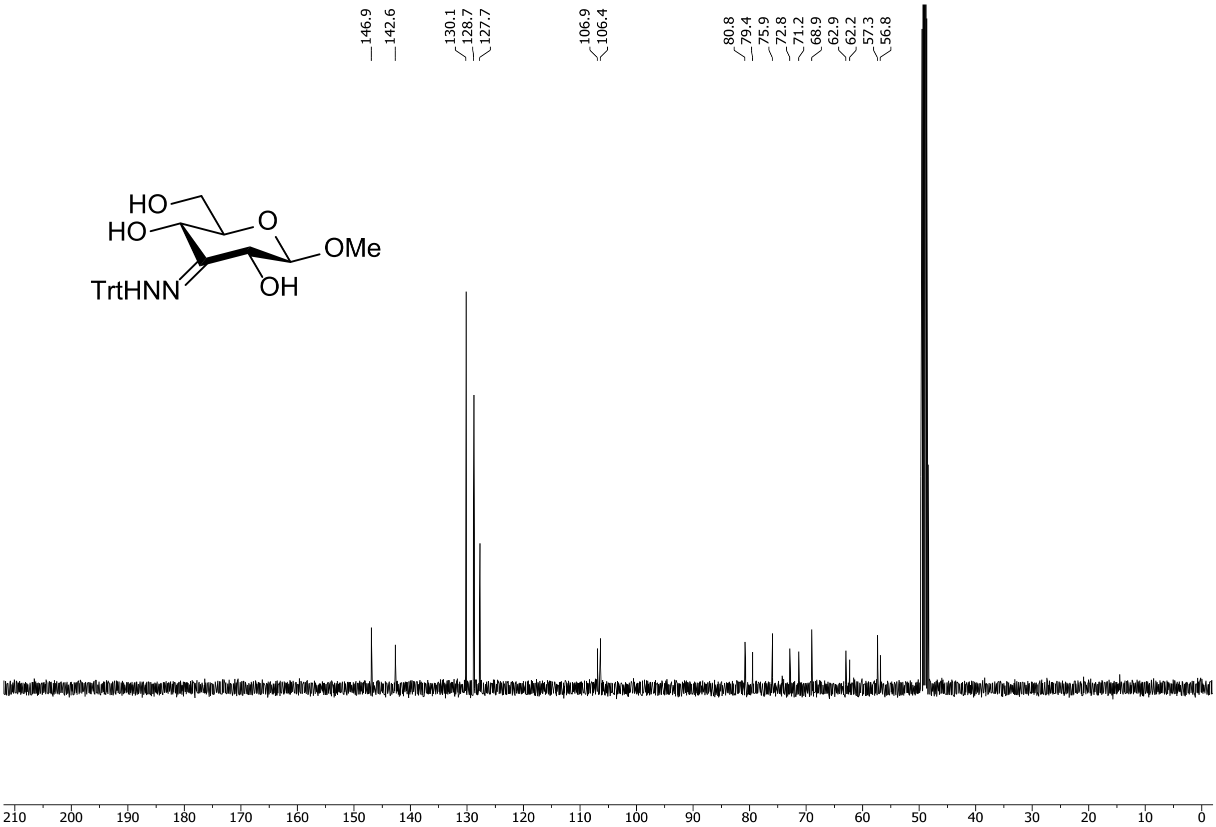

^{1}H-^{1}H COSY of compound **2g**

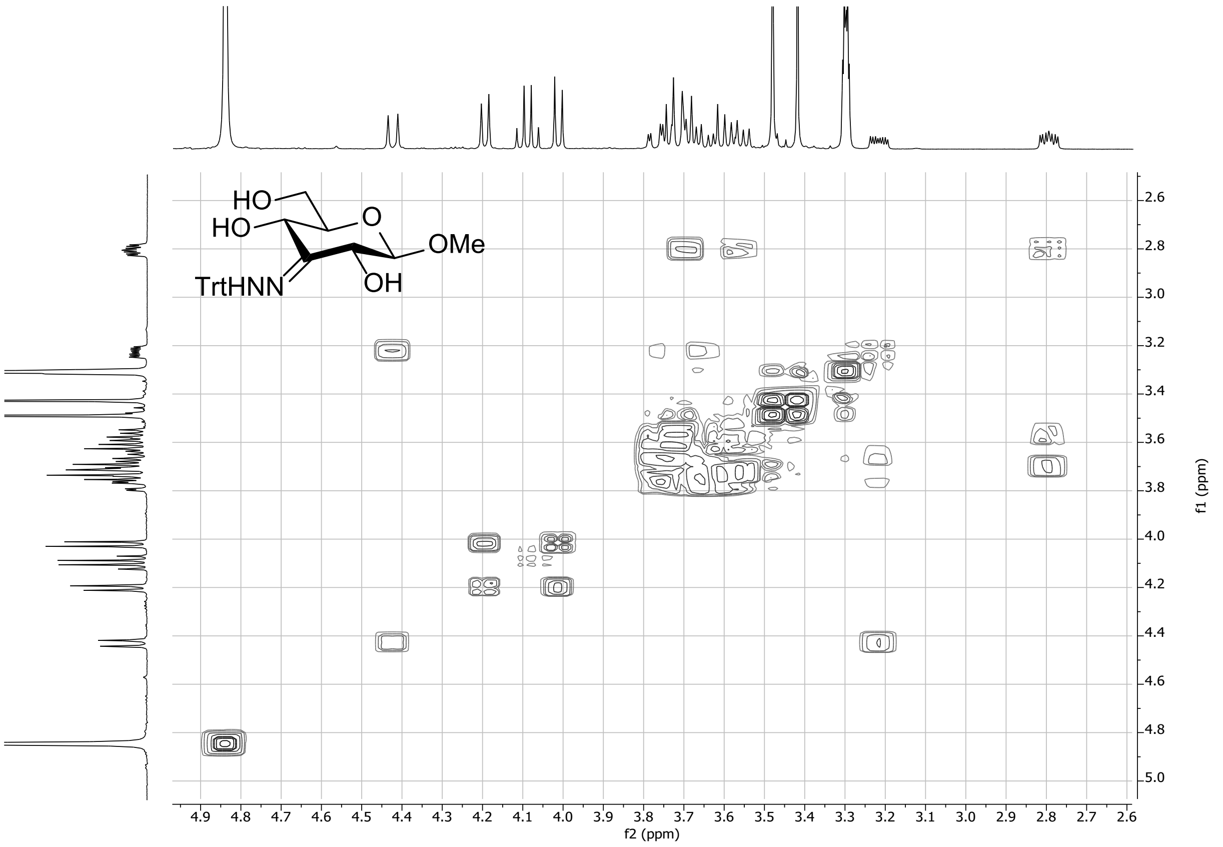

H-^{13}C HSQC of compound **2g**

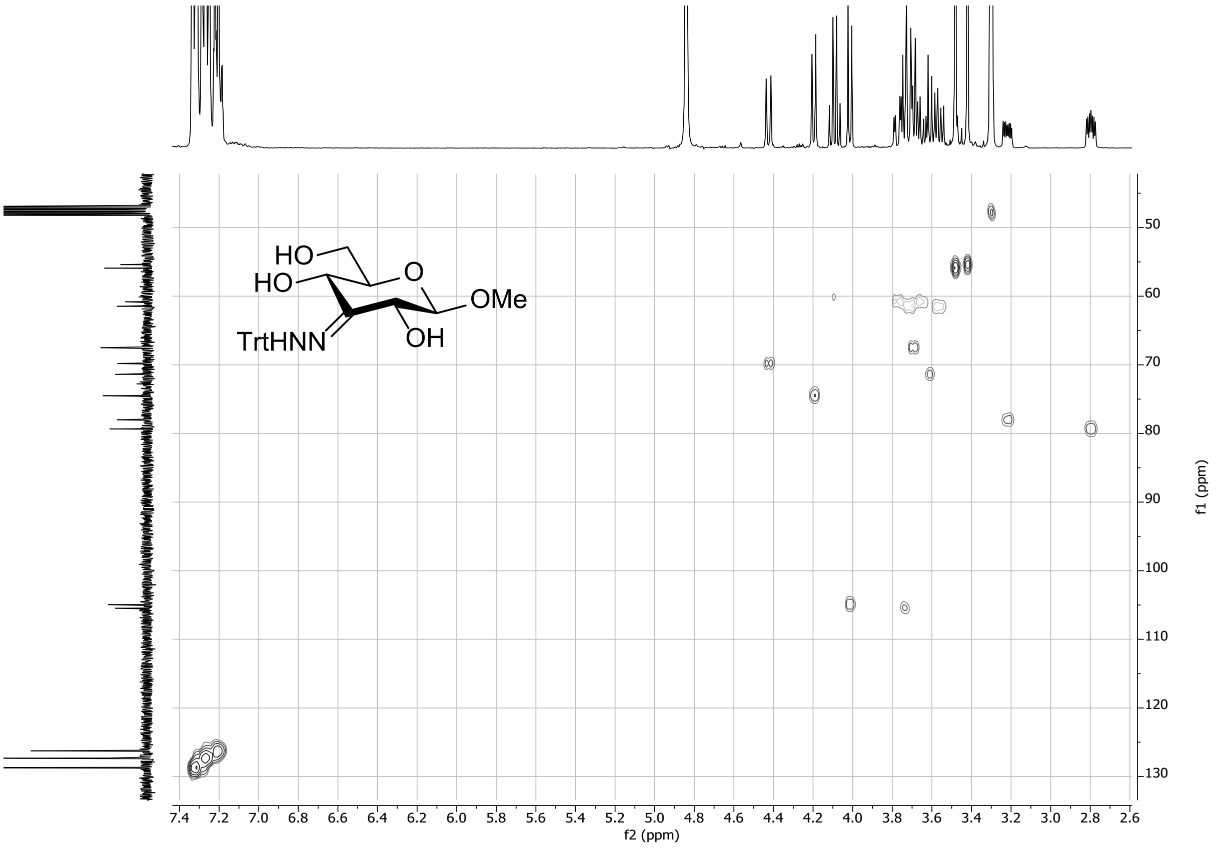

Methyl 3-chloro-3-deoxy-β-D-allo/glucopyranoside (3g)

Mixture of equatorial and axial, 3-equatorial : 3-axial ≈ 1:1.7. as is shown in ^{1}H NMR measured in CD_3CN.

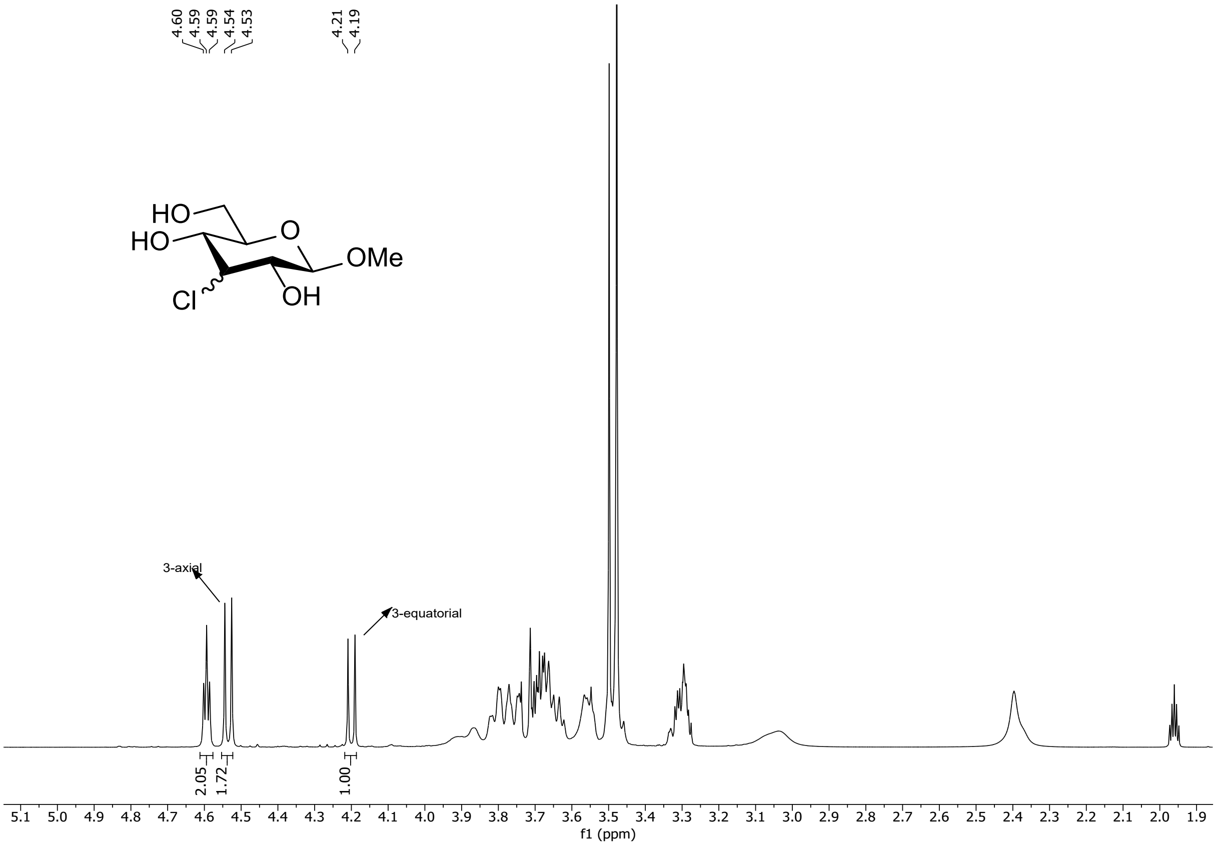

^{1}H NMR, 400 MHz, CD_3OD of compound **3g**

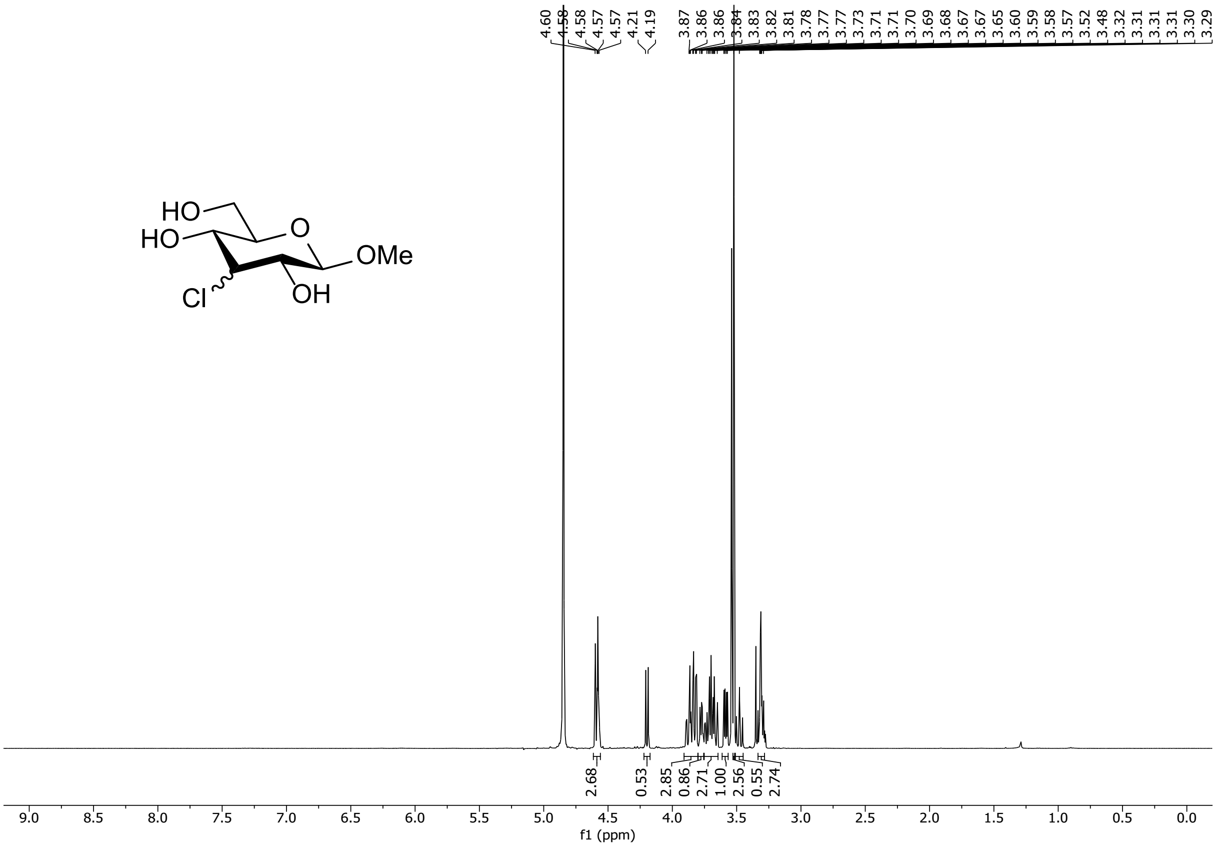

^{13}C NMR, 400 MHz, CD$_3$OD of compound **3g**

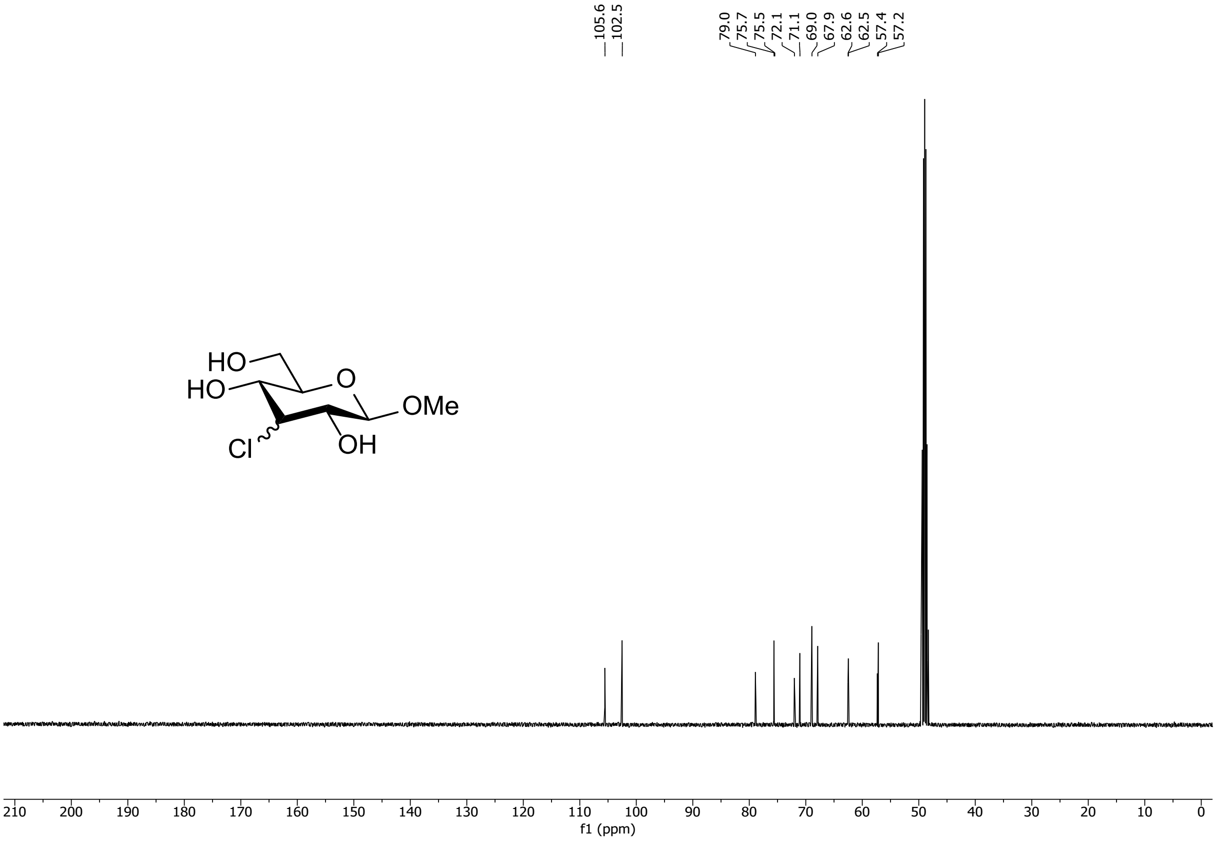

^{1}H-^{1}H COSY of compound **3g**

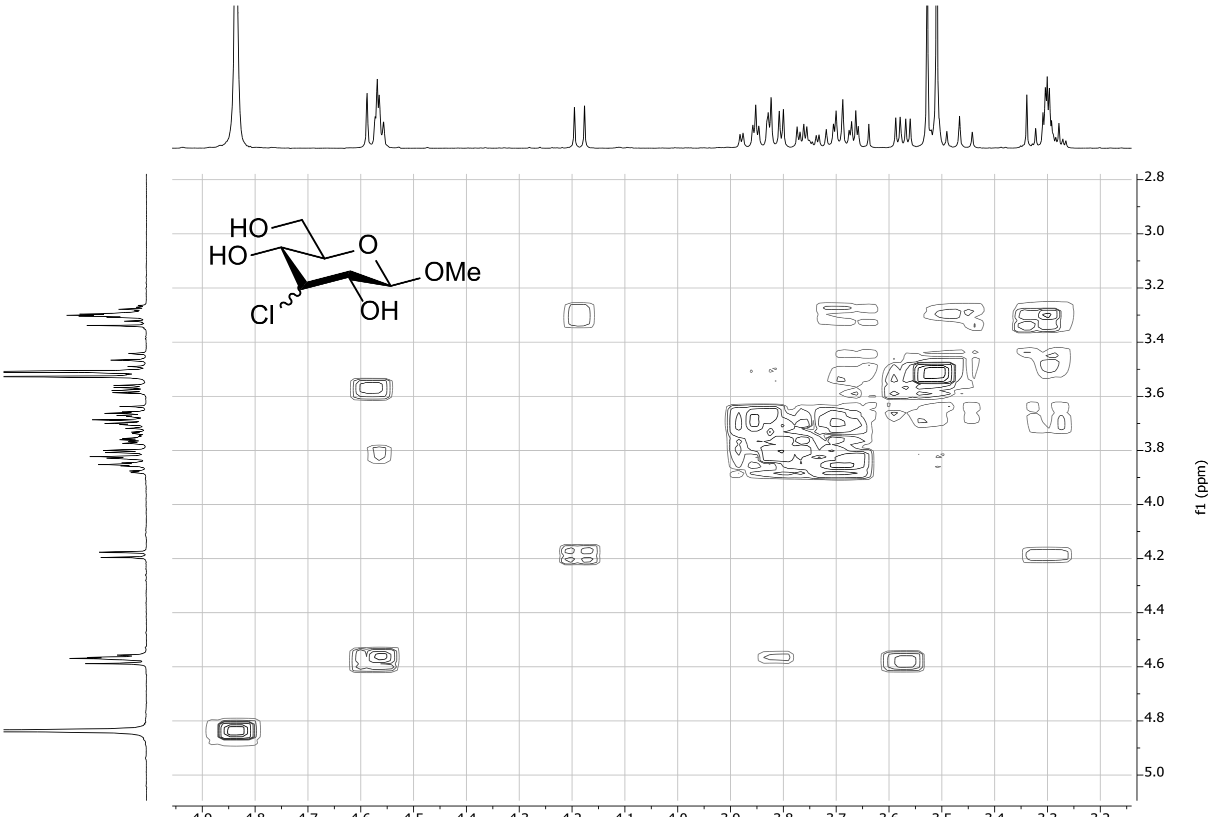

H-^{13}C HSQC of compound **3g**

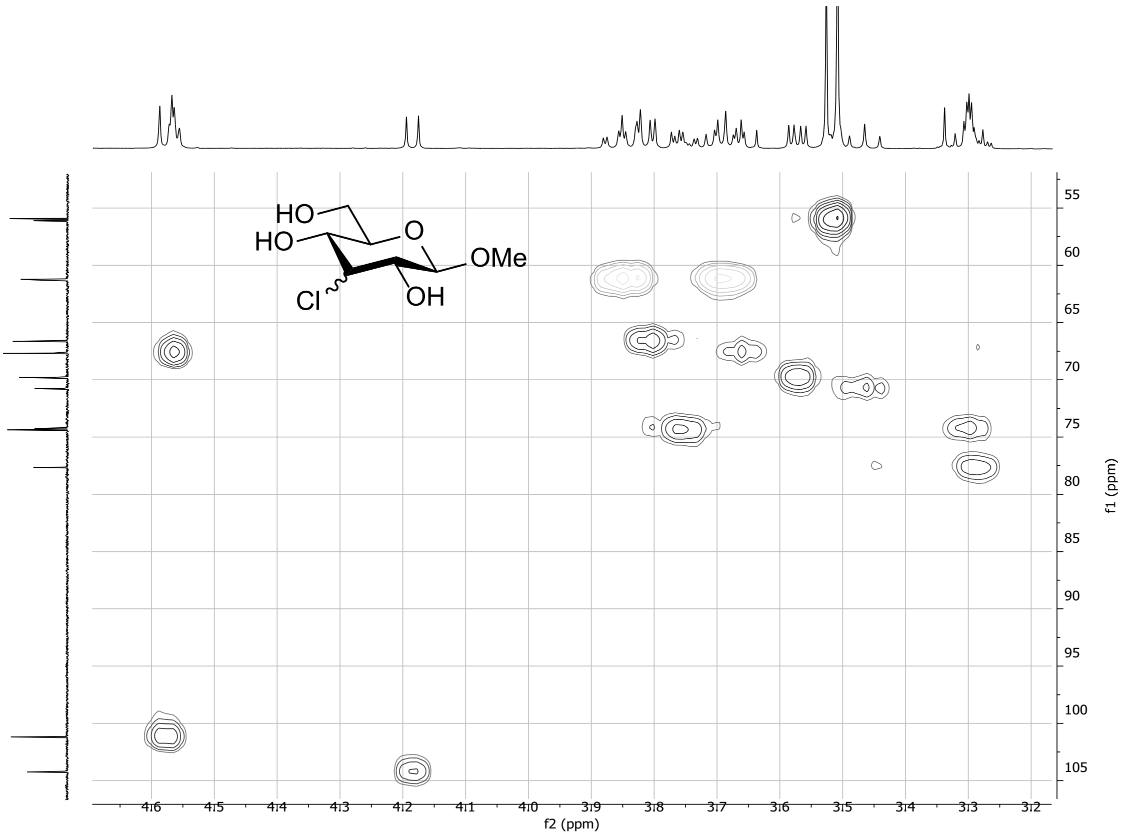

Methyl-3-(2,4-dinitrophenyl)hydrazone-β-D-glucopyranoside (6b)

^{1}H NMR, 400 MHz, CD_3CN of compound **6b**

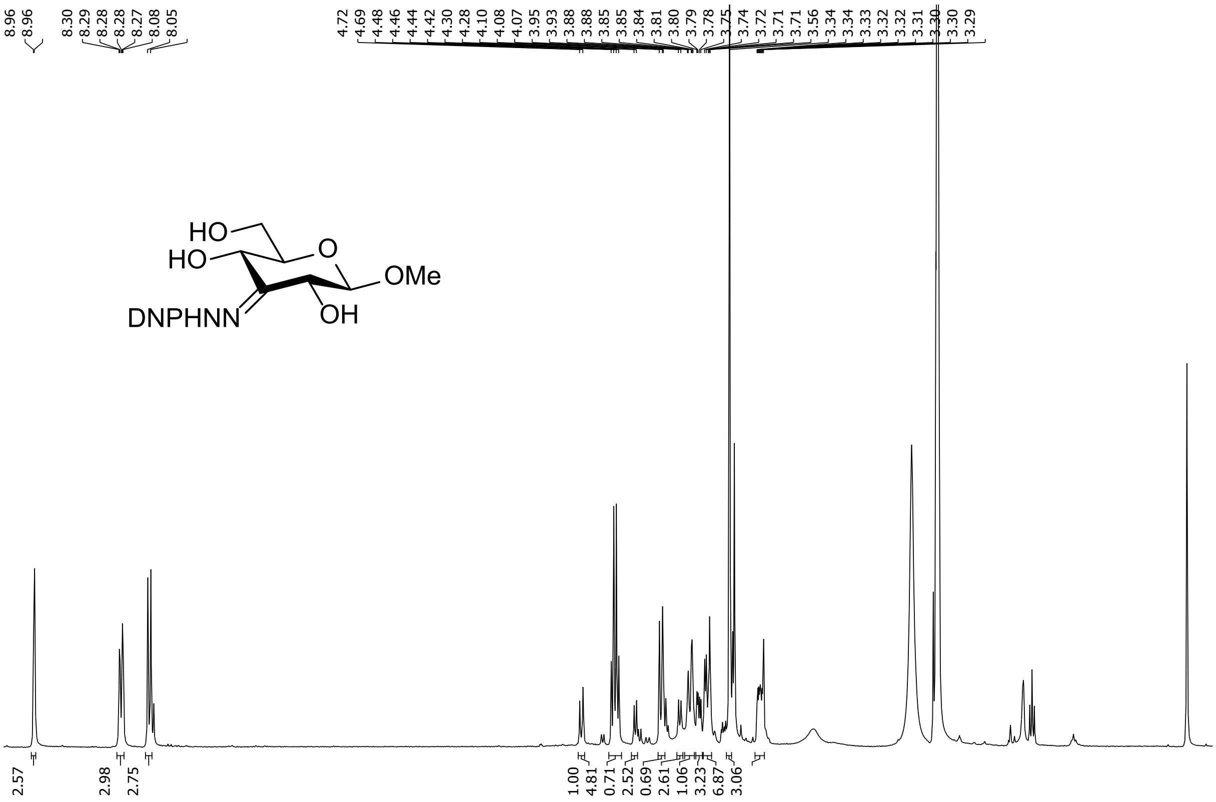

^{13}C NMR, 400 MHz, CD_3CN of compound **6b**

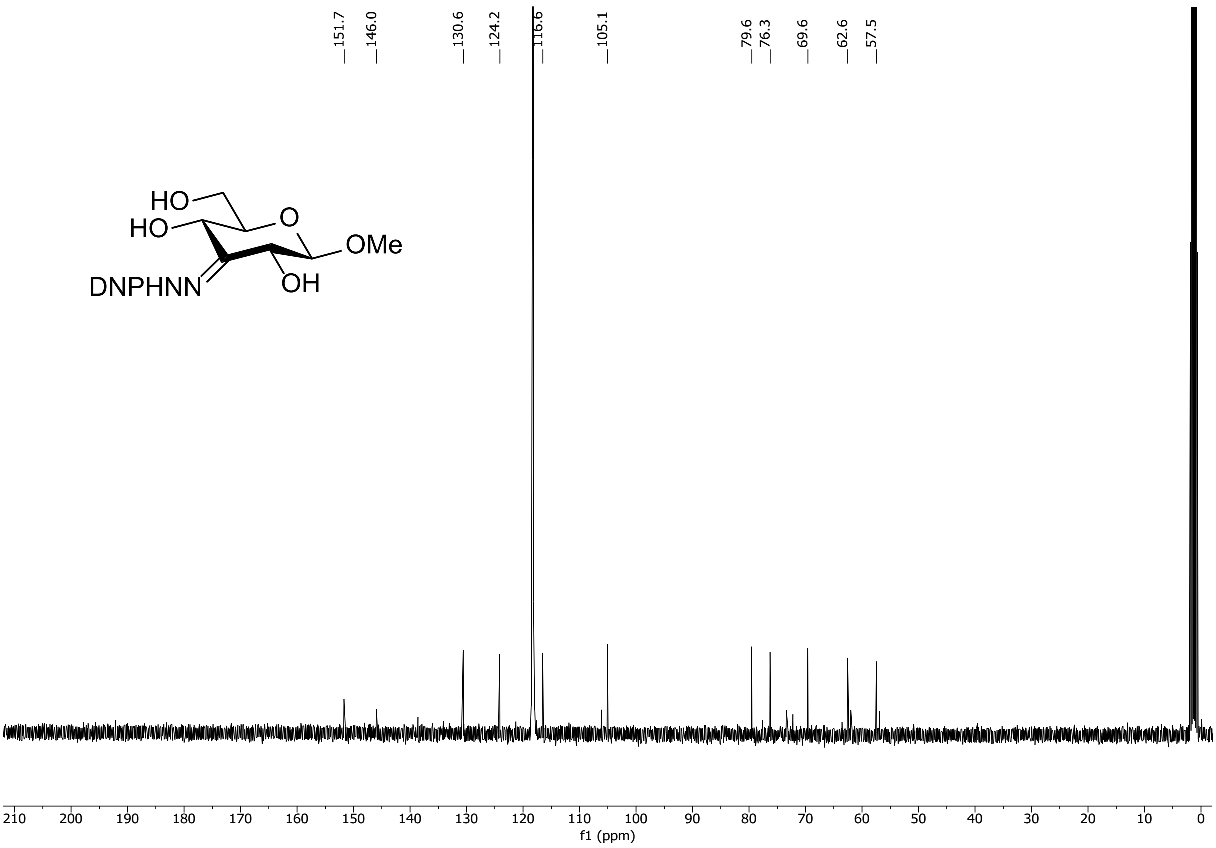

^{1}H-^{1}H COSY of compound **6b**

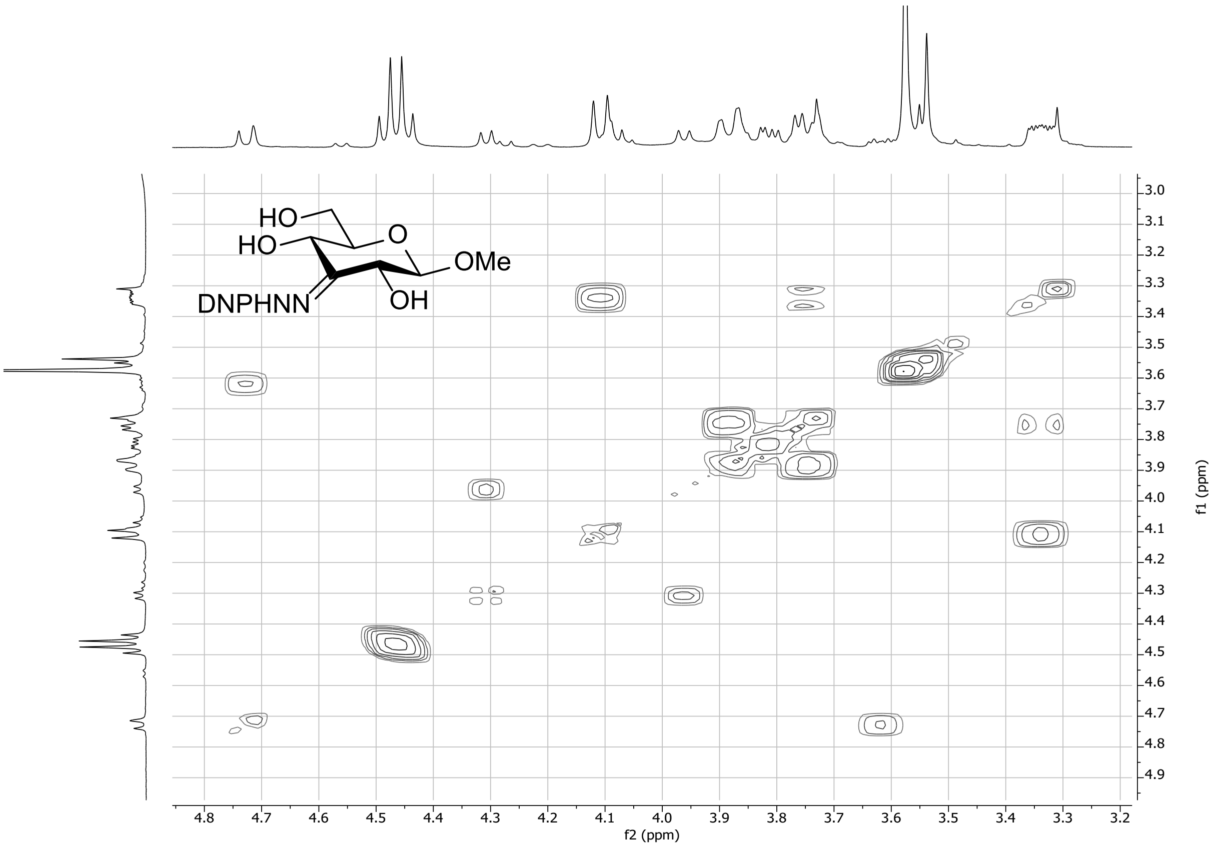

H-^{13}C HSQC of compound **6b**

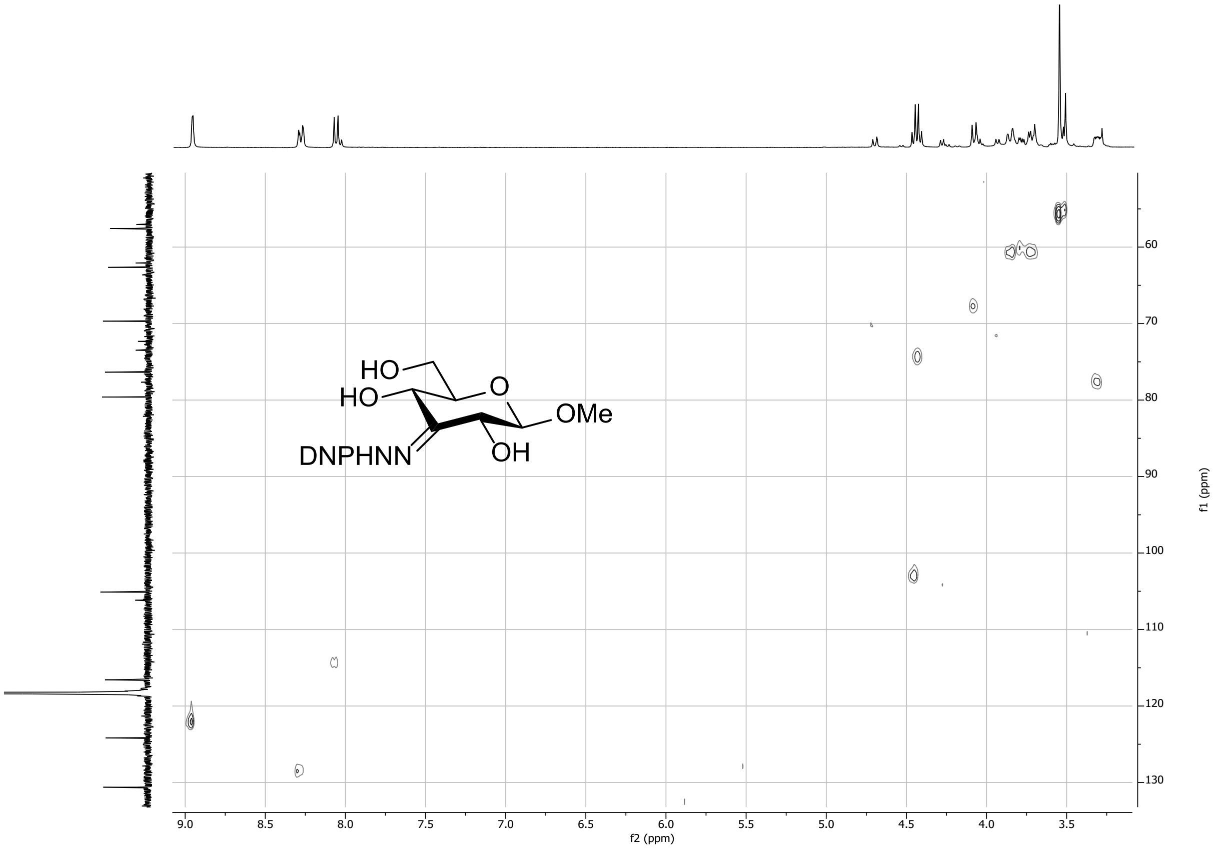

Methyl-3-chloro-3-(2,4-dinitrophenyl)diazene-β-D-glucopyranoside (7b)

^{1}H NMR, 400 MHz, CD_3OD; comparison between **6b** (below) and crude of **7b** (above).

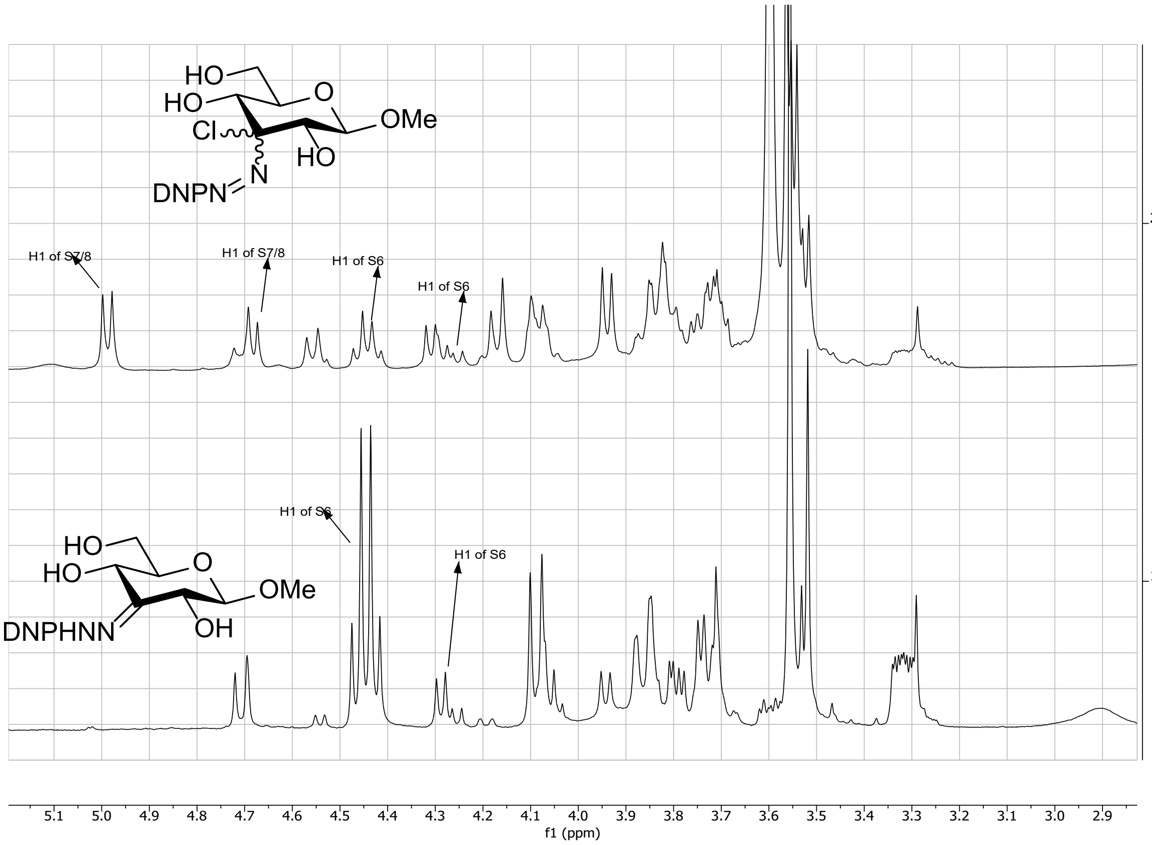

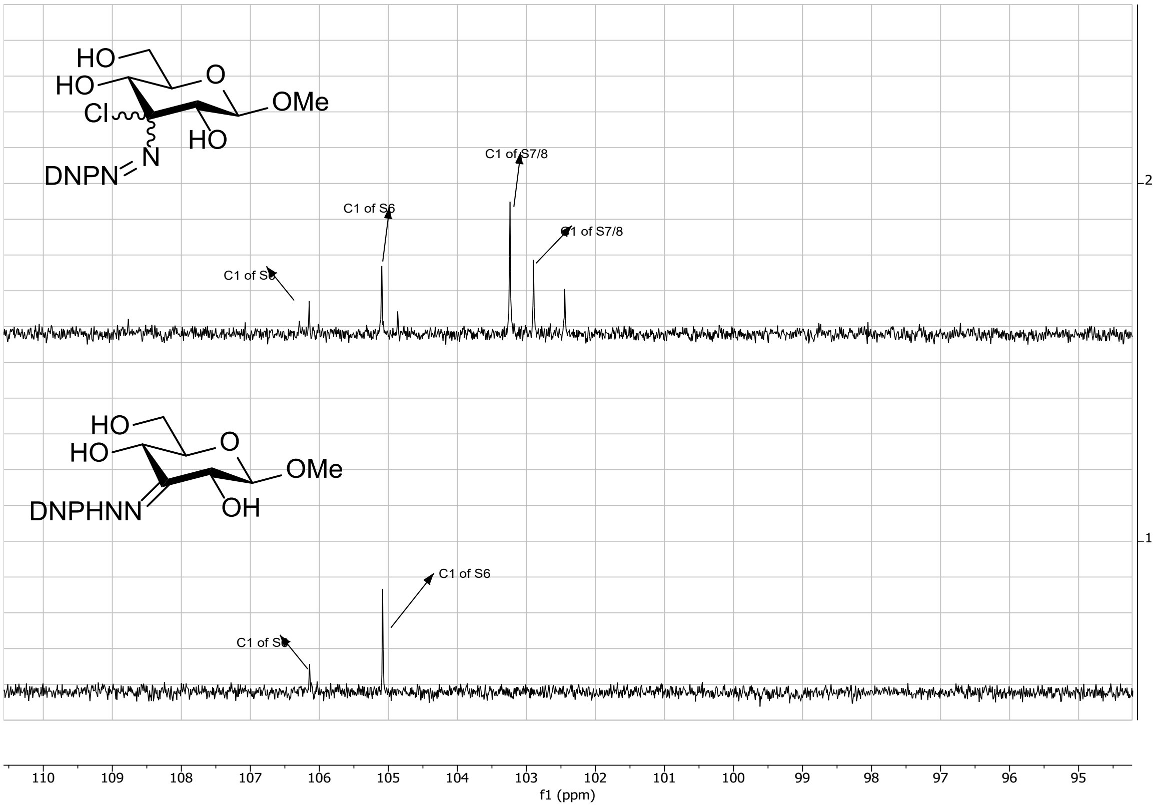

^{1}H NMR, 400 MHz, CD_3CN of compound **7b**

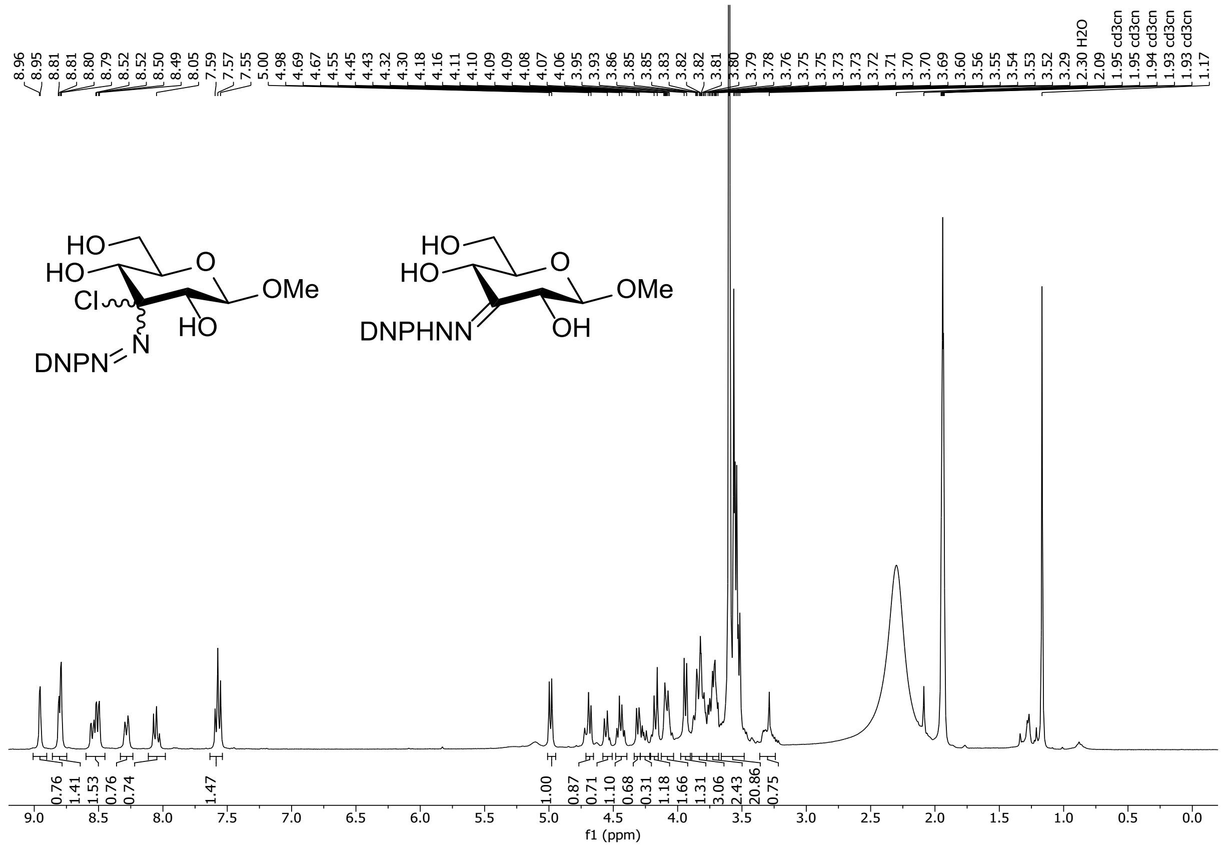

^{13}C NMR, 400 MHz, CD_3CN of compound **7b**

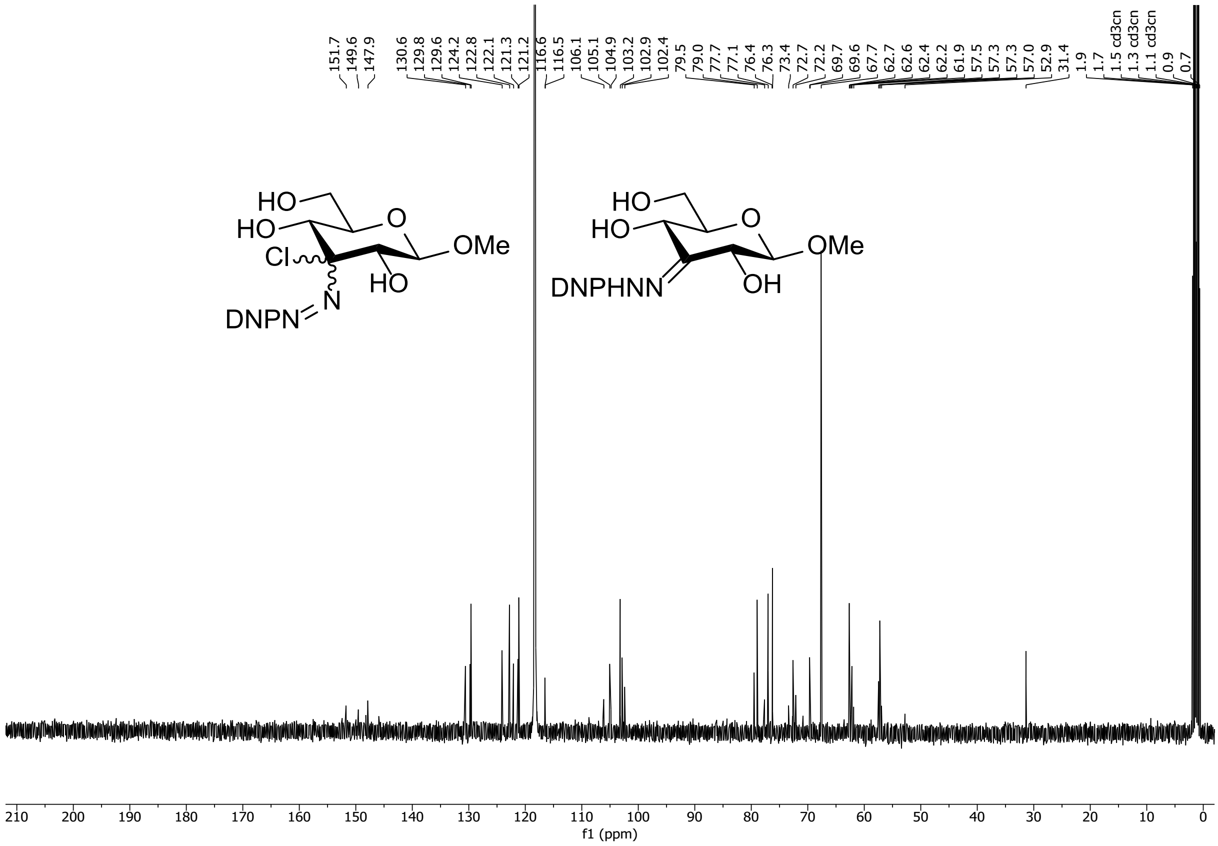

^{1}H-^{1}H COSY of compound **7b**

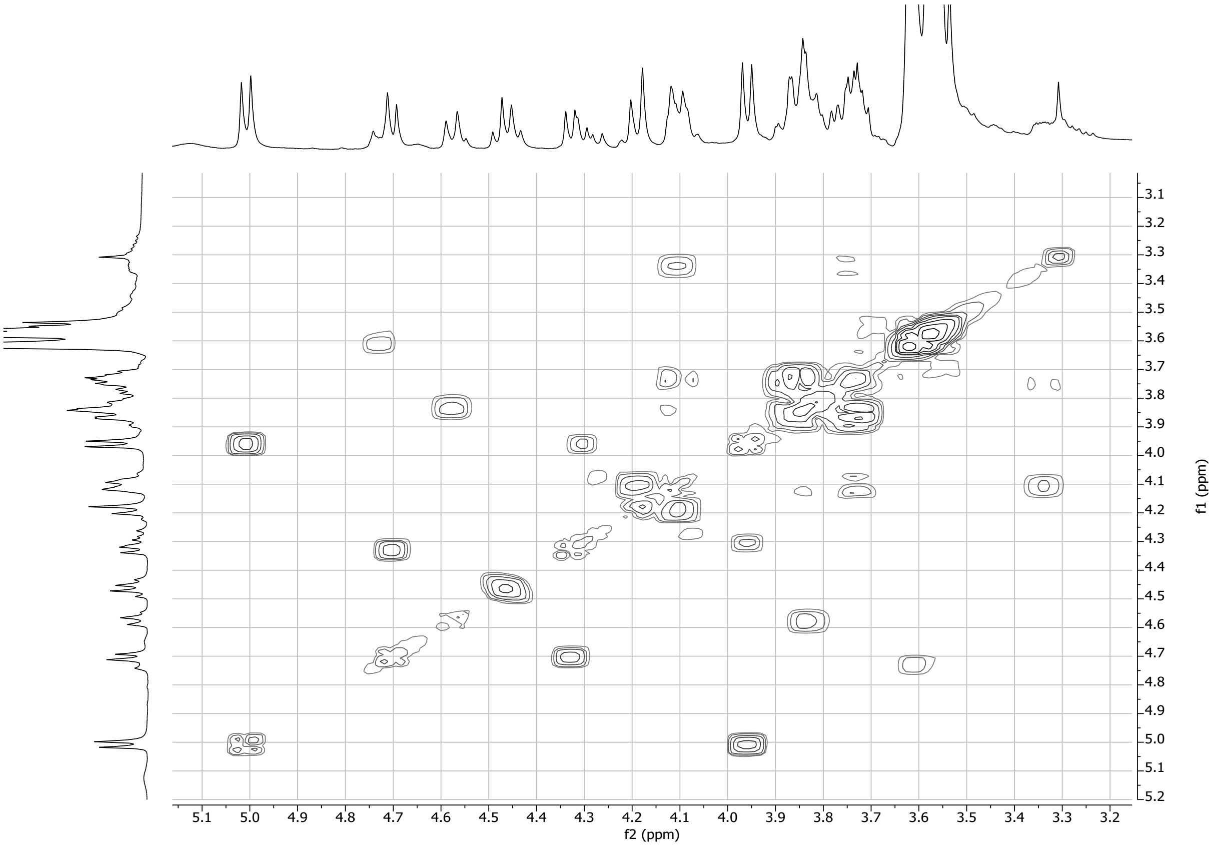

H-^{13}C HSQC of compound **7b**

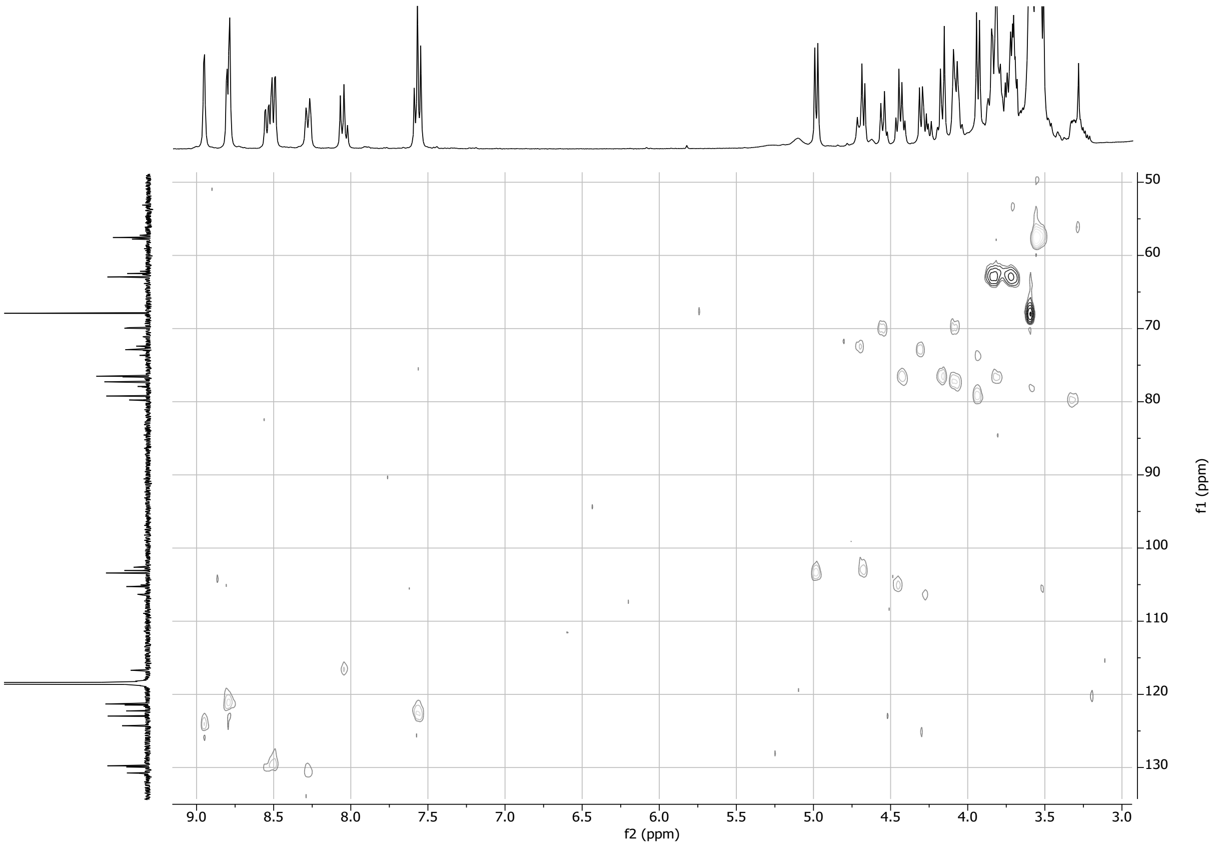

HRMS spectra

HRMS spectra of GlcNAc derivatives

Isopropyl-2-acetamido-2-deoxy-3-(trityl)hydrazone-α-D-glucopyranoside (2a)

Measured with ESI pos.

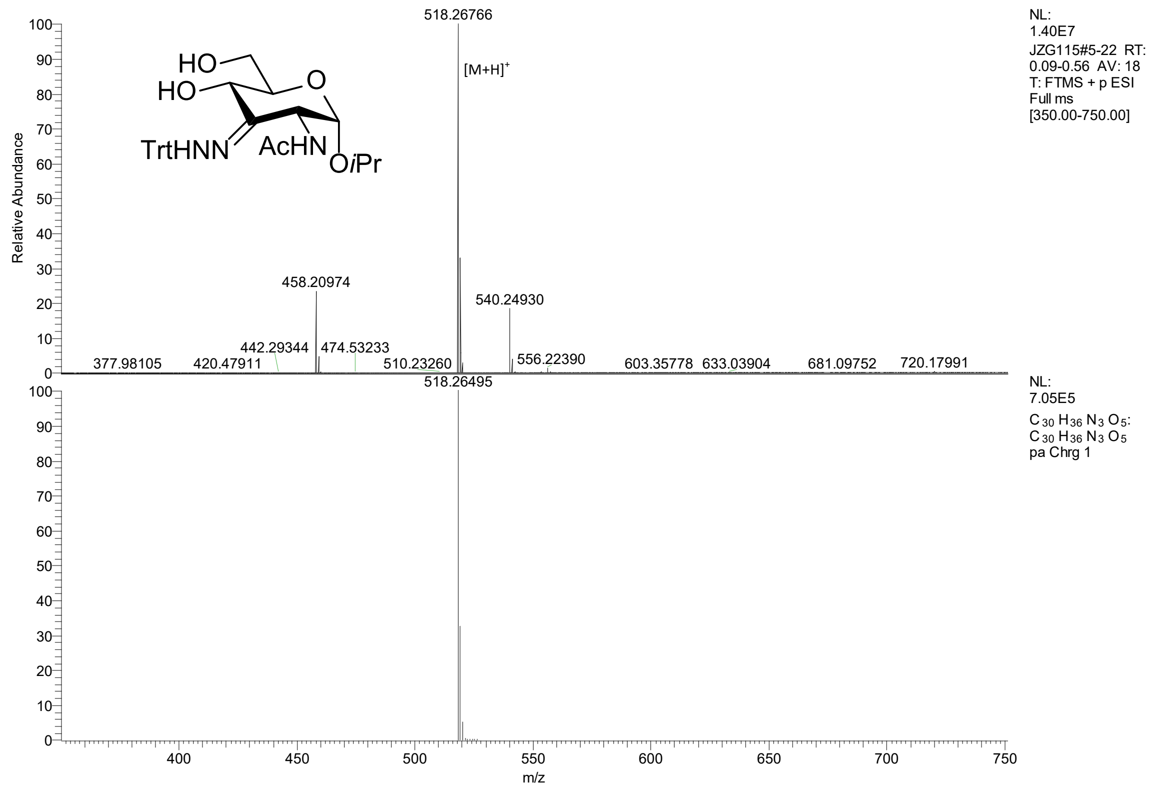

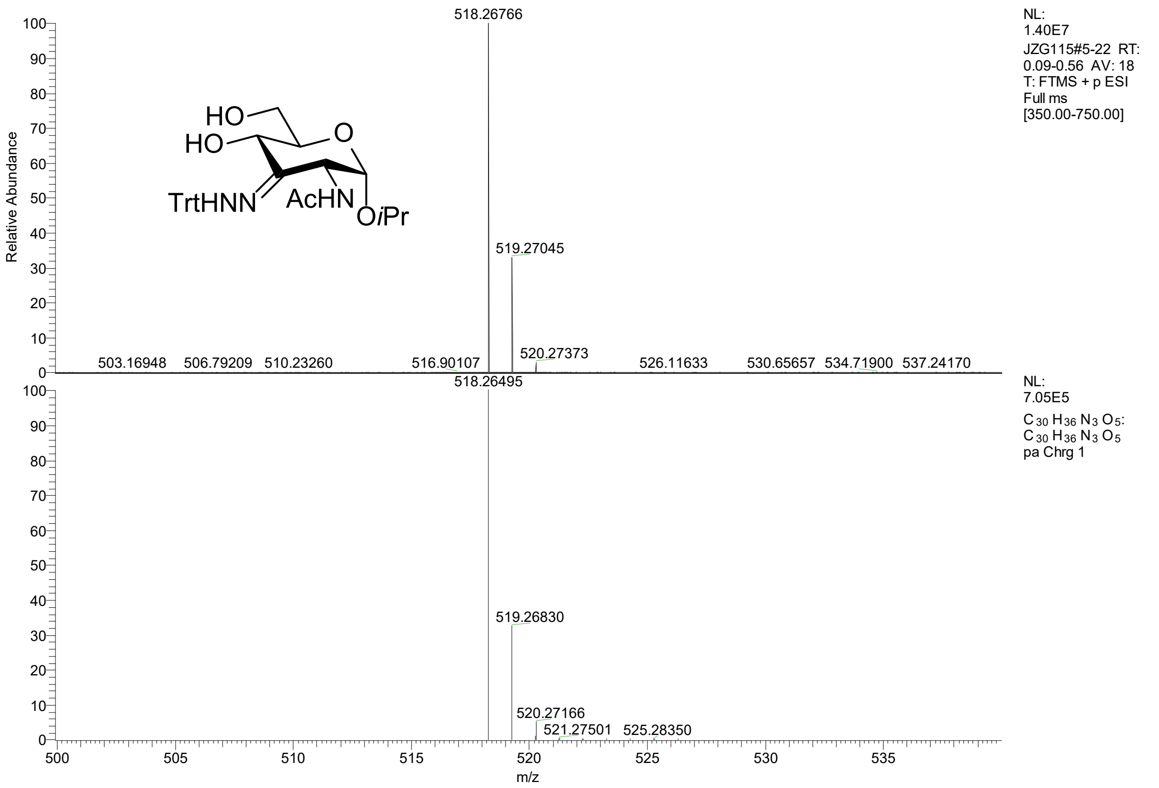

Isopropyl 2-acetmido-3-chloro-2,3-dideoxy-α-D-allo/glucopyranoside (3a)

Measured with ESI pos.

Equatorial:

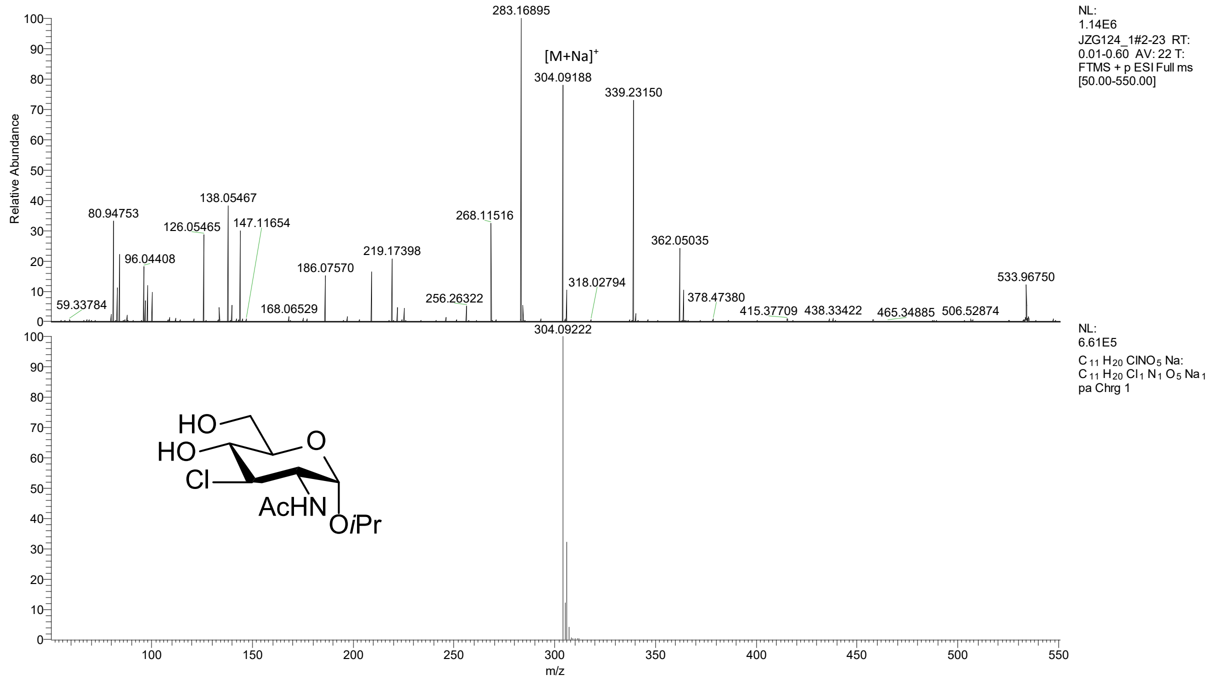

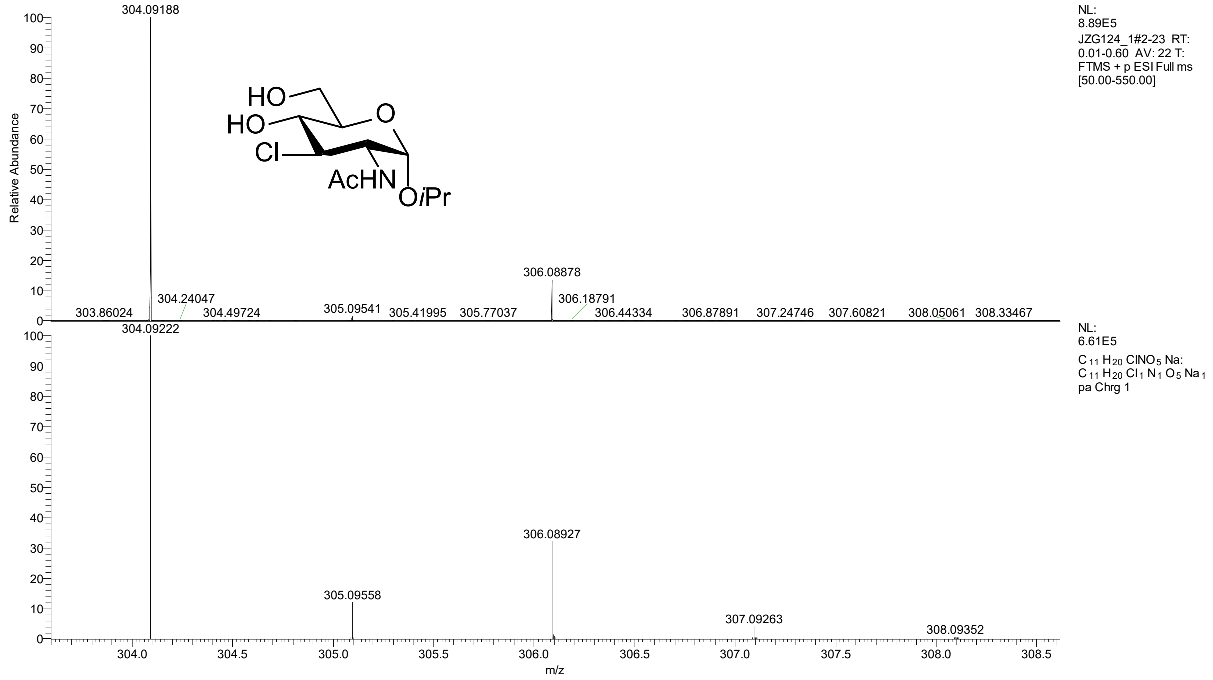

Axial:

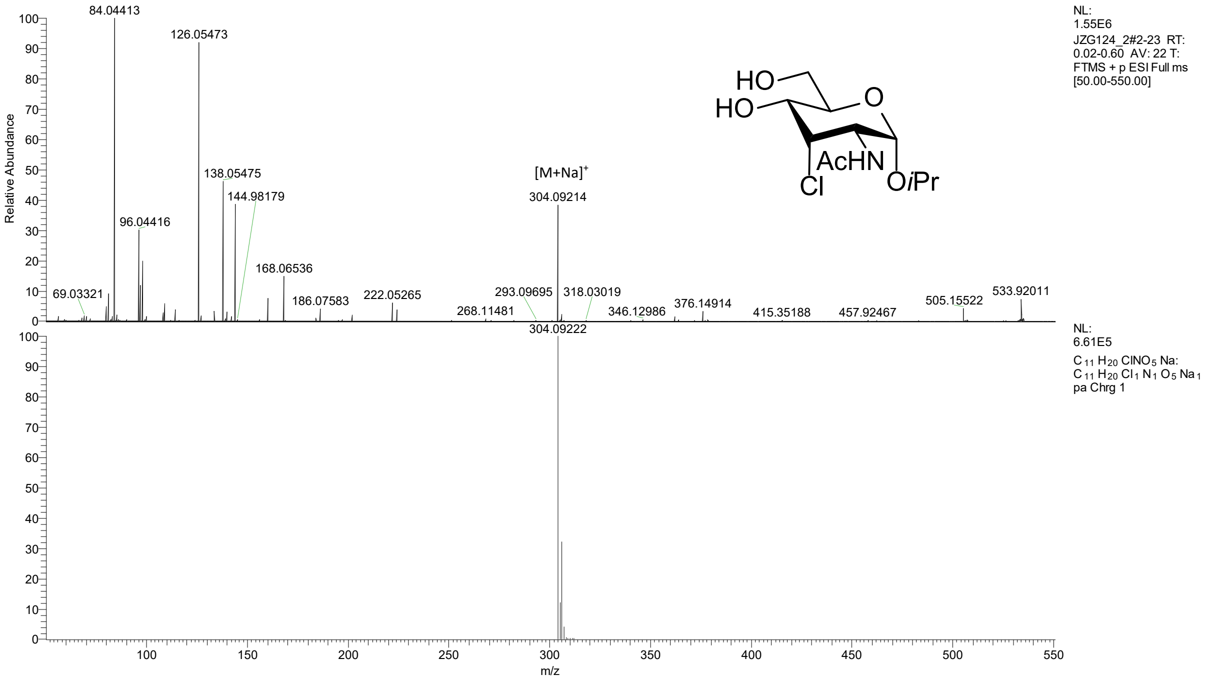

NL: 1.55E6
JZG124_2#2-23 RT: 0.02-0.60 AV: 22 T: FTMS + p ESI Full ms [50.00-550.00]
NL: 6.61E5
C 11 H 20 ClNO 5 Na: C 11 H 20 Cl 1 N 1 O 5 Na 1 pa Chrg 1
Relative Abundance
m/z
[M+Na]+
84.04413
126.05473
138.05475
144.98179
96.04416
168.06536
69.03321
186.07583
222.05265
268.11481
293.09695
304.09214
318.03019
346.12986
376.14914
415.35188
457.92467
505.15522
533.92011
304.09222
HO
HO
O
AcHN
Cl
OiPr

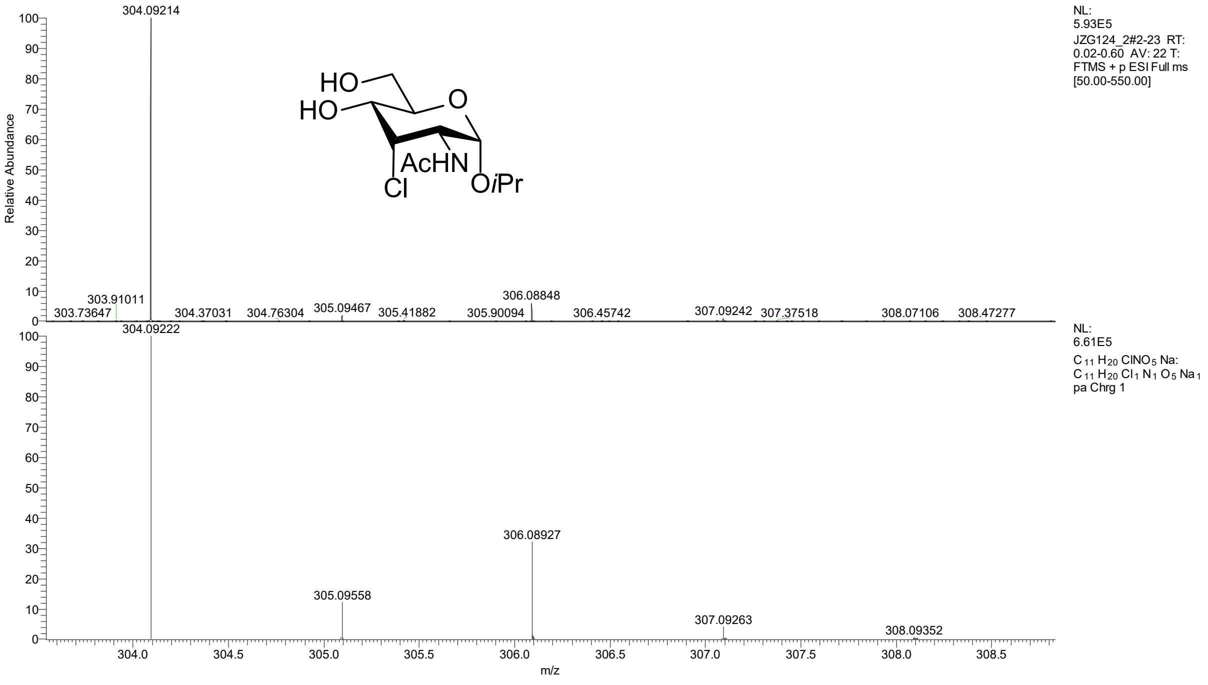

NL: 5.93E5
JZG124_2#2-23 RT: 0.02-0.60 AV: 22 T: FTMS + p ESI Full ms [50.00-550.00]
NL: 6.61E5
C 11 H 20 ClNO 5 Na: C 11 H 20 Cl 1 N 1 O 5 Na 1 pa Chrg 1
Relative Abundance
m/z
304.09214
303.73647
303.91011
304.37031
304.76304
305.09467
305.41882
305.90094
306.08848
306.45742
307.09242
307.37518
308.07106
308.47277
304.09222
305.09558
306.08927
307.09263
308.09352
HO
HO
O
AcHN
Cl
OiPr

HRMS spectra of α-Glc derivatives

Methyl-3-(trityl)hydrazone-α-D-glucopyranoside (2b)

Measured with ESI pos.

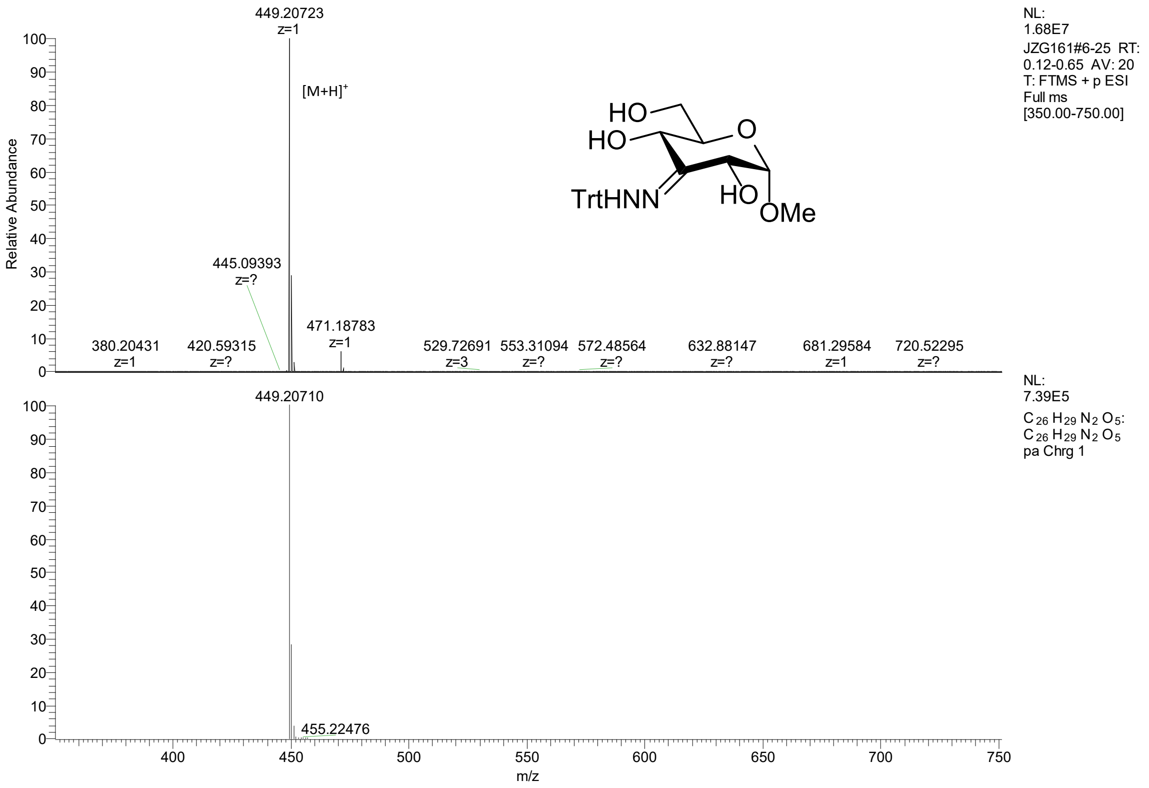

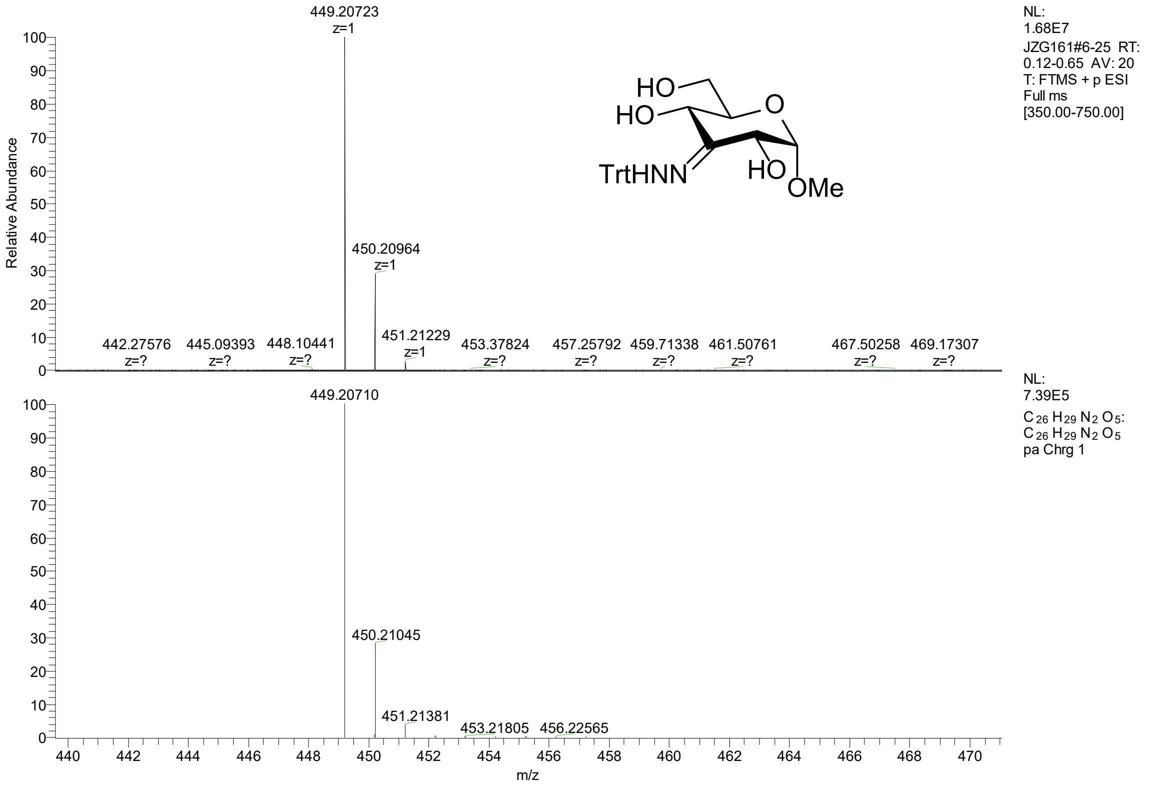

Methyl 3-chloro-3-deoxy-α-D-allo/glucopyranoside (3b)

Measured with ESI neg. in the presence of guanidinium chloride.

Equatorial:

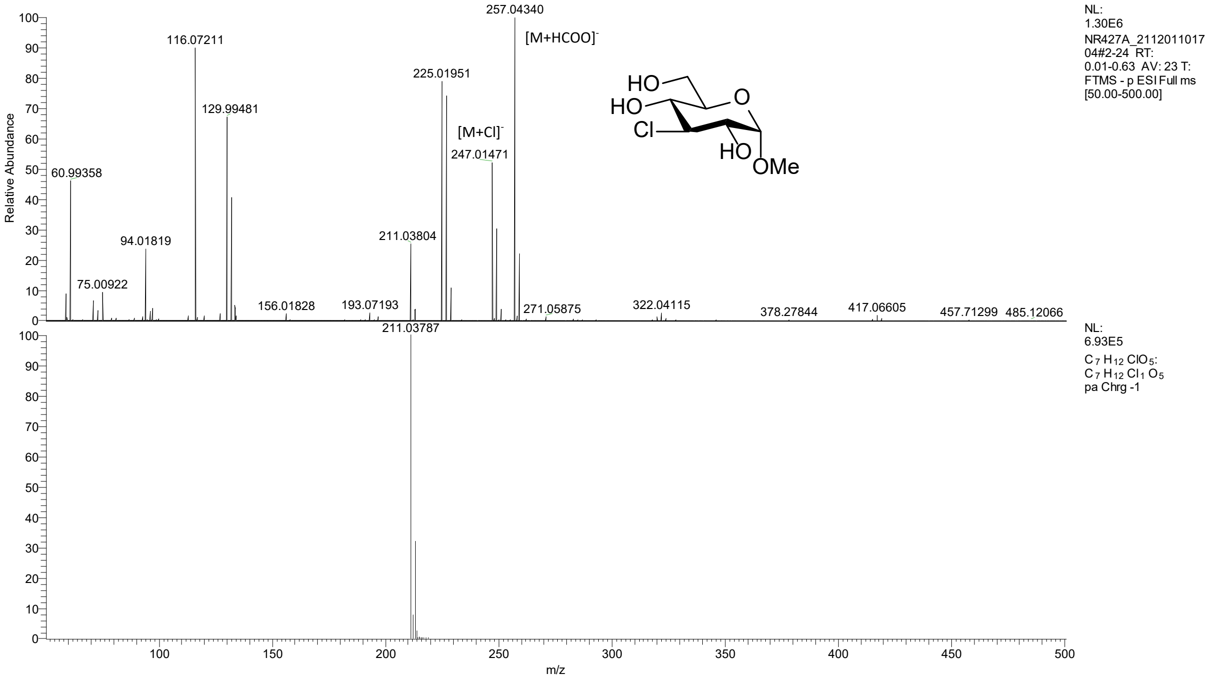

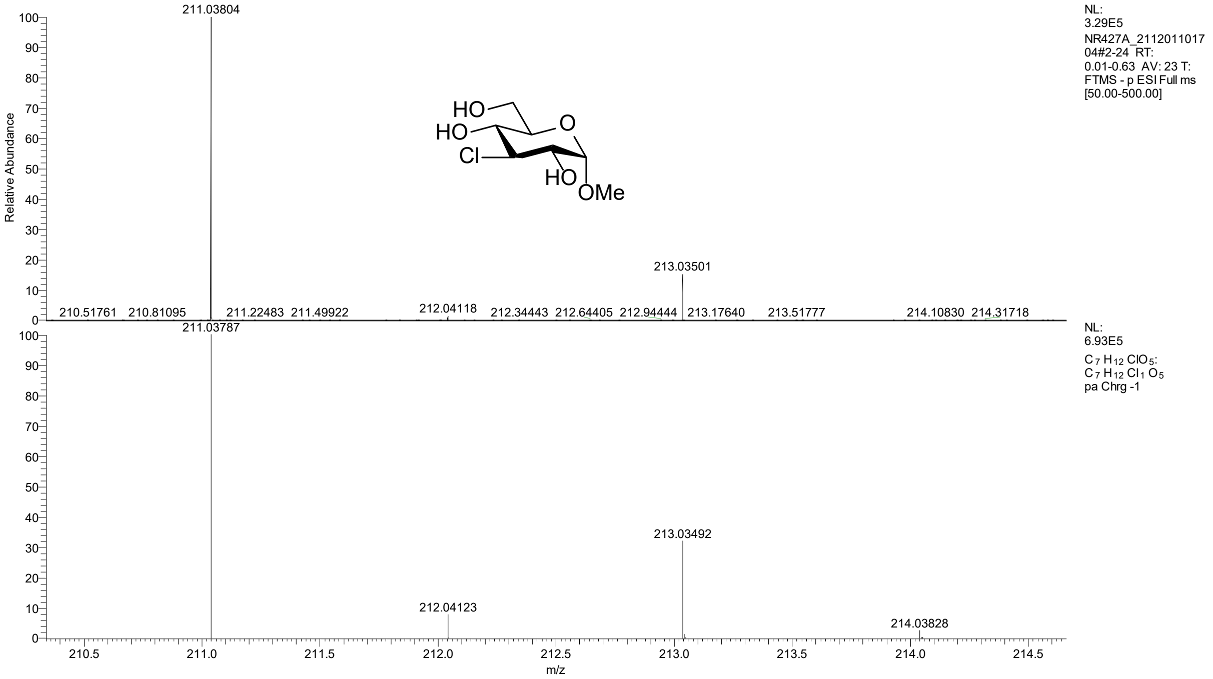

Axial:

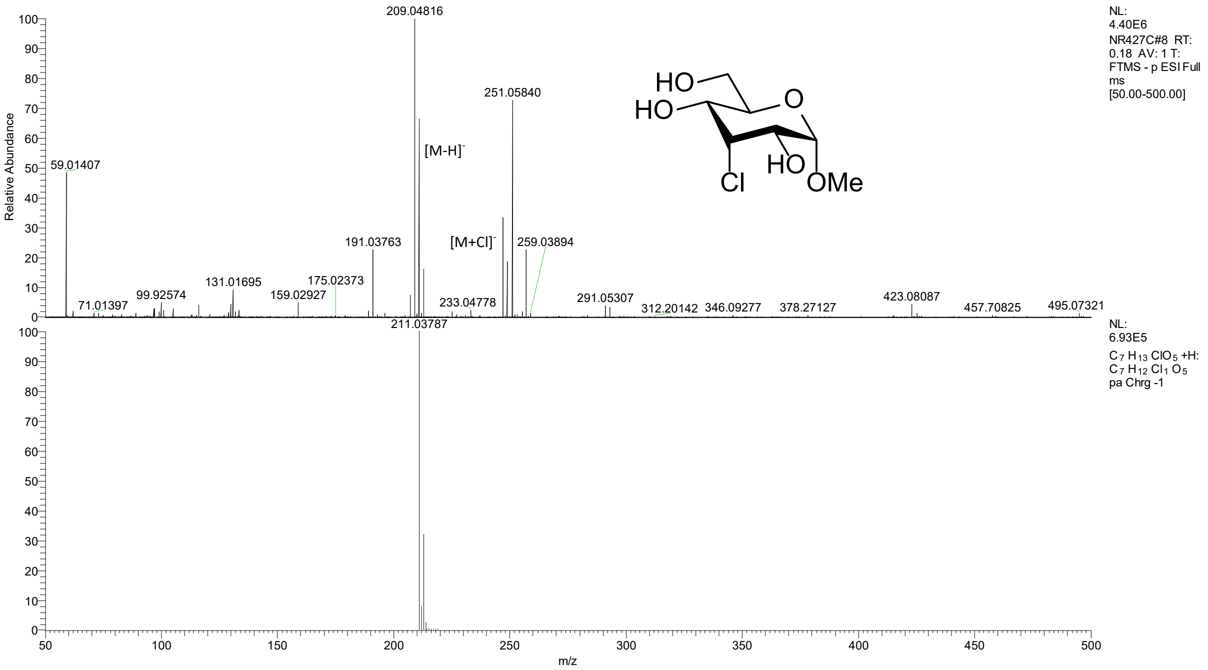
209.04816
251.05840
59.01407
71.01397
99.92574
131.01695
159.02927
175.02373
191.03763
233.04778
259.03894
291.05307
312.20142
346.09277
378.27127
423.08087
457.70825
495.07321
[M-H]-
[M+Cl]-
HO
HO
O
HO
Cl
OMe
Relative Abundance
NL:
4.40E6
NR427C#8 RT:
0.18 AV: 1 T:
FTMS - p ESI Full
ms
[50.00-500.00]
211.03787
NL:
6.93E5
C7 H13 ClO5 +H:
C7 H12 Cl1 O5
pa Chrg -1
m/z

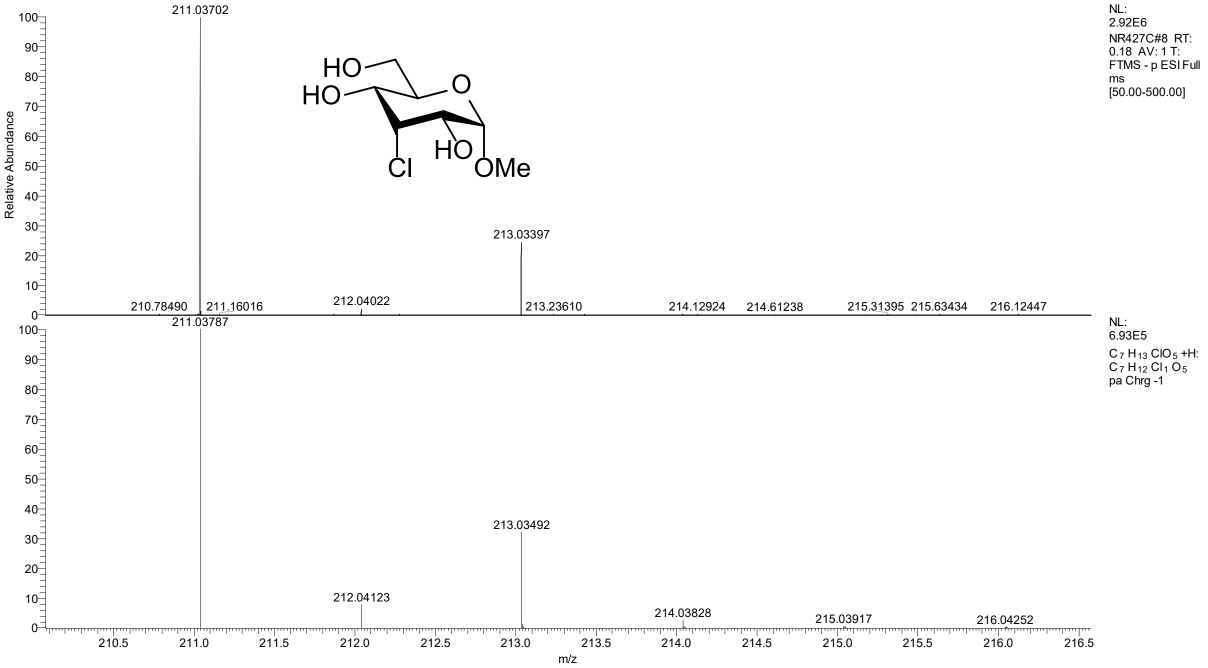
211.03702
210.78490
211.16016
212.04022
213.03397
213.23610
214.12924
214.61238
215.31395
215.63434
216.12447
HO
HO
O
HO
Cl
OMe
Relative Abundance
NL:
2.92E6
NR427C#8 RT:
0.18 AV: 1 T:
FTMS - p ESI Full
ms
[50.00-500.00]
211.03787
212.04123
213.03492
214.03828
215.03917
216.04252
NL:
6.93E5
C7 H13 ClO5 +H:
C7 H12 Cl1 O5
pa Chrg -1
m/z

Methyl 3-S-ethyl-3-deoxy-α-D-allo/glucopyranoside (4)

Measured with ESI neg. in the presence of guanidinium chloride.

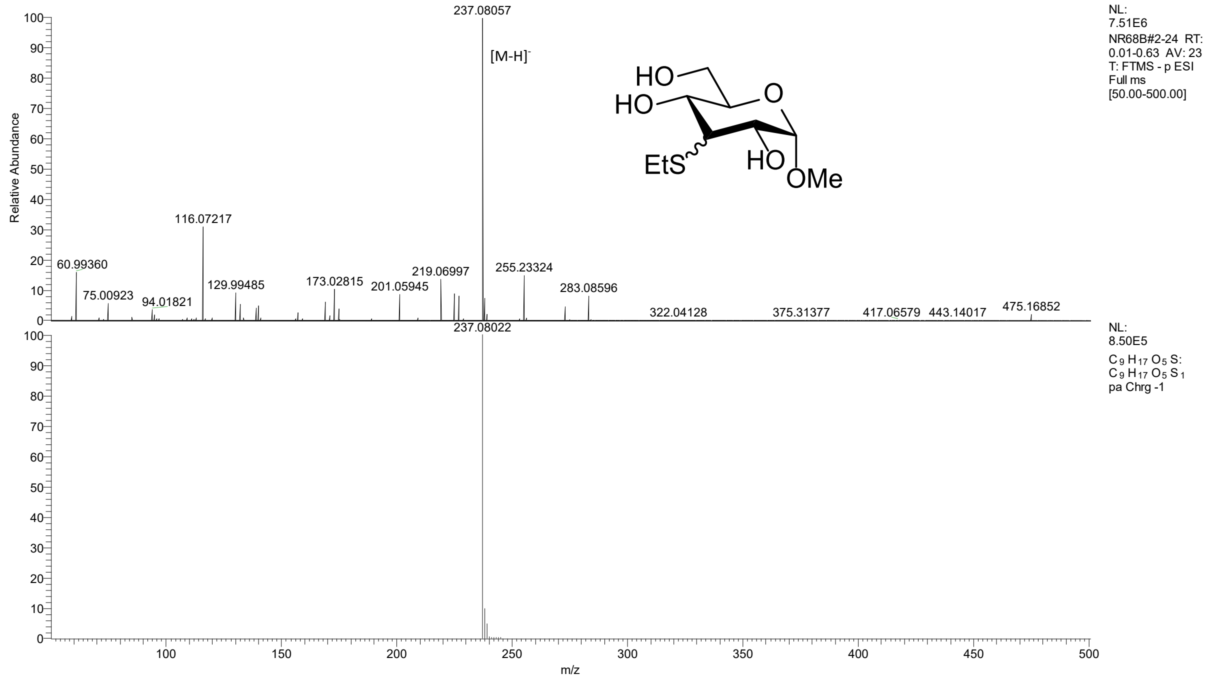

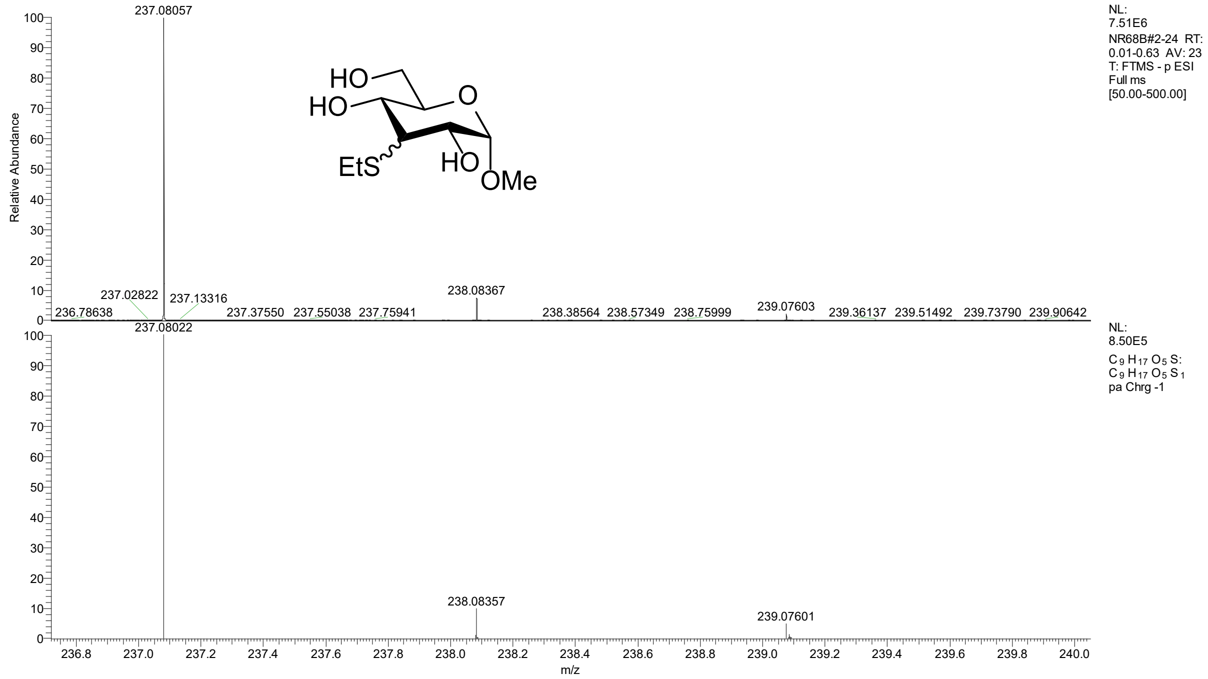

Methyl 3-O-acetyl-α-D-glucopyranoside (S1)

Measured with ESI neg. in the presence of guanidinium chloride.

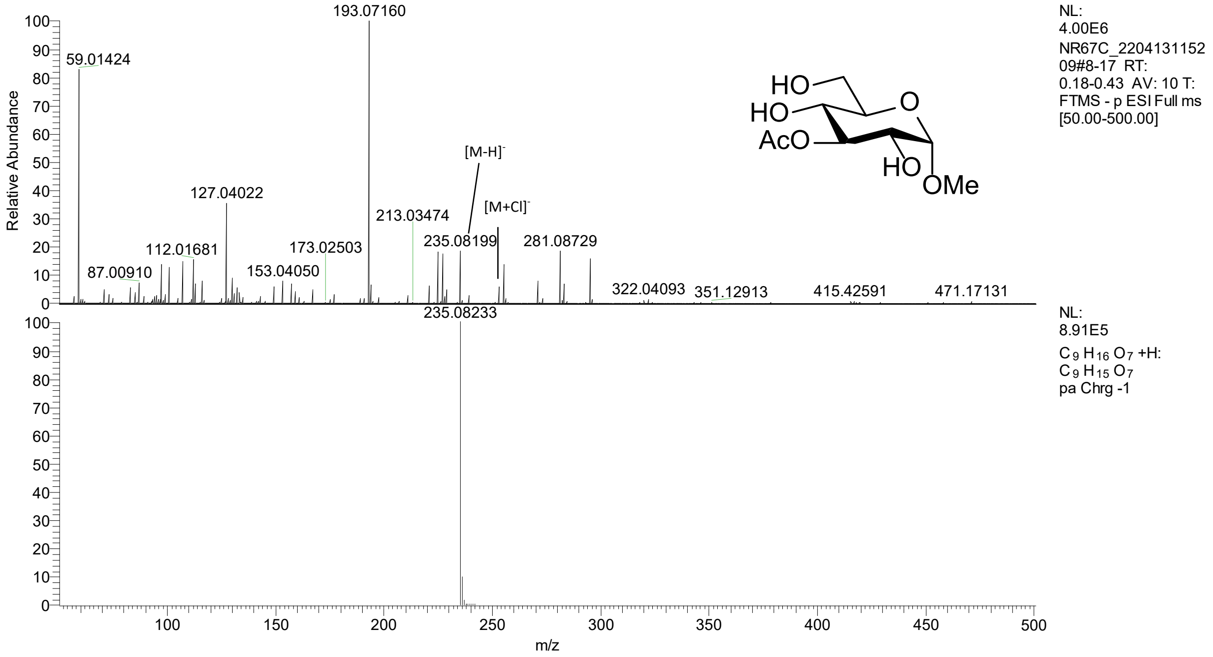

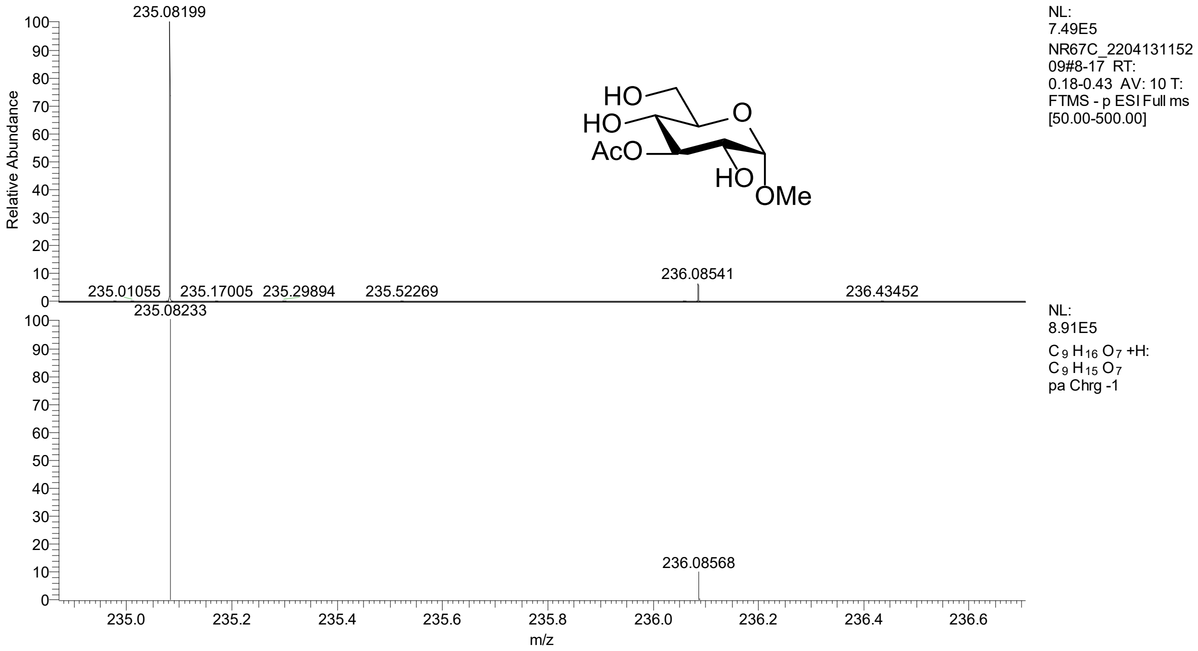

Methyl 3-S-isopropyl-3-deoxy-α-D-allo/glucopyranoside (S2)

Measured with ESI pos.

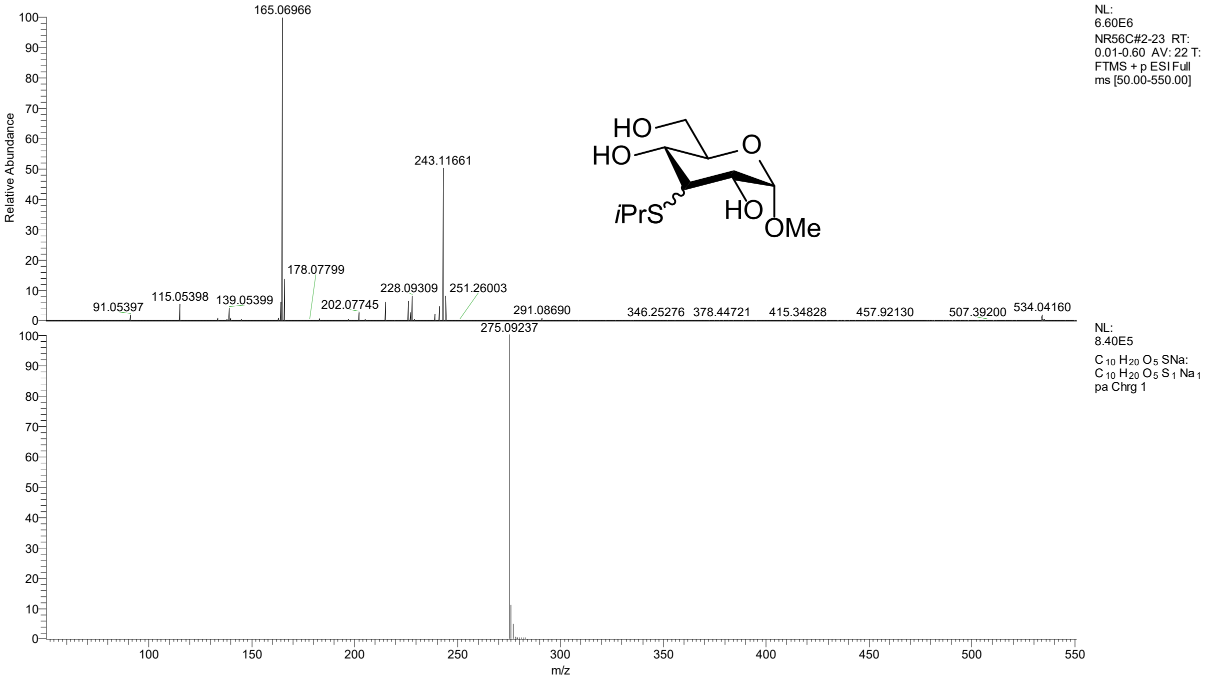

*243 and 165 come from remaining trityl material. The trityl cation causes ion suppression.

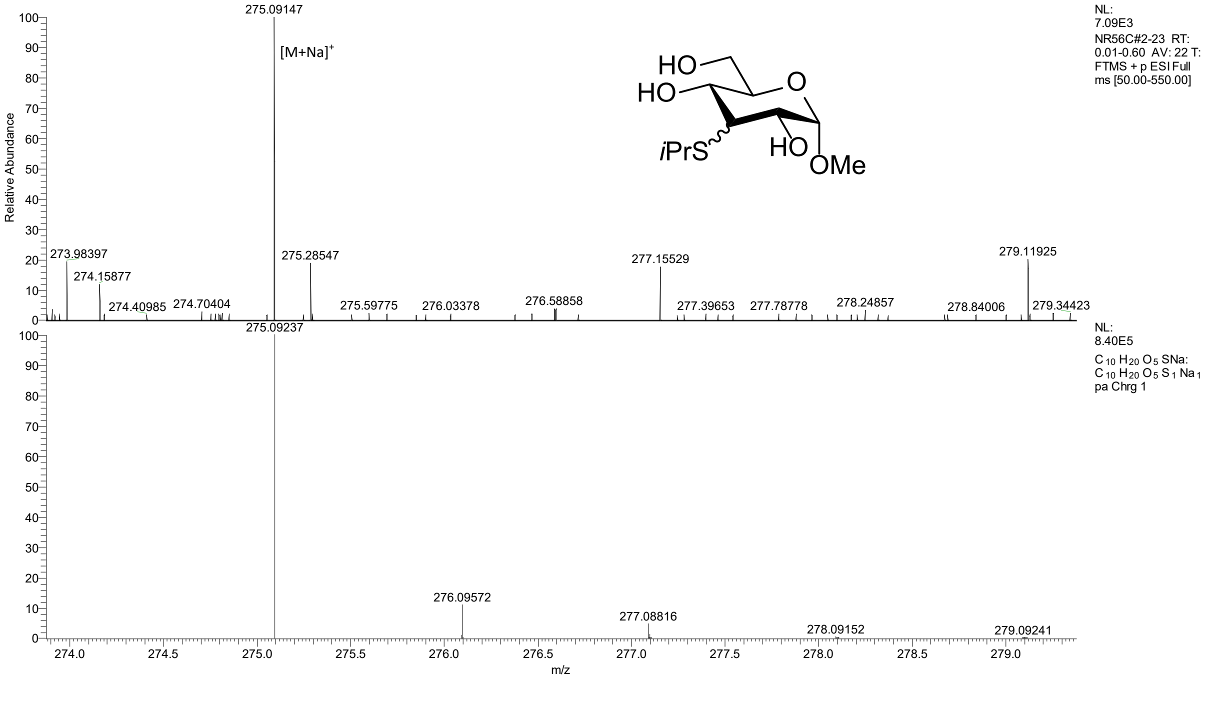

Methyl 3-S-acetyl-3-deoxy-α-D-allo/glucopyranoside (S3)

Measured with ESI neg. in the presence of guanidinium chloride.

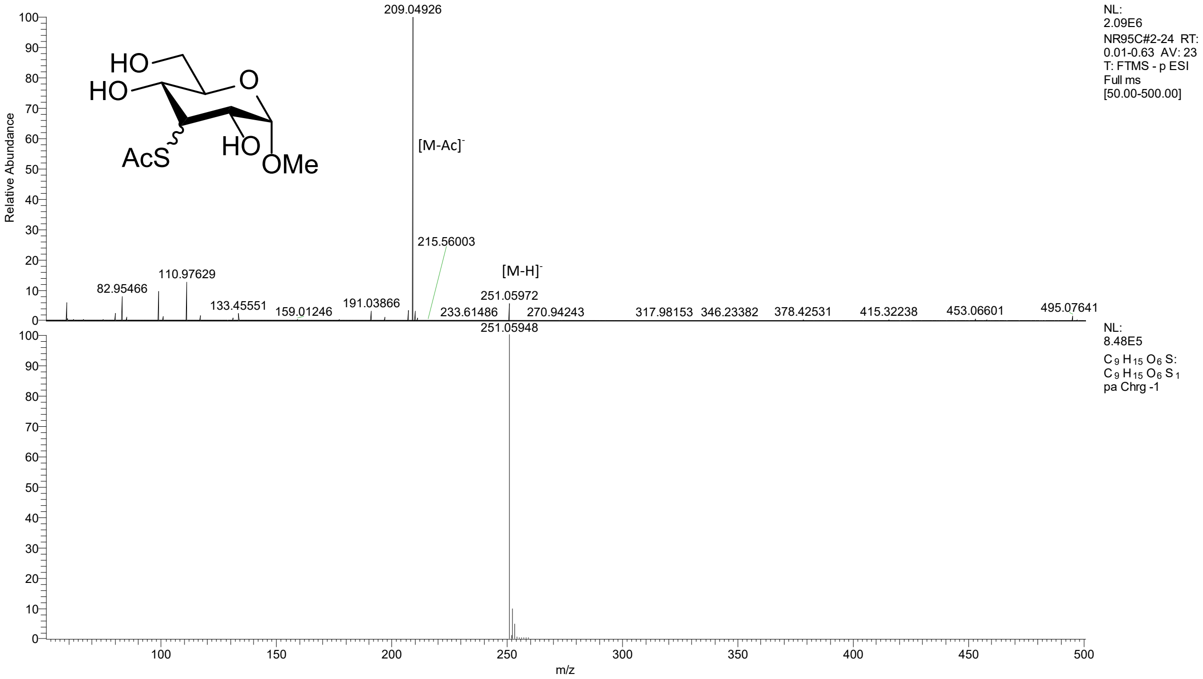

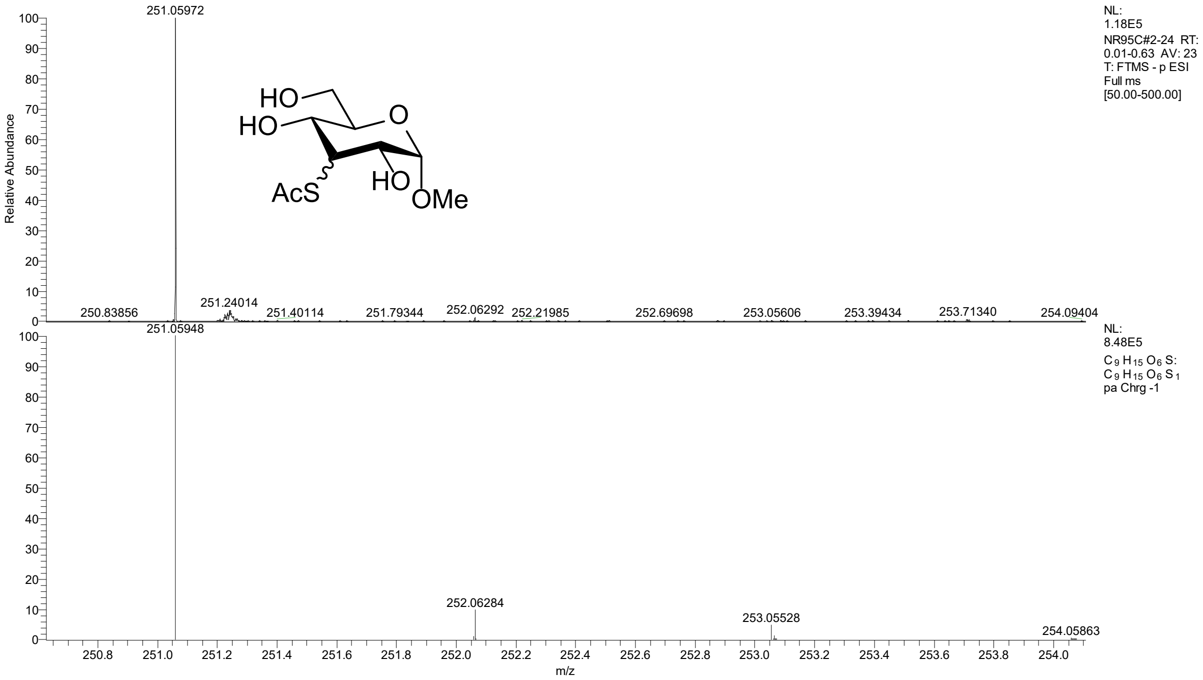

Methyl 3,3-dichloro-3-deoxy-α-D-glucopyranoside (5)

Measured with ESI neg. in the presence of guanidinium chloride.

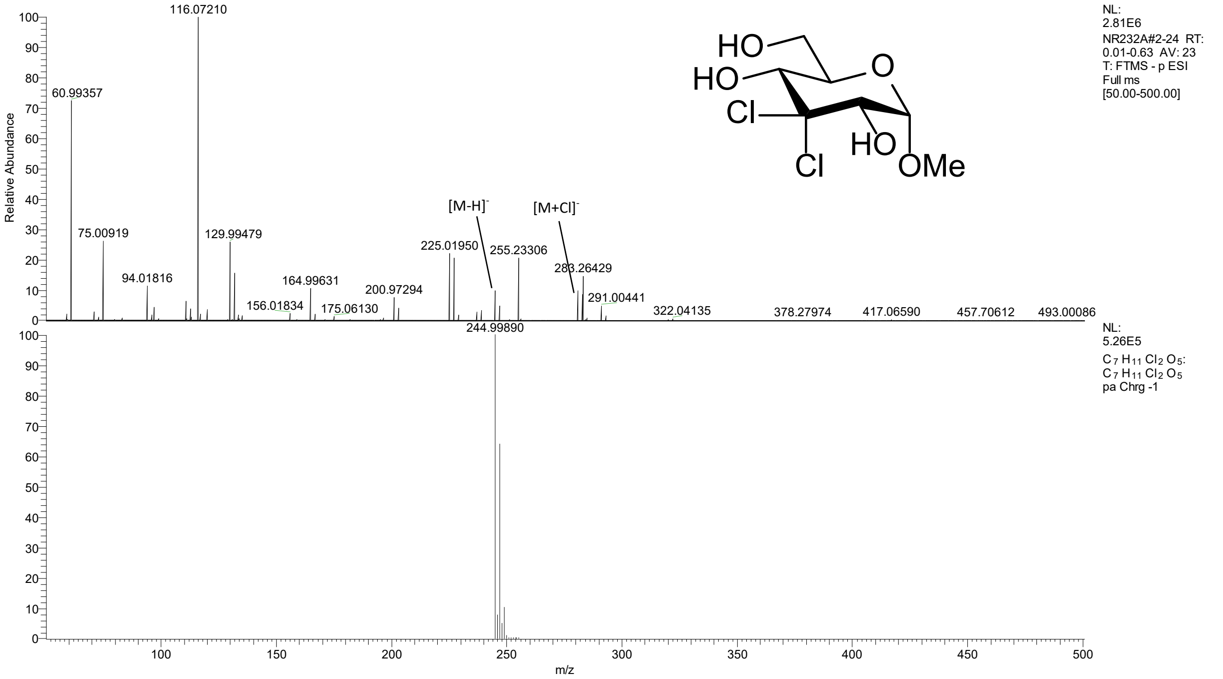

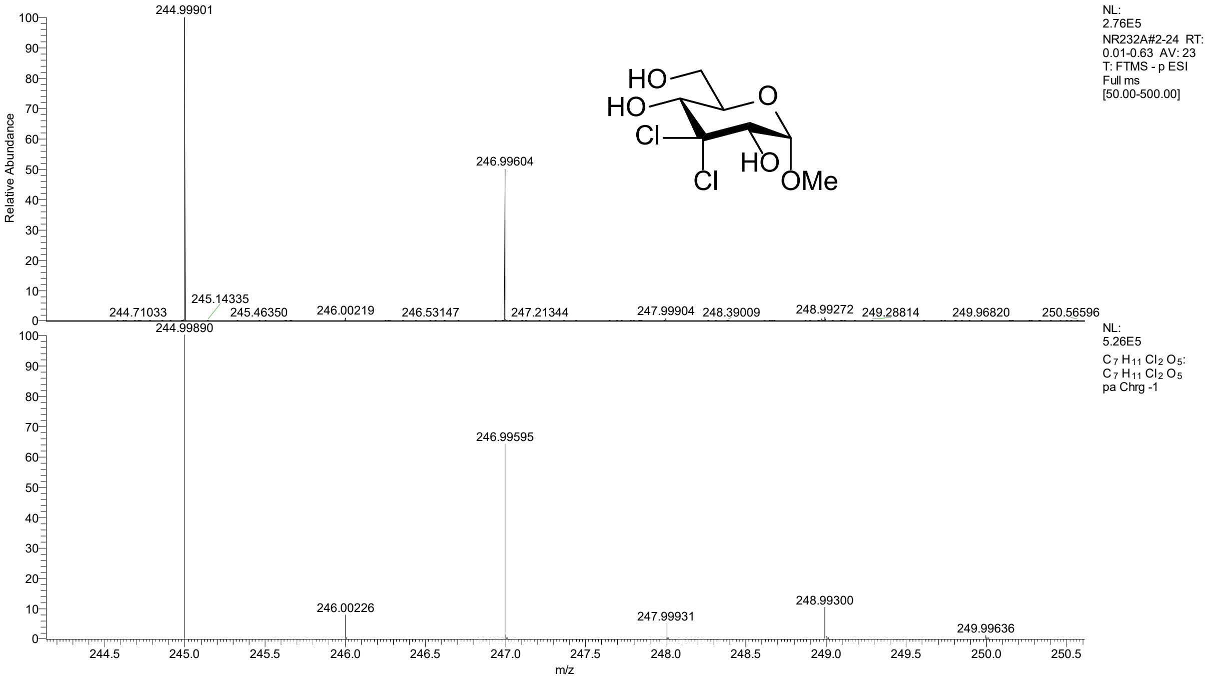

Methyl-3-(2,4-dinitrophenyl)hydrazone-α-D-glucopyranoside (6a)

Measured with ESI pos.

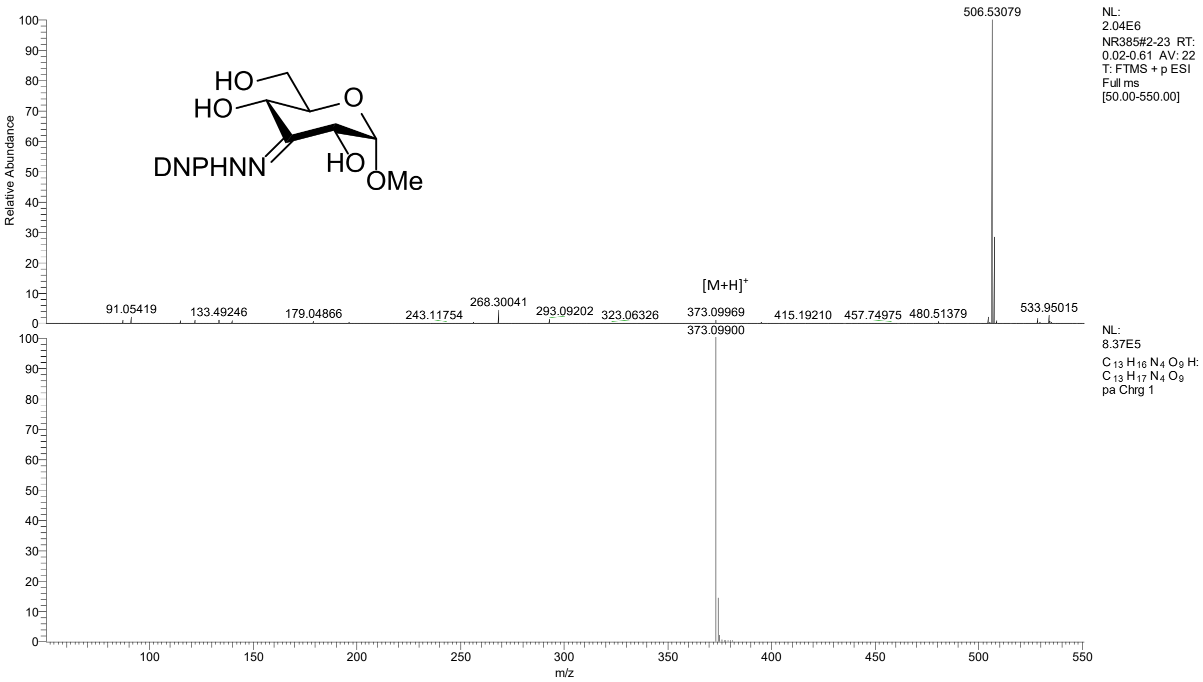

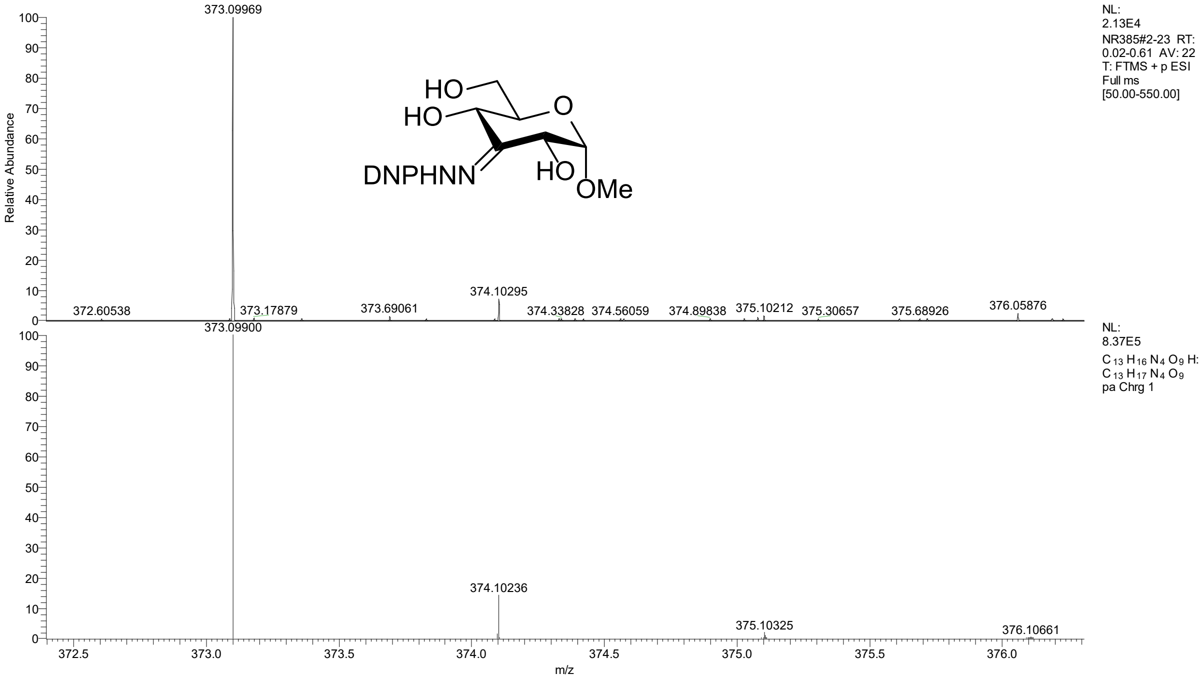

Methyl-3-chloro-3-(2,4-dinitrophenyl)diazene-α-D-glucopyranoside (7a)

Measured with ESI neg. in the presence of guanidinium chloride.

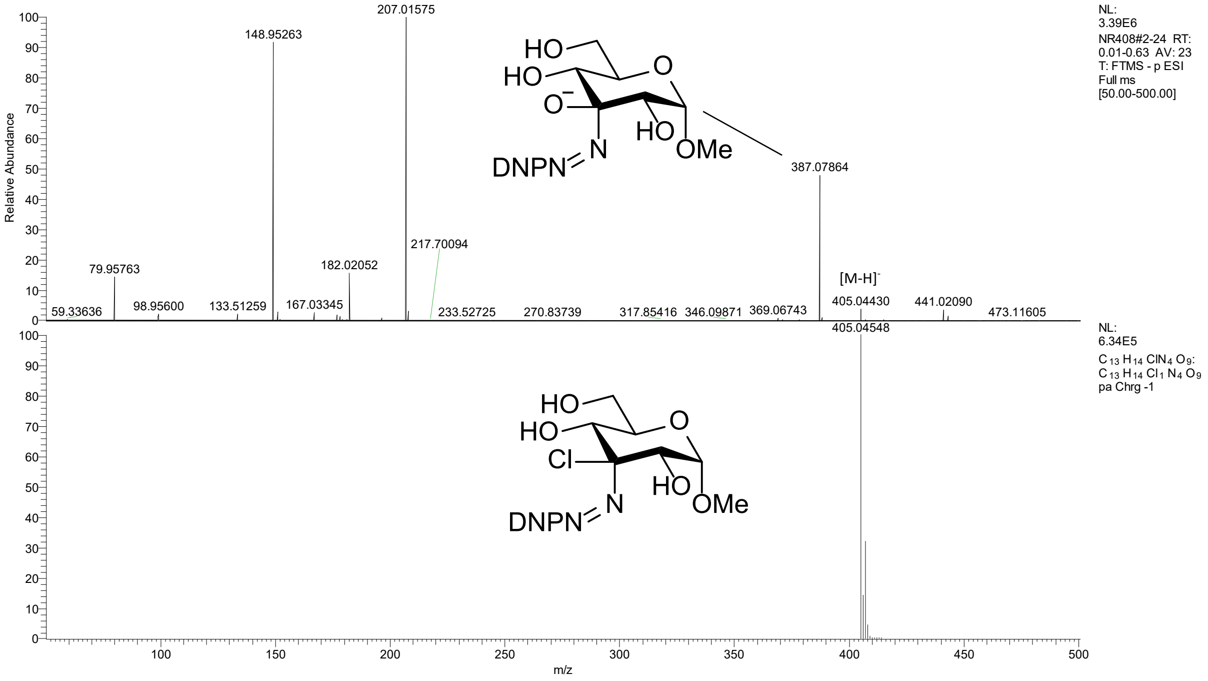

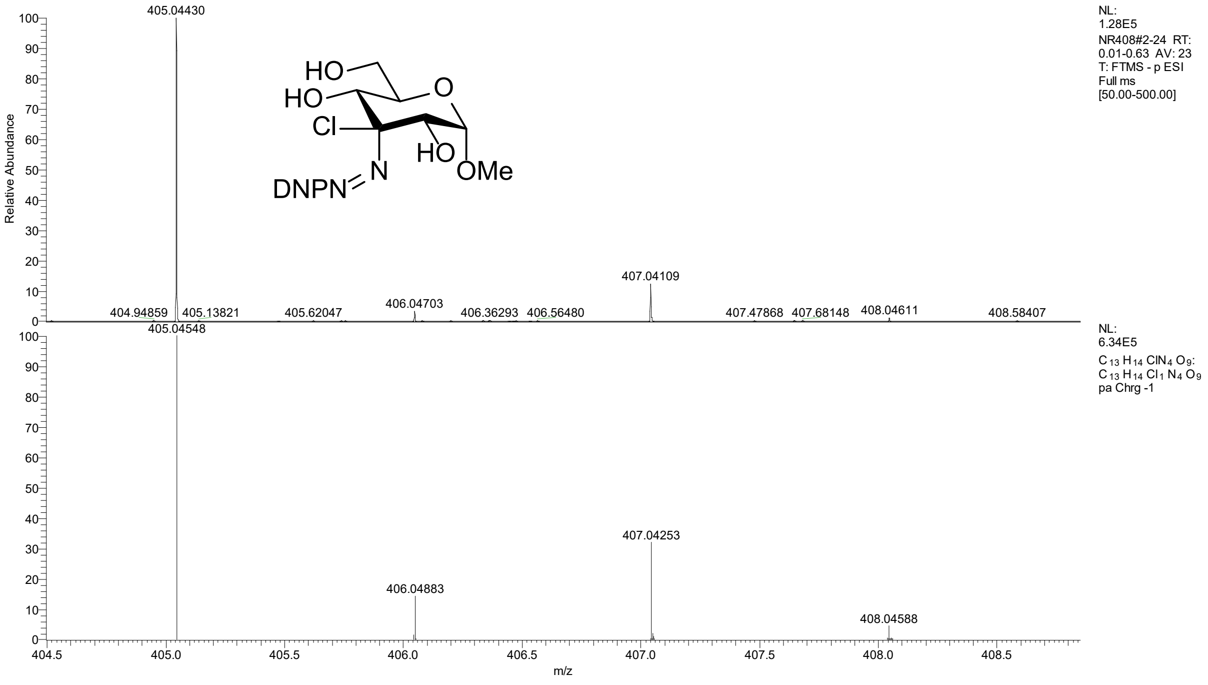

HRMS spectra of Gal derivatives

Methyl 4-(trityl)hydrazone-a-D-xylohexopyranosid-4-ulose (2c)

Measured with ESI pos.

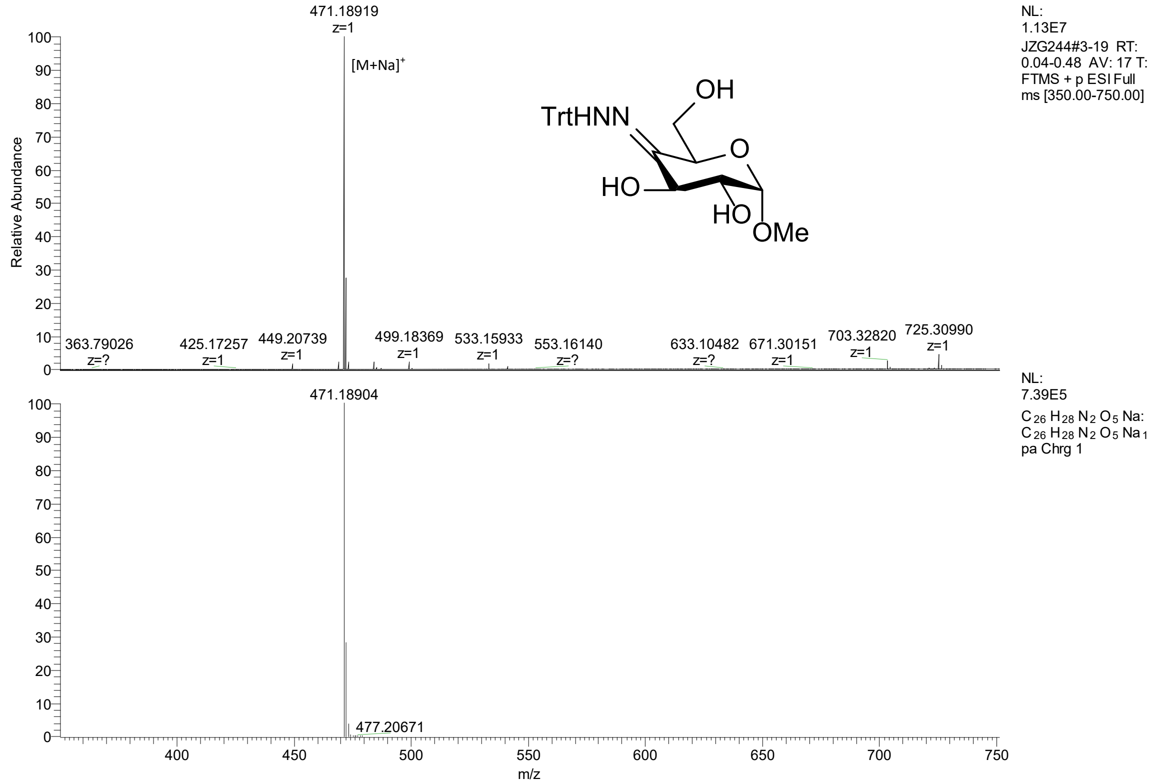

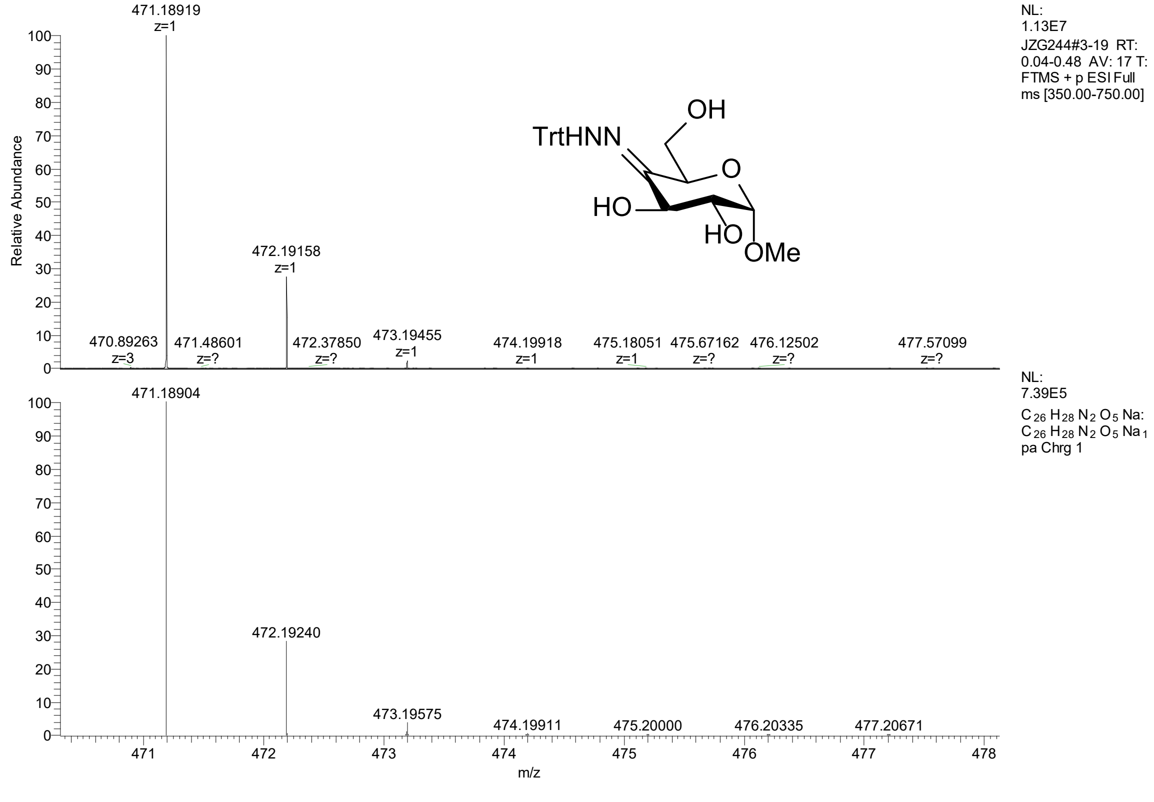

Methyl 4-chloro-4-deoxy-α-D-galacto/glucopyranoside (3c)

Measured with ESI pos.

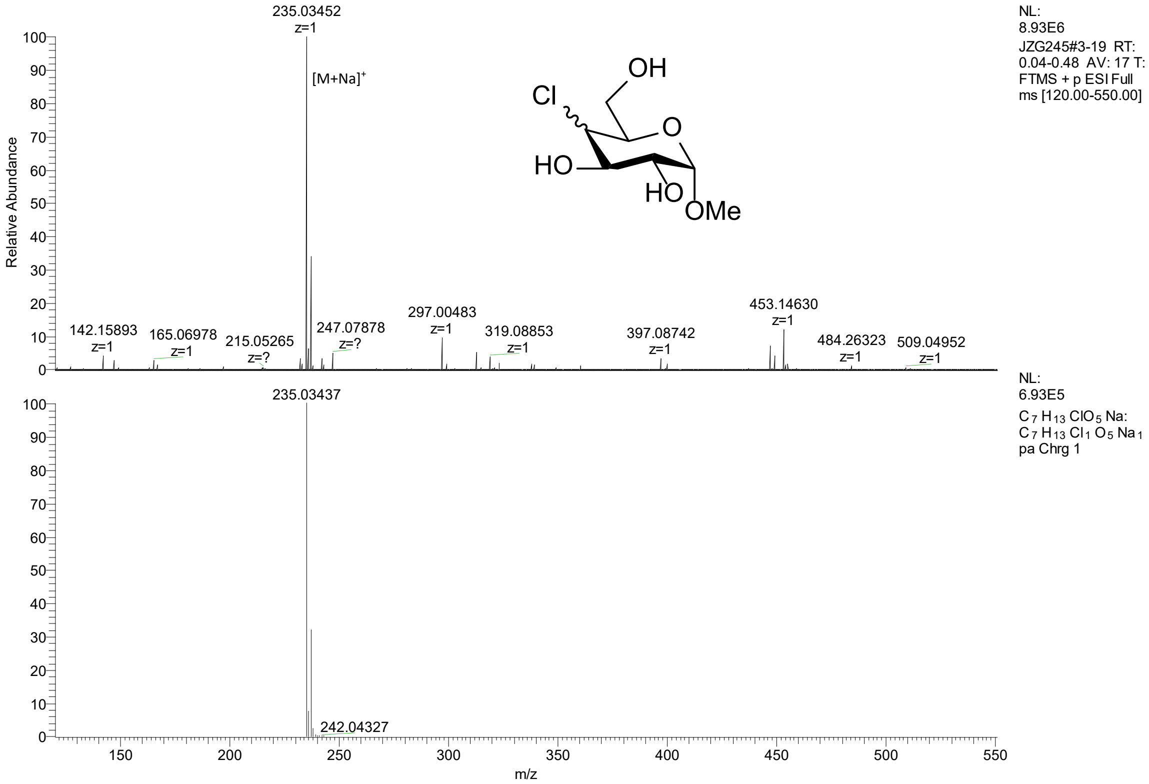

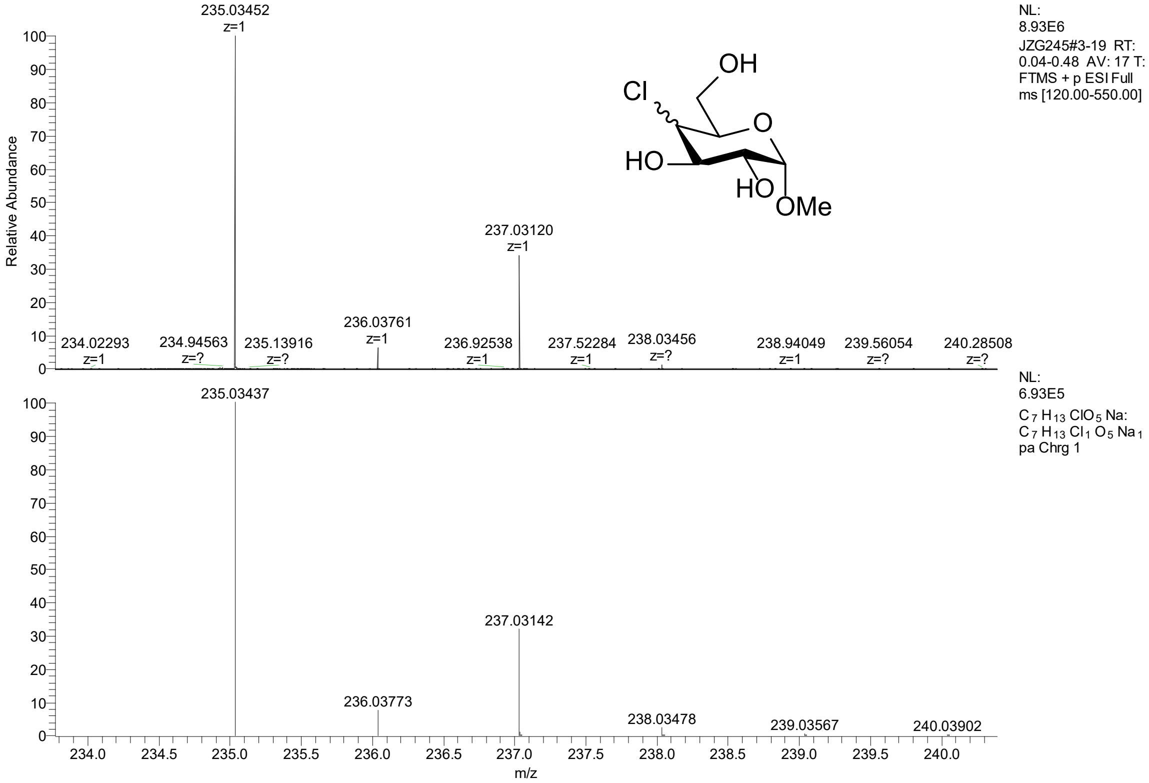

HRMS spectra of Xyl derivatives

Methyl 3-keto-α-D-xylopyranoside (1d)

Measured with ESI neg. in the presence of guanidinium chloride.

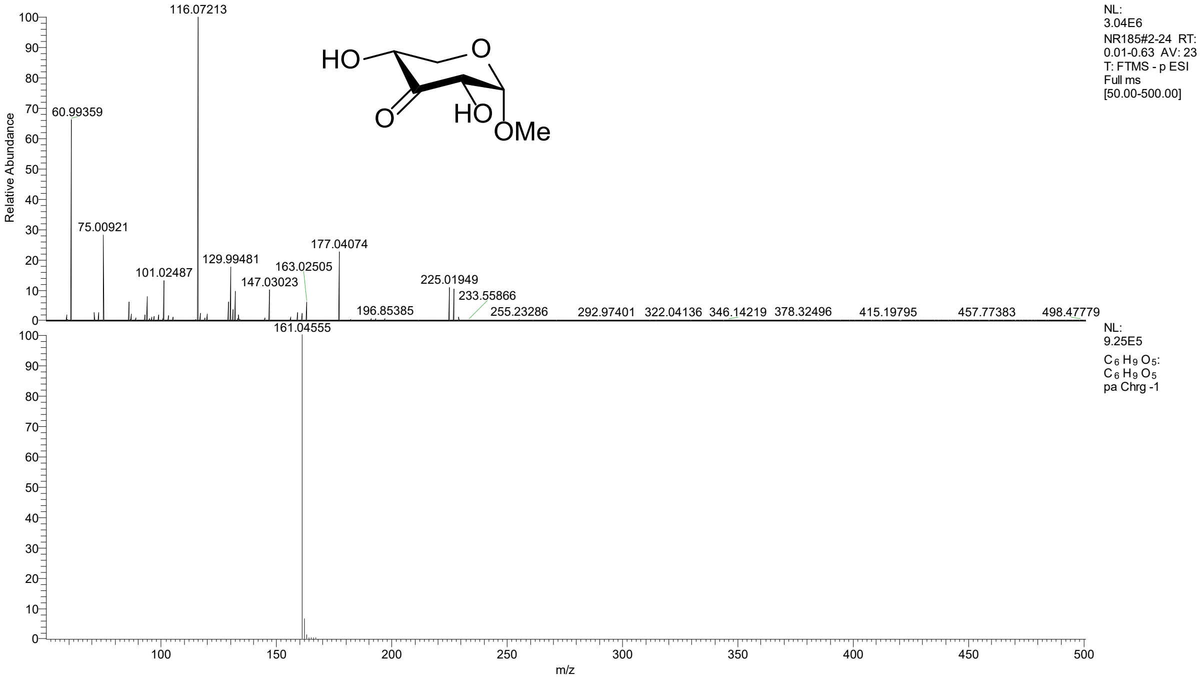

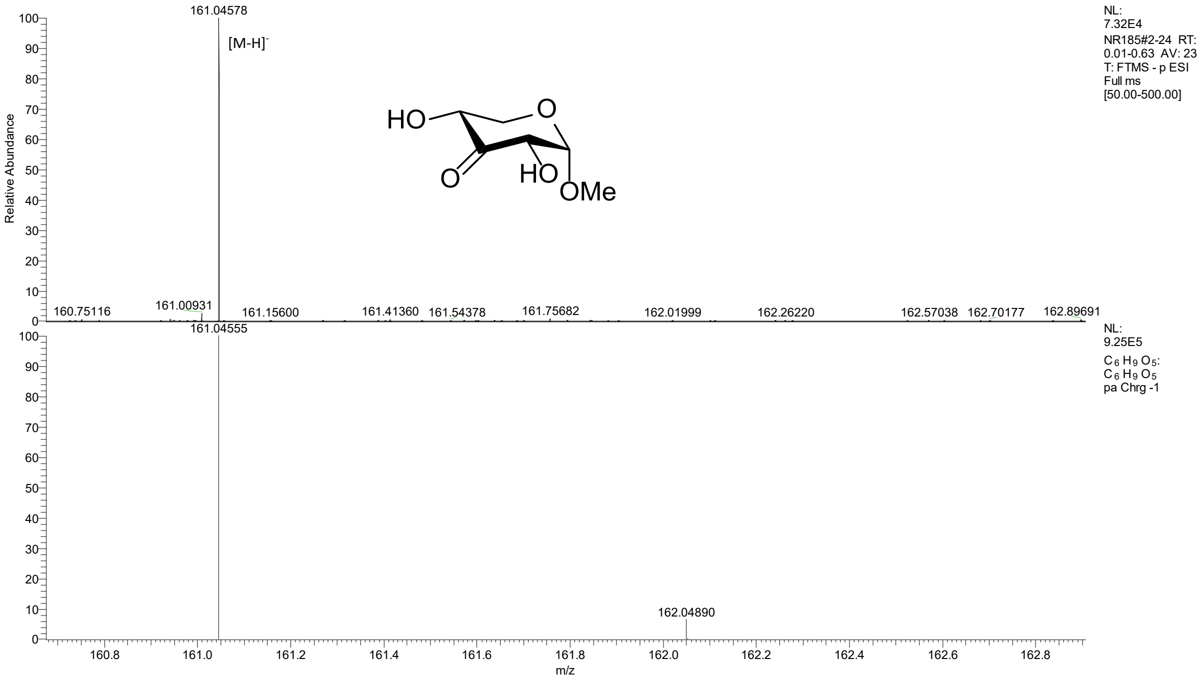

Methyl 3-(trityl)hydrazone-α-D-xylopyranoside (2d)

Measured with ESI pos.

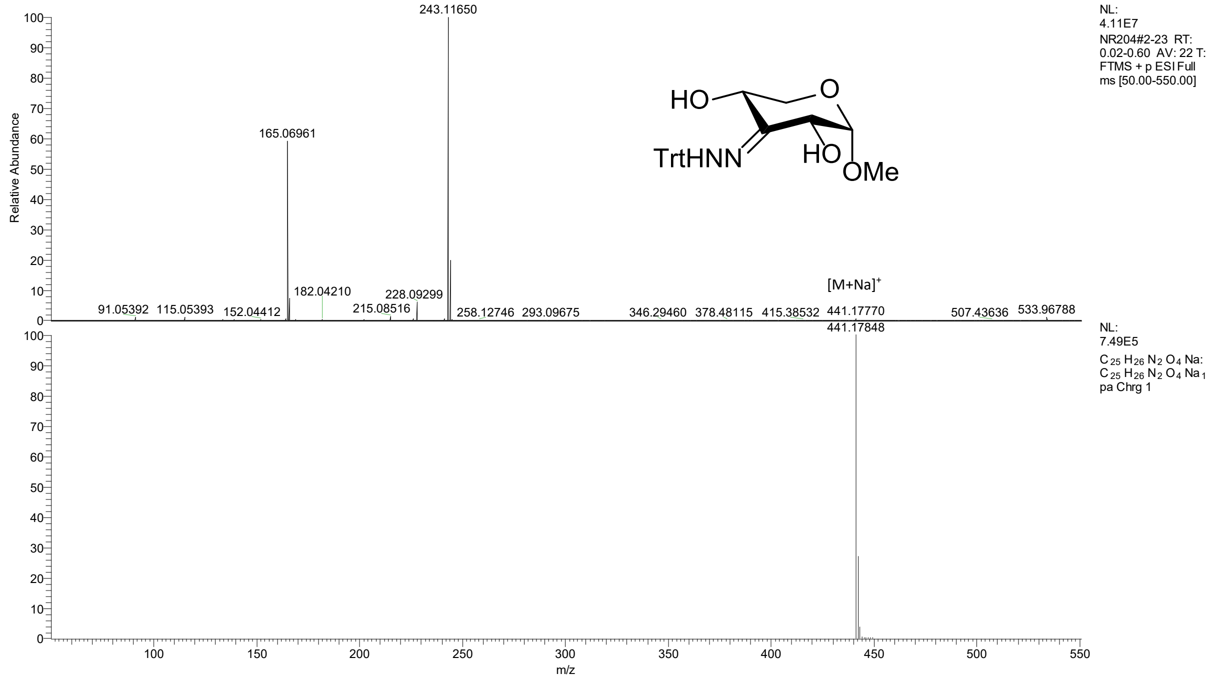

*243 and 165 come from remaining trityl material. The trityl cation causes ion suppression.

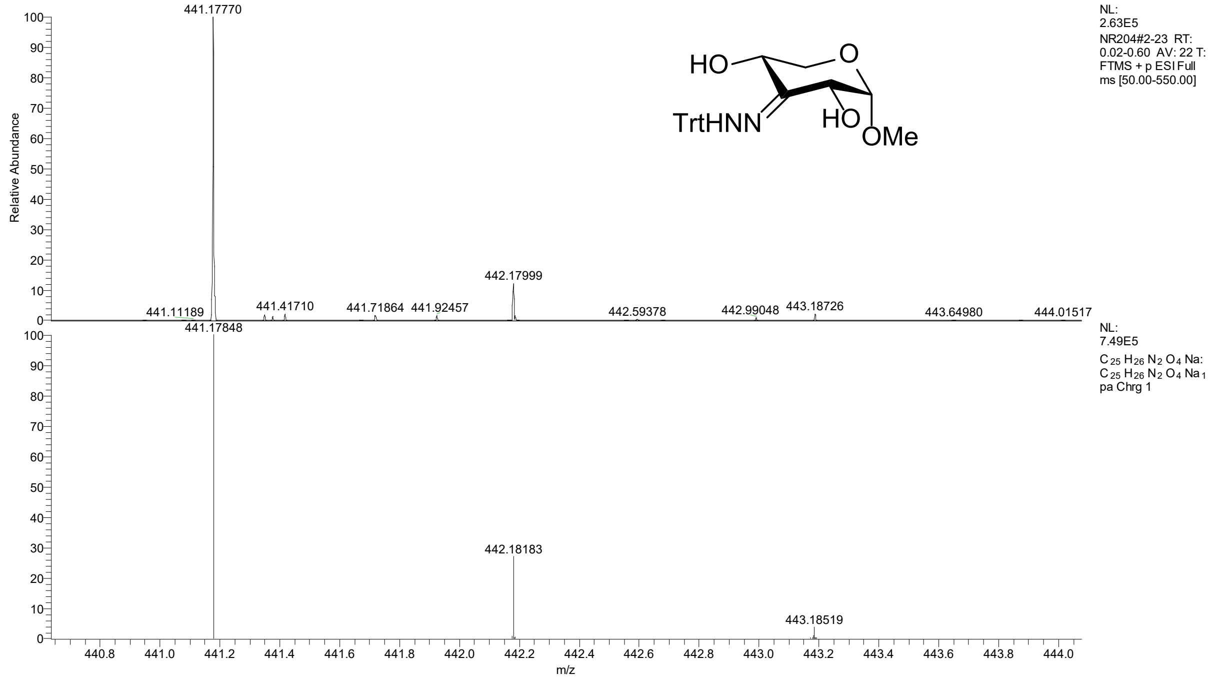

Methyl 3-chloro-3-deoxy-α-D-riboso/xylopyranoside (3d)

Measured with ESI neg. in the presence of guanidinium chloride.

Equatorial:

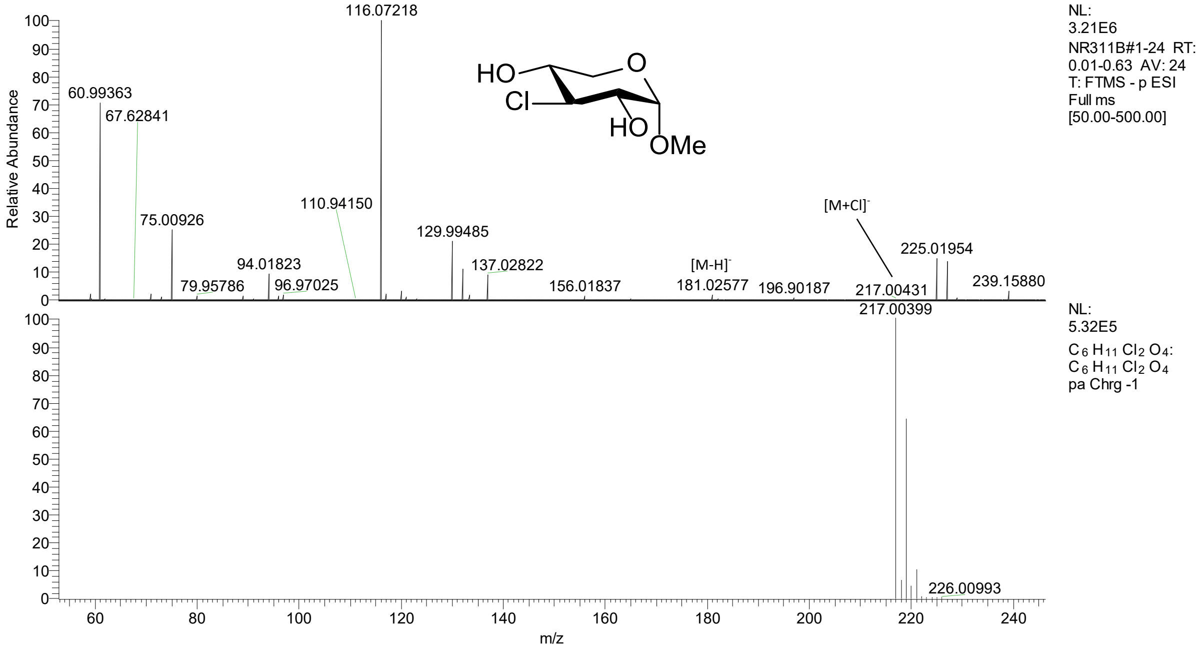

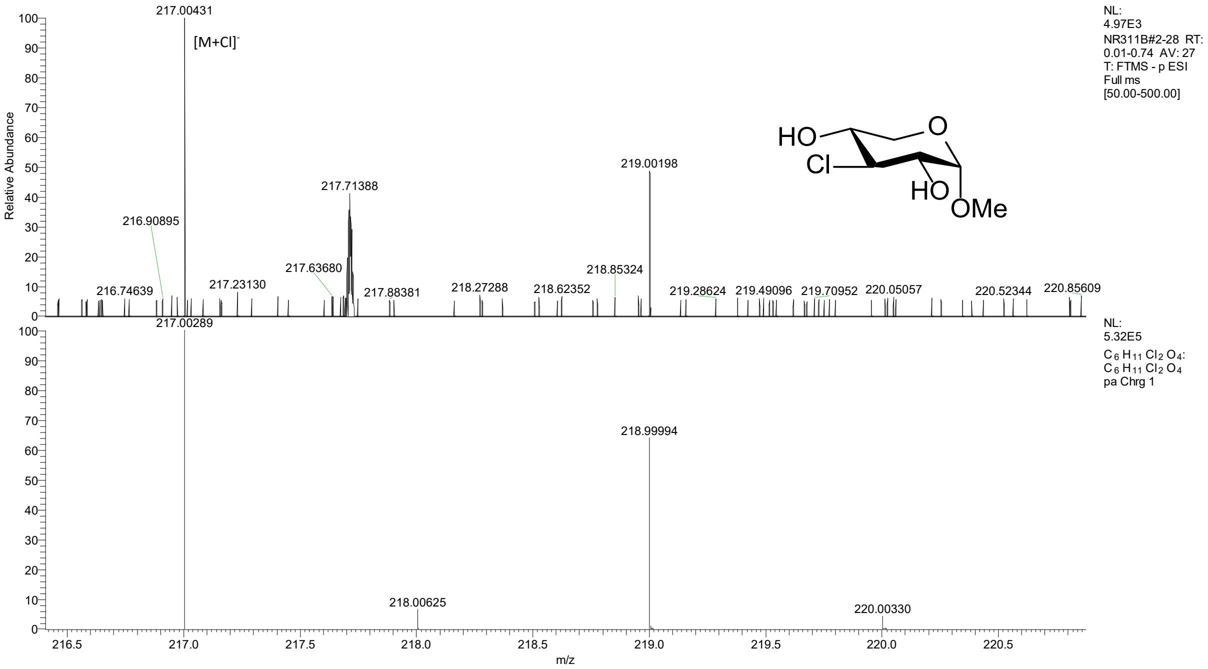

Axial:

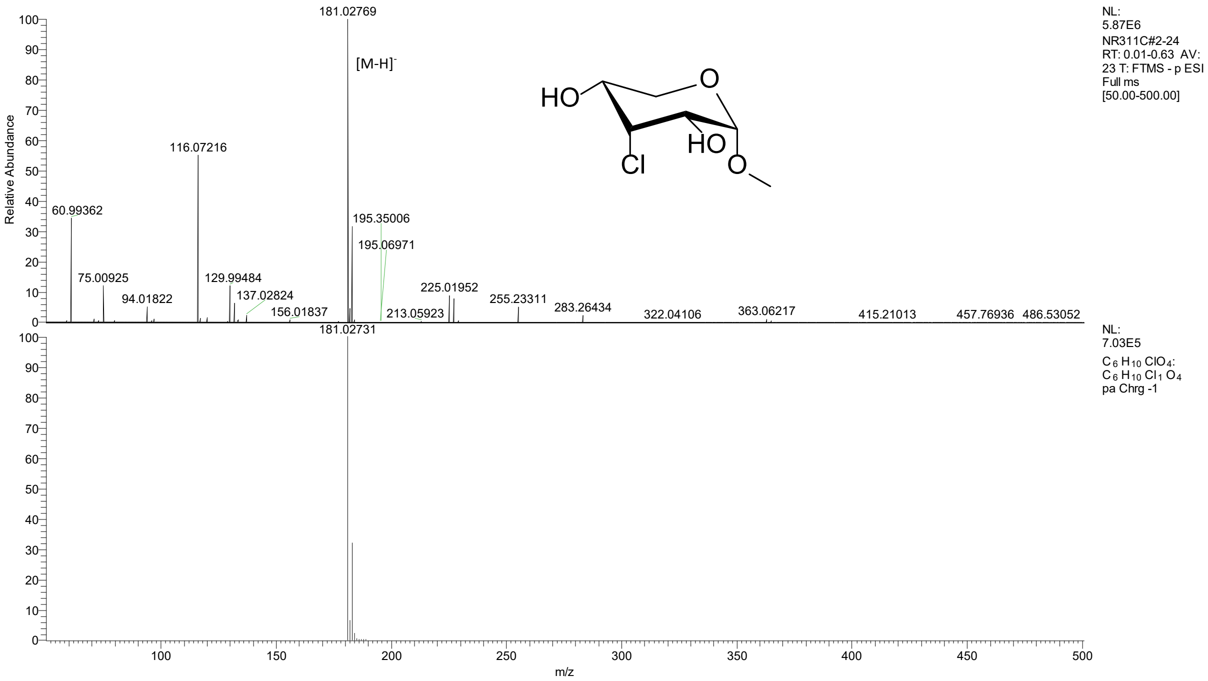

181.02769
[M-H]-
116.07216
60.99362
195.35006
195.06971
75.00925
129.99484
94.01822
137.02824
156.01837
225.01952
213.05923
255.23311
283.26434
322.04106
363.06217
415.21013
457.76936
486.53052
181.02731
HO
O
HO
Cl
O
Relative Abundance
m/z
NL:
5.87E6
NR311C#2-24
RT: 0.01-0.63 AV:
23 T: FTMS - p ESI
Full ms
[50.00-500.00]
NL:
7.03E5
C6 H10 ClO4:
C6 H10 Cl1 O4
pa Chrg -1

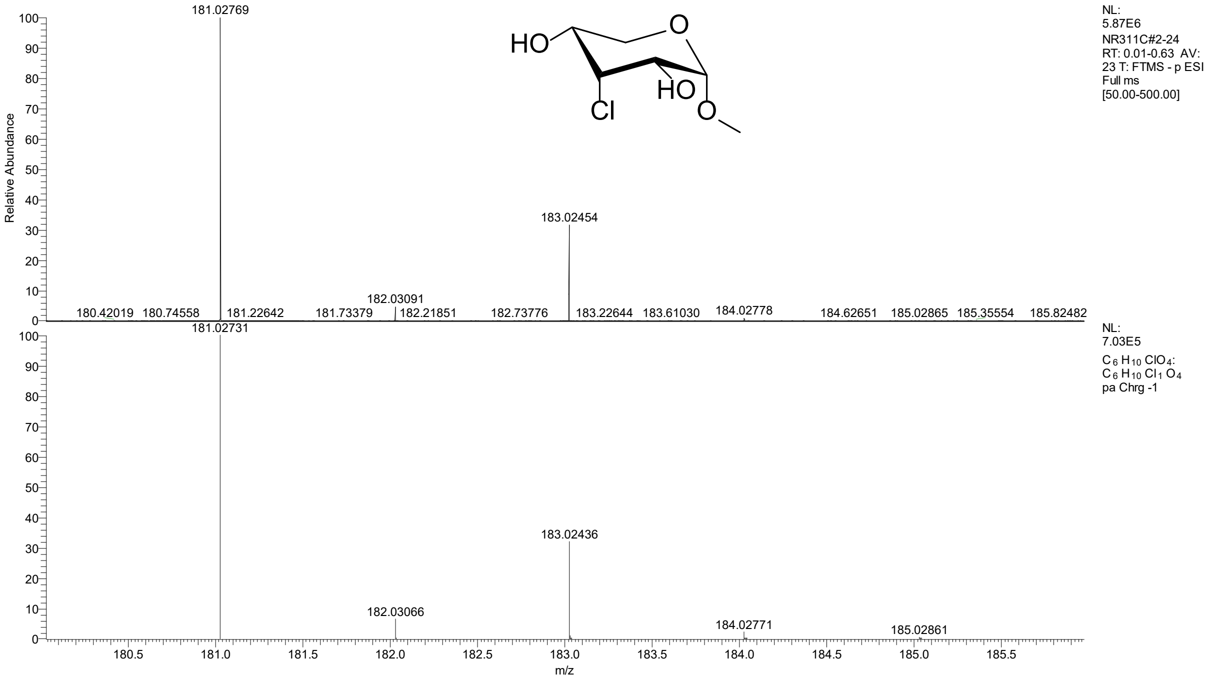

181.02769
183.02454
180.42019
180.74558
181.22642
181.73379
182.03091
182.21851
182.73776
183.22644
183.61030
184.02778
184.62651
185.02865
185.35554
185.82482
181.02731
183.02436
182.03066
184.02771
185.02861
HO
O
HO
Cl
O
Relative Abundance
m/z
NL:
5.87E6
NR311C#2-24
RT: 0.01-0.63 AV:
23 T: FTMS - p ESI
Full ms
[50.00-500.00]
NL:
7.03E5
C6 H10 ClO4:
C6 H10 Cl1 O4
pa Chrg -1

HRMS spectra of cellobiose derivatives

2,3,6,2',3',4',6'-Hepta-*O*-acetyl-cellobiose (S4)

Measured with ESI pos.

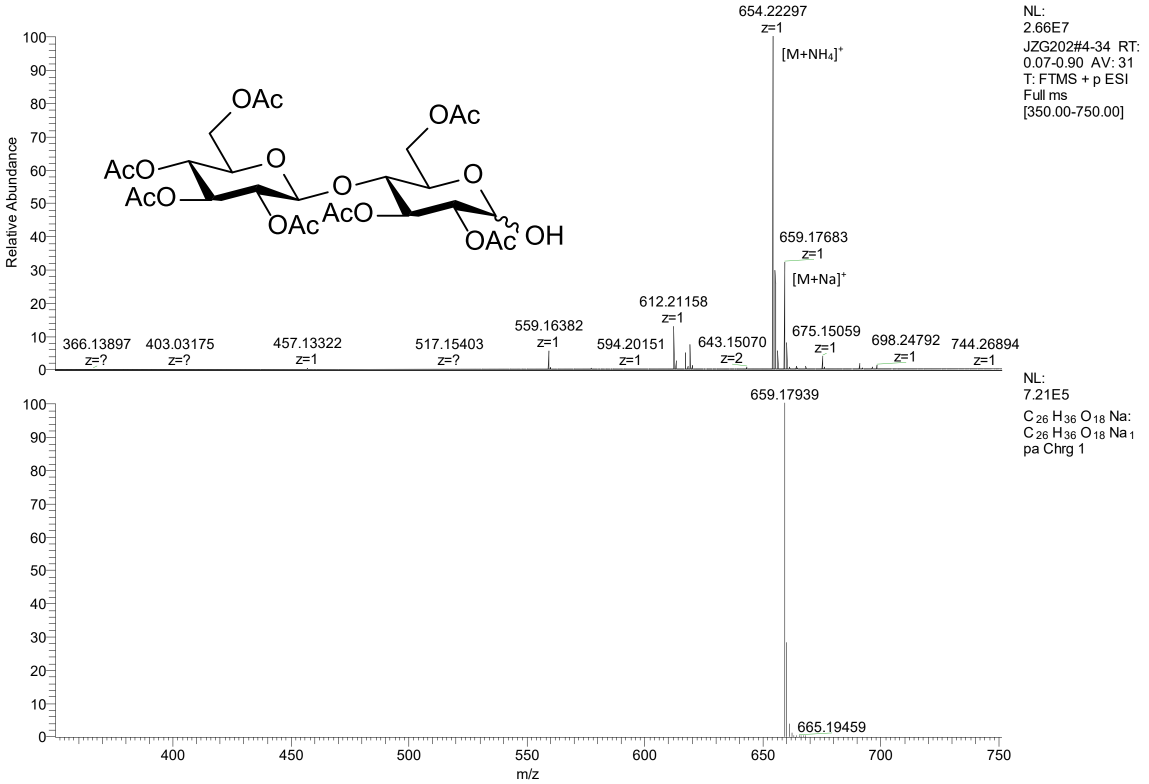

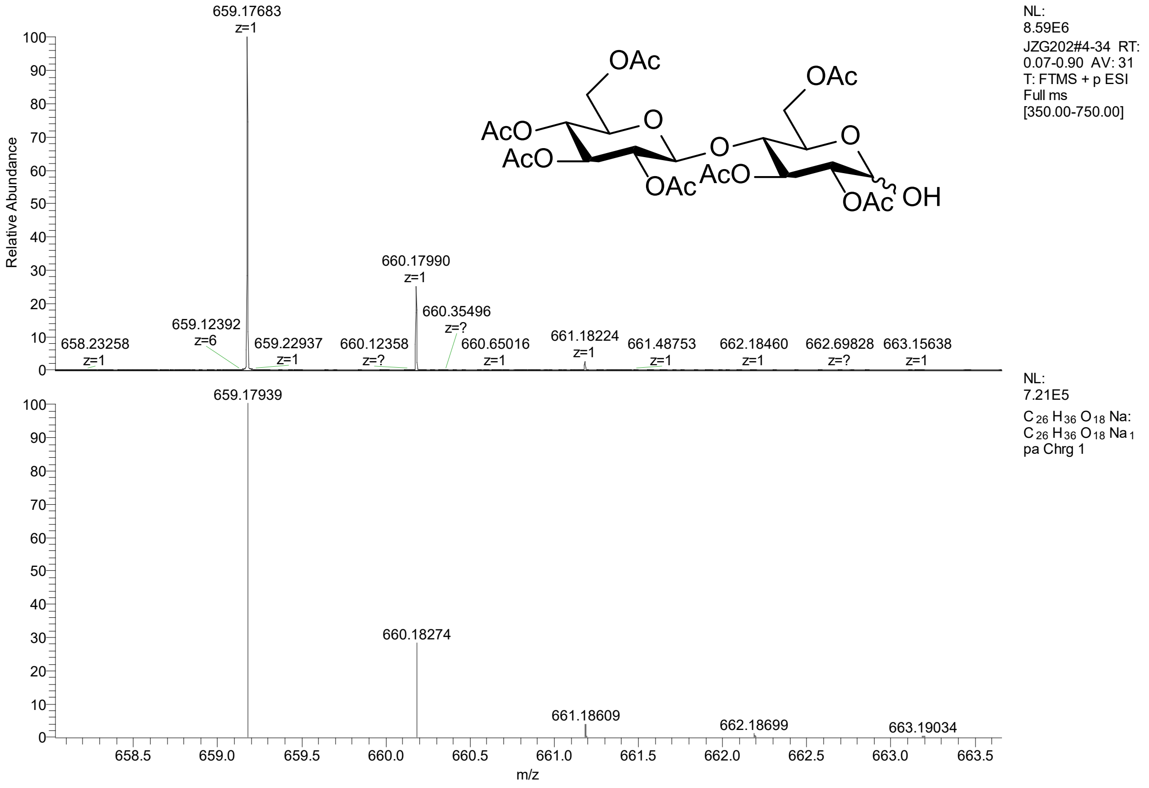

4-tert-butylbenzyl-β-D-heptaacetyl-cellobioside (S5)

Measured with ESI pos.

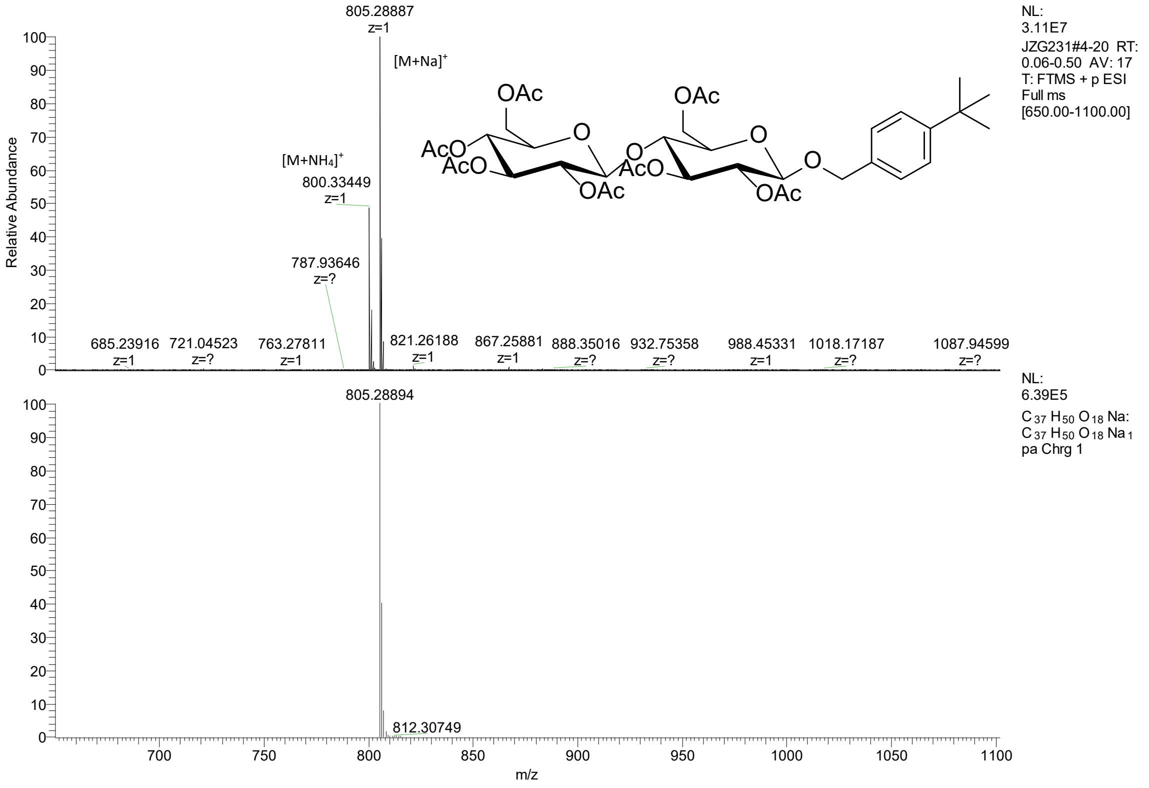

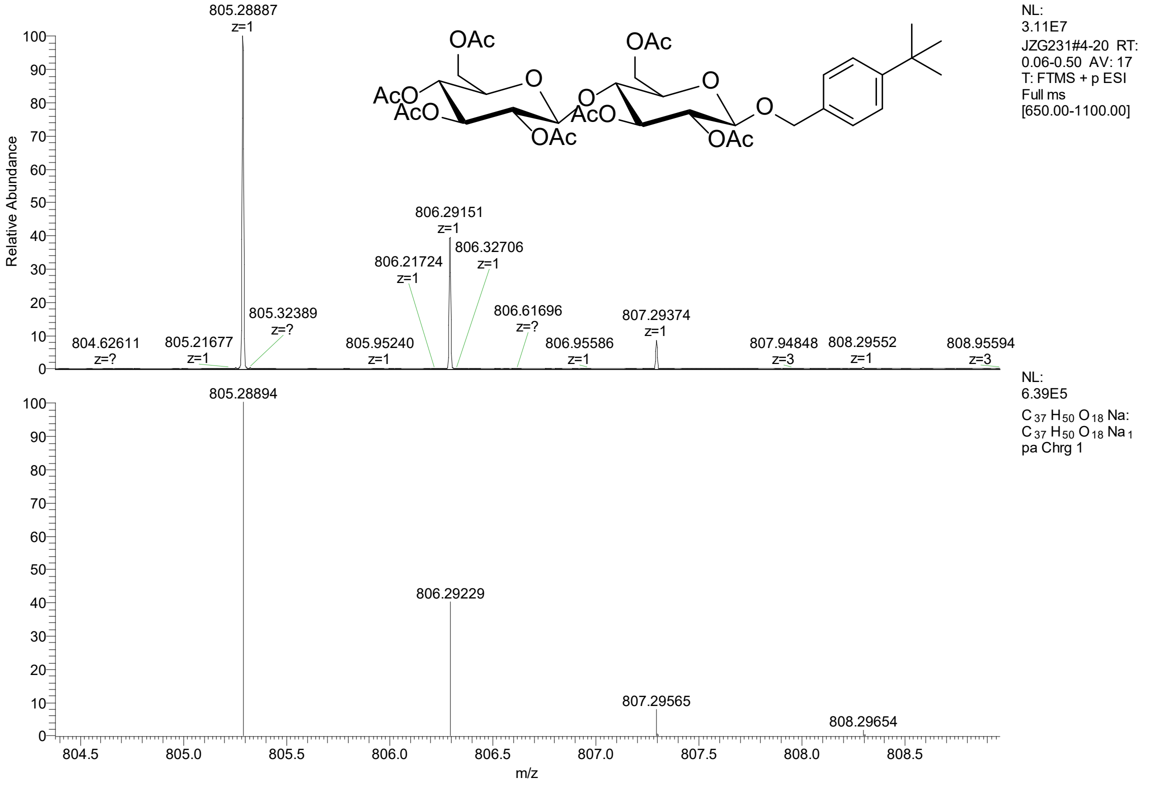

4-tert-butylbenzyl-β-D-cellobioside (S6)

Measured with ESI pos.

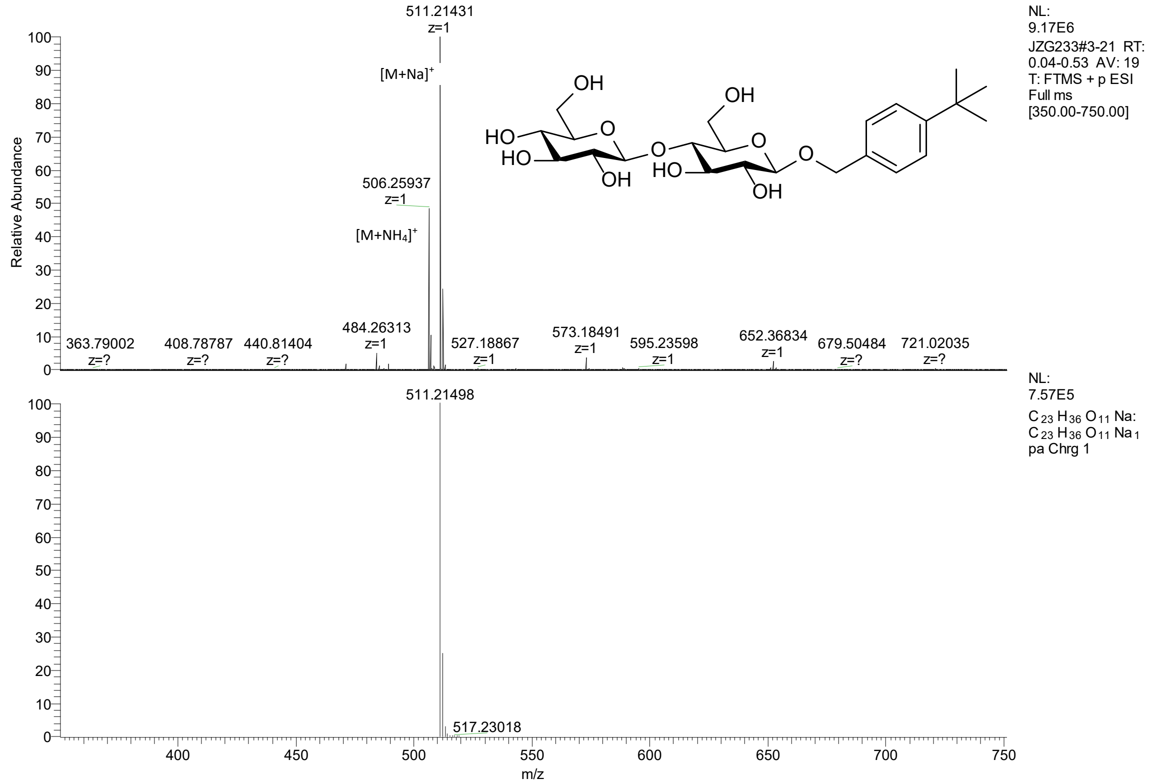

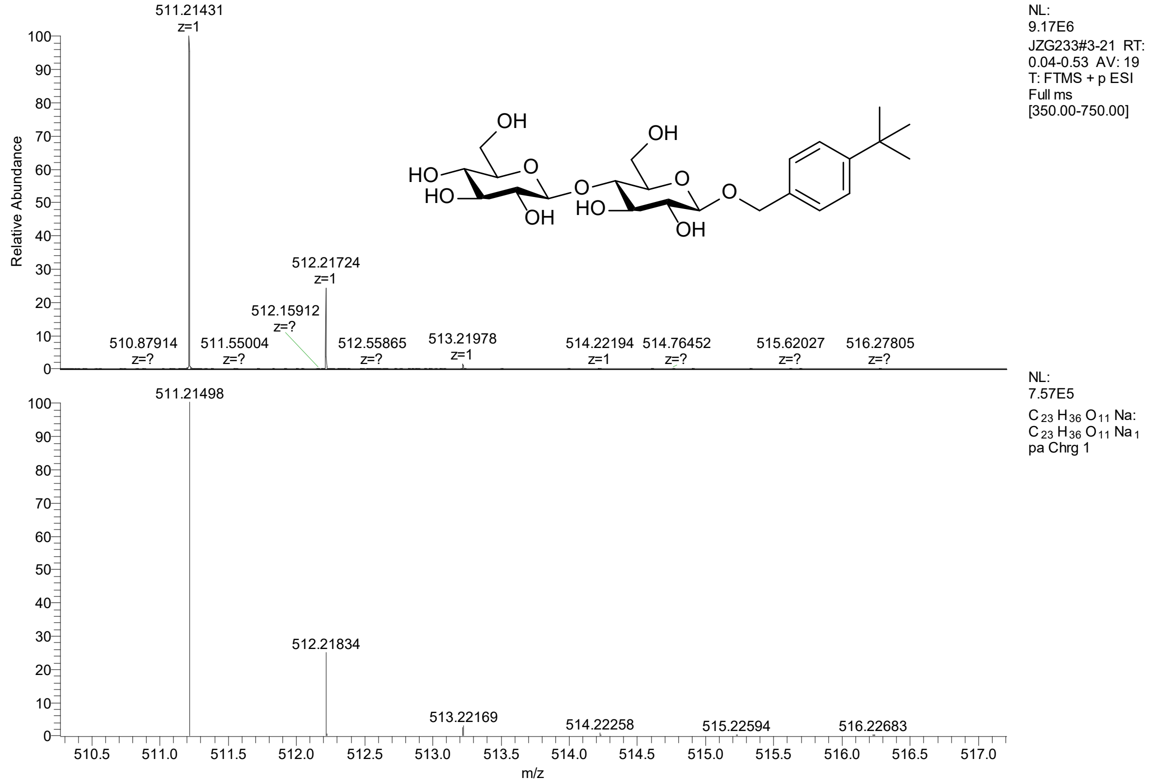

4-tert-butylbenzyl-β-3-ketocellobioside (1e)

Measured with ESI pos.

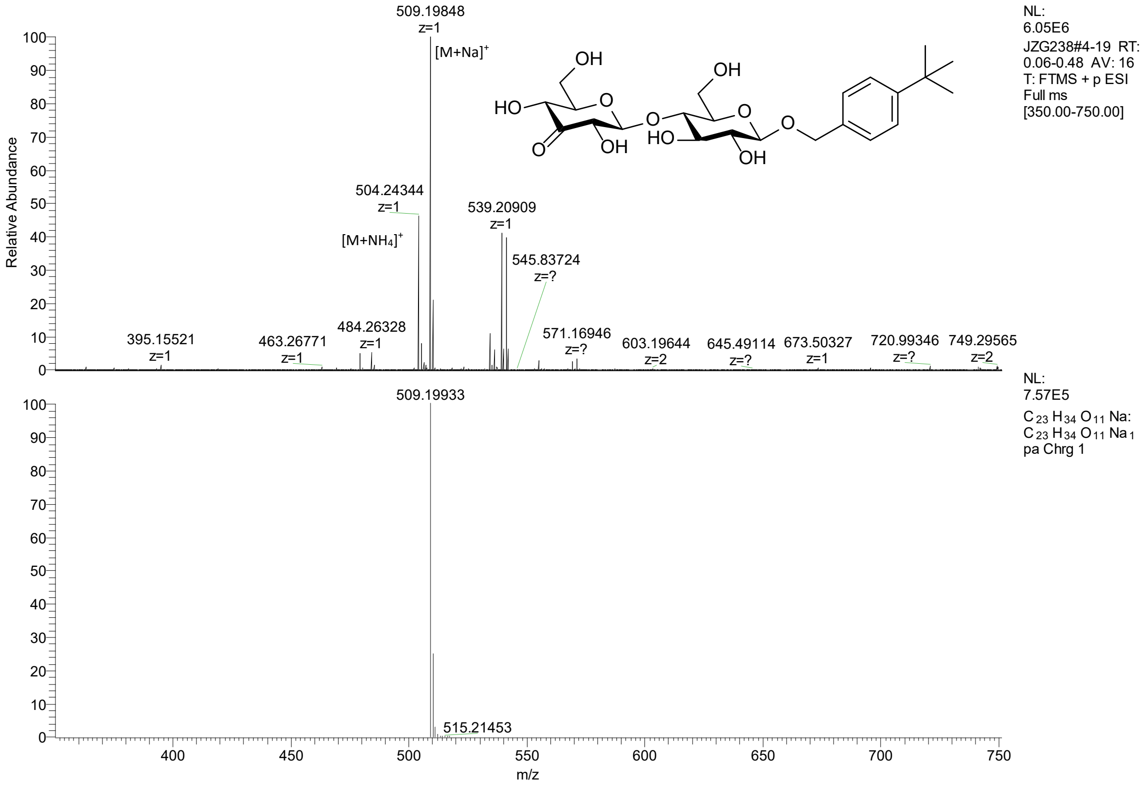

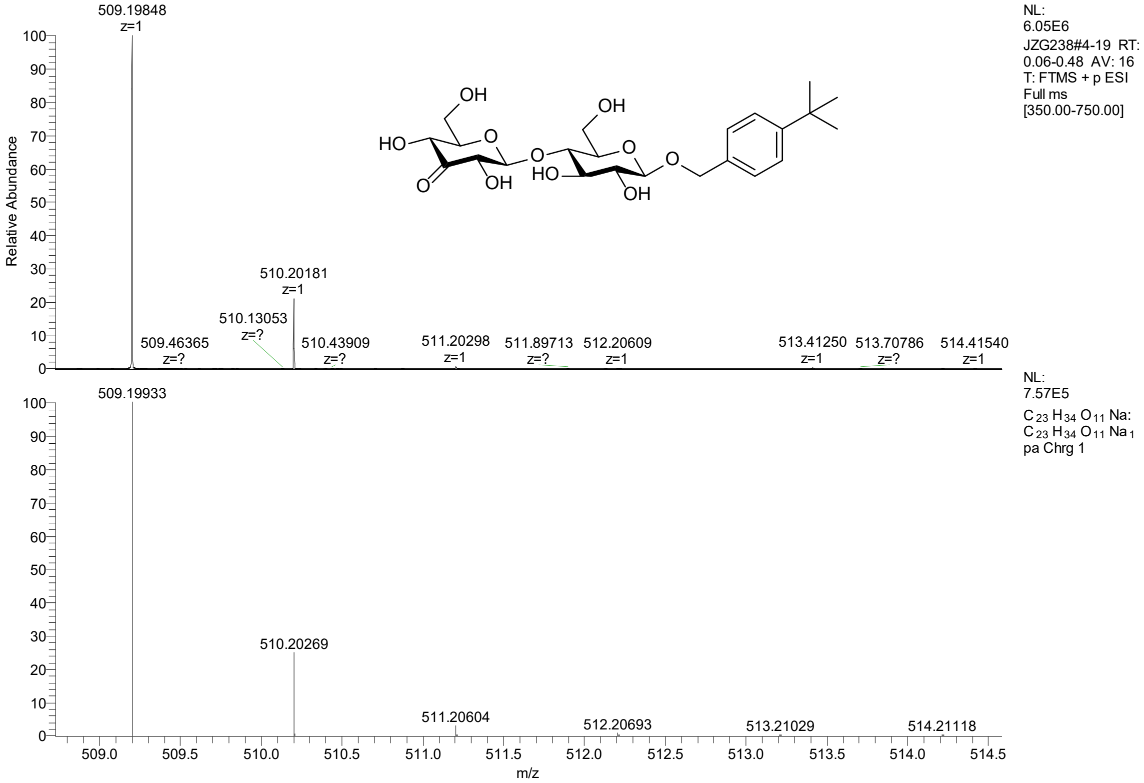

4-tert-butylbenzyl-3-(trityl)hydrazone-β-D-cellobioside (2e)

Measured with ESI pos.

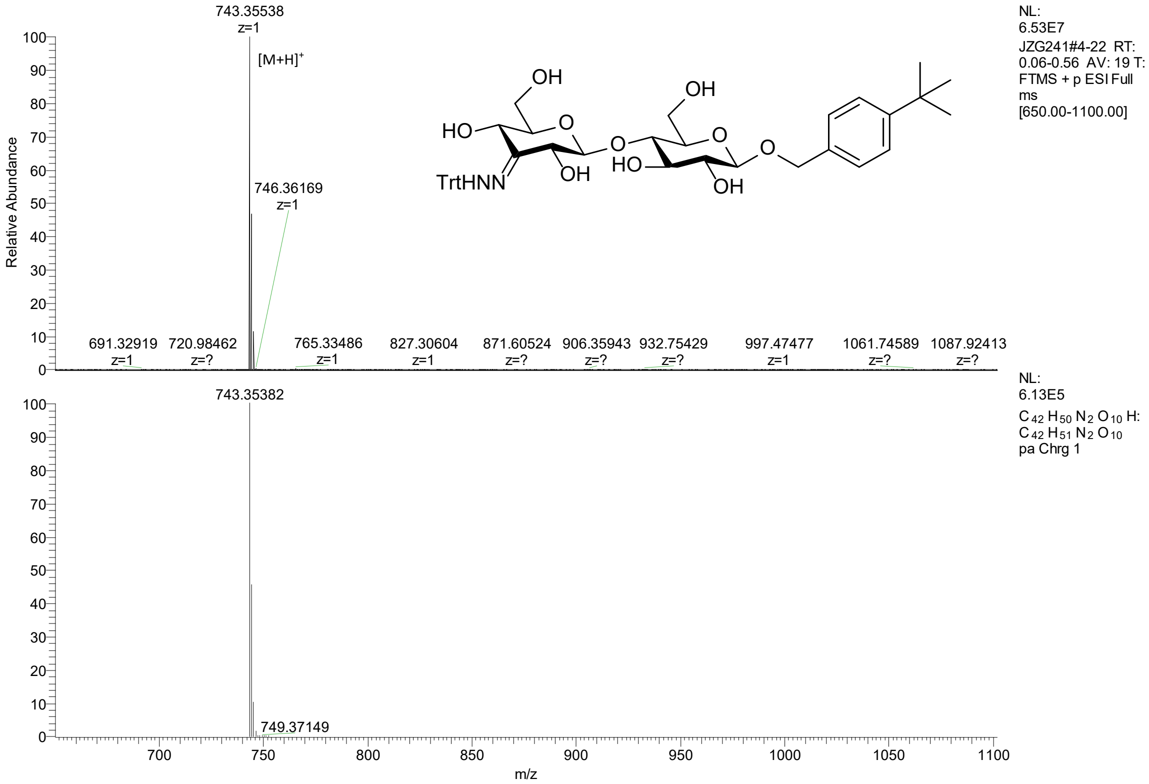

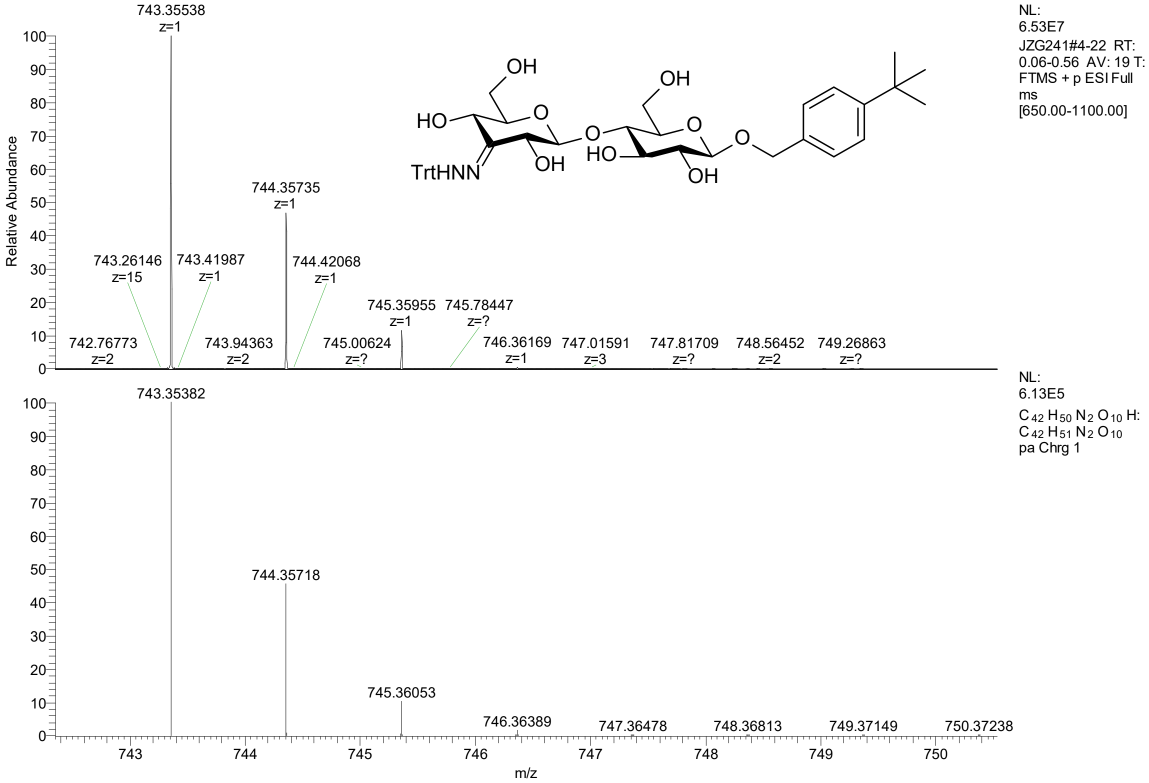

4-tert-butylbenzyl-3-chloro-3-deoxy-β-D-cellobioside (3e)

Measured with ESI pos.

Equatorial:

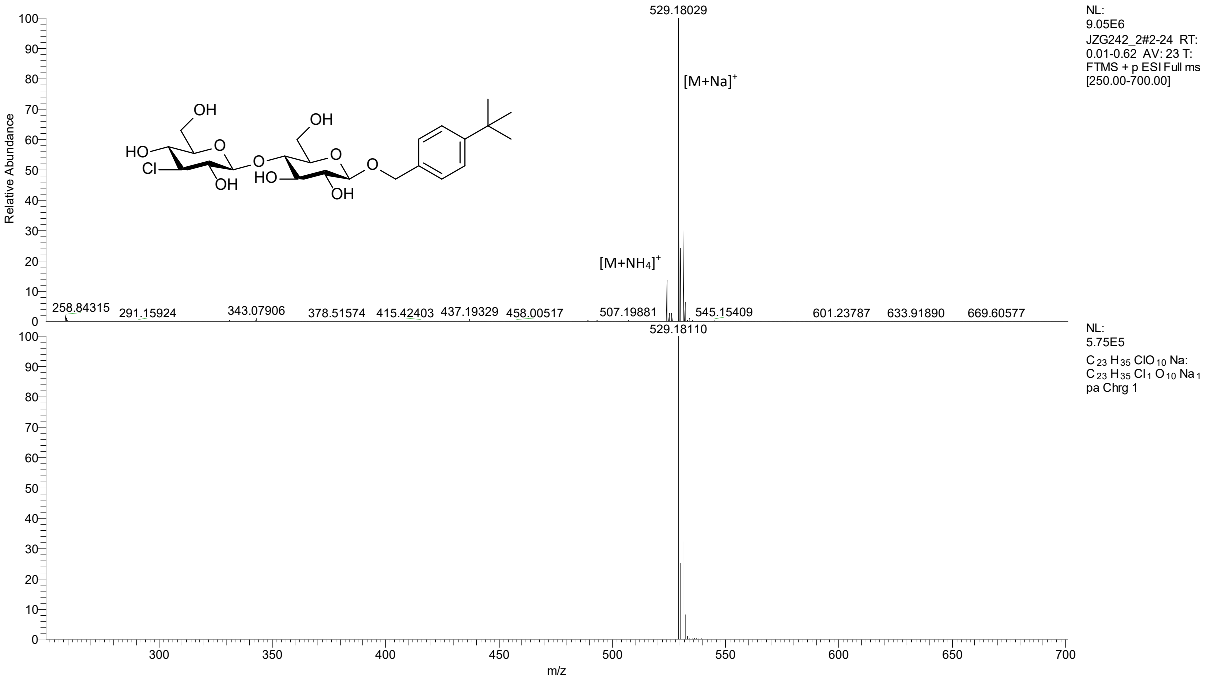

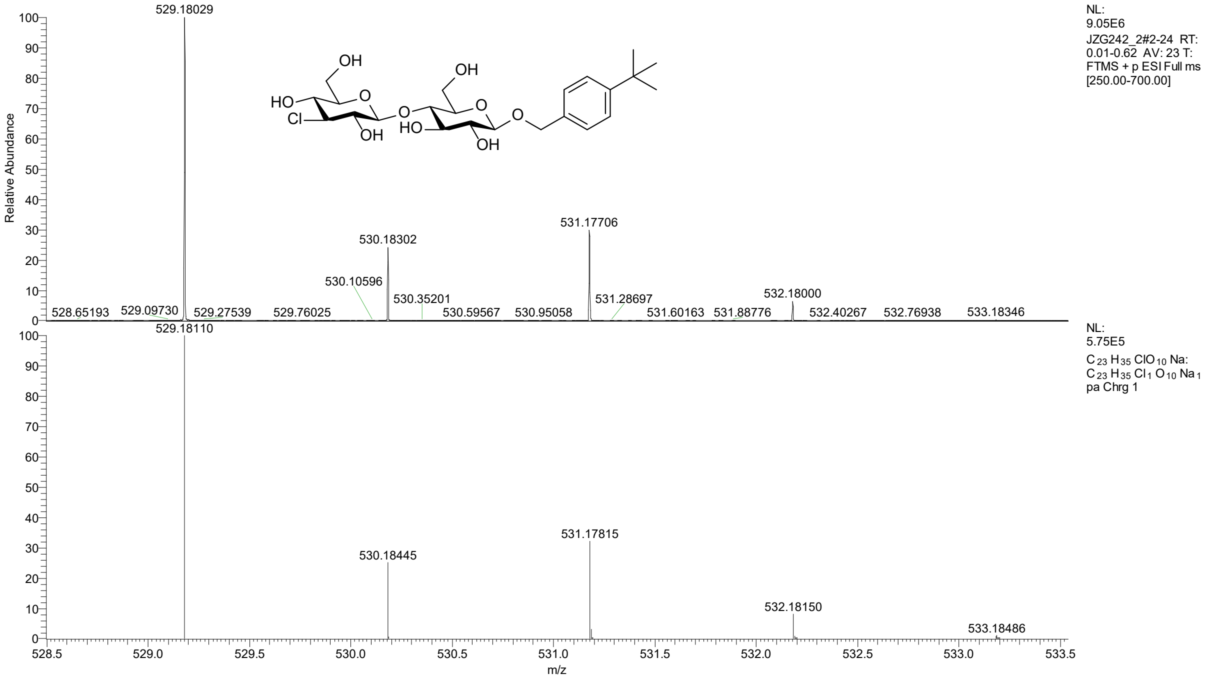

Axial:

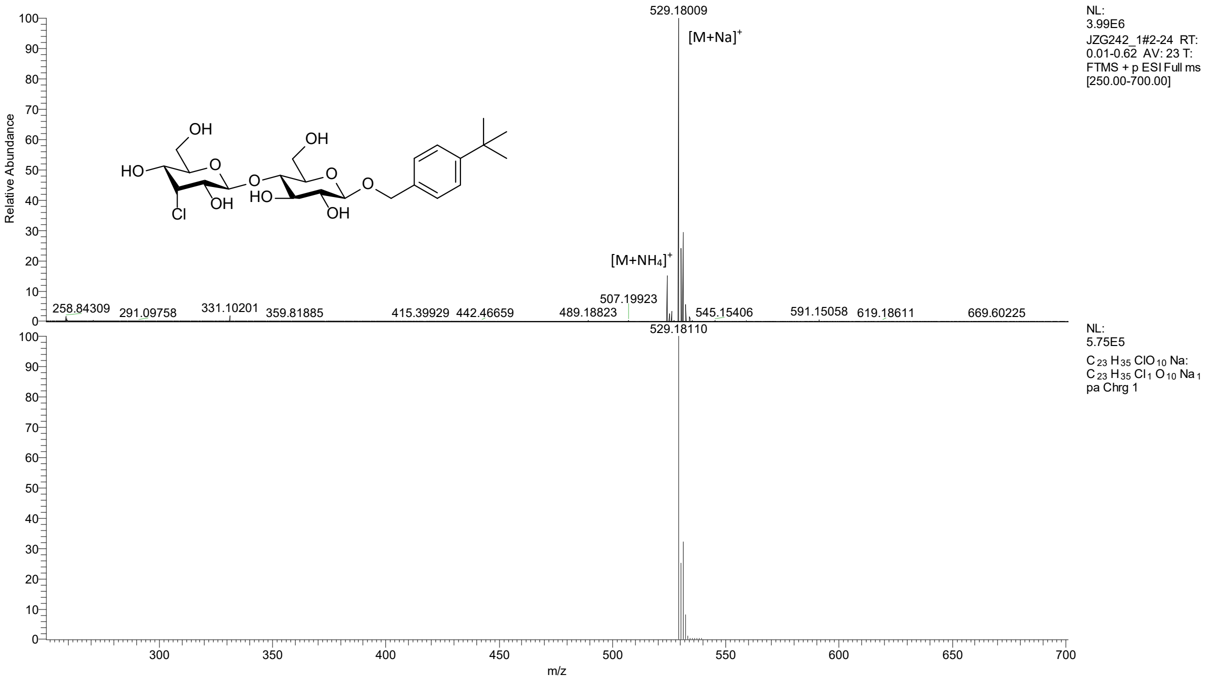
529.18009
[M+Na]+
[M+NH4]+
507.19923
258.84309
291.09758
331.10201
359.81885
415.39929
442.46659
489.18823
545.15406
591.15058
619.18611
669.60225
529.18110
NL:
3.99E6
JZG242_1#2-24 RT:
0.01-0.62 AV: 23 T:
FTMS + p ESI Full ms
[250.00-700.00]
NL:
5.75E5
C 23 H35 ClO10 Na:
C 23 H35 Cl1 O10 Na1
pa Chrg 1
Relative Abundance
m/z
HO
OH
O
Cl
OH
HO
OH
OH

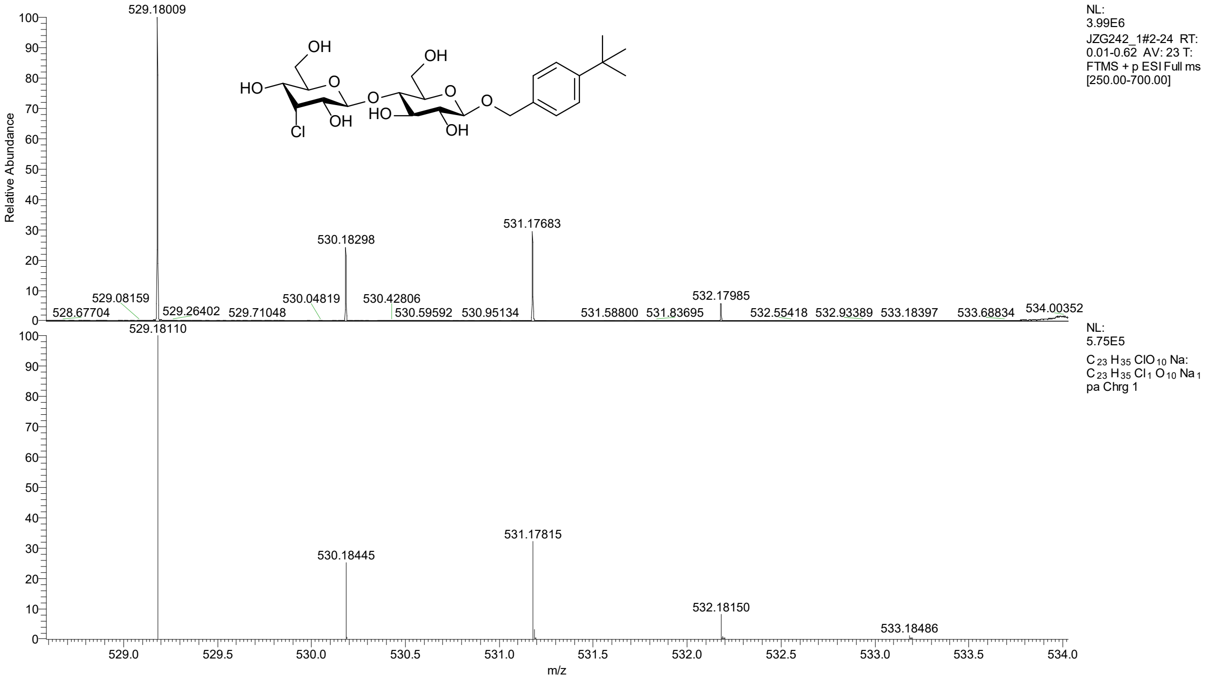
529.18009
529.08159
528.67704
529.26402
529.71048
530.04819
530.18298
530.42806
530.59592
530.95134
531.17683
531.58800
531.83695
532.17985
532.55418
532.93389
533.18397
533.68834
534.00352
529.18110
530.18445
531.17815
532.18150
533.18486
NL:
3.99E6
JZG242_1#2-24 RT:
0.01-0.62 AV: 23 T:
FTMS + p ESI Full ms
[250.00-700.00]
NL:
5.75E5
C 23 H35 ClO10 Na:
C 23 H35 Cl1 O10 Na1
pa Chrg 1
Relative Abundance
m/z

HRMS spectra of maltose derivatives

2,3,6,2',3',4',6'-Hepta-*O*-acetyl-maltose (S7)

Measured with ESI pos.

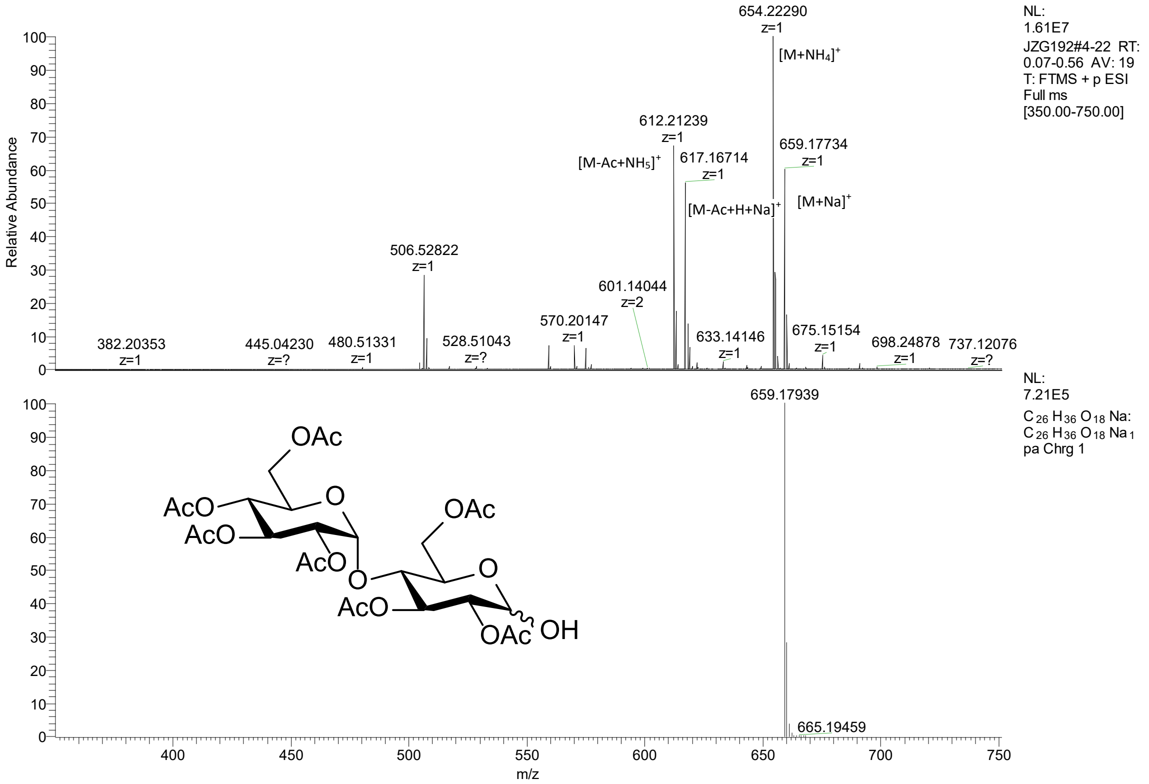

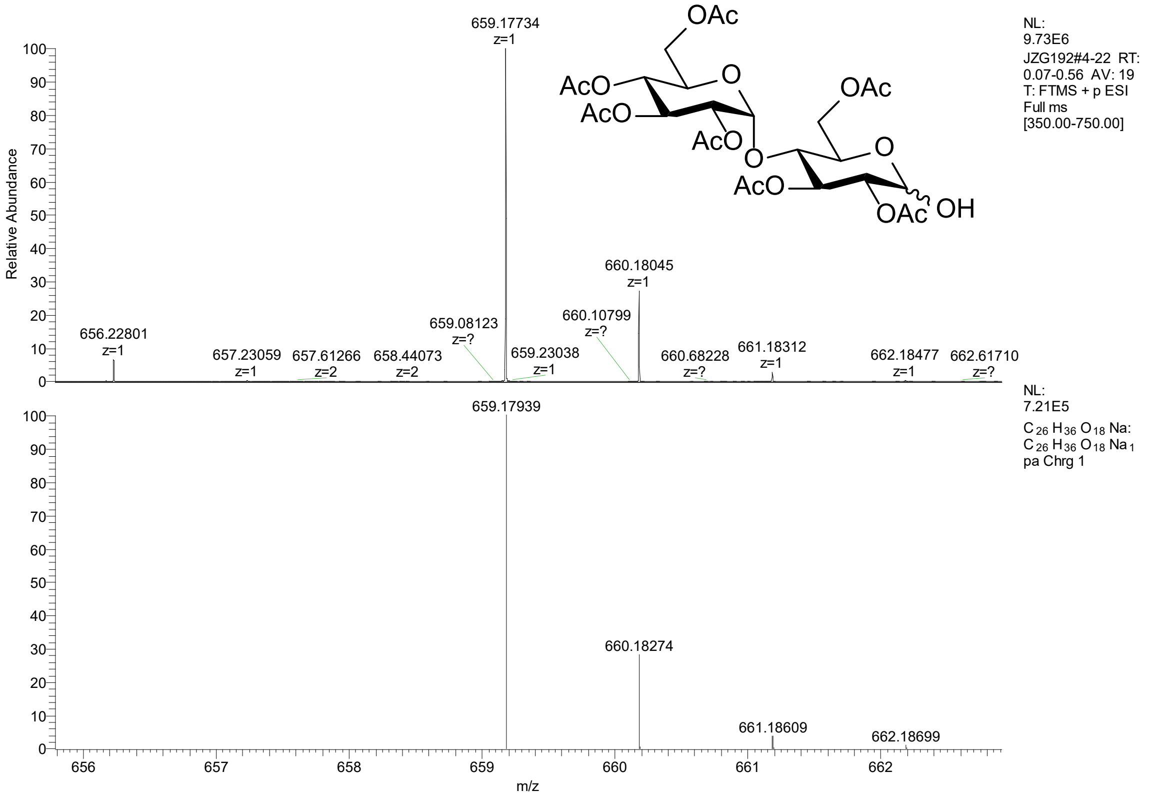

4-tert-butylbenzyl-β-D-heptaacetyl-maltoside (S8)

Measured with ESI pos.

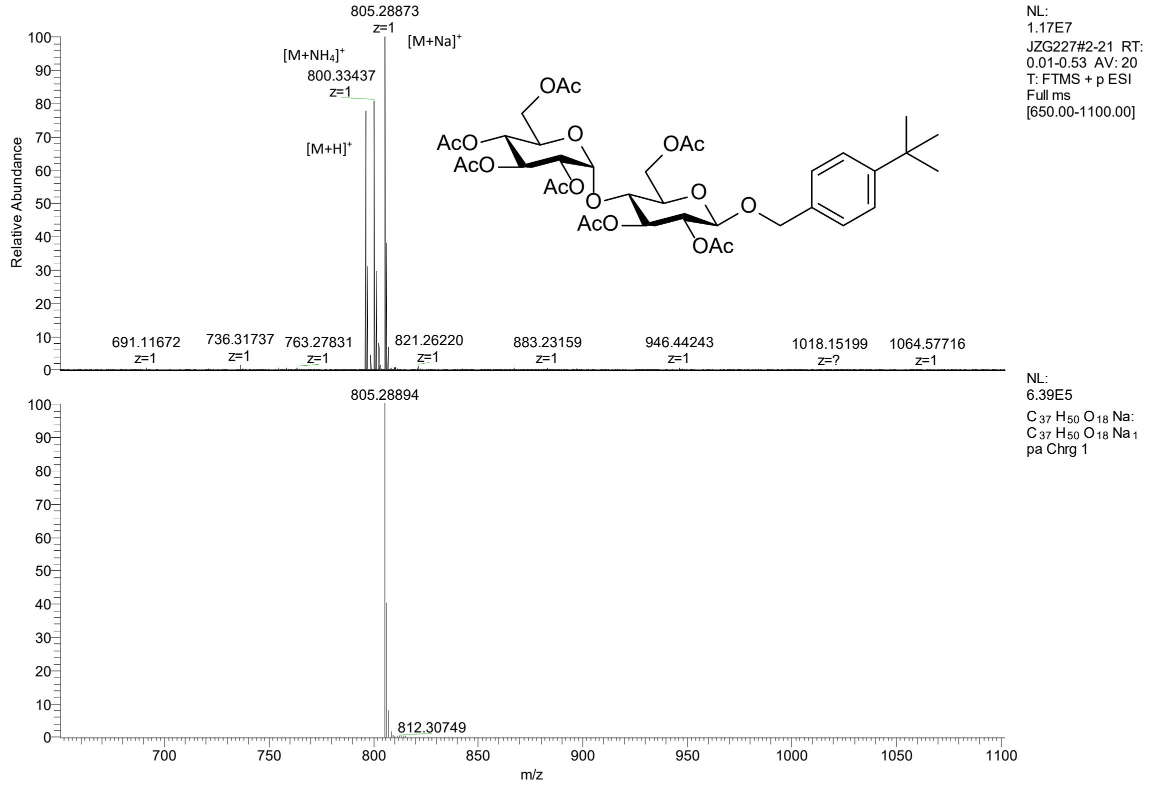

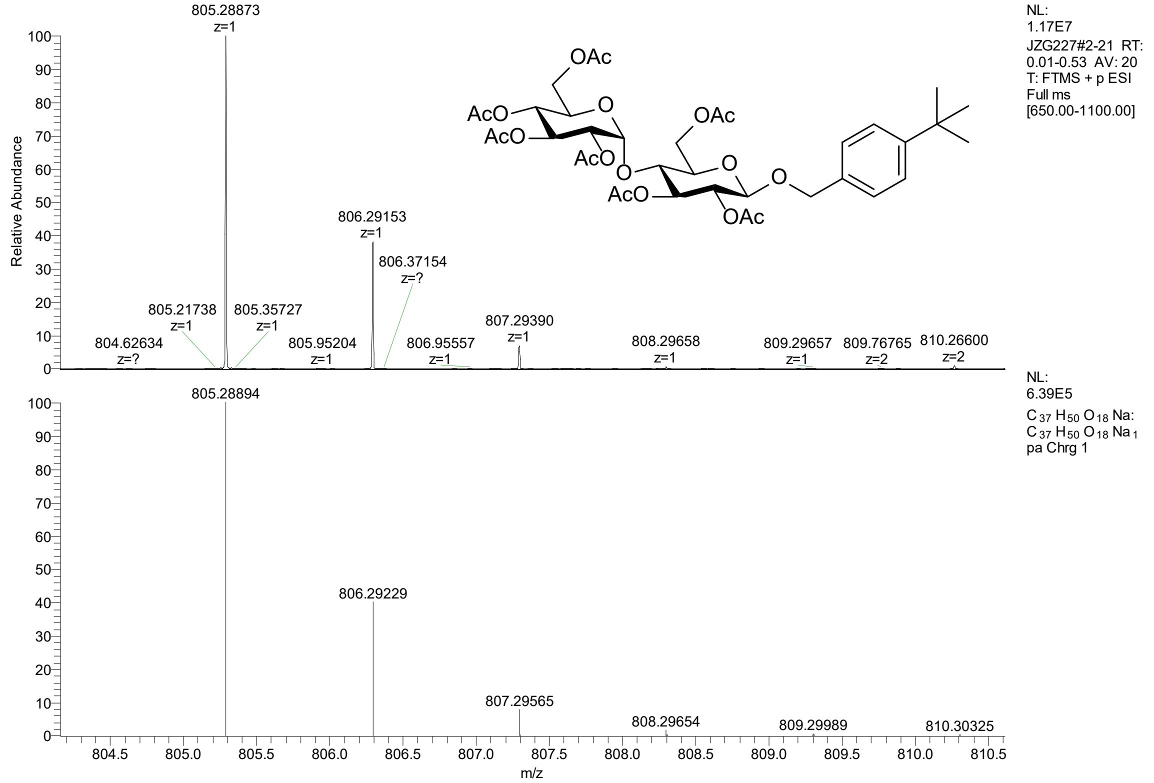

4-tert-butylbenzyl-β-ᴅ-maltoside (S9)

Measured with ESI pos.

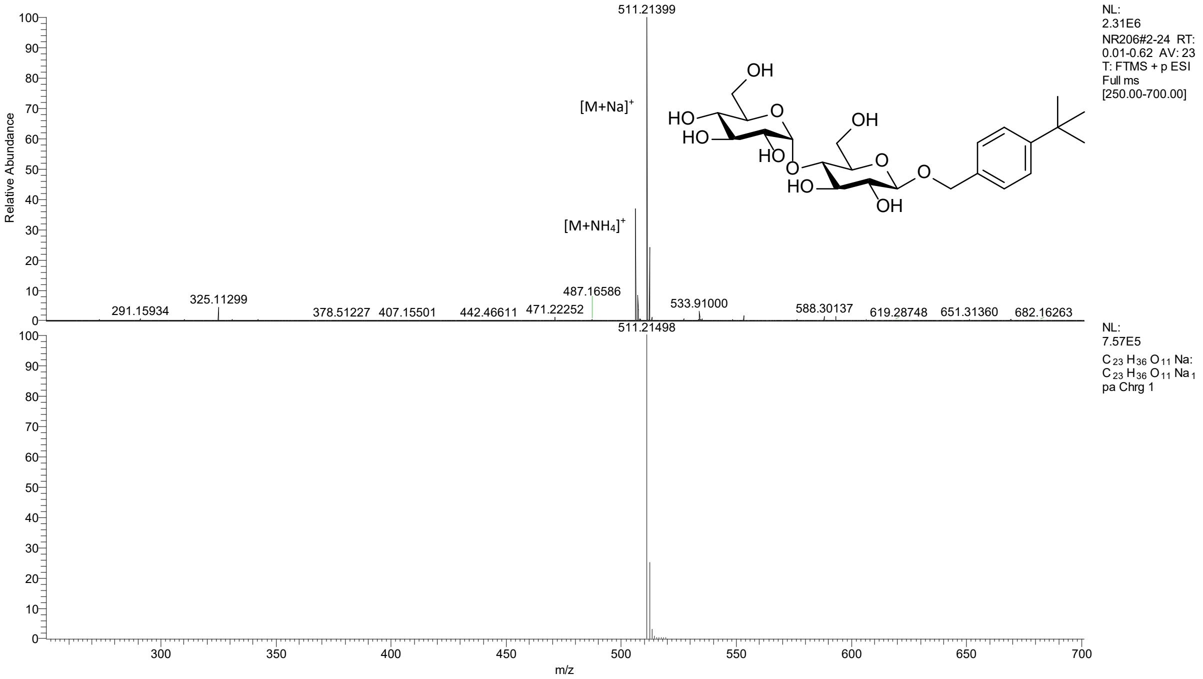

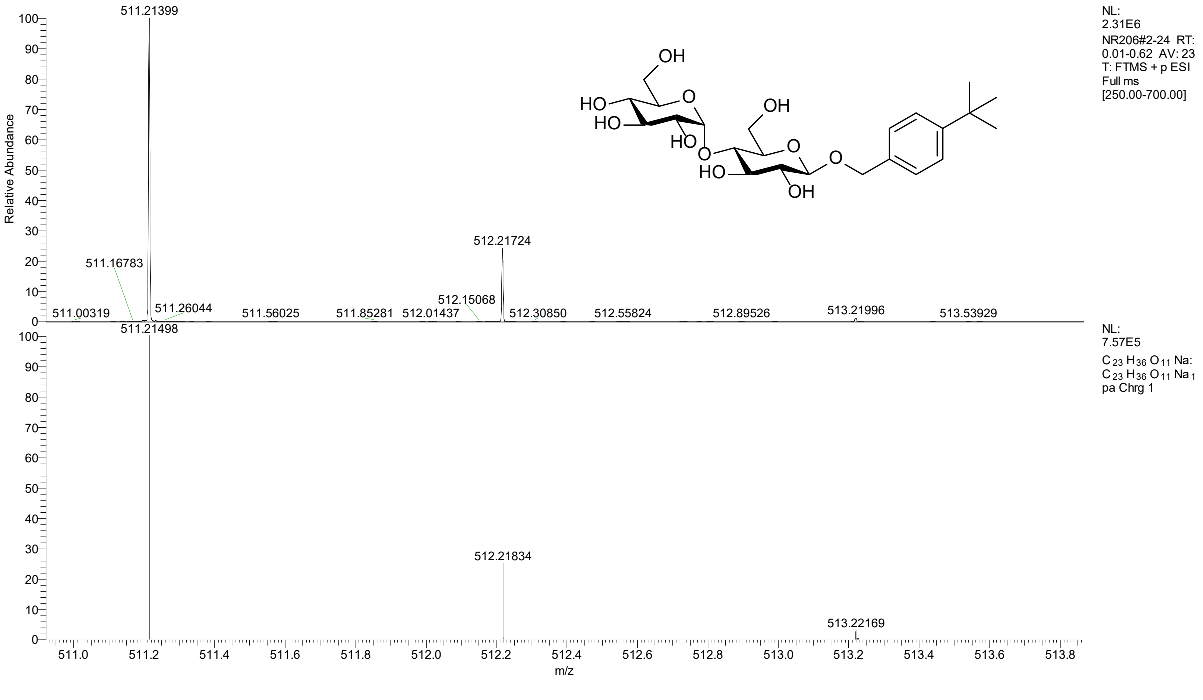

4-tert-butylbenzyl-β-3-ketomaltoside (1f)

Measured with ESI pos.

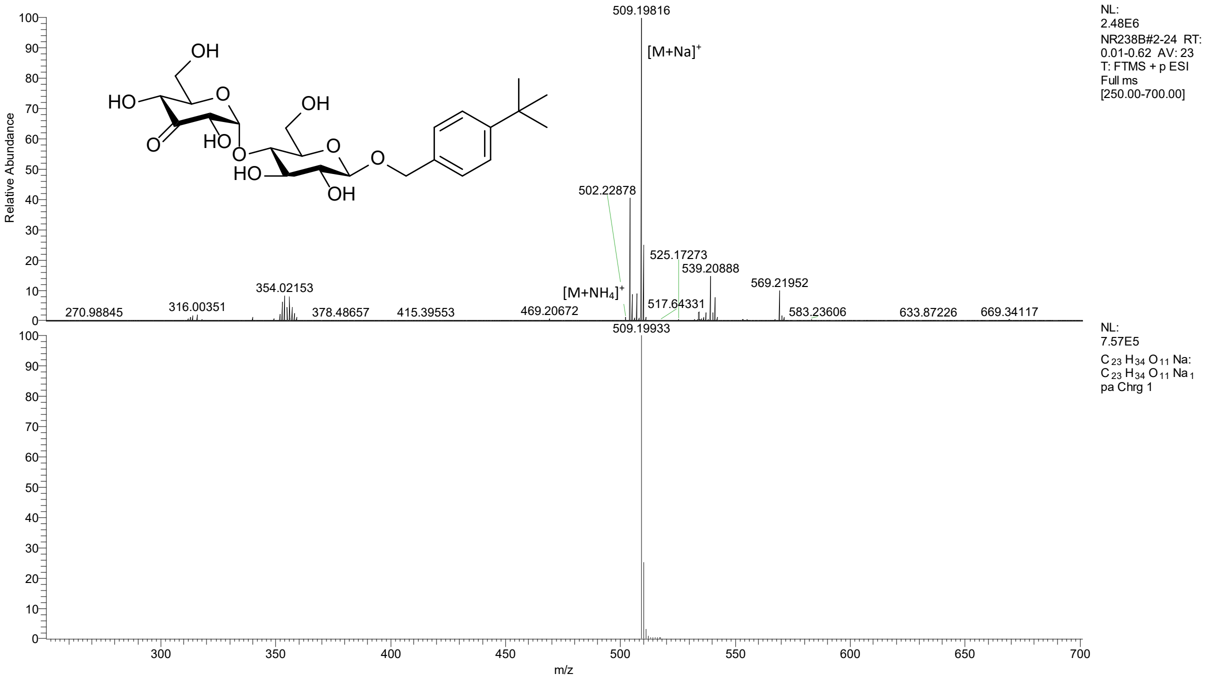

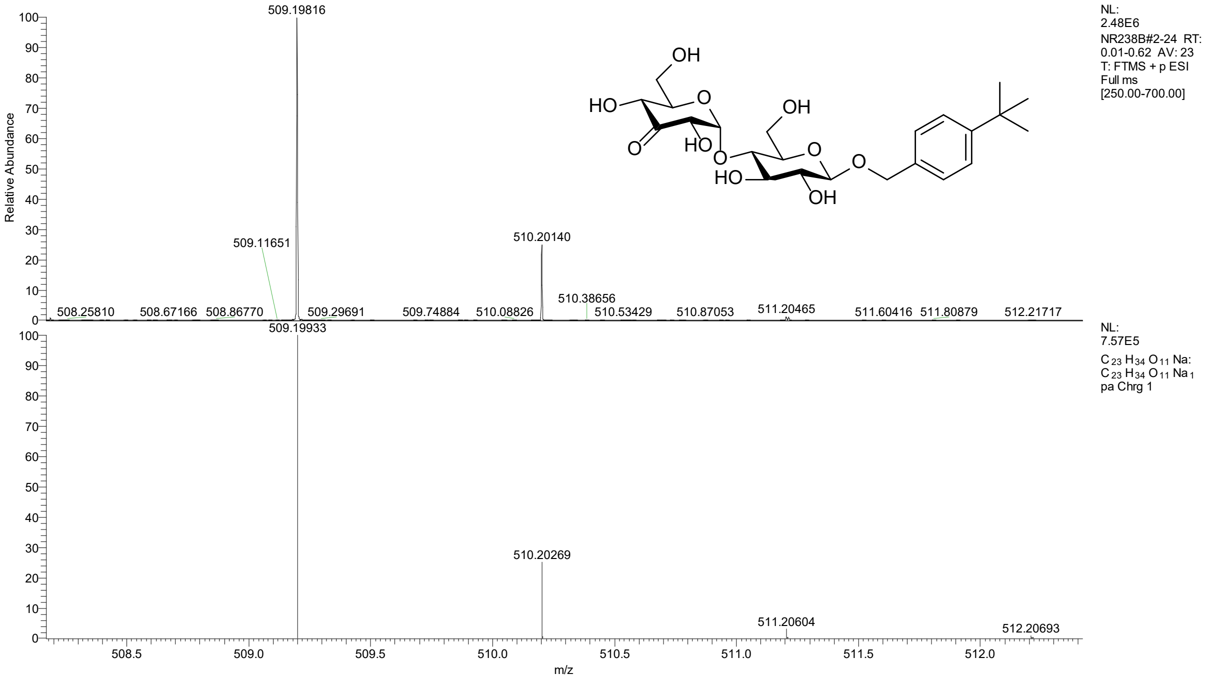

4-tert-butylbenzyl-β-3-(trityl)hydrazone maltoside (2f)

Measured with ESI pos.

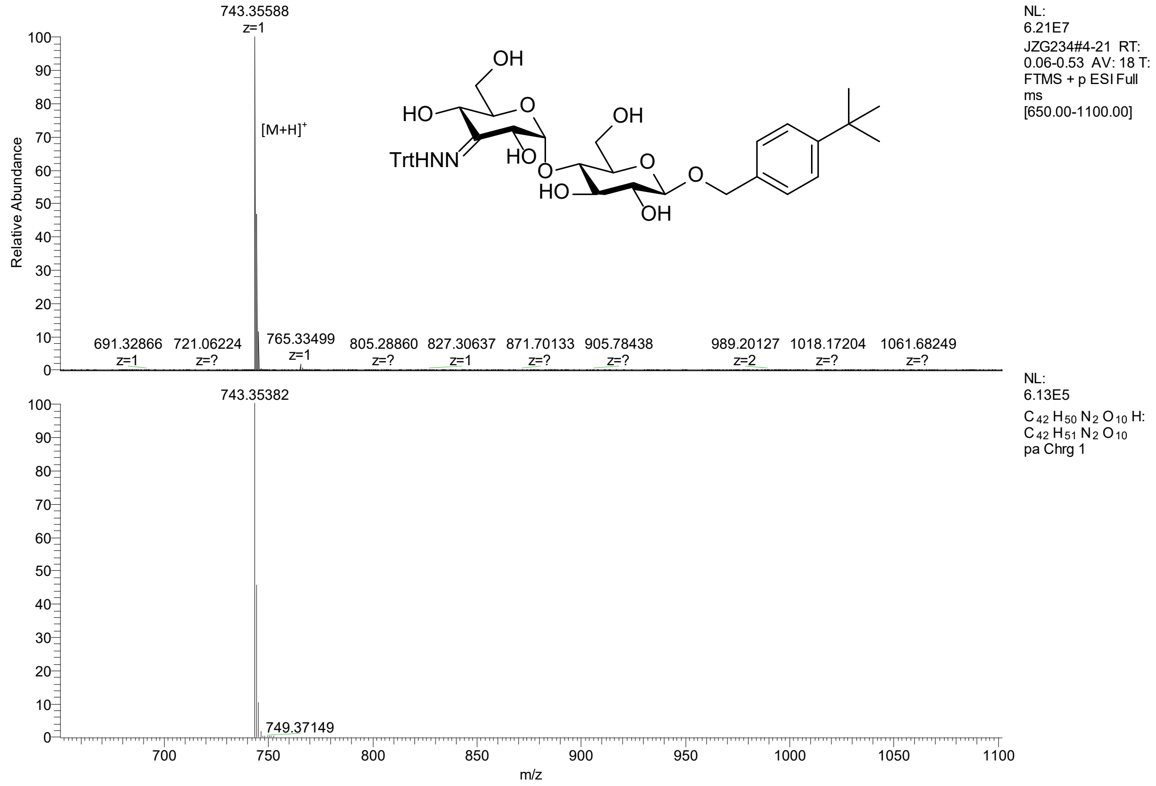

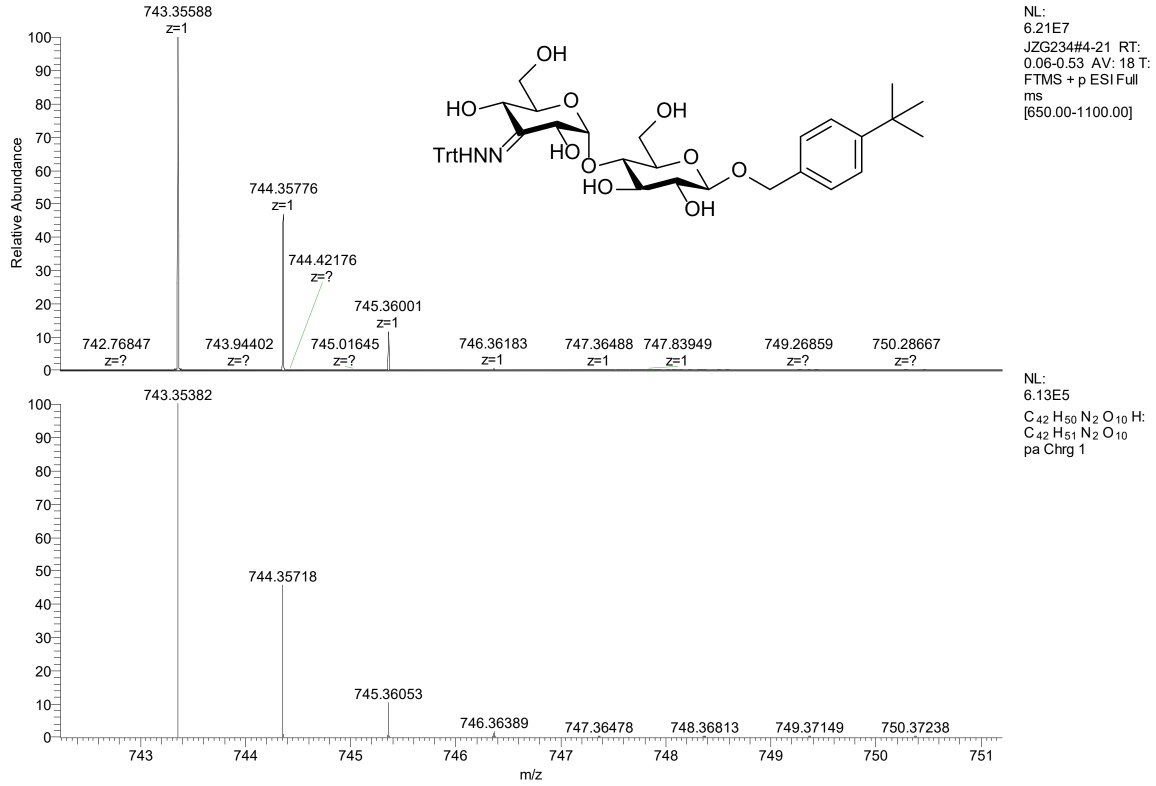

4-tert-butylbenzyl-3-chloro-3-deoxy-β-D-maltoside (3f)

Measured with ESI pos.

Equatorial:

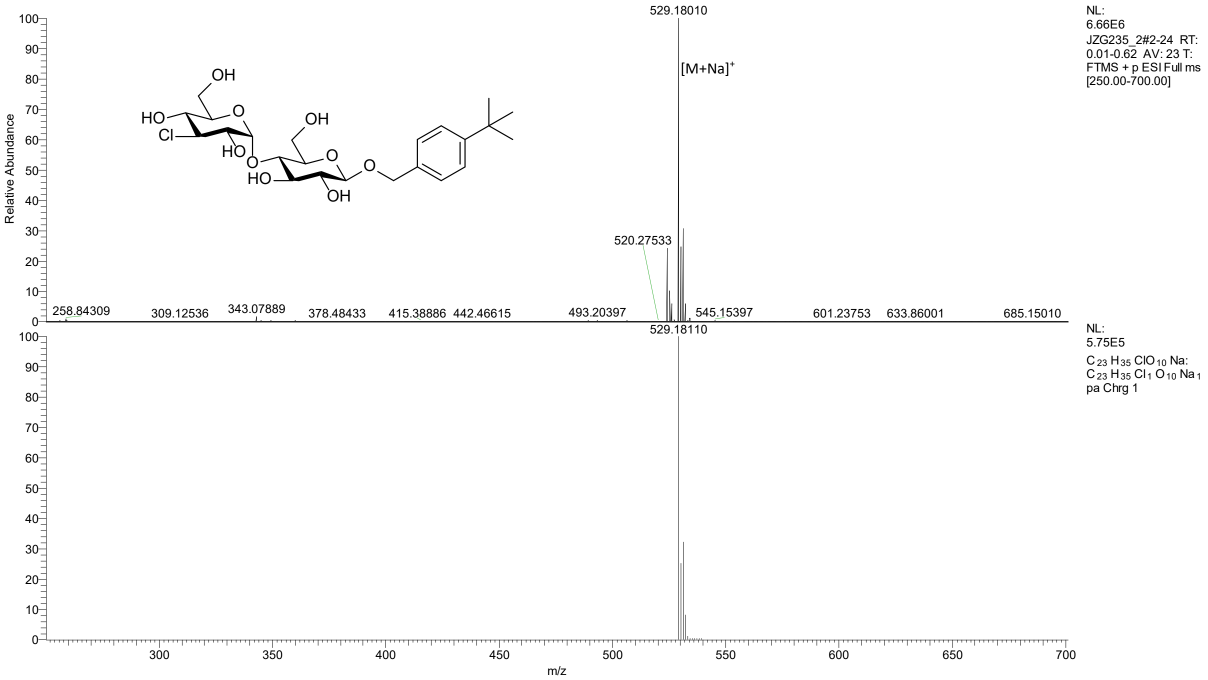

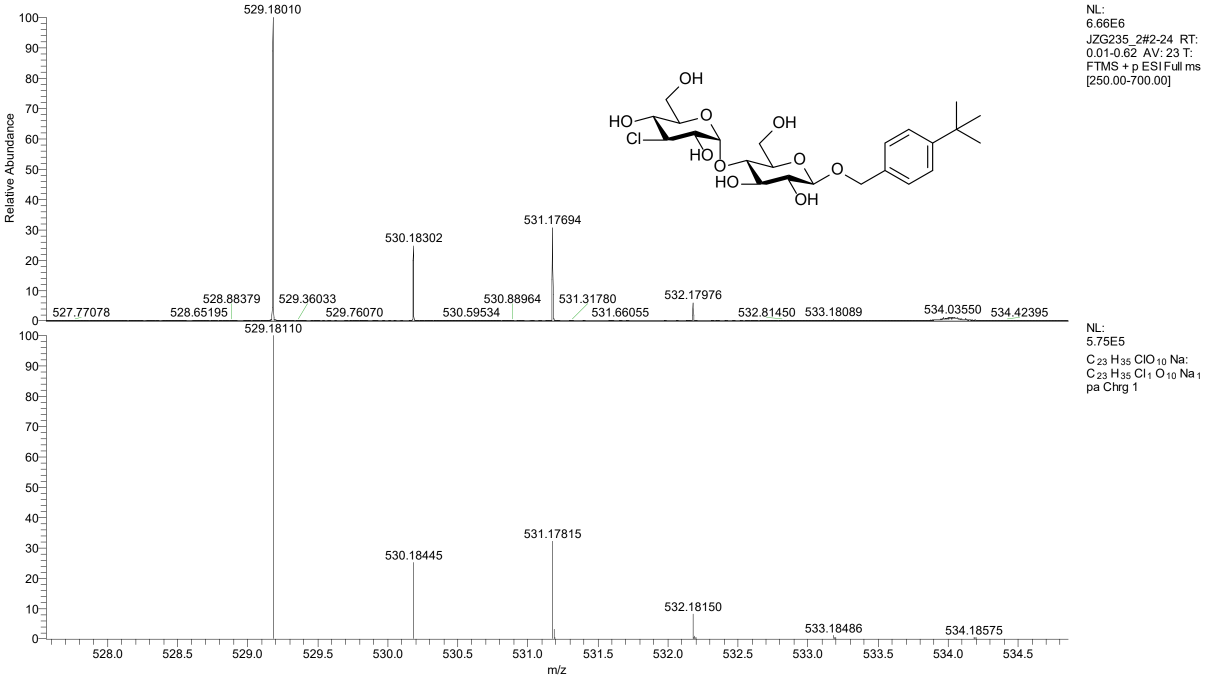

Axial:

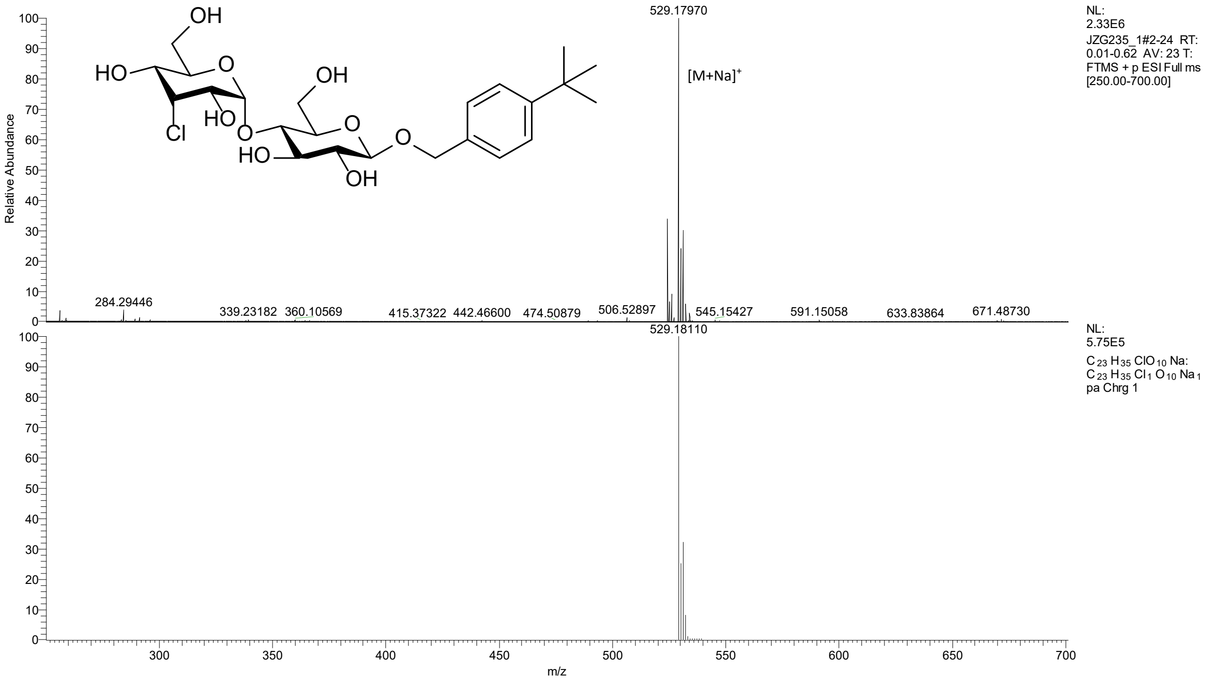
529.17970
[M+Na]+
NL: 2.33E6
JZG235_1#2-24 RT: 0.01-0.62 AV: 23 T: FTMS + p ESI Full ms [250.00-700.00]
284.29446
339.23182
360.10569
415.37322
442.46600
474.50879
506.52897
545.15427
591.15058
633.83864
671.48730
529.18110
NL: 5.75E5
C 23 H 35 ClO 10 Na: C 23 H 35 Cl 1 O 10 Na 1 pa Chrg 1
Relative Abundance
m/z
OH
HO
O
Cl
HO
O
OH
HO
OH
O
O

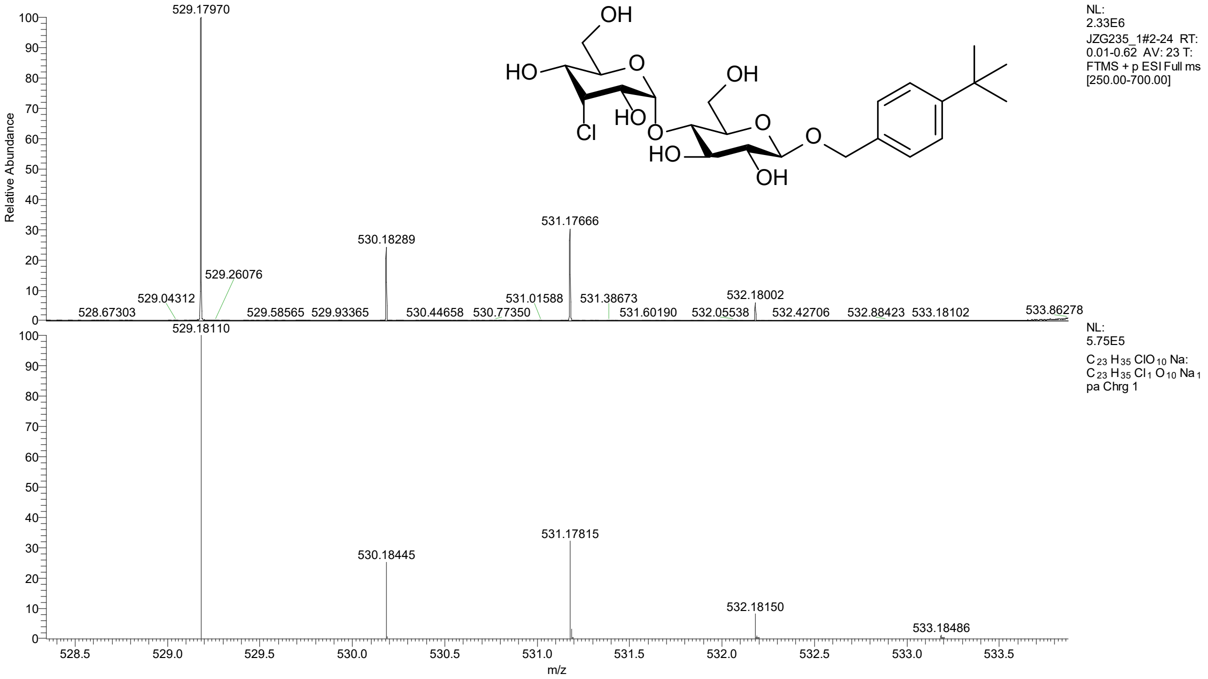
529.17970
NL: 2.33E6
JZG235_1#2-24 RT: 0.01-0.62 AV: 23 T: FTMS + p ESI Full ms [250.00-700.00]
531.17666
530.18289
529.26076
529.04312
528.67303
529.58565
529.93365
530.44658
530.77350
531.01588
531.38673
531.60190
532.18002
532.05538
532.42706
532.88423
533.18102
533.86278
529.18110
NL: 5.75E5
C 23 H 35 ClO 10 Na: C 23 H 35 Cl 1 O 10 Na 1 pa Chrg 1
531.17815
530.18445
532.18150
533.18486
Relative Abundance
m/z

HRMS spectra of β-Glc derivatives

Methyl 3-keto-β-D-glucopyranoside (1g)

Measured with ESI neg. in the presence of guanidinium chloride.

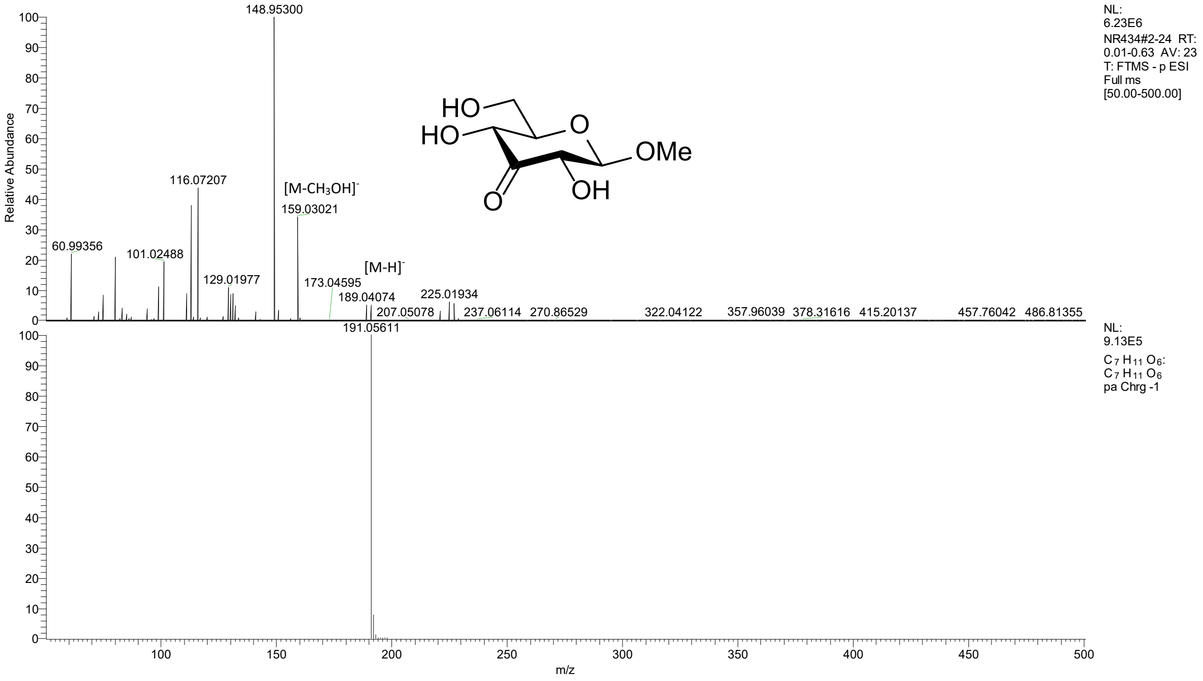

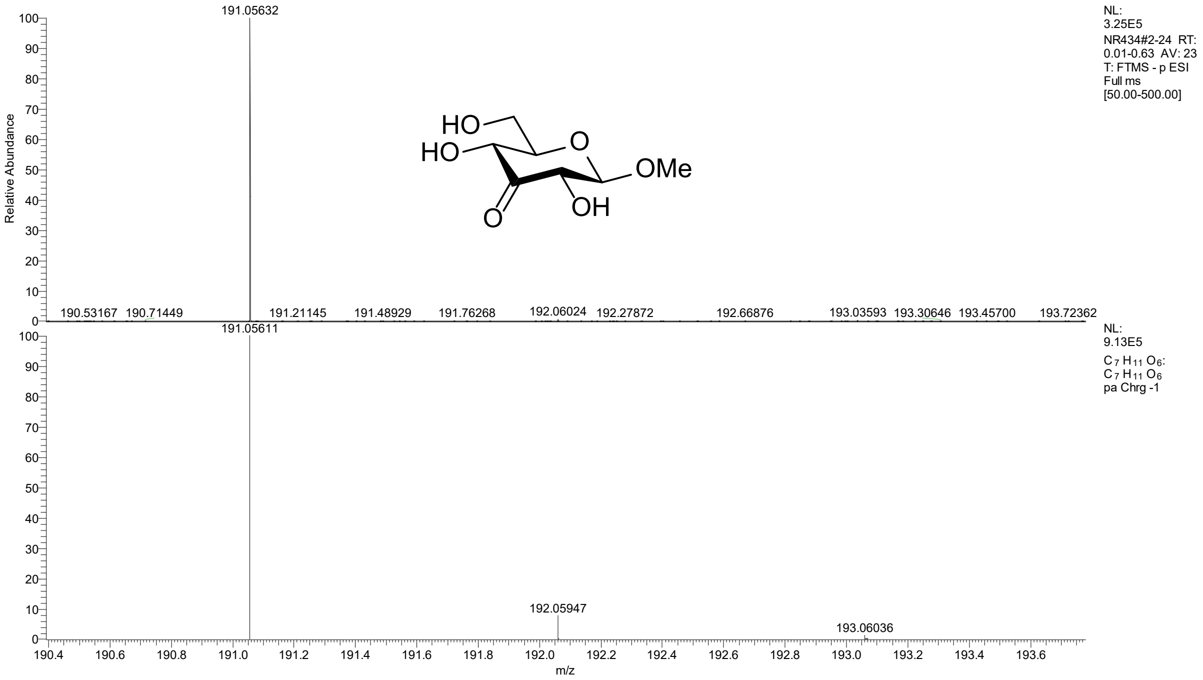

Methyl-3-(trityl)hydrazone-β-D-glucopyranoside (2g)

Measured with ESI pos.

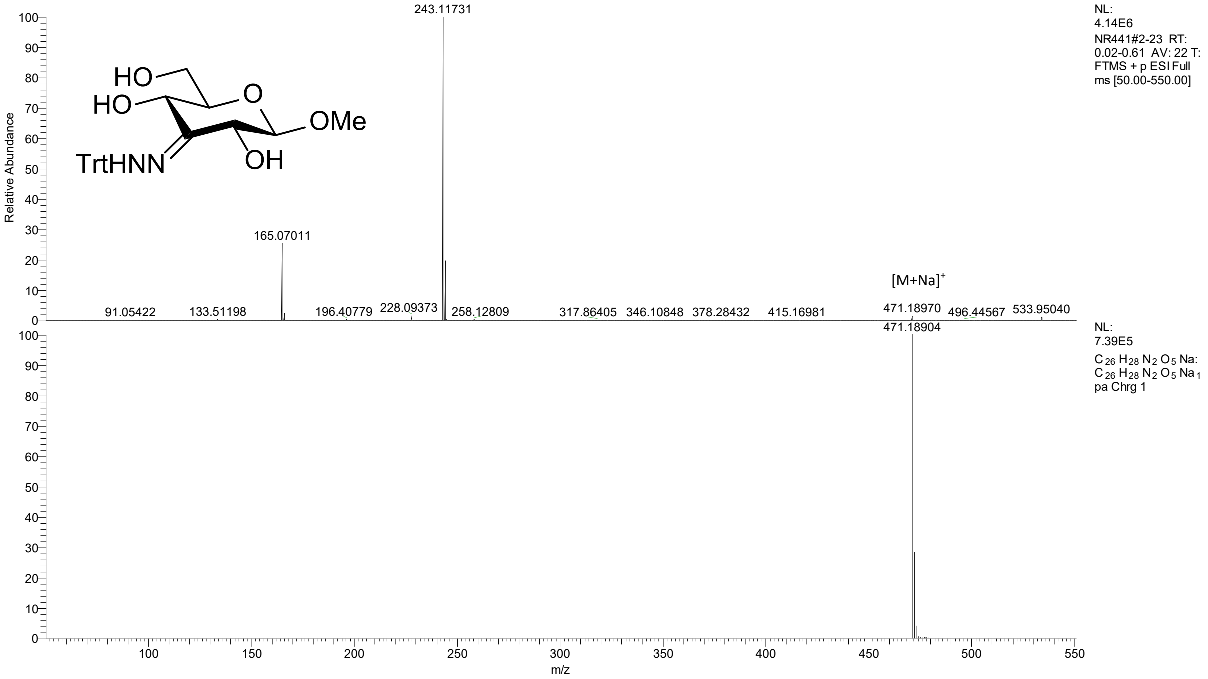

*243 and 165 come from remaining trityl material. The trityl cation causes ion suppression.

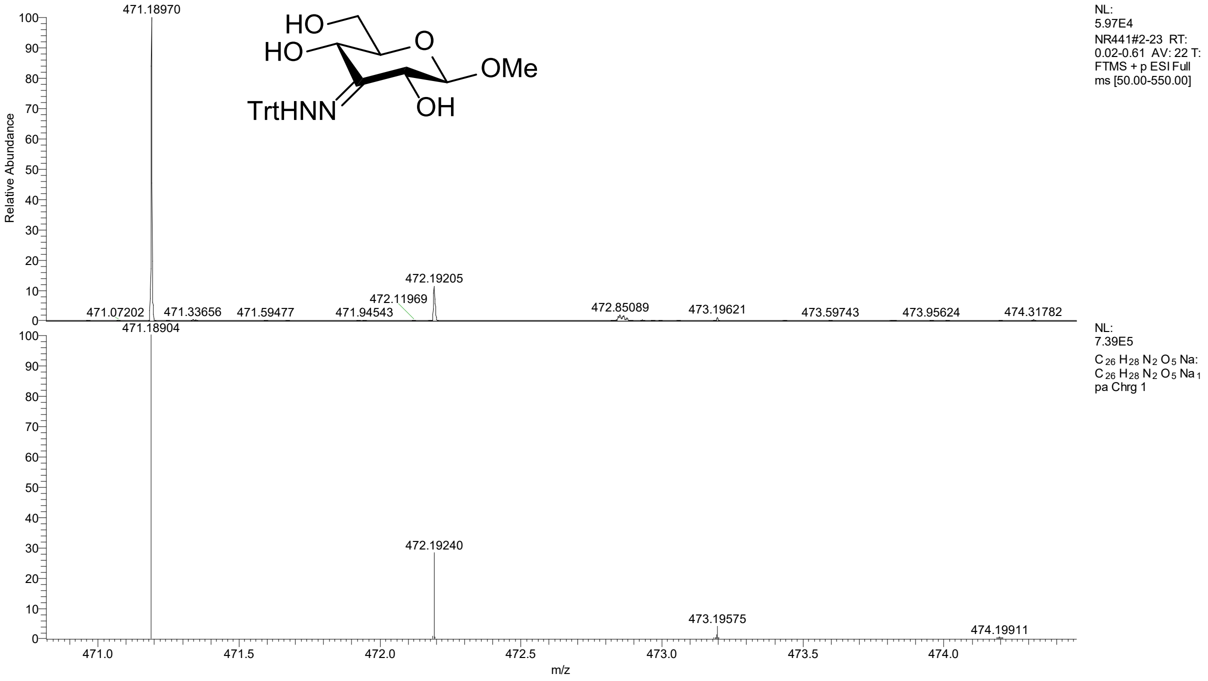

Methyl 3-chloro-3-deoxy-β-D-allo/glucopyranoside (3g)

Measured with ESI neg. in the presence of guanidinium chloride.

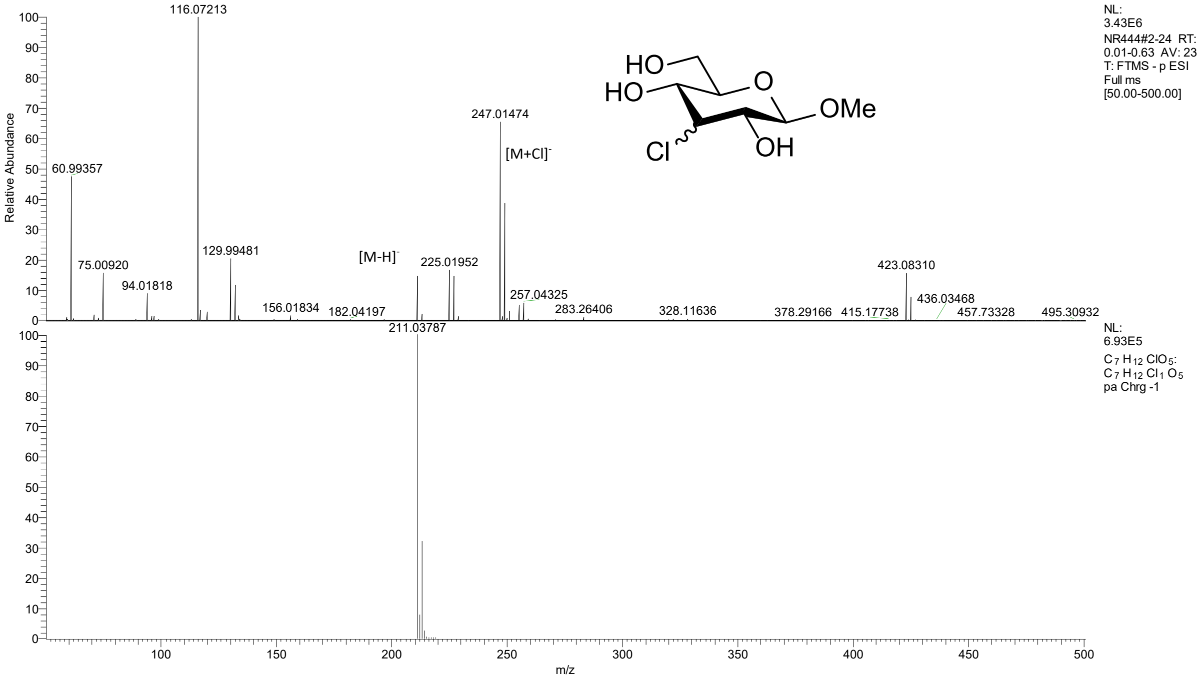

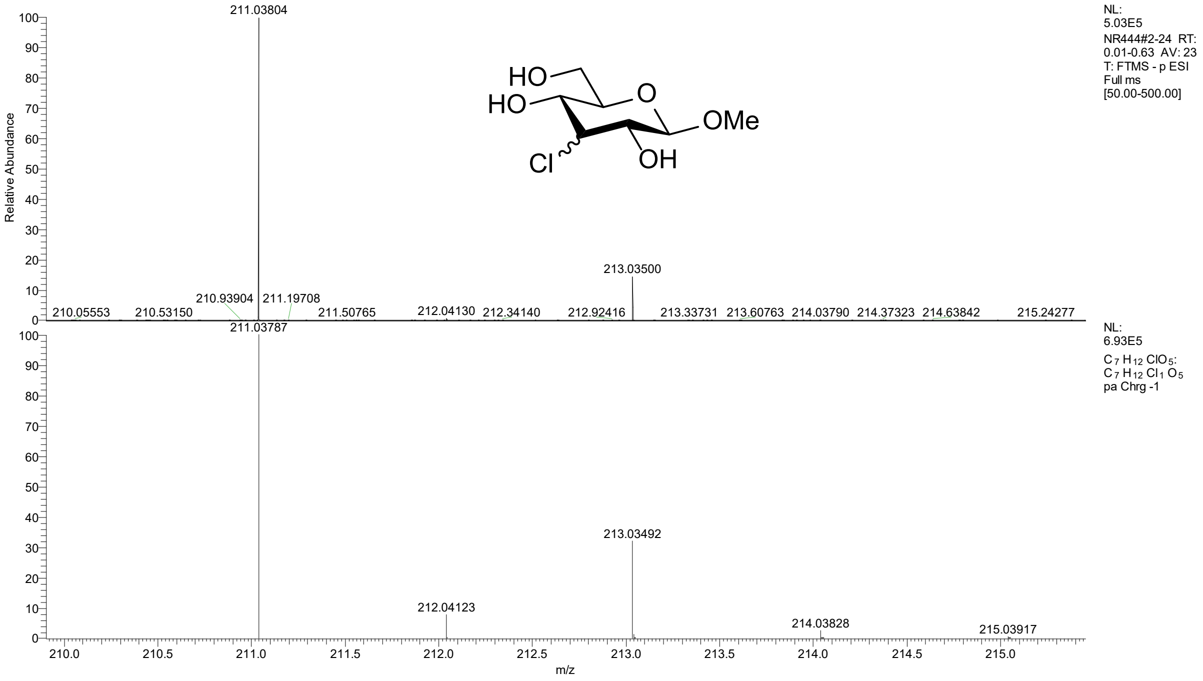

Methyl-3-(2,4-dinitrophenyl)hydrazone-β-D-glucopyranoside (6b)

Measured with ESI pos.

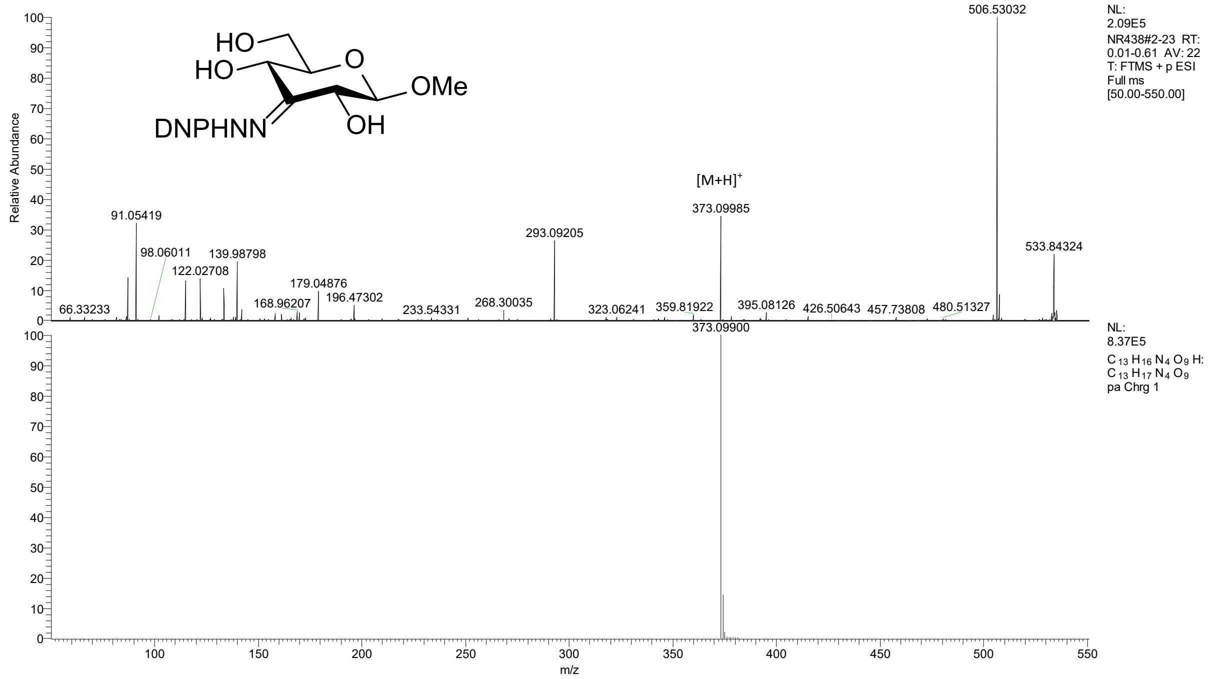

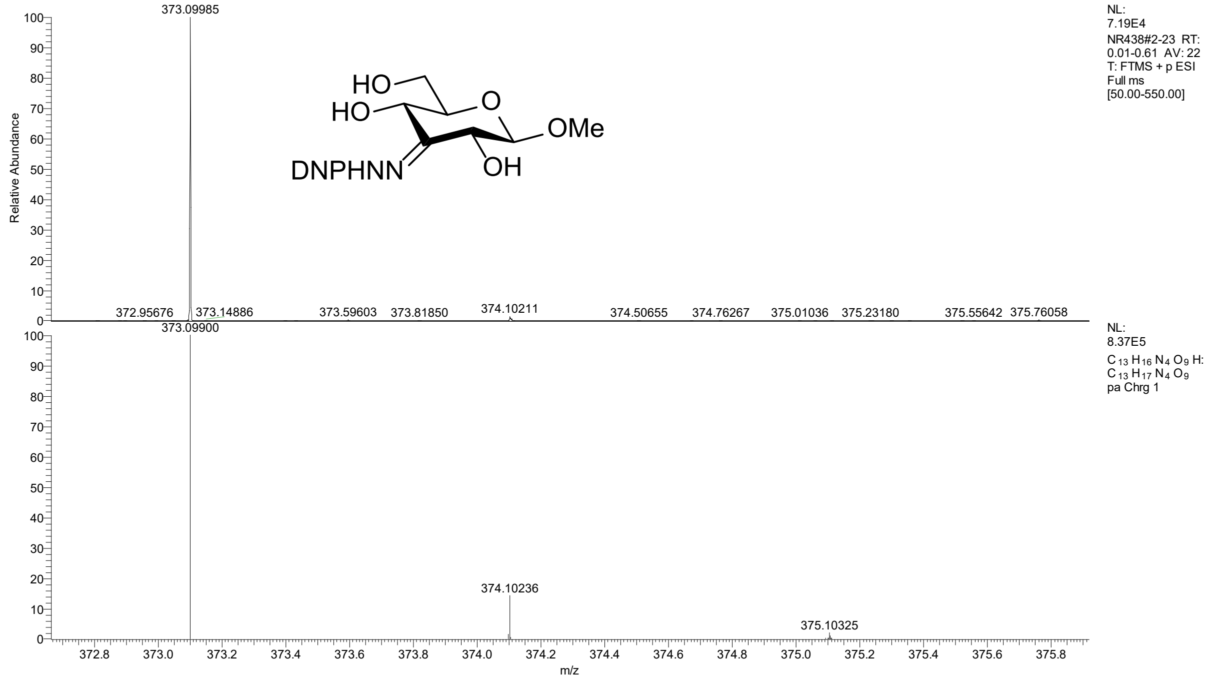

Methyl-3-chloro-3-(2,4-dinitrophenyl)diazene-β-D-glucopyranoside (7b)

Measured with ESI neg. in the presence of guanidinium chloride.

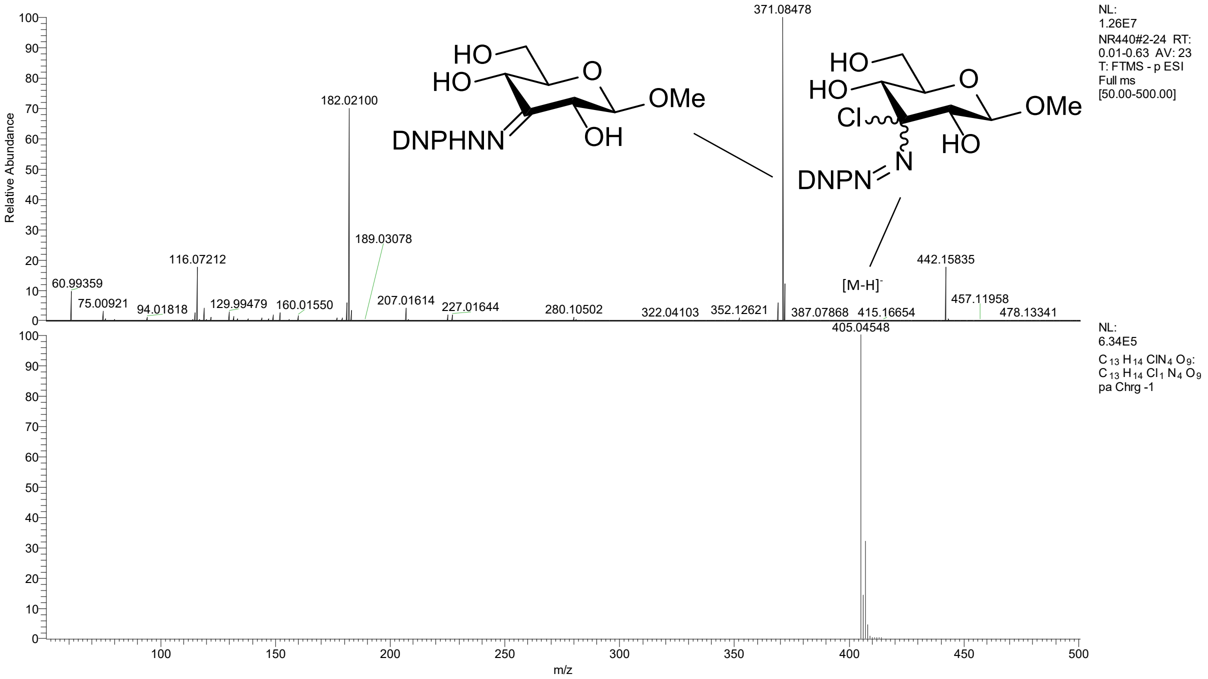

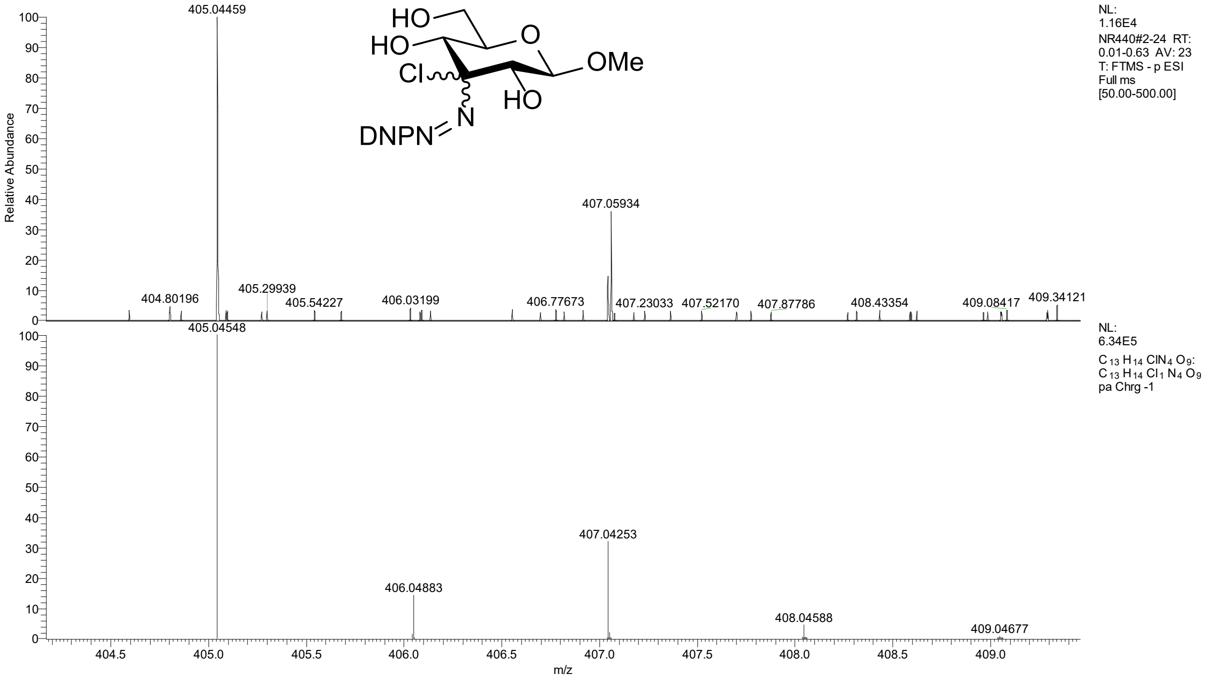

Supplement: Supplementary file 1 — ol2c01992_si_001.pdf [file ol2c01992_si_001.pdf]
